# A diversity-oriented synthesis strategy enabling the combinatorial-type variation of macrocyclic peptidomimetic scaffolds†
†Electronic supplementary information (ESI) available: Experimental procedures, characterization data and details of the computational analyses. See DOI: 10.1039/c5ob00371g



**DOI:** 10.1039/c5ob00371g

**Published:** 2015-03-17

**Authors:** Albert Isidro-Llobet, Kathy Hadje Georgiou, Warren R. J. D. Galloway, Elisa Giacomini, Mette R. Hansen, Gabriela Méndez-Abt, Yaw Sing Tan, Laura Carro, Hannah F. Sore, David R. Spring

**Affiliations:** a Department of Chemistry , University of Cambridge , Lensfield Road , Cambridge , CB2 1EW , UK . Email: spring@ch.cam.ac.uk ; Fax: +44 (0)1223-336362 ; Tel: +44 (0)1223-336498

## Abstract

Macrocyclic peptidomimetics are associated with a broad range of biological activities. However, despite such potentially valuable properties, the macrocyclic peptidomimetic structural class is generally considered as being poorly explored within drug discovery. This has been attributed to the lack of general methods for producing collections of macrocyclic peptidomimetics with high levels of structural, and thus shape, diversity. In particular, there is a lack of scaffold diversity in current macrocyclic peptidomimetic libraries; indeed, the efficient construction of diverse molecular scaffolds presents a formidable general challenge to the synthetic chemist. Herein we describe a new, advanced strategy for the diversity-oriented synthesis (DOS) of macrocyclic peptidomimetics that enables the combinatorial variation of molecular scaffolds (core macrocyclic ring architectures). The generality and robustness of this DOS strategy is demonstrated by the step-efficient synthesis of a structurally diverse library of over 200 macrocyclic peptidomimetic compounds, each based around a distinct molecular scaffold and isolated in milligram quantities, from readily available building-blocks. To the best of our knowledge this represents an unprecedented level of scaffold diversity in a synthetically derived library of macrocyclic peptidomimetics. Cheminformatic analysis indicated that the library compounds access regions of chemical space that are distinct from those addressed by top-selling brand-name drugs and macrocyclic natural products, illustrating the value of our DOS approach to sample regions of chemical space underexploited in current drug discovery efforts. An analysis of three-dimensional molecular shapes illustrated that the DOS library has a relatively high level of shape diversity.

## Abstract 2

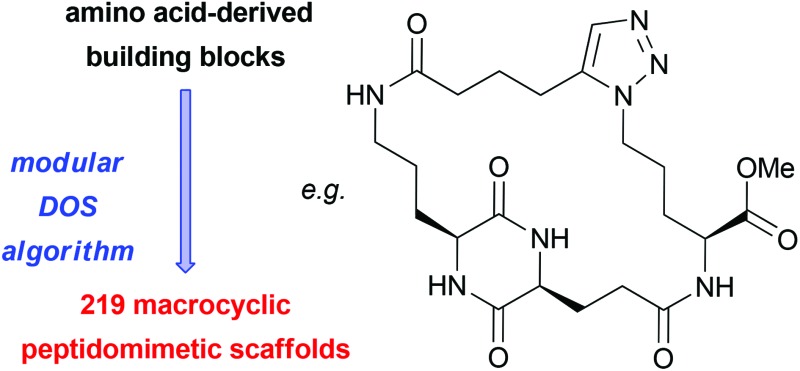
Macrocyclic peptidomimetics are associated with a broad range of biological activities.

## Introduction

**Fig. 1:**
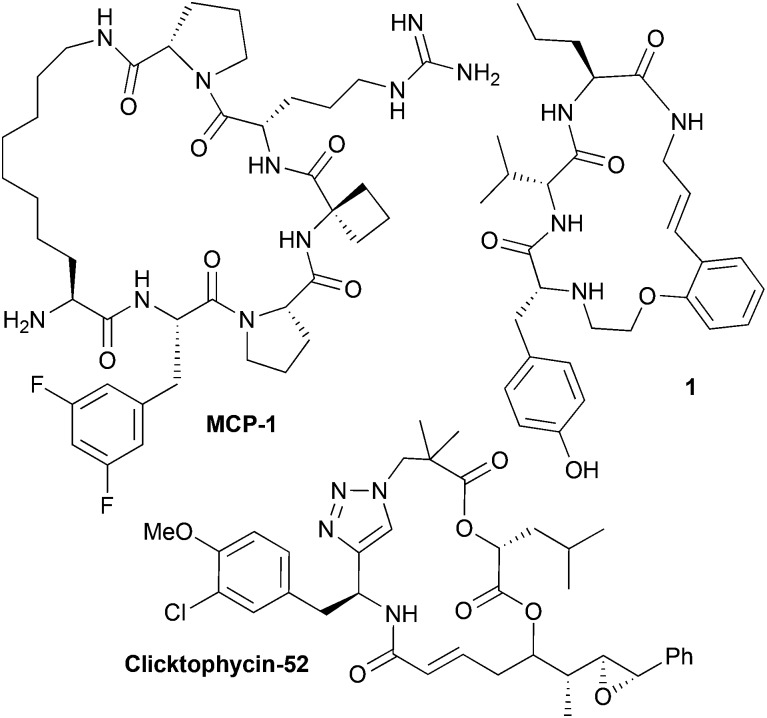
Chemical structures of some biologically active macrocyclic peptidomimetics. MCP-1 is a potent inhibitor of the menin-mixed lineage leukemia 1 protein–protein interaction,^17^ Clicktophycin-52 displays *in vitro* activity against the multidrug resistant human cervix carcinoma cell line KB-V1^8^ and compound **1** is a motilin antagonist.^18^

Macrocycles are ring structures of 12 or more atoms. Where present in a small molecule, the macrocyclic ring architecture typically serves as the molecular scaffold; that is, the core rigidifying structural feature of a molecule that has the largest influence upon how it presents its chemical information (*i.e.* functional groups and potential binding regions) in three dimensions (3D).^1–4^ Macrocyclic peptidomimetics are a sub-class of macrocycles, designed to act as functional substitutes for peptide motifs or proteins, whilst having more desirable biological properties.^4,5^ For example, this can involve the incorporation of an isosteric analogue of a peptide linkage into the macrocyclic ring structure.^4,5^ Many macrocyclic peptidomimetic compounds are known to be capable of modulating biological systems (Fig. 1).^1,2,6–12^ However, despite such potentially valuable properties, macrocyclic peptidomimetics are considered to be a relatively poorly explored structural class within drug discovery.^1,2,9,13^ This can be attributed to a lack of general methods for producing macrocyclic peptidomimetics collections with broad structural (chemical) diversity and thus functional diversity (that is, displaying a broad range of biological activities).^9^ In the context of efficient exploration of the biological capabilities of this compound type, the key aspect of structure is the molecular scaffold (that is, the macrocyclic ring architecture).^14^ It is generally acknowledged that increasing the scaffold diversity in a small molecule library is one of the most effective ways of increasing its overall functional diversity.^14,15^ This has been attributed to a correlation between the scaffold diversity of a compound set and its molecular shape diversity.^14,15^ The overall shape of a molecule is the most fundamental factor controlling its biological effects, and this is intrinsically linked to the molecular scaffold that the molecule possesses.^14–16^ Substantial ‘shape space coverage’ (that is, molecular shape diversity) has been correlated with broad biological activity, and the scaffold diversity of any compound set has a pivotal role in defining its overall molecular shape diversity, with peripheral substituents being of considerably less importance in this regard.^14–16^

There are synthetic methods that can provide collections of macrocyclic peptidomimetics with varying appendages based around similar core scaffolds (core macrocyclic ring architectures), typically designed to modulate a specific biological target or family of related targets.^9–11,19–23^ However, there is a relative dearth of synthetic strategies to produce libraries of macrocyclic peptidomimetics with high scaffold, and thus functional, diversity. Indeed, the efficient construction of diverse molecular scaffolds presents a formidable general challenge to the synthetic chemist.^14,24^

Over the course of the last decade, diversity-oriented synthesis (DOS) has established itself as a field of organic chemistry directed towards the generation of structurally, and thus functionally, diverse small molecule libraries.^14^ DOS involves the conversion of simple and similar starting material(s) to more complex and structurally diverse molecules through relatively short synthetic sequences, with an emphasis typically placed upon the efficient incorporation of multiple molecular scaffolds in the library.^14^ Many ingenious DOS strategies have been reported which have enabled the efficient synthesis of libraries based on tens (up to 82)^25^ of different molecular scaffolds.^14^ Recent years have seen the development of several DOS-type strategies targeted at macrocyclic structures,^3,26,27^ including macrocyclic peptidomimetics in particular.^28^ Notable recent examples come from the research groups of Wessjohann,^28,29^ Marcaurelle,^30^ Marsault^9^ and Harran.^31^ Overall however, there still remains a relative lack of DOS strategies that are specifically directed towards macrocyclic peptidomimetics. Reports beyond the proof-of-principle stage (that is, involving the application of such DOS strategies for the generation of large numbers of structurally diverse compounds based around a variety of macrocyclic peptidomimetic ring architectures) are rare.^9,18,28^

**Scheme 1:**
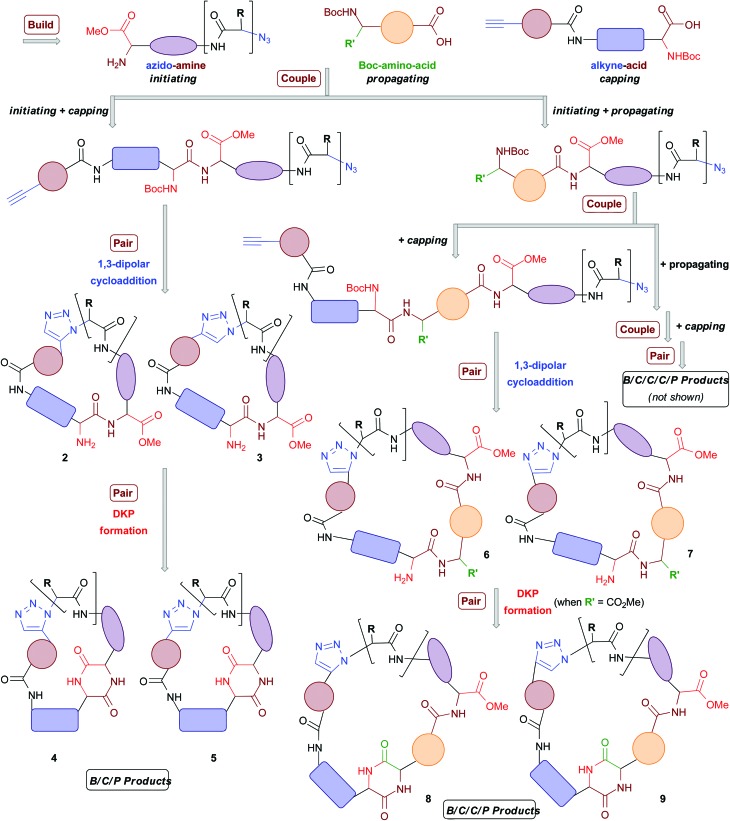
Outline of our advanced DOS strategy towards the combinatorial variation of macrocyclic peptidomimetic scaffolds. The coloured shapes represent major scaffold-defining elements (*i.e.* areas that can be varied to obtain different macrocyclic ring architectures).

A common synthetic algorithm underpinning many DOS pathways is the so-called build/couple/pair (B/C/P) three-phase strategy.^32^ The *build* phase involves the synthesis of starting materials (or building blocks). These are then coupled together in the *couple* phase to produce densely functionalized molecules. In the *pair* phase intramolecular reactions that join pairwise combinations of functional groups incorporated in the build phase are performed to generate diverse molecular scaffolds. Recently, we reported the development of a DOS pathway towards macrocyclic peptidomimetics that was based around the B/C/P synthetic algorithm and two types of building blocks (Scheme 1).^4^ This approach was used to generate a small proof-of-concept library of 14 structurally diverse peptidomimetic compounds based around four different general structural types (**2–5** in Scheme 1). Each compound contained a triazole ring in place of an amide bond. The triazole moiety acts as a peptide bond mimic (hence peptidomimetic; both the *trans*- and the *cis*- amide bond configurations can be mimicked by the 1,4- and 1,5-triazoles, respectively).^4,33,34^ Several compounds also featured a diketopiperazine (DKP) motif in the macrocyclic framework; DKPs occur in numerous natural products and are of significant importance in drug discovery.^35–37^

Recently, Nelson and co-workers have reported a DOS strategy based around a modified B/C/P algorithm that incorporates triplets of building blocks and iterative *couple* steps.^38^ In this approach, “initiating” building blocks were coupled with “propagating” building blocks followed by “terminating” building blocks, in two successive couple phases, to furnish a diverse range of linear substrates for the subsequent pair phase. This synthetic sequence can be thus described as B/C/C/P, with the authors reporting the use of three “initiating”, four “propagating” and three “terminating” building blocks in the synthesis of approximately 14 different scaffolds (including one macrocyclic framework). We envisaged that this elegant concept could be leveraged in the design of an advanced DOS strategy towards macrocyclic peptidomimetics that also incorporated iterative couple steps (B/C/C/P and B/C/C/C/P, Scheme 1). This would allow for an increase in the diversity of macrocyclic peptidomimetic scaffolds synthetically accessible from a given set of building blocks relative to our previous B/C/P sequence involving a single *couple* stage. Herein, we report the successful development of this advanced DOS synthetic algorithm. Application of this approach has enabled the step-efficient synthesis of a library of over 200 compounds, each containing a distinct macrocyclic peptidomimetic scaffold and isolated in milligram quantities. To the best of our knowledge this is the first time that over 100 distinct scaffolds have been generated in a DOS library. Thus this work represents a step-change in the degree of scaffold diversity incorporated in a synthetically-derived small molecule library. Computational analyses indicated that the DOS library has a relatively high level of molecular shape diversity and also samples attractive regions of chemical space underexploited in current drug-discovery efforts.

## Results and discussion

### Outline of the synthetic strategy

Our advanced DOS strategy towards diverse macrocyclic peptidomimetic scaffolds was based around the use of three general types of chiral building blocks (generated in the build phase) of the synthesis: “azido amines” (“initiating” building blocks), “Boc-amino-acids” (“propagating” building blocks) and “alkyne-acids” (“capping” building blocks, Scheme 1).^38^ We envisaged that these building blocks could be combined as illustrated in Scheme 1 (in the couple phase) to yield linear peptides containing both a terminal alkyne and an azide. These functionalities could then potentially be reacted together in the pair phase to access diverse macrocyclic peptidomimetic scaffolds.

Specifically, the pair phase was to be comprised of two cyclization steps. First, a regioselective metal-catalysed “click”-type 1,3-dipolar cycloaddition, selectively generating 1,5-disubstituted triazoles (ruthenium catalysis) or 1,4-disubstituted triazoles (copper catalysis) with concomitant construction of the macrocyclic architecture (general structural types **2** and **3** respectively). The second cyclization step involves an intramolecular cyclization reaction between amine and carbonyl groups to introduce a DKP motif into the macrocyclic framework, leading to general structures **4** and **5**.^4^ In our previous study, 14 B/C/P macrocyclic peptidomimetics based on general structures **2–5** were generated *via* the initial coupling of an “initiating” building block with a “capping” building block in the first couple stage of the DOS.^4^ It was envisaged that larger B/C/C/P products of the general forms **6–9** could also be accessed if “initiating” building blocks were instead first coupled with “propagating” building blocks before being combined with a “capping” building block. Indeed, it was anticipated that iterative coupling with “propagating” building blocks to generate linear peptides of increasing length prior to the installation of a “capping” building block should be possible. For example, two rounds of coupling with “propagating” building blocks, followed by coupling with a “capping” building block and then the pair phase reactions should lead to so-called B/C/C/C/P products. Overall, we anticipated that this advanced DOS algorithm would allow efficient access to a large number of structurally diverse macrocyclic peptidomimetics from a small set of simple, readily available building blocks. In principle, a combinatorial-type variation of molecular scaffold should be possible, since the different building blocks could be assembled in varying combinations, with each different combination ultimately yielding a different macrocyclic scaffold. The DOS strategy should also allow for facile variation in the appendages (that is, the R and R′ groups and other exocyclic groups present in the scaffold-defining elements in Scheme 1), and thus functionality, displayed around the core scaffolds through the use of a range of “initiating” and “propagating” building blocks (derived from a variety of natural and non-natural amino acids). Thus, there is scope for achieving, in a compound efficient fashion, high levels of diversity across the library in both the nature of chemical information present and how it is displayed in 3D. This may be important for any subsequent biological screening of the DOS library compounds, as the nature of the appendages around macrocyclic scaffolds can have a profound effect upon biological activities (for example, it is well know that the sugar appendages in erythromycin and other antibacterial macrocycles are typically vital for the antibacterial activity of such compounds^39^). In addition, the macrocycles resulting from iterative coupling sequences (B/C/C/P, B/C/C/C/P, *etc*.) would be larger in size than those generated using a single couple phase. Consequently, they may be better suited to targeting extended binding interfaces, such as those associated with protein–protein interactions (PPIs), which are traditionally viewed as being notoriously difficult to modulate using small molecules.^14,40–42^

### Library synthesis: *build* stage

**Fig. 2:**
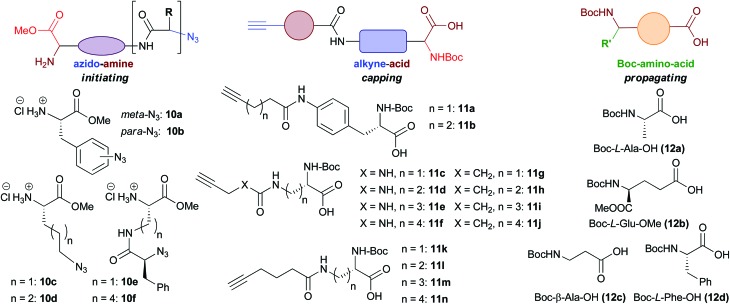
Building blocks used in library synthesis. Complete experimental procedures for the synthesis of the building blocks are given in the ESI.†

**Scheme 2:**
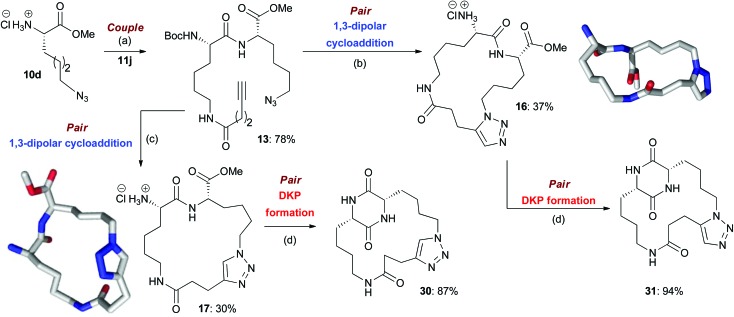
An illustrative example of the synthesis of final macrocyclic peptidomimetics using the B/C/P strategy. The lowest energy conformations (molecular shapes) of two final library compounds **16** and **17** are shown^46^ (conformational search by Molecular Operating Environment (MOE) software package^47^). Conditions: (a) EDC·HCl, HOBt, NEt_3_, CH_2_Cl_2_, rt; (b) (i) [Cp*RuCl]_4_, toluene, reflux; (ii) HCl–dioxane (4.0 M); (c) (i) CuI, DIPEA, THF, reflux; (ii) HCl–dioxane (4.0 M); (d) AcOH–NMM* (1.25 : 1, molar ratio), 2-Butanol, microwave irradiation (*T* = 150 °C). NMM*: morpholinomethyl-polystyrene (loading = 3.51 mmol g^–1^).

**Scheme 3:**
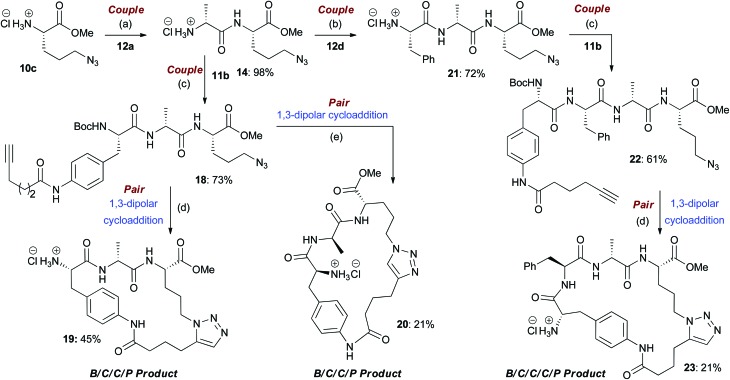
An illustrative example of the synthesis of some final macrocyclic peptidomimetics using a route incorporating iterative coupling steps (*i.e.* B/C/C/P and B/C/C/C/P). (a) (i) EDC·HCl, HOBt, Boc-l-Ala-OH (**12a**), NEt_3_, CH_2_Cl_2_, rt; (ii) TMSCl, MeOH, 0 °C to rt; (b) (i) EDC·HCl, HOBt, Boc-l-Phe-OH (**12d**), NEt_3_, CH_2_Cl_2_, rt; (ii) TMSCl, MeOH, 0 °C to rt; (c) EDC·HCl, HOBt, **11b**, NEt_3_, CH_2_Cl_2_, rt; (d) (i) [Cp*RuCl]_4_, toluene, reflux; (ii) HCl–dioxane (4.0 M); (e) (i) CuI, DIPEA, THF, reflux; (ii) HCl–dioxane (4.0 M).

**Scheme 4:**
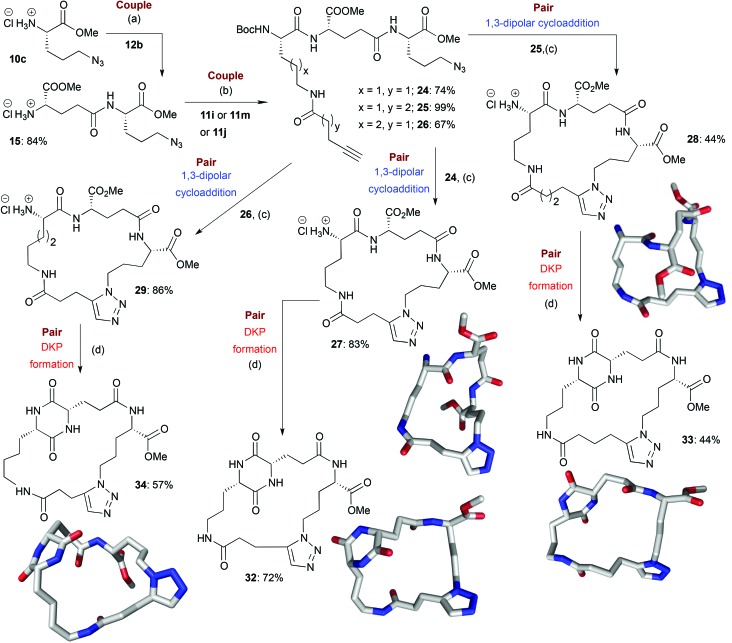
Illustrative example of the synthesis of some final macrocyclic peptidomimetics using a route incorporating two couple steps (*i.e.* B/C/C/P). The lowest energy conformations (molecular shapes) of some final library compounds (**27**, **28**, **32–34**) are shown^46^ (conformational search by MOE software package^47^). Conditions:(a) (i) EDC·HCl, HOBt, Boc-l-Glu-OMe (**12b**), NEt_3_, CH_2_Cl_2_, rt; (ii) TMSCl, MeOH, 0 °C to rt; (b) (i) EDC·HCl, HOBt, **11i** or **11m** or **11j**, NEt_3_, CH_2_Cl_2_, rt; (c) (i) [Cp*RuCl]_4_, toluene, reflux; (ii) HCl–dioxane (4.0 M); (d) AcOH–NMM* (1.25 : 1, molar ratio), 2-Butanol, Microwave irradiation (*T* = 150 °C). NMM*: morpholinomethyl-polystyrene (loading = 3.51 mmol g^–1^).

In total six azido-amines (**10a–f**), 14 alkyne-acids (**11a–n**) and four Boc-amino acids (**12a–d**) were readily prepared from simple, commercially available amino acid starting materials (Fig. 2). These building blocks were then utilised for library formation, as outlined in Scheme 1. The full synthetic sequences leading to a representative selection of final macrocyclic peptidomimetic compounds are discussed in more detail (Schemes 2–4). For a full list of the final compounds synthesised and synthetic details consult the ESI.†

### Library synthesis: couple stage

In the couple stage of the DOS, amide bond formation between various pairs of building-blocks (either “initiating” with “capping” or “initiating” with “propagating”), followed by optional Boc removal afforded a range of linear peptides, obtained as single stereoisomers after purification by column chromatography on silica.^43^ Some examples are shown in Schemes 2–4 (compounds **13–15**).

### Library synthesis: *pair* stage

#### B/C/P sequence

In the first *pair* step of the DOS, “click” type 1,3-dipolar cycloadditions with Cu or Ru catalysts were carried out on the broad range of linear substrates generated in the couple phase to furnish regioisomeric macrocycles containing 1,4- or 1,5-disubstituted triazoles respectively.^44^ A single stereoisomer was detected in the crude ^1^H NMR of each cyclized product, indicating that all the 1,3-dipolar cycloaddition reactions were highly regioselective and proceeded without epimerization of the chiral centres present. Purification by column chromatography on silica gel was generally straightforward and led to the isolation of a range of pure Boc-protected macrocycles, typically in good yields.^45^ Subsequent Boc group removal furnished the final macrocyclic peptidomimetic library members.^45^ The yields calculated over these two steps (macrocyclisation then deprotection) were generally good, with both Cu- and Ru-catalysis giving similar results (the average yield over the two-step sequence was 79% for B/C/P products whose synthesis involved a Cu-catalysed macrocyclisation and 78% for B/C/P products whose synthesis involved a Ru-catalysed macrocyclisation). In terms of the average purity of the final products, the Ru-catalysed process faired marginally better (87% average purity compared to 79% as determined by HPLC analysis).

As a representative example of this sequence, macrocycles **16** and **17** were both obtained from **13** with purities of 95% and 89% respectively (Scheme 2). These two macrocycles are each derived from the same two building blocks, however, they are each based on a different molecular scaffold and have very distinctive predicted molecular shapes (lowest energy conformations).^46^ This illustrates the utility of the DOS strategy to generate scaffold, and thus shape diversity, in a step-efficient fashion through variation in reaction conditions alone.

It is possible to identify some general underlying trends in the macrocyclisation reactions of the B/C/P sequence (if one assumes that the macrocyclisation reaction, rather then subsequent Boc group removal, is the main determinant of the yield and purity of the final product). Linear precursors that incorporated capping building block **11a** or **11b** and/or initiating building block **10a** or **10b** (which would be expected to furnish macrocycles that incorporated an aromatic ring into the macrocyclic ring architecture) were found to especially challenging substrates for the Cu-catalysed macrocyclisation process, with little/no conversion typically observed. It is possible that the presence of the aromatic ring in these substrates restricted their conformational flexibility such that any re-organization needed to bring their reactive termini in close spatial proximity would have been disfavoured (*i.e.* associated with a high degree of strain).^48^ Furthermore, there may be a high degree of strain, in the resulting macrocycles themselves due to the incorporation of the aromatic sp^2^ carbons into the macrocyclic ring architecture, which could have disfavoured their formation. Interestingly, the Ru-catalysed macrocyclisations of linear precursors that incorporated one of these building blocks typically proceeded more smoothly. It is possible that there is less strain associated with the incorporation of a 1,5-, rather than a 1,4-, disubstituted triazole in the resultant macrocycles. For B/C/P products whose synthesis involved a Cu-catalysed macrocyclisation, the yields obtained over the two-step macrocyclisation-deprotection sequence from substrates derived from initiating building block **10c** were often below average, and also typically below the values obtained for analogous substrates incorporating building block **10d**. It is possible that the differences in yields can be attributed to the shorter alkyl chain in **10c**; the macrocyclic ring systems that incorporate this unit would be smaller in size and perhaps more strained than those derived from **10d**, which may disfavour their formation. However, in the case of the corresponding Ru-catalysed macrocyclisation reactions, there was no noticeable difference in the yields obtained.

#### B/C/C/P and B/C/C/C/P sequences

The assembly and regioselective cyclization of alternative linear peptides obtained from different pairs of building blocks allowed for a combinatorial-type variation of macrocyclic scaffolds to be achieved. The cyclization of longer peptides generated by iterative coupling allowed step-economical access to a diverse range of larger peptidomimetic scaffolds. For example, the coupling of **10c** with **12a** furnished compound **14** (Scheme 3). A second coupling step with **11b** (a “capping” building block) provided compound **18**, which could be converted to macrocycles **19** and **20** by cycloaddition followed by Boc group removal; overall these represent the products of a B/C/C/P sequence (Scheme 3). Although the yields over the two steps were only moderate in both cases, the purities of the final compounds were good (purities of 88% and 72% respectively). Alternatively, coupling of **14** with **12d** provided compound **21**, which could then be combined with **11b** to furnish linear peptide **22**. Subsequent Ru-catalysed cycloaddition furnished **23**, formally the product of a B/C/C/C/P sequence, in a good purity (80%). In another example of the use of the B/C/C/P strategy, linear peptides **24**, **25** and **26** were converted to macrocycles **27**, **28** and **29** respectively (purities of 89%, 81% and 77% respectively) (Scheme 4).

For the B/C/C/P and B/C/C/C/P sequences, the average yields over the final two steps (macrocyclisation then deprotection) were generally lower than those seen for the B/C/P sequence (68% for all the B/C/C/P and B/C/C/C/P products whose synthesis involved a Cu-catalysed macrocyclisation and 55% for all the B/C/C/P and B/C/C/C/P products whose synthesis involved a Ru-catalysed macrocyclisation). This can potentially be attributed to unfavourable entropic factors associated with the formation of the larger-sized macrocyclic ring systems or perhaps the increased distance between reactive termini in the acyclic precursors, which may favour competing intermolecular reactions.^47^ Gratifyingly, the average purities of the B/C/C/P and B/C/C/C/P products (83% for products whose synthesis involved Cu-catalysis and 82% in the case of the use of Ru-catalysis) were comparable to those for products obtained *via* the B/C/P sequence.

#### DKP formation

In our previous scoping study, we reported the development of a unique method for introducing DKP units into macrocyclic frameworks based around the use of solid-supported *N*-methylmorpholine (NMM) (morpholinomethyl-polystyrene) in combination with microwave heating under slightly acidic buffered conditions. In general, the final products could be obtained with a good level of purity by simple filtration of the solid support and evaporation to dryness. Application of this protocol to the wider range of substrates employed in this work enabled efficient access to a diverse range of structurally complex DKP-containing macrocyclic peptidomimetics as final library members.^49^ Yields and purities were typically moderate-good (the average yield of all successful DKP formations was 57%, with an average purity of 77%). For example, **16** and **17** could be converted to **31** (94% yield, 87% purity) and **30** (87% yield, 90% purity) respectively (Scheme 2). DKP units could also be introduced into macrocyclic scaffolds generated from cyclization precursors incorporating more than two building blocks. For example, the B/C/C/P macrocycles **27–29** could be successfully converted to DKP-containing products **32–34** (Scheme 4). The fact that this single synthetic transformation yielded products with unique molecular scaffolds, and overall shapes,^46^ quite distinct from those of their corresponding macrocyclic precursors, provides a clear illustration of the utility of the overall DOS strategy in the context of step-efficient scaffold, and thus shape, diversity generation.

For macrocycles that incorporated an aromatic ring in the cyclic architecture, DKP formation was found to be challenging; reactions were typically sluggish, with poor conversion or proceeding in a low yield, presumably because the high rigidity of the macrocycle made additional ring closure difficult (as has previously been noted).^4^ The yields for DKP formations involving B/C/P macrocycles incorporating building block **10c** were generally found to be below average. This could possibly be attributed to the fact that such macrocycles have core ring systems that are relatively small in size (due to the relatively short alkyl chain in **10c**), which may have thus made further intramolecular cyclisation challenging due to high levels of associated strain.^48,50^

Overall, using the strategies outlined above, and a limited number of building blocks, the DOS of a library of 219 macrocyclic peptidomimetics was achieved. Each final compound contains a distinct molecular scaffold amongst other unique structural features. The library was made using parallel synthetic techniques, leading to at least 1 mg of each final product (typically 10 mg or above). All library members were assessed for their identity and quality (HPLC and LCMS). Full characterization of 46 (21%) of the final macrocyclic compounds was undertaken; HPLC and LCMS characterized the rest.

### Diversity assessment

**Fig. 3:**
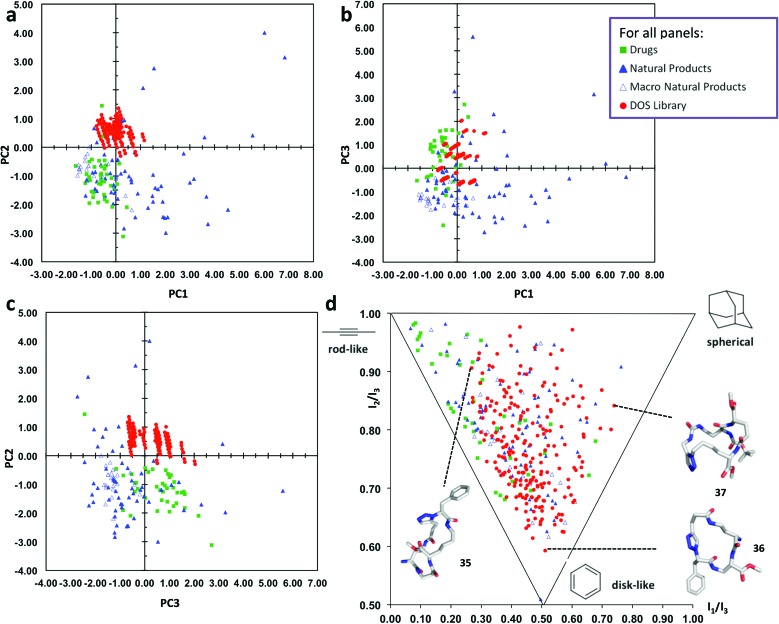
Comparative PCA and PMI plots of 219 macrocyclic DOS library compounds (“DOS library”, solid red circles), 40 top-selling brand-name drugs (“Drugs”, solid green squares), 60 diverse natural products (“Natural Products”, solid blue triangles) and 24 macrocyclic natural products (“Macro Natural Products”, empty blue triangles). (a) PCA plot of PC1 *versus* PC2. (b) PCA plot of PC1 *versus* PC3. (c) PCA plot of PC3 *versus* PC2. (d) PMI plot illustrating the molecular shape diversity of the DOS library. The lowest energy conformations (molecular shapes) of representative DOS library compounds **35–37** based on each of the three extremes of molecular shape types are shown^46^ (conformational search by MOE software package^47^). See ESI† for the structures of **35–37** and more details.

In order to assess the chemical space coverage of the DOS library, we compared it with three reference collections, comprising of 40 top-selling brand-name drugs, 60 diverse natural products and 24 macrocyclic natural products^51^ in a principal component analysis (PCA) based on various structural and physicochemical descriptors (see ESI, Table S1† for full details). The distribution of these four sets of compounds in chemical space is shown in Fig. 3a–c, which represent 87% of the information in the data set (see ESI, Table S2† for full details). The DOS macrocycle library clearly occupies a distinct region of chemical space compared to the drug and macrocyclic natural product libraries, most evident in Fig. 3a and c. PC2 is the principal component that mainly contributes to the separation of the DOS library from the reference drugs and macrocycles in chemical space, as substantial overlap with the drug library occurs in the plot of PC1 against PC3 (Fig. 3b). The most important contributors to the variance in PC2 are log *P*(o/w), *S* log *P* (an alternative way of obtaining log *P*) and log *S* (see ESI, Table S3†). It appears that an increase in hydrophilicity (decrease in log *P*, increase in log *S*) shifts molecules in the positive direction. This agrees with the fact that the DOS library comprises of macrocycles with a large number of polar amide bonds and therefore high hydrophilicity, thus differentiating them from the reference sets of drugs and macrocyclic natural products.

We also evaluated the molecular shape diversity of the same compound data sets by computing normalized ratios of principal moments of inertia (PMI) based on the lowest-energy conformations of the compounds.^46^ The PMI ratios were plotted on a triangular graph as previously described.^15^ This PMI plot (Fig. 3d) intuitively visualizes the shape diversity of each of the four collections in “molecular shape space” spanned by the three basic extreme shape types “rod-like”, “disk-like” and “spherical”. The drug reference set predominantly contains compounds with rod-like shapes with some disc-like features, whereas the natural products display much larger shape diversity. Notably, our DOS compounds exhibit almost the same range of shape diversity as the natural products (albeit lacking in some rod-like character), overlapping to a substantial extent with the drugs and macrocyclic natural products in the PMI plot; thus the library can be said to have a very high level of shape diversity. The structures of three macrocycle library compounds are shown in Fig. 3 to illustrate the diversity in molecular shapes that are obtained by our DOS approach.^46^

## Conclusions

Herein, we have described the development of a new advanced DOS strategy that enables the combinatorial-type variation of macrocyclic peptidomimetic scaffolds. Our synthetic approach is based around a B/C/P algorithm that incorporates iterative couple and pair stages. Application of this new DOS strategy enabled concise, flexible and systematic access to a structurally diverse library of over 200 unique macrocyclic peptidomimetics, each based around a distinct molecular scaffold, from simple, readily available amino acid starting materials. To the best of our knowledge this represents an unprecedented level of scaffold diversity in a synthetically-derived library of macrocyclic peptidomimetic compounds. Each library compound is structurally complex (an important feature for the specificity of biological interactions^52–54^) and rich in functionality, containing numerous potential bimolecular-interacting elements and structural motifs related to those found in numerous biologically active compounds. For example, 27% of macrocyclic library members incorporate a DKP motif, a privileged structural motif in terms of bio-relevance. The remaining library members incorporate a free amine and an ester group, which provide synthetic handles for further derivatisation. This may offer a means of tuning the biological profile of a compound or enable the labelling of compounds for application as chemical probes.^4^ PCA indicates that the compound library accesses regions of chemical space that overlap with some natural products but which are distinct from those addressed by top-selling brand-name drugs and macrocyclic natural products. This illustrates the ability of our DOS approach to sample attractive regions of chemical space underexploited in current drug-discovery efforts. In addition, PMI analysis shows that the DOS library has a relatively high level of shape diversity, thus demonstrating the utility of the DOS strategy for the preparation of shape-diverse libraries.^46^ Another notable feature of the DOS is that all library compounds were generated on milligram (typically multi-milligram) scale. This enabled the identity of all compounds to be unambiguously determined. In addition, the quantities of each compound are sufficient for screening in a large number of biological assays. The DOS library compounds are now being screened against a wide range of biological targets, including challenging ones such as PPIs and multi-drug resistant bacterial strains. The results from these studies, which will be reported in due course, will provide much-needed further insight into the functional capabilities of this class of molecules. The inherent modularity of the DOS should facilitate the systematic modification and optimisation of any library compounds that are found to display interesting biological properties.

In this report, we have demonstrated the generality and robustness of our DOS strategy for the step-efficient synthesis of hundreds of different macrocyclic peptidomimetic scaffolds. In principle, the DOS strategy could be applied on a larger scale, using a greater number of each of the three types of building blocks, in order to access an even greater number of macrocyclic scaffolds. Currently, the most significant bottleneck in the DOS is the need for purification by column chromatography.^43^ It may be possible to adapt the DOS methodology for use on solid support, which would make the strategy more high-throughput and thus more amenable to the preparation of even larger numbers of compounds.

As previously highlighted, the nature of the appendages around macrocyclic scaffolds can have a profound effect upon their biological profiles. The main focus of the study described herein was to develop a general and step-efficient DOS algorithm towards varied macrocyclic peptidomimetic scaffolds; appendage variation was not examined in detail. In principle, there are two general approaches by which appendage diversity could be introduced into macrocyclic libraries generated using this DOS strategy. The first would involve post-pairing elaborations of the macrocyclic scaffolds with different groups through reactions of existing functional handles. For example, with regards to the non-DKP macrocycles described in this report, one could envisage using the free amine group as a site for further elaboration *via* amide bond formation; where a DKP unit is present, it may prove possible to introduce additional chemical functionality around the macrocycle *via*, for example, N-alkylation.^55^ The second approach to the variation of macrocyclic appendages would exploit the inherent modularity of the DOS and involve the use of a range of building blocks that already containing the appendage(s) of interest, or protected forms thereof (*e.g.* variation in the R and R′ groups in the “initiating” and “propagating” building blocks shown in Fig. 2). Only a restricted range of building-block substituents was explored in this report. However, given that all three types of building blocks in the DOS are derived from amino acid starting materials, it should be possible to readily achieve greater levels of diversity in macrocyclic appendages through the use of the wide variety of natural and unnatural α, β and γ amino acid derivatives that are commercially available or readily prepared, in the build phase of the synthesis.^4^
