# Supplementary material for: A diversity-oriented synthesis strategy enabling the combinatorial-type variation of macrocyclic peptidomimetic scaffolds
Source: Org Biomol Chem. 2015 Mar 17;13(15):4570–80. doi: 10.1039/c5ob00371g (PMC4441267; doi:10.1039/c5ob00371g)
Supplement: Supplementary file 1 [file OB-013-C5OB00371G-s001.pdf]

Electronic Supplementary Material (ESI) for Organic & Biomolecular Chemistry.
This journal is © The Royal Society of Chemistry 2015

Supplementary Information

A Diversity-Oriented Synthesis Strategy Enabling the Combinatorial-Type Variation of Macrocyclic Peptidomimetic Scaffolds

Albert Isidro-Llobet, Kathy Hadje Georgiou, Warren R. J. D. Galloway, Elisa Giacomini, Mette Hansen, Gabriela Mendez, Yaw Sing Tan, Laura Carro, Hannah F. Sore, David R. Spring*

Department of Chemistry, University of Cambridge, Lensfield Road, Cambridge, CB2 1EW, United Kingdom

*To whom correspondence may be addressed: Department of Chemistry, University of Cambridge, Lensfield Road, Cambridge, CB2 1EW (UK), Fax: (+44) 1223-336362. E-mail: spring@ch.cam.ac.uk. Homepage: http://www-spring.ch.cam.ac.uk/

Table of Contents

1. COMPOUND LABELLING IN MAUSCRIPT AND SUPPLEMENTARY INFORMATION....3
2. SUPPLEMENTARY FIGURES 10
3. GENERAL METHODS AND EQUIPMENT........ 14
4. GENERAL PROCEDURES........ 16
5. SYNTHESIS OF THE COMMON PRECURSORS........ 18
6. SYNTHESIS OF AZIDO-AMINE BUILDING BLOCKS........ 20
7. SYNTHESIS OF ALKYNE-ACID BUILDING BLOCKS 29
8. SYNTHESIS OF B/C/C/P AND B/C/C/C/P COUPLING UNITS 36
9. PREPARATION OF B/C/P LINEAR AMIDES 38
10. PREPARATION OF B/C/C/P AND B/C/C/C/P LINEAR AMIDES 54
11. PREPARATION OF B/C/P CUAAC MACROCYCLES........ 61
12. PREPARATION OF B/C/C/P AND B/C/C/C/P CUAAC MACROCYCLES........ 71
13. PREPARATION OF B/C/P RUAAC MACROCYCLES 74
14. PREPARATION OF B/C/C/P AND B/C/C/C/P RUAAC MACROCYCLES........ 89
15. PREPARATION OF B/C/P DKPS........ 96
16. PREPARATION OF B/C/C/P AND B/C/C/C/P DKPS........108
17. CHEMOINFORMATIC ANALYSIS........109
18. REFERENCES........140
19. NMR SPECTRA........141

1. Compound labelling in mauscript and Supplementary Information

For the sake of clarity, the compound labeling systems used here in the Supplementary Information is different to that used in the main manuscript. The following table lists the compounds given in the main manuscript that are labeled, and their corresponding labels as used in the Supplementary Information.

Figure/Scheme in manuscript	Compound	Compound label in manuscript	Compound label in Supporting Informtion
**Figure 2**	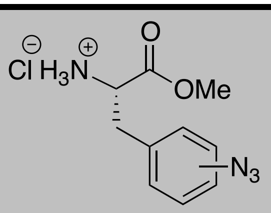	**10a** (*meta*)<br>**10b** *(para)*	Building block F<br>Building block C
**Figure 2**	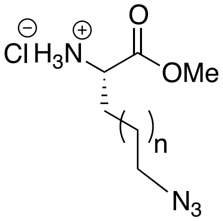	n = 1: **10c**<br>n = 2: **10d**	Building block E<br>Building block D
**Figure 2**	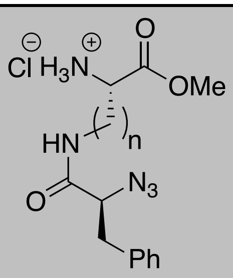	n = 1: **10e**<br>n = 4: **10f**	Building block B<br>Building block A
**Figure 2**	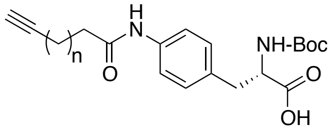	n = 1: **11a**<br>n = 2: **11b**	Building block 6<br>Building block 13
**Figure 2**	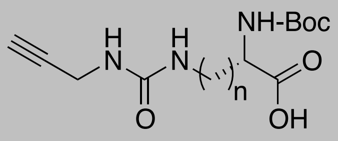	n = 1: **11c**<br>n = 2: **11d**<br>n = 3: **11e**<br>n = 4: **11f**	Building block 12<br>Buildong block 11<br>Building Block 5<br>Building block 4

**Figure 2**	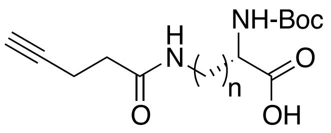	n = 1: **11g**<br>n = 2: **11h**<br>n = 3: **11i**<br>n = 4: **11j**	Building blok 10<br>Building Block 8<br>Building block 2<br>Building block 14
**Figure 2**	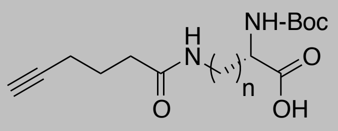	n = 1: **11k**<br>n = 2: **11l**<br>n = 3: **11m**<br>n = 4: **11n**	Building block 9<br>Building block 7<br>Building block 3<br>Building block 1
**Figure 2**	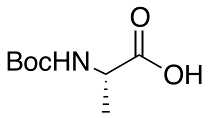	Boc-*L*-Ala-OH **(12a)**	Ala
**Figure 2**	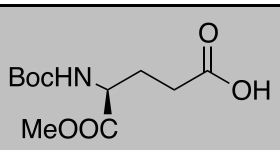	Boc-*L*-Glu-OMe **(12b)**	Glu
**Figure 2**	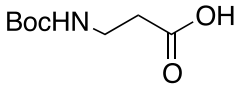	Boc-*β*-Ala-OH **(12c)**	-*β*-Ala
**Figure 2**	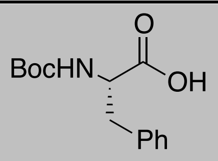	Boc-*L*-Phe-OH **(12d)**	Phe
**Scheme 2**	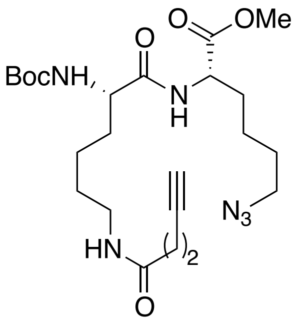	**13**	D14

**Scheme 2**	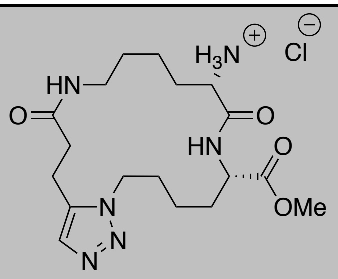	**16**	D14x
**Scheme 2**	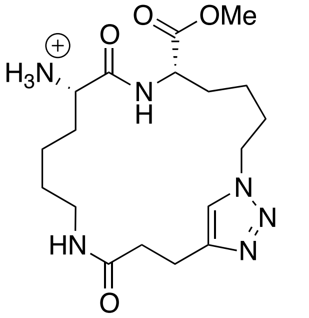	**17**	D14w
**Scheme 2**	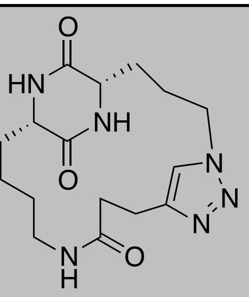	**30**	D14y
**Scheme 2**	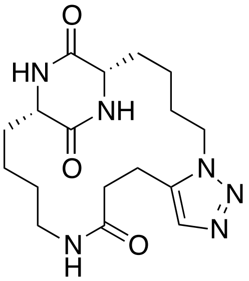	**31**	D14z
**Scheme 3**	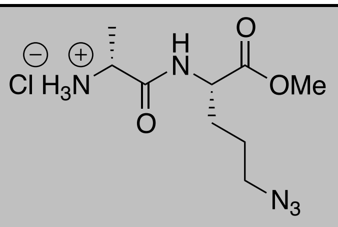	**14**	J
**Scheme 3**	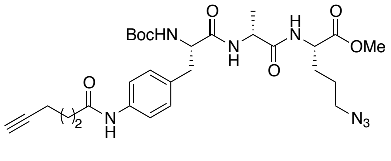	**18**	J13

**Scheme 3**	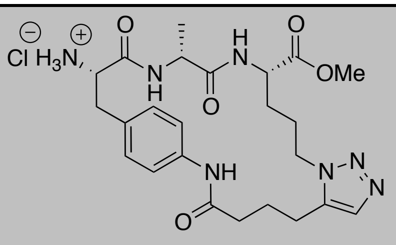	**19**	J13x
**Scheme 3**	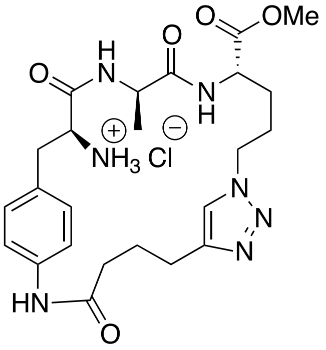	**20**	J13w
**Scheme 3**	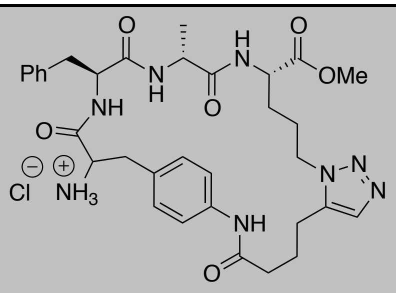	**23**	M13x
**Scheme 3**	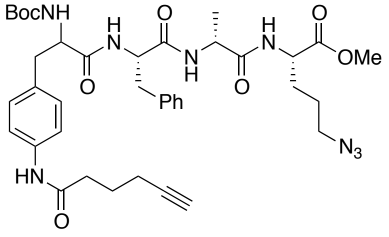	**22**	M13
**Scheme 3**	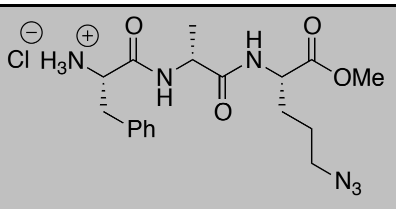	**21**	M
**Scheme 4**	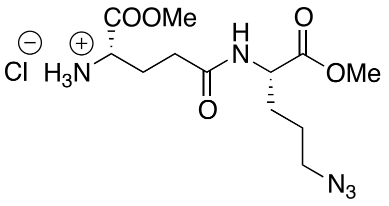	**15**	H

**Scheme 4**	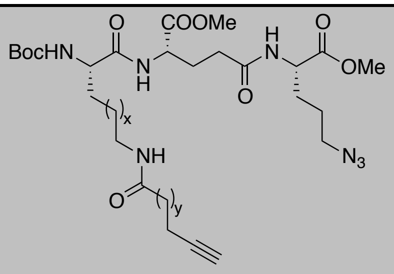	x = 1, y =1: **24**<br>x = 1, y =2: **25**<br>x = 2, y =1: **26**	H2<br>H3<br>H14
**Scheme 4**	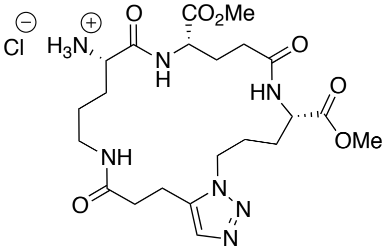	**27**	H2x
**Scheme 4**	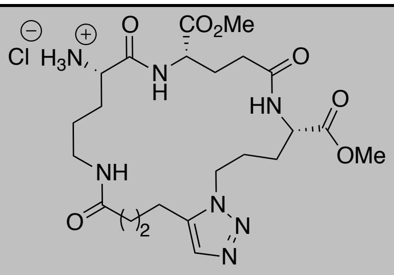	**28**	H3x
**Scheme 4**	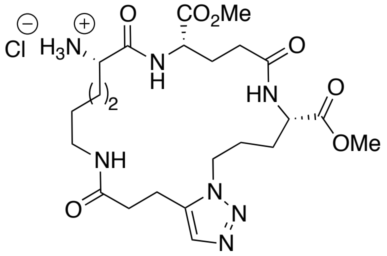	**29**	H14x
**Scheme 4**	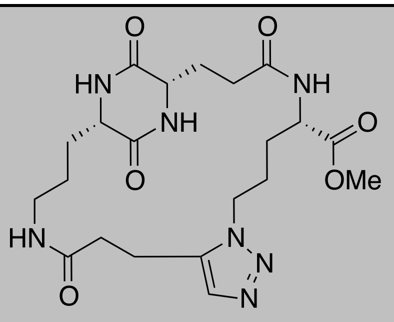	**32**	H2z

**Scheme 4**	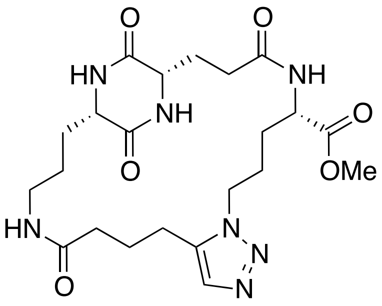	**33**	H3z
**Scheme 4**	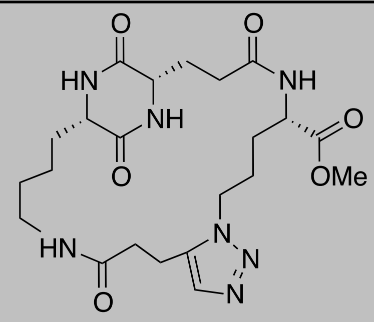	**34**	H14z
**Figure 3**	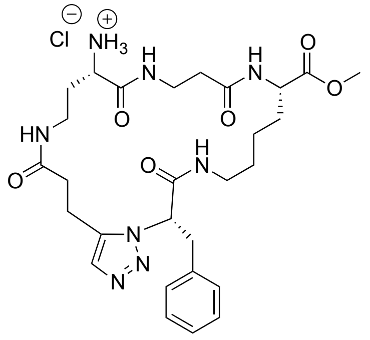	**35**	K8x
**Figure 3**	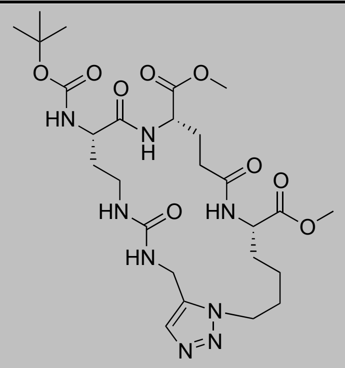	**36**	G11x

**Figure 3**	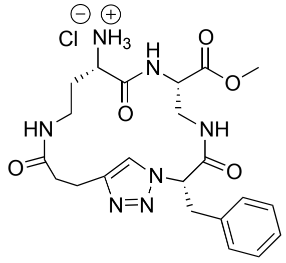	**37**	B8w

2. Supplementary Figures

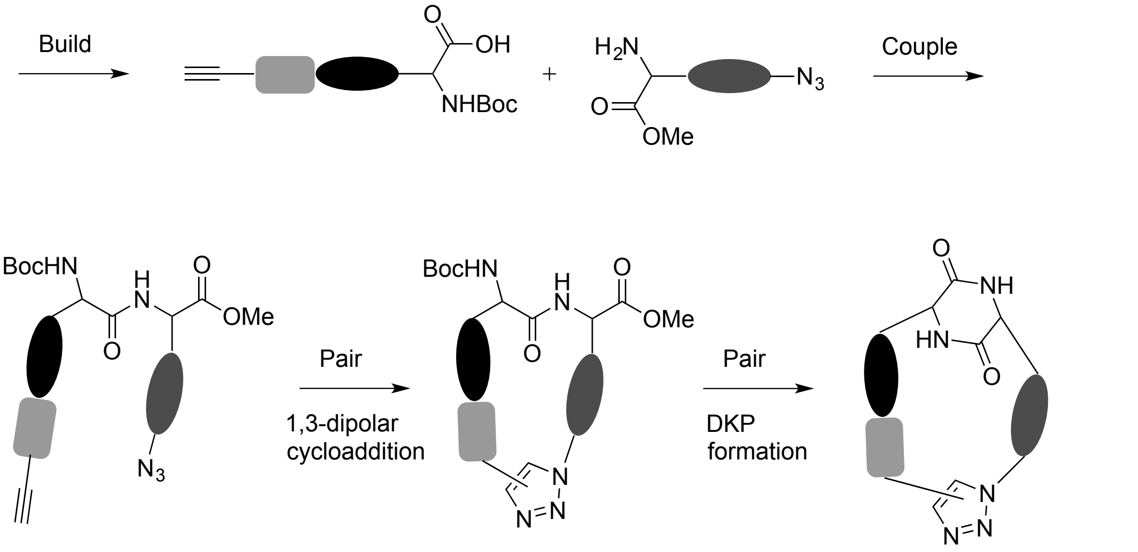

SF1: General scheme for the synthesis of diverse peptidomimetic macrocyclic scaffolds

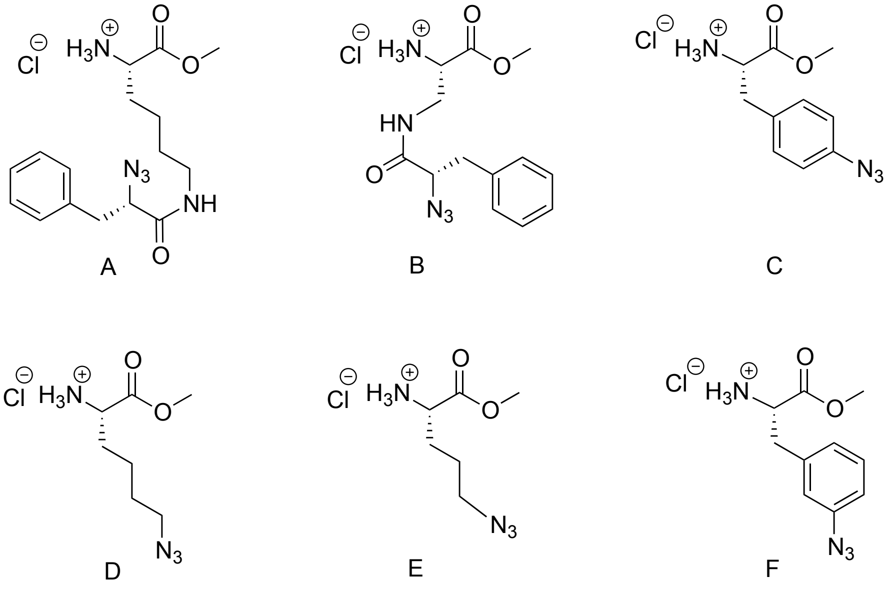

SF2: Azido-amine ("initiating") building blocks

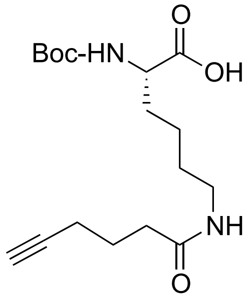

1

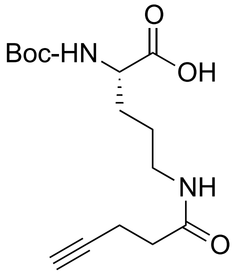

2

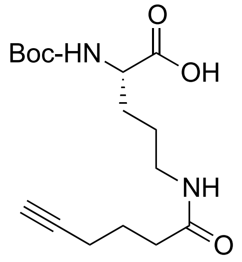

3

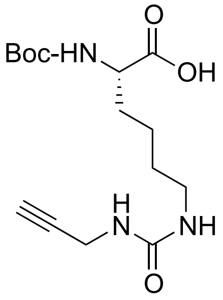

4

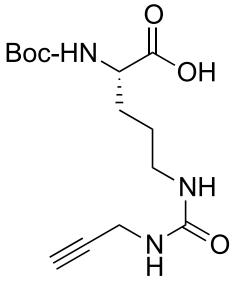

5

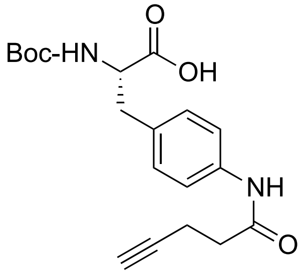

6

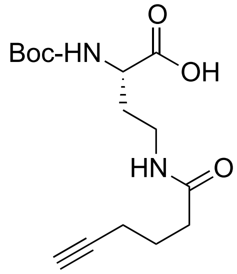

7

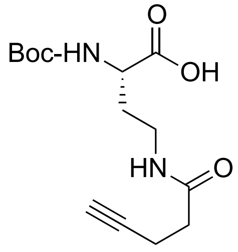

8

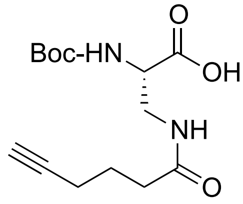

9

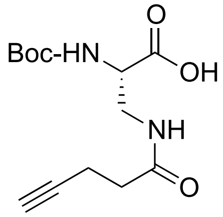

10

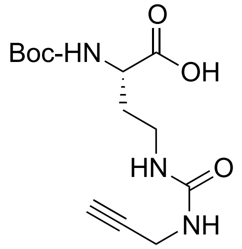

11

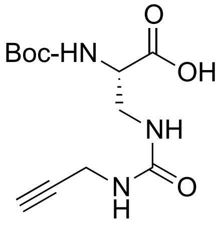

12

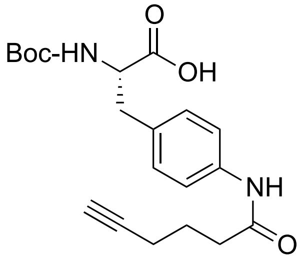

13

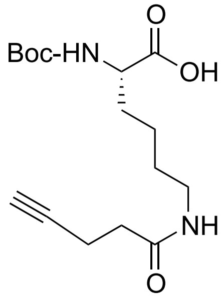

14

SF3: Alkyne-acid ("capping") building blocks

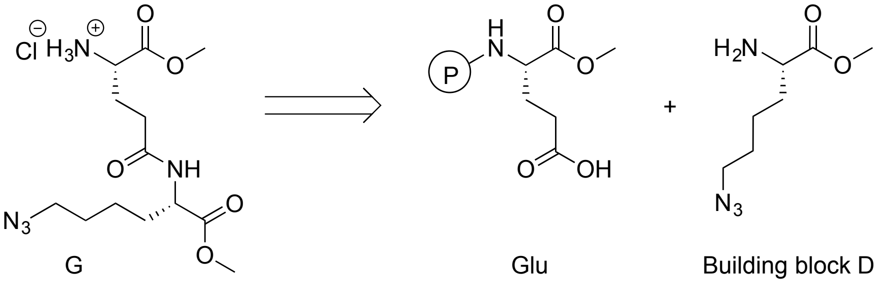

G — Glu — Building block D

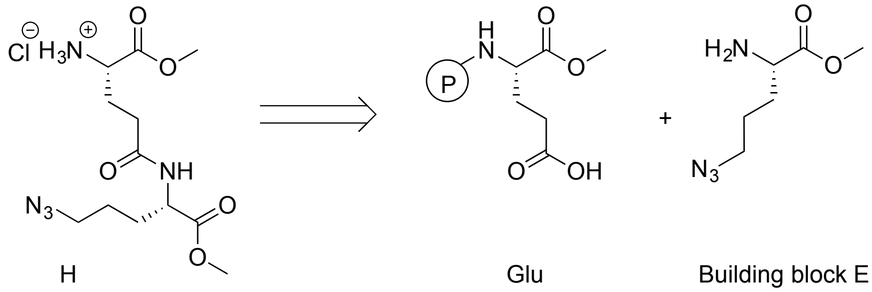

H — Glu — Building block E

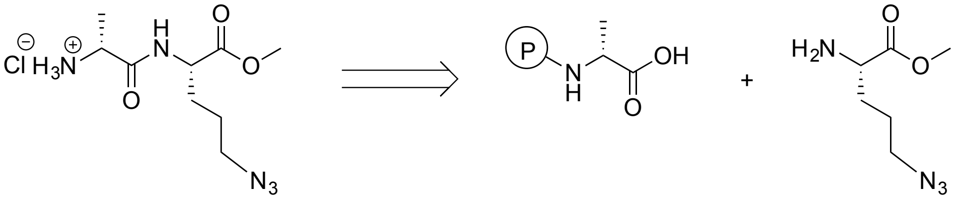

J — ala — Building block E

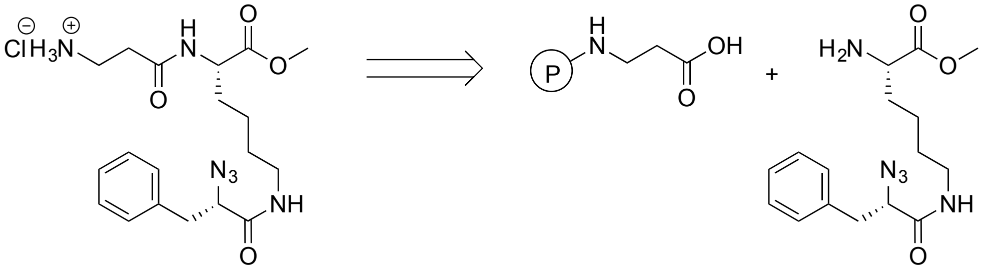

K — β-ala — Building block A

SF4: Synthetic Route to B/C/C/P Coupling Units from Boc-amino-acids ("propagating" building blocks)

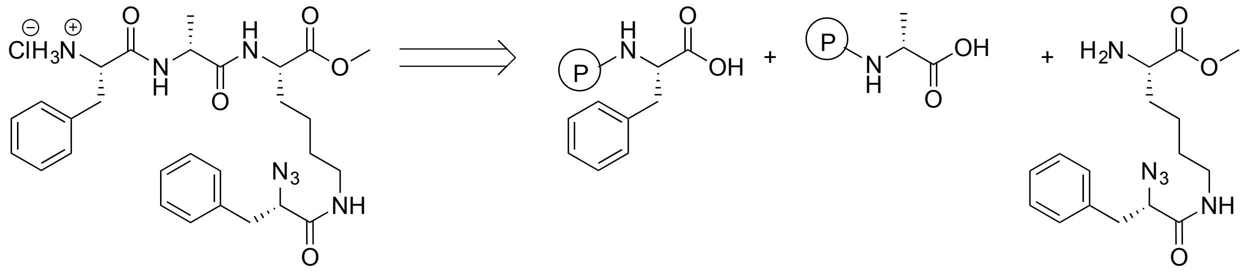

L phe ala Building block A

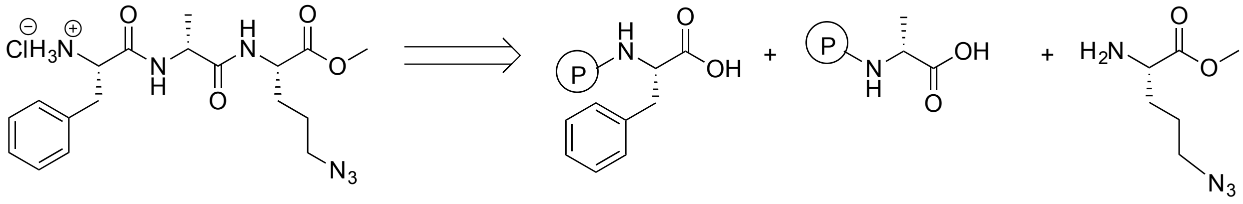

M phe ala Building block E

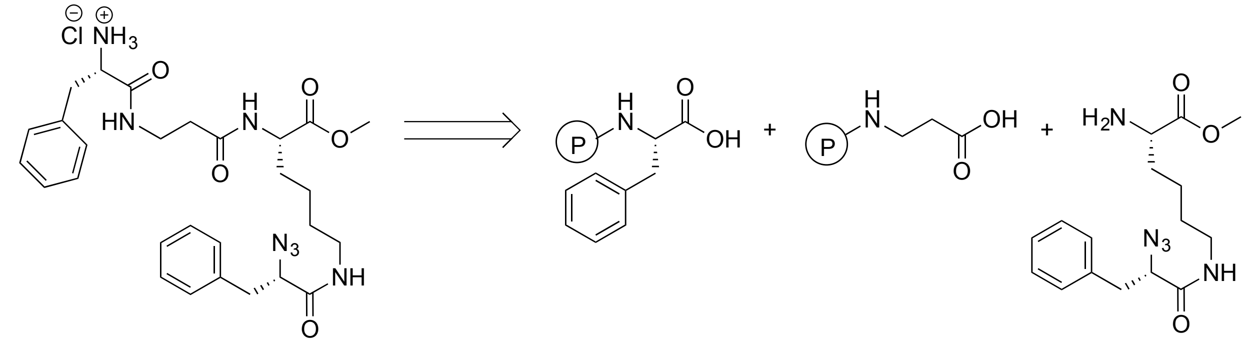

N phe β-ala Building block A

SF5: Synthetic Route to B/C/C/C/P Coupling Units from Boc-amino-acids ("propagating" building blocks)

3. General Methods and Equipment

Except as otherwise indicated, reactions were carried out using oven-dried glassware under nitrogen with dry, freshly distilled solvents. Tetrahydrofuran was distilled from calcium hydride and $LiAlH_4$ in the presence of triphenyl methane. Diethyl ether was distilled from calcium hydride and $LiAlH_4$. CH_2Cl_2, MeOH, toluene, MeCN and hexane were distilled from calcium hydride. Petroleum ether refers to the 40-60 °C fractions. All other reagents were used as obtained from commercial sources.

Room temperature (rt) refers to ambient temperature. Temperatures at 0 °C were maintained using an ice-water bath. Reactions involving microwave irradiation were performed using a CEM Discover® microwave apparatus in 10 ml or 30 ml microwave tubes with clip lids.

Where possible, reactions were monitored by thin layer chromatography (TLC) using glass plates precoated with Merck silica gel 60 F_{254}. Visualization was by the quenching of UV fluorescence (λ_{max} = 254 nm) or by staining with potassium permanganate. Retention factors (R_f) are quoted to 0.01.

Flash column chromatography was carried out using slurry-packed Merck 9385 Kieselgel 60 silica gel under a positive pressure of air or nitrogen.

Preparative HPLC purification was performed on an Agilent 1260 Infinity system fitted with a Supelcosil ABZ+Plus column (250 mm x 21.2 mm, 5 µm) using linear gradient systems (solvent A: 0.1% (v/v) TFA in water, solvent B: 0.05% (v/v) TFA in acetonitrile) at a flow rate of 20 mL min^{-1}.

Analytical HPLC analysis was performed on an Agilent 1260 Infinity system fitted with a Supelcosil ABZ+Plus column (150 mm x 4.6 mm, 3 µm) using linear gradient systems (solvent A: 0.05% (v/v) TFA in water, solvent B: 0.05% (v/v) TFA in acetonitrile) over 15 min at a flow rate of 1 mL min^{-1} and UV detection (λ_{max} = 220 nm and 254 nm). Retention times (t_r) are reported to the nearest 0.01 min. Peak area percentages are calculated for the UV absorbance at 220 nm and reported to the nearest 1%.

Liquid chromatography mass spectrometry (LC-MS) was conducted on an Agilent 1100 series LC with an ESCi Multi-Mode Ionisation Waters ZQ spectrometer. LC system: solvent A: 10 mM NH_4OAc + 0.1% HCOOH in water; solvent B: 95% acetonitrile + 5% H_2O + 0.05% HCOOH; column: Supelcosil™ ABZ^+PLUS column (33 mm × 4.6 mm, 3 µm); gradient: 0.0-0.7 min: 0% B, 0.7-4.2 min: 0-100% B, 4.2-7.7 min: 100% B, 7.7-8.5 min: 100-0% B; DAD spectrum: 190 nm - 600 nm, interval 2.0 nm, peak width 0.200 min). Only molecular ions are reported. ESI refers to the electrospray ionisation technique.

Melting points were obtained using a Büchi Melting Point B-545 melting point apparatus and are uncorrected.

Optical rotations were recorded on a Perkin Elmer 343 polarimeter. $[\alpha]_D$ values are reported in 10^{-1} deg cm^2 g^{-1} at 589 nm, concentration (c) is given in g $(100\ mL)^{-1}$.

Infrared (IR) spectra were recorded on a Perkin-Elmer Spectrum One FT-IR spectrometer with internal referencing as neat films. Selected absorption maxima (ν_{max}) are reported in wavenumbers (cm^{-1}).

Nuclear magnetic resonance (NMR) spectra were recorded using an internal deuterium lock on Bruker DPX 400 (400MHz), Bruker Avance 400 QNP Ultrashield (400 MHz), Bruker Avance 500 BB ATM (500 MHz) and Bruker Avance 500 Cryo Ultrashield (500 MHz) spectrometers. Chemical shifts (δ) are referenced to the solvent signal and are quoted in ppm to the nearest 0.01 ppm for δ_H and to the nearest 0.1 ppm for δ_C. Coupling constants (*J*) are reported in Hertz to the nearest 0.1 Hz. Assignments are supported by DEPT-135, ^1H-^1H COSY, HMQC, HMBC and NOESY spectra where necessary. Data are reported as follows: chemical shift, integration, multiplicity (app., apparent; br, broad; s, singlet; d, doublet; t, triplet; q, quartet; quint, quintet; m, multiplet; or as a combination of these), coupling constant(s) and assignment (corresponding atom in itallics). Diastereotopic protons are assigned as H and $\underline{H}$, where H indicates the proton at higher chemical shift. The numbering schemes used on selected spectra do not follow the IUPAC naming system and are used for the clear assignment of ^1H and ^{13}C spectra.

Low resolution mass spectra (ESI) were recorded using an LCMS system (Agilent 1200 series LC with an ESCi Multi-Mode Ionization Waters ZQ spectrometer using MassLynx 4.1 software).

High resolution mass spectrometry (HRMS) was carried out with a Micromass QTOF or a Waters LCT Premier Mass Spectrometer using electospray ionisation [ESI] or electron ionisation [EI]. The calculated mass value relative to found mass value is within the error limits of ±5 ppm mass units.

4. General Procedures

GP1: Amide formation

The azido-amine (1.0 equiv) was dissolved in anhydrous CH_2Cl_2 and triethylamine (2.2 equiv), EDC.HCl (1.1 equiv) and HOBt.H_2O (1.1 equiv) were then added. Upon dissolution, the alkyne-acid (1.0 equiv) in anhydrous CH_2Cl_2 was added and the reaction was stirred at rt for 18 h. The solvent was removed under reduced pressure and the residue was re-suspended in EtOAc and washed with H_2O. The organic layer was separated and washed with saturated $NaHCO_3$ solution (×2). A second addition of EtOAc was made and the organic fraction was washed with 5% citric acid (×2) and H_2O (×2). The organic phase was dried ($MgSO_4$) and the solvent removed under reduced pressure. The crude material was purified by column chromatography to yield the linear peptide.

GP2: CuAAC Macrocyclization to form 1,4-triazoles

DIPEA (3.0 equiv) was added to a solution of the linear peptide (1.0 equiv, 1.2 mM) in anhydrous THF. The reaction was degassed by bubbling Ar directly into the solution for 30 min. CuI (2.0 equiv) was then added and the reaction was refluxed for 18 h under N_2. The solvent was removed under reduced pressure and the crude material purified by column chromatography or preparative HPLC if necessary.

GP3: RuAAC Macrocyclization to form 1, 5-triazoles

The linear peptide (1.0 equiv., 1.25 mM) was dissolved in anhydrous toluene and the reaction mixture was heated to 80 °C and then degassed by bubbling Ar directly into the solution for 30 min. $[Cp^*RuCl]_4$ (0.1 equiv.) was added and the reaction was heated to reflux for 18 h. The solvent was removed under reduced pressure and the crude material purified by column chromatography or preparative HPLC if necessary.

GP4: Removal of Boc protecting group with TMSCl

The Boc-protected macrocycle was dissolved in MeOH and the suspension was cooled to 0 °C. TMSCl (0.3 ml per 0.035 mmol linear peptide) was added dropwise to the solution. at 0 °C with stirring. The reaction was allowed to warm to rt and stirred until TLC analysis indicated complete consumption of starting material (typically 3 h). The solvent was removed under reduced pressure and the crude material resuspended in CH_2Cl_2 and washed with. $NaHCO_3$. The aqueous layer was extracted with CH_2Cl_2 (×2) and the combined organic fractions were dried ($MgSO_4$) and the solvent removed under reduced pressure. The crude material was purified by column chromatography (or preparative HPLC) if required to yield the macrocyclic peptidomimetic.

GP5: Removal of Boc protecting group with HCl

The Boc-protected macrocycle was treated with 4.0 M HCl/dioxane (1 ml HCl/dioxane per 20 mg sample) and the reaction was stirred at rt for 18 h. The solvent was removed under reduced pressure and the crude material resuspended in CH_2Cl_2 and washed with $NaHCO_3$. The aqueous layer was extracted with CH_2Cl_2 (×2) and the combined organic fractions were dried ($MgSO_4$) and the solvent removed under reduced pressure. The crude material was purified by column chromatography (or preparative HPLC) if required to yield the macrocyclic peptidomimetic.

GP6: Synthesis of diketopiperazine

The macrocycle (0.3 equiv, used directly after the deprotection and used as the salt, without purification) and morpholinomethyl-polystyrene (1.0 equiv) were placed in a microwave (MW) tube. 2-Butanol (40 ml per mmol) and acetic acid (1.25 equiv) were added and the reaction was heated to 150 °C in a microwave (typically 2-3 h). The resin was filtered off and several washings with MeOH and CH_2Cl_2 were performed. The filtrate was evaporated to dryness and the crude material was purified by column chromatography to yield the final DKP-containing macrocycle.

5. Synthesis of the Common Precursors

(*S*)-3-amino-2-((*tert*-butoxycarbonyl)amino)propanoic acid (CP1)

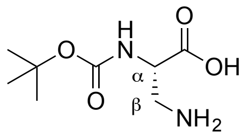

Boc-Asn-OH (8.00 g, 34.4 mmol) was suspended in EtOAc (40 ml), CH_3CN (40 ml) and H_2O (20 ml) and the mixture was cooled to 15 °C. PIDA (13.3 g, 41.3 mmol) was added in a single portion and following 45 min of stirring at 15 °C, the reaction was allowed to warm to rt. TLC analysis after 4 h indicated that most of the starting material was consumed. The reaction mixture was heated to 70 °C for 5 min (until completely dissolved) and then cooled to 0 °C. The mixture was filtered and the precipitate was washed on the filter with cold EtOAc (2 × 10 ml) to afford the title compound **41** as an amorphous white solid (4.72 g, 67%).

R_f = 0.07 (15% MeOH/ 85% CH_2Cl_2). **Mp** = 214-216 °C (EtOAc), lit. mp 216 °C (EtOAc).[1] **δ_H /ppm** (500 MHz, CD_3OD): 4.06 (1H, t, *J*=6.1 Hz, H_α), 3.22-3.09 (2H, m, H_β), 1.46 (9H, s, $C(CH_3)_3$). **δ_C /ppm** (125 MHz, CD_3OD): 174.8 (*C*OOH), 158.0 (O*C*=ONH), 80.9 ($C(CH_3)_3$), 53.9 (C_α), 43.1 (C_β), 28.7 ($C(CH_3)_3$). **v_{max} /cm^{-1}:** 3342 (m, N-H), 2969 (w, C-H), 2929 (w, O-H), 2580 (m, O-H), 1684 (C=O), 1525 (s, N-H). **HRMS** (ESI+) *m/z* found $[M+H]^+$ 205.1178, $C_8H_{17}N_2O_4^+$ required 205.1183 (Δ -2.3 ppm). **$[\alpha]_D^{25}$** = +19.0 (*c* 0.30, MeOH). Spectroscopic data is consistent with literature values.[2]

4-nitrophenyl prop-2-yn-1-ylcarbamate (CP2)

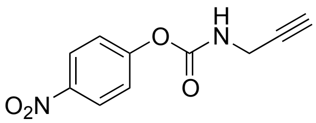

p-Nitrophenylchloroformate (2.98 g, 14.8 mmol) was dissolved in THF (80 mL) and cooled to -55°C in a acetone/acetonitrile/dry ice bath. After 10 minutes, propargylamine was added dropwise over 15 minutes. The resulting mixture was stirred at -55 °C for 45 minutes. The mixture was filtered through a plug of silica gel over Celite. The solids were washed with THF (3x40 mL). The combined eluents were concentrated *in vacuo*. The resulting yellow solid was recrystallised from EtOAc:hexane (1:2). Light brown crystals were isolated by filtration, washed with hexane and dried (1.648 g, 51%).

Spectroscopic data is consistent with literature values.[3]

4-pentynoic acid succinimidyl ester (CP3)

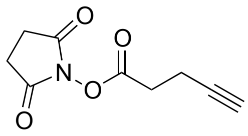

CP3 was prepared by literature procedures.[4]

5-hexynoic acid succinimidyl ester (CP4)

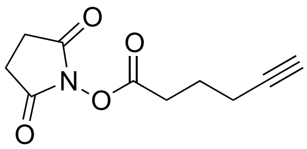

CP4 was prepared by literature procedures.[5]

6. Synthesis of Azido-Amine Building Blocks

Building Block A

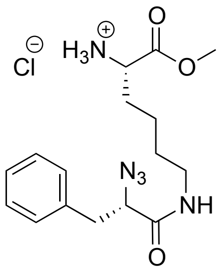

Building block A was prepared by literature procedures.[6]

Building Block B

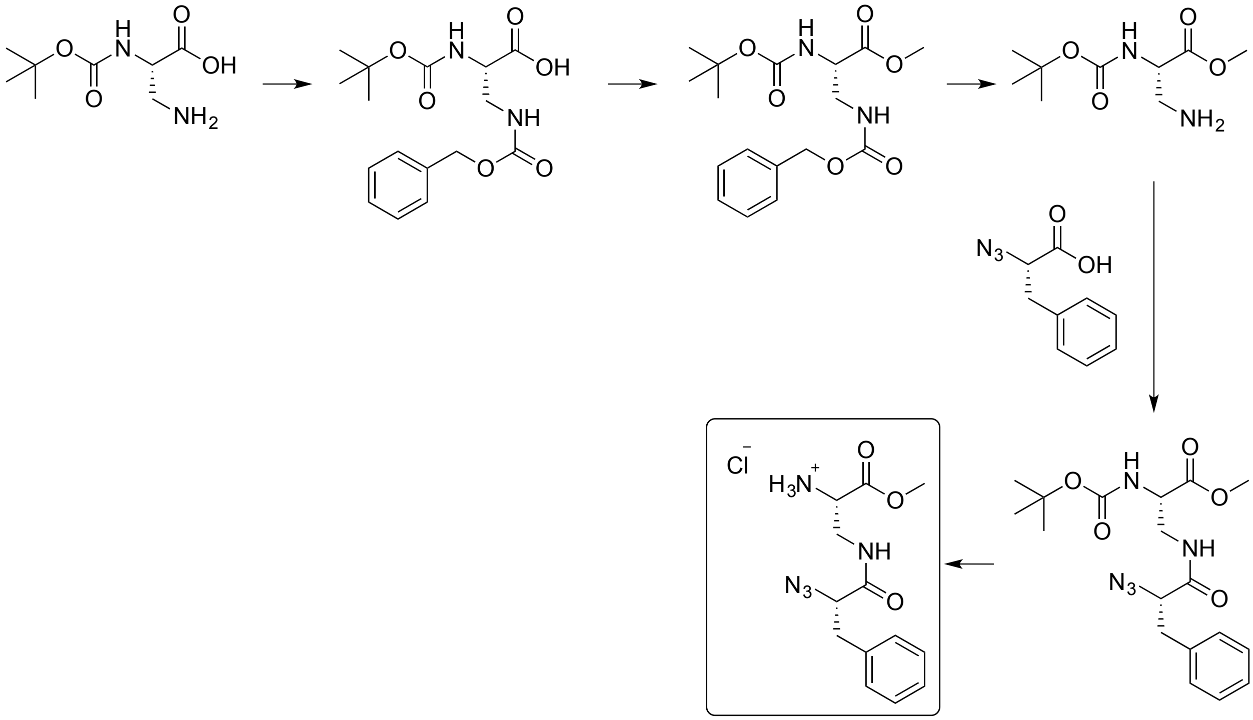

SF6: Synthetic Route to Building Block B

(*S*)-3-(((benzyloxy)carbonyl)amino)-2-((*tert*-butoxycarbonyl)amino) propanoic acid (abbreviated to Boc-Dap(Cbz)-OH)

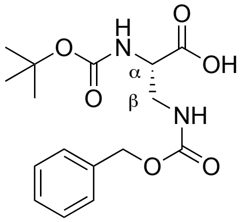

A mixture of Boc-dap-OH **CP1** (2.50 g, 12.2 mmol), K_2CO_3 (3.40 g, 24.6 mmol), KOH (0.695 g, 12.4 mmol), THF (22 ml) and H_2O (7 ml) was cooled to 0 °C. Benzyl chloroformate (2.60 ml, 18.2 mmol) was added dropwise over an hour and the reaction was allowed to warm to rt and stirred for 18 h. The organic solvents were removed under reduced pressure and the residue was diluted with H_2O (110 ml). The aqueous phase was acidified with citric acid to a pH of 4 and extracted with CH_2Cl_2 (2 × 15 ml). The combined organic fractions were dried ($MgSO_4$) and the solvent removed under reduced pressure. The crude material was purified by column chromatography (1% AcOH/ 10% MeOH/ 89% CH_2Cl_2) to afford the title compound **3** as a white solid (2.24 g, 54%).

R_f = 0.45 (1% AcOH/ 10% MeOH/ 89% CH_2Cl_2). **Mp** = 47-49 °C (CH_2Cl_2). **δ_H /ppm** (500 MHz, $CDCl_3$): 8.94 (1H, br s, COO*H*), 7.37-7.27 (5H, m, 5 × ArC*H*), 5.77 (1H, br s, C_α-N*H*), 5.55 (1H, br s, C_β-N*H*), 5.07 (2H, s, C*H*$_2$Ph), 4.45-4.20 (1H, m, H_α), 3.70-3.40 (2H, m, H_β), 1.43 (9H, s, C(C*H*$_3$)$_3$). **δ_C /ppm** (125 MHz, $CDCl_3$): 173.8 (*C*OOH), 157.6 (O*C*=ONH), 156.4 (O*C*=ONH), 136.2 (Ar*C*), 128.7 (Ar*C*H), 128.3 (Ar*C*H), 128.2 (Ar*C*H), 80.9 (*C*(CH_3)$_3$), 67.4 (*C*H_2Ph), 54.6 (C_α), 42.8(C_β), 28.4 (C(*C*H_3)$_3$). **v_{max} /cm^{-1}:** 3342 (m, N-H), 2979 (m, C-H), 1688 (s, C=O), 1516 (s, N-H). **HRMS** (ESI+) *m/z* found $[M+Na]^+$ 361.1393, $C_{16}H_{22}N_2O_6Na^+$ required 361.1376 (Δ -4.7 ppm). **$[\alpha]_D^{25}$** = -8.5 (*c* 0.86, MeOH). Spectroscopic data is consistent with literature values.[7]

(*S*)-methyl 3-(((benzyloxy)carbonyl)amino)-2-((*tert*-butoxycarbonyl) amino)propanoate

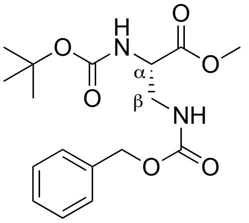

Boc-Dap(Cbz)-OH (1.50 g, 4.43 mmol) was dissolved in anhydrous CH_2Cl_2 (14 ml) and EDC.HCl (0.849 g, 4.43 mmol), anhydrous MeOH (0.72 ml, 18 mmol) and DMAP (0.054 g, 0.44 mmol) were added. The reaction was stirred at rt for 3 h. H_2O (10 ml) was added and the organic and aqueous layers were separated. The organic layer was washed with H_2O (10 ml), saturated $NaHCO_3$ solution (2 × 10 ml), 5% citric acid (2 × 10 ml) and H_2O (2 × 10 ml). The organic fraction was dried ($MgSO_4$) and the solvent removed under reduced pressure. Purification by column chromatography (5-10% MeOH/ CH_2Cl_2) gave the title compound (1.24 g, 79%) as a clear oil.

$\mathbf{R_f}$ = 0.73 (10% MeOH/ 90% CH_2Cl_2). **δ_H /ppm** (400 MHz, $CDCl_3$): 7.39-7.26 (5H, m, 5 × ArC*H*), 5.42 (1H, br s, C_α-N*H*), 5.14 (1H, br s, C_β-N*H*), 5.09 (2H, s, C*H*$_2$Ph), 4.42-4.31 (1H, m, H_α), 3.73 (3H, s, OC*H*$_3$), 3.64-3.53 (2H, m, H_β), 1.43 (9H, s, C(C*H*$_3$)$_3$). **δ_C /ppm** (125 MHz, $CDCl_3$): 171.3 (*C*OOMe), 156.8 (O*C*=ONH), 155.6 (O*C*=ONH), 136.4 (Ar*C*), 128.7 (Ar*C*H), 128.3 (Ar*C*H), 128.3 (Ar*C*H), 80.4 (*C*(CH$_3$)$_3$), 67.1 (*C*H$_2$Ph), 54.1 (C_α), 52.8 (O*C*H$_3$), 43.1 (C_β), 28.4 (C(*C*H$_3$)$_3$). **v_{max} /cm^{-1}:** 3343 (m, N-H), 2976 (m, C-H), 1694 (s, C=O), 1515 (s, N-H). **HRMS** (ESI+) *m/z* found $[M+H]^+$ 353.1716, $C_{17}H_{25}N_2O_6^+$ required 353.1713 (Δ 0.8 ppm). **$[\alpha]_D^{25}$** = -9.1 (*c* 1.85, MeOH). Spectroscopic data is consistent with literature values.[2a, 7a]

(*S*)-methyl 3-amino-2-((*tert*-butoxycarbonyl)amino)propanoate

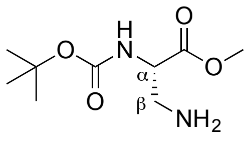

To a solution of (*S*)-methyl 3-(((benzyloxy)carbonyl)amino)-2-((*tert*-butoxycarbonyl) amino)propanoate (0.300 g, 0.851 mmol) in MeOH (3.3 ml) was added 5% w/w Pd/C (15 mg). The suspension was stirred under an atmosphere of hydrogen gas (balloon pressure) for 18 h and then filtered through a bed of celite®. The solvent was removed under reduced pressure and the crude mixture was purified by column chromatography (2-5% MeOH/ 1% 7N NH_3 in MeOH/ CH_2Cl_2) to furnish the title compound as a clear oil (0.180 g, 73%).

$\mathbf{R_f}$= 0.44 (10% MeOH/ 1% 7N NH_3 in MeOH/ 89% CH_2Cl_2). **δ_H /ppm** (500 MHz, $CDCl_3$): 5.36 (1H, br s, C_α-N*H*), 4.36-4.26 (1H, m, H_α), 3.77 (3H, s, OC*H*$_3$), 3.05 (2H, d, *J*=4.3 Hz, H_β), 1.46 (9H, s, C(C*H*$_3$)$_3$). **δ_C /ppm** (125 MHz, $CDCl_3$): 172.3 (*C*OOMe), 155.7 (O*C*=ONH), 80.2 (*C*(CH$_3$)$_3$), 56.0 (C_α), 52.6 (O*C*H$_3$), 44.1 (C_β), 28.5 (C(*C*H$_3$)$_3$). **v_{max} /cm^{-1}:** 3315 (m, N-H), 2977 (m, C-H), 1695 (s, C=O), 1511 (s, N-H). **HRMS** (ESI+) *m/z* found $[M+Na]^+$ 241.1155, $C_9H_{18}N_2O_4Na^+$ required 241.1159 (Δ -1.4 ppm). **$[\alpha]_D^{25}$** = +6 (*c* 0.12, MeOH). Spectroscopic data is consistent with literature values.[8]

(*S*)-2-azido-3-phenylpropanoic acid

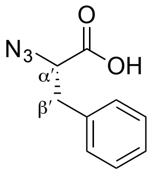

3-Azidosulfonyl-3*H*-imidazole-1-ium hydrogen sulfate (8.01 g, 25.4 mmol) was added to a mixture of L-phenylalanine (3.50 g, 21.2 mmol), K_2CO_3 (7.91 g, 57.2 mmol) and copper sulfate pentahydrate (0.052 g, 0.21 mmol) in MeOH (105 ml). The reaction was stirred at rt for 18 h. The solvent was removed under reduced pressure and the crude material diluted with H_2O (100 ml) and acidified with conc. HCl to a pH of 2. The aqueous layer was extracted with EtOAc (3 × 100 ml) and the combined organic fractions were dried ($MgSO_4$) and the solvent removed under

reduced pressure. The crude oil was purified by column chromatography (1% AcOH/ 25% EtOAc / 74% Pet ether) to yield the title compound as a yellow oil (3.62 g, 89%).

R_f = 0.30 (1% AcOH/ 25% EtOAc / 74% Pet ether 40:60). **δ_H /ppm** (500 MHz, $CDCl_3$): 9.76 (1H, br s, COO*H*), 7.37-7.24 (5H, m, 5 × ArC*H*), 4.17 (1H, dd, *J*=8.9, 5.0 Hz, $H_{\alpha'}$), 3.25 (1H, dd, *J*=14.1, 5.0 Hz, $H_{\beta'}$), 3.05 (1H, dd, *J*=14.1, 8.9 Hz, $\underline{H}_{\beta'}$). **δ_C /ppm** (125 MHz, $CDCl_3$): 175.7 (*C*OOH), 135.7 (Ar*C*), 129.4 (Ar*C*H), 128.9 (Ar*C*H), 127.6 (Ar*C*H), 63.2 ($C_{\alpha'}$), 37.7 ($C_{\beta'}$). **v_{max} /cm^{-1}:** 3031 (m, C-H), 2105 (s, N_3), 1715 (s, C=O). **HRMS** (ESI-) *m/z* found $[M-H]^-$ 190.0617, $C_9H_8N_3O_2^-$ required 190.0611 (Δ 3.4 ppm). **$[\alpha]_D^{25}$** = -72.0 (*c* 0.25, MeOH). Spectroscopic data is consistent with literature values.[9]

(*S*)-methyl-3-((*S*)-2-azido-3-phenylpropanamido)-2-((*tert*-butoxycarbonyl)amino) propanoate

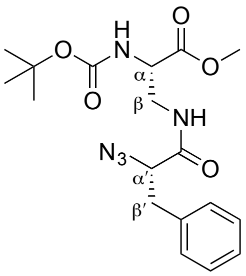

(*S*)-Methyl 3-amino-2-((*tert*-butoxycarbonyl)amino)propanoate (0.150 g, 0.687 mmol) was dissolved in CH_2Cl_2 (1.5 ml) and $HOBt.H_2O$ (0.105 g, 0.687 mmol) and EDC.HCl (0.132 g, 0.687 mmol) were added at rt. Once all the solids were dissolved, (*S*)-2-azido-3-phenylpropanoic acid (0.131 g, 0.687 mmol) in CH_2Cl_2 (1.5 ml) was added and the reaction was stirred for 18 h at rt. The solvent was removed under reduced pressure and the crude mixture was diluted with EtOAc (5 ml) and H_2O (5 ml). The layers were separated and the organic layer was washed with saturated $NaHCO_3$ solution (2 × 5 ml), 5% citric acid (2 × 5 ml) and H_2O (2 × 5 ml). The organic fraction was dried ($MgSO_4$) and the solvent removed under reduced pressure. The crude material was purified by column chromatography (0-5% $MeOH/CH_2Cl_2$) to afford the title compound as an amorphous cream solid (0.140 g, 52%).

R_f = 0.48 (5% MeOH/ 95% CH_2Cl_2). **Mp** = 84-87 °C (5% MeOH/ 95% CH_2Cl_2). **δ_H /ppm** (500 MHz, $CDCl_3$): 7.36-7.22 (5H, m, 5 × ArC*H*), 6.71 (1H br s, C_β-N*H*), 5.40 (1H, d, *J*=5.9 Hz, C_α-N*H*), 4.41-4.33 (1H, m, H_α), 4.16 (1H, dd, *J*=8.5, 4.3 Hz, $H_{\alpha'}$), 3.76 (3H, s, OC*H*$_3$), 3.68-3.53 (2H, m, H_β), 3.32 (1H, dd, *J*=14.1, 4.3 Hz, $H_{\beta'}$), 2.96 (1H, dd, *J*=14.1, 8.5 Hz, $\underline{H}_{\beta'}$), 1.45 (9H, s, C(C*H*$_3$)$_3$). **δ_C /ppm** (125 MHz, $CDCl_3$): 171.0 (*C*=ONH), 169.7 (*C*OOMe), 155.7 (O*C*=ONH), 136.3 (Ar*C*), 129.6 (Ar*C*H), 128.8 (Ar*C*H), 127.4 (Ar*C*H), 80.6 (*C*(CH$_3$)$_3$), 65.7 ($C_{\alpha'}$), 53.7 (C_α), 52.9 (O*C*H$_3$), 41.8 (C_β), 38.7 ($C_{\beta'}$), 28.4 (C(*C*H$_3$)$_3$). **v_{max} /cm^{-1}:** 3308 (m, N-H), 3003 (w, C-H), 2117 (s, N_3) 1750 (s, C=O), 1691 (s, C=O), 1652 (s, C=C). **HRMS** (ESI+) *m/z* found $[M+H]^+$ 392.1944, $C_{18}H_{26}N_5O_5^+$ required 392.1934 (Δ 2.5 ppm). **$[\alpha]_D^{25}$** = +10.0 (*c* 0.26, MeOH).

(*S*)-3-((*S*)-2-azido-3-phenylpropanamido)-1-methoxy-1-oxopropan-2-aminium chloride (Building Block B)

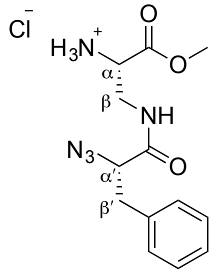

TMSCl (4.70 ml, 0.0370 mmol) was added dropwise to MeOH (19.7 ml) at 0 °C. The solution was stirred for 10 min at 0 °C and then added dropwise to (*S*)-methyl-3-((*S*)-2-azido-3-phenylpropanamido)-2-((*tert*-butoxycarbonyl)amino) propanoate (2.39 g, 6.11 mmol) at rt. The solution was stirred at rt. TLC analysis after 3 h indicated that all the starting material had been consumed. The solvent was removed under reduced pressure following which co-evaporations with MeOH (20 ml) and CH_2Cl_2 (3 × 20 ml) gave the title compound as an amorphous cream solid (1.98 g, 99%).

Mp = 148-152 °C (CH_2Cl_2). **δ_H /ppm** (400 MHz, d_6-DMSO): 8.90 (1H, t, *J*=5.8 Hz, C_β-N*H*), 8.76 (3H, s, NH_3^+), 7.34-7.13 (5H, m, 5 × ArC*H*), 4.12-4.00 (2H, m, Hα and $H_{\alpha'}$), 3.67 (3H, s, OCH_3), 3.65-3.50 (2H, m, H_β), 3.14 (1H, dd, *J*=14.1, 4.2 Hz, $H_{\beta'}$), 2.86 (1H, dd, *J*=14.1, 10.1 Hz, $\underline{H}_{\beta'}$). **δ_C /ppm** (101 MHz, d_6-DMSO): 170.0 (*C*OOMe), 168.1 (*C*=ONH), 137.1 (Ar*C*), 129.1 (Ar*C*H), 128.4 (Ar*C*H), 126.7 (Ar*C*H), 62.9 ($C_{\alpha'}$), 53.0 (O*C*H_3), 51.8 (C_α), 38.6 (C_β), 37.0 ($C_{\beta'}$). **ν_{max} /cm^{-1}:** 3333 (m, N-H), 2830 (s, C-H), 2100 (s, N_3), 1740 (s, C=O), 1657 (s, C=O), 1529 (s, C=C). **HRMS** (ESI+) *m/z* found $[M+H]^+$ 292.1417, $C_{13}H_{18}N_5O_3^+$ required 292.1410 (Δ 2.4 ppm). **$[\alpha]_D^{25}$** = +12.0 (*c* 0.29, MeOH).

Building Block C

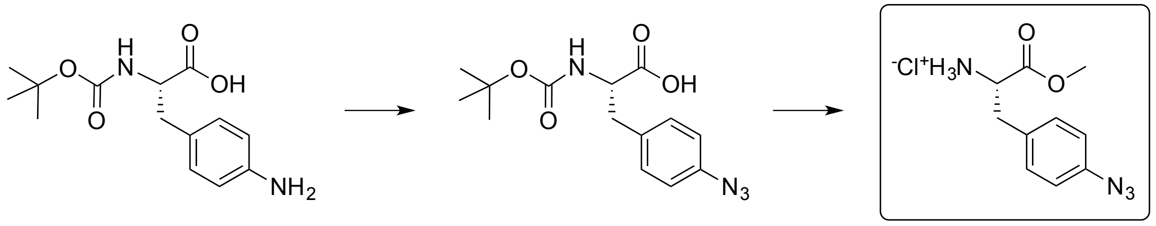

SF7: Synthetic Route to Building Block C

(*S*)-3-(4-azidophenyl)-2-((*tert*-butoxycarbonyl)amino)propanoic acid

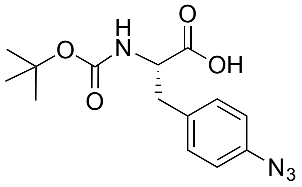

3-Azidosulfonyl-3*H*-imidazole-1-ium hydrogen sulfate (2.13 g, 7.87 mmol) was added to a mixture of Boc-Phe(4-NH_2)-OH (1.84 g, 6.56 mmol), K_2CO_3 (2.06 g, 14.9 mmol) and copper sulfate pentahydrate (16.4 mg, 0.0656 mmol) in MeOH (33 ml). The reaction was stirred at rt for 18 h. The solvent was removed under reduced pressure and the crude material diluted with H_2O (20 ml) and acidified with conc. HCl to a pH of 2. The aqueous layer was extracted with EtOAc (3 × 20 ml) and the combined organic fractions were dried ($MgSO_4$) and the solvent removed under reduced pressure. The crude oil was purified by column chromatography (1% AcOH/ 5% MeOH / 94% CH_2Cl_2) to yield the title compound as an orange oil (1.76 g, 73%).

R_f = 0.34 (5% MeOH/ 1% AcOH/ 94% CH_2Cl_2). **δ_H /ppm** (500 MHz, *d*$_6$-DMSO): 7.17 (2H, d, *J*=8.4 Hz, 2 × ArC*H*), 6.97 (2H, d, *J*=8.4 Hz, 2 × ArC*H*), 4.93 (1H, d, *J*=7.3 Hz, BocN*H*), 4.58 (1H, m, H_α), 3.18 (1H, dd, *J*=13.9 Hz and 5.1 Hz, H_β), 3.06 (1H, dd, *J*=13.9 Hz and 6.5 Hz, H_β) 1.44 (9H, s, C(C*H*$_3$)$_3$). **δ_C /ppm** (125 MHz, *d*$_6$-DMSO): 175.2 (*C*OOH), 155.5 (Boc *C*=O), 139.1 (Ar*C*), 132.7 (Ar*C*), 130.9 (Ar*C*H), 119.4 (Ar*C*H), 80.7(*C*(CH$_3$)$_3$), 54.4 (C_α), 37.3 (C_β), 28.4 (C(*C*H$_3$)$_3$). **ν_{max} /cm^{-1}:** 2979 (w, O-H), 2112 (s, N_3), 1687 (s, C=O), 1506 (s, N-H). **HRMS** (ESI+) *m/z* found $[M+H]^+$ 307.1386, $C_{14}H_{19}N_4O_4^+$ required 307.1401 (Δ -4.99 ppm). **$[\alpha]_D^{25}$** = +39.9 (*c* 0.51, $CHCl_3$). Spectroscopic data is consistent with literature values.[10]

(*S*)-3-(4-azidophenyl)-1-methoxy-1-oxopropan-2-aminium chloride (Building Block C)

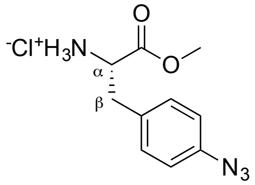

TMSCl (1.57 ml, 12.4 mmol) was added dropwise over 15 min to a solution of the azide (800 mg, 2.61 mmol) in MeOH (3 ml) at 0 °C. The reaction was allowed to warm to rt and stirred for 5h. Dry diethyl ether (4.6 ml) was added and the slurry was stirred for 30 min and then filtered. The precipitate was washed with cold diethyl ether (2 × 2 ml) and then dried *in vacuo* to afford the title compound as a cream solid (596 mg, 89%).

Mp = 183-186 °C (Et_2O). **δ_H /ppm** (500 MHz, *d*$_6$-DMSO): 8.65 (3H, s, NH_3^+), 7.33-7.21 (2H, m, 2 × ArC*H*), 7.15-7.04 (2H, m, 2 × ArC*H*), 4.26 (1H, t, *J*=6.4 Hz, H_α), 3.67 (3H, s, OC*H*$_3$), 3.17 (1H, d, *J*=14.1 Hz and 6.0 Hz, H_β), 3.09 (1H, dd, J=14.1 Hz and 7.2 Hz, $\underline{H}_\beta$). **δ_C /ppm** (125 MHz, *d*$_6$-DMSO): 169.3 (*C*OOMe), 138.4 (Ar*C*), 131.6(Ar*C*), 131.1 (Ar*C*H), 119.3 (Ar*C*H), 53.2 (C_α), 52.7 (O*C*H$_3$), 35.2 (C_β). **ν_{max} /cm^{-1}:** 2805 (m, C-H), 2123 (m, N_3), 1742 (s, C=O), 1578 (m, C=C), 1509 (s, N-H). **HRMS** (ESI+) *m/z* found $[M+H]^+$ 221.1027, $C_{10}H_{13}N_4O_2^+$ required 221.1033 (Δ -2.7 ppm). **$[\alpha]_D^{25}$** = +15.1 (*c* 0.56, MeOH).

Building Block D

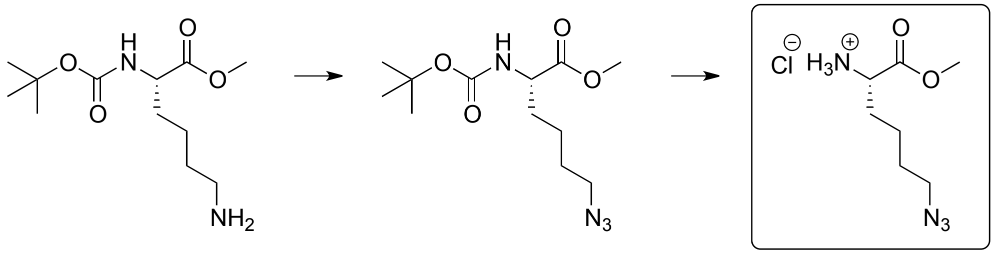

SF8: Synthetic Route to Building Block D

(*S*)-6-azido-1-methoxy-1-oxohexan-2-aminium chloride (Building Block D)

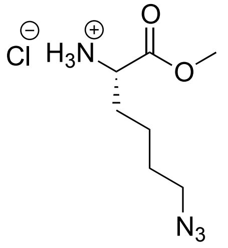

Imidazole-1-sulfonyl azide hydrochloride (2.2 g, 10.52 mmol) was added to a mixture of Boc-L-Lys-OMe (2.28 g, 8.77 mmol), $CuSO_4.5H_2O$ (21.9 mg, 0.09 mmol) and K_2CO_3 (2.06 g, 14.9 mmol) in MeOH (45 mL). The reaction mixture was stirred overnight at room temperature. The solvent was removed under reduced pressure followed by addition of H_2O (60 mL); the mixture was acidified to pH 2-4 with 5% citric acid and extracted with EtOAc (3 x 80 mL). The organic fractions were dried over $MgSO_4$ and the solvent evaporated under reduced pressure. The resulting orange oil was purified by column chromatography yielding 1.74 g of Boc-L-Lys(N_3)-OMe. The Boc group was removed by following GP4 to yeild the desired product as a white solid (1.24g, 73% yield over 2 steps).

δ_H /ppm (500 MHz, *d*$_6$-DMSO): 8.59 (3H, bs), 4.09-3.96 (1H, m), 3.75 (3H, s), 3.40-3.20 (2H, m), 1.86-1.76 (2H, m), 1.59-1.26 (4H, m). **δ_C /ppm** (100 MHz, *d*$_6$-DMSO): 170.8, 53.7, 52.6, 51.2, 30.4, 28.6, 22.4. **v_{max} /cm^{-1}:** 3424, 2925, 2870, 2093, 1743, 1596, 1509, 1231. **HRMS** (ESI+) *m/z* found $[M+H]^+$ 187.1187, $C_7H_{15}N_4O_2^+$ required 187.1195.

Building Block E

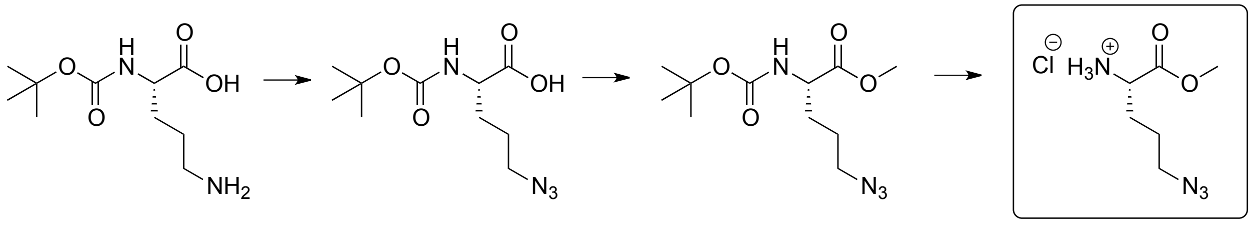

SF9: Synthetic Route to Building Block E

(*S*)-5-azido-1-methoxy-1-oxopentan-2-aminium chloride (Building Block E)

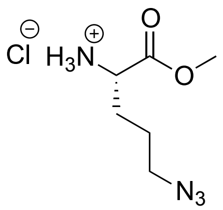

Imidazole-1-sulfonyl azide hydrochloride (4.78 g, 22.8 mmol) was added to a mixture of Boc-L-Orn-OH (4.41 g, 19 mmol), $CuSO_4.5H_2O$ (47.4 mg, 0.19 mmol), and K_2CO_3 (7.09 g, 51.3 mmol). The reaction mixture was stirred overnight at room temperature. The solvent was removed under reduced pressure followed by addition of H_2O (75 mL); the mixture was acidified to pH 2-4 with 5% citric acid and extracted with EtOAc (3 x 150 mL). The organic fractions were dried over $MgSO_4$ and the solvent evaporated under reduced pressure to yeild Boc-Orn(N3)-OH as a yellow oil. Formation of the corresponding methyl ester was achieved by following a similar procedure to (*S*)-methyl-3-((*S*)-2-azido-3-phenylpropanamido)-2-((*tert*-butoxycarbonyl)amino) propanoate. The Boc group was removed by following GP4 to yield the desired product as a white solid (2.84 g, 72% yield over 3 steps).

δ_H /ppm (400 MHz, *d_6*-DMSO): 8.72 (3H, bs), 4.15-3.94 (1H, m), 3.74 (3H, s), 3.44-3.26 (2H, m), 1.90-1.81 (2H, m), 1.79- 1.51 (2H, m). **δ_C /ppm** (100 MHz, *d_6*-DMSO): 170.2, 53.3, 51.9, 50.4, 27.7, 24.4. **v_{max} /cm^{-1}:** 3377, 2970, 2902, 2093, 1744, 1509, 1440, 1280, 1057. **HRMS** (ESI+) *m/z* found $[M+Na]^+$ 195.0867, $C_6H_{12}N_4O_2Na^+$ required 195.0858.

Building Block F

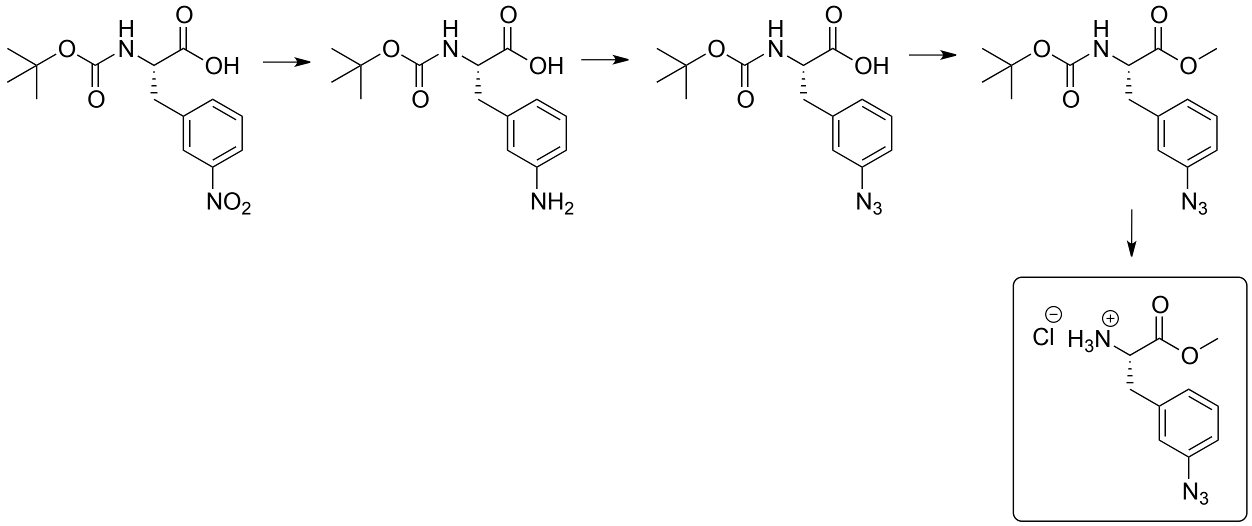

SF9: Synthetic Route to Building Block F

(*S*)-3-(3-azidophenyl)-1-methoxy-1-oxopropan-2-aminium chloride (Building Block F)

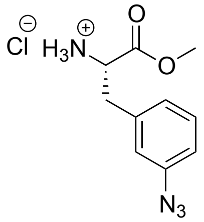

N-Boc-3-nitro-L-phenylalanine (610 mg, 1.95 mmol) was dissolved in 25 mL of MeOH, following by addiction of K_2CO_3 (323 mg, 2.34 mmol) and 10% Pd on charcoal. The reaction mixture was hydrogenated at room temperature under 55 psi. After 4 hours the reaction is stopped, filtered over Celite and evaporated to dryness to yield the potassium salf of N-Boc-3-amine-L-phenylalanine as a white solid (672 mg), this intermediate was used in the next reaction without further purification. N-Boc-3-amine-L-phenylalanine potassium salt (672 mg, 1.95 mmol), immidazol sulfonyl azide bisulfate (635 mg, 2.34 mmol), $CuSO_4$ pentahydrate (5 mg, 0.0195 mmol), were dissolved in 12 mL of MeOH, and the reaction mixture was stirred at room temperature for 18 hours. The solvent was evaporated and 6 mL of water was added to the slurry. The aqueous phase was acidified with 1M HCl until pH 3 and extracted with EtOAc (3 x 15 mL); the organic phase was dried over $MgSO_4$ and purified by flash column (DCM/MeOH/AcOH 95/5/0.6) to give N-Boc-3-azide-L-phenylalanine as a pink solid (330 mg). Formation of the corresponding methyl ester was achieved by following a similar procedure to (*S*)-methyl-3-((*S*)-2-azido-3-phenylpropanamido)-2-((*tert*-butoxycarbonyl)amino) propanoate. The Boc group was removed by following GP4 to yield the desired product as a violet solid (261 mg, 94% yield).

Mp = 160-162 °C (CH_2Cl_2). **δ_H /ppm** (500 MHz, d_6-DMSO): 8.52 (3H, br), 7.37 (1H, t, *J*=7.9 Hz), 7.06 – 6.97 (3H, m), 4.34 (1H, t, *J*=6.6 Hz), 3.69 (3H, s), 3.13 (2H, d, *J*=6.7 Hz). **δ_C /ppm** (100 MHz, d_6-DMSO): 169.4, 139.7, 136.8, 130.4, 126.4, 120.2, 118.3, 53.1, 52.9, 35.6. **ν_{max} /cm^{-1}:** 2825, 2628, 2106, 1735, 1593, 1578, 1492, 1479, 1441, 1390, 1290, 1242, 1211, 1140, 1082. **HRMS** (ESI+) *m/z* found $[M+H]^+$ 221.1047, $C_{10}H_{13}N_4O_2^+$ required 221.1039. **$[\alpha]_D^{26.2}$** = +9.0 (*c* 0.000867, MeOH).

7. Synthesis of Alkyne-Acid Building Blocks

Building block 1

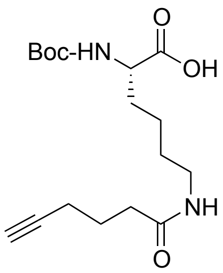

Boc-L-Lys-OH (1.72 g, 7 mmol) was suspended in dry DMF (25 mL) and DIPEA (1.27 mL, 7.7 mmol) was added followed by a solution of 5-hexynoic acid (1.61 g, 7.7 mmol) in dry DMF (10 mL). The solution was stirred for 5 min at rt followed by addition of extra DIPEA (1.27 mL, 7.7 mmol) and further stirring for 4h at rt. Most of the DMF was removed under reduced pressure (T= 35-40°C) and H_2O (50 mL) was added and the pH adjusted to 8-9 wIth saturated aqueous Na_2CO_3. The resulting mixture was washed with diethyl ether (3 x 25 mL). The pH of the aqueous phase was adjusted to pH 2-3 with concentrated HCl and extracted with EtOAc (4 x 30 mL). The combined organic fractions were dried with $MgSO_4$ and evaporated to dryness to yield the product as a yellow oil (2.45 g, 80% purity, 82.3% yield).

δ_H /ppm (500 MHz, DMSO-*d6*): 12.40 (1H, bs), 7.80 (1H, t, *J*=5.6 Hz), 7.02 (1H, d, *J*=8.0 Hz), 3.83-3.77 (1H, m), 3.04-2.95 (2H, m), 2.77 (1H, t, *J*=2.7 Hz), 2.17-2.09 (4H, m), 1.69-1.58 (3H, m), 1.58-1.48 (1H, m), 1.44-1.18 (4H, m), 1.37 (9H, s). **δ_C /ppm** (125 MHz, DMSO-*d6*): 174.3, 172.8, 171.2, 155.6, 84.1, 78.0, 71.5, 53.4, 38.2, 34.2, 30.4, 28.8, 28.2, 24.3, 23.1, 17.4. **ν_{max} /cm^{-1}:** 3359, 2941, 1720, 1683, 1626, 1525, 1436, 1160. **HRMS** (ESI+) *m/z* found $[M+Na]^+$ 363.1908, $C_{17}H_{28}N_2O_5Na^+$ required 363.1896.

Building block 2

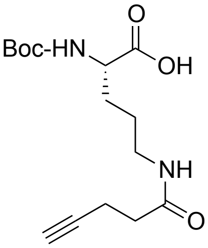

A procedure analogous to the one used for the synthesis of building block 1, except using Boc-L-Orn-OH and 4-pentynoic acid as starting materials afforded building block 2 as a yellow oil (1.61 g, 80% purity, 98% yield).

δ_H /ppm (500 MHz, DMSO-*d6*): 12.44 (1H, bs), 7.85 (1H, t, *J*=5.5 Hz), 7.05 (1H, d, *J*=8.0 Hz), 3.86-3.78 (1H, m), 3.05-2.97 (2H, m), 2.73 (1H, t, *J*=2.6 Hz), 2.34 (2H, td, *J*=7.5, 2.6 Hz), 2.24

(2H, t, J=7.5 Hz), 1.68-1.61 (1H, m), 1.56-1.30 (3H, m), 1.37 (9H, s). **δ_C /ppm** (125 MHz, DMSO-d_6): 174.1, 172.8, 170.1, 155.6, 83.8, 78.0, 71.3, 53.3, 39.5, 34.3, 28.3, 28.2, 26.0, 14.3. **v_{max} /cm^{-1}:** 3285, 3086, 2978, 2933, 1738, 1695, 1626, 1550, 1162. **HRMS** (ESI+) *m/z* found $[M+H]^+$ 313.1762, $C_{15}H_{25}N_2O_5^+$ required 313.1763.

Building block 3

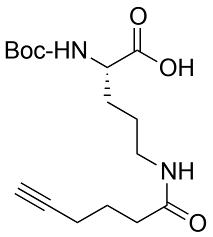

A procedure analogous to the one used for the synthesis of building block 1, except using Boc-L-Orn-OH and 5-hexynoic acid as starting materials afforded building block 3 as a yellow oil (1.76 g, 82% purity, 88% yield).

δ_H /ppm (400 MHz, DMSO-d_6): 12.44 (1H, bs), 7.81 (1H, t, J=5.4 Hz), 7.08 (1H, d, J=8.0 Hz), 3.87-3.81 (1H, m, J=4.0 Hz), 3.11-2.95 (2H, m), 2.79 (1H, t, J=2.6 Hz), 2.21-2.10 (4H, m), 1.72-1.57 (3H, m), 1.58- 1.30 (3H, m), 1.39 (9H, s, 9H). **δ_C /ppm** (100 MHz, DMSO-d_6): 174.6, 171.7, 156.3, 84.6, 78.4, 72.0, 53.8, 38.5, 34.6, 28.7, 28.7, 26.4, 24.8, 17.9. **v_{max} /cm^{-1}:** 3309, 2931, 1701, 1641, 1525, 1367, 1161. **HRMS** (ESI+) *m/z* found $[M+H]^+$ 327.1935, $C_{16}H_{27}N_2O_5^+$ required 327.1920.

Building block 4

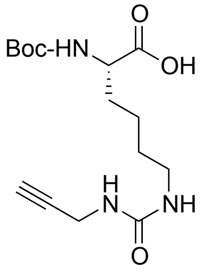

Boc-*L*-Lys(Z)-OH (2.70 g, 7.10 mmol) was dissolved in MeOH (25 mL) and hydrogenated overnight over 5% palladium charcoal (135 mg) at room temperature and pressure. The mixture was filtered through a pad of Celite and the solvent was removed under reduced pressure. The slurry was co-evaporated with CH_2Cl_2 to give Boc-L-Lys-OH as a white solid which was used without further purification (1.68 g). 4-Nitrophenyl prop-2-yn-1-ylcarbamate (CP2, 1.36 g, 6.17 mmol), Boc-L-Lys-OH (1.67 g, 6.79 mmol), and CH_2Cl_2 (60 mL) was added to a round bottomed flask equipped with a stir bar. Et_3N (1.80 mL, 13.0 mmol) was added to give a yellow suspension. The mixture was stirred at RT overnight. The solvent was evaporated, and the residue purified by flash column chromatography (0-1% acetic acid in EtOAc). Co-evaporations with toluene were performed to give a white solid (1.56 g, 74% yield over 2 steps).

δ_H /ppm (400 MHz, d_6-DMSO): 12.38 (1H, br s), 7.01 (1H, d, *J*=8.0 Hz), 6.10 (1H, t, *J*=5.5 Hz), 5.95 (1H, t, *J*=5.5 Hz), 3.85-3.78 (1H, m), 3.77 (2H, dd, *J*=5.5, 2.5 Hz), 3.01 (1H, t, *J*=2.5 Hz), 2.99-2.91 (2H, m), 1.70-1.42 (2H, m), 1.38 (9H, s), 1.39-1.20 (4H, m). **δ_C /ppm** (100 MHz, d_6-DMSO): 174.2, 157.4, 155.5, 82.5, 77.8, 72.4, 53.4, 38.6, 30.4, 29.5, 28.7, 28.1, 22.9. **ν_{max} /cm^{-1}:** 3388, 3304, 2939, 2162, 1729, 1684, 1622, 1530, 1209, 1159. **HRMS** (ESI+) *m/z* found $[M+H]^+$ 328.1880, $C_{15}H_{25}N_3O_5^+$ required 328.1872. **$[\alpha]_D^{25}$** = +5.6 (*c* 0.51, MeOH).

Building block 5

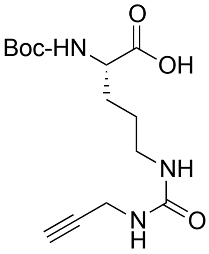

A procedure analogous to the one used for the synthesis of building block 4, except using Boc-(Z)-Orn-OH as starting material afforded building block 5 as a clear oil (832 mg, 54% yield over 2 steps).

δ_H /ppm (500 MHz, d_6-DMSO): 12.45 (1H, br s), 7.06 (1H, d, *J*=8.0 Hz), 6.12 (1H, t, *J*=5.5 Hz), 5.98 (1H, t, *J*=5.5 Hz), 3.83 (1H, td, *J*=8.5, 4.5 Hz), 3.76 (2H, dd, *J*=6.0, 2.5 Hz), 3.02 (1H, t, *J*=2.5 Hz), 2.95 (2H, dd, *J*=12.80, 6.51 Hz), 1.71-1.56 (1H, m), 1.37 (9H, s), 1.55-1.31 (3H, m). **δ_C /ppm** (125 MHz, d_6-DMSO): 174.1, 157.4, 155.5, 82.5, 77.9, 72.4, 53.3, 38.9, 28.7, 28.2, 28.1, 26.7. **ν_{max} /cm^{-1}:** 3296, 2930, 2163, 1979, 1633, 1563, 1245, 1159. **HRMS** (ESI+) *m/z* found $[M+H]^+$ 314.1723, $C_{14}H_{23}N_3O_5^+$ required 314.1716. **$[\alpha]_D^{25}$** = +9.2 (*c* 0.49, MeOH).

Building block 6

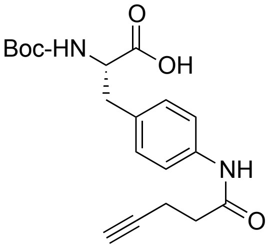

Building block 6 was prepared by literature procedures.[6]

Building block 7

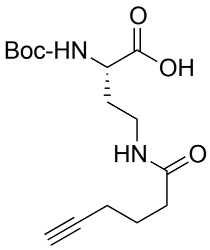

A procedure analogous to the one used for the synthesis of building block 1, except using Boc-L-Dab-OH and 5-hexynoic acid as starting materials afforded building block 7 as a yellow oil (1.36 g, 86% purity, 89% yield).

δ_H /ppm (400 MHz, d_6-DMSO): 12.50 (1H, bs), 7.87 (1H, t, *J*=5.2 Hz), 7.12 (1H, d, *J*=8.1 Hz), 3.99-3.82 (1H, m), 3.20-2.98 (2H, m), 2.80 (1H, t, *J*=2.6 Hz), 2.18-2.12 (4H, m), 1.90-1.75 (1H, m), 1.73-1.58 (3H, m), 1.39 (9H, s). **δ_C /ppm** (100 MHz, d_6-DMSO): 174.5, 171.9, 156.0, 84.6, 78.5, 72.0, 51.8, 36.1, 34.6, 31.2, 28.7, 24.7, 17.9. **v_{max} /cm^{-1}:** 3294, 2979, 2938, 1679, 1601, 1568, 1366, 1215, 1159. **HRMS** (ESI+) *m/z* found $[M+Na]^+$ 335.1584, $C_{15}H_{24}N_2O_5Na^+$ required 335.1583.

Building block 8

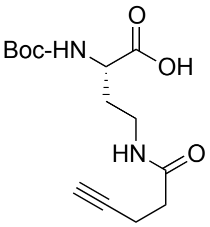

A procedure analogous to the one used for the synthesis of building block 1, except using Boc-L-Dab-OH and 4-pentynoic acid as starting materials afforded building block 8 as a yellow oil (1.28 g, 83% purity, 85% yield).

δ_H /ppm (500 MHz, d_6-DMSO): 12.53 (1H, bs), 7.93 (1H, t, *J*=5.2 Hz), 7.12 (1H, d, *J*=8.1 Hz), 3.95–3.90 (1H, m), 3.28-3.06 (2H, m), 2.76 (1H, t, *J*=2.5 Hz), 2.36 (2H, td, *J*=6.5, 1.8 Hz), 2.27 (2H, t, *J*=7.2 Hz), 1.89-1.76 (1H, m), 1.75-1.61 (1H, m), 1.39 (9H, s). **δ_C /ppm** (125 MHz, d_6-DMSO): 174.8, 171.1, 156.4, 84.6, 78.9, 72.2, 52.2, 36.6, 35.02, 31.4, 29.1, 15.09. **v_{max} /cm^{-1}:** 3296, 2975, 2930, 1703, 1628, 1542, 1365, 1215, 1160. **HRMS** (ESI+) *m/z* found $[M+H]^+$ 299.1598, $C_{14}H_{23}N_2O_5^+$ required 299.1607.

Building block 9

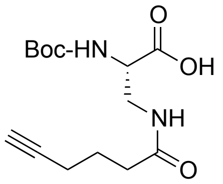

A procedure analogue to the one used for the synthesis of building block 10, except using hexynoyl-OSu instead of pentynoyl-OSu afforded building block 9 as a white solid (1.42 g, 95% yield).

δ_H /ppm (400 MHz, d_6-DMSO): 12.56 (1H, br s), 7.91 (1H, t, *J*=5.5 Hz), 6.92 (1H, d, *J*=8.0 Hz), 4.03 (1H, dt, *J*=7.5, 5.0 Hz), 3.43-3.23 (2H, m), 2.76 (1H, t, *J*=2.5 Hz), 2.17-2.10 (4H, m), 1.64 (2H, p, *J*=7.0 Hz), 1.38 (9H, s). **δ_C /ppm** (100 MHz, d_6-DMSO): 172.2, 172.0, 155.2, 84.0, 78.1, 71.3, 53.4, 39.7, 34.0, 28.1, 24.2, 17.2. **v_{max} /cm^{-1}:** 3291, 2979, 1739, 1679, 1611, 1534, 1364, 1296, 1240, 1159. **HRMS** (ESI+) *m/z* found $[M+H]^+$ 299.1612, $C_{14}H_{23}N_2O_5^+$ required 299.1607. **$[\alpha]_D^{25}$** = -3.2 (*c* 0.49, MeOH).

Building block 10

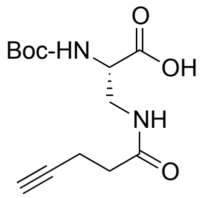

To a suspension of Boc-*L*-Dap-OH (1.00 g, 4.91 mmol), in dry DMF (20 mL), was added a fraction of DIPEA (940 µL, 5.4 mmol). A solution of pentynoyl-OSu (1.15 g, 5.90 mmol) in dry DMF (10 mL) was then added dropwise over 10 minutes. After 5 minutes a second fraction of DIPEA (940 µL, 5.4 mmol) was added and the mixture was stirred at rt for 5 hours. The organic solvent was removed under reduced pressure and the slurry was diluted with H_2O (25 mL). The pH was adjusted to 8-9 by addition of saturated Na_2CO_3 and washings with Et_2O (3 x 15 mL) were performed. The aqueous layer was then acidified to pH 2-3 with conc. HCl and extracted with EtOAc (3 x 20 mL). The organic extracts were dried (MgSO4) and evaporated to dryness under reduced pressure. After precipitations in Et_2O, building block 10 was obtained as a white solid in 89% yield.

δ_H /ppm (400 MHz, d_6-DMSO): 12.57 (1H, br s), 8.02-7.93 (1H, m), 6.94 (1H, d, *J*=8.5 Hz), 4.02 (1H, dt, *J*=7.5, 5.0 Hz), 3.41 (1H, td, *J*=13.3, 5.2 Hz), 3.36-3.23 (1H, m), 2.73 (1H, t, *J*=2.5 Hz), 2.38-2.22 (4H, m), 1.38 (9H, s). **δ_C /ppm** (100 MHz, d_6-DMSO): 172.1, 170.7, 155.3, 83.6, 78.2, 71.2, 53.4, 39.8, 34.0, 28.1, 14.1. **v_{max} /cm^{-1}:** 3290, 3265, 3087, 2979, 1738, 1703, 1626, 1556, 1277, 1161. **HRMS** (ESI+) *m/z* found $[M+H]^+$ 285.1461, $C_{13}H_{21}N_2O_5^+$ required 285.1450. **$[\alpha]_D^{25}$** = -5.8 (*c* 0.51, MeOH).

Building block 11

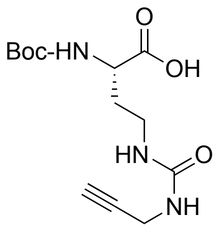

A procedure analogues to the one used for the synthesis of building block 4, except using Boc-Dab-OH as starting material afforded building block 11 as a white foam after flash column chromatography (1% Acetic acid: 9% MeOH: 90% EtOAc) (998 mg, 80% yield).

δ_H /ppm (500 MHz, d_6-DMSO): 12.45 (1H, br s), 7.06 (1H, d, *J*=8.0 Hz), 6.23 (1H, t, *J*=5.5 Hz), 6.03 (1H, t, *J*=5.5 Hz), 3.88-3.82 (1H, m), 3.77 (2H, dd, *J*=5.5, 2.5 Hz), 3.12-3.01 (1H, m), 3.03 (1H, t, *J*=2.5 Hz), 3.00-2.91 (1H, m), 1.82-1.72 (1H, m), 1.65-1.55 (1H, m), 1.38 (1H, s). **δ_C /ppm** (125 MHz, d_6-DMSO): 174.1, 157.4, 155.6, 82.5, 78.0, 72.5, 51.3, 36.3, 31.6, 28.7, 28.1. **v_{max} /cm^{-1}:** 3295, 2979, 1685, 1641, 1563, 1367, 1248, 1157. **HRMS** (ESI+) *m/z* found $[M+Na]^+$ 322.1386, $C_{13}H_{21}N_3O_5Na^+$ required 322.1379. **$[\alpha]_D^{25}$** = -10.5 (*c* 0.53, MeOH).

Building block 12

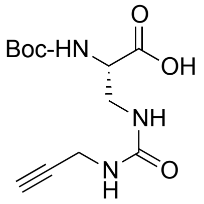

A procedure analogues to the one used for the synthesis of building block 4, except using common precursor 1 as starting material afforded building block 12 as a white foam after flash column chromatography (5% MeOH: 1% AcOH: 94% EtOAc) (1.19 g, 79% yield).

δ_H /ppm (400 MHz, d_6-DMSO): 12.61 (1H, br s), 6.95 (1H, d, *J*=7.5 Hz), 6.46 (1H, t, *J*=5.5 Hz), 6.14-6.06 (1H, m), 3.93-3.81 (1H, m), 3.80-3.75 (2H, m), 3.41 (1H, dt, *J*=13.0, 5.0 Hz), 3.21-3.11 (1H, m), 3.03 (1H, t, *J*=2.5 Hz), 1.38 (9H, s). **δ_C /ppm** (100 MHz, d_6-DMSO): 172.4, 157.5, 155.3, 82.2, 78.0, 72.5, 54.6, 40.5, 28.7, 28.1. **v_{max} /cm^{-1}:** 3376, 3306, 3261, 2979, 1743, 1406, 1627, 1558, 1255, 1160. **HRMS** (ESI+) *m/z* found $[M+H]^+$ 286.1391, $C_{12}H_{20}N_3O_5^+$ required 286.1403. **$[\alpha]_D^{25}$** = -7.1 (*c* 0.62, MeOH).

Building block 13

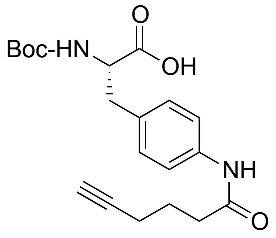

To a solution of 5-hexynoic acid (0.528 mL, 4.67 mmol, 1 eq) and Oxyma pure (634 mg, 4.67 mmol, 1 eq) in dry DMF, was added DCC (964 mg, 4.67 mmol, 1 eq). After an hour of stirring at room temperature, a solution of Boc-L-4-amino-phenylalanine-OH (1.31 g, 4.67 mmol, 1 eq) and DIPEA (1.70 mL, 10.3 mmol, 2.2 eq) in dry DMF was added, and the reaction mixture was stirred overnight. The precipitated dicyclohexylurea was filtered off, and the solvent evaporated under reduced pressure. The slurry was dissolved in 15 mL of EtOAc, and the organic phase was washed with 5% citric acid (2 x 10 mL), dried over $MgSO_4$ and evaporated to dryness. Purification by flash chromathography of the crude product (98.5% DCM: 1.5% MeOH: 1% AcOH) afforded the building block 13 as a white solid (1.13 g, 64% yield).

Mp = 138-140 °C (1: 1.5: 98.5, AcOH/ MeOH/ CH_2Cl_2). **δ_H /ppm** (400 MHz, d_6-DMSO): 9.83 (1H, s), 7.45 (2H, d, *J*=8.4 Hz), 7.15 (2H, d, *J*=7.6 Hz), 7.02 (1H, d, *J*=8.4 Hz), 4.00 (1H, m), 2.90 (1H, dd, *J*= 20.0, 4.0 Hz), 2.79 (1H, t, *J*=2.5 Hz), 2.76-2.70 (1H, m), 2.36 (2H, t, *J*= 7.6 Hz), 2.18 (2H, td, *J*=7.0, 2.6 Hz), 1.75-1.70 (2H, m), 1.30 (9H, s). **δ_C /ppm** (100 MHz, d_6-DMSO): 174.0, 170.9, 155.9, 138.1, 133.0, 129.7, 119.3, 84.5, 78.5, 72.1, 55.8, 36.3, 35.5, 28.6, 24.4, 17.8. **v_{max} /cm^{-1}:** 3549, 3351, 2975, 2162, 2008, 1727, 1706, 1661, 1597, 1523, 1411, 1367, 1308, 1248, 1161, 1057, 1027, 939, 897, 829, 779. **HRMS** (ESI+) *m/z* found $[M+H]^+$ 375.1927, $C_{20}H_{27}N_2O_5^+$ required 375.1920. **$[\alpha]_D^{26.2}$** = +15 (*c* 0.001, MeOH).

Building block 14

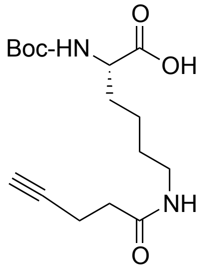

Building block 14 was prepared by literature procedures.[6]

8. Synthesis of B/C/C/P and B/C/C/C/P Coupling Units

See *SF4* and *SF5* for overiew of synthetic routes used.

	Compound	Method, Yield (%), Purity (%)	Analysis
**G**	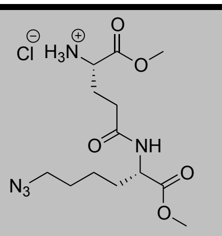	GP1 & GP4<br>71%<br>90%	**δ_H /ppm** (400 MHz, $CDCl_3$): 8.66 (3H, bs), 8.46 (1H, d, *J*=7.3 Hz), 4.23-4.16 (1H, m), 4.04-3.92 (1H, m), 3.73 (3H, s), 3.61 (3H, s), 3.32 (2H, t, *J*=6.8 Hz), 2.46-2.22 (2H, m), 2.06-1.95 (2H, m), 1.75-1.56 (2H, m), 1.58-1.43 (2H, m), 1.43-1.28 (2H, m). **δ_C /ppm** (100 MHz, $CDCl_3$): 173.0, 171.5, 170.2, 53.3, 52.4, 52.3, 51.9, 50.9, 30.8, 30.6, 28.3, 26.4, 23.2. **v_{max} /cm^{-1}:** 3316, 2902, 2095, 1738, 1645, 1528, 1436, 1251, 1227, 1082. **HRMS** (ESI+) *m/z* found $[M+H]^+$ 330.1763, $C_{13}H_{24}N_5O_5^+$ required 330.1772.
**H**	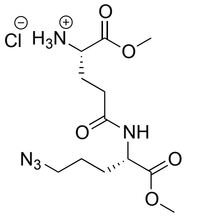	GP1 & GP4<br>84%<br>90%	**δ_H /ppm** (400 MHz, $CDCl_3$): 8.68 (3H, bs), 8.50 (1H, d, *J*=7.3 Hz), 4.27-4.19 (1H, m), 4.03-3.94 (1H, m), 3.73 (3H, s), 3.62 (3H, s), 3.34 (2H, t, *J*=6.7 Hz), 2.45-2.23 (2H, m), 2.06-1.95 (2H, m), 1.80-1.50 (4H, m). **δ_C /ppm** (101 MHz, $CDCl_3$): 172.8, 171.5, 170.1, 53.3, 52.4, 52.1, 51.9, 50.6, 30.6, 28.4, 26.4, 25.3. **v_{max} /cm^{-1}:** 3310, 2958, 2094, 1741, 1645, 1537, 1438, 1224, 1078. **HPLC** (5-100% ACN) *Rt* 10.53 mins. **HRMS** (ESI+) *m/z* found $[M+H]^+$ 316.1609, $C_{12}H_{22}N_5O_5^+$ required 316.1615.
**J**	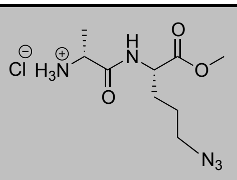	GP1 & GP4<br>98%<br>83%	**HPLC** (5-100% ACN) *Rt* 9.66 mins. **LCMS** $[M+H]^+$ 244.10.
**K**	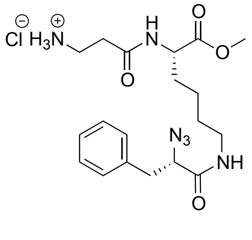	GP1 & GP4<br>90%<br>86%	**HPLC** (5-100% ACN) *Rt* 7.80 mins. **LCMS** $[M+H]^+$ 405.17.
**L**	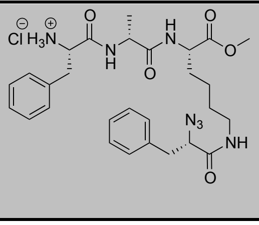	GP1 & GP4<br>89%<br>77%	**HPLC** (5-100% ACN) *Rt* 8.91 mins. **LCMS** $[M+H]^+$ 552.33.

**M**	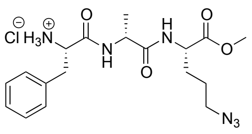	GP1 & GP4<br><br>72%<br><br>81%	**HPLC** (5-100% ACN) *Rt* 7.48 mins. **LCMS** $[M+H]^+$ 391.17.
**N**	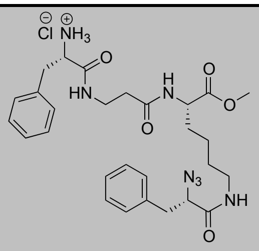	GP1 & GP4<br><br>80%<br><br>78%	**HPLC** (5-100% ACN) *Rt* 8.78 mins. **LCMS** $[M+H]^+$ 552.25.

9. Preparation of B/C/P Linear Amides

	Compound	Method, Yield (%), Purity (%)	Analysis
**A1**	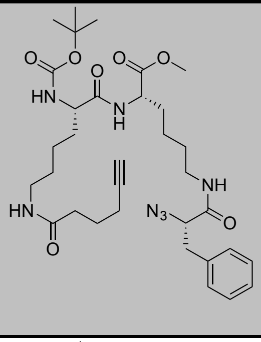	GP1<br>78%<br>88%	**HPLC** (30-100% ACN) *Rt* 8.26 mins. **LCMS** $[M+H]^+$ 656.33.
**A2**	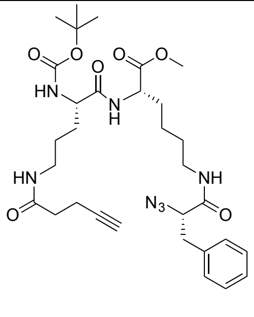	GP1<br>76%<br>89%	**HPLC** (30-100% ACN) *Rt* 7.73 mins. **LCMS** $[M+H]^+$ 628.86.
**A3**	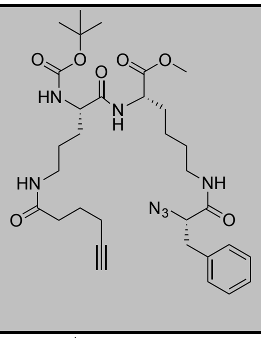	GP1<br>78%<br>88%	**HPLC** (30-100% ACN) *Rt* 8.07 mins. **LCMS** $[M+H]^+$ 642.50.
**A4**	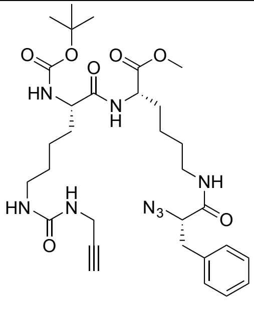	GP1<br>71%<br>96%	**HPLC** (5-100% ACN) *Rt* 10.07 mins. **LCMS** $[M+H]^+$ 643.54.

**A5**	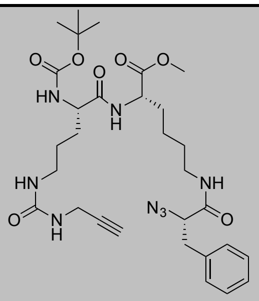	GP1<br>84%<br>83%	**HPLC** (5-100% ACN) *Rt* 10.06 mins. **LCMS** $[M+H]^+$ 628.33.
**A6**	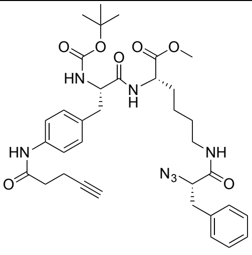	GP1<br>26%<br>86%	**HPLC** (5-100% ACN) *Rt* 11.71 mins. **LCMS** $[M+H]^+$ 676.54.
**A7**	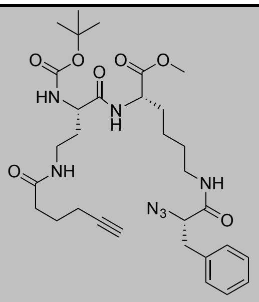	GP1<br>26%<br>90%	**HPLC** (30-100% ACN) *Rt* 8.05 mins. **LCMS** $[M+H]^+$ 628.54.
**A8**	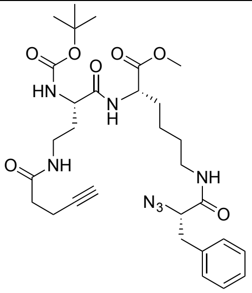	GP1<br>70%<br>89%	**HPLC** (5-100% ACN) *Rt* 7.70 mins. **LCMS** $[M+H]^+$ 614.55.
**A9**	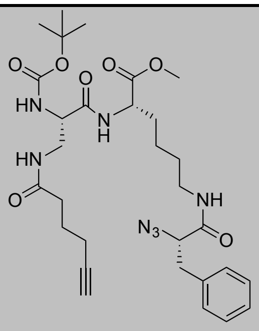	GP1<br>60%<br>91%	**HPLC** (5-100% ACN) *Rt* 11.28 mins. **LCMS** $[M+H]^+$ 613.21.

**A10**	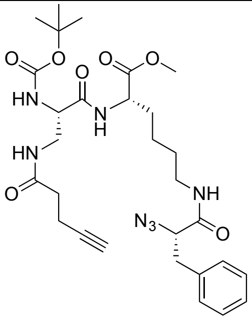	GP1<br>49%<br>86%	**HPLC** (5-100% ACN) *Rt* 11.04 mins. **LCMS** $[M+H]^+$ 599.31.
**A11**	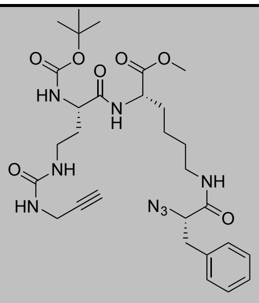	GP1<br>81%<br>94%	**HPLC** (5-100% ACN) *Rt* 10.49 mins. **LCMS** $[M+H]^+$ 614.32.
**A12**	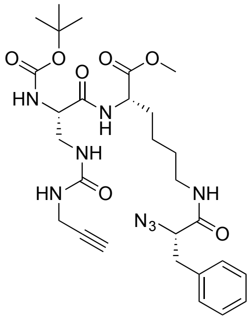	GP1<br>59%<br>93%	**δ_H /ppm** (500 MHz, $CDCl_3$): 7.39-7.24 (5H, m), 7.13 (1H, d, *J*=7.5 Hz), 6.93-6.84 (1H, m), 6.53 (1H, t, *J*=6.0 Hz), 6.11 (1H, s), 5.83-5.71 (1H, m), 4.67 (1H, dt, *J*=10.0, 4.5 Hz), 4.20 (1H, dd, *J*=8.0, 4.5 Hz), 4.16-4.22 (1H, m), 4.08 (1H, ddd, *J*=17.5, 6.0, 2.5 Hz), 4.01-3.89 (1H, m), 3.72 (3H, s), 3.71-3.54 (2H, m), 3.44-3.35 (1H, m), 3.33 (1H, dd, *J*=14.0, 4.5 Hz), 3.17-3.08 (1H, m), 3.03 (1H, dd, *J*=14.0, 8.0 Hz), 2.18 (1H, t, *J*=2.5 Hz), 1.86-1.76 (1H, m), 1.69-1.58 (1H, m), 1.57-1.49 (1H, m), 1.47 (9H, s), 1.43-1.22 (3H, m). **δ_C /ppm** (101 MHz, $CDCl_3$): 172.4, 170.8, 169.9, 159.4, 156.2, 135.8, 129.5, 128.8, 127.4, 80.9, 80.2, 70.9, 65.5, 57.2, 52.4, 51.2, 42.6, 39.5, 38.7, 32.2, 29.9, 28.9, 28.3, 22.5. **HPLC** (5-100% ACN) *Rt* 10.85 mins. **HRMS** (ESI+) *m/z* found $[M+H]^+$ 601.3081, $C_{28}H_{41}N_8O_7^+$ required 601.3098. **$[\alpha]_D^{25}$** = +4.2 (*c* 0.55, $CHCl_3$).
**A13**	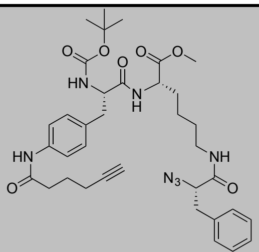	GP1<br>86%<br>87%	**HPLC** (5-100% ACN) *Rt* 11.90 mins. **LCMS** $[M+H]^+$ 690.20.
**B1**	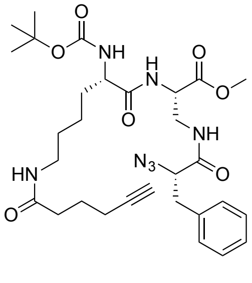	GP1<br>60%<br>96%	**HPLC** (5-100% ACN) *Rt* 10.95 mins. **LCMS** $[M+H]^+$ 614.43.

**B2**	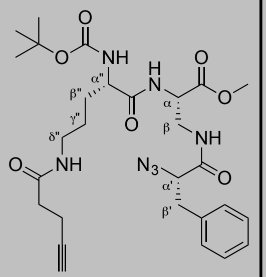	GP1<br>52%<br>94%	**R_f** = 0.33 (10% MeOH/ 90% CH_2Cl_2). **Mp** = 104-108 °C (2% MeOH/ 98% CH_2Cl_2. **δ_H /ppm** (400 MHz, $CDCl_3$): 7.39-7.19 (6H, m, 5 × ArC*H* and C_{α}-N*H*), 7.06-6.92 (1H, m, C_{β}-N*H*), 6.03-5.93 (1H, m, $C_{\delta''}$-N*H*), 5.19 (1H, d, *J*=6.6 Hz, BocN*H*), 4.57 (1H, dt, *J*=7.4, 5.1 Hz, H_{α}), 4.25-4.16 (1H, m, $H_{\alpha''}$), 4.13 (1H, dd, *J*=8.4, 4.6 Hz, $H_{\alpha'}$), 3.76 (3H, s, OCH_3), 3.70-3.62 (2H, m, H_{β}), 3.57-3.43 (1H, m, $H_{\delta''}$), 3.30 (1H, dd, *J*=14.0, 4.6 Hz, $H_{\beta'}$), 3.25-3.15 (1H, m, $\underline{H}_{\delta''}$), 2.97 (1H, dd, *J*=14.0, 8.4 Hz, $\underline{H}_{\beta'}$), 2.55-2.47 (2H, m, $COCH_2CH_2$), 2.44-2.35 (2H, m, $COCH_2CH_2$), 2.03 (1H, t, *J*=2.6 Hz, C*H*≡C), 1.87-1.75 (1H, m, $H_{\beta''}$), 1.67-1.55 (3H, m, $\underline{H}_{\beta''}$ and $H_{\gamma''}$), 1.43 (9H, s, $C(CH_3)_3$). **δ_C /ppm** (101 MHz, $CDCl_3$): 172.5 ($C_{a''}$*C*=O), 171.9 (*C*=OCH_2CH_2), 170.4 (*C*OOMe), 170.0 ($C_{a'}$*C*=O), 155.9 (Boc *C*=O), 136.4 (Ar*C*), 129.6 (Ar*C*H), 128.8 (Ar*C*H), 127.4 (Ar*C*H), 83.1 (*C*≡CH), 80.3 ($C(CH_3)_3$), 69.8 (C≡*C*H), 65.4 ($C_{\alpha'}$), 53.6 ($C_{\alpha''}$), 53.0 (OCH_3), 52.8 (C_{α}), 40.8 (C_{β}), 38.6 ($C_{\delta''}$), 38.6 ($C_{\beta'}$), 35.5 ($COCH_2CH_2$), 30.1 ($C_{\beta''}$), 28.4 ($C(CH_3)_3$), 25.7 ($C_{\gamma''}$), 15.1 ($COCH_2CH_2$). **v_{max} /cm^{-1}:** 3290 (m, alkyne C-H), 2938 (w, C-H), 2114 (m, N_3), 1744 (m, C=O), 1649 (s, C=O), 1522 (s, C=C). **HPLC** (5-100% ACN) *Rt* 10.53 mins. **HRMS** (ESI+) *m/z* found $[M+H]^+$ 586.2991, $C_{28}H_{40}N_7O_7^+$ required 586.2989 (Δ 0.3 ppm). **$[\alpha]_D^{25}$** = +47.0 (*c* 0.24, $CHCl_3$).
**B3**	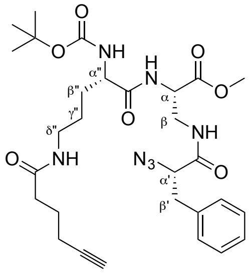	GP1<br>45%<br>98%	**R_f** = 0.17 (5% MeOH/ 95%CH_2Cl_2). **Mp** = 82-85 °C (5% MeOH/ 95% CH_2Cl_2). **δ_H /ppm** (500 MHz, $CDCl_3$): 7.37-7.18 (6H, m, 5 × ArC*H* and C_{α}-N*H*), 7.01 (1H, t, *J*=6.6 Hz, C_{β}-N*H*), 5.87-5.76 (1H, m, $C_{\delta''}$-N*H*), 5.19 (1H, d, *J*=7.2 Hz, BocN*H*), 4.57 (1H, dt, *J*=7.4, 5.1 Hz, H_{α}), 4.26-4.18 (1H, m, $H_{\alpha''}$), 4.12 (1H, dd, *J*=8.5, 4.6 Hz, $H_{\alpha'}$), 3.76 (3H, s, OCH_3), 3.71-3.63 (2H, m, H_{β}), 3.54-3.46 (1H, m, $H_{\delta''}$), 3.30 (1H, dd, *J*=14.0, 4.6 Hz, $H_{\beta'}$), 3.22-3.14 (1H, m, $\underline{H}_{\delta''}$), 2.98 (1H, dd, *J*=14.0, 8.5 Hz, $\underline{H}_{\beta'}$), 2.32 (2H, t, *J*=7.4 Hz, $COCH_2CH_2CH_2$), 2.24 (2H, td, *J*=6.8, 2.5 Hz, $COCH_2CH_2CH_2$), 1.97 (1H, t, *J*=2.5 Hz, C*H*≡C), 1.89-1.74 (3H, m, $COCH_2CH_2CH_2$ and $H_{\beta''}$), 1.67-1.51 (3H, m, $\underline{H}_{\beta''}$and $H_{\gamma''}$), 1.43 (9H, s, $C(CH_3)_3$). **δ_C /ppm** (125 MHz, $CDCl_3$): 173.2 (*C*=$OCH_2CH_2CH_2$), 172.5 ($C_{a''}$*C*=O), 170.4 (*C*OOMe), 170.0 ($C_{a'}$*C*=O), 155.9 (Boc *C*=O), 136.4 (Ar*C*), 129.6 (Ar*C*H), 128.8 (Ar*C*H), 127.4 (Ar*C*H), 83.6 (*C*≡CH), 80.3 ($C(CH_3)_3$), 69.5 (C≡*C*H), 65.4 ($C_{\alpha'}$), 53.6 ($C_{\alpha''}$), 53.0 (OCH_3), 52.7 (C_{α}), 40.8 (C_{β}), 38.6 ($C_{\beta'}$), 38.5 ($C_{\delta''}$), 35.3 ($COCH_2CH_2CH_2$), 30.1 ($C_{\beta''}$), 28.4 ($C(CH_3)_3$), 25.8 ($C_{\gamma''}$), 24.3 ($COCH_2CH_2CH_2$), 18.0 ($COCH_2CH_2CH_2$). **v_{max} /cm^{-1}:** 3294 (m, alkyne C-H), 2929 (w, C-H), 2114 (N_3), 1659 (s, C=O), 1521 (s, C=C). **HPLC** (5-100% ACN) *Rt* 10.8 mins. **HRMS** (ESI+) *m/z* found $[M+H]^+$ 600.3134, $C_{29}H_{42}N_7O_7^+$ required 600.3146 (Δ -2.0 ppm). **$[\alpha]_D^{25}$** = +50.0 (*c* 0.14, $CHCl_3$).
**B4**	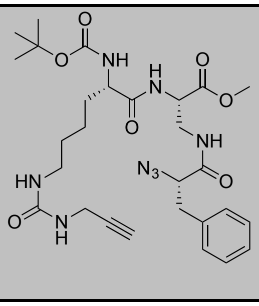	GP1<br>51%<br>99%	**HPLC** (5-100% ACN) *Rt* 10.46 mins. **LCMS** $[M+H]^+$ 601.31.

**B5**	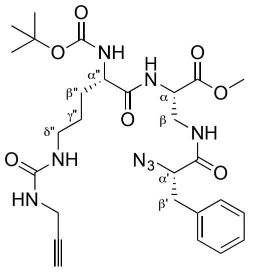	GP1<br><br>51%<br><br>95%	**R_f** = 0.44 (10% MeOH/ 90% CH_2Cl_2). **Mp** = 105-108 °C (2% MeOH/ 98% CH_2Cl_2). **δ_H /ppm** (500 MHz, $CDCl_3$): 7.47 (1H, d, *J*=7.5 Hz, C_α-N*H*), 7.40-7.21 (5H, m, 5 × ArC*H*), 7.10 (1H, app. s, C_β-N*H*), 5.28 (1H, app. s, BocN*H*), 5.03-4.93 (2H, m, $C_{\delta''}$-N*H* and N*H*$CH_2C\equiv CH$), 4.57 (1H, dt, *J*=7.5 Hz and 5.2 Hz, H_α), 4.26-4.18 (1H, m, $H_{\alpha''}$), 4.16-4.10 (1H, m, $H_{\alpha'}$), 4.01-3.88 (2H, m, NHC*H_2*C≡CH), 3.75 (3H, s, OC*H_3*), 3.73-3.59 (2H, m, H_β), 3.45-3.34 (1H, m, $H_{\delta''}$), 3.29 (1H, dd, *J* = 14.0, 4.7 Hz, $H_{\beta'}$), 3.22-3.12 (1H, m, $\underline{H}_{\delta''}$), 2.98 (1H, dd, *J* = 14.0, 8.5 Hz, $H_{\beta'}$), 2.23 (1H, t, *J* = 2.5 Hz, C*H*≡C), 1.89-1.78 (1H, m, $H_{\beta''}$), 1.65-1.51 (3H, m, $\underline{H}_{\beta''}$ and $H_{\gamma''}$), 1.42 (9H, s, C(C*H_3*)$_3$). **δ_C /ppm** (125 MHz, $CDCl_3$): 172.7 ($C_{a''}$*C*=O), 170.7 (*C*OOMe), 170.1 ($C_{a'}$*C*=O), 158.3 (NH*C*=ONH), 156.0 (Boc *C*=O), 136.4 (Ar*C*), 129.6 (ArCH), 128.8 (Ar*C*H), 127.4 (Ar*C*H), 80.8 (*C*≡CH), 80.3 (*C*(CH_3)$_3$), 71.5 (C≡*C*H), 65.4 ($C_{\alpha'}$), 53.6 ($C_{\alpha''}$), 53.0 (O*C*H_3), 52.7 (C_α), 40.8 (C_β), 39.4 ($C_{\delta''}$), 38.6 ($C_{\beta'}$), 30.3 (*C*H_2C≡CH) and $C_{\beta'}$), 28.4 (C(*C*H_3)$_3$), 26.2 ($C_{\gamma''}$). **v_{max} /cm^{-1}:** 3324 (m, alkyne C-H), 2945 (w, C-H), 2114 (m, N_3), 1738 (m, C=O), 1648 (s, C=O), 1518 (s, C=C). **HPLC** (5-100% ACN) *Rt* 10.27 mins. **HRMS** (ESI+) *m/z* found $[M+Na]^+$ 609.2756, $C_{27}H_{38}N_8O_7Na^+$ required 609.2761 (Δ -0.8 ppm). **$[\alpha]_D^{25}$** = +47.0 (*c* 0.26, $CHCl_3$).
**B6**	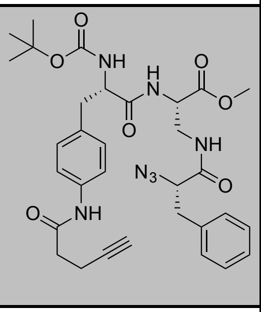	GP1<br><br>39%<br><br>89%	**HPLC** (5-100% ACN) *Rt* 11.50 mins. **LCMS** $[M+H]^+$ 634.20.
**B7**	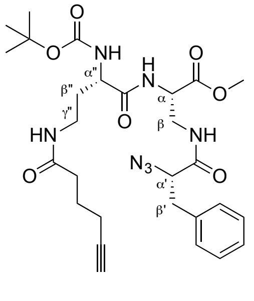	GP1<br><br>51%<br><br>95%	**R_f** = 0.45 (10% MeOH/ 90% CH_2Cl_2). **Mp** = 137-138 °C (2% MeOH/ 98% CH_2Cl_2). **δ_H /ppm** (400 MHz, $CDCl_3$): 8.26 (1H, d, *J*=6.7 Hz, C_α-N*H*), 7.38-7.20 (5H, m, 5 × ArC*H*), 7.12 (1H, t, *J*=5.0 Hz, C_β-N*H*), 6.23 (1H, app. s, $C_{\gamma''}$-N*H*), 5.53 (1H, d, *J*=6.7 Hz, BocN*H*), 4.61-4.52 (1H, m, H_α), 4.16-3.99 (2H, m, $H_{\alpha'}$ and $H_{\alpha''}$), 3.99-3.88 (1H, m, $H_{\gamma''}$), 3.89-3.78 (1H, m, H_β), 3.75 (3H, s, OC*H_3*), 3.68-3.56 (1H, m, $\underline{H}_\beta$), 3.29 (1H, dd, *J*=14.0, 4.5 Hz, $H_{\beta'}$), 3.13-2.99 (1H, m, $\underline{H}_{\gamma''}$), 2.93 (1H, dd, *J*=14.0, 8.9 Hz, $\underline{H}_{\beta'}$), 2.39 (2H, t, *J*=7.4 Hz, COC*H_2*CH_2CH_2), 2.27 (2H, td, *J*=6.8, 2.5 Hz, $COCH_2CH_2$C*H_2*), 2.00 (1H, t, *J*=2.5 Hz, C*H*≡C), 1.95-1.77 (4H, m, $COCH_2$C*H_2*CH_2 and $H_{\beta''}$), 1.42 (9H, s, C(C*H_3*)$_3$). **δ_C /ppm** (101 MHz, $CDCl_3$): 174.3 (*C*=O$CH_2CH_2CH_2$), 171.4 ($C_{a''}$*C*=O), 170.5 (*C*OOMe), 169.9 ($C_{a'}$*C*=O), 155.5 (Boc *C*=O), 136.6 (Ar*C*), 129.5 (Ar*C*H), 128.8 (Ar*C*H), 127.3 (Ar*C*H), 83.4 (*C*≡CH), 80.2 (*C*(CH_3)$_3$), 65.6 ($C_{\alpha'}$), 53.0 (C_α), 53.0 (O*C*H_3), 51.4 ($C_{\alpha''}$), 40.5 (C_β), 38.7 ($C_{\beta'}$), 36.2 ($C_{\gamma''}$), 35.3 (CO*C*$H_2CH_2CH_2$), 34.3 ($C_{\beta''}$ or $COCH_2CH_2CH_2$), 28.5 (C(*C*H_3)$_3$), 24.3 ($C_{\beta''}$ or $COCH_2CH_2CH_2$)), 18.0 ($COCH_2CH_2$*C*H_2). **v_{max} /cm^{-1}:** 3282 (m, alkyne C-H), 2950 (w, C-H), 2115 (m, N_3), 1746 (m, C=O), 1652 (s, C=O), 1523 (s, C=C). **HPLC** (5-100% ACN) *Rt* 10.82 mins. **HRMS** (ESI+) *m/z* found $[M+H]^+$ 586.2982, $C_{28}H_{40}N_7O_7^+$ required 586.2989 (Δ -1.2 ppm). **$[\alpha]_D^{25}$** = +8.0 (*c* 0.27, $CHCl_3$).

**B8**	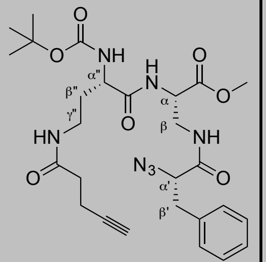	GP1<br>65%<br>99%	**R_f** = 0.47 (10% MeOH/ 90% CH_2Cl_2). **Mp** = 95-96 °C (2% MeOH/ 98% CH_2Cl_2). **δ_H /ppm** (400 MHz, $CDCl_3$): 8.06 (1H, d, *J*=7.0 Hz, C_α-N*H*), 7.40-7.19 (5H, m, 5 × ArC*H*), 7.09 (1H, t, *J*=6.1 Hz, C_β-N*H*), 6.38 (1H, s, $C_{\gamma''}$-N*H*), 5.51 (1H, d, *J*=7.0 Hz, BocN*H*), 4.60-4.53 (1H, m, H_α), 4.19-4.03 (2H, m, $H_{\alpha'}$ and $H_{\alpha''}$), 3.97-3.85 (1H, m, $H_{\gamma''}$), 3.85-3.76 (1H, m, H_β), 3.76 (3H, s, OCH_3), 3.67-3.57 (1H, m, $\underline{H}_\beta$), 3.29 (1H, dd, *J*=14.0, 4.6 Hz, $H_{\beta'}$), 3.15-3.04 (1H, m, $\underline{H}_{\gamma''}$), 2.93 (1H, dd, *J*=14.0, 8.8 Hz, $\underline{H}_{\beta'}$), 2.62-2.50 (2H, m, $COCH_2CH_2$), 2.50-2.38 (2H, m, $COCH_2CH_2$), 2.07 (1H, t, *J*=2.5 Hz, C*H*≡C), 1.95-1.78 (2H, m, $H_{\beta''}$), 1.42 (9H, s, C(CH_3)$_3$). **δ_C /ppm** (101 MHz, $CDCl_3$): 172.8 (*C*=OCH_2CH_2), 171.5 ($C_{\alpha''}$*C*=O), 170.4 (*C*OOMe), 170.0 ($C_{\alpha'}$*C*=O), 155.6 (Boc *C*=O), 136.5 (Ar*C*), 129.5 (ArCH), 128.1 (Ar*C*H), 127.3 (ArCH), 82.8 (*C*≡CH), 80.2 (*C*(CH_3)$_3$), 70.2 (C≡*C*H), 65.6 ($C_{\alpha'}$), 53.0 (C_α), 53.0 (OCH_3), 51.5 ($C_{\alpha''}$), 40.6 (C_β), 38.7 ($C_{\beta'}$), 36.2 ($C_{\gamma''}$), 35.6 (COCH_2CH_2), 34.0 ($C_{\beta''}$), 28.4 (C(CH_3)$_3$), 15.1 ($COCH_2CH_2$). **v_{max} /cm^{-1}:** 3298 (m, alkyne C-H), 2936 (w, C-H), 2112 (m, N_3), 1743 (m, C=O), 1649 (s, C=O), 1520 (s, C=C). **HPLC** (5-100% ACN) *Rt* 10.54 mins. **HRMS** (ESI+) *m/z* found $[M+Na]^+$ 594.2656, $C_{27}H_{37}N_7O_7Na^+$ required 594.2652 (Δ 0.7 ppm). **$[\alpha]_D^{25}$** = +6.0 (*c* 0.27, $CHCl_3$).
**B9**	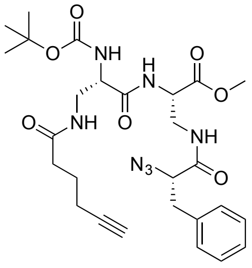	GP1<br>44%<br>88%	**δ_H /ppm** (400 MHz, $CDCl_3$): 7.38-7.22 (6H, m), 6.96 (1H, t, *J*=6.0 Hz), 6.61-6.50 (1H, m), 5.85 (1H, d, *J*=4.0 Hz), 4.51 (1H, td, *J*=6.5, 4.0 Hz), 4.18-4.09 (2H, m), 3.83-3.68 (2H, m), 3.76 (3H, s), 3.63-3.44 (2H, m), 3.29 (1H, dd, *J*=14.0, 5.0 Hz), 2.99 (1H, dd, *J*=14.0, 8.5 Hz), 2.37 (2H, t, *J*=7.0), 2.28-2.23 (2H, m), 1.97 (1H, t, *J*=2.5 Hz), 1.90-1.79 (2H, m), 1.44 (9H, s). **δ_C /ppm** (101 MHz, $CDCl_3$): 174.2, 170.9, 170.0, 169.9, 155.9, 136.1, 129.5, 128.7, 127.3, 83.4, 80.6, 69.3, 65.2, 55.8, 53.0, 52.9, 41.4, 40.6, 38.4, 34.8, 28.2, 23.9, 17.8. **HPLC** (5-100% ACN) *Rt* 10.98 mins. **HRMS** (ESI+) *m/z* found $[M+H]^+$ 572.2836, $C_{27}H_{38}N_7O_7^+$ required 572.2833. **$[\alpha]_D^{25}$** = +3.8 (*c* 0.35, $CHCl_3$).
**B10**	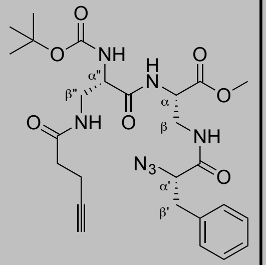	GP1<br>53%<br>93%	**R_f** = 0.33 (10% MeOH/ 90% CH_2Cl_2). **Mp** = 143-144 °C (2% MeOH/ 98% CH_2Cl_2). **δ_H /ppm** (400 MHz, $CDCl_3$): 7.41-7.19 (6H, m, 5 × ArC*H* and C_α-N*H*), 6.90-6.82 (1H, m, C_β-N*H*), 6.76 (1H, app. s, $C_{\beta''}$-N*H*,), 5.79 (1H, s, BocN*H*), 4.61-4.47 (1H, m, H_α), 4.25-4.07 (2H, m, $H_{\alpha'}$ and $H_{\alpha''}$), 3.99-3.79 (2H, m, H_β and $H_{\beta''}$), 3.77 (3H, s, OCH_3), 3.60-3.45 (2H, m, $\underline{H}_\beta$ and $\underline{H}_{\beta''}$), 3.29 (1H, dd, *J*=14.0, 4.6 Hz, $H_{\beta'}$), 2.99 (1H, dd, *J*=14.0, 8.3 Hz, $\underline{H}_{\beta'}$), 2.71-2.36 (4H, m, $COCH_2CH_2$ and $COCH_2CH_2$), 2.07-2.04 (1H, m, C*H*≡C), 1.44 (9H, s, C(CH_3)$_3$). **δ_C /ppm** (101 MHz, $CDCl_3$): 173.1 (*C*=OCH_2CH_2), 171.1 ($C_{\alpha''}$*C*=O), 170.2 ($C_{\alpha'}$*C*=O and *C*OOMe), 156.0 (Boc *C*=O), 136.2 (Ar*C*), 129.6 (Ar*C*H), 128.9 (Ar*C*H), 127.5 (Ar*C*H), 83.1 (*C*≡CH), 80.7 (*C*(CH_3)$_3$), 69.8 (C≡*C*H), 65.4 ($C_{\alpha'}$), 55.9 ($C_{\alpha''}$), 53.3 (C_α), 53.2 (OCH_3), 41.3 ($C_{\beta''}$), 40.9 (C_β), 38.6 ($C_{\beta'}$), 35.2 (COCH_2CH_2), 28.4 (C(CH_3)$_3$), 15.2 ($COCH_2CH_2$). **v_{max} /cm^{-1}:** 3301 (m, alkyne C-H), 2935 (w, C-H), 2112 (m, N_3), 1743 (m, C=O), 1652 (s, C=O), 1524 (s, C=C). **HPLC** (5-100% ACN) *Rt* 10.72 mins. **HRMS** (ESI+) *m/z* found $[M+Na]^+$ 580.2502, $C_{26}H_{35}N_7O_7Na^+$ required 580.2496 (Δ 1.0 ppm). **$[\alpha]_D^{25}$** = +3.0 (*c* 0.23, $CHCl_3$).

**B11**	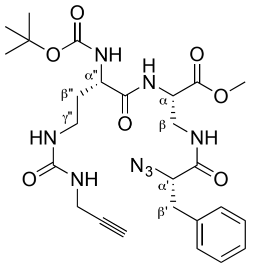	GP1<br>74%<br>86%	**R$_f$** = 0.40 (10% MeOH/ 90% CH_2Cl_2). **Mp** = 62-65 °C (2% MeOH/ 98% CH_2Cl_2). **δ$_H$ /ppm** (500 MHz, $CDCl_3$): 8.62 (1H, d, *J*=7.1 Hz, $C_α$-N*H*), 7.35-7.19 (6H, m, 5 × ArC*H* and $C_β$-N*H*), 5.67 (1H, d, *J*=7.4 Hz, BocN*H*), 5.41-5.32 (1H, m, $C_{γ''}$-N*H*), 5.18-5.07 (1H, m, N*H*CH_2C≡CH), 4.55 (1H, ddd, *J*=7.1, 5.3, 3.8 Hz, $H_α$), 4.20-4.13 (1H, m, $H_{α''}$), 4.10 (1H, dd, *J*=8.8, 4.7 Hz, $H_{α'}$), 3.98 (2H, m, NHCH_2C≡CH), 3.94-3.82 (2H, m, $H_β$ and $H_{γ''}$), 3.75 (3H, s, OCH_3), 3.60-3.53 (1H, m, $H_β$), 3.28 (1H, dd, *J*=14.0, 4.7 Hz, $H_{β'}$), 3.13-3.03 (1H, m, $H_{γ''}$), 2.94 (1H, dd, *J*=14.0, 8.8 Hz, $H_{β'}$), 2.20 (1H, t, *J*=2.5 Hz, C*H*≡C), 1.91-1.76 (2H, m, $H_{β''}$), 1.41 (9H, s, C(CH_3)$_3$). **δ$_C$ /ppm** (125 MHz, $CDCl_3$): 171.6 ($C_{a''}$*C*=O), 170.6 (*C*OOMe), 169.9 ($C_{a'}$*C*=O), 158.9 (NH*C*=ONH), 155.6 (Boc *C*=O), 136.6 (Ar*C*), 129.5 (Ar*C*H), 128.8 (Ar*C*H), 127.3 (Ar*C*H), 80.6 (*C*≡CH), 80.2 (*C*(CH_3)$_3$), 71.6 (C≡*C*H), 65.6 ($C_{α'}$), 53.1 (O*C*H_3), 52.9 ($C_α$), 51.2 ($C_{α''}$), 40.4 ($C_β$), 38.7 ($C_{β'}$), 36.6 ($C_{γ''}$), 35.2 ($C_{β''}$), 30.3 (*C*H_2C≡CH), 28.5 (C(*C*H_3)$_3$). **$ν_{max}$ /cm^{-1}:** 3310 (m, alkyne C-H), 2928 (w, C-H), 2111 (m, N_3), 1740 (m, C=O), 1652 (s, C=O), 1523 (s, C=C). **HPLC** (5-100% ACN) *Rt* 10.27 mins. **HRMS** (ESI+) *m/z* found $[M+Na]^+$ 595.2606, $C_{26}H_{37}N_8O_7Na^+$ required 595.2605 (Δ 0.2 ppm). **$[α]_D^{25}$** = +26.0 (*c* 0.24, $CHCl_3$).
**B12**	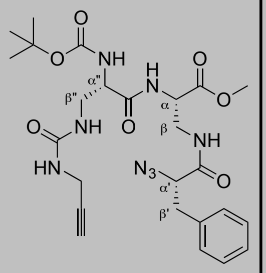	GP1<br>62%<br>95%	**R$_f$** = 0.35 (10% MeOH/ 90% CH_2Cl_2). **Mp** = 98-99 °C (2% MeOH/ 98% CH_2Cl_2). **δ$_H$ /ppm** (400 MHz, $CDCl_3$): 7.42-7.17 (6H, m, $C_α$-N*H* and 5 × ArC*H*), 6.86 (1H, app. s, $C_β$-N*H*), 5.99 (1H, app. s, BocN*H*), 5.67 (1H, dd, *J*=7.9, 4.9 Hz, $C_{β''}$-N*H*), 5.48 (1H, app. s, N*H*CH_2C≡CH), 4.61 (1H, app. s, $H_α$), 4.30-4.19 (1H, m, $H_{α'}$), 4.15-4.04 (1H, m, $H_{α''}$), 4.02 (1H, dd, *J*=5.8, 2.5 Hz, NHCH_2C≡CH), 3.99-3.95 (1H, m, NHCH_2C≡CH), 3.94-3.84 (1H, m, $H_β$), 3.83-3.68 (4H, m, $H_{β''}$ and OCH_3), 3.56-3.40 (2H, m, $H_{β''}$ and $H_β$), 3.26 (1H, dd, *J*=14.0, 5.0 Hz, $H_{β'}$), 3.04 (1H, dd, *J*=14.0 Hz and 7.8 Hz, $H_{β'}$), 2.19 (1H, t, *J*=2.5 Hz, C*H*≡C), 1.45 (9H, s, C(CH_3)$_3$). **δ$_C$ /ppm** (101 MHz, $CDCl_3$): 171.5 ($C_{a''}$*C*=O), 170.4 ($C_{a'}$*C*=O), 170.1 (*C*OOMe), 158.7 (NH*C*=ONH), 156.1 (Boc *C*=O), 135.8 (Ar*C*), 129.6 (Ar*C*H), 128.9 (Ar*C*H), 127.5 (Ar*C*H), 80.9 (*C*≡CH), 80.7 (*C*(CH_3)$_3$), 71.5 (C≡*C*H), 65.1 ($C_{α'}$), 56.8 ($C_{α''}$), 53.2 (O*C*H_3), 53.1 ($C_α$), 42.2 ($C_{β''}$), 40.8 ($C_β$), 38.5 ($C_{β'}$), 30.2 (*C*H_2C≡CH), 28.4 (C(*C*H_3)$_3$). **$ν_{max}$ /cm^{-1}:** 3323 (m, alkyne C-H), 2954 (w, C-H), 2113 (m, N_3), 1736 (m, C=O), 1649 (s, C=O), 1517 (s, C=C). **HPLC** (5-100% ACN) *Rt* 10.39 mins. **HRMS** (ESI+) *m/z* found $[M+Na]^+$ 581.2448, $C_{25}H_{34}N_8O_7Na^+$ required 581.2448 (Δ 0.0 ppm). **$[α]_D^{25}$** = +21.2 (*c* 0.31, $CHCl_3$)
**B13**	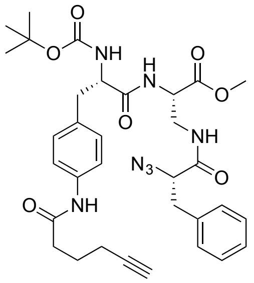	GP1<br>86%<br>87%	**HPLC** (5-100% ACN) *Rt* 11.70 mins. **LCMS** $[M+H]^+$ 648.26.

**B14**	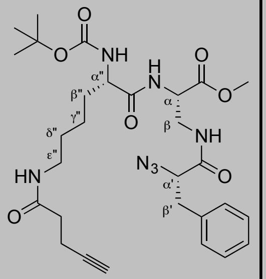	GP1<br>69%<br>98%	$\mathbf{R_f}$ = 0.47 (10% MeOH/ 90% CH_2Cl_2). **Mp** = 118-119 °C (CH_2Cl_2). $\boldsymbol{\delta_H}$ **/ppm** (400 MHz, $CDCl_3$): 7.33-6.95 (7H, m, 5 × ArC*H*, C_α-N*H*, C_β-N*H*), 6.18-6.05 (1H, m, $C_{\varepsilon''}$-N*H*), 5.29 (1H, d, *J*=6.7 Hz, BocN*H*), 4.56-4.45 (1H, m, H_α), 4.12 (1H, dd, *J*=8.5, 4.4 Hz, $H_{\alpha'}$), 4.01-3.90 (1H, m, $H_{\alpha''}$), 3.76-3.47 (5H, m, OCH_3 and H_β), 3.29-3.12 (3H, m, $H_{\varepsilon''}$ and $H_{\beta'}$), 2.89 (1H, dd, *J*=14.0 Hz and 8.5 Hz, $\underline{H}_{\beta'}$), 2.51-2.38 (2H, m, CH_2C≡C), 2.36-2.22 (2H, m, CH_2C=O), 1.96 (1H, t, *J*=2.6 Hz, C*H*≡C), 1.83-1.68 (1H, m, $H_{\beta''}$), 1.66-1.52 (1H, m, $\underline{H}_{\beta''}$), 1.52-1.41 (2H, m, $H_{\delta''}$), 1.40-1.25 (11H, m, $H_{\gamma''}$ and C(CH_3)$_3$). $\boldsymbol{\delta_C}$ **/ppm** (125 MHz, $CDCl_3$): 172.7 ($C_{\alpha''}$*C*=O), 171.5 (*C*=OCH_2CH_2), 170.4 (*C*OOMe), 170.2 ($C_{\alpha'}$*C*=O), 155.9 (Boc *C*=O), 136.3 (Ar*C*), 129.5 (Ar*C*H), 128.7 (Ar*C*H), 127.3 (Ar*C*H), 83.1 (*C*≡CH), 80.2 (*C*(CH_3)$_3$), 69.6 (C≡*C*H), 65.3 ($C_{\alpha'}$), 54.6 ($C_{\alpha''}$), 53.0 (O*C*H_3), 52.8 (C_α), 40.7 (C_β), 38.8 ($C_{\varepsilon''}$ or $C_{\beta'}$), 38.5 ($C_{\varepsilon''}$ or $C_{\beta'}$), 35.5 (CO*C*H_2CH_2), 31.6 ($C_{\beta''}$), 29.0 ($C_{\delta''}$), 28.3 (C(*C*H_3)$_3$), 22.4 ($C_{\gamma''}$), 15.0 (COCH_2*C*H_2). $\mathbf{v_{max}}$ **/cm^{-1}:** 3312 (m, alkyne C-H), 2929 (w, C-H), 2112 (m, N_3), 1744 (m, C=O), 1649 (s, C=O), 1522 (s, C=C). **HPLC** (5-100% ACN) *Rt* 10.72 mins. **HRMS** (ESI+) *m/z* found $[M+H]^+$ 600.3133, $C_{29}H_{42}N_7O_7^+$ required 600.3146 (Δ -2.2 ppm). $\mathbf{[\alpha]_D^{25}}$ = +41.0 (*c* 0.22, $CHCl_3$).
**C2**	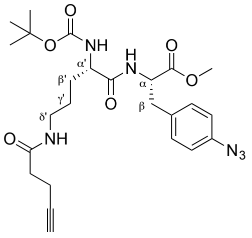	GP1<br>80%<br>98%	$\mathbf{R_f}$ = 0.45 (10% MeOH/ 90% CH_2Cl_2). **Mp** = 142-144 °C (CH_2Cl_2). $\boldsymbol{\delta_H}$ **/ppm** (400 MHz, $CDCl_3$): 7.16 (2H, d, *J*=8.4 Hz, 2 × ArC*H*), 6.96 (2H, d, *J*=8.4 Hz, 2 × ArC*H*), 6.91 (1H, d, *J*=8.0 Hz, C_α-N*H*), 5.89 (1H, app s, $C_{\delta'}$-N*H*), 5.14 (1H, d, *J*=6.9 Hz, BocN*H*), 4.82-4.73 (1H, m, H_α), 4.34-4.22 (1H, m, $H_{\alpha'}$), 3.70 (3H, s, OCH_3), 3.65-3.51 (1H, m, $H_{\delta'}$), 3.20-3.08 (2H, m, $\underline{H}_{\delta'}$ and H_β), 3.03 (1H, dd, *J*=13.9, 7.1 Hz, $\underline{H}_\beta$), 2.51-2.45 (2H, m, COCH_2C*H*$_2$), 2.38 (2H, dd, *J*=10.8, 3.8 Hz, COC*H*$_2$CH_2), 2.01 (1H, t, *J*=2.6 Hz, C*H*≡C), 1.84-1.72 (1H, m, $H_{\beta'}$), 1.68-1.48 (3H, m , $\underline{H}_{\beta'}$ and $H_{\gamma'}$) 1.44 (9H, s, C(CH_3)$_3$). $\boldsymbol{\delta_C}$ **/ppm** (125 MHz, d_6-DMSO): 172.3 ($C_{a'}$*C*=O), 172.0 (*C*OOMe), 171.9 (*C*=OCH_2CH_2), 155.8 (Boc *C*=O), 139.0 (Ar*C*), 132.9 (Ar*C*), 130.8 (Ar*C*H), 119.4 (Ar*C*H), 83.2 (*C*≡CH), 80.1 (*C*(CH_3)$_3$), 69.6 (C≡*C*H), 53.5 (C_α), 52.9 ($C_{\alpha'}$), 52.5 (O*C*H_3), 38.3 ($C_{\delta'}$), 37.4 (C_β), 35.5 (CO*C*H_2CH_2), 30.5 ($C_{\beta'}$), 28.5 (C(*C*H_3)$_3$), 25.9 ($C_{\gamma'}$), 15.0 (COCH_2*C*H_2). $\mathbf{v_{max}}$ **/cm^{-1}:** 3284 (m, alkyne C-H), 2950 (w, C-H), 2111 (m, N_3), 1739 (m, C=O), 1663 (s, C=O), 1518 (s, C=C). **HPLC** (5-100% ACN) *Rt* 10.9 mins. **HRMS** (ESI+) *m/z* found $[M+H]^+$ 515.2637, $C_{25}H_{35}N_6O_6^+$ required 515.2618 (Δ 3.7 ppm). $\mathbf{[\alpha]_D^{25}}$ = +48.4 (*c* 0.93, $CHCl_3$).

**C3**	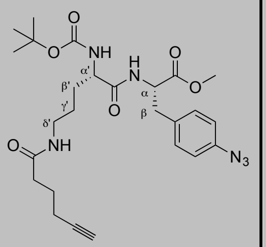	GP1<br>73%<br>85%	**R_f** = 0.47 (10% MeOH/ 90% CH_2Cl_2). **Mp** = 130-133 °C (CH_2Cl_2). **$δ_H$ /ppm** (500 MHz, $CDCl_3$): 7.17 (2H, d, *J*=8.4 Hz, 2 × Ar*CH*), 7.02-6.93 (3H, m, $C_α$-N*H* and 2 × Ar*CH*), 5.77 (1H, app s, $C_{δ'}$-N*H*), 5.15 (1H, d, *J*=7.4 Hz, BocN*H*), 4.81-4.73 (1H, m, $H_α$), 4.34-4.24 (1H, m, $H_{α'}$), 3.70 (3H, s, OCH_3), 3.62-3.52 (1H, m, $H_{δ'}$), 3.18-3.07 (2H, m, $\underline{H}_{δ'}$ and $H_β$), 3.02 (1H, dd, *J*=13.9, 7.4 Hz, $\underline{H}_β$), 2.32 (2H, t, *J*=7.4 Hz, $COCH_2CH_2CH_2$), 2.25 (2H, td, *J*=6.8, 2.6 Hz, $COCH_2CH_2CH_2$), 1.97 (1H, t, *J*=2.6 Hz, C*H*≡C), 1.86-1.72 (3H, m, $COCH_2CH_2CH_2$ and $H_{β'}$), 1.61-1.46 (3H, m, $H_{γ'}$ and $\underline{H}_{β'}$), 1.43 (9H, s, $C(CH_3)_3$). **$δ_C$ /ppm** (125 MHz, $CDCl_3$): 173.1 ($C{=}OCH_2CH_2CH_2$), 172.3 and 172.0 ($C_{a'}C$=O and *C*OOMe), 155.8 (Boc *C*=O), 139.0 (Ar*C*), 132.9 (Ar*C*), 130.8 (ArCH), 119.4 (ArCH), 83.7 (*C*≡CH), 80.1 ($C(CH_3)_3$), 69.4(C≡*C*H), 53.6 ($C_α$), 52.9 ($C_{α'}$), 52.5 (OCH_3), 38.2 ($C_{δ'}$), 37.4 ($C_β$), 35.2 ($COCH_2CH_2CH_2$), 30.6 ($C_{β'}$), 28.5($C(CH_3)_3$), 26.0 ($C_{γ'}$), 24.2 ($COCH_2CH_2CH_2$), 18.0 ($COCH_2CH_2CH_2$). **v_{max} /cm^{-1}:** 3319 (m, alkyne C-H), 2940 (w, C-H), 2112 (m, N_3), 1738 (m, C=O), 1664 (s, C=O), 1519 (s, C=C). **HPLC** (5-100% ACN) *Rt* 11.17 mins. **HRMS** (ESI+) *m/z* found $[M+H]^+$ 529.2792, $C_{26}H_{37}N_6O_6^+$ required 529.2775 (Δ 3.2 ppm). **$[α]_D^{25}$** = +39.1 (*c* 0.66, $CHCl_3$).
**C5**	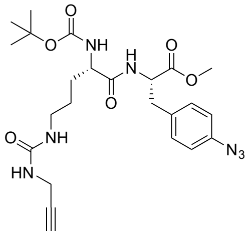	GP1<br>34%<br>89%	**HPLC** (5-100% ACN) *Rt* 10.60 mins. **LCMS** $[M+H]^+$ 516.24.
**C7**	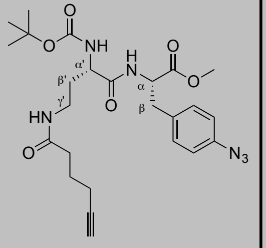	GP1<br>74%<br>92%	**R_f** = 0.37 (10% MeOH/ 90% CH_2Cl_2). **Mp** = 94-96°C (CH_2Cl_2). **$δ_H$ /ppm** (500 MHz, $CDCl_3$): 7.96 (1H, d, *J*=7.4 Hz, $C_α$-N*H*), 7.21 (2H, d, *J*=8.4 Hz, 2 × Ar*CH*), 6.98-6.92 (2H, m, 2 × Ar*CH*), 6.19 (1H, app s, $C_{γ'}$-N*H*), 5.45 (1H, d, *J*=7.0 Hz, BocN*H*), 4.74 (1H, td, *J*=8.1, 5.4 Hz, $H_α$), 4.01 (1H, dd, *J*=14.2, 7.0 Hz, $H_{α'}$), 3.94-3.82 (1H, m, $H_{γ'}$), 3.72 (3H, s, OCH_3), 3.17 (1H, dd, *J*=14.0, 5.4 Hz, $H_β$), 3.04 (1H, dd, *J*=14.0, 8.1 Hz, $\underline{H}_β$), 3.01-2.95 (1H, m, $\underline{H}_{γ'}$), 2.37 (2H, t, *J*=7.4 Hz, $COCH_2CH_2CH_2$), 2.31-2.25 (2H, m, $COCH_2CH_2CH_2$), 1.99 (1H, t, *J*=2.6 Hz, C*H*≡C), 1.92-1.85 (1H, m, $COCH_2CH_2CH_2$), 1.84-1.77 (2H, m, $H_{β'}$), 1.42 (9H, s, $C(CH_3)_3$). **$δ_C$ /ppm** (125 MHz, $CDCl_3$): 173.8 ($C{=}OCH_2CH_2CH_2$), 172.0 (*C*OOMe), 171.3 ($C_{a'}C$=O), 155.5 (Boc *C*=O), 139.0 (Ar*C*), 133.1 (Ar*C*), 130.8 (Ar*C*H), 119.3 (Ar*C*H), 83.6 (*C*≡CH), 80.1 ($C(CH_3)_3$), 69.5 (C≡*C*H), 54.1 ($C_α$), 52.6 (OCH_3), 51.3 ($C_{α'}$), 37.2 ($C_β$), 36.0 ($C_{γ'}$), 35.1 ($COCH_2CH_2CH_2$), 34.6 ($C_{β'}$), 28.5 ($C(CH_3)_3$), 24.2 ($COCH_2CH_2CH_2$), 18.0 ($COCH_2CH_2CH_2$). **v_{max} /cm^{-1}:** 3283 (m, alkyne C-H), 2942 (w, C-H), 2113 (m, N_3), 1741 (m, C=O), 1662 (s, C=O), 1523 (s, C=C). **HPLC** (5-100% ACN) *Rt* 11.2 mins. **HRMS** (ESI+) *m/z* found $[M+H]^+$ 537.2443, $C_{25}H_{34}N_6O_6Na^+$ required 537.2438 (Δ 0.9 ppm). **$[α]_D^{25}$** = -5.7 (*c* 0.74, $CHCl_3$)..

**C8**	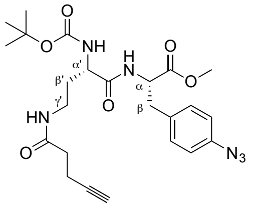	GP1<br>64%<br>94%	**R_f** = 0.45 (10% MeOH/ 90% CH_2Cl_2). **Mp** = 121-125 °C (CH_2Cl_2). **δ_H /ppm** (500 MHz, $CDCl_3$): 7.73 (1H, d, *J*=6.9 Hz, C_α-N*H*), 7.19 (2H, d, *J*=8.3 Hz, 2 × ArC*H*), 7.00-6.92 (2H, m, 2 × ArC*H*), 6.31 (1H, app s, $C_{\gamma'}$-N*H*), 5.41 (1H, d, *J*=7.1 Hz, BocN*H*), 4.78-4.72 (1H, m, H_α), 4.06 (1H, dd, *J*=14.0, 7.1 Hz, $H_{\alpha'}$), 3.95-3.82 (1H, m, $H_{\gamma'}$), 3.72 (3H, s, OC*H_3*), 3.17 (1H, dd, *J*=14.0, 5.4 Hz, H_β), 3.09-2.96 (2H, m, $\underline{H}_\beta$ and $\underline{H}_{\gamma'}$), 2.63-2.48 (2H, m, $COCH_2CH_2$), 2.44 (2H, dd, *J*=10.5, 4.4 Hz, $COCH_2CH_2$), 2.00 (1H, t, *J*=2.6 Hz, C*H*≡C), 1.87-1.78 (2H, m, $H_{\beta'}$), 1.42 (9H, s, $C(CH_3)_3$). **δ_C /ppm** (125 MHz, $CDCl_3$): 172.6 (*C*=OCH_2CH_2), 172.3 (*C*OOMe), 171.6 ($C_{a'}$*C*=O), 155.8 (Boc *C*=O), 139.3 (Ar*C*), 133.3 (Ar*C*), 131.1 (Ar*C*H), 119.6 (Ar*C*H), 83.3 (*C*≡CH), 80.4 (*C*$(CH_3)_3$), 70.0 (C≡*C*H), 54.3 (C_α), 52.9 (O*C*H_3), 51.6 ($C_{\alpha'}$), 37.4 (C_β), 36.3 ($C_{\gamma'}$), 35.8 ($COCH_2CH_2$), 34.5 ($C_{\beta'}$), 28.7 ($C(CH_3)_3$), 15.4 ($COCH_2CH_2$). **v_{max} /cm^{-1}:** 3306 (m, alkyne C-H), 2976 (w, C-H), 2112 (m, N_3), 1737 (m, C=O), 1645 (s, C=O), 1523 (s, C=C). **HPLC** (5-100% ACN) *Rt* 10.92 mins. **HRMS** (ESI+) *m/z* found $[M+H]^+$ 501.2473, $C_{24}H_{33}N_6O_6^+$ required 501.2462 (Δ 2.2 ppm). **$[\alpha]_D^{25}$** = +1.0 (*c* 0.28, $CHCl_3$).
**C10**	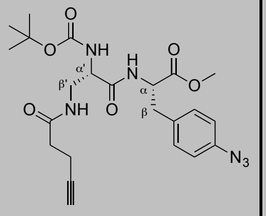	GP1<br>80%<br>89%	**R_f** = 0.44 (10% MeOH/ 90% CH_2Cl_2). **Mp** = 146-149 °C (CH_2Cl_2). **δ_H /ppm** (500 MHz, $CDCl_3$): 7.24 (1H, br s, C_α-N*H*), 7.12 (2H, d, *J*=8.4 Hz, 2 × ArC*H*), 6.95 (2H, d, *J*=8.4 Hz, 2 × ArC*H*), 6.38 (1H, app s, $C_{\beta'}$-N*H*), 5.73 (1H, app s, BocN*H*), 4.81-4.69 (1H, m, H_α), 4.21-4.12 (1H, m, $H_{\alpha'}$), 3.75-3.68 (4H, m, OC*H_3* and $H_{\beta'}$), 3.55-3.44 (1H, m, $\underline{H}_{\beta'}$), 3.15 (1H, dd, *J*=14.1, 5.5 Hz, H_β), 3.03 (1H, dd, *J*=14.1, 7.3 Hz, $\underline{H}_\beta$), 2.51 (2H, ddd, *J*=13.6, 6.9, 2.6 Hz, $COCH_2CH_2$), 2.40-2.33 (2H, m, $COCH_2CH_2$), 2.00 (1H, t, *J*=2.6 Hz, C*H*≡C), 1.43 (9H, s, $C(CH_3)_3$). **δ_C /ppm** (125 MHz, $CDCl_3$): 172.7 (*C*=OCH_2CH_2), 171.6 (*C*OOMe), 170.7 ($C_{a'}$*C*=O), 156.3 (Boc *C*=O), 139.1 (Ar*C*), 132.7 (Ar*C*), 130.7 (Ar*C*H), 119.4 (Ar*C*H), 82.9 (*C*≡CH), 80.7 (*C*$(CH_3)_3$), 69.7 (C≡*C*H), 55.4 ($C_{\alpha'}$), 53.7 (C_α), 52.7 (O*C*H_3), 41.7 ($C_{\beta'}$), 37.3 (C_β), 35.3 ($COCH_2CH_2$), 28.4 ($C(CH_3)_3$), 15.0 ($COCH_2CH_2$). **v_{max} /cm^{-1}:** 3301 (m, alkyne C-H), 2935 (w, C-H), 2110 (m, N_3), 1737 (m, C=O), 1663 (s, C=O), 1526 (s, C=C). **HPLC** (5-100% ACN) *Rt* 11.06 mins. **HRMS** (ESI+) *m/z* found $[M+H]^+$ 487.2325, $C_{23}H_{31}N_6O_6^+$ required 487.2305 (Δ 4.1 ppm). **$[\alpha]_D^{25}$** = -6.5 (*c* 1.13, $CHCl_3$).
**C11**	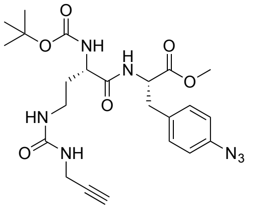	GP1<br>82%<br>85%	**HPLC** (5-100% ACN) *Rt* 10.61 mins. **LCMS** $[M+H]^+$ 502.31.

**C12**	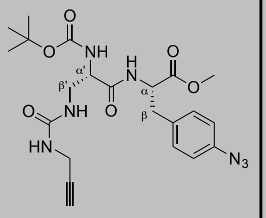	GP1<br>82%<br>94%	**R_f** = 0.47 (10% MeOH/ 90% CH_2Cl_2). **Mp** = 148-151 °C (CH_2Cl_2). **δ_H /ppm** (500 MHz, $CDCl_3$): 7.39 (1H, d, *J*=4.8 Hz, C_{α}-N*H*), 7.10 (2H, d, *J*=8.4 Hz, 2 × ArC*H*), 6.94 (2H, d, *J*=8.4 Hz, 2 × ArC*H*), 6.21 (1H, app s, BocN*H*), 5.56 (1H, s, app $C_{\beta'}$-N*H*), 5.19 (1H, app s, N*H*$CH_2C\equiv CH$), 4.78-4.66 (1H, m, H_{α}), 4.14 (1H, d, *J*=4.7 Hz, $H_{\alpha'}$), 3.98 (2H, app s, $NHCH_2C\equiv CH$), 3.71 (3H, s, OCH_3), 3.62-3.42 (2H, m, $H_{\beta'}$), 3.10 (1H, dd, *J*=14.0, 5.6 Hz, H_{β}), 3.01 (1H, dd, *J*=14.0, 7.0 Hz, H_{β}), 2.22 (1H, t, *J*=2.5 Hz, C*H*≡C), 1.42 (9H, s, $C(CH_3)_3$). **δ_C /ppm** (125 MHz, $CDCl_3$): 171.7 (*C*OOMe), 171.3 ($C_{a'}$*C*=O), 158.7 (NH*C*=ONH), 156.5 (Boc *C*=O), 139.1 (Ar*C*), 132.7 (Ar*C*), 130.7 (ArCH), 119.4 (Ar*C*H), 80.6 (*C*≡CH), 80.6 (*C*($CH_3)_3$), 71.5 (C≡*C*H), 56.2 ($C_{\alpha'}$), 53.8 (C_{α}), 52.7 (O*C*H_3), 42.8 ($C_{\beta'}$), 37.3 (C_{β}), 30. 3 (*C*$H_2C\equiv CH$), 28.4 ($C(CH_3)_3$). **v_{max} /cm^{-1}:** 3319 (m, alkyne C-H), 2981 (w, C-H), 2109 (m, N_3), 1731 (m, C=O), 1648 (s, C=O), 1527 (s, C=C). **HPLC** (5-100% ACN) *Rt* 10.66 mins. **HRMS** (ESI+) *m/z* found $[M+H]^+$ 488.2257, $C_{22}H_{30}N_7O_6^+$ required 488.2258 (Δ -0.2 ppm). **$[\alpha]_D^{25}$** = -3.1 (*c* 0.76, $CHCl_3$).
**C14**	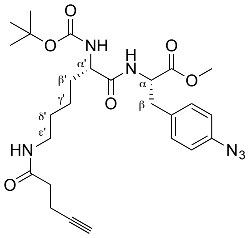	GP1<br>59%<br>81%	**R_f** = 0.31 (10% MeOH/ 90% CH_2Cl_2). **Mp** = 100-103 °C (CH_2Cl_2). **δ_H /ppm** (500 MHz, $CDCl_3$): 7.11 (2H, d, *J*=8.5 Hz, 2 × ArC*H*), 6.95 (2H, d, *J*=8.5 Hz, 2 × ArC*H*), 6.56 (1H, d, *J*=7.8 Hz, C_{α}-N*H*), 5.83 (1H, app s, $C_{\varepsilon'}$-N*H*), 5.11 (1H, d, *J*=6.8 Hz, BocN*H*), 4.87-4.76 (1H, m, H_{α}), 4.06-3.95 (1H, m, $H_{\alpha'}$), 3.73 (3H, s, OC*H*$_3$), 3.34-3.19 (2H, m, $H_{\varepsilon'}$), 3.14 (1H, dd, *J*=14.0, 5.7 Hz, H_{β}), 3.04 (1H, dd, *J*=14.0, 6.4 Hz, $\underline{H}_{\beta}$), 2.53 (2H, td, *J*=7.1, 2.6 Hz, $COCH_2CH_2$), 2.43-2.35 (2H, m, $COCH_2CH_2$), 2.02 (1H, t, *J*=2.6 Hz, C*H*≡C), 1.85-1.72 (1H, m, $H_{\beta'}$), 1.63-1.47 (3H, m, $H_{\beta'}$ and $H_{\delta'}$), 1.44 (9H, s, $C(CH_3)_3$), 1.39-1.32 (2H, m, $H_{\gamma'}$). **δ_C /ppm** (125 MHz, $CDCl_3$): 171.9 and 171.8 (*C*OOMe and $C_{a'}$*C*=O), 171.3 (*C*=OCH_2CH_2), 155.8 (Boc *C*=O), 139.1 (Ar*C*), 132.6 (Ar*C*), 130.8 (ArCH), 119.4 (Ar*C*H), 83.2 (*C*≡CH), 80.3 (*C*($CH_3)_3$), 69.6 (C≡*C*H), 54.5 ($C_{\alpha'}$), 53.3 (C_{α}), 52.6 (O*C*H_3), 38.8 ($C_{\varepsilon'}$), 37.4 (C_{β}), 35.6 (CO*C*H_2CH_2), 31.7 ($C_{\beta'}$), 29.1 ($C_{\delta'}$), 28.5 ($C(CH_3)_3$), 22.5 ($C_{\gamma'}$), 15.1 ($COCH_2CH_2$). **v_{max} /cm^{-1}:** 3312 (m, alkyne C-H), 2936 (w, C-H), 2115 (m, N_3), 1739 (m, C=O), 1644 (s, C=O), 1523 (s, C=C). **HPLC** (5-100% ACN) *Rt* 11.08 mins. **HRMS** (ESI+) *m/z* found $[M+H]^+$ 529.2783, $C_{26}H_{37}N_6O_6^+$ required 529.2775 (Δ 1.5 ppm). **$[\alpha]_D^{25}$** = +25.1 (*c* 1.08, $CHCl_3$).
**D1**	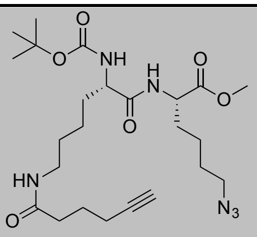	GP1<br>80%<br>87%	**HPLC** (30-100% ACN) *Rt* 7.49 mins.
**D2**	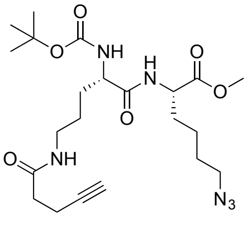	GP1<br>90%<br>80%	**HPLC** (30-100% ACN) *Rt* 6.81 mins. **LCMS** $[M+H]^+$ 481.70.

**D3**	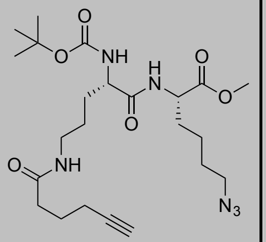	GP1<br>77%<br>92%	**δ_H /ppm** (400 MHz, $CDCl_3$): 7.08 (1H, d, *J*=8.1 Hz), 5.85 (1H, t), 5.22 (1H, d, *J*=8.2 Hz), 4.52 (1H, td, *J*=8.2, 5.0 Hz), 4.39-4.32 (1H, m), 3.71 (3H, s), 3.66-3.58 (1H, m), 3.27 (2H, t, *J*= 6.8 Hz), 3.16-3.07 (1H, m), 2.32 (2H, t, *J*=7.4 Hz), 2.24 (2H, td, *J*=6.9, 2.7 Hz), 1.96 (1H, t, *J*=2.6 Hz), 1.92-1.35 (12H, m), 1.43 (9H, s). **δ_C /ppm** (101 MHz, $CDCl_3$): 173.3, 172.8, 172.5, 156.0, 83.7, 80.0, 69.3, 52.7, 52.5, 52.7, 51.2, 38.1, 35.2, 31.7, 30.6, 28.5, 28.5, 26.0, 24.3, 22.8, 18.0. **v_{max} /cm^{-1}:** 3316, 2946, 2083, 1737, 1682, 1654, 1642, 1535, 1516, 1245, 1165. **HPLC** (30-100% ACN) *Rt* 7.23 mins. **HRMS** (ESI+) *m/z* found $[M+Na]^+$ 517.2742, $C_{23}H_{38}N_6O_6Na^+$ required 517.2751.
**D4**	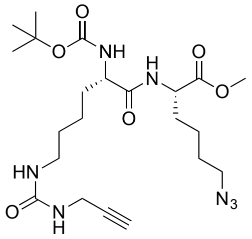	GP1<br>68%<br>89%	**HPLC** (5-100% ACN) *Rt* 10.04 mins. **LCMS** $[M+H]^+$ 496.31.
**D5**	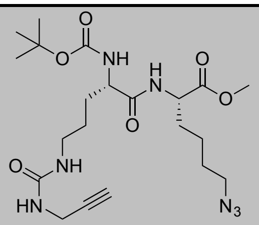	GP1<br>63%<br>83%	**HPLC** (5-100% ACN) *Rt* 10.10 mins. **LCMS** $[M+H]^+$ 482.29.
**D6**	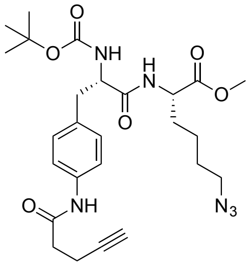	GP1<br>65%<br>79%	**HPLC** (5-100% ACN) *Rt* 11.25 mins. **LCMS** $[M-BOC+H]^+$ 429.28.
**D7**	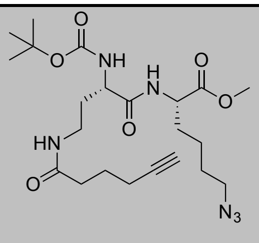	GP1<br>76%<br>91%	**HPLC** (30-100% ACN) *Rt* 7.23 mins. **LCMS** $[M+H]^+$ 481.48.
**D8**	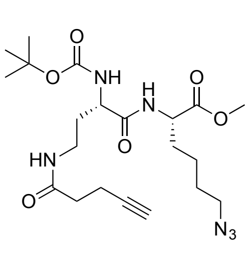	GP1<br>46%<br>97%	**δ_H /ppm** (400 MHz, $CDCl_3$): 7.87 (1H, d, *J*=7.7 Hz), 6.35 (1H, bp), 5.49 (1H, d, *J*=7.6 Hz), 4.54 (1H, td, *J*=8.0, 5.0 Hz), 4.17-4.06 (1H, m), 3.99-3.88 (1H, m), 3.74 (3H, s), 3.32 (2H, t, *J*=6.8 Hz), 3.10-2.98 (1H, m), 2.61-2.48 (2H, m), 2.52-2.39 (2H, m), 2.06 (1H, t, *J*=2.5 Hz), 1.98-1.43 (8H, m), 1.88-1.48 (8H, m), 1.43 (9H, s). **δ_C /ppm** (101 MHz, $CDCl_3$): 172.8, 172.5, 171.4, 155.5, 83.0, 80.1, 69.8, 52.5, 52.5, 51.2, 51.2, 36.2, 35.5, 34.5, 31.5, 28.5, 28.5, 22.9, 15.2. **v_{max} /cm^{-1}:** 3663, 3296, 2095, 1741, 1652, 1521, 1366, 1250, 1222, 1165, 1052. **HPLC** (30-100% ACN) *Rt* 6.78 mins. **HRMS** (ESI+) *m/z* found $[M+Na]^+$ 489.2446, $C_{21}H_{34}N_6O_6Na^+$ required 489.2438.

**D9**	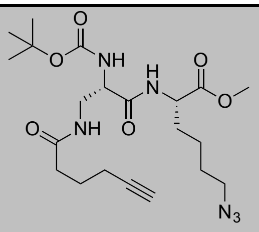	GP1<br>68%<br>96%	**HPLC** (5-100% ACN) *Rt* 10.91 mins. **LCMS** $[M+H]^+$ 467.21.
**D10**	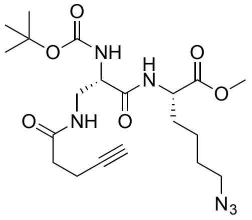	GP1<br>63%<br>99%	**HPLC** (5-100% ACN) *Rt* 10.26 mins. **LCMS** $[M+H]^+$ 453.19.
**D11**	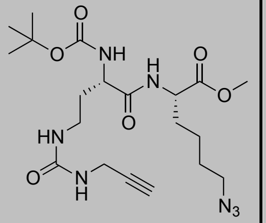	GP1<br>49%<br>99%	**δ_H /ppm** (500 MHz, $CDCl_3$): 8.44 (1H, d, *J*=7.0 Hz), 5.66 (1H, d, *J*=7.5 Hz), 5.42 (1H, dd, *J*=7.5, 5.0 Hz), 5.14 (1H, t, *J*=5.0 Hz), 4.51 (1H, dt, *J*=8.0, 5.0 Hz), 4.21-4.14 (1H, m), 4.03 (1H, ddd, *J*=17.5, 5.5, 2.5 Hz), 3.94 (1H, ddd, *J*=17.5, 5.5, 2.5 Hz), 3.89-3.80 (1H, m), 3.74 (3H, s), 3.29 (2H, t, *J*=7.0 Hz), 3.08-2.99 (1H, m), 2.22 (1H, t, *J*=2.5, 2.5 Hz), 1.95-1.77 (4H, m), 1.69-1.44 (4H, m), 1.42 (9H, s). **δ_C /ppm** (125 MHz, $CDCl_3$): 172.8, 171.5, 158.6, 155.5, 80.7, 80.0, 71.2, 52.4, 52.4, 51.1, 51.0, 36.4, 35.5, 31.1, 30.1, 28.3, 22.8. **HPLC** (5-100% ACN) *Rt* 9.61 mins. **HRMS** (ESI+) *m/z* found $[M+H]^+$ 468.2563, $C_{20}H_{34}N_7O_6^+$ required 468.2571. **$[\alpha]_D^{25}$** = -8.0 (*c* 0.52, $CHCl_3$).
**D12**	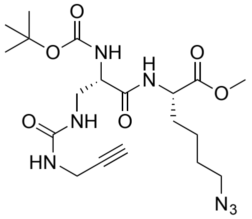	GP1<br>73%<br>83%	**HPLC** (5-100% ACN) *Rt* 10.05 mins. **LCMS** $[M+H]^+$ 454.24.
**D13**	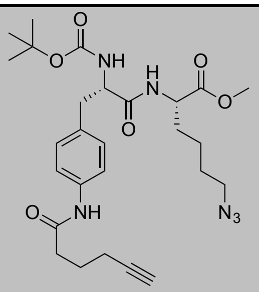	GP1<br>88%<br>97%	**HPLC** (5-100% ACN) *Rt* 11.60 mins. **LCMS** $[M+H]^+$ 543.
**D14**	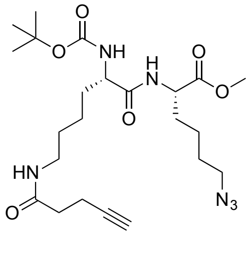	GP1<br>78%<br>79%	**HPLC** (30-100% ACN) *Rt* 7.06 mins. **LCMS** $[M+H]^+$ 495.21.

**E1**	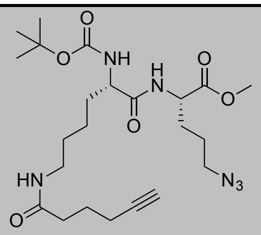	GP1<br>55%<br>88%	**HPLC** (5-100% ACN) *Rt* 10.16 mins. **LCMS** $[M+H]^+$ 495.41.
**E2**	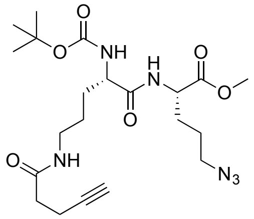	GP1<br>70%<br>88%	**HPLC** (5-100% ACN) *Rt* 9.62 mins. **LCMS** $[M+H]^+$ 467.33.
**E3**	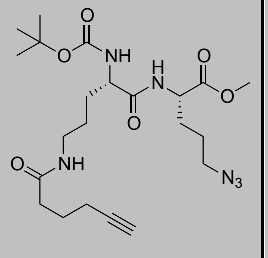	GP1<br>61%<br>91%	**δ_H /ppm** (400 MHz, $CDCl_3$): 7.15 (1H, d, *J*=8.2 Hz), 5.82 (1H, t, *J*=6.4 Hz), 5.22 (1H, d, *J*=8.2 Hz), 4.60-4.46 (1H, m), 4.41-4.29 (1H, m), 3.72 (3H, s), 3.69-3.55 (1H, m), 3.32 (2H, t, *J*=6.5 Hz), 3.15-3.06 (1H, m), 2.33 (2H, t, *J*=7.4 Hz), 2.24 (2H, td, *J*=6.9, 2.6 Hz), 1.96 (1H, t, *J*=2.6 Hz), 2.02-1.89 (1H, m), 1.88-1.48 (9H, m), 1.43 (9H, s). **δ_C /ppm** (100 MHz, $CDCl_3$): 173.3, 172.6, 172.5, 155.9, 83.6, 80.0, 69.3, 52.7, 52.5, 51.7, 50.9, 38.1, 35.2, 30.6, 29.3, 28.4, 26.0, 25.1, 24.2, 18.0. **v_{max} /cm^{-1}:** 3670, 3326, 3257, 2972, 2902, 2088, 1736, 1683, 1655, 1634, 1519, 1249, 1169, 1055. **HPLC** (5-100% ACN) *Rt* 10.00 mins. **LCMS** $[M+H]^+$ 481.39.
**E4**	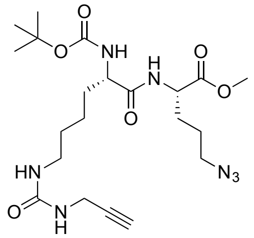	GP1<br>69%<br>89%	**HPLC** (5-100% ACN) *Rt* 9.60 mins. **LCMS** $[M+H]^+$ 482.29.
**E5**	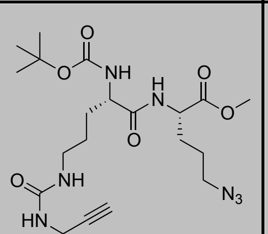	GP1<br>69%<br>92%	**δ_H /ppm** (400 MHz, $CDCl_3$): 7.42 (1H, d, *J*=8.0 Hz), 5.35 (1H, d, *J*=8.0 Hz), 5.13 (2H, m), 4.54 (1H, dt, *J*=8.0, 5.0 Hz), 4.44-4.23 (1H, m), 3.96 (2H, td, *J*=5.5, 2.5 Hz), 3.74 (3H, s), 3.53-3.39 (1H, m), 3.33 (2H, t, *J*=6.5 Hz), 3.13 (1H, dd, *J*=13.5, 5.0 Hz), 2.23 (1H, t, *J*=2.5 Hz), 2.02-1.90 (1H, m), 1.84-1.48 (7H, m), 1.43 (9H, s). **δ_C /ppm** (100 MHz, $CDCl_3$): 172.6, 158.3, 155.9, 80.8, 79.9, 71.1, 52.8, 52.5, 51.7, 50.8, 38.9, 30.3, 30.0, 29.2, 28.3, 26.2, 25.0. **HPLC** (5-100% ACN) *Rt* 9.36 mins. **HRMS** (ESI+) *m/z* found $[M+H]^+$ 468.2587, $C_{20}H_{34}N_7O_6^+$ required 468.2571. **$[\alpha]_D^{25}$** = +10.9 (*c* 0.49, $CHCl_3$).
**E6**	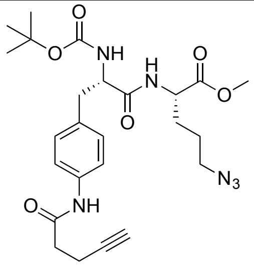	GP1<br>62%<br>82%	**HPLC** (5-100% ACN) *Rt* 10.92 mins. **LCMS** $[M+H]^+$ 515.33.

**E7**	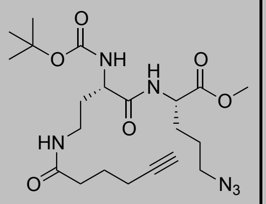	GP1<br>68%<br>92%	**δ_H /ppm** (400 MHz, $CDCl_3$): 8.17 (1H, d, *J*=7.9 Hz), 6.21 (1H, t, *J*=6.3 Hz), 5.51 (1H, d, *J*=7.5 Hz), 4.61-4.51 (1H, m), 4.11-4.02 (1H, m), 3.99-3.88 (1H, m), 3.73 (3H, s), 3.32 (2H, t, *J*=6.6 Hz), 3.05-2.95 (1H, m), 2.37 (2H, t, *J*=7.4 Hz), 2.32-2.23 (2H, m), 1.99 (1H, t, *J*=2.7 Hz), 2.02-1.61 (8H, m), 1.42 (9H, s). **δ_C /ppm** (100 MHz, $CDCl_3$): 174.0, 172.5, 171.5, 155.5, 83.6, 80.0, 69.5, 52.6, 52.1, 51.3, 50.9, 36.1, 35.2, 34.8, 29.1, 28.5, 25.2, 24.3, 18.0. **v_{max} /cm^{-1}:** 3670, 3312, 2972, 2902, 2097, 1745, 1687, 1649, 1524, 1394, 1249, 1164, 1052. **HPLC** (5-100% ACN) *Rt* 10.00 mins. **HRMS** (ESI+) *m/z* found $[M+Na]^+$ 489.2437, $C_{21}H_{34}N_6O_6Na^+$ required 489.2438.
**E8**	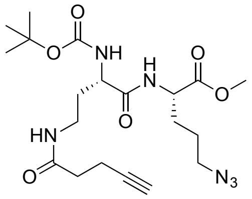	GP1<br>75%<br>77%	**HPLC** (5-100% ACN) *Rt* 9.79 mins. **LCMS** $[M+H]^+$ 453.31.
**E9**	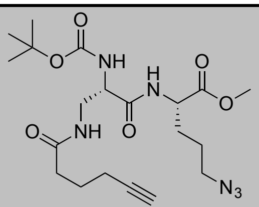	GP1<br>69%<br>93%	**HPLC** (5-100% ACN) *Rt* 10.17 mins. **LCMS** $[M+H]^+$ 453.26.
**E10**	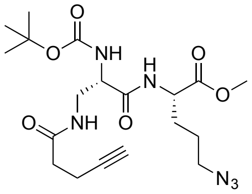	GP1<br>66%<br>94%	**HPLC** (5-100% ACN) *Rt* 9.82 mins. **LCMS** $[M+H]^+$ 439.22.
**E11**	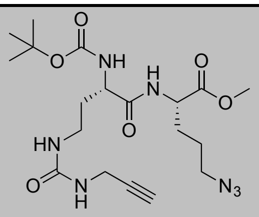	GP1<br>35%<br>87%	**HPLC** (5-100% ACN) *Rt* 9.33 mins. **LCMS** $[M+H]^+$ 454.23.
**E12**	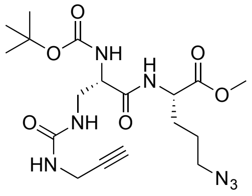	GP1<br>55%<br>96%	**HPLC** (5-100% ACN) *Rt* 9.41 mins. **LCMS** $[M+H]^+$ 440.22.

**E13**	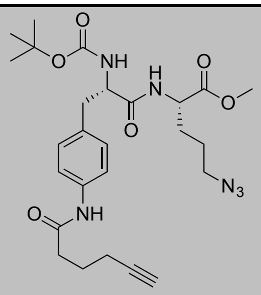	GP1<br>84%<br>90%	**HPLC** (5-100% ACN) *Rt* 11.30 mins. **LCMS** $[M\text{-}H]^+$ 527.
**E14**	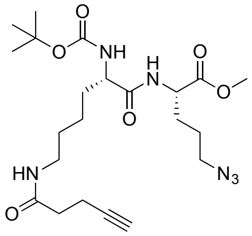	GP1<br>73%<br>84%	**HPLC** (5-100% ACN) *Rt* 10.05 mins. **LCMS** $[M+H]^+$ 481.35.
**F2**	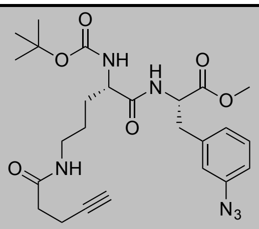	GP1<br>84%<br>83%	**HPLC** (5-100% ACN) *Rt* 10.92 mins. **LCMS** $[M+H]^+$ 515.21.
**F3**	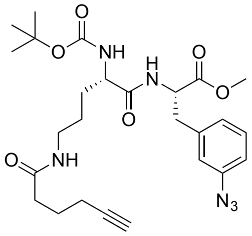	GP1<br>77%<br>77%	**HPLC** (5-100% ACN) *Rt* 11.08 mins. **LCMS** $[M+H]^+$ 529.29.
**F14**	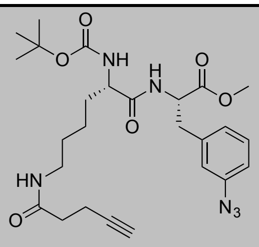	GP1<br>77%<br>77%	**HPLC** (5-100% ACN) *Rt* 11.08 mins. **LCMS** $[M+H]^+$ 529.29.

10. Preparation of B/C/C/P and B/C/C/C/P Linear Amides

	Compound	Method, Yield (%), Purity (%)	Analysis
**G1**	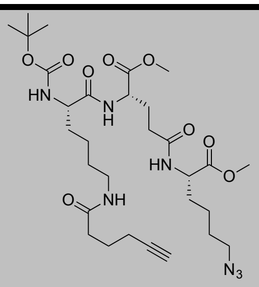	GP1<br>48%<br>97%	**HPLC** (5-100% ACN) *Rt* 10.36 mins. **LCMS** $[M+H]^+$ 652.49.
**G2**	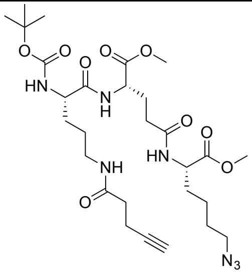	GP1<br>56%<br>86%	**HPLC** (5-100% ACN) *Rt* 9.88 mins. **LCMS** $[M+H]^+$ 624.42.
**G3**	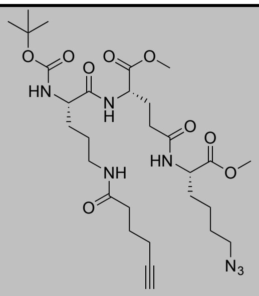	GP1<br>82%<br>81%	**HPLC** (5-100% ACN) *Rt* 10.20 mins. **LCMS** $[M+H]^+$ 638.43.
**G4**	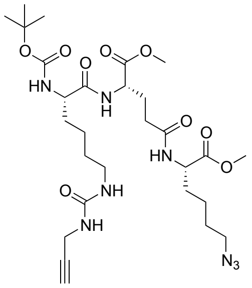	GP1<br>74%<br>75%	**δ_H /ppm** (400 MHz, $CDCl_3$): 7.50 (1H, d, *J*=7.0 Hz), 7.29 (1H, d, *J*=7.5 Hz), 5.38 (1H, d, *J*=7.5 Hz), 5.25 (1H, t, *J*=5.5 Hz), 5.20 (1H, t, *J*=5.5 Hz), 4.64-4.55 (1H, m), 4.52 (1H, td, *J*=8.0, 5.0 Hz), 4.21-4.10 (1H, m), 3.97 (2H, dd, *J*=5.5, 2.5 Hz), 3.76 (3H, s), 3.75 (3H, s), 3.27 (2H, t, *J*=6.74, 6.74 Hz), 3.26-3.17 (2H, m), 2.37-2.18 (3H, m), 2.21 (1H, t, *J*=2.5 Hz), 2.17-2.02 (1H, m), 1.88-1.43 (12H, m), 1.43 (9H, s). **δ_C /ppm** (100 MHz, $CDCl_3$): 173.7, 172.8, 172.7, 158.1, 156.0, 81.1, 80.2, 70.9, 54.1, 52.7, 52.6, 52.2, 51.5, 51.1, 39.5, 31.8, 31.5, 31.1, 29.9, 28.8, 28.3, 28.3, 26.7, 22.9, 22.1. **ν_{max} /cm^{-1}:** 3294, 2951, 2097, 1759, 1651, 1537, 1246, 1206, 1165. **HPLC** (5-100% ACN) *Rt* 9.86 mins. **HRMS** (ESI+) *m/z* found $[M+Na]^+$ 661.3279, $C_{14}H_{23}N_3O_5Na^+$ required 661.3285. **$[\alpha]_D^{25}$** = +6.7 (*c* 0.52, $CHCl_3$).

**G5**	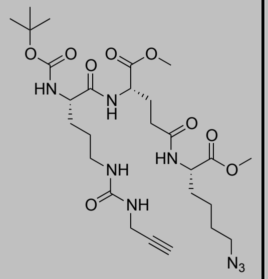	GP1<br>67%<br>78%	**δ_H /ppm** (400 MHz, $CDCl_3$): 7.61 (1H, s), 7.42 (1H, d, *J*=8.4Hz), 7.42 (1H, d, *J*=8.4Hz), 5.43-5.16 (2H, m), 4.66-4.48 (1H, m), 4.51-4.38 (1H, m), 4.27-4.17 (1H, m), 4.16-4.06 (1H, m), 4.00-3.86 (1H, m), 3.76 (3H, s), 3.72 (3H, s), 3.34-3.21 (4H, m), 2.35-2.03 (5H, m), 1.99-1.52 (10H, m), 1.46 (9H, s). **δ_C /ppm** (100 MHz, $CDCl_3$): 174.1, 173.6, 172.9, 172.4, 158.7, 156.2, 81.5, 80.2, 71.7, 54.2, 52.8, 52.7, 52.6, 51.6, 51.2, 39.4, 32.0, 31.3, 30.2, 28.5, 27.5, 26.2, 23.1. **v_{max} /cm^{-1}:** 3676, 3297, 2988, 2902, 2094, 1735, 1662, 1646, 1541, 1519, 1394, 1250, 1167, 1056. **HPLC** (5-100% ACN) *Rt* 9.67 mins. **HRMS** (ESI+) *m/z* found $[M+H]^+$ 625.3296, $C_{27}H_{45}N_8O_9^+$ required 625.3310.
**G6**	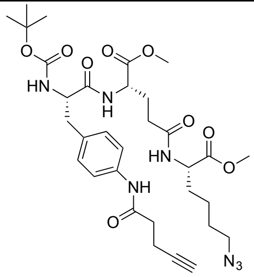	GP1<br>40%<br>97%	**HPLC** (5-100% ACN) *Rt* 10.86 mins. **LCMS** $[M+H]^+$ 672.41.
**G7**	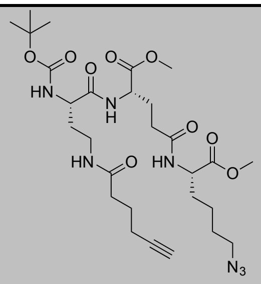	GP1<br>76%<br>78%	**HPLC** (5-100% ACN) *Rt* 10.18 mins. **LCMS** $[M+H]^+$ 624.41.
**G8**	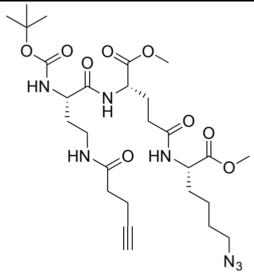	GP1<br>82%<br>87%	**HPLC** (5-100% ACN) *Rt* 9.87 mins. **LCMS** $[M+H]^+$ 610.39.
**G9**	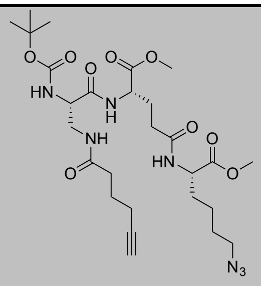	GP1<br>42%<br>97%	**HPLC** (5-100% ACN) *Rt* 10.27 mins. **LCMS** $[M+H]^+$ 610.38.

**G10**	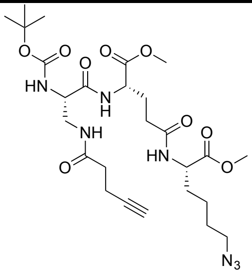	GP1<br>42%<br>97%	**HPLC** (5-100% ACN) *Rt* 9.98 mins. **LCMS** $[M+H]^+$ 596.37.
**G11**	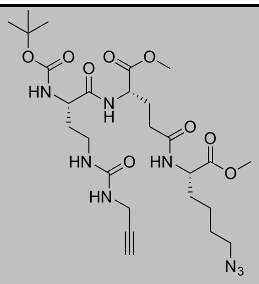	GP1<br>27%<br>99%	**HPLC** (5-100% ACN) *Rt* 9.54 mins. **LCMS** $[M+H]^+$ 611.36.
**G12**	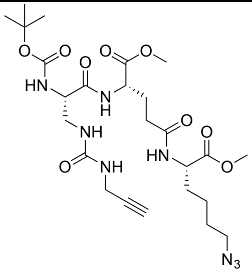	GP1<br>66%<br>71%	**HPLC** (5-100% ACN) *Rt* 9.86 mins. **LCMS** $[M+H]^+$ 597.41.
**G13**	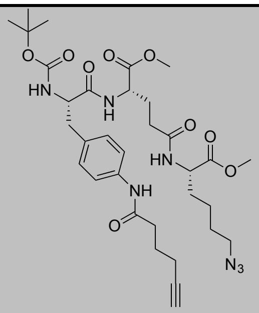	GP1<br>77%<br>84%	**HPLC** (5-100% ACN) *Rt* 11.20 mins. **LCMS** $[M+H]^+$ 686.29.
**G14**	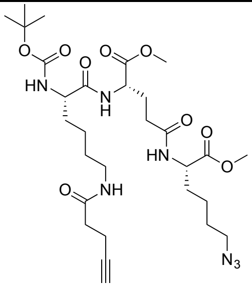	GP1<br>88%<br>80%	**HPLC** (5-100% ACN) *Rt* 10.18 mins. **LCMS** $[M+H]^+$ 638.43.

**H1**	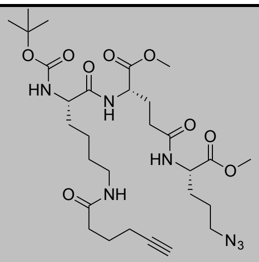	GP1<br>74%<br>92%	**HPLC** (5-100% ACN) *Rt* 9.93 mins. **LCMS** $[M+H]^+$ 638.55.
**H2**	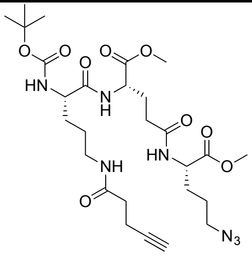	GP1<br>74%<br>90%	**HPLC** (5-100% ACN) *Rt* 9.45 mins. **LCMS** $[M+H]^+$ 610.39.
**H3**	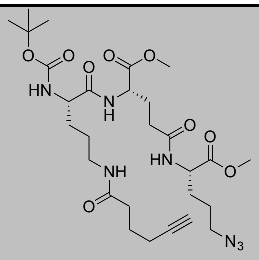	GP1<br>99%<br>82%	**HPLC** (5-100% ACN) *Rt* 9.74 mins. **LCMS** $[M+H]^+$ 624.44.
**H4**	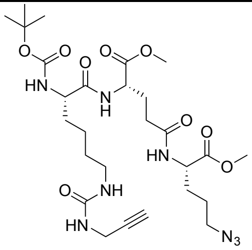	GP1<br>88%<br>84%	**HPLC** (5-100% ACN) *Rt* 9.40 mins. **LCMS** $[M+H]^+$ 625.45.
**H5**	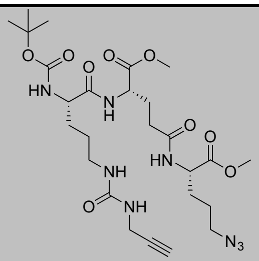	GP1<br>57%<br>87%	**HPLC** (5-100% ACN) *Rt* 9.18 mins. **LCMS** $[M+H]^+$ 611.36.
**H6**	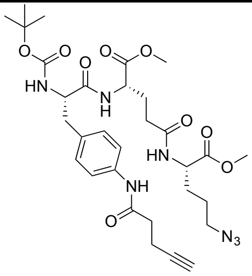	GP1<br>55%<br>77%	**HPLC** (5-100% ACN) *Rt* 10.55 mins. **LCMS** $[M+H]^+$ 658.46.

**H7**	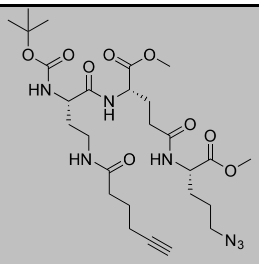	GP1<br>69%<br>90%	**HPLC** (5-100% ACN) *Rt* 9.72 mins. **LCMS** $[M+H]^+$ 610.34.
**H8**	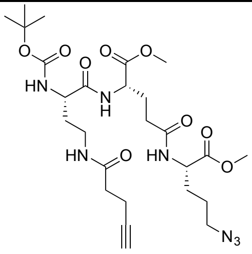	GP1<br>82%<br>91%	**HPLC** (5-100% ACN) *Rt* 9.40 mins. **LCMS** $[M+H]^+$ 596.34.
**H9**	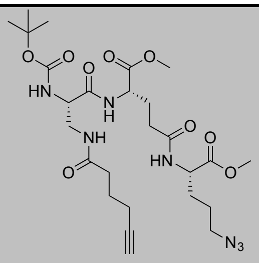	GP1<br>83%<br>71%	**HPLC** (5-100% ACN) *Rt* 9.88 mins. **LCMS** $[M+H]^+$ 596.36.
**H10**	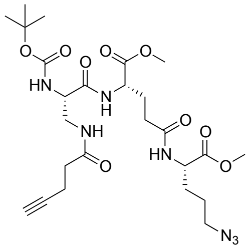	GP1<br>53%<br>84%	**δ_H /ppm** (400 MHz, $CDCl_3$): 7.57 (1H, d, *J*=7.0 Hz), 6.96 (1H, d, *J*=7.5 Hz), 6.76 (1H, t, *J*=6.0 Hz), 5.92 (1H, d, *J*=6.5 Hz), 4.62-4.51 (2H, m), 4.25 (1H, m), 3.76 (3H, s), 3.74 (3H, s), 3.72-3.53 (2H, m), 3.31 (2H, dt, *J*=6.5, 1.0 Hz), 2.58-2.47 (2H, m), 2.47-2.40 (2H, m), 2.35-2.21 (3H, m), 2.04 (1H, t, *J*=2.5 Hz), 2.02-1.89 (3H, m), 1.82-1.55 (3H, m), 1.44 (9H, s). **δ_C /ppm** (100 MHz, $CDCl_3$): 173.0, 172.6, 172.2, 172.1, 171.2, 156.0, 82.9, 80.5, 69.4, 55.0, 52.7, 52.7, 51.9, 51.8, 50.7, 41.4, 35.0, 32.1, 29.2, 28.3, 27.3, 25.0, 14.8. **HPLC** (5-100% ACN) *Rt* 9.56 mins. **HRMS** (ESI+) *m/z* found $[M+H]^+$ 582.2873, $C_{25}H_{40}N_7O_9^+$ required 582.2888. $[\alpha]_D^{25}$ = -14.6 (*c* 0.50, $CHCl_3$).
**H11**	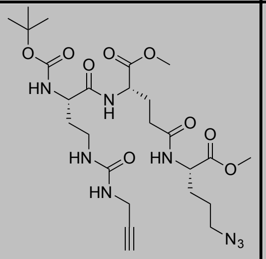	GP1<br>41%<br>89%	**HPLC** (5-100% ACN) *Rt* 9.13 mins. **LCMS** $[M+H]^+$ 597.33.

**H12**	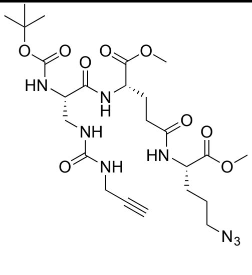	GP1<br>66%<br>76%	**HPLC** (5-100% ACN) *Rt* 9.24 mins. **LCMS** $[M+H]^+$ 583.30<br>.
**H14**	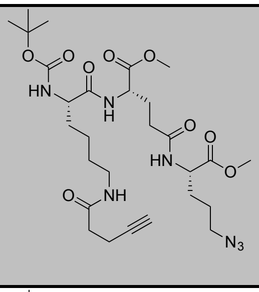	GP1<br>67%<br>91%	**HPLC** (5-100% ACN) *Rt* 9.65 mins. **LCMS** $[M+H]^+$ 624.44.
**J13**	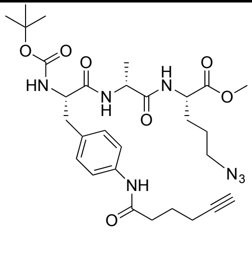	GP1<br>73%<br>88%	**HPLC** (5-100% ACN) *Rt* 10.71mins. **LCMS** $[M+H]^+$ 600.34.
**K3**	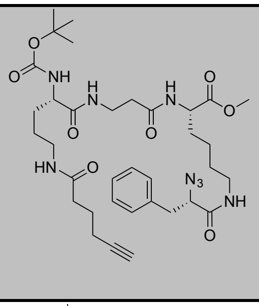	GP1<br>62%<br>80%	**HPLC** (5-100% ACN) *Rt* 10.35 mins. **LCMS** $[M+H]^+$ 713.52.
**K8**	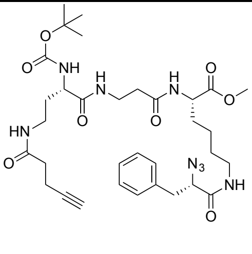	GP1<br>67%<br>82%	**HPLC** (5-100% ACN) *Rt* 10.09 mins. **LCMS** $[M+H]^+$ 685.47.
**L7**	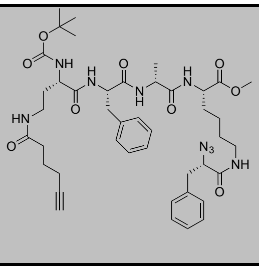	GP1<br>79%<br>78%	**HPLC** (5-100% ACN) *Rt* 11.60 mins. **LCMS** $[M+H]^+$ 846.67.

**L9**	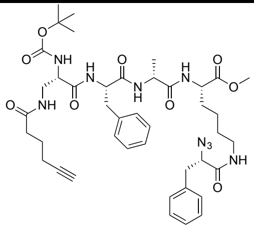	GP1<br>62%<br>79%	**HPLC** (5-100% ACN) *Rt* 832.57 mins. **LCMS** $[M+H]^+$ 747.50.
**M13**	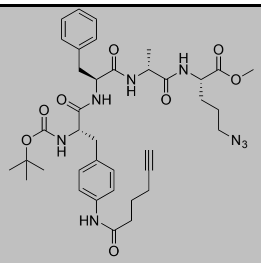	GP1<br>61%<br>82%	**HPLC** (5-100% ACN) *Rt* 11.67 mins. **LCMS** $[M+H]^+$ 747.50.
**N8**	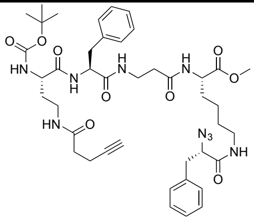	GP1<br>72%<br>77%	**HPLC** (5-100% ACN) *Rt* 11.05 mins. **LCMS** $[M+H]^+$ 832.64.

11. Preparation of B/C/P CuAAC Macrocycles

	Compound	Method, Yield (%), Purity (%)	Analysis
**A1 w**	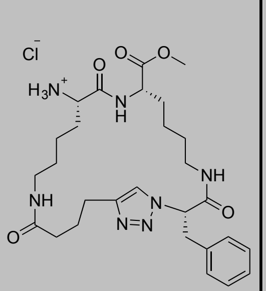	GP2 & GP4<br>54%<br>97%	**δ$_H$ /ppm** (500 MHz, *d*$_6$-DMSO): 8.75 (1H, d, *J*=6.6 Hz,), 8.43 (1H, t, *J*=5.6 Hz), 8.17 (3H, d, *J*=5.4 Hz), 7.98 (1H, s), 7.67 (1H, t, *J*=5.9 Hz), 7.26-7.11 (5H, m), 5.45 (1H, dd, *J*=8.6, 7.0 Hz), 4.17-4.06 (1H, m), 3.87-3.79 (1H, m), 3.58 (3H, s), 3.42-3.33 (2H, m), 3.29-3.19 (1H, m), 3.17-3.06 (1H, m), 3.06 -2.97 (1H, m), 2.90- 2.80 (1H, m), 2.56 (2H, td, *J*=7.3, 2.8 Hz), 2.07 (2H, td, *J*=7.4, 2.4 Hz), 1.87-1.74 (1H, m), 1.74 -1.47 (4H, m), 1.47-1.02 (8H, m). **δ$_C$ /ppm** (125 MHz, *d*$_6$-DMSO): 171.9, 171.8, 168.9, 167.2, 146.1, 136.7, 129.0, 128.4, 126.7, 121.7, 64.0, 52.5, 52.0, 38.2, 37.4, 36.8, 35.0, 30.8, 29.7, 28.5, 25.3, 24.5, 23.3, 22.5, 21.7, 21.1. **v$_{max}$ /cm^{-1}:** 3228, 3060, 2929, 1736, 1670, 1551, 1440, 1212, 742. **HPLC** (5-80% ACN) *Rt* 8.02 mins. **HRMS** (ESI+) *m/z* found [M+H]$^+$ 556.3237, $C_{28}H_{42}N_7O_5^+$ required 556.3242.
**A2 w**	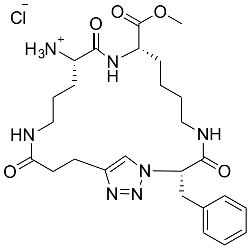	GP2 & GP4<br>88%<br>78%	**HPLC** (0-60% ACN) *Rt* 9.21 mins. **LCMS** [M+H]$^+$ 528.26.
**A3 w**	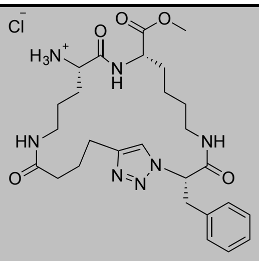	GP2 & GP4<br>99%<br>81%	**HPLC** (5-100% ACN) *Rt* 7.13 mins. **LCMS** [M+H]$^+$ 542.28.
**A4 w**	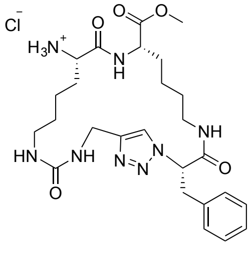	GP2 & GP4<br>99%<br>71%	**HPLC** (5-100% ACN) *Rt* 7.08 mins. **LCMS** [M+H]$^+$ 543.25.

**A5 w**	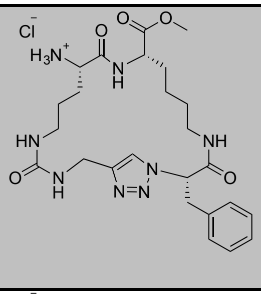	GP2 & GP4<br>87%<br>75%	**HPLC** (5-100% ACN) *Rt* 6.91 mins. **LCMS** $[M+H]^+$ 529.23.
**A7 w**	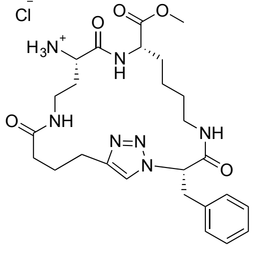	GP2 & GP4<br>88%<br>51%	**HPLC** (5-100% ACN) *Rt* 7.38 mins. **LCMS** $[M+H]^+$ 528.18.
**A8 w**	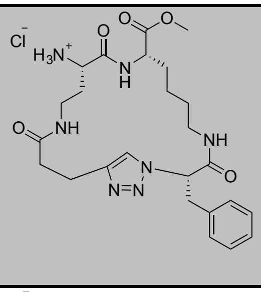	GP2 & GP4<br>91%<br>70%	**HPLC** (5-100% ACN) *Rt* 7.26 mins. **LCMS** $[M+H]^+$ 514.23.
**A9 w**	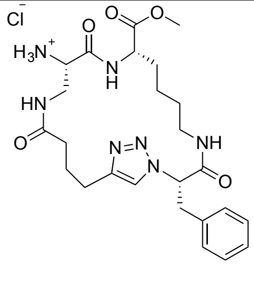	GP2 & GP4<br>99%<br>88%	**HPLC** (5-100% ACN) *Rt* 7.26 mins. **LCMS** $[M+H]^+$ 514.16.
**A10 w**	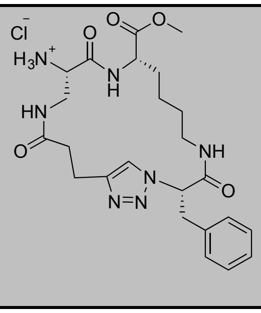	GP2 & GP4<br>99%<br>68%	**HPLC** (5-100% ACN) *Rt* 7.18 mins. **LCMS** $[M+H]^+$ 500.13.
**A11 w**	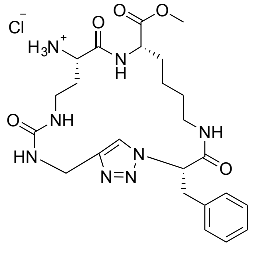	GP2 & GP4<br>80%<br>76%	**HPLC** (5-100% ACN) *Rt* 7.19 mins. **LCMS** $[M+H]^+$ 515.21.

**A12 w**	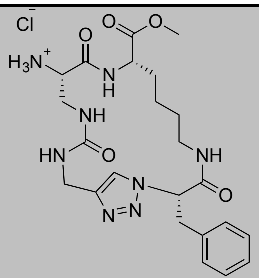	GP2 & GP4<br>99%<br>86%	**HPLC** (5-100% ACN) *Rt* 6.88 mins. **LCMS** $[M+H]^+$ 501.11.
**A13 w**	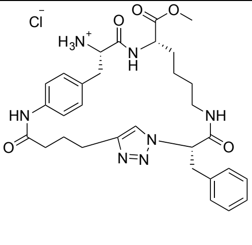	GP2 & GP5<br>11%<br>79%	**HPLC** (5-100% ACN) *Rt* 7.59 mins. **LCMS** $[M+H]^+$ 590.21.
**B1 w**	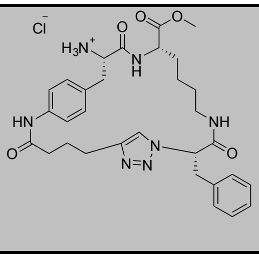	GP2 & GP4<br>61%<br>70%	**HPLC** (5-100% ACN) *Rt* 6.77 mins. **LCMS** $[M+H]^+$ 514.31.
**B2 w**	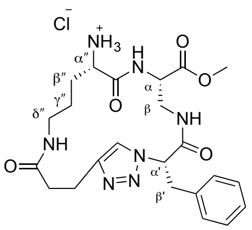	GP2 & GP4<br>45%<br>74%	**Mp** = 215-217 °C (CH_2Cl_2). **δ_H /ppm** (500 MHz, d_6-DMSO): 9.10 (1H, d, *J*=6.5 Hz, C_α-N*H*), 8.94 (1H, dd, *J*=8.5, 3.9 Hz, C_β-N*H*), 8.16 (3H , d, *J*=5.5 Hz, NH_3^+) 8.06 (1H, t, *J*=6.0 Hz, $C_{\delta''}$-N*H*),7.69 (1H, s, triazole C*H*), 7.27-7.09 (5H, m, 5 × ArC*H*), 5.45 (1H, dd, *J*=8.4, 7.1 Hz, $H_{\alpha'}$), 4.08 (1H, dt, *J*=6.5, 4.5 Hz, H_α), 3.95 (1H, ddd, *J*=13.7, 8.5, 4.5 Hz, H_β), 3.89-3.80 (1H, m, $H_{\alpha''}$), 3.43-3.36 (2H, m, $H_{\beta'}$), 3.33 (3H, s, OCH_3), 3.16-3.00 (2H, m, $\underline{H}_\beta$ and $H_{\delta''}$), 2.97-2.75 (3H, m, $\underline{H}_{\delta''}$ and $COCH_2CH_2$), 2.38 (2H, t, *J*=6.2 Hz, $COCH_2CH_2$), 1.67-1.39 (4H, m, $H_{\beta''}$ and $H_{\gamma''}$). **δ_C /ppm** (125 MHz, d_6-DMSO): 171.0 (*C*=OCH_2CH_2), 169.7 (*C*OOMe), 168.7 ($C_{\alpha''}$*C*=O), 167.7 ($C_{\alpha'}$*C*=O), 145.8 (triazole C), 136.4 (Ar*C*), 128.9 (Ar*C*H), 128.3 (Ar*C*H), 126.7 (Ar*C*H), 121.4 (triazole *C*H), 64.0 ($C_{\alpha'}$), 53.2 (C_α), 51.8 (OCH_3), 51.6 ($C_{\alpha''}$), 38.8 (C_β), 38.3 ($C_{\delta''}$), 37.0 ($C_{\beta'}$), 33.9 ($COCH_2CH_2$), 28.7 ($C_{\beta''}$), 24.3 ($C_{\gamma''}$), 21.1 ($COCH_2CH_2$). **v_{max} /cm^{-1}:** 3224 (NH str), 2924 (CH str), 1737 (C=O str), 1683 (C=O str), 1543, 1437, 1212, 1148. **HPLC** (5-100% ACN) *Rt* 8.41 mins. **HRMS** (ESI+) *m/z* found $[M+H]^+$ 486.2458, $C_{23}H_{32}N_7O_5^+$ required 486.2459. **$[\alpha]_D^{25}$** = -2.5 (*c* 0.58, MeOH).

**B3 w**	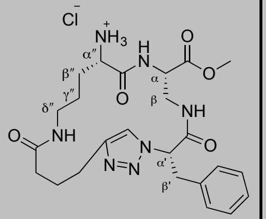	GP2 & GP4<br>99%<br>91%	**δ_H /ppm** (500 MHz, *d*$_6$-DMSO): 9.42 (1H, d, *J*=7.5 Hz, C_{α}-N*H*), 8.80 (1H, dd, *J*=9.4 Hz and 3.0 Hz, C_{β}-N*H*), 8.22 (3H, d, *J*=4.5 Hz, NH_3^+), 8.17 (1H, s, triazole C*H*), 7.89 (1H, t, *J*=5.9 Hz, $C_{\delta''}$-N*H*), 7.28-7.09 (5H, m, 5 × ArC*H*), 5.34-5.28 (1H, m, $H_{\alpha'}$), 4.34 (1H, dt, *J*=7.5, 3.4 Hz, H_{α}), 4.15 (1H, ddd, *J*=13.3, 9.4, 3.4 Hz, H_{β}), 4.00-3.92 (1H, m, $H_{\alpha''}$), 3.57 (3H, s, OC*H*$_3$), 3.54-3.43 (2H, m, $H_{\beta'}$), 3.26-3.18 (1H, m, $H_{\delta''}$), 3.04-2.98 (1H, m, $\underline{H}_{\beta}$), 2.98-2.92 (1H, m, $\underline{H}_{\delta''}$), 2.58 (2H, t, *J*=6.7 Hz, $COCH_2CH_2C\mathit{H_2}$), 2.19-2.11 (1H, m, $CO\mathit{CH_2}CH_2CH_2$), 2.08-2.02 (1H, m, $COC\underline{H_2}CH_2CH_2$), 1.89-1.76 (3H, m, $COCH_2C\mathit{H_2}CH_2$ and $H_{\beta''}$), 1.74-1.64 (1H, m, $\underline{H}_{\beta''}$), 1.59-1.52 (2H, m, $H_{\gamma''}$). **δ_C /ppm** (125 MHz, *d*$_6$-DMSO): 171.9 (*C*=OCH$_2$CH$_2$), 169.8 (*C*OOMe), 169.1 ($C_{\alpha''}$*C*=O), 166.9 ($C_{\alpha'}$*C*=O), 145.6 (triazole C), 136.7 (Ar*C*), 129.1 (ArCH), 128.3 (Ar*C*H), 126.6 (Ar*C*H), 123.1 (triazole *C*H), 64.1 ($C_{\alpha'}$), 52.3 (C_{α}), 51.2 (O*C*H$_3$), 51.1 ($C_{\alpha''}$), 36.7 ($C_{\delta''}$), 35.4 ($C_{\beta'}$), 34.2 ($CO\mathit{C}H_2CH_2CH_2$), 27.9 ($C_{\beta''}$), 25.1 ($COCH_2CH_2CH_2$), 23.7 ($COCH_2CH_2CH_2$), 23.5 ($C_{\gamma''}$). **v_{max} /cm^{-1}:** 3231 (NH str), 2935 (CH str), 1739 (C=O str), 1684 (C=O str), 1640, 1548, 1440, 1215, 1146, 1054. **HPLC** (5-100% ACN) *Rt* 8.76 mins. **HRMS** (ESI+) *m/z* found $[M+H]^+$ 500.2632, $C_{24}H_{34}N_7O_5^+$ required 500.2621. **$[\alpha]_D^{25}$** = +3.0 (*c* 0.075, MeOH).
**B4 w**	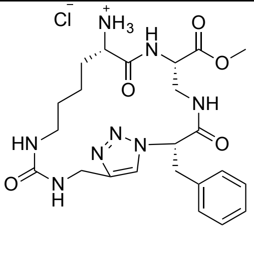	GP2 & GP4<br>70%<br>78%	**HPLC** (5-25% ACN) *Rt* 13.56 mins. **LCMS** $[M+H]^+$ 501.26.
**B5 w**	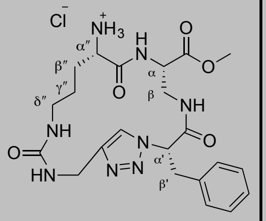	GP2 & GP4<br>96%<br>89%	**Mp** = 228-230 °C (CH_2Cl_2). **δ_H /ppm** (500 MHz, *d*$_6$-DMSO): 9.24 (1H, d, *J*=7.0 Hz, C_{α}-N*H*), 9.19 (1H, dd, *J*=8.9, 3.3 Hz, C_{β}-N*H*), 8.14 (3H, d, *J*=4.3 Hz, NH_3^+), 7.90 (1H, s, triazole C*H*), 7.26-7.13 (5H, m, 5 × ArC*H*), 6.38 (1H, s, N*H*CH$_2$), 6.17 (1H, s, $C_{\delta''}$-N*H*), 5.49-5.39 (1H, m, $H_{\alpha'}$), 4.37 (1H, d, *J*=16.1 Hz, NHC*H*$_2$), 4.13 (1H, dt, *J*=7.0, 3.3 Hz, H_{α}), 4.07 (1H, d, *J*=16.1 Hz, $NHC\underline{H_2}$), 4.02-3.95 (1H, m, H_{β}), 3.95-3.88 (1H, m, $H_{\alpha''}$), 3.52 (1H, dd, *J*=14.0, 8.4 Hz, $H_{\beta'}$), 3.45 (1H, dd, *J*=14.0, 6.9 Hz, $\underline{H}_{\beta'}$), 3.16-3.07 (4H, m, OC*H*$_3$ and $H_{\delta''}$), 3.07-3.02 (1H, m, $\underline{H}_{\beta}$), 2.92-2.82 (1H, m, $\underline{H}_{\delta''}$), 1.80-1.66 (2H, m, $H_{\beta''}$), 1.58-1.33 (2H, m, $H_{\gamma''}$). **δ_C /ppm** (125 MHz, *d*$_6$-DMSO): 169.9 (*C*OOMe), 169.1 ($C_{\alpha''}$*C*=O), 167.2 ($C_{\alpha'}$*C*=O), 158.0 (NH*C*=ONH), 147.5 (triazole C), 136.6 (Ar*C*), 128.9 (Ar*C*H), 128.3 (Ar*C*H), 126.7 (Ar*C*H), 122.3 (triazole *C*H), 64.0 ($C_{\alpha'}$), 52.9 (C_{α}), 51.9 ($C_{\alpha''}$), 51.6 (O*C*H$_3$), 38.9 (C_{β}), 38.8 ($C_{\delta''}$), 35.7 ($C_{\beta'}$), 35.3 (NH*C*H$_2$), 28.7 ($C_{\beta''}$), 25.6 ($C_{\gamma''}$). **v_{max} /cm^{-1}:** 3233 (NH str), 2924 (CH str), 1737 (C=O str), 1678 (C=O str), 1542, 1437, 1260, 1145. **HPLC** (5-100% ACN) *Rt* 8.45 mins. **HRMS** (ESI+) *m/z* found $[M+H]^+$ 487.2417, $C_{22}H_{31}N_8O_5^+$ required 487.2417. **$[\alpha]_D^{25}$** = -13.3 (*c* 0.68, MeOH).

**B7 w**	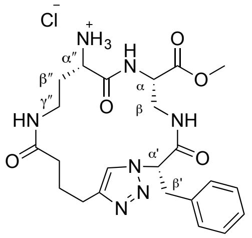	GP2 & GP4<br><br>70%<br><br>94%	**Mp** = 223-225 °C (CH_2Cl_2). **δ_H /ppm** (500 MHz, d_6-DMSO): 9.41 (1H, d, J=6.5 Hz, C_α-NH), 8.24 (3H, d, J=5.5 Hz, NH_3^+), 8.14 (1H, dd, J=8.5, 3.7 Hz, C_β-NH), 7.92-7.82 (2H, m, $C_{\gamma''}$-NH and triazole CH), 7.28-7.02 (5H, m, 5 × ArCH), 5.44 (1H, dd, J=9.5, 5.9 Hz, $H_{\alpha'}$), 4.28 (1H, app. dt, J=6.5, 3.2 Hz, H_α), 4.03 (1H, ddd, J=14.2, 8.5, 6.0 Hz, H_β), 3.73-3.67 (1H, m, $H_{\alpha''}$), 3.58 (3H, s, OCH_3), 3.51-3.46 (1H, m, $H_{\beta'}$), 3.38 (1H, dd, J=14.1, 9.5 Hz, $\underline{H}_{\beta'}$), 3.33-3.25 (1H, m, $H_{\gamma''}$), 3.23-3.18 (1H, m, $\underline{H}_\beta$), 3.10-3.02 (1H, m, $\underline{H}_{\gamma''}$), 2.68-2.52 (2H, m, $COCH_2CH_2CH_2$), 2.27-2.10 (2H, m, $COCH_2CH_2CH_2$), 1.99-1.84 (2H, m, $COCH_2CH_2CH_2$), 1.81-1.68 (2H, m, $H_{\beta''}$). **δ_C /ppm** (125 MHz, d_6-DMSO): 172.9 (C=$OCH_2CH_2CH_2$), 169.9 (COOMe), 168.3 ($C_{\alpha''}C$=O), 167.7 ($C_{\alpha'}C$=O), 145.7 (triazole C), 136.6 (ArC), 128.9 (ArCH), 128.2 (ArCH), 126.6 (ArCH), 122.7 (triazole CH), 64.2 ($C_{\alpha'}$), 52.6 (C_α), 52.3 (OCH_3), 50.0 ($C_{\alpha''}$), 38.9 (C_β), 36.5 ($C_{\beta'}$), 33.9 ($C_{\gamma''}$), 33.1 ($COCH_2CH_2CH_2$), 31.7 ($C_{\beta''}$), 23.7 ($COCH_2CH_2CH_2$), 23.6 ($COCH_2CH_2CH_2$). **v_{max} /cm^{-1}:** 3212 (NH str), 2925 (CH str), 1740 (C=O str), 1686 (C=O str), 1546, 1437, 1214, 1149. **HPLC** (5-100% ACN) Rt 8.58 mins. **HRMS** (ESI+) m/z found $[M+H]^+$ 486.2455, $C_{23}H_{32}N_7O_5^+$ required 486.2465. **$[\alpha]_D^{25}$** = -22.5 (c 0.88, MeOH).
**B8 w**	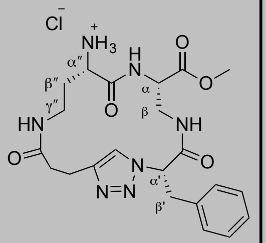	GP2 & GP4<br><br>81%<br><br>98%	**Mp** = 223-226 °C (CH_2Cl_2). **δ_H /ppm** (500 MHz, d_6-DMSO): 8.84 (1H, d, J=6.5 Hz, C_α-NH), 8.50 (1H, dd, J=8.4, 4.1 Hz, C_β-NH), 8.18 (3H, d, J=4.4 Hz, NH_3^+), 8.04 (1H, t, J=5.9 Hz, $C_{\gamma''}$-NH), 7.90 (1H, s, triazole CH), 7.27-7.04 (5H, m, 5 × ArCH), 5.50 (1H dd, J=9.7, 6.0 Hz, $H_{\alpha'}$), 4.07 (1H, ddd, J=9.5, 6.5, 3.3 Hz, H_α), 4.02-3.93 (1H, m, H_β), 3.58 (3H, s, OCH_3), 3.51-3.43 (1H, m, $H_{\alpha''}$), 3.38 (1H, dd, J=14.0, 6.0 Hz, $H_{\beta'}$), 3.27 (1H, dd, J=14.0, 9.7 Hz, $\underline{H}_{\beta'}$), 3.23-3.12 (2H, m, $H_{\gamma''}$ and $\underline{H}_\beta$), 3.11-3.01 (1H, m, $\underline{H}_{\gamma''}$), 2.94-2.76 (2H, m, $COCH_2CH_2$), 2.47-2.37 (2H, m, $COCH_2CH_2$), 1.78-1.65 (1H, m, $H_{\beta''}$), 1.63-1.51 (1H, m, $\underline{H}_{\beta''}$). **δ_C /ppm** (125 MHz, d_6-DMSO): 171.6 (C=OCH_2CH_2), 169.6 (COOMe), 168.3 ($C_{\alpha''}C$=O and $C_{\alpha'}C$=O), 145.3 (triazole C), 136.3 (ArC), 128.8 (ArCH), 128.2 (ArCH), 126.7 (ArCH), 121.5 (triazole CH), 64.0 ($C_{\alpha'}$), 53.2 (C_α), 52.1 (OCH_3), 50.2 ($C_{\alpha''}$), 38.1 (C_β), 37.8 ($C_{\beta'}$), 34.4 ($C_{\gamma''}$), 34.3 ($COCH_2CH_2$), 31.3 ($C_{\beta''}$), 21.5 ($COCH_2CH_2$). **v_{max} /cm^{-1}:** 3215 (NH str), 3035 (CH str), 2925 (CH str), 1742 (C=O str), 1682 (C=O str), 1542, 1433, 1213, 1151. **HPLC** (5-100% ACN) Rt 8.31 mins. **HRMS** (ESI+) m/z found $[M+H]^+$ 472.2301, $C_{22}H_{30}N_7O_5^+$ required 472.2303. **$[\alpha]_D^{25}$** = -28.2 (c 0.53, MeOH).
**B9 w**	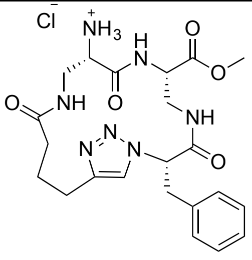	GP2 & GP4<br><br>99%<br><br>61%	**HPLC** (5-100% ACN) Rt 6.74 mins. **LCMS** $[M+H]^+$ 472.16.

**B10 w**	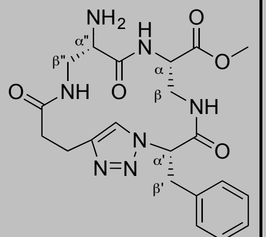	GP2 & GP4<br>83%<br>85%	**R_f** = 0.27 (10% MeOH/ 90% CH_2Cl_2). **δ_H /ppm** (400 MHz, d_6-DMSO, 120 °C): 7.68 (1H, app s, C_β-N*H*), 7.66 (1H, s, triazole C*H*), 7.26-7.11 (5H, m, 5 × ArC*H*), 6.94 (1H, s, N*H*), 5.39 (1H, dd, *J*=8.3, 6.6 Hz, $H_{\alpha'}$), 4.25 (dd, *J*=8.5, 3.6 Hz, H_α), 3.76-3.67 (1H, m, H_β), 3.67 (3H, s, OCH_3), 3.54 (1H, dd, *J*=14.5, 6.6 Hz, $H_{\beta'}$), 3.47-3.27 (5H, m, $\underline{H}_{\beta'}$,$H_{\beta''}$, $H_{\alpha''}$, $\underline{H}_\beta$), 2.95-2.89 (2H, m, $COCH_2CH_2$), 2.42 (2H, t, *J*=6.0 Hz, $COCH_2CH_2$). **δ_C /ppm** (125 MHz, d_6-DMSO): 173.7 ($C_{\alpha''}C$=O), 172.9 (*C*=OCH_2CH_2), 170.3 (*C*OOMe), 168.2 ($C_{a'}C$=O), 146.2 (triazole *C*), 136.4 (Ar*C*), 129.0 (Ar*C*H), 128.3 (Ar*C*H), 126.7 (Ar*C*H), 120.8 (triazole *C*H), 64.0 ($C_{\alpha'}$), 54.2 ($C_{\alpha''}$), 54.2 (C_α), 52.2 (OCH_3), 42.1 ($C_{\beta''}$), C_β below DMSO signal, 36.6 ($C_{\beta'}$), 35.2 ($COCH_2CH_2$), 21.4 ($COCH_2CH_2$). **v_{max} /cm^{-1}:** 3331 (w, N-H), 2925 (w, C-H), 1743 (m, C=O), 1663 (s, C=O), 1522 (m, C=C). **HPLC** (5-100% ACN) *Rt* 9.11 mins. **HRMS** (ESI+) *m/z* found $[M+H]^+$ 458.2153, $C_{21}H_{28}N_7O_5^+$ required 458.2152 (Δ 0.2 ppm). **$[\alpha]_D^{25}$** = +11.2 (*c* 1.56, MeOH).
**B11 w**	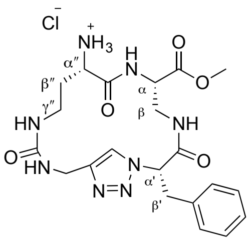	GP2 & GP4<br>64%<br>94%	**Mp** = 224-225 °C (CH_2Cl_2). **δ_H /ppm** (500 MHz, d_6-DMSO): 8.56 (1H, d, *J*=7.0 Hz, C_α-N*H*), 8.32 (1H, dd, *J*=7.2, 4.4 Hz, C_β-N*H*), 8.19 (3H, d, *J*=4.3 Hz, NH_3^+), 8.02 (1H, s, triazole C*H*), 7.27-7.06 (5H, m, 5 × ArC*H*), 6.62 (1H, app. s, N*H*CH_2), 6.38 (1H, app. s, $C_{\gamma''}$-N*H*), 5.55 (1H, dd, *J*=10.0, 5.6 Hz, $H_{\alpha'}$), 4.30 (1H, dt, *J*=7.0, 4.1 Hz, H_α), 4.26-4.07 (2H, m, NHCH_2), 3.87-3.77 (1H, m, H_β), 3.62 (3H, s, OCH_3), 3.52 (1H, dd, *J*=14.3, 5.6 Hz, $H_{\beta'}$), $H_{\alpha''}$ below H_2O peak, 3.33-3.24 (2H, m, $\underline{H}_\beta$ and $\underline{H}_{\beta'}$), 3.19-3.08 (2H, m, $H_{\gamma''}$), 1.92-1.81 (1H, m, $H_{\beta''}$), 1.70-1.59 (1H, m, $\underline{H}_{\beta''}$). **δ_C /ppm** (125 MHz, d_6-DMSO): 169.8 (*C*OOMe), 169.0 ($C_{\alpha''}C$=O), 168.3 ($C_{\alpha'}C$=O), 159.1 (NH*C*=ONH), 146.9 (triazole C), 136.4 (Ar*C*), 128.8 (Ar*C*H), 128.3 (Ar*C*H), 126.7 (Ar*C*H), 122.1 (triazole *C*H), 64.1 ($C_{\alpha'}$), 52.7 (C_α), 52.3 (OCH_3), 49.9 ($C_{\alpha''}$), 38.5 (C_β), 37.0 ($C_{\beta'}$), 35.7 (NHCH_2), 35.0 ($C_{\gamma''}$), 31.9 ($C_{\beta''}$). **v_{max} /cm^{-1}:** 3210 (NH str), 2921 (CH str), 1740 (C=O str), 1683 (C=O str), 1553, 1436, 1214, 1147. **HPLC** (5-100% ACN) *Rt* 8.33 mins. **HRMS** (ESI+) *m/z* found $[M+H]^+$ 473.2267, $C_{21}H_{29}N_8O_5^+$ required 473.2261. **$[\alpha]_D^{25}$** = -13.9 (*c* 0.35, MeOH).
**B12 w**	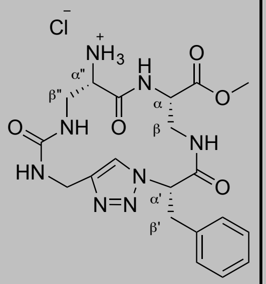	GP2 & GP4<br>99%<br>96%	**Mp** = 212-216 °C (CH_2Cl_2). **δ_H /ppm** (500 MHz, d_6-DMSO): 8.57 (1H, d, *J*=6.0 Hz, C_α-N*H*), 8.51-8.45 (1H, m, $C_{\beta''}$-N*H*), 8.42 (3H, d, *J*=5.6 Hz, NH_3^+), 8.16 (1H, s, triazole C*H*), 7.27-7.08 (5H, m, 5 × ArC*H*), 6.51 (1H, s, C_β-N*H*), 6.32 (1H, t, *J*=5.6 Hz, N*H*CH_2), 5.46 (1H, dd, *J*=8.2, 6.8 Hz, $H_{\alpha'}$), 4.53-4.36 (1H, m, H_β), 4.18-4.07 (2H, m, $H_{\alpha''}$ and H_α), 4.07-3.92 (2H, m, $H_{\beta''}$ and $\underline{H}_\beta$), 3.62-3.52 (1H, m, $H_{\beta'}$), 3.47-3.39 (1H, m, NHCH_2), 3.37 (3H, s, OCH_3), 3.27 (1H, dd, *J*=14.1, 8.2 Hz, $\underline{H}_{\beta'}$), 3.07-2.95 (2H, m, $\underline{H}_{\beta''}$ and NHC$\underline{H}_2$). **δ_C /ppm** (125 MHz, d_6-DMSO): 170.0 (*C*OOMe), 167.8 ($C_{\alpha''}C$=O), 166.9 ($C_{\alpha'}C$=O), 158.0 (NH*C*=ONH), 147.5 (triazole C), 136.7 (Ar*C*), 129.1 (Ar*C*H), 128.2 (Ar*C*H), 126.6 (Ar*C*H), 122.7 (triazole *C*H), 63.9 ($C_{\alpha'}$), 52.9 ($C_{\alpha''}$), 52.3 (OCH_3), 50.9 (C_α), 40.5 (NHCH_2), 38.9 ($C_{\beta''}$), 35.7 ($C_{\beta'}$), 35.4 (C_β). **v_{max} /cm^{-1}:** 3208 (NH str), 3031 (CH str), 2919 (CH str), 1741 (C=O str), 1678 (C=O str), 1546, 1435, 1211, 1146. **HPLC** (5-100% ACN) *Rt* 8.74 mins. **HRMS** (ESI+) *m/z* found $[M+H]^+$ 459.2104, $C_{20}H_{27}N_8O_5^+$ required 459.2104.**$[\alpha]_D^{25}$** = -0.5 (*c* 2.05, MeOH).

**B14 w**	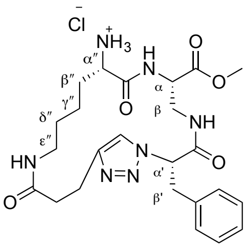	GP2 & GP4<br><br>98%<br><br>96%	**Mp** = 135-138 °C (CH_2Cl_2). **δ_H /ppm** (500 MHz, *d*$_6$-DMSO): 9.06 (1H, dd, *J*=8.6, 3.7 Hz, C_β-N*H*), 8.93 (1H, d, *J*=6.7 Hz, C_α-N*H*), 8.20 (3H, d, *J*=4.5 Hz, NH_3^+), 7.70 (1H, s, triazole C*H*), 7.64 (1H, t, *J*=5.5 Hz, $C_{\varepsilon''}$-N*H*), 7.29-7.07 (5H , m, 5 × ArC*H*), 5.49 (1H, dd, *J*=9.2, 6.3 Hz, $H_{\alpha'}$), 4.30-4.24 (1H, m, H_α), 3.94 (1H, ddd, *J*=14.0, 8.6, 5.4 Hz, H_β), 3.83-3.74 (1H, m, $H_{\alpha''}$), 3.47 (3H, s, OCH_3), 3.42-3.28 (2H, m, $H_{\beta'}$), 3.22-3.13 (1H, m, $\underline{H_\beta}$), 3.12-2.97 (2H, m, $H_{\varepsilon''}$), 2.85 (2H, t, *J*=6.2 Hz, $COCH_2CH_2$), 2.44-2.33 (2H, m, $COCH_2CH_2$), 1.83-1.72 (1H, m, $H_{\beta''}$), 1.69-1.58 (1H, m, $\underline{H_{\beta''}}$), 1.42-1.35 (2H, m, $H_{\delta''}$), 1.35-1.26 (2H, m, $H_{\gamma''}$). **δ_C /ppm** (125 MHz, *d*$_6$-DMSO): 171.0 (*C*=OCH_2CH_2), 169.7 (*C*OOMe), 168.6 ($C_{\alpha''}$*C*=O), 168.5 ($C_{\alpha'}$*C*=O), 145.9 (triazole C), 136.2 (Ar*C*), 128.9 (Ar*C*H), 128.3 (Ar*C*H), 126.8 (Ar*C*H), 121.6 (triazole *C*H), 63.9 ($C_{\alpha'}$), 53.2 (C_α), 52.1 (O*C*H_3), 51.8 ($C_{\alpha''}$), C_β below DMSO signal, 37.7 ($C_{\varepsilon''}$), 37.3 ($C_{\beta'}$), 33.9 (CO*C*H_2CH_2), 30.5 ($C_{\beta''}$), 28.3 ($C_{\delta''}$), 21.1 ($COCH_2$*C*H_2), 20.8 ($C_{\gamma''}$). **v_{max} /cm^{-1}:** 3226 (NH str), 2921 (CH str), 1740 (C=O str), 1687 (C=O str), 1545, 1436, 1212, 1148. **HPLC** (5-100% ACN) *Rt* 8.8 mins. **HRMS** (ESI+) *m/z* found $[M+H]^+$ 500.2618, $C_{24}H_{34}N_7O_5^+$ required 500.2616. **$[\alpha]_D^{25}$** = +24.7 (*c* 2.54, MeOH)
**D1 w**	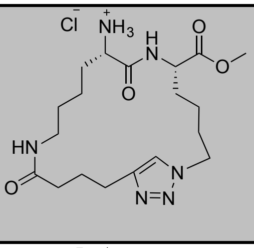	GP2 & GP4<br><br>98%<br><br>68%	**HPLC** (5-30% ACN) *Rt* 6.74 mins. **LCMS** $[M+H]^+$ 409.18.
**D2 w**	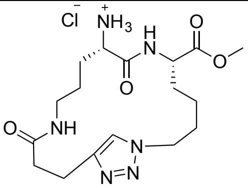	GP2 & GP4<br><br>93%<br><br>90%	**HPLC** (5-100% ACN) *Rt* 7.13 mins. **LCMS** $[M+H]^+$ 381.22.
**D3 w**	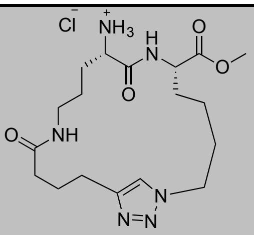	GP2 & GP4<br><br>99%<br><br>85%	**HPLC** (0-60% ACN) *Rt* 5.44 mins. **LCMS** $[M+H]^+$ 395.17.
**D5 w**	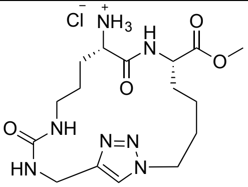	GP2 & GP4<br><br>99%<br><br>83%	**HPLC** (5-100% ACN) *Rt* 4.37 mins. **LCMS** $[M+H]^+$ 382.12.

**D7 w**	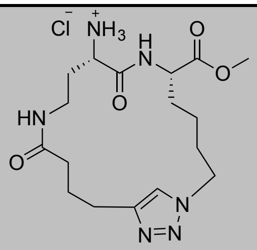	GP2 & GP4<br>99%<br>89%	**HPLC** (5-45% ACN) *Rt* 7.13 mins. **LCMS** $[M+H]^+$ 381.15.
**D8 w**	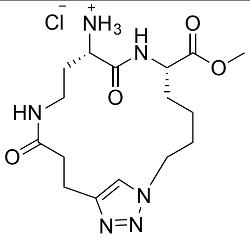	GP2 & GP4<br>62%<br>84%	**HPLC** (5-100% ACN) *Rt* 4.71 mins. **LCMS** $[M+H]^+$ 367.21.
**D10 w**	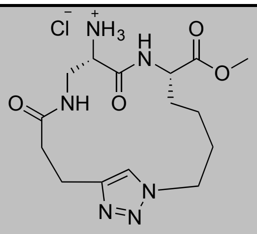	GP2 & GP4<br>59%<br>85%	**HPLC** (5-100% ACN) *Rt* 4.45 mins. **LCMS** $[M+H]^+$ 353.12.
**D11 w**	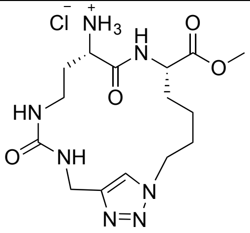	GP2 & GP4<br>99%<br>73%	**HPLC** (5-100% ACN) *Rt* 4.60 mins. **LCMS** $[M+H]^+$ 368.26.
**D12 w**	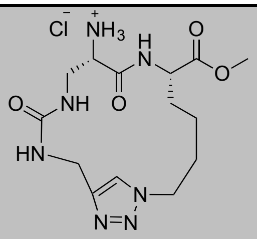	GP2 & GP4<br>56%<br>44%	**HPLC** (5-100% ACN) *Rt* 4.29 mins. **LCMS** $[M+H]^+$ 354.09.
**D14 w**	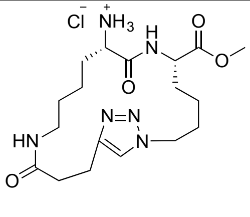	GP2 & GP4<br>30%<br>89%	**HPLC** (5-80% ACN) *Rt* 4.86 mins. **LCMS** $[M+H]^+$ 395.23.

**E1w**	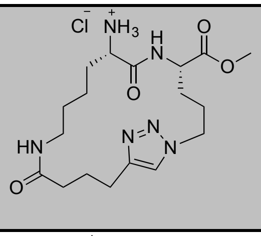	GP2 & GP4<br>40%<br>67%	**HPLC** (5-100% ACN) *Rt* 4.62 mins. **LCMS** $[M+H]^+$ 395.24.
**E2w**	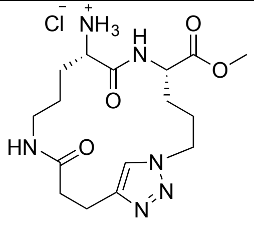	GP2 & GP4<br>85%<br>74%	**HPLC** (5-100% ACN) *Rt* 4.09 mins. **LCMS** $[M+H]^+$ 367.21.
**E3w**	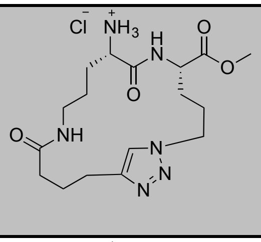	GP2 & GP4<br>71%<br>86%	**HPLC** (5-100% ACN) *Rt* 4.43 mins. **LCMS** $[M+H]^+$ 381.30.
**E4w**	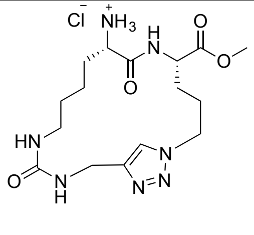	GP2 & GP4<br>93%<br>70%	**HPLC** (5-100% ACN) *Rt* 3.83 mins. **LCMS** $[M+H]^+$ 382.20.
**E5w**	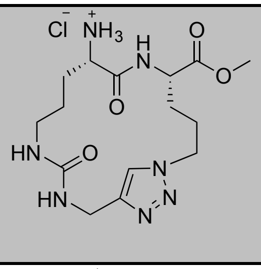	GP2 & GP4<br>99%<br>74%	**HPLC** (5-100% ACN) *Rt* 3.86 mins. **LCMS** $[M+H]^+$ 368.26.
**E7w**	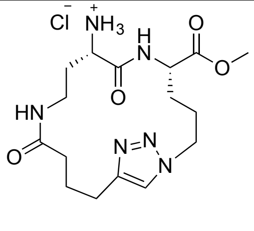	GP2 & GP4<br>60%<br>71%	**HPLC** (5-100% ACN) *Rt* 4.70 mins. **LCMS** $[M+H]^+$ 367.21.
**E8w**	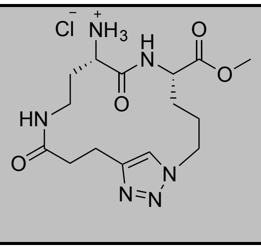	GP2 & GP4<br>99%<br>58%	**HPLC** (5-100% ACN) *Rt* 4.14 mins. **LCMS** $[M+H]^+$ 353.19.

**E9w**	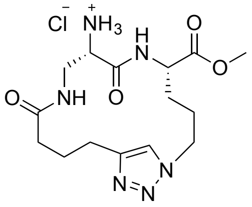	GP2 & GP4<br>52%<br>59%	**HPLC** (5-100% ACN) *Rt* 4.41 mins. **LCMS** $[M+H]^+$ 353.12.
**E10w**	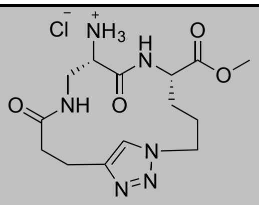	GP2 & GP4<br>53%<br>94%	**HPLC** (5-100% ACN) *Rt* 3.85 mins. **LCMS** $[M+H]^+$ 339.10.
**E11w**	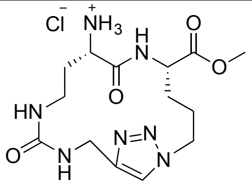	GP2 & GP4<br>29%<br>97%	**HPLC** (5-100% ACN) *Rt* 4.04 mins. **LCMS** $[M+H]^+$ 354.17.
**E14w**	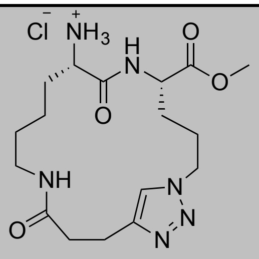	GP2 & GP4<br>99%<br>73%	**HPLC** (5-100% ACN) *Rt* 4.11 mins. **LCMS** $[M+H]^+$ 381.22.

12. Preparation of B/C/C/P and B/C/C/C/P CuAAC Macrocycles

	Compound	Method, Yield (%), Purity (%)	Analysis
**G1 w**	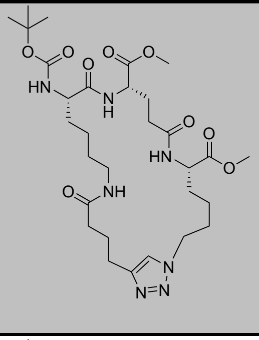	GP2<br>70%<br>61%	**HPLC** (5-100% ACN) *Rt* 7.70 mins. **LCMS** $[M+H]^+$ 652.39.
**G2 w**	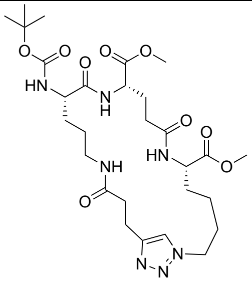	GP2<br>71%<br>83%	**HPLC** (5-100% ACN) *Rt* 7.39 mins. **LCMS** $[M+H]^+$ 624.54.
**G3 w**	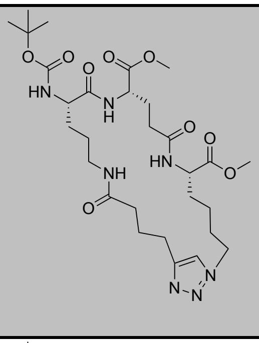	GP2<br>99%<br>87%	**HPLC** (5-100% ACN) *Rt* 7.51 mins. **LCMS** $[M+H]^+$ 638.28.
**G4 w**	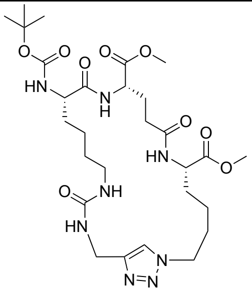	GP2<br>62%<br>92%	**δ_H /ppm** (400 MHz, d_6-DMSO): 8.14 (1H, d, *J*=7.2 Hz), 8.05 (1H, d, *J*=8.3 Hz), 7.78 (1H, s), 6.79 (1H, d, *J*=7.8 Hz), 6.26 (1H, t, *J*=5.9 Hz), 5.93 (1H, t, *J*=5.9 Hz), 4.33-4.16 (5H, m), 3.95-3.83 (1H, m), 3.58 (3H, s), 3.57 (3H, s), 3.16-3.02 (1H, m), 2.95-2.80 (1H, m), 1.84-1.08 (13H, m), 1.37 (9H, s). **δ_C /ppm** (100 MHz, d_6-DMSO): 173.1, 172.8, 172.6, 171.9, 158.7, 122.9, 78.5, 54.3, 52.3, 51.8, 51.4, 49.6, 39.1, 35.6, 31.9, 30.8, 30.9, 29.9, 29.7, 28.7, 26.4, 22.8, 22.4. **ν_{max} /cm^{-1}:** 3304, 2920, 1739, 1650, 1544, 1436, 1366, 1249, 1212, 1164, 1048. **HPLC** (5-100% ACN) *Rt* 7.56 mins. **HRMS** (ESI+) *m/z* found $[M+Na]^+$ 661.3287, $C_{28}H_{46}N_8O_9Na^+$ required 661.3285.

**G5** **w**	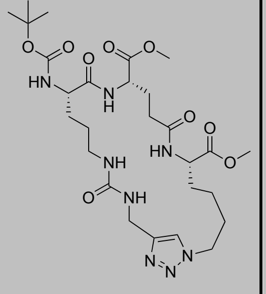	GP2<br>60%<br>98%	**δ_H /ppm** (500 MHz, *d*$_6$-DMSO): 8.18 (1H, d, *J*=7.8 Hz), 8.02 (1H, d, *J*=8.3 Hz), 7.82 (1H, s), 6.79 (1H, d, *J*=8.5 Hz), 6.39 (1H, t, *J*=5.9 Hz), 6.04 (1H, t, *J*=6.1 Hz), 4.40-4.06 (7H, m), 3.60 (3H, s), 3.59 (3H, s), 3.28- 3.13 (1H, m), 2.96-2.84 (1H, m), 2.31-2.20 (1H, m), 2.10 (1H, m), 2.04-1.90 (1H, m), 1.88-1.55 (4H, m), 1.53-1.42 (1H, m), 1.40-1.30 (4H, m), 1.37 (9H, s), 1.12-1.01 (2H, m). **δ_C /ppm** (125 MHz, *d*$_6$-DMSO): 172.6, 172.2, 171.4, 158.8, 155.6, 146.5, 123.0, 78.1, 52.8, 52.0, 52.0, 50.9, 50.8, 49.0, 37.2, 35.3, 30.8, 30.0, 28.7, 28.3, 27.1, 26.7, 21.8. **ν_{max} /cm^{-1}:** 3314, 2924, 1733, 1648, 1525, 1251, 1169, 1051. **HPLC** (5-45% ACN) *Rt* 11.65 mins. **HRMS** (ESI+) *m/z* found $[M+H]^+$ 625.3298, $C_{27}H_{45}N_8O_9^+$ required 625.3310.
**G9** **w**	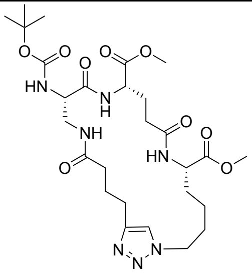	GP2<br>69%<br>81%	**HPLC** (5-45% ACN) *Rt* 12.13 mins. **LCMS** $[M+H]^+$ 610.23.
**G10** **w**	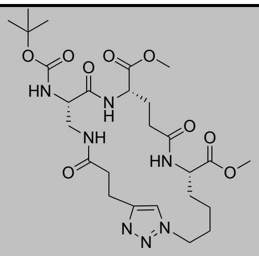	GP2<br>94%<br>89%	**HPLC** (5-45% ACN) *Rt* 11.83 mins. **LCMS** $[M+H]^+$ 596.21.
**G11** **w**	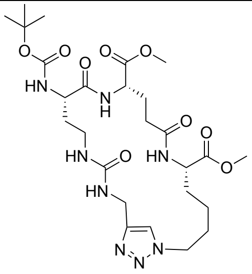	GP2<br>67%<br>74%	**HPLC** (15-30% ACN) *Rt* 11.08 mins. **LCMS** $[M+H]^+$ 611.21.
**G12** **w**	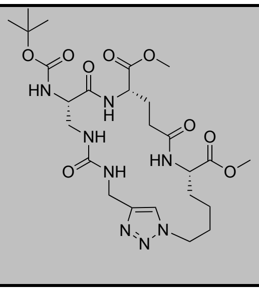	GP2<br>99%<br>85%	**HPLC** (15-30% ACN) *Rt* 11.55 mins. **LCMS** $[M+H]^+$ 597.18.

**G13 w**	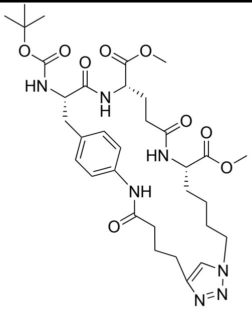	GP2<br>12%<br>93%	**HPLC** (5-100% ACN) *Rt* 8.46 mins. **LCMS** $[M+H]^+$ 686.29.
**G14 w**	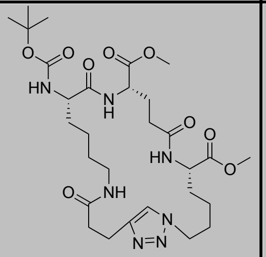	GP2<br>69%<br>82%	**HPLC** (5-100% ACN) *Rt* 7.49 mins. **LCMS** $[M+H]^+$ 638.28.
**H12 w**	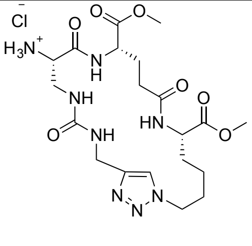	GP2 & GP4<br>78%<br>90%	**HPLC** (5-100% ACN) *Rt* 4.94 mins. **LCMS** $[M+H]^+$ 483.11.
**H14 w**	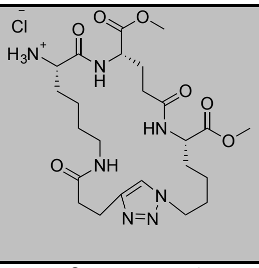	GP2 & GP4<br>86%<br>74%	**HPLC** (5-30% ACN) *Rt* 7.31 mins. **LCMS** $[M+H]^+$ 542.28.
**J13 w**	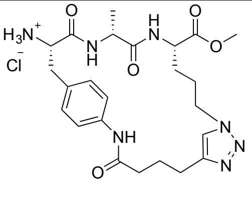	GP2 & GP5<br>21%<br>72%	**HPLC** (5-100% ACN) *Rt* 5.34 mins. **LCMS** $[M+H]^+$ 500.28.

13. Preparation of B/C/P RuAAC Macrocycles

	Compound	Method, Yield (%), Purity (%)	Analysis
**A1x**	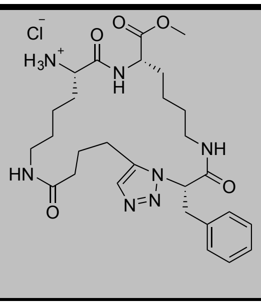	GP3 & GP4<br>60%<br>86%	**HPLC** (5-80% ACN) *Rt* 7.74 mins. **LCMS** $[M+H]^+$ 556.27.
**A2x**	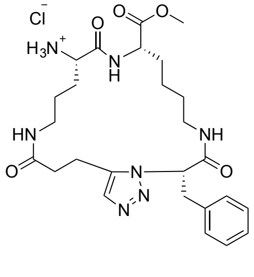	GP3 & GP4<br>97%<br>81%	**HPLC** (5-60% ACN) *Rt* 8.86 mins. **LCMS** $[M+H]^+$ 528.29.
**A3x**	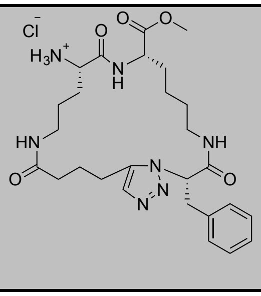	GP3 & GP4<br>86%<br>75%	**HPLC** (5-100% ACN) *Rt* 6.93 mins. **LCMS** $[M+H]^+$ 542.28.
**A4x**	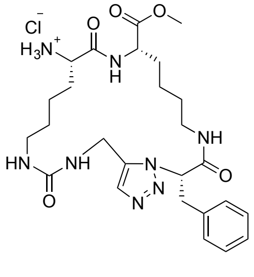	GP3 & GP4<br>81%<br>81%	**HPLC** (5-100% ACN) *Rt* 6.79 mins. **LCMS** $[M+H]^+$ 543.33.

**A5x**	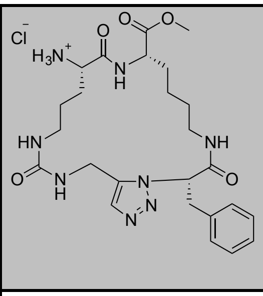	GP3 &<br>GP4<br>56%<br>94%	**HPLC** (5-100% ACN) *Rt* 6.67 mins. **LCMS** $[M+H]^+$ 529.23.
**A6x**	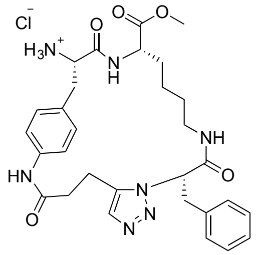	GP3 &<br>GP5<br>57%<br>70%	**δ_H /ppm** (500 MHz, d_6-DMSO): 10.13 (1H, s), 8.47 (3H, d, *J*=4.3 Hz), 8.26 (1H, d, *J*=5.1 Hz,), 7.71 (1H, t, *J*=5.5 Hz), 7.47 (1H, m), 7.40 (2H, d, *J*=8.4 Hz), 7.21-7.09 (5H, m), 7.06 (2H, d, *J*=6.9 Hz), 5.42 (1H, dd, *J*=9.6, 6.0 Hz), 4.10-3.98 (1H, m), 3.59 (1H, m), 3.47 (3H, s), 3.51-3.38 (2H, m), 3.17 (1H, dd, 1H, *J*=13.6, 4.7 Hz), 3.14-3.05 (1H, m), 2.84-2.71 (3H, m), 2.71-2.56 (2H, m), 2.55-2.40 (1H, m), 1.54-1.37 (1H, m), 1.33-0.92 (3H, m), 0.88-0.57 (1H, m). **δ_C /ppm** (125 MHz, d_6-DMSO): 170.8, 170.3, 166.9, 137.7, 137.6, 137.0, 131.5, 129.8, 129.5, 129.0, 128.2, 126.6, 119.4, 72.2, 70.6, 66.4, 61.8, 60.3, 52.8, 52.70, 51.8, 43.7, 36.7, 36.0, 34.6, 29.4, 28.4 22.2, 18.0. **ν_{max} /cm^{-1}:** 3295, 2925, 1732, 1663, 1540, 701. **HPLC** (5-100% ACN) *Rt* 7.52mins. **HRMS** (ESI+) *m/z* found $[M+H]^+$ 576.2928, $C_{30}H_{38}N_7O_5^+$ required 576.2934.
**A7x**	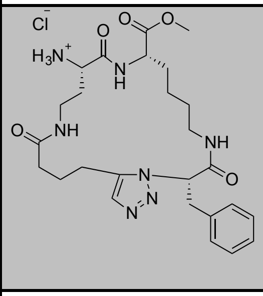	GP3 &<br>GP4<br>87%<br>76%	**HPLC** (5-100% ACN) *Rt* 7.11 mins. **LCMS** $[M+H]^+$ 528.23.
**A8x**	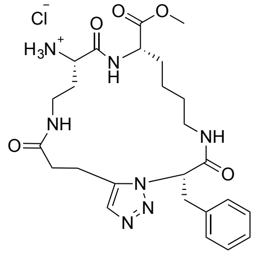	GP3 &<br>GP4<br>90%<br>85%	**HPLC** (5-100% ACN) *Rt* 7.04 mins. **LCMS** $[M+H]^+$ 514.24.
**A9x**	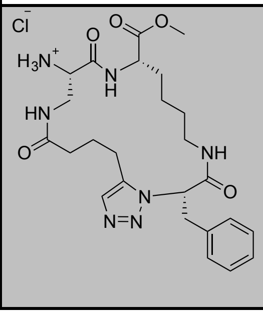	GP3 &<br>GP4<br>99%<br>88%	**HPLC** (5-100% ACN) *Rt* 6.88 mins. **LCMS** $[M+H]^+$ 514.23.

**A10 x**	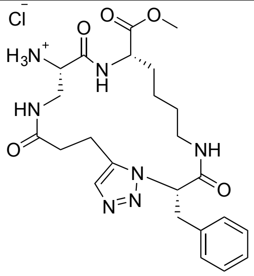	GP3 & GP4<br>99%<br>74%	**HPLC** (5-100% ACN) *Rt* 6.63 mins. **LCMS** $[M+H]^+$ 500.21.
**A11 x**	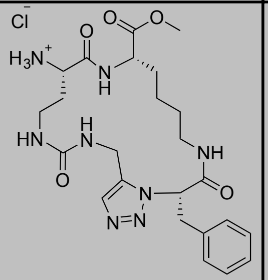	GP3 & GP4<br>78%<br>84%	**HPLC** (5-100% ACN) *Rt* 6.94 mins. **LCMS** $[M+H]^+$ 515.28.
**A12 x**	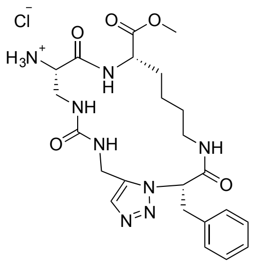	GP3 & GP4<br>38%<br>97%	**δ_H /ppm** (500 MHz, d_6-DMSO): 8.92 (1H, d, *J*=5.5 Hz), 8.43 (3H, d, *J*=4.5 Hz), 8.01 (1H, t, *J*=6.0 Hz), 7.51 (1H, s), 7.23-7.11 (4H, m), 7.05-6.99 (2H, m), 6.32 (1H, t, *J*=6.0 Hz), 5.43 (1H, dd, *J*=10.5, 5.0 Hz), 4.23-4.11 (1H, m), 4.01-3.77 (3H, m), 3.60 (3H, s), 3.59-3.46 (2H, m), 3.44-3.32 (1H, m), 3.28-3.13 (2H, m), 3.09-2.98 (1H, m), 1.88-1.74 (1H, m), 1.70-1.58 (1H, m), 1.52-1.25 (3H, m), 1.16-1.01 (1H, m). **δ_C /ppm** (125 MHz, d_6-DMSO): 171.5, 167.9, 167.1, 158.0, 136.6, 136.1, 132.3, 128.7, 128.2, 126.6, 61.9, 52.8, 52.6, 52.0, 41.0, 38.2, 37.6, 31.9, 28.8, 28.0, 21.4. **HPLC** (5-100% ACN) *Rt* 6.78 mins. **HRMS** (ESI+) *m/z* found $[M+H]^+$ 501.2585, $C_{23}H_{33}N_8O_5^+$ required 501.2574. $[\alpha]_D^{25}$ = +44.5 (*c* 0.50, MeOH).
**A13 x**	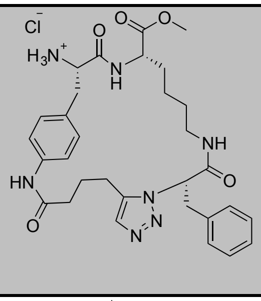	GP3 & GP5<br>50%<br>89%	**ν_{max} /cm^{-1}:** 3311, 2922, 2852, 2161, 2029, 1726, 1661, 1601, 1537, 1456, 1416, 1253, 1119. **HPLC** (5-100% ACN) *Rt* 7.40 mins. **HRMS** (ESI+) *m/z* found $[M+H]^+$ 590.3080, $C_{31}H_{40}N_7O_5^+$ required 590.3090.
**B1x**	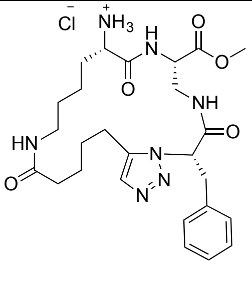	GP3 & GP4<br>99%<br>91%	**HPLC** (5-100% ACN) *Rt* 6.53 mins. **LCMS** $[M+H]^+$ 514.31.

**B2x**	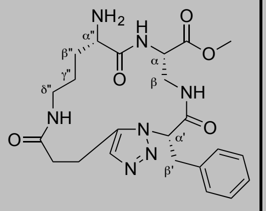	GP3 & GP4<br><br>93%<br><br>99%	**δ$_H$ /ppm** (500 MHz, d_6-DMSO, 90°C): 8.04 (1H, app s, C_α-N*H*), 7.93 (3H, br s, NH_3^+), 7.87 (1H, app s, $C_{\delta''}$-N*H*), 7.79 (1H, app s, C_β-N*H*), 7.45 (1H, s, triazole C*H*), 7.24-7.16 (3H, m, 3 × ArC*H*), 7.14-7.10 (2H, m, 2 × ArC*H*), 5.31 (1H, dd, *J* = 8.7, 6.5 Hz, $H_{\alpha'}$), 4.36-431 (1H, m, H_α), 3.93 (2H, m, H_β and $H_{\alpha''}$), 3.57-3.53 (2H, m, $H_{\beta'}$), 3.51 (3H, s, OCH_3), 3.45-3.36 (1H, m, $H_{\delta''}$), 3.33-3.27 (1H, m, H_β), $H_{\delta''}$ below H_2O signal, 2.80-2.69 (1H, m, $COCH_2CH_2$), 2.69-2.58 (1H, m, $COCH_2CH_2$), $COCH_2CH_2$ below DMSO signal, 1.70-1.56 (3H, m, $H_{\beta''}$ and $H_{\gamma''}$), 1.53-1.42 (1H, m, $H_{\gamma''}$). **δ$_C$ /ppm** (125 MHz, d_6-DMSO): 171.8 (C=OCH_2CH_2), 169.8 (*C*OOMe), 169.4 ($C_{\alpha''}C$=O), 167.8 ($C_{a'}C$=O), 137.2 (triazole *C*), 136.8 (Ar*C*), 131.0 (triazole *C*H), 129.0 (Ar*C*H), 128.3 (Ar*C*H), 126.7 (Ar*C*H), 62.0 ($C_{\alpha'}$), 52.4 (C_α), 52.2 (OCH_3), 50.9 ($C_{\alpha''}$), C_β below DMSO signal, 36.5 ($C_{\beta'}$), 36.3 ($C_{\delta''}$), 33.6 ($COCH_2CH_2$), 28.0 ($C_{\beta''}$), 24.3 ($C_{\gamma''}$), 19.1 ($COCH_2CH_2$). **v_{max} /cm^{-1}:** 3276 (w, N-H), 2951 (w, C-H), 1743 (w, C=O), 1673 (s, C=O), 1545 (m, C=C). **HPLC** (5-100% ACN) *Rt* 8.85 mins. **HRMS** (ESI+) *m/z* found $[M+Na]^+$ 508.2280, $C_{23}H_{31}N_7O_5Na^+$ required 508.2284 (Δ -0.8 ppm). **$[\alpha]_D^{25}$** = +50.0 (*c* 1.46, MeOH).
**B3x**	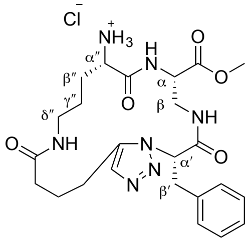	GP3 & GP4<br><br>46%<br><br>99%	**Mp** = 202-204 °C (CH_2Cl_2). **δ$_H$ /ppm** (500 MHz, d_6-DMSO): 8.99 (1H, d, *J*=7.0 Hz, C_α-N*H*), 8.91 (1H, dd, *J*=8.4, 4.1 Hz, C_β-N*H*), 8.19 (3H, d, *J*=4.8 Hz, NH_3^+), 8.15 (1H, t, *J*=6.1 Hz, $C_{\delta''}$-N*H*), 7.41 (1H, s, triazole C*H*), 7.23-7.11 (5H, m, 5 × ArC*H*), 5.29 (1H, dd, *J*=10.2, 5.3 Hz, $H_{\alpha'}$), 4.28 (1H, dt, *J*=7.0, 3.5 Hz, H_α), 4.05-3.89 (2H, m, $H_{\alpha''}$ and H_β), 3.62-3.49 (2H, m, $H_{\beta'}$), 3.28 (3H, s, OCH_3), 3.24-3.10 (2H, m, H_β and $H_{\delta''}$), 3.09-2.96 (1H, m, $H_{\delta''}$), 2.39-2.30 (2H, m, $COCH_2CH_2CH_2$), 2.27-2.18 (1H, m, $COCH_2CH_2CH_2$), 2.18-2.09 (1H, m, $COCH_2CH_2CH_2$), 1.83-1.67 (3H, m, $COCH_2CH_2CH_2$ and $H_{\beta''}$), 1.67-1.52 (2H, m, $H_{\gamma''}$), 1.52-1.39 (1H, m, $COCH_2CH_2CH_2$). **δ$_C$ /ppm** (125 MHz, d_6-DMSO): 171.9 (C=$OCH_2CH_2CH_2$), 169.8 (*C*OOMe), 169.0 ($C_{\alpha''}C$=O), 167.7 ($C_{\alpha'}C$=O), 138.0 (triazole C), 136.8 (Ar*C*), 130.8 (triazole *C*H), 128.9 (Ar*C*H), 128.2 (Ar*C*H), 126.7 (Ar*C*H), 61.8 ($C_{\alpha'}$), 52.7 (C_α), 51.9 (OCH_3), 51.3 ($C_{\alpha''}$), C_β below DMSO peak, 37.1 ($C_{\beta'}$), 37.1 ($C_{\delta''}$), 34.6 ($COCH_2CH_2CH_2$), 28.3 ($C_{\beta''}$), 24.3 ($C_{\gamma''}$), 23.9 ($COCH_2CH_2CH_2$), 21.3 ($COCH_2CH_2CH_2$). **v_{max} /cm^{-1}:** 3222 (NH str), 2926 (CH str), 1736 (C=O str), 1683 (C=O str), 1544, 1438, 1213, 1140. **HPLC** (5-100% ACN) *Rt* 6.49 mins. **HRMS** (ESI+) *m/z* found $[M+H]^+$ 500.2632, $C_{24}H_{34}N_7O_5^+$ required 500.2621. **$[\alpha]_D^{25}$** = +53.0 (*c* 0.15, MeOH).
**B4x**	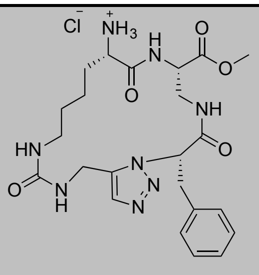	GP3 & GP4<br><br>96%<br><br>94%	**HPLC** (5-25% ACN) *Rt* 13.76 mins. **LCMS** $[M+H]^+$ 501.26.

**B5x**	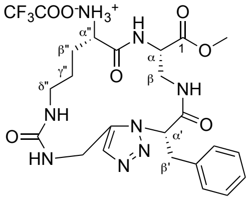	GP3 & GP4<br>86%<br>92%	**δ_H /ppm** (500 MHz, d_6-DMSO, 90°C): 8.30 (1H, app s, C_{α}-N*H*), 7.80 (3H, br s, NH_3^+), 7.58 (1H, dd, *J*=5.8, 4.5 Hz, C_{β}-N*H*), 7.43 (1H, s, triazole C*H*), 7.24-7.12 (3H, m, 3 × ArC*H*), 7.01-6.95 (2H, m, 2 × ArC*H*), 6.56 (1H, app t, *J*=5.9 Hz, CH_2-N*H*), 6.38-6.25 (1H, m, $C_{\delta''}$-N*H*), 5.62 (1H, dd, *J*=10.4, 5.1 Hz, $H_{\alpha'}$), 4.42 (1H, app s, H_{α}), 4.02 (1H, dd, *J*=16.3, 6.3 Hz, CH_2), 3.98-3.91 (2H, m, H_{β} and $H_{\alpha''}$), 3.74 (1H, dd, *J*=16.3, 5.5 Hz, $C\underline{H}_2$), 3.55 (1H, dd, *J*=14.1, 5.1 Hz, $H_{\beta'}$), 3.44-3.27 (3H, m, $\underline{H}_{\beta}$, $\underline{H}_{\beta'}$, $H_{\delta''}$), 2.86-2.74 (1H, m, $\underline{H}_{\delta''}$), 1.80-1.61 (2H, m, $H_{\beta''}$), 1.61-1.44 (2H, m, $H_{\gamma''}$). **δ_C /ppm** (125 MHz, d_6-DMSO):. 169.2 (*C*OOMe), 169.0 ($C_{\alpha''}$*C*=O), 167.3($C_{a'}$*C*=O), 158.8 (NH*C*=ONH), 137.3 (triazole *C*), 136.7 (Ar*C*), 131.8 (triazole *C*H), 128.8 (Ar*C*H), 128.2 (Ar*C*H), 126.6 (Ar*C*H), 61.2 ($C_{\alpha'}$), 52.3(C_{α}), 52.0 (OCH_3), 50.4($C_{\alpha''}$), C_{β} below DMSO signal, 37.0 ($C_{\delta''}$), 37.0 ($C_{\beta'}$), 31.9 (CH_2), 27.7($C_{\beta''}$), 25.3($C_{\gamma''}$). **v_{max} /cm^{-1}:** 3284 (w, N-H), 2934 (w, C-H), 1743 (w, C=O), 1677 (s, C=O), 1546 (s, C=C). **HPLC** (5-100% ACN) *Rt* 8.63 mins. **HRMS** (ESI+) *m/z* found $[M+H]^+$ 487.2423, $C_{22}H_{31}N_8O_5^+$ required 487.2417 (Δ 1.2 ppm). **$[\alpha]_D^{25}$** = -11.0 (*c* 0.59, MeOH).
**B7x**	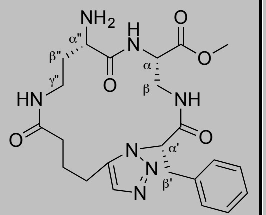	GP3 & GP4<br>99%<br>86%	**R_f** = 0.08 (15% MeOH/ 85% CH_2Cl_2). **δ_H /ppm** (500 MHz, d_6-DMSO, 60 °C): 8.27 (1H, t, *J* = 5.2 Hz, C_{β}-N*H*), 7.94 (1H, app s, C_{α}-N*H*), 7.89 (1H, t, *J*=5.3 Hz, $C_{\gamma''}$-N*H*), 7.37 (1H, s, triazole C*H*), 7.22-7.06 (5H, m, 5 × ArC*H*), 5.38 (1H, dd, *J*=10.5, 5.1 Hz, $H_{\alpha'}$), 4.35 (1H, dd, *J*=5.6, 3.8 Hz, H_{α}), 3.68-3.58 (1H, m, H_{β}), 3.55 (3H, s, OCH_3), 3.56-3.42 (3H, m, $H_{\beta'}$ and $\underline{H}_{\beta}$), 3.33-3.27 (1H, m, $H_{\alpha''}$), $H_{\gamma''}$ below H_2O signal, 2.47-2.30 (2H, m, $COCH_2CH_2CH_2$), 2.26-2.11 (2H, m, $COCH_2CH_2CH_2$), 1.82-1.73 (1H, m, $COCH_2CH_2CH_2$), 1.73-1.64 (2H, m, $COCH_2C\underline{H}_2CH_2$ and $H_{\beta''}$), 1.60-1.50 (1H, m, $\underline{H}_{\beta''}$). **δ_C /ppm** (125 MHz, d_6-DMSO): 174.9 ($C_{\alpha''}$*C*=O), 172.1 (C=$OCH_2CH_2CH_2$), 170.4 (*C*OOMe), 168.0 ($C_{a'}$*C*=O), 138.1 (triazole *C*), 136.6 (Ar*C*), 131.3 (triazole *C*H), 128.9 (Ar*C*H), 128.2 (Ar*C*H), 126.7 (Ar*C*H), 62.1 ($C_{\alpha'}$), 52.7 ($C_{\alpha''}$), 52.2 (C_{α}), 52.1 (OCH_3), C_{β} below DMSO signal, 37.0 ($C_{\beta'}$), 35.6 ($C_{\gamma''}$), 35.0 ($C_{\beta''}$), 34.3 ($COCH_2CH_2CH_2$), 23.5 ($COCH_2CH_2CH_2$), 21.5 ($COCH_2CH_2CH_2$). **v_{max} /cm^{-1}:** 3260 (m, N-H), 2934 (m, C-H), 1740 (m, C=O), 1644 (s, C=O), 1541 (s, C=C). **HPLC** (5-100% ACN) *Rt* 8.98 mins. **HRMS** (ESI+) *m/z* found $[M+H]^+$ 486.2488, $C_{23}H_{32}N_7O_5^+$ required 486.2465 (Δ 4.7 ppm). **$[\alpha]_D^{25}$** = +0.8 (*c* 2.25, MeOH).
**B8x**	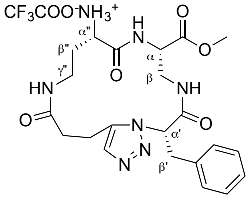	GP3 & GP4<br>60%<br>96%	**δ_H /ppm** (500 MHz, d_6-DMSO, 120 °C): 7.94 (1H, s, N*H*), 7.69 (1H, app s, C_{β}-N*H*), 7.38 (1H, s, triazole C*H*), 7.21-7.11 (3H, m, 3 × ArC*H*), 7.01-6.97 (2H, m, 2 × ArC*H*), 5.36 (1H, dd, *J*=10.4, 4.9 Hz, $H_{\alpha'}$), 4.40 (1H, t, *J*=5.0 Hz, H_{α}), 3.77-3.66 (3H, m, $H_{\alpha''}$ and H_{β}), 3.63 (3H, s, OCH_3), 3.57 (1H,dd, *J*=14.0, 4.9 Hz, $H_{\beta'}$), 3.42 (1H,dd, *J*=14.0, 10.4 Hz, $\underline{H}_{\beta'}$), 3.28-3.20 (1H,m, $H_{\gamma''}$), 3.13-3.02 (1H, m, $\underline{H}_{\gamma''}$), 2.66-2.40 (4H, m, $COCH_2CH_2$ and $COCH_2CH_2$), 1.95-1.86 (1H, m, $H_{\beta''}$), 1.82-1.74 (1H, m, $\underline{H}_{\beta''}$). **δ_C /ppm** (125 MHz, d_6-DMSO):.173.0 (C=OCH_2CH_2), 170.0 (*C*OOMe), 168.1 ($C_{\alpha''}$*C*=O), 168.0 ($C_{a'}$*C*=O), 138.3 (triazole *C*), 136.8 (Ar*C*), 131.0 (triazole *C*H), 128.7 (Ar*C*H), 128.2 (Ar*C*H), 126.6 (Ar*C*H), 61.5 ($C_{\alpha'}$), 52.2 (OCH_3), 51.9 (C_{α}), 50.5 ($C_{\alpha''}$), C_{β} below DMSO signal, 38.3 ($C_{\beta'}$), 34.7 ($C_{\gamma''}$), 33.8 ($COCH_2CH_2$), 31.5 ($C_{\beta''}$), 17.3 ($COCH_2CH_2$). **v_{max} /cm^{-1}:** 3260 (w, N-H), 2950 (w, C-H), 1741 (m, C=O), 1687 (s, C=O), 1551 (m, C=C). **HPLC** (5-100% ACN) *Rt* 8.90 mins. **HRMS** (ESI+) *m/z* found $[M+H]^+$ 472.2301, $C_{22}H_{30}N_7O_5^+$ required 472.2308 (Δ -1.5 ppm). **$[\alpha]_D^{25}$** = +41.0 (*c* 1.3, MeOH).

**B9x**	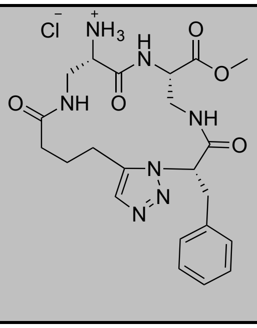	GP3 & GP4<br>99%<br>72%	**HPLC** (5-100% ACN) *Rt* 6.44 mins. **LCMS** $[M+H]^+$ 472.24.
**B10 x**	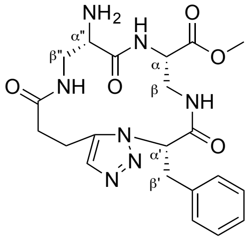	GP3 & GP4<br>89%<br>78%	**R$_f$** = 0.16 (15% MeOH/ 85% CH_2Cl_2). **δ_H /ppm** (400 MHz, d_6-DMSO):. 7.92 (1H, s, C_α-N*H*), 7.86 (1H, dd, *J*=6.3, 5.4 Hz, C_β-N*H*), 7.64 (1H, t, *J* = 6.1 Hz, $C_{\beta''}$-N*H*), 7.48 (1H, s, triazole C*H*), 7.26-7.10 (5H, m, 5 × ArC*H*), 5.25 (1H, dd, *J* = 8.5, 7.0 Hz, $H_{\alpha'}$), 4.26 (1H, app s, H_α), 3.63-3.43 (4H, m, H_β, $H_{\beta'}$ and $H_{\beta''}$), 3.58 (3H, s, OCH_3), $\underline{H}_\beta$ below H_2O signal, 3.26-3.18 (1H, m, $H_{\alpha''}$), 3.10-3.01 (1H, m, $\underline{H}_{\beta''}$), 2.77-2.65 (1H, m, $COCH_2CH_2$), 2.59-2.43 (2H, m, $COCH_2C\underline{H_2}$ and $COCH_2CH_2$), 2.40-2.28 (1H, m, $COC\underline{H}_2CH_2$). **δ_C /ppm** (125 MHz, d_6-DMSO):. 174.1 ($C_{\alpha''}$*C*=O), 171.9 (*C*=OCH_2CH_2), 170.6 (*C*OOMe), 167.5 ($C_{a'}$*C*=O), 137.3 (triazole *C*), 136.8 (Ar*C*), 131.3 (triazole *C*H), 129.0 (Ar*C*H), 128.2 (Ar*C*H), 126.7 (Ar*C*H), 62.1 ($C_{\alpha'}$), 54.1 ($C_{\alpha''}$), 52.4 (OCH_3), 52.0 (C_α), 42.6 $C_{\beta''}$, C_β below DMSO signal, 36.5 ($C_{\beta'}$), 33.8 (COCH_2CH_2), 19.3 ($COCH_2CH_2$). **ν_{max} /cm^{-1}:** 3254 (w, N-H), 2970 (w, C-H), 1739 (w, C=O), 1666 (s, C=O), 1546 (m, C=C). **HPLC** (5-100% ACN) *Rt* 8.75 mins. **HRMS** (ESI+) *m/z* found $[M+H]^+$ 458.2141, $C_{21}H_{28}N_7O_5^+$ required 458.2152 (Δ -2.4 ppm). **$[\alpha]_D^{25}$** = +60.1 (*c* 1.46, MeOH).
**B11 x**	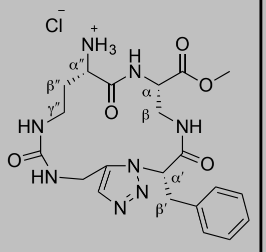	GP3 & GP4<br>52%<br>98%	**Mp** = 221-225 °C (CH_2Cl_2). **δ_H /ppm** (500 MHz, d_6-DMSO): 9.81 (1H, d, *J*=7.0 Hz, C_α-N*H*), 8.45 (1H, t, *J*=5.9 Hz, C_β-N*H*), 8.24 (3H, d, *J*=5.6 Hz, NH_3^+), 7.40 (1H, s, triazole C*H*), 7.21-7.06 (3H, m, 3 × ArC*H*), 7.02-6.91 (2H, m, 2 × ArC*H*), 6.86 (1H, t, *J*=5.0 Hz, N*H*CH_2), 6.67 (1H, t, *J*=6.1 Hz, $C_{\gamma''}$-N*H*), 5.66 (1H, dd, *J*=11.7, 4.1 Hz, $H_{\alpha'}$), 4.42-4.33 (1H, m, H_α), 3.85-3.70 (3H, m, NHCH_2, $H_{\alpha''}$ and H_β), 3.65 (1H, dd, *J*=15.4, 5.0 Hz, NHC$\underline{H_2}$), 3.58 (3H, s, OCH_3), 3.56-3.50 (1H, m, $\underline{H}_\beta$), $H_{\beta'}$ below H_2O signal, 3.37 (1H, dd, *J*=13.6, 11.7 Hz, $\underline{H}_{\beta'}$), 3.33-3.23 (1H, m, $H_{\gamma''}$), 2.98-2.87 (1H, m, $\underline{H}_{\gamma''}$), 1.87-1.76 (1H, m, $H_{\beta''}$), 1.75-1.64 (1H, m, $\underline{H}_{\beta''}$). **δ_C /ppm** (125 MHz, d_6-DMSO): 170.2 (*C*OOMe), 168.3 ($C_{\alpha''}$*C*=O), 168.1 ($C_{\alpha'}$*C*=O), 159.7 (NH*C*=ONH), 137.2 (triazole C), 136.8 (Ar*C*), 132.0 (triazole *C*H), 128.6 (Ar*C*H), 128.2 (Ar*C*H), 126.6 (Ar*C*H), 61.8 ($C_{\alpha'}$), 52.2 (OCH_3), 51.6 (C_α), 50.3 ($C_{\alpha''}$), C_β below DMSO signal, 38.4 ($C_{\beta'}$), 35.6 ($C_{\gamma''}$), 32.8 ($C_{\beta''}$), 31.3 (NHCH_2). **ν_{max} /cm^{-1}:** 3239 (NH str), 2920 (CH str), 1736 (C=O str), 1683 (C=O str), 1648, 1563, 1285, 1214, 1137. **HPLC** (5-100% ACN) *Rt* 8.64 mins. **HRMS** (ESI+) *m/z* found $[M+H]^+$ 473.2264, $C_{21}H_{29}N_8O_5^+$ required 473.2261. **$[\alpha]_D^{25}$** = +40.0 (*c* 0.29, MeOH).

**B12 x**	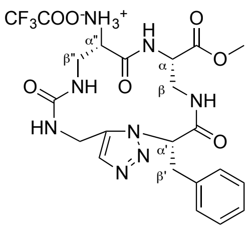	GP3 & GP4<br>40%<br>97%	**δ_H /ppm** (500 MHz, d_6-DMSO, 120 °C): 7.47 (1H, s, triazole C*H*), 7.26-7.11 (3H, m, 3 × ArC*H*), 7.09-6.92 (3H, m, 2 × ArC*H* and C_β-N*H*), 6.52 (1H, app s, CH_2-N*H*), 6.06 (1H, app s, $C_{\beta''}$-N*H*), 5.62 (1H, dd, *J*=10.3, 5.0 Hz, $H_{\alpha'}$), 4.27 (1H, dd, *J*=8.3, 4.1 Hz, H_α), 4.01 (1H, dd, *J*=15.7, 6.9 Hz, C*H_2*), 3.97-3.87 (2H, m, C$\underline{H}_2$ and $H_{\beta''}$), 3.83-3.74 (2H, m, $H_{\alpha''}$ and H_β), 3.60-3.51 (2H, m, $\underline{H}_\beta$ and $H_{\beta'}$), 3.43 (1H, dd, *J*=14.1, 10.3 Hz, $H_{\beta'}$), 3.26-3.18 (2H, m, $H_{\beta''}$). **δ_C /ppm** (125 MHz, d_6-DMSO):. 169.4 (*C*OOMe), 167.4 ($C_{a''}$*C*=O), 166.7 ($C_{a'}$*C*=O), 158.3 (NH*C*=ONH), 136.7 (Ar*C*), 136.5 (triazole *C*), 132.7 (triazole *C*H), 128.7 (Ar*C*H), 128.3 (Ar*C*H), 126.7 (Ar*C*H), 61.2 ($C_{\alpha'}$), 52.8 ($C_{\alpha''}$), 52.6 (C_α), 52.1 (O*C*H_3), 38.1 (C_β), 37. 5 ($C_{\beta'}$), 30.9 (*C*H_2). **v_{max} /cm^{-1}:** 3291 (w, N-H), 2953 (w, C-H), 1736 (w, C=O), 1673 (s, C=O), 1561 (m, C=C). **HPLC** (5-100% ACN) *Rt* 8.52 mins. **HRMS** (ESI+) *m/z* found $[M+H]^+$ 459.2097, $C_{20}H_{27}N_8O_5^+$ required 459.2104 (Δ -1.5 ppm). **$[\alpha]_D^{25}$** = -10.6 (*c* 0.68, MeOH).
**B13 x**	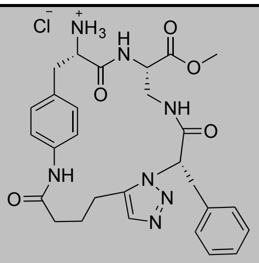	GP3 & GP5<br>15%<br>92%	**HPLC** (5-100% ACN) *Rt* 6.97 mins. **LCMS** $[M+H]^+$ 548.13.
**B14 x**	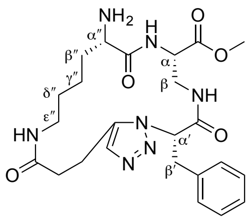	GP3 & GP4<br>90%<br>92%	**R_f** = 0.18 (15% MeOH/ 85% CH_2Cl_2). **Mp** = 140-143 °C (CH_2Cl_2). **δ_H /ppm** (400 MHz, $CDCl_3$): 8.16-7.98 (2H, m, C_β-N*H* and C_α-N*H*), 7.89 (1H, dd, *J*=6.2, 5.3 Hz, $C_{\varepsilon''}$-N*H*), 7.45 (1H, s, triazole C*H*), 7.28-7.10 (5H, m, 5 × ArC*H*), 5.30 (1H, dd, *J*=9.4, 6.1 Hz, $H_{\alpha'}$), 4.37-4.30 (1H, m, H_α), 3.87-3.77 (1H, m, H_β), 3.62-3.41 (2H, m, $H_{\beta'}$), 3.46 (3H, s, OC*H_3*), 3.30-3.23 (1H, m, $H_{\varepsilon''}$), 3.23-3.16 (1H, m, $H_{\alpha''}$), 3.16-3.12 (1H, m, $\underline{H}_\beta$), 3.02-2.84 (1H, m, $\underline{H}_{\varepsilon''}$), 2.73-2.49 (2H, m, $COCH_2CH_2$), 2.41-2.31 (2H, m, $COCH_2CH_2$), 1.55-1.44 (2H, m, $H_{\beta''}$), 1.44-1.26 (2H, m, $H_{\delta''}$), 1.25-1.06 (2H, m, $H_{\gamma''}$). **δ_C /ppm** (125 MHz, $CDCl_3$): 175.0 ($C_{\alpha''}$*C*=O), 170.8 (*C*=OCH_2CH_2), 170.3 (*C*OOMe), 167.6 ($C_{\alpha'}$*C*=O), 137.6 (triazole C), 136.7 (Ar*C*), 130.8 (triazole *C*H), 129.0 (Ar*C*H), 128.3 (Ar*C*H), 126.7 (Ar*C*H), 61.8 ($C_{\alpha'}$), 54.1 ($C_{\alpha''}$), 52.0 (C_α), 51.9 (OCH_3), C_β below solvent signal, 38.0 ($C_{\varepsilon''}$), 36.8 ($C_{\beta'}$), 34.8 ($C_{\beta''}$), 33.5 ($COCH_2CH_2$), 28.8 ($C_{\delta''}$), 21.6 ($C_{\gamma''}$), 18.9 ($COCH_2CH_2$). **v_{max} /cm^{-1}:** 3269 (m, N-H), 2924 (s, C-H), 1744 (m, C=O), 1648 (s, C=O), 1646 (s, C=C), 1546 (s, C=C). **HPLC** (5-100% ACN) *Rt* 9.12 mins. **HRMS** (ESI+) *m/z* found $[M+H]^+$ 500.2614, $C_{24}H_{34}N_7O_5^+$ required 500.2621 (Δ -1.4 ppm). **$[\alpha]_D^{25}$** = +14.3 (*c* 0.43, MeOH).
**C2x**	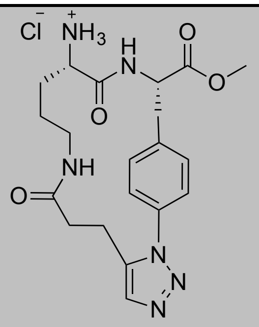	GP3 & GP4<br>62%<br>75%	**HPLC** (5-100% ACN) *Rt* 5.26 mins. **LCMS** $[M+H]^+$ 415.16.

**C3x**	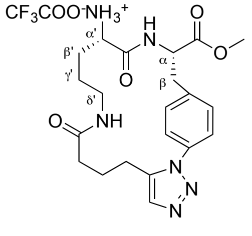	GP3 & GP5<br>97%<br>95%	**δ_H /ppm** (500 Hz, d_6-DMSO, 70 °C): 8.33 (1H, d, *J*=6.5 Hz, C_{α}-N*H*), 8.12 (3H, br s, NH_3^+), 7.68 (1H, s, triazole C*H*), 7.57 (1H, t, *J*=5.7 Hz, $C_{\delta'}$-N*H*), 7.50-7.35 (4H, m, 4 × ArC*H*), 4.53-4.45 (1H, m, H_{α}), 3.82-3.76 (1H, m, $H_{\alpha'}$), 3.66 (3H, s, OCH_3), 3.38 (1H, dd, *J*=14.2, 9.4 Hz, H_{β}), 3.26 (1H, dd, *J*=14.2, 4.1 Hz, $\underline{H}_{\beta}$), 3.06-2.85 (2H, m, $H_{\delta'}$), 2.67-2.54 (2H, m, $COCH_2CH_2CH_2$), 1.99 (2H, t, *J*=6.6 Hz, $COCH_2CH_2CH_2$), 1.73-1.65 (1H, m, $COCH_2CH_2CH_2$), 1.57-1.50 (2H, m, $H_{\beta'}$), 1.39-1.27 (2H, m, $H_{\gamma'}$). **δ_C /ppm** (125 MHz, d_6-DMSO): 171.4 ($C=OCH_2CH_2CH_2$), 170.5 (*C*OOMe), 168.9 ($C_{a'}C=O$), 138. 9 (Ar*C*), 137.9 (triazole *C*), 134.6 (Ar*C*), 132.0 (triazole *C*H), 130.2 (Ar*C*H), 125.5 (Ar*C*H), 54.1 (C_{α}), 52.0 (OCH_3), 51.6 ($C_{\alpha'}$), 37.9 ($C_{\delta'}$), 34.3 (C_{β}), 34.3 ($COCH_2CH_2CH_2$), 28.6 ($C_{\beta'}$), 24.4 ($C_{\gamma'}$), 23.6 ($COCH_2CH_2CH_2$), 21.9 ($COCH_2CH_2CH_2$). **ν_{max} /cm^{-1}:** 3261 (w, N-H), 2945 (w, C-H), 1739 (m, C=O), 1675 (s, C=O), 1546 (s, C=C). **HPLC** (5-100% ACN) *Rt* 5.58 mins. **HRMS** (ESI+) *m/z* found $[M+H]^+$ 429.2224, $C_{21}H_{29}N_6O_4^+$ required 429.2245 (Δ -4.9 ppm). **$[\alpha]_D^{25}$** = -29.2 (*c* 0.43, MeOH).
**C5x**	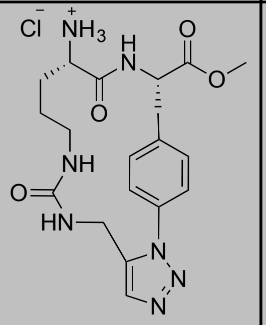	GP3 & GP5<br>46%<br>89%	**HPLC** (5-100% ACN) *Rt* 5.24 mins. **LCMS** $[M+H]^+$ 416.16.
**C7x**	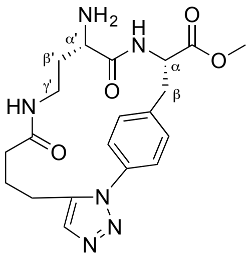	GP3 & GP5<br>97%<br>95%	**R_f** = 0.11 (15% MeOH/ 85% CH_2Cl_2). **δ_H /ppm** (400 MHz, d_6-DMSO): 8.18 (1H, d, *J*=9.5 Hz, C_{α}-NH), 7.87 (1H, t, *J*=5.9 Hz, $C_{\gamma'}$-N*H*), 7.77 (1H, s, triazole C*H*), 7.43 (2H, d, *J*=8.5 Hz, 2 × ArC*H*), 7.35 (2H, d, *J*=8.5 Hz, 2 × ArC*H*), 4.83-4.73 (1H, m, H_{α}), 3.72 (3H, s, OCH_3), H_{β} below H_2O signal, 3.08 (1H, dd, *J*=13.5, 12.3 Hz, $\underline{H}_{\beta}$), 3.03-2.84 (3H, m, $H_{\alpha'}$ and $H_{\gamma'}$), 2.57-2.33 (2H, m, $COCH_2CH_2CH_2$), 2.08 (2H, t, *J*=6.1 Hz, $COCH_2CH_2CH_2$), 2.03-1.80 (2H, m, $COCH_2CH_2CH_2$), 1.34-1.08 (1H, m, $H_{\beta'}$), 0.93-0.70 (1H, m, $\underline{H}_{\beta'}$). **δ_C /ppm** (125 MHz, d_6-DMSO): 174.8 ($C_{a'}C=O$), 171.9 (*C*OOMe), 171.1($C=OCH_2CH_2CH_2$), 138.8 (Ar*C*), 137.7 (triazole *C*), 134.1 (Ar*C*), 131.6 (triazole *C*H), 130.5 (Ar*C*H), 124.6 (Ar*C*H), 54.2 ($C_{\alpha'}$), 52.1 (OCH_3), 51.5 (C_{α}), 36.6 ($C_{\gamma'}$), 36.1 ($C_{\beta'}$), 35.9 (C_{β}), 34.5 ($COCH_2CH_2CH_2$), 24.3 ($COCH_2CH_2CH_2$), 21.78 ($COCH_2CH_2CH_2$). **ν_{max} /cm^{-1}:** 3274 (m, N-H), 2944 (w, C-H), 1742 (s, C=O), 1642 (s, C=O), 1541 (s, C=C). **HPLC** (5-100% ACN) *Rt* 5.70 mins. **HRMS** (ESI+) *m/z* found $[M+H]^+$ 415.2072, $C_{20}H_{27}N_6O_4^+$ required 415.2088 (Δ -3.9 ppm). **$[\alpha]_D^{25}$** = -8.8 (*c* 0.65, MeOH).

**C8x**	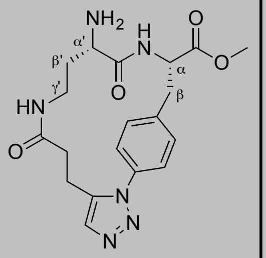	GP3 & GP5<br><br>83%<br><br>81%	**R_f** = 0.15 (15% MeOH/ 85% CH_2Cl_2). **δ_H /ppm** (500 Hz, *d_6*-DMSO, 70 °C): 7.66 (1H, s, triazole C*H*), 7.55-7.29 (5H, m, N*H* and 4 × ArC*H*), 7.05 (1H, s, N*H*), 4.84 (1H, dd, *J*=11.8, 3.6 Hz, H_α), 3.75 (3H, s, OCH_3), 3.33 (1H, dd, *J* =14.2, 3.6 Hz, H_β), $\underline{H}_\beta$, $H_{\alpha'}$ and COCH_2CH_2 below H_2O signal, $H_{\gamma'}$ below DMSO signal, 2.24-2.08 (1H, m, COCH_2CH_2), 2.08-1.89 (1H, m, COCH_2CH_2), 1.44-1.31 (1H, m, $H_{\beta'}$), 1.31-1.13 (1H, m, $\underline{H}_{\beta'}$). **δ_C /ppm** (125 MHz, *d_6*-DMSO): 175.3 ($C_{a'}$*C*=O), 172.4 (*C*OOMe), 170.6 (*C*=OCH_2CH_2), 138.9 (Ar*C*), 138.3 (triazole *C*), 135. 3 (Ar*C*), 133. 6 (triazole *C*H), 130.4 (Ar*C*H), 125.5 (Ar*C*H), 54.6 ($C_{a'}$), 52.6 (OCH_3), 52.0 (C_α), 36.7 (COCH_2CH_2), 36.5 (C_β), 35.8 ($C_{\gamma'}$), 34.4 ($C_{\beta'}$), 20.3 (COCH_2CH_2). **v_{max} /cm^{-1}:** 3312 (m, N-H), 2926 (m, C-H), 1749 (s, C=O), 1630 (s, C=O), 1538 (s, C=C). **HPLC** (5-100% ACN) *Rt* 5.23 mins. **HRMS** (ESI+) *m/z* found $[M+H]^+$ 401.1915, $C_{19}H_{25}N_6O_4^+$ required 401.1932 (Δ -4.2 ppm). **$[\alpha]_D^{25}$** = -39.5 (*c* 0.34, MeOH).
**C10 x**	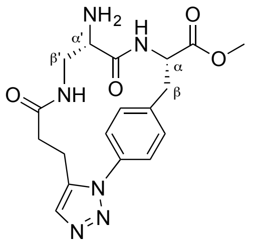	GP3 & GP5<br><br>33%<br><br>83%	**R_f** = 0.50 (15% MeOH/ 85% CH_2Cl_2). **δ_H /ppm** (500 Hz, *d_6*-DMSO, 70 °C): 7.65 (1H, s, triazole C*H*), 7.55 (1H, s, C_α-N*H*), 7.51-7.34 (4H, m, 4 × ArC*H*), 6.55 (1H, s, $C_{\beta'}$-N*H*), 4.73 (1H, dd, *J*=12.3, 4.1 Hz, H_α), 3.75 (3H, s, OCH_3), 3.39 (1H, dd, *J*=13.5, 4.1 Hz, H_β), 3.30-3.24 (2H, m, $H_{\alpha'}$ and $H_{\beta'}$), 3.21-3.15 (1H, m, $\underline{H}_{\beta'}$), $\underline{H}_\beta$ below H_2O signal, 2.95-2.87 (2H, m, COCH_2CH_2), 2.10-1.98 (1H, m, COCH_2CH_2), 1.86-1.77 (1H, m, COC$\underline{H}_2$CH_2). **δ_C /ppm** (125 MHz, *d_6*-DMSO): 172.7 ($C_{a'}$*C*=O), 171.9 (*C*OOMe), 171.9 (*C*=OCH_2CH_2), 139.4 (Ar*C*), 137.9 (triazole *C*), 134.3 (Ar*C*), 132.3 (triazole *C*H), 131.6 (Ar*C*H), 130.0 (Ar*C*H), 126.3 (Ar*C*H), 124.8 (Ar*C*H), 55.0 ($C_{a'}$), 52.6 (C_α), 52.3 (OCH_3), 42.4 ($C_{\beta'}$), 35.8 (C_β), 33.1 (COCH_2CH_2), 17.9 (COC$H_2$$CH_2$). **$v_{max}$ /cm^{-1}:** 3307 (w, N-H), 2924 (w, C-H), 1745 (m, C=O), 1649 (s, C=O), 1518 (s, C=C). **HPLC** (5-100% ACN) *Rt* 5.36 mins. **HRMS** (ESI+) *m/z* found $[M+H]^+$ 387.1763, $C_{18}H_{23}N_6O_4^+$ required 387.1781 (Δ -4.6 ppm). **$[\alpha]_D^{25}$** = -22.0 (*c* 0.13, MeOH).
**C11 x**	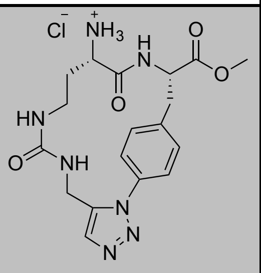	GP3 & GP5<br><br>40%<br><br>82%	**HPLC** (5-100% ACN) *Rt* 5.18 mins. **LCMS** $[M+H]^+$ 402.14.
**C12 x**	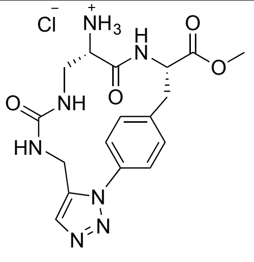	GP3 & GP5<br><br>27%<br><br>91%	**HPLC** (5-100% ACN) *Rt* 5.21 mins. **LCMS** $[M+H]^+$ 388.12.

**C14x**	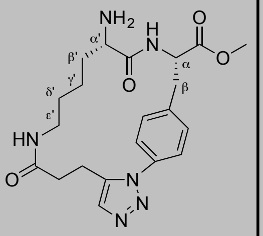	GP3 & GP5<br>68%<br>98%	**R_f** = 0.24 (15% MeOH/ 85% CH_2Cl_2). **δ_H /ppm** (500 Hz, d_6-DMSO): 8.20-8.10 (1H, m, C_α-N*H*), 7.68 (1H, s, triazole C*H*), 7.56 (1H, t, *J*=5.6 Hz, $C_{\varepsilon'}$-N*H*), 7.44-7.31 (5H, m, 5 × ArC*H*), 4.63-4.49 (1H, m, H_α), 3.64 (3H, s, OCH_3), H_β below H_2O signal, 3.08-2.92 (3H, m, $H_{\alpha'}$, $\underline{H}_\beta$ and $H_{\varepsilon'}$), 2.87-2.73 (3H, m, $\underline{H}_{\varepsilon'}$ and $COCH_2CH_2$), 2.22-2.03 (2H, m, $COCH_2CH_2$), 1.40-1.13 (4H, m, $H_{\beta'}$ and $H_{\delta'}$), 1.13-0.90 (2H, m, $H_{\gamma'}$). **δ_C /ppm** (125 MHz, d_6-DMSO): 174.8 ($C_{a'}$*C*=O), 172.0 (*C*OOMe), 170.2 (*C*=OCH_2CH_2), 139.2 (Ar*C*), 137.4 (triazole *C*), 134.3 (Ar*C*), 132.4 (triazole *C*H), 130.2 (Ar*C*H), 125.0 (Ar*C*H), 54.9 ($C_{a'}$), 52.5 (C_α), 52.2 (OCH_3), 38.5 ($C_{\varepsilon'}$), 35.8 (C_β), 34.4 ($C_{\beta'}$), 34.3 ($COCH_2CH_2$), 28.3 ($C_{\delta'}$), 22.4 ($C_{\gamma'}$), 20.2 ($COCH_2CH_2$). **v_{max} /cm^{-1}:** 3293 (m, N-H), 2922 (m, C-H), 1738 (m, C=O), 1648 (s, C=O), 1518 (s, C=C). **HPLC** (5-100% ACN) *Rt* 5.35 mins. **HRMS** (ESI+) *m/z* found $[M+H]^+$ 429.2233, $C_{21}H_{29}N_6O_4^+$ required 429.2250 (Δ -4.0 ppm). **$[\alpha]_D^{25}$** = -9.7 (*c* 0.32, MeOH).
**D1x**	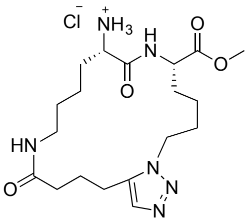	GP3 & GP4<br>65%<br>99%	**δ_H /ppm** (500 MHz, d_6-DMSO): 8.77 (1H, d, *J*=5.3 Hz), 8.21 (3H, d, *J*=5.3 Hz), 7.88 (1H, t, *J*=5.6 Hz), 7.51 (1H, s), 4.33-4.14 (2H, m), 4.13-4.02 (1H, m), 3.84-3.74 (1H, m), 3.53 (3H, s), 3.15-3.03 (2H, m), 2.61 (2H, t, *J*=8.0 Hz), 2.23-2.08 (2H, m), 1.89-1.61 (8H, m), 1.51-1.25 (5H, m), 1.11-0.96 (1H, m). **δ_C /ppm** (125 MHz, d_6-DMSO): 171.7, 171.2, 169.0, 137.4, 131.4, 52.7, 51.8, 51.8, 46.4, 37.7, 34.2, 30.6, 29.6, 29.3, 28.3, 24.5, 21.8, 21.6, 21.1. **v_{max} /cm^{-1}:** 3228, 2925, 1734, 1679, 1636, 1551, 1457, 1207. **HPLC** (5-30% ACN) *Rt* 6.96 mins. **HRMS** (ESI+) *m/z* found $[M+H]^+$ 409.2567, $C_{19}H_{33}N_6O_4^+$ required 409.2563.
**D2x**	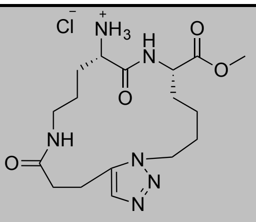	GP3 & GP4<br>82%<br>78%	**HPLC** (5-100% ACN) *Rt* 4.55 mins. **LCMS** $[M+H]^+$ 381.30.
**D3x**	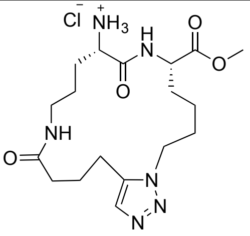	GP3 & GP4<br>99%<br>86%	**HPLC** (5-60% ACN) *Rt* 5.06 mins. **LCMS** $[M+H]^+$ 395.23.
**D4x**	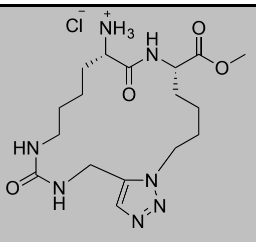	GP3 & GP4<br>99%<br>95%	**HPLC** (5-100% ACN) *Rt* 4.27 mins. **LCMS** $[M+H]^+$ 396.22.

**D5x**	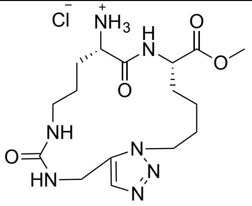	GP3 & GP4<br>99%<br>90%	**HPLC** (5-100% ACN) *Rt* 4.30 mins. **LCMS** $[M+H]^+$ 382.20.
**D6x**	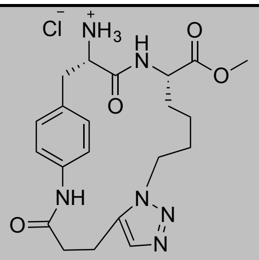	GP3 & GP5<br>71%<br>66%	**HPLC** (5-100% ACN) *Rt* 5.22 mins. **LCMS** $[M+H]^+$ 429.13.
**D7x**	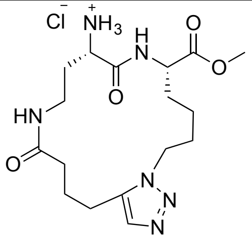	GP3 & GP4<br>99%<br>72%	**HPLC** (5-100% ACN) *Rt* 5.31 mins. **LCMS** $[M+H]^+$ 381.24.
**D8x**	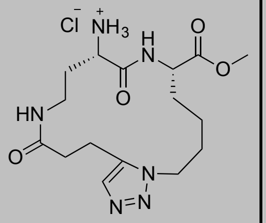	GP3 & GP4<br>99%<br>97%	**δ_H /ppm** (500 MHz, d_6-DMSO): 9.16 (1H, d, *J*=6.4 Hz), 8.45 (1H, t, *J*=5.9 Hz), 8.22 (3H, d, *J*=5.6 Hz), 7.55 (1H, s), 4.42-4.35 (1H, m), 4.24-4.17 (1H, m), 3.99 (1H, m), 3.59 (3H, s), 3.46 (1H, m), 3.42 (1H, m), 3.00 (1H, m), 2.97-2.86 (1H, m), 2.89-2.79 (1H, m), 2.63-2.53 (2H, m), 2.03-1.94 (1H, m), 1.94-1.87 (1H, m), 1.83-1.70 (3H, m), 1.68-1.62 (1H, m), 1.31-1.19 (1H, m), 0.93-0.85 (1H, m). **δ_C /ppm** (125 MHz, d_6-DMSO): 172.6, 171.6, 167.6, 137.1, 131.8, 52.8, 52.1, 50.0, 46.6, 34.5, 34.0, 31.3, 29.0, 27.7, 21.8, 18.3. **v_{max} /cm^{-1}:** 3335, 2925, 1728, 1635, 1562, 1441, 1226. **HPLC** (5-100% ACN) *Rt* 5.36 mins. **LCMS** $[M+H]^+$ 367.28.
**D9x**	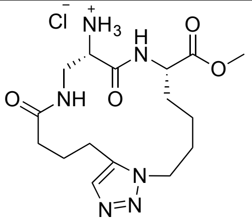	GP3 & GP4<br>99%<br>71%	**HPLC** (5-100% ACN) *Rt* 4.60 mins. **LCMS** $[M+H]^+$ 367.21.
**D10x**	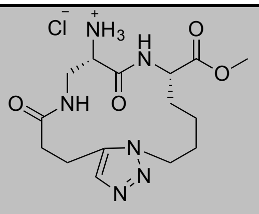	GP3 & GP4<br>88%<br>71%	**HPLC** (5-100% ACN) *Rt* 4.63 mins. **LCMS** $[M+H]^+$ 353.19.

**D11x**	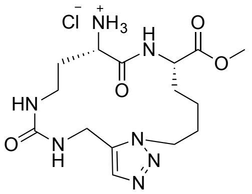	GP3 & GP4<br>99%<br>92%	**δ_H /ppm** (500 MHz, d_6-DMSO): 9.51 (1H, d, *J*=6.5 Hz), 8.25 (3H, d, *J*=4.5 Hz), 7.58 (1H, s), 7.08 (1H, t, *J*=5.5 Hz), 6.80 (1H, t, *J*=5.5 Hz), 4.56 (1H, td, *J*=14.0, 7.0 Hz), 4.38 (1H, dd, *J*=15.5, 6.0 Hz), 4.21-4.08 (2H, m), 4.07-4.01 (1H, m), 3.61 (3H, s), 3.55-3.31 (2H, m), 3.16-3.03 (1H, m), 2.24-2.08 (1H, m), 2.03-1.57 (5H, m), 1.46-1.25 (1H, m), 1.18-1.08 (1H, m). **δ_C /ppm** (125 MHz, d_6-DMSO): 171.7, 167.7, 159.1, 60.1, 52.7, 51.9, 49.9, 46.1, 35.4, 31.9, 31.5, 28.9, 28.4, 27.6, 21.6. **HPLC** (5-100% ACN) *Rt* 5.13 mins. **HRMS** (ESI+) *m/z* found $[M+H]^+$ 368.2055, $C_{15}H_{26}N_7O_4^+$ required 368.2046. **$[\alpha]_D^{25}$** = -4.5 (*c* 0.40, MeOH).
**D12x**	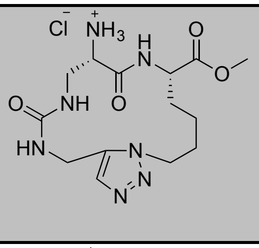	GP3 & GP4<br>73%<br>97%	**HPLC** (5-100% ACN) *Rt* 6.37 mins. **LCMS** $[M+H]^+$ 354.09.
**D13x**	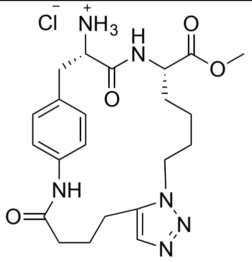	GP3 & GP5<br>66%<br>98%	**HPLC** (5-100% ACN) *Rt* 5.69 mins. **LCMS** $[M+H]^+$ 443.15.
**D14x**	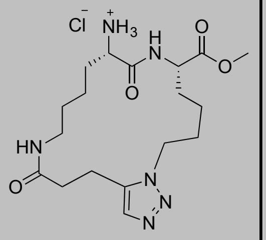	GP3 & GP4<br>37%<br>95%	**δ_H /ppm** (500 MHz, d_6-DMSO): 8.84 (1H, d, *J*=5.4 Hz), 8.19 (3H, d, *J*=4.7 Hz), 7.99 (1H, t, *J*=5.6 Hz), 7.48 (1H, s), 4.38-4.18 (2H, m), 4.08-3.96 (1H, m), 3.80-3.72 (1H, m), 3.52 (3H, s), 3.21-3.10 (1H, m), 3.09-2.98 (1H, m), 2.95-2.78 (2H, m), 2.56-2.44 (1H, m), 2.40-2.28 (1H, m), 1.87-1.62 (6H, m), 1.49-1.15 (5H, m), 1.06-0.93 (1H, m). **δ_C /ppm** (125 MHz, d_6-DMSO): 171.4, 170.6, 168.8, 137.0, 131.4, 52.8, 51.9, 51.7, 46.4, 37.6, 34.1, 30.5, 29.4, 29.0, 28.0, 21.9, 20.9, 18.9. **v_{max} /cm^{-1}:** 3233, 2924, 2857, 1732, 1645, 1554, 1439, 1209. **HPLC** (5-80% ACN) *Rt* 4.77 mins. **HRMS** (ESI+) *m/z* found $[M+H]^+$ 395.2393, $C_{18}H_{31}N_6O_4^+$ required 395.2407.
**E1x**	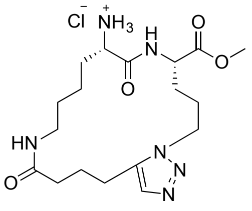	GP3 & GP4<br>99%<br>99%	**δ_H /ppm** (500 MHz, d_6-DMSO): 8.92 (1H, d, *J*=4.9 Hz), 8.23 (3H, d, *J*=5.5 Hz), 8.00 (1H, t, *J*=5.5 Hz), 7.51 (1H, s), 4.33-4.16 (2H, m), 4.10-4.03 (1H, m), 3.84-3.75 (1H, m), 3.59 (3H, s), 3.17-3.07 (1H, m), 3.07-2.98 (1H, m), 2.72-2.53 (2H, m), 2.26-2.07 (1H, m), 2.02-1.59 (8H, m), 1.52-1.16 (4H, m). **δ_C /ppm** (125 MHz, d_6-DMSO): 171.8, 171.1, 169.0, 137.0, 131.7, 52.6, 52.0, 51.8, 46.8, 37.7, 34.6, 30.5, 28.1, 26.7, 25.7, 24.2, 21.8, 21.3. **v_{max} /cm^{-1}:** 3367, 2924, 1731, 1633, 1562, 1448, 1224, 1066. **HPLC** (5-100% ACN) *Rt* 4.58 mins. **LCMS** $[M+H]^+$ 395.24.
**E2x**	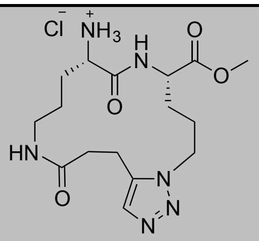	GP3 & GP4<br>99%<br>78%	**HPLC** (5-100% ACN) *Rt* 4.25 mins. **LCMS** $[M+H]^+$ 367.28.

**E3x**	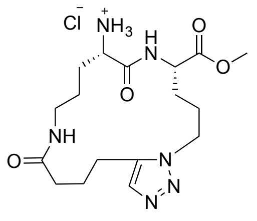	GP3 & GP4<br>99%<br>98%	**δ_H /ppm** (500 MHz, d_6-DMSO): 8.96 (1H, d, *J*=5.2 Hz), 8.26- 8.18 (4H, m), 7.52 (1H, s), 4.31-4.18 (2H, m), 4.09-4.02 (1H, m), 3.89-3.82 (1H, m), 3.60 (3H, s), 3.19-3.06 (1H, m), 3.06-2.94 (1H, m), 2.61 (2H, t, *J*=7.8 Hz), 2.25-2.09 (2H, m), 1.94-1.82 (4H, m), 1.79-1.58 (4H, m), 1.58-1.48 (2H, m). **δ_C /ppm** (125 MHz, d_6-DMSO): 172.1, 170.9, 169.0, 136.7, 131.5, 52.2, 52.0, 51.5, 46.8, 37.3, 34.3, 34.2, 28.6, 26.1, 26.1, 24.8, 23.5, 21.5. **v_{max} /cm^{-1}:** 3360, 2952, 1636, 1560, 1445, 1221. **HPLC** (5-100% ACN) *Rt* 4.39 mins. **HRMS** (ESI+) *m/z* found $[M+H]^+$ 381.2239, $C_{17}H_{29}N_6O_4^+$ required 381.2245.
**E4x**	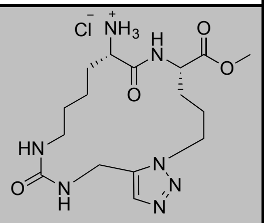	GP3 & GP4<br>99%<br>89%	**HPLC** (5-100% ACN) *Rt* 3.90 mins. **LCMS** $[M+H]^+$ 382.20.
**E5x**	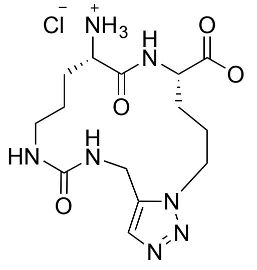	GP3 & GP4<br>99%<br>90%	**HPLC** (5-100% ACN) *Rt* 3.96 mins. **LCMS** $[M+H]^+$ 368.18.
**E6x**	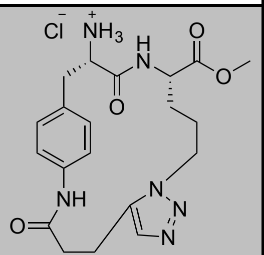	GP3 & GP5<br>44%<br>93%	**v_{max} /cm^{-1}:** 3388, 2920, 2851, 1926, 1719, 1672, 1605, 1572, 1541, 1445, 1418, 1345, 1321, 1297, 1263, 1228, 1173, 1145, 1118, 1011. **HPLC** (5-100% ACN) *Rt* 4.89 mins. **HRMS** (ESI+) *m/z* found $[M+H]^+$ 415.2104, $C_{20}H_{27}N_6O_4^+$ required 415.2094.
**E7x**	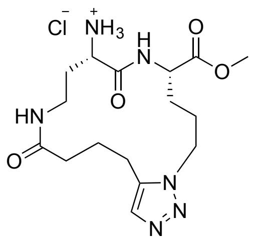	GP3 & GP4<br>99%<br>95%	**δ_H /ppm** (500 MHz, d_6-DMSO): 9.15 (1H, *J*=5.6 Hz), 8.35-8.26 (4H, m), 7.51 (1H, s), 4.33-4.22 (2H, m), 4.18-4.10 (1H, m), 4.01-3.93 (1H, m), 3.61 (3H, s), 3.38-3.23 (1H, m), 2.79-2.65 (1H, m), 2.64-2.49 (1H, m), 2.37-2.13 (2H, m), 2.06-1.69 (7H, m), 1.63-1.49 (1H, m). **δ_C /ppm** (125 MHz, d_6-DMSO): 172.7, 171.06, 168.57, 136.9, 131.5, 52.1, 52.1, 50.2, 46.9, 34.5, 34.3, 31.5, 26.2, 25.8, 24.1, 21.6. **v_{max} /cm^{-1}:** 3344, 2924, 1728, 1635, 1559, 1443, 1231, 1076. **HPLC** (5-100% ACN) *Rt* 4.23 mins. **LCMS** $[M+H]^+$ 367.28.
**E8x**	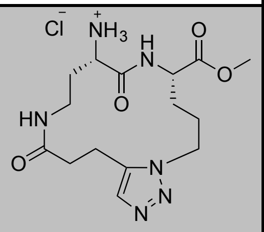	GP3 & GP4<br>82%<br>75%	**HPLC** (5-100% ACN) *Rt* 4.23 mins. **LCMS** $[M+H]^+$ 353.27.

**E9x**	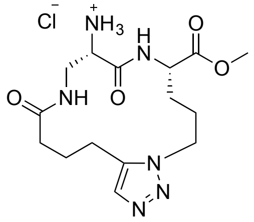	GP3 & GP4<br>99%<br>97%	**HPLC** (5-100% ACN) *Rt* 4.36 mins. **LCMS** $[M+H]^+$ 353.19.
**E10 x**	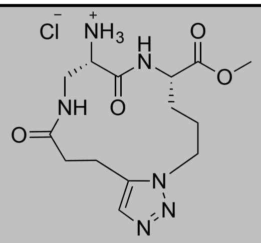	GP3 & GP4<br>99%<br>71%	**HPLC** (5-100% ACN) *Rt* 3.86 mins. **LCMS** $[M+H]^+$ 339.10.
**E11 x**	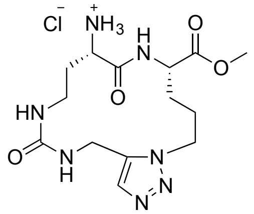	GP3 & GP4<br>99%<br>97%	**HPLC** (5-100% ACN) *Rt* 3.65 mins. **LCMS** $[M+H]^+$ 354.24.
**E12 x**	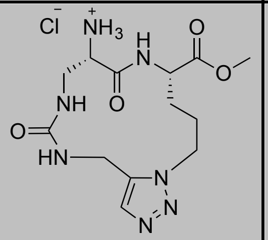	GP3 & GP4<br>80%<br>78%	**HPLC** (5-100% ACN) *Rt* 3.85 mins. **LCMS** $[M+H]^+$ 340.08.
**E13 x**	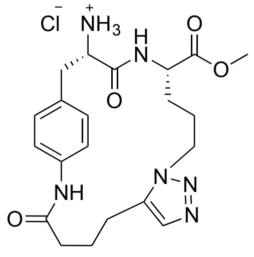	GP3 & GP5<br>68%<br>95%	**HPLC** (5-100% ACN) *Rt* 5.37 mins. **LCMS** $[M+H]^+$ 429.13.
**E14 x**	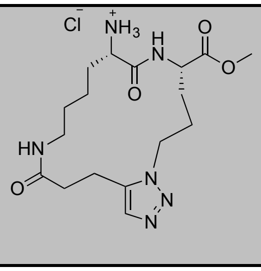	GP3 & GP4<br>99%<br>90%	**HPLC** (5-100% ACN) *Rt* 4.27 mins. **LCMS** $[M+H]^+$ 381.22.

**F2x**	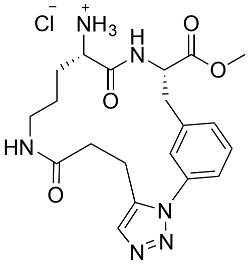	GP3 & GP5<br><br>76%<br><br>90%	**v_{max} /cm^{-1}:** 3232, 3054, 2937, 2112, 1737, 1678, 1643, 1543, 1493. **HPLC** (5-100% ACN) *Rt* 5.72 mins. **HRMS** (ESI+) *m/z* found $[M+H]^+$ 415.2079, $C_{20}H_{27}N_6O_4^+$ required 415.2088.
**F3x**	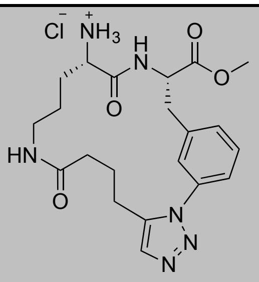	GP3 & GP%<br><br>68%<br><br>86%	**HPLC** (5-100% ACN) *Rt* 5.78 mins. **LCMS** $[M+H]^+$ 429.16.
**F14 x**	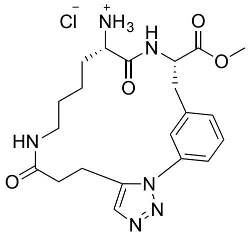	GP3 & GP5<br><br>61%<br><br>89%	**HPLC** (5-100% ACN) *Rt* 5.72 mins. **LCMS** $[M+H]^+$ 429.16.

14. Preparation of B/C/C/P and B/C/C/C/P RuAAC Macrocycles

	Compound	Method, Yield (%), Purity (%)	Analysis
**G1x**	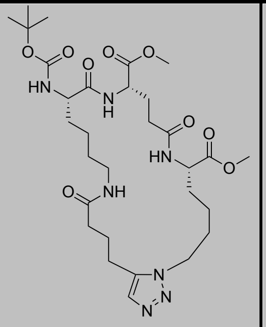	GP3<br>24%<br>85%	**HPLC** (5-100% ACN) *Rt* 7.70 mins. **LCMS** $[M+H]^+$ 652.54.
**G2x**	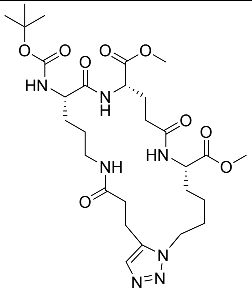	GP3<br>65%<br>91%	**HPLC** (5-100% ACN) *Rt* 7.43 mins. **LCMS** $[M+H]^+$ 624.48.
**G3x**	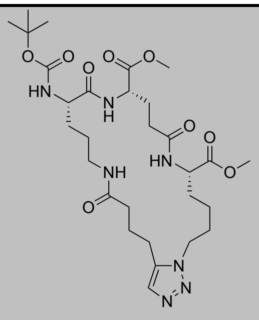	GP3<br>29%<br>88%	**HPLC** (5-100% ACN) *Rt* 7.71 mins. **LCMS** $[M+H]^+$ 638.21.
**G4x**	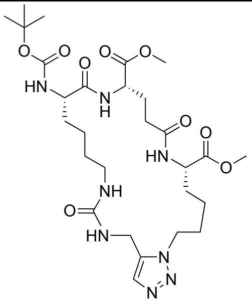	GP3<br>54%<br>85%	**HPLC** (5-100% ACN) *Rt* 7.65 mins. **LCMS** $[M+H]^+$ 639.41.

**G5x**	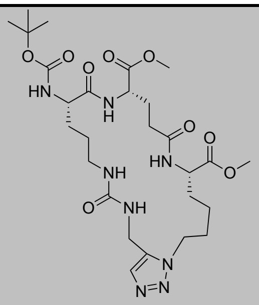	GP3<br>46%<br>72%	**HPLC** (5-45% ACN) *Rt* 11.57 mins. **LCMS** $[M+H]^+$ 625.46.
**G6x**	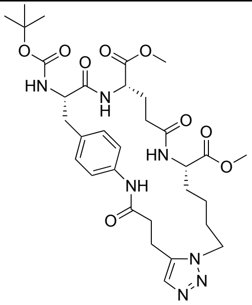	GP3<br>62%<br>74%	**HPLC** (5-100% ACN) *Rt* 8.51 mins. **LCMS** $[M+H]^+$ 672.41.
**G7x**	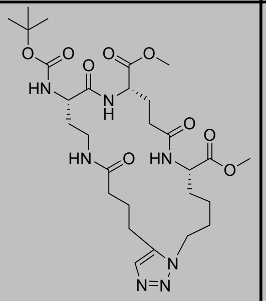	GP3<br>68%<br>94%	**HPLC** (5-100% ACN) *Rt* 7.41 mins. **LCMS** $[M+H]^+$ 624.48.
**G8x**	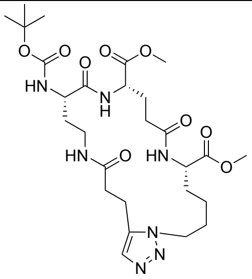	GP3<br>68%<br>92%	**HPLC** (5-100% ACN) *Rt* 7.32 mins. **LCMS** $[M+H]^+$ 610.46.
**G9x**	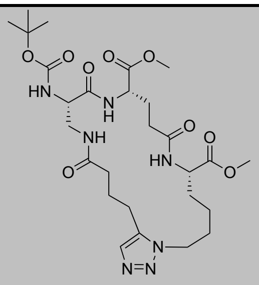	GP3<br>84%<br>93%	**HPLC** (5-45% ACN) *Rt* 12.24 mins. **LCMS** $[M+H]^+$ 610.46.

**G10 x**	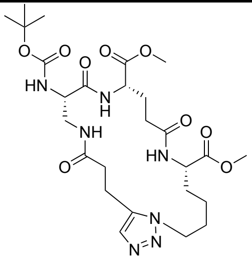	GP3<br>82%<br>91%	**HPLC** (5-45% ACN) *Rt* 12.00 mins. **LCMS** $[M+H]^+$ 596.36.
**G11 x**	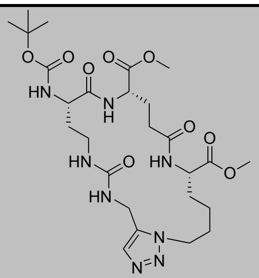	GP3<br>54%<br>72%	**HPLC** (15-30% ACN) *Rt* 10.87 mins. **LCMS** $[M+H]^+$ 611.51.
**G12 x**	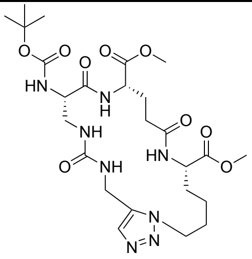	GP3<br>99%<br>87%	**HPLC** (15-30% ACN) *Rt* 11.45 mins. **LCMS** $[M+H]^+$ 597.41.
**G13 x**	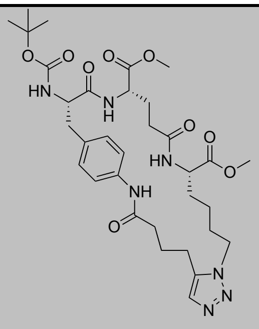	GP3<br>42%<br>90%	**HPLC** (5-100% ACN) *Rt* 8.69 mins. **LCMS** $[M+H]^+$ 686.29.
**G14 x**	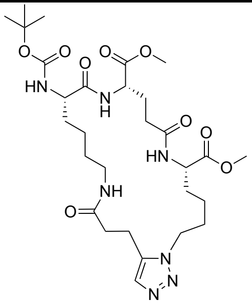	GP3<br>26%<br>75%	**HPLC** (5-100% ACN) *Rt* 7.86 mins. **LCMS** $[M+H]^+$ 638.28.

**H1x**	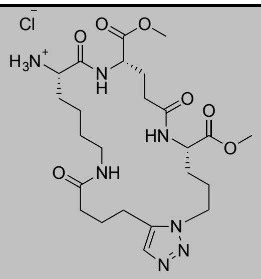	GP3 & GP4<br>87%<br>87%	**HPLC** (5-100% ACN) *Rt* 5.19 mins. **LCMS** $[M+H]^+$ 538.30.
**H2x**	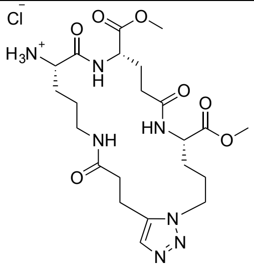	GP3 & GP4<br>83%<br>89%	**HPLC** (5-100% ACN) *Rt* 4.92 mins. **LCMS** $[M+H]^+$ 496.16.
**H3x**	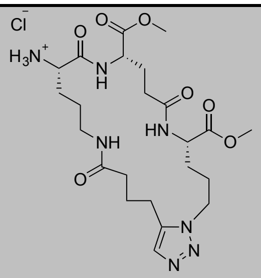	GP3 & GP4<br>44%<br>81%	**HPLC** (5-100% ACN) *Rt* 5.08 mins. **LCMS** $[M+H]^+$ 524.36.
**H4x**	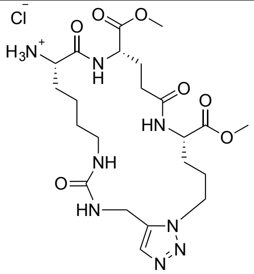	GP3 & GP4<br>75%<br>62%	**HPLC** (5-100% ACN) *Rt* 4.90 mins. **LCMS** $[M+H]^+$ 525.18.
**H5x**	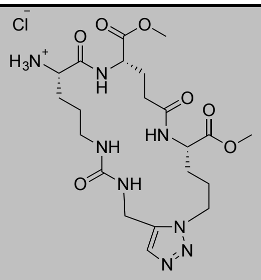	GP3 & GP4<br>26%<br>68%	**HPLC** (5-100% ACN) *Rt* 4.69 mins. **LCMS** $[M+H]^+$ 511.31.

**H6x**	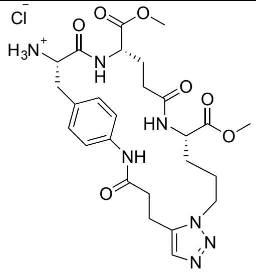	GP3 & GP5<br>61%<br>68%	**HPLC** (5-100% ACN) *Rt* 5.63 mins. **LCMS** $[M+H]^+$ 558.18.
**H7x**	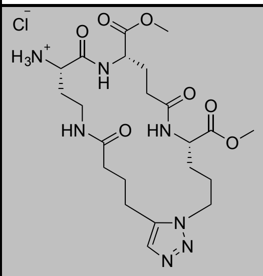	GP3 & GP4<br>64%<br>96%	**HPLC** (5-100% ACN) *Rt* 5.23 mins. **LCMS** $[M+H]^+$ 510.26.
**H8x**	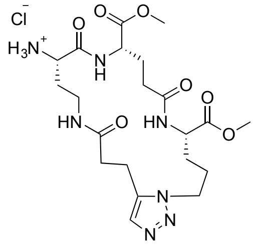	GP3 & GP4<br>52%<br>88%	**HPLC** (5-100% ACN) *Rt* 4.99 mins. **LCMS** $[M+H]^+$ 496.23.
**H9x**	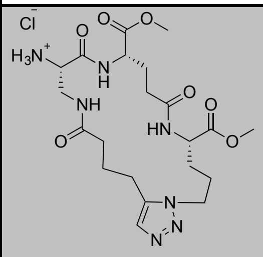	GP3 & GP4<br>65%<br>67%	**HPLC** (5-100% ACN) *Rt* 4.97 mins. **LCMS** $[M+H]^+$ 496.08.
**H10x**	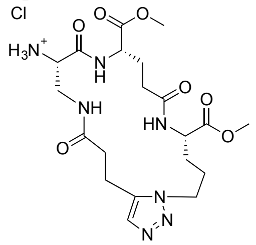	GP3 & GP4<br>86%<br>66%	**HPLC** (5-100% ACN) *Rt* 4.78 mins. **LCMS** $[M+H]^+$ 482.14.
**H11x**	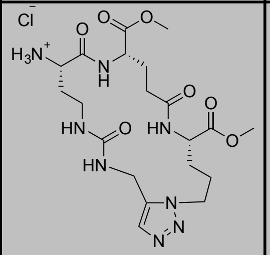	GP3 & GP4<br>35%<br>69%	**HPLC** (5-100% ACN) *Rt* 4.89 mins. **LCMS** $[M+H]^+$ 497.21.

**H12 x**	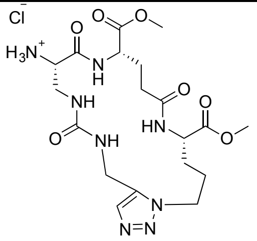	GP3 & GP4<br>57%<br>78%	**HPLC** (5-100% ACN) *Rt* 4.59 mins. **LCMS** $[M+H]^+$ 483.19.
**H14 x**	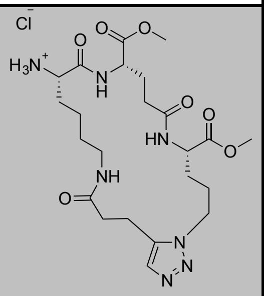	GP3 & GP4<br>86%<br>77%	**HPLC** (5-30% ACN) *Rt* 7.46 mins. **LCMS** $[M+H]^+$ 524.28.
**J13 x**	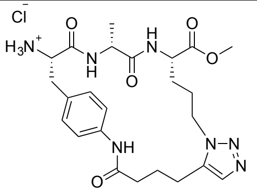	GP3 & GP5<br>45%<br>88%	**HPLC** (5-100% ACN) *Rt* 5.36 mins. **LCMS** $[M+H]^+$ 500.28.
**K3x**	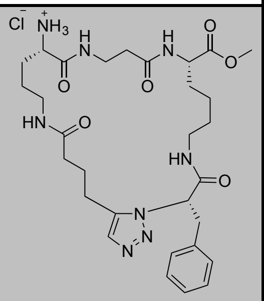	GP3 & GP4<br>48%<br>83%	**HPLC** (5-100% ACN) *Rt* 6.89 mins. **LCMS** $[M+H]^+$ 613.32.
**K8x**	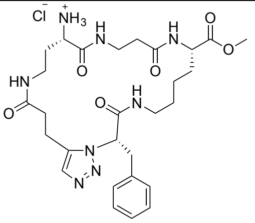	GP3 & GP4<br>46%<br>80%	**HPLC** (5-100% ACN) *Rt* 6.85 mins. **LCMS** $[M+H]^+$ 585.33.
**L7x**	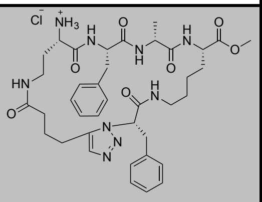	GP3<br>43%<br>92%	**HPLC** (5-100% ACN) *Rt* 10.17 mins. **LCMS** $[M+H]^+$ 846.59.

**L9x**	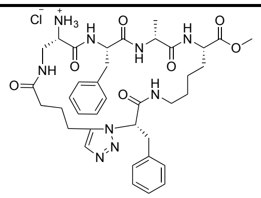	GP3<br>43%<br>89%	**HPLC** (5-100% ACN) *Rt* 10.17 mins. **LCMS** $[M+H]^+$ 833.56.
**M13x**	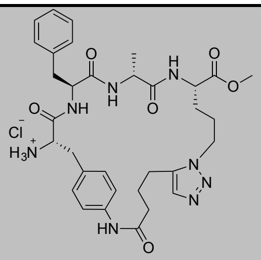	GP3 & GP5<br>21%<br>80%	**HPLC** (5-100% ACN) *Rt* 6.65 mins. **LCMS** $[M+H]^+$ 647.36.
**N8x**	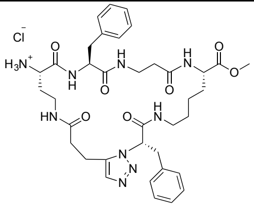	GP3<br>15%<br>93%	**HPLC** (5-100% ACN) *Rt* 8.40 mins. **LCMS** $[M+H]^+$ 832.64.

15. Preparation of B/C/P DKPs

	Compound	Method, Yield (%), Purity (%)	Analysis
**A1y**	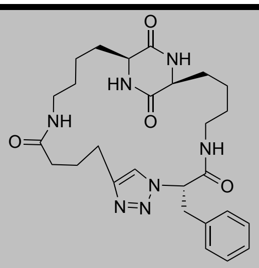	GP6<br>85%<br>90%	**HPLC** (5-80% ACN) *Rt* 7.56 mins. **LCMS** $[M+H]^+$ 524.26.
**A1z**	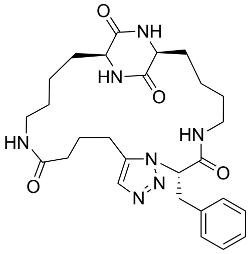	GP6<br>84%<br>90%	**HPLC** (5-80% ACN) *Rt* 7.43 mins. **LCMS** $[M+H]^+$ 524.26.
**A2y**	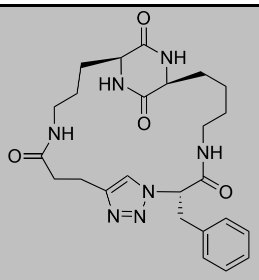	GP6<br>90%<br>86%	**HPLC** (5-45% ACN) *Rt* 9.48 mins. **LCMS** $[M+H]^+$ 496.23.
**A2z**	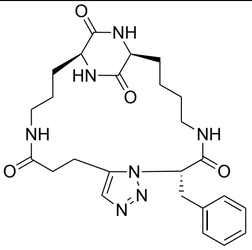	GP6<br>17%<br>61%	**HPLC** (5-45% ACN) *Rt* 9.81 mins. **LCMS** $[M+H]^+$ 496.23.

**A3y**	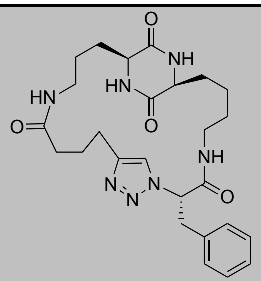	GP6<br>92%<br>78%	**HPLC** (5-100% ACN) *Rt* 6.75 mins. **LCMS** $[M+H]^+$ 510.41.
**A3z**	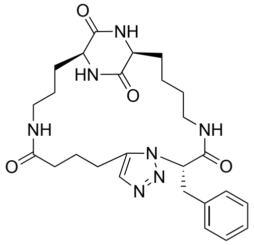	GP6<br>28%<br>74%	**HPLC** (5-100% ACN) *Rt* 6.53 mins. **LCMS** $[M+H]^+$ 510.26.
**A4z**	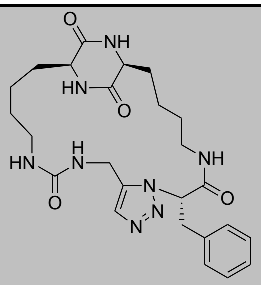	GP6<br>44%<br>95%	**HPLC** (5-45% ACN) *Rt* 10.07 mins. **LCMS** $[M+H]^+$ 511.23.
**A5z**	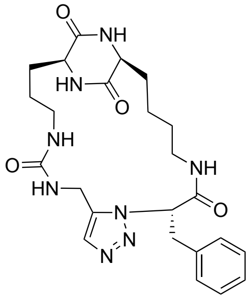	GP6<br>56%<br>64%	**HPLC** (5-45% ACN) *Rt* 9.51 mins. **LCMS** $[M+H]^+$ 497.21.
**A6z**	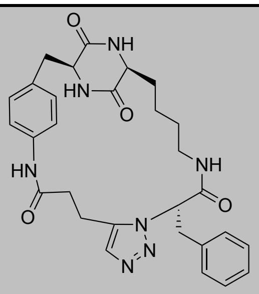	GP6<br>19%<br>64%	**HPLC** (5-100% ACN) *Rt* 7.47 mins. **LCMS** $[M+H]^+$ 544.15.

**A7y**	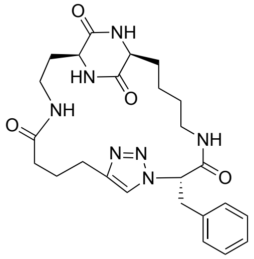	GP6<br>42%<br>51%	**HPLC** (5-45% ACN) *Rt* 9.99 mins. **LCMS** $[M+H]^+$ 496.08.
**A7z**	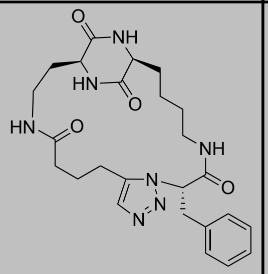	GP6<br>34%<br>53%	**HPLC** (5-45% ACN) *Rt* 9.90 mins. **LCMS** $[M+H]^+$ 496.23.
**A8z**	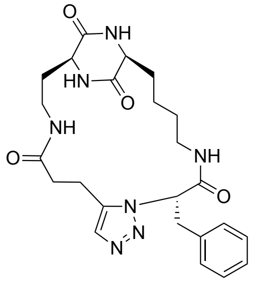	GP6<br>98%<br>56%	**HPLC** (5-45% ACN) *Rt* 9.61 mins. **LCMS** $[M+H]^+$ 482.21.
**A9y**	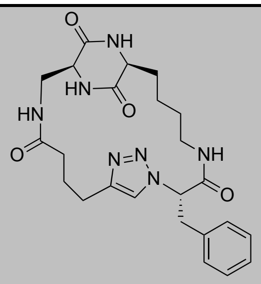	GP6<br>44%<br>61%	**HPLC** (5-100% ACN) *Rt* 6.71 mins. **LCMS** $[M+H]^+$ 482.14.
**A9z**	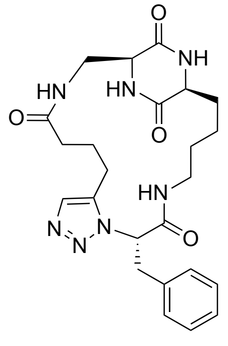	GP6<br>69%<br>50%	**HPLC** (5-100% ACN) *Rt* 6.48 mins. **LCMS** $[M+H]^+$ 482.14.

**A10 z**	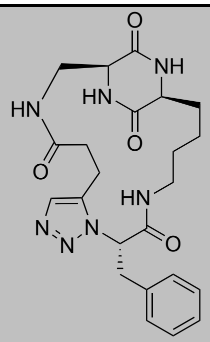	GP6<br>23%<br>57%	**HPLC** (5-100% ACN) *Rt* 6.43 mins. **LCMS** $[M+H]^+$ 468.11.
**A11 z**	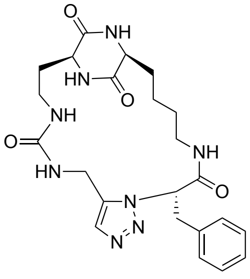	GP6<br>33%<br>71%	**HPLC** (5-100% ACN) *Rt* 6.08 mins. **LCMS** $[M+H]^+$ 483.11.
**A12 y**	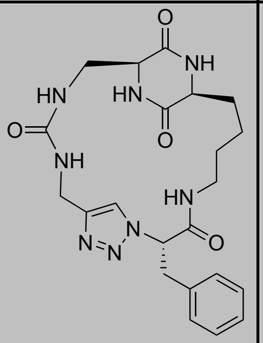	GP6<br>58%<br>64%	**HPLC** (5-100% ACN) *Rt* 6.27 mins. **LCMS** $[M+H]^+$ 469.09.
**A12 z**	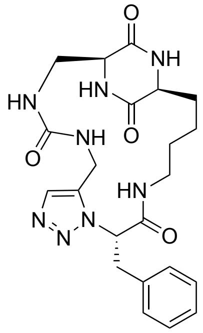	GP6<br>60%<br>64%	**HPLC** (5-100% ACN) *Rt* 6.34 mins. **LCMS** $[M+H]^+$ 469.16.
**A13 z**	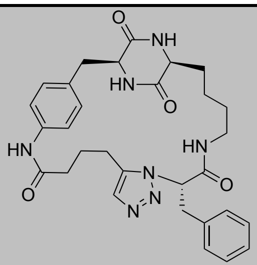	GP6<br>48%<br>95%	**HPLC** (5-100% ACN) *Rt* 7.27 mins. **LCMS** $[M+H]^+$ 558.18.

**B1y**	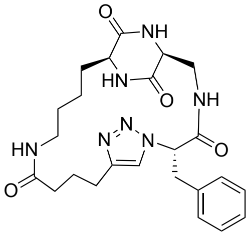	GP6<br>89%<br>57%	**HPLC** (5-45% ACN) *Rt* 9.92 mins. **LCMS** $[M+H]^+$ 482.21.
**B1z**	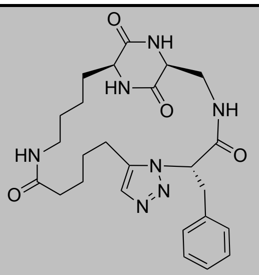	GP6<br>92%<br>88%	**HPLC** (5-45% ACN) *Rt* 9.36 mins. **LCMS** $[M+H]^+$ 482.21.
**B2y**	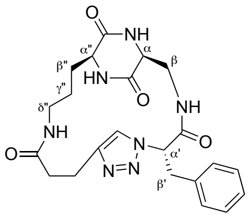	GP6<br>73%<br>82%	**R_f** = 0.18 (15% MeOH/ 85% CH_2Cl_2). **δ_H /ppm** (500 Hz, d_6-DMSO, 120 °C): 7.60-7.55 (2H, m, triazole C*H* and N*H*), 7.49 (1H, s, N*H*), 7.27-7.06 (7H, m, 5 × ArC*H* and 2 × N*H*), 5.45 (1H, dd, *J*=8.4, 6.6 Hz, $H_{\alpha'}$), 3.99 (1H, s, H_{α}), 3.91-3.83 (1H, m, H_{β}), 3.74 (1H, s, $H_{\alpha''}$), 3.44 (1H, dd, *J*=14.1, 6.6 Hz, $H_{\beta'}$), 3.33 (2H, dd, *J*=14.1, 8.4 Hz, $\underline{H}_{\beta'}$), 3.16-3.05 (1H, m, $H_{\delta''}$), 2.98-2.88 (2H, m, $H_{\delta''}$ and $COCH_2CH_2$), $COCH_2C\underline{H_2}$ below H_2O signal, 2.44-2.40 (2H, m, $COCH_2CH_2$), 1.66-1.54 (1H, m, $H_{\beta''}$), 1.35-1.24 (3H, m, $\underline{H}_{\beta''}$ and $H_{\gamma''}$). **δ_C /ppm** (125 MHz, d_6-DMSO): 171.1 (C=OCH_2CH_2), 167.4 ($C_{\alpha'}C$=O), 167.2 ($C_{\alpha}C$=O or $C_{\alpha''}C$=O), 164.7 ($C_{\alpha}C$=O or $C_{\alpha''}C$=O), 144.9 (triazole *C*), 136.4 (Ar*C*), 128.9 (Ar*C*H), 128.2 (Ar*C*H), 126.7 (Ar*C*H), 121.8 (triazole *C*H), 63.6 ($C_{\alpha'}$), 54.0 (C_{α}), 53.7 ($C_{\alpha''}$), 40.3 (C_{β}), 38.6 ($C_{\beta'}$), 37.8 ($C_{\delta''}$), 33.9 ($COCH_2CH_2$), 30.2 ($C_{\beta''}$), 25.2 ($C_{\gamma''}$), 21.3 ($COCH_2CH_2$). **ν_{max} /cm^{-1}:** 3263 (m, N-H), 2926 (m, C-H str), 1661 (s, C=O str), 1550 (m, C=C). **HPLC** (5-100% ACN) *Rt* 5.99 mins. **HRMS** (ESI+) *m/z* found $[M+H]^+$ 454.2220, $C_{22}H_{28}N_7O_4^+$ required 454.2203 (Δ 3.7 ppm). **$[\alpha]_D^{25}$** = +15.0 (*c* 0.21, MeOH).
**B2z**	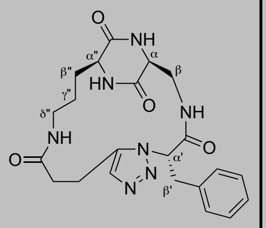	GP6<br>88%<br>76%	**R_f** = 0.11 (15% MeOH/ 85% CH_2Cl_2). **δ_H /ppm** (500 Hz, d_6-DMSO, 90 °C): 7.79 (1H, s, C_{β}-N*H*), 7.74 (1H, s, N*H*), 7.60-7.51 (2H, m, 2 × N*H*), 7.41 (1H, s, triazole C*H*), 7.25-7.11 (3H, m, 3 × ArC*H*), 6.97 (2H, dd, *J*=7.3, 1.6 Hz, 2 × ArC*H*), 5.32 (1H, dd, *J*=9.9, 5.0 Hz, $H_{\alpha'}$), 4.12-4.03 (1H, m, H_{α}), 3.77 (1H, t, *J*=4.5 Hz, $H_{\alpha''}$), 3.74-3.67 (1H, m, H_{β}), 3.66-3.58 (1H, m, H_{β}), 3.49 (1H, dd, *J*=13.9, 5.0 Hz, $H_{\beta'}$), 3.32 (1H, dd, *J*=13.9, 9.9 Hz, $H_{\beta'}$), $H_{\delta''}$ below H_2O signal, $COCH_2CH_2$ below H_2O signal, 2.39-2.21 (3H, m, $COCH_2C\underline{H_2}$, $COCH_2CH_2$), 1.71-1.57 (2H, m, $H_{\beta''}$), 1.41-1.23 (2H, m, $H_{\gamma''}$). **δ_C /ppm** (125 MHz, d_6-DMSO): 170.9 (C=OCH_2CH_2), 167.6 ($C_{\alpha'}C$=O), 167.5 ($C_{\alpha}C$=O or $C_{\alpha''}C$=O), 164.9 ($C_{\alpha}C$=O or $C_{\alpha''}C$=O), 137.7 (triazole *C*), 136.7 (Ar*C*), 131.0 (triazole *C*H), 129.1 (Ar*C*H), 128.2 (Ar*C*H), 126.7 (Ar*C*H), 61.3 ($C_{\alpha'}$), 54.3 ($C_{\alpha''}$), 53.9 (C_{α}), 40.5 (C_{β}), 38.4 ($C_{\delta''}$), 37.9 ($C_{\beta'}$), 34.2 ($COCH_2CH_2$), 30.3 ($C_{\beta''}$), 24.9 ($C_{\gamma''}$), 19.1 ($COCH_2CH_2$). **ν_{max} /cm^{-1}:** 3242 (m, N-H), 2921 (m, C-H), 1661 (s, C=O), 1545 (m, C=C). **HPLC** (5-100% ACN) *Rt* 6.22 mins. **HRMS** (ESI+) *m/z* found $[M+H]^+$ 454.2202, $C_{22}H_{28}N_7O_4^+$required 454.2203(Δ -0.2 ppm). **$[\alpha]_D^{25}$** = -7.5 (*c* 0.32, MeOH).

**B3y**	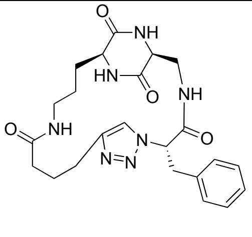	GP6<br>90%<br>66%	**HPLC** (5-45% ACN) *Rt* 9.17 mins. **LCMS** $[M+H]^+$ 468.13.
**B3z**	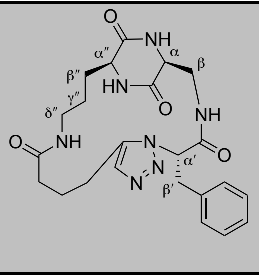	GP6<br>85%<br>62%	**HPLC** (5-45% ACN) *Rt* 9.07 mins. **LCMS** $[M+H]^+$ 468.11.
**B4y**	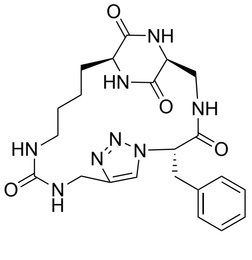	GP6<br>89%<br>73%	**HPLC** (5-45% ACN) *Rt* 5.83 mins. **LCMS** $[M+H]^+$ 469.16.
**B4z**	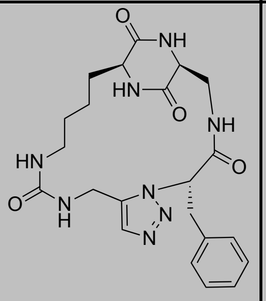	GP6<br>28%<br>67%	**HPLC** (5-100% ACN) *Rt* 6.19 mins. **LCMS** $[M+H]^+$ 469.24.
**B5y**	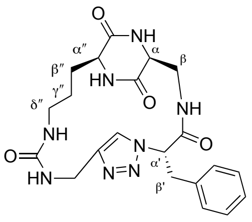	GP6<br>38%<br>74%	**Mp** = 245-249 °C (CH_2Cl_2). **δ_H /ppm** (500 MHz, d_6-DMSO): 8.10 (1H, app. s, C_β-N*H*), 8.04 (1H, app. s, $C_{\alpha''}$-N*H*), 7.91 (1H, app. s, C_α-N*H*), 7.60 (1H, s, triazole C*H*), 7.28-7.05 (5H, m, 5 × ArC*H*), 6.37 (1H, t, *J*=6.6 Hz, N*H*CH_2), 6.04 (1H, t, *J*=6.2 Hz, $C_{\delta''}$-N*H*), 5.54 (1H, t, *J*=7.6 Hz, $H_{\alpha'}$), 4.20 (1H, dd, *J*=15.9, 7.0 Hz, NHCH_2), 4.11-4.04 (1H, m, NHC$\underline{H_2}$), 4.04-3.93 (2H, m, H_β and H_α), 3.72 (1H, app. s, $H_{\alpha''}$), 3.47-3.37 (2H, m, $H_{\beta'}$), 3.07-3.00 (1H, m, $H_{\delta''}$), 2.99-2.88 (2H, m, $\underline{H}_\beta$ and $\underline{H}_{\delta''}$), 1.77-1.67 (1H, m, $H_{\beta''}$), 1.48-1.29 (3H, m, $\underline{H}_{\beta''}$ and $H_{\gamma''}$). **δ_C /ppm** (125 MHz, d_6-DMSO): 167.6 ($C_{\alpha''}$*C*=O or C_α*C*=O), 167.1 ($C_{\alpha'}$*C*=O), 164.7 ($C_{\alpha''}$*C*=O or C_α*C*=O), 158.5 (NH*C*=ONH), 146.9 (triazole C), 136.7 (Ar*C*), 129.0 (Ar*C*H), 128.3 (Ar*C*H), 126.7 (Ar*C*H), 122.1 (triazole *C*H), 63.4 ($C_{\alpha'}$), 54.4 ($C_{\alpha''}$), 53.8 (C_α), C_β below DMSO signal, 38.6 ($C_{\delta''}$), 36.8 ($C_{\beta'}$), 35.5 (NH*C*H_2), 29.1 ($C_{\beta''}$), 27.2 ($C_{\gamma''}$). **v_{max} /cm^{-1}:** 3281 (NH str), 2921 (CH str), 1670 (C=O str), 1548, 1454, 1119, 1051. **HPLC** (5-100% ACN) *Rt* 5.83 mins. **HRMS** (ESI+) *m/z* found $[M+H]^+$ 455.2159, $C_{21}H_{27}N_8O_4^+$ required 455.2155. **$[\alpha]_D^{25}$** = +4.0 (*c* 0.15, MeOH).

**B7z**	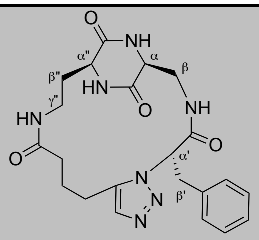	GP6<br>49%<br>62%	**HPLC** (5-100% ACN) *Rt* 6.38 mins. **LCMS** $[M+H]^+$ 454.09.
**B8y**	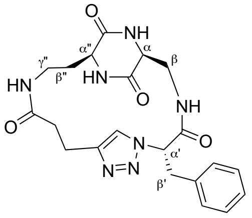	GP6<br>62%<br>90%	$\mathbf{R_f}$ = 0.12 (15% MeOH/ 85% CH_2Cl_2). $\boldsymbol{\delta_H}$ **/ppm** (500 Hz, d_6-DMSO): 8.40 (1H, dd, *J*=8.0, 4.2 Hz, C_β-N*H*), 8.28 (1H, d, *J*=2.4 Hz, $C_{\alpha''}$-N*H*), 7.93 (1H, d, *J*=2.3 Hz, C_α-N*H*), 7.67 (1H, dd, *J*=8.1, 3.8 Hz, $C_{\gamma''}$-N*H*), 7.61 (1H, s, triazole C*H*), 7.26-7.13 (3H, m, 3 × ArC*H*), 7.12-7.06 (2H, m, 2 × ArC*H*), 5.60 (1H, t, *J*=7.7 Hz, $H_{\alpha'}$), 3.99 (1H, ddd, *J*=13.6, 8.0, 1.4 Hz, H_β), 3.93-3.87 (1H, m, H_α), 3.54-3.49 (1H, m, $H_{\alpha''}$), 3.44-3.37 (1H, m, $H_{\gamma''}$), 3.26 (2H, d, *J*=7.7 Hz, $H_{\beta'}$), 2.92-2.83 (2H, m, $\underline{H}_\beta$ and $COCH_2CH_2$), 2.78 (1H, dt, *J*=14.3, 4.8 Hz, $COCH_2C\underline{H}_2$), 2.68-2.59 (1H, m, $\underline{H}_{\gamma''}$), 2.39-2.32 (1H, m, $COCH_2CH_2$), 2.28 (1H, dt, *J*=10.4, 5.0 Hz, $COC\underline{H}_2CH_2$), 1.81-1.73 (1H, m, $H_{\beta''}$), 0.78-0.67 (1H, m, $\underline{H}_{\beta''}$). $\boldsymbol{\delta_C}$ **/ppm** (125 MHz, d_6-DMSO): 170.7 (*C*=OCH_2CH_2), 167.9 ($C_{a'}$*C*=O), 167.0 ($C_{\alpha''}$*C*=O), 165.1 (C_α*C*=O), 144.6 (triazole *C*), 136.3 (Ar*C*), 129.0 (ArCH), 128.2 (Ar*C*H), 126.7 (Ar*C*H), 121.5 (triazole *C*H), 63.4 ($C_{\alpha'}$), 54.3 (C_α), 53.4 ($C_{\alpha''}$), 40.6 (C_β), 38.5 ($C_{\beta'}$), 36.8 ($C_{\beta''}$), 36.1 ($C_{\gamma''}$), 35.2 ($COCH_2CH_2$), 21.8 ($COCH_2CH_2$). $\mathbf{v_{max}}$ **/cm^{-1}:** 3224 (m, N-H), 2933 (m, C-H), 1663 (s, C=O str), 1549 (s, C=C). **HRMS** (ESI+) *m/z* found $[M+H]^+$ 440.2054, $C_{21}H_{26}N_7O_4^+$ required 440.2046 (Δ 1.8 ppm). $\mathbf{[\alpha]_D^{25}}$ = +40.0 (*c* 0.15, MeOH).
**B8z**	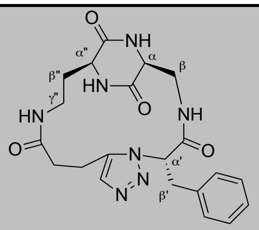	GP6<br>91%<br>64%	**HPLC** (5-100% ACN) *Rt* 6.20 mins. **LCMS** $[M+H]^+$ 440.12.
**B10y**	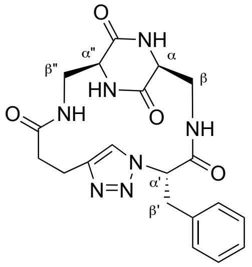	GP6<br>24%<br>80%	**HPLC** (5-45% ACN) *Rt* 5.83 mins. **LCMS** $[M+H]^+$ 469.16.

**B10 z**	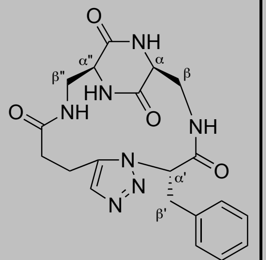	GP6<br>74%<br>74%	**R_f** = 0.07 (15% MeOH/ 85% CH_2Cl_2). **δ_H /ppm** (400 Hz, d_6-DMSO): 8.17 (1H, s, N*H*-CH-CH_2-NH), 7.85 (1H, d, *J*=10.5 Hz, NH'-CH'-CH_2'-N*H'*), 7.76 (1H, d, *J*=9.4 Hz, NH-CH-CH_2-N*H*), 7.65 (1H, s, N*H'*-CH'-CH_2'-NH'), 7.51 (1H, s, triazole C*H*), 7.31-7.00 (5H, m, 5 × ArC*H*), 5.35 (1H, t, *J*=7.5 Hz, Hα'), 4.18 (1H, ddd, *J*=12.5, 9.4, 1.6 Hz, NH-CH-C*H_2*-NH), 4.07 (1H, app s, NH-C*H*-CH_2-NH), 3.92-3.82 (2H, m, NH'-C*H'*-CH_2'-NH' and NH'-CH'-C*H_2'*-NH'), 3.77 (1H, dd, *J*=13.9, 7.4 Hz, $H_{\beta'}$), 3.47-3.38 (1H, m, $H_{\beta'}$), 3.18-3.10 (1H, m, NH'-CH'-CH_2'-NH'), 2.85 (1H, ddd, *J*=17.6, 7.6, 3.5 Hz, $COCH_2CH_2$), 2.69 (1H, dd, *J*=12.5, 2.8 Hz, NH-CH-CH_2-NH), $COCH_2CH_2$ below H_2O signal, 2.37-2.24 (1H, m, $COCH_2CH_2$), 2.03-1.92 (1H, m, $COCH_2CH_2$). **δ_C /ppm** (125 MHz, d_6-DMSO): 171.7 (*C*=O), 167.8 (*C*=O), 165.8 (*C*=O), 164.4 (*C*=O), 136.6 (ArC), 135.6 (triazole *C*), 133.0 (triazole *C*H), 129.2 (Ar*C*H), 128.3 (ArCH), 126.8 (Ar*C*H), 61.8 ($C_{\alpha'}$), 54.9 (*C*H), 54.0 (*C*H'), *C*H_2 and *C*H_2' below DMSO signal 36.2 ($C_{\beta'}$), 34.8 ($COCH_2CH_2$), 20.6 ($COCH_2CH_2$). **v_{max} /cm^{-1}:** 3233 (w, N-H), 2923 (m, C-H), 1661 (s, C=O), 1550 (m, C=C). **HPLC** (5-100% ACN) *Rt* 6.20 mins. **HRMS** (ESI+) *m/z* found $[M+H]^+$ 426.1890, $C_{20}H_{24}N_7O_4^+$ required 426.1890 (Δ 0.0 ppm). **$[\alpha]_D^{25}$** = +14.0 (*c* 0.23, MeOH).
**B12 z**	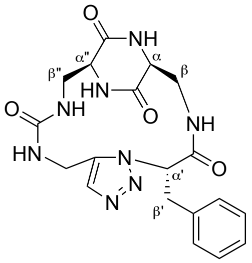	GP6<br>57%<br>82%	**R_f** = 0.02 (15% MeOH/ 85% CH_2Cl_2). **δ_H /ppm** (500 Hz, d_6-DMSO): 8.02 (1H, s, C_α-N*H*), 7.92 (1H, s, $C_{\alpha''}$-N*H*), 7.37 (1H, d, *J*=5.4 Hz, triazole C*H*), 7.23-7.09 (3H, m, 3 × ArC*H*), 6.98 (1H, t, *J*=6.0 Hz, N*H*CH_2), 6.94 (2H, dd, *J*=7.7, 1.5 Hz, 2 × ArC*H*), 6.89 (1H, t, *J*=5.3 Hz, C_β-N*H*), 5.84 (1H, t, *J*=6.1 Hz, $C_{\beta''}$-N*H*), 5.44 (1H, dd, *J*=10.6, 4.8 Hz, $H_{\alpha'}$), 4.05 (1H, app s, H_α), 3.98 (1H, dd, *J*=15.8, 5.4 Hz, NHC*H_2*), 3.84-3.80 (1H, m, $H_{\alpha''}$), 3.61 (1H, ddd, *J*=13.4, 5.5, 2.5 Hz, H_β), 3.46 (1H, dd, *J*=13.6, 4.8 Hz, $H_{\beta'}$), 3.43-3.36 (2H, m, NHCH_2 and H_β), $H_{\beta''}$ below H_2O signal, 3.27-3.22 (1H, m, $H_{\beta'}$). **δ_C /ppm** (125 MHz, d_6-DMSO): 167.0 ($C_{a'}$*C*=O), 166.1 (C_α*C*=O or $C_{\alpha''}$*C*=O), 164.4 (C_α*C*=O or $C_{\alpha''}$*C*=O), 157.5 (NH*C*=ONH), 136.8 (triazole *C*), 136.8 (Ar*C*), 132.3 (triazole *C*H), 128.9 (Ar*C*H), 128.4 (ArCH), 126.8 (Ar*C*H), 61.6 ($C_{\alpha'}$), 54.9 ($C_{\alpha''}$), 53.7 (C_α), 40.8 (C_β), 40.6 ($C_{\beta''}$), 38.6 ($C_{\beta'}$), 31.1 (NH*C*H_2). **v_{max} /cm^{-1}:** 3248 (m, N-H str), 2923 (m, C-H str), 1662 (s, C=O str), 1564 (m, C=C). **HPLC** (5-100% ACN) *Rt* 5.96 mins. **HRMS** (ESI+) *m/z* found $[M+H]^+$ 427.1843, $C_{19}H_{23}N_8O_4^+$required 427.1842 (Δ 0.2 ppm). **$[\alpha]_D^{25}$** = -17.0 (*c* 0.19, MeOH).
**B14 y**	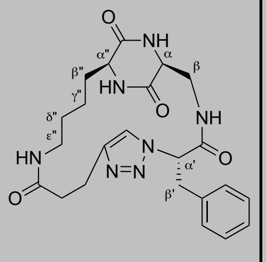	GP6<br>55%<br>80%	**R_f** = 0.25 (15% MeOH/ 85% CH_2Cl_2). **δ_H /ppm** (500 Hz, d_6-DMSO, 120 °C): 7.66-7.55 (2H, m, triazole C*H* and C_β-N*H*), 7.44 (1H, s, N*H*), 7.34 (1H, s, C_α-N*H*), 7.18 (5H, m, 5 × ArC*H*), 7.04 (1H, s, N*H*), 5.50 (1H, dd, *J*=8.7, 6.4 Hz, $H_{\alpha'}$), 3.95 (1H, app s, H_α), 3.83-3.73 (2H, m, H_β and $H_{\alpha''}$), 3.45 (1H, dd, *J*=14.2, 6.4 Hz, $H_{\beta'}$), 3.38-3.26 (2H, m, $H_{\beta'}$ and H_β), 3.15-3.06 (1H, m, $H_{\varepsilon''}$), 3.06-2.97 (1H, m, $H_{\varepsilon''}$), 2.97- 2.85 (2H, m, $COCH_2$C*H_2*), 2.47-2.35 (2H, m CO*CH_2*CH_2), 1.69-1.61 (2H, m, $H_{\beta''}$), 1.38-1.29 (2H, m, $H_{\delta''}$), 1.18-1.09 (2H, m, $H_{\gamma''}$). **δ_C /ppm** (125 MHz, d_6-DMSO): 170.9 (*C*=OCH_2CH_2), 167.8 ($C_{a'}$*C*=O), 167.8 ($C_{\alpha''}$*C*=O or C_α*C*=O), 165.5 ($C_{\alpha''}$*C*=O or C_α*C*=O), 145.3 (triazole *C*), 136.3 (Ar*C*), 129.0 (Ar*C*H), 128.2 (ArCH), 126.7 (Ar*C*H), 121.6 (triazole *C*H), 63.7 ($C_{\alpha'}$), 54.5 (C_α), 53.3 ($C_{\alpha''}$), 41.0 (C_β), $C_{\beta'}$ below DMSO signal, 36.9 ($C_{\varepsilon''}$), 34.4 (CO*C*H_2CH_2), 33.4 ($C_{\beta''}$), 27.5 ($C_{\delta''}$), 21.2 ($COCH_2$*C*H_2), 19.0 ($C_{\gamma''}$). **v_{max} /cm^{-1}:** 3252 (m, N-H), 2925 (m, C-H), 1662 (s, C=O), 1543 (m, C=C). **HPLC** (5-100% ACN) *Rt* 6.06 mins. **HRMS** (ESI+) *m/z* found $[M+H]^+$ 468.2356, $C_{23}H_{30}N_7O_4^+$ required 468.2354 (Δ 0.4 ppm). **$[\alpha]_D^{25}$** = +0.8 (*c* 0.55, MeOH).

**B14z**	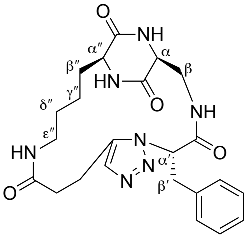	GP6<br>99%<br>78%	**R_f** = 0.10 (10% MeOH/ 90% CH_2Cl_2). **Mp** = 176-178 °C (CH_2Cl_2). **δ_H /ppm** (400 MHz, *d*$_6$-DMSO): 8.47 (1H, dd, *J*=6.4, 5.1 Hz, C_β-N*H*), 8.08 (1H, d, *J*=2.4 Hz, C_α-N*H*), 7.96 (1H, dd, *J*=6.9, 5.2 Hz, $C_{\varepsilon''}$-N*H*), 7.90 (1H, d, *J*=1.8 Hz, $C_{\alpha''}$-N*H*), 7.46 (1H, s, triazole C*H*), 7.29-7.08 (5H, m, 5 × ArCH), 5.60 (1H, dd, *J*=10.5 Hz and 4.7 Hz, $H_{\alpha'}$), 4.15-4.04 (1H, m, H_α), 3.84-3.73 (1H, m, $H_{\alpha''}$), 3.71-3.53 (2H, m, $H_{\beta'}$), 3.53-3.43 (1H, m, H_β), $\underline{H}_\beta$ and $H_{\varepsilon''}$ below H_2O signal, 2.96-2.84 (1H, m, $\underline{H}_{\varepsilon''}$), 2.84-2.64 (1H, m, $COCH_2CH_2$), $H_{c'}$ below DMSO signal, 2.49-2.38 (2H, m, $COCH_2CH_2$), 1.81-1.56 (2H, m, $H_{\beta''}$), 1.52-1.39 (1H, m, $H_{\delta''}$), 1.39-1.16 (3H, m, $H_{\delta''}$ and $H_{\gamma''}$). **δ_C /ppm** (125 MHz, *d*$_6$-DMSO): 171.4 (*C*=OCH_2CH_2), 167.9 ($C_\alpha C$=O or $C_{\alpha''}C$=O), 167.5 ($C_{\alpha'}C$=O), 166.2 ($C_\alpha C$=O or $C_{\alpha''}C$=O), 138.2 (triazole C), 137.3 (Ar*C*), 131.0 (triazole *C*H), 128.7 (Ar*C*H), 128.2 (ArCH), 126.5 (Ar*C*H), 61.6 ($C_{\alpha'}$), 54.4 ($C_{\alpha''}$), 52.7 (C_α), 41.7 (C_β), 36.1 ($C_{\varepsilon''}$), 35.7 ($C_{\beta'}$), 32.8 ($COCH_2CH_2$), 31.7 ($C_{\beta''}$), 28.4 ($C_{\delta''}$), 19.4 ($C_{\gamma''}$), 18.7 ($COCH_2CH_2$). **v_{max} /cm^{-1}:** 3239 (w, N-H), 2924 (m, C-H), 1650 (s, C=O str), 1544 (s, C=C). **HPLC** (5-100% ACN) *Rt* 6.35 mins. **HRMS** (ESI+) *m/z* found $[M+H]^+$ 468.2376, $C_{23}H_{30}N_7O_4^+$ required 468.2359 (Δ 2.3 ppm). **$[\alpha]_D^{25}$** = -10.0 (*c* 0.235, MeOH).
**D1z**	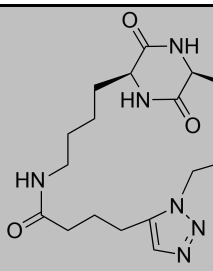	GP6<br>99%<br>64%	**HPLC** (5-25% ACN) *Rt* 5.10 mins. **LCMS** $[M+H]^+$ 377.27.
**D2y**	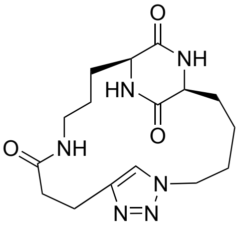	GP6<br>99%<br>91%	**HPLC** (5-45% ACN) *Rt* 3.36 mins. **LCMS** $[M+H]^+$ 349.13.
**D3y**	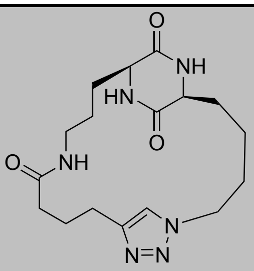	GP6<br>53%<br>70%	**HPLC** (5-45% ACN) *Rt* 4.45 mins. **LCMS** $[M+H]^+$ 363.31.
**D7z**	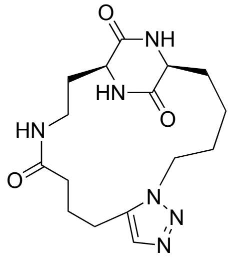	GP6<br>30%<br>71%	**HPLC** (5-45% ACN) *Rt* 4.00 mins. **LCMS** $[M+H]^+$ 349.14.

**D8y**	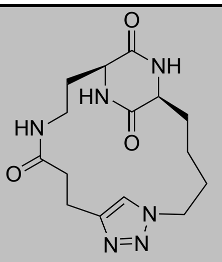	GP6<br>41%<br>74%	**HPLC** (5-45% ACN) *Rt* 3.36 mins. **LCMS** $[M+H]^+$ 335.10.
**D8z**	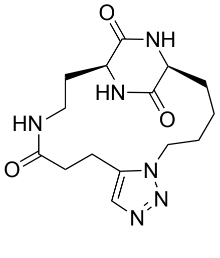	GP6<br>33%<br>72%	**HPLC** (5-100% ACN) *Rt* 3.39 mins. **LCMS** $[M+H]^+$ 335.13.
**D9z**	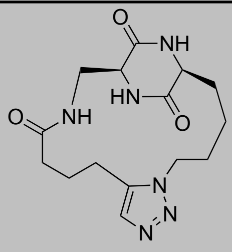	GP6<br>21%<br>75%	**HPLC** (5-100% ACN) *Rt* 3.54 mins. **LCMS** $[M+H]^+$ 335.13.
**D11y**	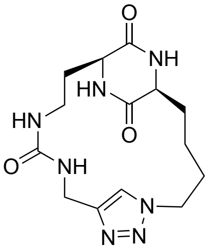	GP6<br>32%<br>66%	**HPLC** (5-100% ACN) *Rt* 2.99 mins. **LCMS** $[M+H]^+$ 336.10.
**D13z**	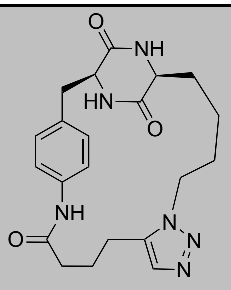	GP6<br>9%<br>78%	**HPLC** (5-100% ACN) *Rt* 5.30 mins. **LCMS** $[M+H]^+$ 411.13.
**D14y**	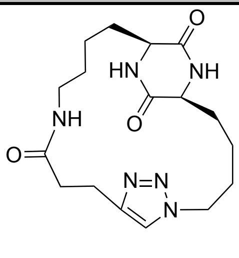	GP6<br>87%<br>90%	**v_{max} /cm^{-1}:** 3228, 2924, 2859, 1653, 1549, 1452, 1333, 1270, 1055. **HPLC** (5-80% ACN) *Rt* 3.84 mins. **HRMS** (ESI+) *m/z* found $[M+H]^+$ 363.2140, $C_{17}H_{27}N_6O_3^+$ required 363.2145.

**D14z**	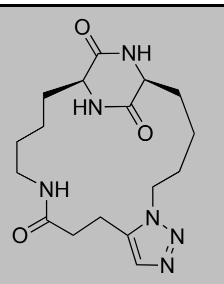	GP6<br>94%<br>87%	**δ_H /ppm** (500 MHz, d_6-DMSO): 8.01 (1H, d, *J*=1.8 Hz), 7.97 (1H, s), 7.87 (1H, t, *J*=5.6 Hz), 7.52 (1H, s), 4.30 (2H, t, *J*=6.4 Hz), 3.94- 3.90 (1H, m), 3.88-3.83 (1H, m), 3.19-3.01 (2H, m), 2.91-2.80 (2H, m), 2.40-2.33 (2H, m), 1.87-1.71 (4H, m), 1.68-1.58 (1H, m), 1.57-1.47 (1H, m), 1.44-0.98 (6H, m). **δ_C /ppm** (125 MHz, d_6-DMSO): 170.8, 167.3, 167.2, 136.7, 131.5, 53.9, 53.9, 46.6, 38.4, 34.7, 32.3, 31.6, 30.1, 28.4, 20.8, 20.3, 19.9. **v_{max} /cm^{-1}:** 3229, 2925, 2859, 1650, 1551, 1431, 1332. **HPLC** (5-80% ACN) *Rt* 3.66 mins. **HRMS** (ESI+) *m/z* found $[M+Na]^+$ 385.1966, $C_{17}H_{26}N_6O_3Na^+$ required 385.1964.
**E1y**	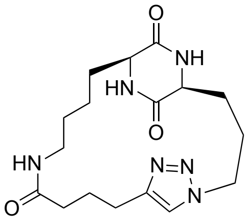	GP6<br>90%<br>76%	**HPLC** (5-45% ACN) *Rt* 4.06 mins. **LCMS** $[M+H]^+$ 363.23.
**E1z**	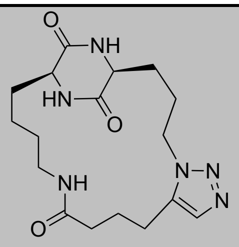	GP6<br>43%<br>95%	**HPLC** (5-45% ACN) *Rt* 4.12 mins. **LCMS** $[M+H]^+$ 363.16.
**E3z**	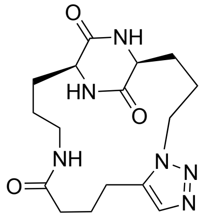	GP6<br>32%<br>93%	**HPLC** (5-45% ACN) *Rt* 3.60 mins. **LCMS** $[M+H]^+$ 349.14.
**E6z**	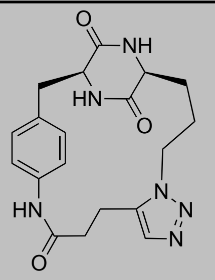	GP6<br>16%<br>99%	**HPLC** (5-100% ACN) *Rt* 4.59 mins. **LCMS** $[M+H]^+$ 383.10.
**E7z**	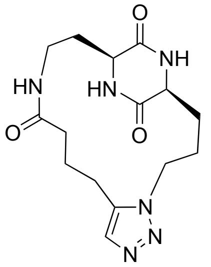	GP6<br>30%<br>85%	**HPLC** (5-45% ACN) *Rt* 3.36 mins. **LCMS** $[M+H]^+$ 335.13.

**E8z**	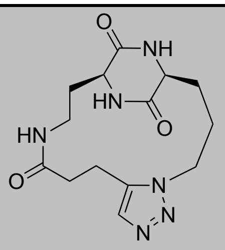	GP6<br>34%<br>84%	**HPLC** (5-45% ACN) *Rt* 2.65 mins. **LCMS** $[M+H]^+$ 321.12.
**E9z**	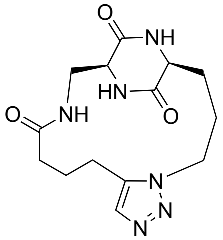	GP6<br>36%<br>86%	**HPLC** (5-45% ACN) *Rt* 3.16 mins. **LCMS** $[M+H]^+$ 321.12.
**E11 z**	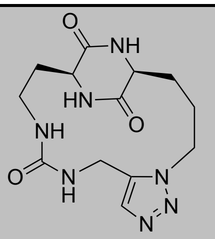	GP6<br>22%<br>74%	**HPLC** (5-100% ACN) *Rt* 2.82 mins. **LCMS** $[M+H]^+$ 322.17.
**E13 z**	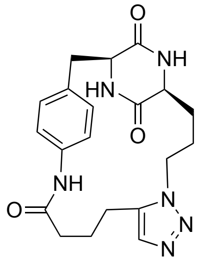	GP6<br>42%<br>87%	**HPLC** (5-100% ACN) *Rt* 5.06 mins. **LCMS** $[M+H]^+$ 397.12.
**E14 z**	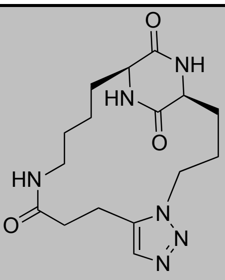	GP6<br>23%<br>90%	**HPLC** (5-45% ACN) *Rt* 3.43 mins. **LCMS** $[M+H]^+$ 349.14.

16. Preparation of B/C/C/P and B/C/C/C/P DKPs

	Compound	Method, Yield (%), Purity (%)	Analysis
**H2z**	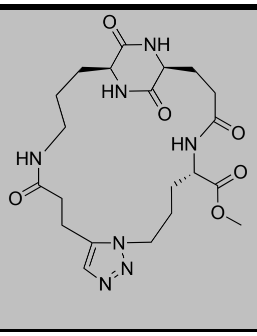	GP6<br>72%<br>73%	**HPLC** (5-45% ACN) *Rt* 5.19 mins. **LCMS** $[M+H]^+$ 478.09.
**H3z**	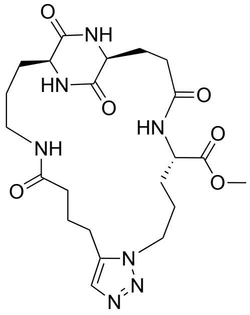	GP6<br>44%<br>71%	**HPLC** (5-45% ACN) *Rt* 5.78 mins. **LCMS** $[M+H]^+$ 492.11.
**H14z**	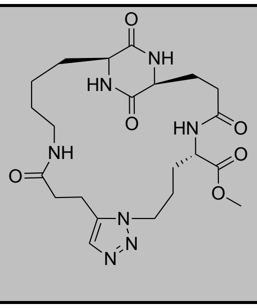	GP6<br>57%<br>89%	**HPLC** (5-45% ACN) *Rt* 5.49 mins. **LCMS** $[M+H]^+$ 492.26.

17. Chemoinformatic analysis

Principal component analysis

Principal component analysis (PCA) was carried out using the Molecular Operating Environment (MOE) software package[11]. A total of 15 physicochemical properties (Table S1) were obtained for 222 macrocylic DOS library members and established reference sets of 40 top-selling brand-name drugs, 60 diverse natural products and 24 macrocyclic natural products[12].

The summary of the PCA is shown in Table S2. The first three principal components account for 87.3% of the variance in the dataset and were used to generate Figures 3a-c in the manuscript.

Table S1. Physicochemical properties used in PCA

Parameter	Description
a_acc	number of H-bond acceptor atoms
a_aro	number of aromatic atoms
a_don	number of H-bond donor atoms
a_nN	number of nitrogen atoms
a_nO	number of oxygen atoms
b_rotN	number of rotatable bonds
chiral	number of chiral centers
KierFlex	molecular flexibility
logP(o/w)	log octanol/water partition coefficient
log S	log solubility in water
mr	molar refractivity
rings	number of rings
SlogP	log octanol/water partition coefficient
TPSA	topological polar surface area
Weight	molecular weight

Table S2. Standard deviation and contribution of each principal component to variance

	PC1	PC2	PC3	PC4	PC5	PC6	PC7	PC8	PC9	PC10
standard deviation	2.775	1.895	1.342	0.950	0.638	0.431	0.363	0.320	0.242	0.189
proportion of variance	0.514	0.640	0.120	0.060	0.027	0.012	0.009	0.007	0.004	0.002
cumulative proportion	0.514	0.753	0.873	0.933	0.960	0.973	0.982	0.988	0.992	0.995

Table S3. Component loadings for PCA of macrocycle library with established reference sets

Parameter	PC1	PC2	PC3
a_acc	0.038066	0.017781	-0.016773
a_aro	0.004001	0.005698	0.097251
a_don	0.041175	0.051005	-0.009315
a_nN	0.011825	0.073803	0.072352
a_nO	0.033946	-0.003718	-0.041977
b_rotN	0.018641	-0.011356	-0.017137
chiral	0.025172	-0.019564	-0.043522
KierFlex	0.027100	-0.003892	-0.030649
logP(o/w)	0.003847	-0.106012	0.004302
log S	-0.038518	0.084259	-0.073837
mr	0.026018	-0.005560	0.015126
rings	0.045324	-0.046568	0.262761
SlogP	0.000576	-0.122025	0.037147
TPSA	0.001592	0.001856	-0.000087
Weight	0.000677	-0.000057	0.000261

PCA Data:

DRUGS	PC1	PC2	PC3
Lipitor	0.44035336	-2.0922842	2.1781542
Nexium	-0.56550181	-0.68104434	1.0659434
Prevacid	-0.59435779	-0.92088705	1.1955206
Flonase	-0.14596421	-2.0885949	-0.57547647
Serevent	-0.061018318	-1.4764915	0.034017656
Singulair	0.29012862	-3.1157372	2.7073617
Effexor	-1.0479238	-1.1570442	-0.28889498
Plavix	-0.98261434	-1.5518504	0.69276941
Zocor	-0.36533195	-1.9022555	-0.87527949
Norvasc	-0.36513421	-0.94790769	-0.38823062
Lexapro	-0.90844232	-1.5983214	0.9173618
Seroquel	-0.61090952	-1.1148515	1.1556581
Protonix	-0.40576226	-0.63025695	0.93926328
Ambien	-1.0581216	-1.1308053	0.90474534
Actos	-0.61026162	-1.2560296	0.73286134
Zoloft	-1.0674506	-1.9562593	1.008045
Wellbutrin	-1.2362236	-1.2577442	-0.39570323
Avandia	-0.62388283	-0.96976894	0.7288534
Risperdal	-0.63164562	-1.2829981	1.3195344
Zyprexa	-1.0861667	-1.034258	0.81028336
Topamax	-0.4540301	-0.46410626	-1.1624413
Toprol	-0.87203783	-0.68965971	-0.87318957
Zetia	-0.44707555	-1.8197278	1.6259458
Fosamax	-0.56960052	1.4469109	-2.4331801

Abilify	-0.48024434	-1.6537925	1.2831823
Levaquin	-0.68553865	-0.47166136	0.31969854
Lamictal	-1.0722635	-0.50426841	0.85313201
Celebrex	-0.69964743	-1.2551666	1.5841386
Benazepril	-0.1722537	-1.0853065	0.47855175
Zyrtec	-0.47942379	-1.2031868	0.61839736
Coreg	-0.20518997	-1.339842	1.62435
Valtrex	-0.49919569	0.38027838	-0.43706414
Adderall	-1.6239618	-0.65025598	-0.53189367
Aciphex	-0.45343667	-0.75192112	1.0078642
Cymbalta	-0.92316884	-1.7507811	1.1712037
Crestor	0.14056866	-0.57007229	0.1367476
Diovan	0.054656371	-1.2894883	1.4949378
Tricor	-0.71295768	-1.9348887	0.51353991
Concerta	-1.1811528	-0.94267678	-0.35627496
Imitrex	-0.96518046	-0.42220587	0.019565227
**NATURAL PRODUCTS**	**PC1**	**PC2**	**PC3**
Forskolin	-0.22793074	-0.91015011	-1.172423
SQ26180	-0.87785536	0.66188121	-1.7100738
CephamycinC	0.33941141	0.94354922	-1.575893
Thienamycin	-0.76859432	0.17549638	-1.2966894
Artemisinin	-0.84689111	-1.4530129	-0.63662207
Coformycin	-0.54190427	0.84613544	-0.64143258
Arglabin	-1.1851399	-1.1351717	-0.51894414
Mizoribine	-0.59756994	1.015848	-1.0570055

Compactin	-0.44222924	-1.6801987	-0.96994025
Bestatin	-0.46068856	-0.25168779	-0.91969138
Plaunotol	-0.77315277	-1.5268536	-1.451203
Spergualin	0.23765998	0.91931868	-2.0552449
Taxol	1.9018565	-1.8936352	1.5840536
Rapamycin	1.914791	-2.4318838	-1.1371188
AvermectinB1a	1.9849749	-2.4668901	-0.73449647
PseudomonicAcidA	0.61720502	-1.125052	-1.9112897
Daptomycin	6.8387823	3.143348	-0.37000597
MidecamycinA1	1.8331497	-1.3506995	-2.1281457
EchinocandinB	3.58724	0.34384269	-0.91759968
CalicheamicinG1	4.5329742	-2.1788938	-0.43363619
Validamycin	1.0950502	2.0700731	-2.728308
CyclosporinA	3.0024731	-1.2290826	-1.1754317
FK506	1.5112494	-1.9028938	-1.1877556
Lipstatin	0.33398086	-2.8370755	-1.2085571
Geldanamycin	0.37187731	-0.82606244	-1.2769804
Actinonin	-0.15347186	-0.45159864	-1.5673654
Discodermolide	0.88530988	-1.6108406	-2.080821
Monensin	1.3066674	-2.1165187	-1.20472
CalyculinA	3.1664143	-1.1070825	-1.4811376
AmphotericinB	2.7472551	-0.22179392	-2.4432735
Adriamycin	0.75543278	-0.17172959	0.35423809
GinkgolideB	0.076751307	-0.42398295	-0.68785477
PhorbolMA	0.78936607	-2.4432287	-0.67789841
Vancomycin	5.5328674	0.41190192	3.1565979
TrapoxinB	0.52696449	-1.0188068	1.0778458
Vincristine	1.4792458	-1.6977637	2.2975564

Colchicine	-0.42637482	-1.0862933	-0.1127376
Trichostatin	-0.83681005	-0.86081833	-0.52208447
Fumagillin	0.074679755	-1.7961711	-0.92736107
Staurosporine	-0.10866929	-1.9755068	3.2671399
ErythromycinA	1.5086728	-0.96488166	-2.0675776
Streptomycin	1.5435889	2.7522595	-2.2994957
PenicillinG	-0.61026227	-0.60710198	-0.1307178
ZaragozicAcidA	1.706357	-1.4598362	-1.0643396
TalaromycinB	-1.2211469	-1.6863494	-0.92124099
Spongistatin1	3.7285621	-2.6810601	-1.2223319
Radicicol	-0.56284487	-1.185524	-0.17036377
SalicylihalamideA	-0.1880146	-1.5681546	-0.31392559
BrevetoxinB	2.0220592	-2.9930613	0.54134393
RifamycinB	1.5982065	-1.4188566	-0.030950295
Quinine	-0.76435798	-1.1905845	0.68034565
MycobactinS	2.098506	-2.4474444	-0.70494437
Telomestatin	0.63835406	-1.2211164	5.5911064
DuocarmycinA	0.20226467	-0.92060089	1.3871363
Bleomycin	6.0183692	4.0021272	0.19731249
BrefeldinA	-0.94019729	-0.88748139	-1.2230606
CytochalasinB	-0.051231857	-1.5982953	0.16306159
EpothiloneA	-0.087701909	-1.3141584	-0.46590239
Apoptolidin	3.6647611	-1.7290736	-2.2622764
Lactacystin	-0.09661448	0.32651547	-1.9021096

MACROCYCLIC NATURAL PRODUCTS	PC1	PC2	PC3
O1[C@@H](C[C@@H]2O[C@@H]2\C=C\[C@@H](O)CC1=O)C	-1.254164	-0.48006925	-1.288004
O1[C@@H](C[C@@H]2O[C@@H]2[C@H](O)CC(=O)CC1=O)C	-1.1514407	-0.23300733	-1.3959749
O1[C@@H](CC[C@H](O)\C=C\[C@@H](O)CC1=O)C	-1.2388685	-0.33497229	-1.5778235
O1[C@@H](C[C@H](O)\C=C\[C@H](O)[C@@H](O)CC1=O)C	-1.0686992	0.012939053	-1.7640325
O1[C@@H](CCC\C=C\C(=O)CC1=O)C	-1.4776767	-0.76367152	-1.2776806
O1CC\C=C/C[C@@H]2[C@@H](CC1=O)CCC2=O	-1.4233081	-0.68217933	-1.1074992
O1[C@@H](C)C(=O)CCCCCCC1=O	-1.4533968	-0.83640671	-1.2657919
O1[C@@H](C)C(=O)CC\C=C/CCC1=O	-1.5170075	-0.60832649	-1.3510424
O1[C@H](CCC)[C@@H](O)[C@@H](O)\C=C\CC[C@H](OC(=O)\C=C\C=C\C)C1=O	-0.53092515	-1.0286188	-1.5801451
O1[C@@H](CC\C=C\C(=O)\C=C/C1=O)C	-1.4869215	-0.75560492	-1.266059
O1C\C=C(\CC\C=C(\CCC1=O)/C)/C	-1.547037	-0.98188704	-1.1152238
O1C\C=C(\C[C@@H](C[C@](O)(C)[C@H](C)[C@@H](C)C1=O)C)/C	-1.1631334	-1.1730063	-1.2919884
O1[C@H](C[C@@H]2O[C@H](C[C@@H](O)C2)Cc2c(C1=O)c(O)ccc2)C\C=C\NC(=O)/C=C\C=C/CC	-0.091985688	-1.3463534	-0.19813366
O1[C@@H](CCCCC[C@H](O)[C@H](O)\C=C\C1=O)C	-1.1146244	-0.66072041	-1.485773
O1[C@@H](CCCCC[C@H](O)[C@@H](O)\C=C/C1=O)C	-1.1146244	-0.66072041	-1.485773
O1[C@H](CCCCC[C@@H](O)C(=O)\C=C/C1=O)C	-1.1839074	-0.77925378	-1.3672585
O1[C@H](CCCCCC(=O)c2c(CC1=O)cc(O)cc2O)C	-0.88771194	-0.99940687	-0.4087877
O1[C@@](C[C@H](O)[C@@H](CC[C@@H]2O[C@](O)(CC1=O)C[C@H](O[C@@H]1O[C@@H](C)[C@H](OC)[C@@H](C)[C@H]1OC)C2)C)(CC\C=C\C=C\CC)C	0.6509608	-2.191673	-1.2758291
O1[C@H](CC)[C@@](O)(\C=C\C(=O)[C@@H](C[C@H](C)[C@H](O[C@H]2O[C@@H](CC(N(C)C)[C@H]2O)C)[C@@H](C)C1=O)C)C	-0.001966619	-1.1076781	-1.5600653
O1[C@H]([C@H](O)C)[C@@H](\C=C\C(=O)[C@@H](C[C@H](C)[C@H](O[C@@H]2O[C@@H](C[C@H](N(C)C)[C@H]2O)C)[C@@H](C)C1=O)C)C	0.01451358	-1.0140181	-1.6557032
O1[C@H](/C(=C/C=C/[C@H](C[C@H]2O[C@@H]2[C@@H]([C@@H](O)CC)C)C)/C)[C@H](\C=C\[C@H](OC(=O)C)[C@](O)(CC[C@@H](O)CC1=O)C)C	0.39372656	-1.6928641	-1.5938256

O1[C@@H](C)[C@@H](O)[C@@H](N)[C@@H](O)[C@@H]1O[C@H]1CC[C@@H](CCC[C@@H](CCCNC(=O)[C@@H]1C)CC)C	0.01742956	-1.0436323	-1.2689165
O1[C@H](C\C=C/C(=O)[C@@H](O)[C@@H](O)C[C@H]2O[C@@H]2c2c(C1=O)c(O)cc(OC)c2)C	-0.29570138	-0.57549876	-0.58165306
O1[C@@H](C[C@@H]2O[C@H]2\C=C\[C@H](O)[C@@H](O)\C=C\C[C@@H](CC1=O)C)[C@@H](O)C[C@H](\C=C\C\C=C\C)C	-0.045893408	-1.4481987	-1.3369541
**DOS COMPOUND LIBRARY**	**PC1**	**PC2**	**PC3**
O=C1NCCCC[C@H](NC(=O)[C@@H](N)CCCCNC(=O)CCCc2nnn(c2)[C@H]1Cc1ccccc1)C(OC)=O	0.24811314	0.068257265	0.58580607
O=C1NCCCC[C@H](NC(=O)[C@@H](N)CCCCNC(=O)CCCc2n(nnc2)[C@H]1Cc1ccccc1)C(OC)=O	0.24821968	0.068234496	0.58586806
O=C1NCCCC[C@@H]2NC(=O)[C@@H](NC2=O)CCCCNC(=O)CCCc2nnn(c2)[C@H]1Cc1ccccc1	0.047085293	-0.007600318	1.0117433
O=C1NCCCC[C@@H]2NC(=O)[C@@H](NC2=O)CCCCNC(=O)CCCc2n(nnc2)[C@H]1Cc1ccccc1	0.04719184	-0.007623088	1.0118053
O=C1NCCCC[C@H](NC(=O)[C@@H](N)CCCNC(=O)CCc2nnn(c2)[C@H]1Cc1ccccc1)C(OC)=O	0.15436238	0.30244216	0.5365209
O=C1NCCCC[C@H](NC(=O)[C@@H](N)CCCNC(=O)CCc2n(nnc2)[C@H]1Cc1ccccc1)C(OC)=O	0.15446892	0.30241939	0.53658289
O=C1NCCCC[C@@H]2NC(=O)[C@@H](NC2=O)CCCNC(=O)CCc2nnn(c2)[C@H]1Cc1ccccc1	-0.042385653	0.2259711	0.95758951
O=C1NCCCC[C@@H]2NC(=O)[C@@H](NC2=O)CCCNC(=O)CCc2n(nnc2)[C@H]1Cc1ccccc1	-0.042279106	0.22594833	0.95765144
O=C1NCCCC[C@H](NC(=O)[C@@H](N)CCCNC(=O)CCCc2nnn(c2)[C@H]1Cc1ccccc1)C(OC)=O	0.20108035	0.18537183	0.56135368
O=C1NCCCC[C@H](NC(=O)[C@@H](N)CCCNC(=O)CCCc2n(nnc2)[C@H]1Cc1ccccc1)C(OC)=O	0.20118691	0.18534905	0.56141567
O=C1NCCCC[C@@H]2NC(=O)[C@@H](NC2=O)CCCNC(=O)CCCc2nnn(c2)[C@H]1Cc1ccccc1	0.002175333	0.10920988	0.98487771
O=C1NCCCC[C@@H]2NC(=O)[C@@H](NC2=O)CCCNC(=O)CCCc2n(nnc2)[C@H]1Cc1ccccc1	0.00228188	0.10918711	0.98493963
O=C1NCCCC[C@H](NC(=O)[C@@H](N)CCCCNC(=O)NCc2nnn(c2)[C@H]1Cc1ccccc1)C(OC)=O	0.2564576	0.3840912	0.62051564
O=C1NCCCC[C@H](NC(=O)[C@@H](N)CCCCNC(=O)NCc2n(nnc2)[C@H]1Cc1ccccc1)C(OC)=O	0.25656417	0.38406843	0.62057757

O=C1NCCCC[C@@H]2NC(=O)[C@@H](NC2=O)CCCCNC(=O)NCc2n(nnc2)[C@H]1Cc1ccccc1	0.05908246	0.30770487	1.0424241
O=C1NCCCC[C@H](NC(=O)[C@@H](N)CCCNC(=O)NCc2nnn(c2)[C@H]1Cc1ccccc1)C(OC)=O	0.20995161	0.50113326	0.59539074
O=C1NCCCC[C@H](NC(=O)[C@@H](N)CCCNC(=O)NCc2n(nnc2)[C@H]1Cc1ccccc1)C(OC)=O	0.21005815	0.50111049	0.59545267
O=C1NCCCC[C@@H]2NC(=O)[C@@H](NC2=O)CCCNC(=O)NCc2n(nnc2)[C@H]1Cc1ccccc1	0.014723856	0.42443934	1.01485
O=C1NCCCC[C@H](NC(=O)[C@@H](N)Cc2ccc(NC(=O)CCc3n(nnc3)[C@H]1Cc1ccccc1)cc2)C(OC)=O	0.34129611	-0.10761018	1.5972095
O=C1NCCCC[C@@H]2NC(=O)[C@@H](NC2=O)Cc2ccc(NC(=O)CCc3n(nnc3)[C@H]1Cc1ccccc1)cc2	0.15282303	-0.18526225	2.0087402
O=C1NCCCC[C@H](NC(=O)[C@@H](N)CCNC(=O)CCCc2nnn(c2)[C@H]1Cc1ccccc1)C(OC)=O	0.15436238	0.30244216	0.5365209
O=C1NCCCC[C@H](NC(=O)[C@@H](N)CCNC(=O)CCCc2n(nnc2)[C@H]1Cc1ccccc1)C(OC)=O	0.15446892	0.30241939	0.53658289
O=C1NCCCC[C@@H]2NC(=O)[C@@H](NC2=O)CCNC(=O)CCCc2nnn(c2)[C@H]1Cc1ccccc1	-0.042385653	0.2259711	0.95758951
O=C1NCCCC[C@@H]2NC(=O)[C@@H](NC2=O)CCNC(=O)CCCc2n(nnc2)[C@H]1Cc1ccccc1	-0.042279106	0.22594833	0.95765144
O=C1NCCCC[C@H](NC(=O)[C@@H](N)CCNC(=O)CCc2nnn(c2)[C@H]1Cc1ccccc1)C(OC)=O	0.10797069	0.4194667	0.51129252
O=C1NCCCC[C@H](NC(=O)[C@@H](N)CCNC(=O)CCc2n(nnc2)[C@H]1Cc1ccccc1)C(OC)=O	0.10807724	0.41944394	0.51135445
O=C1NCCCC[C@@H]2NC(=O)[C@@H](NC2=O)CCNC(=O)CCc2n(nnc2)[C@H]1Cc1ccccc1	-0.086480334	0.34265915	0.92992556
O=C1NCCCC[C@H](NC(=O)[C@@H](N)CNC(=O)CCCc2nnn(c2)[C@H]1Cc1ccccc1)C(OC)=O	0.10797069	0.4194667	0.51129252
O=C1NCCCC[C@H](NC(=O)[C@@H](N)CNC(=O)CCCc2n(nnc2)[C@H]1Cc1ccccc1)C(OC)=O	0.10807724	0.41944394	0.51135445
O=C1NCCCC[C@@H]2NC(=O)[C@@H](NC2=O)CNC(=O)CCCc2nnn(c2)[C@H]1Cc1ccccc1	-0.086586878	0.34268191	0.92986357
O=C1NCCCC[C@@H]2NC(=O)[C@@H](NC2=O)CNC(=O)CCCc2n(nnc2)[C@H]1Cc1ccccc1	-0.086480334	0.34265915	0.92992556
O=C1NCCCC[C@H](NC(=O)[C@@H](N)CNC(=O)CCc2nnn(c2)[C@H]1Cc1ccccc1)C(OC)=O	0.0619171	0.53644395	0.48565236
O=C1NCCCC[C@H](NC(=O)[C@@H](N)CNC(=O)CCc2n(nnc2)[C@H]1Cc1ccccc1)C(OC)=O	0.062023625	0.53642118	0.48571429
O=C1NCCCC[C@@H]2NC(=O)[C@@H](NC2=O)CNC(=O)CCc2n(nnc2)[C@H]1Cc1ccccc1	-0.13031098	0.45931816	0.90174609
O=C1NCCCC[C@H](NC(=O)[C@@H](N)CCNC(=O)NCc2nnn(c2)[C@H]1Cc1ccccc1)C(OC)=O	0.16377425	0.61812925	0.56986445
O=C1NCCCC[C@H](NC(=O)[C@@H](N)CCNC(=O)NCc2n(nnc2)[C@H]1Cc1ccccc1)C(OC)=O	0.1638808	0.61810648	0.56992644

O=C1NCCCC[C@@H]2NC(=O)[C@@H](NC2=O)CCNC(=O)NCc2nnn(c2)[C@H]1Cc1ccccc1	-0.029274285	0.54112345	0.98683387
O=C1NCCCC[C@@H]2NC(=O)[C@@H](NC2=O)CCNC(=O)NCc2n(nnc2)[C@H]1Cc1ccccc1	0.11793721	0.7350778	0.5439207
O=C1NCCCC[C@H](NC(=O)[C@@H](N)CNC(=O)NCc2nnn(c2)[C@H]1Cc1ccccc1)C(OC)=O	0.11804377	0.73505503	0.54398263
O=C1NCCCC[C@H](NC(=O)[C@@H](N)CNC(=O)NCc2n(nnc2)[C@H]1Cc1ccccc1)C(OC)=O	-0.073008098	0.65777868	0.95829785
O=C1NCCCC[C@@H]2NC(=O)[C@@H](NC2=O)CNC(=O)NCc2nnn(c2)[C@H]1Cc1ccccc1	-0.072901577	0.65775591	0.95835978
O=C1NCCCC[C@@H]2NC(=O)[C@@H](NC2=O)CNC(=O)NCc2n(nnc2)[C@H]1Cc1ccccc1	0.38631293	-0.22442926	1.6237961
O=C1NCCCC[C@H](NC(=O)[C@@H](N)Cc2ccc(NC(=O)CCCc3nnn(c3)[C@H]1Cc1ccccc1)cc2)C(OC)=O	0.38641948	-0.22445203	1.6238581
O=C1NCCCC[C@H](NC(=O)[C@@H](N)Cc2ccc(NC(=O)CCCc3n(nnc3)[C@H]1Cc1ccccc1)cc2)C(OC)=O	0.19610819	-0.30184108	2.0374911
O=C1NCCCC[C@@H]2NC(=O)[C@@H](NC2=O)Cc2ccc(NC(=O)CCCc3n(nnc3)[C@H]1Cc1ccccc1)cc2	0.10797069	0.4194667	0.51129252
O=C1NCCCC[C@H](NC(=O)CCNC(=O)[C@@H](N)CCNC(=O)CCCc2n(nnc2)[C@H]1Cc1ccccc1)C(OC)=O	0.10807724	0.41944394	0.51135445
O=C1NCCCC[C@H](NC(=O)CCNC(=O)[C@@H](N)CCNC(=O)CCc2n(nnc2)[C@H]1Cc1ccccc1)C(OC)=O	-0.086586878	0.34268191	0.92986357
O=C1NC[C@H](NC(=O)[C@@H](N)CCCCNC(=O)CCCc2nnn(c2)[C@H]1Cc1ccccc1)C(OC)=O	-0.086480334	0.34265915	0.92992556
O=C1NC[C@H](NC(=O)[C@@H](N)CCCCNC(=O)CCCc2n(nnc2)[C@H]1Cc1ccccc1)C(OC)=O	0.016213611	0.65337223	0.45958358
O=C1NC[C@@H]2NC(=O)[C@@H](NC2=O)CCCCNC(=O)CCCc2nnn(c2)[C@H]1Cc1ccccc1	0.01632016	0.65334946	0.45964554
O=C1NC[C@@H]2NC(=O)[C@@H](NC2=O)CCCCNC(=O)CCCc2n(nnc2)[C@H]1Cc1ccccc1	-0.17386697	0.57594681	0.87303478
O=C1NC[C@H](NC(=O)[C@@H](N)CCCNC(=O)CCc2nnn(c2)[C@H]1Cc1ccccc1)C(OC)=O	-0.17376043	0.57592404	0.8730967
O=C1NC[C@H](NC(=O)[C@@H](N)CCCNC(=O)CCc2n(nnc2)[C@H]1Cc1ccccc1)C(OC)=O	0.0619171	0.53644395	0.48565236
O=C1NC[C@@H]2NC(=O)[C@@H](NC2=O)CCCNC(=O)CCc2nnn(c2)[C@H]1Cc1ccccc1	0.062023625	0.53642118	0.48571429
O=C1NC[C@@H]2NC(=O)[C@@H](NC2=O)CCCNC(=O)CCc2n(nnc2)[C@H]1Cc1ccccc1	-0.13041754	0.45934093	0.90168411
O=C1NC[C@H](NC(=O)[C@@H](N)CCCNC(=O)CCCc2nnn(c2)[C@H]1Cc1ccccc1)C(OC)=O	-0.13031098	0.45931816	0.90174609
O=C1NC[C@H](NC(=O)[C@@H](N)CCCNC(=O)CCCc2n(nnc2)[C@H]1Cc1ccccc1)C(OC)=O	0.11793721	0.7350778	0.5439207
O=C1NC[C@@H]2NC(=O)[C@@H](NC2=O)CCCNC(=O)CCCc2nnn(c2)[C@H]1Cc1ccccc1	0.11804377	0.73505503	0.54398263

O=C1NC[C@@H]2NC(=O)[C@@H](NC2=O)CCCNC(=O)CCCc2n(nnc2)[C@H]1Cc1ccccc1	-0.073008098	0.65777868	0.95829785
O=C1NC[C@H](NC(=O)[C@@H](N)CCCCNC(=O)NCc2nnn(c2)[C@H]1Cc1ccccc1)C(OC)=O	-0.072901577	0.65775591	0.95835978
O=C1NC[C@H](NC(=O)[C@@H](N)CCCCNC(=O)NCc2n(nnc2)[C@H]1Cc1ccccc1)C(OC)=O	0.072452165	0.85197729	0.51754248
O=C1NC[C@@H]2NC(=O)[C@@H](NC2=O)CCCCNC(=O)NCc2nnn(c2)[C@H]1Cc1ccccc1	0.072558716	0.85195452	0.51760447
O=C1NC[C@@H]2NC(=O)[C@@H](NC2=O)CCCCNC(=O)NCc2n(nnc2)[C@H]1Cc1ccccc1	-0.11625445	0.77435821	0.92934954
O=C1NC[C@H](NC(=O)[C@@H](N)CCCNC(=O)NCc2nnn(c2)[C@H]1Cc1ccccc1)C(OC)=O	0.016213611	0.65337223	0.45958358
O=C1NC[C@H](NC(=O)[C@@H](N)CCCNC(=O)NCc2n(nnc2)[C@H]1Cc1ccccc1)C(OC)=O	0.01632016	0.65334946	0.45964554
O=C1NC[C@@H]2NC(=O)[C@@H](NC2=O)CCCNC(=O)NCc2nnn(c2)[C@H]1Cc1ccccc1	-0.17376043	0.57592404	0.8730967
O=C1NC[C@@H]2NC(=O)[C@@H](NC2=O)CCCNC(=O)NCc2n(nnc2)[C@H]1Cc1ccccc1	-0.029127406	0.7702499	0.43306863
O=C1NC[C@H](NC(=O)[C@@H](N)CCNC(=O)CCCc2nnn(c2)[C@H]1Cc1ccccc1)C(OC)=O	-0.029020857	0.77022713	0.43313056
O=C1NC[C@H](NC(=O)[C@@H](N)CCNC(=O)CCCc2n(nnc2)[C@H]1Cc1ccccc1)C(OC)=O	-0.21692467	0.69249821	0.84389853
O=C1NC[C@@H]2NC(=O)[C@@H](NC2=O)CCNC(=O)CCCc2nnn(c2)[C@H]1Cc1ccccc1	-0.21681811	0.69247544	0.84396046
O=C1NC[C@@H]2NC(=O)[C@@H](NC2=O)CCNC(=O)CCCc2n(nnc2)[C@H]1Cc1ccccc1	-0.029127406	0.7702499	0.43306863
O=C1NC[C@H](NC(=O)[C@@H](N)CCNC(=O)CCc2nnn(c2)[C@H]1Cc1ccccc1)C(OC)=O	-0.029020857	0.77022713	0.43313056
O=C1NC[C@H](NC(=O)[C@@H](N)CCNC(=O)CCc2n(nnc2)[C@H]1Cc1ccccc1)C(OC)=O	-0.074093565	0.88707548	0.40608901
O=C1NC[C@@H]2NC(=O)[C@@H](NC2=O)CCNC(=O)CCc2nnn(c2)[C@H]1Cc1ccccc1	-0.073987022	0.88705271	0.40615097
O=C1NC[C@@H]2NC(=O)[C@@H](NC2=O)CCNC(=O)CCc2n(nnc2)[C@H]1Cc1ccccc1	-0.25958046	0.80899388	0.81425786
O=C1NC[C@H](NC(=O)[C@@H](N)CNC(=O)CCCc2nnn(c2)[C@H]1Cc1ccccc1)C(OC)=O	-0.25947392	0.80897111	0.81431979
O=C1NC[C@H](NC(=O)[C@@H](N)CNC(=O)CCCc2n(nnc2)[C@H]1Cc1ccccc1)C(OC)=O	0.027331058	0.96882612	0.49071223
O=C1NC[C@H](NC(=O)[C@@H](N)CNC(=O)CCc2nnn(c2)[C@H]1Cc1ccccc1)C(OC)=O	0.027437607	0.96880335	0.49077418
O=C1NC[C@H](NC(=O)[C@@H](N)CNC(=O)CCc2n(nnc2)[C@H]1Cc1ccccc1)C(OC)=O	-0.017414143	1.0856229	0.46341133
O=C1NC[C@@H]2NC(=O)[C@@H](NC2=O)CNC(=O)CCc2nnn(c2)[C@H]1Cc1ccccc1	-0.017307594	1.0856	0.46347329

O=C1NC[C@@H]2NC(=O)[C@@H](NC2=O)CNC(=O)CCc2n(nnc2)[C@H]1Cc1ccccc1	-0.20145877	1.0073311	0.87002373
O=C1NC[C@H](NC(=O)[C@@H](N)CCNC(=O)NCc2nnn(c2)[C@H]1Cc1ccccc1)C(OC)=O	0.25197065	0.1259447	1.5427848
O=C1NC[C@H](NC(=O)[C@@H](N)CCNC(=O)NCc2n(nnc2)[C@H]1Cc1ccccc1)C(OC)=O	0.0619171	0.53644395	0.48565236
O=C1NC[C@@H]2NC(=O)[C@@H](NC2=O)CCNC(=O)NCc2nnn(c2)[C@H]1Cc1ccccc1	0.062023625	0.53642118	0.48571429
O=C1NC[C@@H]2NC(=O)[C@@H](NC2=O)CCNC(=O)NCc2n(nnc2)[C@H]1Cc1ccccc1	-0.13041754	0.45934093	0.90168411
O=C1NC[C@H](NC(=O)[C@@H](N)CNC(=O)NCc2nnn(c2)[C@H]1Cc1ccccc1)C(OC)=O	-0.13031098	0.45931816	0.90174609
O=C1NC[C@H](NC(=O)[C@@H](N)CNC(=O)NCc2n(nnc2)[C@H]1Cc1ccccc1)C(OC)=O	0.69852942	0.022557393	-0.54421753
O=C1NC[C@@H]2NC(=O)[C@@H](NC2=O)CNC(=O)NCc2nnn(c2)[C@H]1Cc1ccccc1	0.60173368	0.25719118	-0.59034264
O=C1NC[C@@H]2NC(=O)[C@@H](NC2=O)CNC(=O)NCc2n(nnc2)[C@H]1Cc1ccccc1	0.60184026	0.25716841	-0.59028065
O=C1NC[C@H](NC(=O)[C@@H](N)Cc2ccc(NC(=O)CCCc3n(nnc3)[C@H]1Cc1ccccc1)cc2)C(OC)=O	0.64995313	0.13990319	-0.56715852
O=C1NC[C@H](NC(=O)[C@@H](N)CCCCNC(=O)CCc2nnn(c2)[C@H]1Cc1ccccc1)C(OC)=O	0.65005964	0.13988042	-0.56709659
O=C1NC[C@H](NC(=O)[C@@H](N)CCCCNC(=O)CCc2n(nnc2)[C@H]1Cc1ccccc1)C(OC)=O	0.70421034	0.33877948	-0.50663412
O=C1NC[C@@H]2NC(=O)[C@@H](NC2=O)CCCCNC(=O)CCc2nnn(c2)[C@H]1Cc1ccccc1	0.70431691	0.33875671	-0.50657219
O=C1NC[C@@H]2NC(=O)[C@@H](NC2=O)CCCCNC(=O)CCc2n(nnc2)[C@H]1Cc1ccccc1	0.65617806	0.4560425	-0.53007632
O=C1N[C@@H](C)C(=O)N[C@@H](CCCCNC(=O)[C@@H](n2nncc2CCCC(=O)NCC[C@H](NC(OC(C)(C)C)=O)C(=O)N[C@H]1Cc1ccccc1)Cc1ccccc1)C(OC)=O	0.65628463	0.45601973	-0.5300144
O=C1N[C@@H](C)C(=O)N[C@@H](CCCCNC(=O)[C@@H](n2nncc2CCCC(=O)NC[C@H](NC(OC(C)(C)C)=O)C(=O)N[C@H]1Cc1ccccc1)Cc1ccccc1)C(OC)=O	0.7807368	-0.15173836	0.47970814
O=C1NCCC(=O)N[C@@H](CCCCNC(=O)[C@H](n2nncc2CCC(=O)NCC[C@H](NC(OC(C)(C)C)=O)C(=O)N[C@H]1Cc1ccccc1)Cc1ccccc1)C(OC)=O	0.60184026	0.25716841	-0.59028065
O=C1N[C@@H](CCC(=O)N[C@@H](CCCCn2nncc2CCCC(=O)NCCCC[C@@H]1NC(OC(C)(C)C)=O)C(OC)=O)C(OC)=O	0.55387986	0.37442017	-0.61378133
O=C1N[C@@H](CCC(=O)N[C@@H](CCCCn2nnc(CCC(=O)NCCC[C@@H]1NC(OC(C)(C)C)=O)c2)C(OC)=O)C(OC)=O	0.55377328	0.37444293	-0.61384326
O=C1N[C@@H](CCC(=O)N[C@@H](CCCCn2nncc2CCC(=O)NCCC[C@@H]1NC(OC(C)(C)C)=O)C(OC)=O)C(OC)=O	0.55387986	0.37442017	-0.61378133

O=C1N[C@@H](CCC(=O)N[C@@H](CCCCn2nnc(CCCC(=O)NCCC[C@@H]1NC(OC(C)(C)C)=O)c2)C(OC)=O)C(OC)=O	0.50608093	0.49165723	-0.63767248
O=C1N[C@@H](CCC(=O)N[C@@H](CCCCn2nncc2CCCC(=O)NCCC[C@@H]1NC(OC(C)(C)C)=O)C(OC)=O)C(OC)=O	0.5061875	0.49163446	-0.63761055
O=C1N[C@@H](CCC(=O)N[C@@H](CCCCn2nnc(CNC(=O)NCCCC[C@@H]1NC(OC(C)(C)C)=O)c2)C(OC)=O)C(OC)=O	0.60840744	0.57326901	-0.55384022
O=C1N[C@@H](CCC(=O)N[C@@H](CCCCn2nncc2CNC(=O)NCCCC[C@@H]1NC(OC(C)(C)C)=O)C(OC)=O)C(OC)=O	0.60851395	0.57324624	-0.55377829
O=C1N[C@@H](CCC(=O)N[C@@H](CCCCn2nnc(CNC(=O)NCCC[C@@H]1NC(OC(C)(C)C)=O)c2)C(OC)=O)C(OC)=O	0.56090724	0.69045776	-0.5779379
O=C1N[C@@H](CCC(=O)N[C@@H](CCCCn2nncc2CNC(=O)NCCC[C@@H]1NC(OC(C)(C)C)=O)C(OC)=O)C(OC)=O	0.56101382	0.69043499	-0.57787597
O=C1N[C@@H](CCC(=O)N[C@@H](CCCCn2nncc2CCC(=O)Nc2ccc(C[C@@H]1NC(OC(C)(C)C)=O)cc2)C(OC)=O)C(OC)=O	0.82724375	-0.26877284	0.50464481
O=C1N[C@@H](CCC(=O)N[C@@H](CCCCn2nncc2CCCC(=O)NCC[C@@H]1NC(OC(C)(C)C)=O)C(OC)=O)C(OC)=O	0.82735032	-0.26879561	0.50470674
O=C1N[C@@H](CCC(=O)N[C@@H](CCCCn2nncc2CCC(=O)NCC[C@@H]1NC(OC(C)(C)C)=O)C(OC)=O)C(OC)=O	0.64995313	0.13990319	-0.56715852
O=C1N[C@@H](CCC(=O)N[C@@H](CCCCn2nnc(CCCC(=O)NC[C@@H]1NC(OC(C)(C)C)=O)c2)C(OC)=O)C(OC)=O	0.65005964	0.13988042	-0.56709659
O=C1N[C@@H](CCC(=O)N[C@@H](CCCCn2nncc2CCCC(=O)NC[C@@H]1NC(OC(C)(C)C)=O)C(OC)=O)C(OC)=O	-0.3582198	0.38549411	0.55179918
O=C1N[C@@H](CCC(=O)N[C@@H](CCCCn2nnc(CCC(=O)NC[C@@H]1NC(OC(C)(C)C)=O)c2)C(OC)=O)C(OC)=O	-0.31338629	0.26869094	0.57884723
O=C1N[C@@H](CCC(=O)N[C@@H](CCCCn2nncc2CCC(=O)NC[C@@H]1NC(OC(C)(C)C)=O)C(OC)=O)C(OC)=O	-0.30139366	0.58401531	0.60908031
O=C1N[C@@H](CCC(=O)N[C@@H](CCCCn2nnc(CNC(=O)NCC[C@@H]1NC(OC(C)(C)C)=O)c2)C(OC)=O)C(OC)=O	-0.3582198	0.38549411	0.55179918
O=C1N[C@@H](CCC(=O)N[C@@H](CCCCn2nncc2CNC(=O)NCC[C@@H]1NC(OC(C)(C)C)=O)C(OC)=O)C(OC)=O	-0.40262392	0.50223738	0.5242222
O=C1N[C@@H](CCC(=O)N[C@@H](CCCCn2nnc(CNC(=O)NC[C@@H]1NC(OC(C)(C)C)=O)c2)C(OC)=O)C(OC)=O	-0.44658321	0.61891878	0.49609238
O=C1N[C@@H](CCC(=O)N[C@@H](CCCCn2nncc2CNC(=O)NC[C@@H]1NC(OC(C)(C)C)=O)C(OC)=O)C(OC)=O	-0.34565976	0.70074946	0.58108586
O=C1N[C@@H](CCC(=O)N[C@@H](CCCCn2nnc(CCCC(=O)Nc3ccc(C[C@@H]1NC(OC(C)(C)C)=O)cc3)c2)C(OC)=O)C(OC)=O	-0.38926807	0.81737649	0.55265558
O=C1N[C@@H](CCC(=O)N[C@@H](CCCCn2nncc2CCCC(=O)Nc2ccc(C[C@@H]1NC(OC(C)(C)C)=O)cc2)C(OC)=O)C(OC)=O	-0.31338629	0.26869094	0.57884723
O=C1N[C@@H](CCC(=O)N[C@@H](CCCCn2nnc(CCC(=O)NCCCC[C@@H]1NC(OC(C)(C)C)=O)c2)C(OC)=O)C(OC)=O	-0.40272149	0.34650555	-0.40710318
O=C1N[C@@H](CCC(=O)N[C@@H](CCCCn2nncc2CCC(=O)NCCCC[C@@H]1NC(OC(C)(C)C)=O)C(OC)=O)C(OC)=O	-0.40261495	0.34648278	-0.40704125
O=C1N[C@@H](Cc2ccc(-n3nncc3CCC(=O)NCCC[C@@H]1N)cc2)C(OC)=O	-0.60722136	0.27113587	0.023022361

O=C1N[C@@H](Cc2ccc(-n3nncc3CCCC(=O)NCCC[C@@H]1N)cc2)C(OC)=O	-0.4983004	0.5809564	-0.45440438
O=C1N[C@@H](Cc2ccc(-n3nncc3CNC(=O)NCCC[C@@H]1N)cc2)C(OC)=O	-0.49819386	0.58093363	-0.45434245
O=C1N[C@@H](Cc2ccc(-n3nncc3CCCC(=O)NCC[C@@H]1N)cc2)C(OC)=O	-0.69676793	0.50472921	-0.031315621
O=C1N[C@@H](Cc2ccc(-n3nncc3CCC(=O)NCC[C@@H]1N)cc2)C(OC)=O	-0.45072392	0.46376085	-0.43049601
O=C1N[C@@H](Cc2ccc(-n3nnc(CNC(=O)NCC[C@@H]1N)c3)cc2)C(OC)=O	-0.45061737	0.46373808	-0.43043408
O=C1N[C@@H](Cc2ccc(-n3nncc3CNC(=O)NC[C@@H]1N)cc2)C(OC)=O	-0.65229815	0.38797906	-0.003874723
O=C1N[C@@H](Cc2ccc(-n3nncc3CCC(=O)NCCCC[C@@H]1N)cc2)C(OC)=O	-0.3959946	0.66257566	-0.37065896
O=C1N[C@@H](CCCCn2nnc(CCCC(=O)NCCCC[C@@H]1N)c2)C(OC)=O	-0.44337204	0.77975315	-0.39505053
O=C1N[C@@H](CCCCn2nncc2CCCC(=O)NCCCC[C@@H]1N)C(OC)=O	-0.4432655	0.77973038	-0.3949886
O=C1NCCCC[C@@H]2NC(=O)[C@@H](NC2=O)CCCCn2nncc2CCC1	-0.31301522	0.17112637	0.60850126
O=C1N[C@@H](CCCCn2nnc(CCC(=O)NCCC[C@@H]1N)c2)C(OC)=O	-0.4983004	0.5809564	-0.45440438
O=C1N[C@@H](CCCCn2nncc2CCC(=O)NCCC[C@@H]1N)C(OC)=O	-0.49819386	0.58093363	-0.45434245
O=C1NCCC[C@@H]2NC(=O)[C@@H](NC2=O)CCCCn2nnc(CC1)c2	-0.69666141	0.50470644	-0.031253681
O=C1N[C@@H](CCCCn2nnc(CCCC(=O)NCCC[C@@H]1N)c2)C(OC)=O	-0.54542726	0.69808894	-0.47885954
O=C1N[C@@H](CCCCn2nncc2CCCC(=O)NCCC[C@@H]1N)C(OC)=O	-0.54532069	0.69806617	-0.47879761
O=C1NCCC[C@@H]2NC(=O)[C@@H](NC2=O)CCCCn2nnc(CCC1)c2	-0.74071497	0.62140614	-0.05939351
O=C1N[C@@H](CCCCn2nncc2CNC(=O)NCCCC[C@@H]1N)C(OC)=O	-0.74060839	0.62138337	-0.05933157
O=C1N[C@@H](CCCCn2nnc(CNC(=O)NCCC[C@@H]1N)c2)C(OC)=O	-0.54532069	0.69806617	-0.47879761
O=C1N[C@@H](CCCCn2nncc2CNC(=O)NCCC[C@@H]1N)C(OC)=O	-0.74060839	0.62138337	-0.05933157
O=C1N[C@@H](CCCCn2nncc2CCC(=O)Nc2ccc(C[C@@H]1N)cc2)C(OC)=O	-0.5920794	0.81515503	-0.50389498
O=C1N[C@@H](CCCCn2nnc(CCCC(=O)NCC[C@@H]1N)c2)C(OC)=O	-0.59197289	0.81513226	-0.503833
O=C1N[C@@H](CCCCn2nncc2CCCC(=O)NCC[C@@H]1N)C(OC)=O	-0.49018738	0.89684427	-0.41993961
O=C1NCC[C@@H]2NC(=O)[C@@H](NC2=O)CCCCn2nncc2CCC1	-0.49008083	0.8968215	-0.41987768
O=C1N[C@@H](CCCCn2nnc(CCC(=O)NCC[C@@H]1N)c2)C(OC)=O	-0.68342686	0.81987202	-0.002903873
O=C1N[C@@H](CCCCn2nncc2CCC(=O)NCC[C@@H]1N)C(OC)=O	-0.53652281	1.0138686	-0.44542193
O=C1NCC[C@@H]2NC(=O)[C@@H](NC2=O)CCCCn2nnc(CC1)c2	-0.53641623	1.0138458	-0.44535998

O=C1NCC[C@@H]2NC(=O)[C@@H](NC2=O)CCCCn2nncc2CC1	-0.26776776	0.054265343	0.63504297
O=C1N[C@@H](CCCCn2nncc2CCCC(=O)NC[C@@H]1N)C(OC)=O	-0.45712849	-0.023261616	1.0476351
O=C1NC[C@@H]2NC(=O)[C@@H](NC2=O)CCCCn2nncc2CCC1	-0.45072392	0.46376085	-0.43049601
O=C1N[C@@H](CCCCn2nnc(CCC(=O)NC[C@@H]1N)c2)C(OC)=O	-0.45061737	0.46373808	-0.43043408
O=C1N[C@@H](CCCCn2nncc2CCC(=O)NC[C@@H]1N)C(OC)=O	-0.65229815	0.38797906	-0.003874723
O=C1N[C@@H](CCCCn2nnc(CNC(=O)NCC[C@@H]1N)c2)C(OC)=O	-0.65219164	0.38795629	-0.003812783
O=C1N[C@@H](CCCCn2nncc2CNC(=O)NCC[C@@H]1N)C(OC)=O	-0.45072392	0.46376085	-0.43049601
O=C1N[C@@H](CCCCn2nnc(CNC(=O)NC[C@@H]1N)c2)C(OC)=O	-0.45061737	0.46373808	-0.43043408
O=C1N[C@@H](CCCCn2nncc2CNC(=O)NC[C@@H]1N)C(OC)=O	-0.65229815	0.38797906	-0.003874723
O=C1N[C@@H](CCCCn2nncc2CCCC(=O)Nc2ccc(C[C@@H]1N)cc2)C(OC)=O	-0.65219164	0.38795629	-0.003812783
O=C1Nc2ccc(C[C@@H]3NC(=O)[C@@H](NC3=O)CCCCn3nncc3CCC1)cc2	-0.54542726	0.69808894	-0.47885954
O=C1N[C@@H](CCCCn2nnc(CCC(=O)NCCCC[C@@H]1N)c2)C(OC)=O	-0.54532069	0.69806617	-0.47879761
O=C1N[C@@H](CCCCn2nncc2CCC(=O)NCCCC[C@@H]1N)C(OC)=O	-0.4983004	0.5809564	-0.45440438
O=C1NCCCC[C@@H]2NC(=O)[C@@H](NC2=O)CCCCn2nnc(CC1)c2	-0.49819386	0.58093363	-0.45434245
O=C1NCCCC[C@@H]2NC(=O)[C@@H](NC2=O)CCCCn2nncc2CC1	-0.69666141	0.50470644	-0.031253681
O=C1N[C@@H](CCCn2nnc(CCCC(=O)NCCCC[C@@H]1N)c2)C(OC)=O	-0.44337204	0.77975315	-0.39505053
O=C1N[C@@H](CCCn2nncc2CCCC(=O)NCCCC[C@@H]1N)C(OC)=O	-0.4432655	0.77973038	-0.3949886
O=C1NCCCC[C@@H]2NC(=O)[C@@H](NC2=O)CCCn2nnc(CCC1)c2	-0.49018738	0.89684427	-0.41993961
O=C1NCCCC[C@@H]2NC(=O)[C@@H](NC2=O)CCCn2nncc2CCC1	-0.49008083	0.8968215	-0.41987768
O=C1N[C@@H](CCCn2nnc(CCC(=O)NCCC[C@@H]1N)c2)C(OC)=O	-0.35784873	0.28792951	0.5814532
O=C1N[C@@H](CCCn2nncc2CCC(=O)NCCC[C@@H]1N)C(OC)=O	-0.54220092	0.20968623	0.98830926
O=C1N[C@@H](CCCn2nnc(CCCC(=O)NCCC[C@@H]1N)c2)C(OC)=O	-0.54542726	0.69808894	-0.47885954
O=C1N[C@@H](CCCn2nncc2CCCC(=O)NCCC[C@@H]1N)C(OC)=O	-0.54532069	0.69806617	-0.47879761
O=C1NCCC[C@@H]2NC(=O)[C@@H](NC2=O)CCCn2nncc2CCC1	-0.74060839	0.62138337	-0.05933157
O=C1N[C@@H](CCCn2nnc(CNC(=O)NCCCC[C@@H]1N)c2)C(OC)=O	-0.5920794	0.81515503	-0.50389498
O=C1N[C@@H](CCCn2nncc2CNC(=O)NCCCC[C@@H]1N)C(OC)=O	-0.59197289	0.81513226	-0.503833
O=C1N[C@@H](CCCn2nnc(CNC(=O)NCCC[C@@H]1N)c2)C(OC)=O	-0.78401023	0.73798424	-0.088079214
O=C1N[C@@H](CCCn2nncc2CNC(=O)NCCC[C@@H]1N)C(OC)=O	-0.5920794	0.81515503	-0.50389498

O=C1N[C@@H](CCCn2nncc2CCC(=O)Nc2ccc(C[C@@H]1N)cc2)C(OC)=O	-0.59197289	0.81513226	-0.503833
O=C1Nc2ccc(C[C@@H]3NC(=O)[C@@H](NC3=O)CCCn3nncc3CC1)cc2	-0.78401023	0.73798424	-0.088079214
O=C1N[C@@H](CCCn2nnc(CCCC(=O)NCC[C@@H]1N)c2)C(OC)=O	-0.63823086	0.93215132	-0.52954662
O=C1N[C@@H](CCCn2nncc2CCCC(=O)NCC[C@@H]1N)C(OC)=O	-0.63812435	0.93212855	-0.52948469
O=C1NCC[C@@H]2NC(=O)[C@@H](NC2=O)CCCn2nncc2CCC1	-0.53652281	1.0138686	-0.44542193
O=C1N[C@@H](CCCn2nnc(CCC(=O)NCC[C@@H]1N)c2)C(OC)=O	-0.53641623	1.0138458	-0.44535998
O=C1N[C@@H](CCCn2nncc2CCC(=O)NCC[C@@H]1N)C(OC)=O	-0.72643363	0.93641281	-0.032016985
O=C1NCC[C@@H]2NC(=O)[C@@H](NC2=O)CCCn2nncc2CC1	-0.58224648	1.1307999	-0.47147179
O=C1N[C@@H](CCCn2nnc(CCCC(=O)NC[C@@H]1N)c2)C(OC)=O	-0.31301522	0.17112637	0.60850126
O=C1N[C@@H](CCCn2nncc2CCCC(=O)NC[C@@H]1N)C(OC)=O	-0.49988762	0.093243361	1.0182478
O=C1NC[C@@H]2NC(=O)[C@@H](NC2=O)CCCn2nncc2CCC1	-0.4983004	0.5809564	-0.45440438
O=C1N[C@@H](CCCn2nnc(CCC(=O)NC[C@@H]1N)c2)C(OC)=O	-0.49819386	0.58093363	-0.45434245
O=C1N[C@@H](CCCn2nncc2CCC(=O)NC[C@@H]1N)C(OC)=O	-0.69666141	0.50470644	-0.031253681
O=C1N[C@@H](CCCn2nnc(CNC(=O)NCC[C@@H]1N)c2)C(OC)=O	-0.25666147	0.46332175	0.63663626
O=C1N[C@@H](CCCn2nncc2CNC(=O)NCC[C@@H]1N)C(OC)=O	-0.31324393	0.26476851	0.57900637
O=C1N[C@H]2CCNC(=O)NCc3n(nnc3)CCC[C@@H]1NC2=O	-0.31324393	0.26476851	0.57900637
O=C1N[C@@H](CCCn2nncc2CNC(=O)NC[C@@H]1N)C(OC)=O	0.69842291	0.022580164	-0.54427946
O=C1N[C@@H](CCCn2nncc2CCCC(=O)Nc2ccc(C[C@@H]1N)cc2)C(OC)=O	0.23767319	0.6933527	-0.60169977
O=C1Nc2ccc(C[C@@H]3NC(=O)[C@@H](NC3=O)CCCn3nncc3CCC1)cc2	0.14101417	0.92796409	-0.64791197
O=C1N[C@@H](CCCn2nnc(CCC(=O)NCCCC[C@@H]1N)c2)C(OC)=O	-0.063761488	0.8526333	-0.21745801
O=C1N[C@@H](CCCn2nncc2CCC(=O)NCCCC[C@@H]1N)C(OC)=O	0.18918927	0.81067997	-0.62461621
O=C1NCCCC[C@@H]2NC(=O)[C@@H](NC2=O)CCCn2nncc2CC1	-0.01794026	0.7356863	-0.19147816
O=C1N[C@@H](CCCn2nnc(CCCC(=O)Nc3ccc(C[C@H](N)C(=O)N[C@@H]1C)cc3)c2)C(OC)=O	0.24340335	1.0095731	-0.56430316
O=C1N[C@@H](CCCn2nncc2CCCC(=O)Nc2ccc(C[C@H](N)C(=O)N[C@@H]1C)cc2)C(OC)=O	0.19545785	1.1268268	-0.58791894
O=C1N[C@H](C)C(=O)N[C@H](CCCn2nncc2CCCC(=O)Nc2ccc(C[C@@H](N)C(=O)N[C@H]1Cc1ccccc1)cc2)C(OC)=O	0.32163292	0.5188095	0.42014155
O=C1N[C@@H](Cc2cc(-n3nncc3CNC(=O)NCCCC[C@@H]1N)ccc2)C(OC)=O	0.14101417	0.92796409	-0.64791197
O=C1N[C@@H](Cc2cc(-n3nncc3CCCC(=O)NCCC[C@@H]1N)ccc2)C(OC)=O	0.093161009	1.0452033	-0.67160493

O=C1N[C@@H](Cc2cc(-n3nncc3CCC(=O)NCCCC[C@@H]1N)ccc2)C(OC)=O	0.093161009	1.0452033	-0.67160493
O=C1N[C@@H](CCC(=O)N[C@@H](CCCn2nncc2CCCC(=O)NCCCC[C@@H]1N)C(OC)=O)C(OC)=O	0.045643277	1.162396	-0.69571394
O=C1N[C@@H](CCC(=O)N[C@@H](CCCn2nncc2CCC(=O)NCCC[C@@H]1N)C(OC)=O)C(OC)=O	0.14783782	1.2440351	-0.61193961
O=C1N[C@@H](CCCn2nncc2CCC(=O)NCCC[C@@H]2NC(=O)[C@@H](NC2=O)CC1)C(OC)=O	0.1004501	1.3612193	-0.6364463
O=C1N[C@@H](CCC(=O)N[C@@H](CCCn2nncc2CCCC(=O)NCCC[C@@H]1N)C(OC)=O)C(OC)=O	0.10055664	1.3611965	-0.63638437
O=C1N[C@@H](CCCn2nncc2CCCC(=O)NCCC[C@@H]2NC(=O)[C@@H](NC2=O)CC1)C(OC)=O	0.13554282	0.64209908	-0.592893
O=C1N[C@@H](CCC(=O)N[C@@H](CCCn2nncc2CNC(=O)NCCCC[C@@H]1N)C(OC)=O)C(OC)=O	0.13564937	0.64207631	-0.59283108
O=C1N[C@@H](CCC(=O)N[C@@H](CCCn2nncc2CNC(=O)NCCC[C@@H]1N)C(OC)=O)C(OC)=O	-0.01794026	0.7356863	-0.19147816
O=C1N[C@@H](CCC(=O)N[C@@H](CCCn2nncc2CCC(=O)Nc2ccc(C[C@@H]1N)cc2)C(OC)=O)C(OC)=O	0.031486288	0.38723004	0.58770502
O=C1N[C@@H](CCC(=O)N[C@@H](CCCn2nncc2CCCC(=O)NCC[C@@H]1N)C(OC)=O)C(OC)=O	0.031592838	0.38720727	0.58776695
O=C1N[C@@H](CCC(=O)N[C@@H](CCCn2nncc2CCC(=O)NCC[C@@H]1N)C(OC)=O)C(OC)=O	0.51395273	0.49620727	0.53745496
O=C1N[C@@H](CCC(=O)N[C@@H](CCCn2nncc2CCCC(=O)NC[C@@H]1N)C(OC)=O)C(OC)=O	0.4197062	0.73046649	0.48865402
O=C1N[C@@H](CCC(=O)N[C@@H](CCCn2nncc2CCC(=O)NC[C@@H]1N)C(OC)=O)C(OC)=O	1.1532365	0.23998451	1.5045735
O=C1N[C@@H](CCC(=O)N[C@@H](CCCn2nncc2CNC(=O)NCC[C@@H]1N)C(OC)=O)C(OC)=O	1.1070197	0.35698327	1.4791632
O=C1N[C@@H](CCC(=O)N[C@@H](CCCn2nnc(CNC(=O)NC[C@@H]1N)c2)C(OC)=O)C(OC)=O	0.68571883	0.10869613	1.5765334
O=C1N[C@@H](CCC(=O)N[C@@H](CCCn2nncc2CNC(=O)NC[C@@H]1N)C(OC)=O)C(OC)=O	1.0726154	0.45212886	1.4788277

Principal moment of inertia calculations

We compared the molecular shape diversity of our DOS library with the same reference sets of 124 compounds used in the PCA.

The LowModeMD conformational search algorithm in the MOE software package[11] was used to generate low-energy 3D conformers for each compound. The MMFF94x force field was used with the generalized Born solvation model for the minimizations. Sampling and minimization parameters were implemented as follows:

Rejection Limit: 150

Iteration Limit: 10000

MM Iteration Limit: 500

RMS Gradient: 0.005

RMSD Limit: 0.15

Energy Window: 0.01

Refinement Conformation Limit: 300

Only the conformer with the lowest energy was retained for principal moment of inertia (PMI) calculations. Normalized PMI ratios (I_1/I_3 and I_2/I_3) of these conformers were obtained from MOE and then plotted on a triangular graph, with the coordinates (0,1), (0.5,0.5) and (1,1) representing a perfect rod, disc and sphere respectively (Figure Xd).

PMI data:

DRUGS:

Compound	npr1	npr2
rod	0.000000	0.500000
sphere	1.000000	1.000000
Discodermolide	0.500000	0.500000
Abilify	0.295654	0.778329
Aciphex	0.075929	0.980037
Aciphex	0.082531	0.982998
Actos	0.463255	0.696069
Adderall	0.187001	0.927327
Ambien	0.377477	0.680460
Avandia	0.375426	0.822949
Benazepril	0.445433	0.816395
Celebrex	0.363167	0.687574
Concerta	0.429713	0.689608
Coreg	0.656530	0.721788
Crestor	0.270494	0.811394
Cymbalta	0.396282	0.751873
Diovan	0.275662	0.849159
Effexor	0.353232	0.802320
Flonase	0.253542	0.963402
Fosamax	0.677628	0.780477
Imitrex	0.247709	0.819678
Lamictal	0.229390	0.911644
Levaquin	0.190810	0.844335
Lexapro	0.455280	0.723125
Lipitor	0.529854	0.809632
Nexium	0.275484	0.797230
Norvasc	0.427443	0.811931
Plavix	0.296689	0.880172
Prevacid	0.099878	0.966122
Prevacid	0.303180	0.886000
Protonix	0.089751	0.955345
Protonix	0.094608	0.959908
Risperdal	0.090427	0.936130
Serevent	0.509771	0.760083
Seroquel	0.165779	0.929957
Singulair	0.272087	0.916115
Topamax	0.351737	0.800199
Toprol	0.136820	0.913152
Tricor	0.207635	0.830835
Valtrex	0.287954	0.751349
Wellbutrin	0.195106	0.948008
Zetia	0.246216	0.805541

Zocor	0.403071	0.711209
Zoloft	0.299490	0.953590
Zyprexa	0.348574	0.692557
Zyrtec	0.199646	0.836693
**Drug-AVG**	**0.300776**	**0.834714**

NATURAL PRODUCTS:

Compound	npr1	npr2
Actinonin	0.314978	0.841802
Adriamycin	0.283220	0.800172
AmphotericinB	0.215551	0.847453
Apoptolidin	0.301694	0.830875
Bleomycin	0.404781	0.826205
BrefeldinA	0.255142	0.817388
BrevetoxinB	0.149647	0.899486
CalyculinA	0.442236	0.896401
Colchicine	0.395296	0.837965
Colchicine	0.258193	0.846521
Colchicine	0.458247	0.773003
Colchicine	0.513293	0.755147
CytochalasinB	0.431674	0.740608
Discodermolide	0.182128	0.981632
DuocarmycinA	0.104513	0.945885
EpothiloneA	0.447710	0.804309
ErythromycinA	0.485996	0.813810
Fumagillin	0.066479	0.974218
Geldanamycin	0.369201	0.725345
Geldanamycin	0.392818	0.769442
GinkgolideB	0.363537	0.879045
Lactacystin	0.478299	0.938709
Monensin	0.221267	0.914230
MycobactinS	0.567410	0.787169
PenicillinG	0.227679	0.840740
PhorbolMA	0.403734	0.770927
PhorbolMA	0.512005	0.787906
Radicicol	0.490589	0.823386
RifamycinB	0.534830	0.679088
RifamycinB	0.544966	0.674059
RifamycinB	0.618686	0.762072
RifamycinB	0.524795	0.868426
RifamycinB	0.648821	0.813721
RifamycinB	0.678080	0.844921
RifamycinB	0.627544	0.817624
RifamycinB	0.601820	0.886052
SalicylihalamideA	0.191225	0.848920
Staurosporine	0.464212	0.664055

Streptomycin	0.335744	0.786998
TalaromycinB	0.186426	0.935125
Telomestatin	0.496427	0.509642
TrapoxinB	0.460215	0.680871
Trichostatin	0.288807	0.802339
Vancomycin	0.516891	0.626096
Vincristine	0.482176	0.946784
Quinine	0.303785	0.834042
Spongistatin1	0.428667	0.817984
ZaragozicAcidA	0.287707	0.920770
Arglabin	0.402854	0.721308
Artemisinin	0.541282	0.644874
Bestatin	0.297570	0.838998
CephamycinC	0.488698	0.856650
Coformycin	0.298204	0.821609
Compactin	0.430983	0.716564
Forskolin	0.520873	0.688349
Mizoribine	0.239424	0.857007
Plaunotol	0.460051	0.900390
Spergualin	0.541169	0.946050
SQ26180	0.435835	0.794101
Thienamycin	0.476680	0.854387
AvermectinB1a	0.278083	0.812448
Calicheamicin	0.227585	0.920330
CyclosporinA	0.418415	0.935663
Daptomycin	0.762741	0.908347
EchinocandinB	0.317238	0.945521
FK506	0.377179	0.761235
Lipstatin	0.462998	0.738670
MidecamycinA1	0.325184	0.904263
PseudomonicAcidA	0.380836	0.748719
Rapamycin	0.445581	0.764618
Taxol	0.437027	0.832107
Validamycin	0.451444	0.807012
**Nat. Prod.-AVG**	**0.402459**	**0.818175**

MACROCYCLIC NATURAL PRODUCTS:

**Compound**	**npr1**	**npr2**
decarestrictineA1	0.469217	0.640137
decarestrictineB	0.610153	0.676987
decarestrictineC1	0.523140	0.616675
decarestrictineD	0.460594	0.707181
diplodialideA	0.395167	0.735937
jasmineketolactone	0.361150	0.799690
phoracantholideI	0.533624	0.723704
phoracantholideJ	0.463326	0.712112
pinolidoxin	0.297314	0.861372
pyrenolideA	0.343546	0.781822
ferrulactone1	0.560992	0.709680
'2,4,6,8-tetramethyl-3,4-dihydroxydec-8(9)-enolide'	0.577023	0.641499
apicularenA	0.354153	0.885766
cladospolideA	0.320632	0.796694
cladospolideB	0.438580	0.721334
cladospolideD	0.377547	0.753806
curvularin	0.303779	0.797272
lyngbouilloside	0.249436	0.959705
methymycin	0.436257	0.864653
neomethymycin	0.496348	0.948657
pladienolideB	0.134666	0.972028
fluvirucinA1	0.243234	0.862219
hypothemycin	0.329504	0.776135
iriomoteolide3a	0.277092	0.789016

DOS COMPOUND LIBRARY:

Compound	mseq	E	npr1	npr2
O=C1NCCCC[C@H](NC(=O)[C@@H](N)CCCCNC(=O)CCCc2nnn(c2)[C@H]1Cc1ccccc1)C(OC)=O	1	-18.579687	0.49725053	0.74374086
O=C1NCCCC[C@H](NC(=O)[C@@H](N)CCCCNC(=O)CCCc2n(nnc2)[C@H]1Cc1ccccc1)C(OC)=O	2	-14.322118	0.60735232	0.73507661
O=C1NCCCC[C@@H]2NC(=O)[C@@H](NC2=O)CCCCNC(=O)CCCc2nnn(c2)[C@H]1Cc1ccccc1	3	-3.2473774	0.36002895	0.72888464
O=C1NCCCC[C@@H]2NC(=O)[C@@H](NC2=O)CCCCNC(=O)CCCc2n(nnc2)[C@H]1Cc1ccccc1	4	-0.17650503	0.41468045	0.74923283
O=C1NCCCC[C@H](NC(=O)[C@@H](N)CCCNC(=O)CCc2nnn(c2)[C@H]1Cc1ccccc1)C(OC)=O	5	-19.520784	0.31402269	0.83290458
O=C1NCCCC[C@H](NC(=O)[C@@H](N)CCCNC(=O)CCc2n(nnc2)[C@H]1Cc1ccccc1)C(OC)=O	6	-9.6214275	0.37753254	0.72670829
O=C1NCCCC[C@@H]2NC(=O)[C@@H](NC2=O)CCCNC(=O)CCc2nnn(c2)[C@H]1Cc1ccccc1	7	-9.8396349	0.63542295	0.85442364
O=C1NCCCC[C@@H]2NC(=O)[C@@H](NC2=O)CCCNC(=O)CCc2n(nnc2)[C@H]1Cc1ccccc1	8	-1.8342946	0.51106262	0.76519442
O=C1NCCCC[C@H](NC(=O)[C@@H](N)CCCNC(=O)CCCc2nnn(c2)[C@H]1Cc1ccccc1)C(OC)=O	9	-18.378744	0.34596333	0.74453539
O=C1NCCCC[C@H](NC(=O)[C@@H](N)CCCNC(=O)CCCc2n(nnc2)[C@H]1Cc1ccccc1)C(OC)=O	10	-11.323607	0.50315166	0.87528473
O=C1NCCCC[C@@H]2NC(=O)[C@@H](NC2=O)CCCNC(=O)CCCc2nnn(c2)[C@H]1Cc1ccccc1	11	-4.5649004	0.5886426	0.7012735
O=C1NCCCC[C@@H]2NC(=O)[C@@H](NC2=O)CCCNC(=O)CCCc2n(nnc2)[C@H]1Cc1ccccc1	12	3.5529692	0.46205592	0.77413642
O=C1NCCCC[C@H](NC(=O)[C@@H](N)CCCCNC(=O)NCc2nnn(c2)[C@H]1Cc1ccccc1)C(OC)=O	13	-88.543777	0.44779876	0.69951922
O=C1NCCCC[C@H](NC(=O)[C@@H](N)CCCCNC(=O)NCc2n(nnc2)[C@H]1Cc1ccccc1)C(OC)=O	14	-108.15442	0.38272107	0.86493093
O=C1NCCCC[C@@H]2NC(=O)[C@@H](NC2=O)CCCCNC(=O)NCc2n(nnc2)[C@H]1Cc1ccccc1	15	-96.003441	0.33027884	0.84070128
O=C1NCCCC[C@H](NC(=O)[C@@H](N)CCCNC(=O)NCc2nnn(c2)[C@H]1Cc1ccccc1)C(OC)=O	16	-96.254021	0.55230206	0.85025734
O=C1NCCCC[C@H](NC(=O)[C@@H](N)CCCNC(=O)NCc2n(nnc2)[C@H]1Cc1ccccc1)C(OC)=O	17	-114.26881	0.29692572	0.841272
O=C1NCCCC[C@@H]2NC(=O)[C@@H](NC2=O)CCCNC(=O)NCc2n(nnc2)[C@H]1Cc1ccccc1	18	-94.35981	0.41035461	0.84274638
O=C1NCCCC[C@H](NC(=O)[C@@H](N)Cc2ccc(NC(=O)CCc3n(nnc3)[C@H]1Cc1ccccc1)cc2)C(OC)=O	19	21.7721	0.37631527	0.85723019

O=C1NCCCC[C@@H]2NC(=O)[C@@H](NC2=O)Cc2ccc(NC(=O)CCc3n(nnc3)[C@H]1Cc1ccccc1)cc2	20	32.2103	0.4299	0.9097
O=C1NCCCC[C@H](NC(=O)[C@@H](N)CCNC(=O)CCCc2nnn(c2)[C@H]1Cc1ccccc1)C(OC)=O	21	-20.424511	0.62381011	0.7540288
O=C1NCCCC[C@H](NC(=O)[C@@H](N)CCNC(=O)CCCc2n(nnc2)[C@H]1Cc1ccccc1)C(OC)=O	22	-22.050838	0.37934071	0.83368534
O=C1NCCCC[C@@H]2NC(=O)[C@@H](NC2=O)CCNC(=O)CCCc2nnn(c2)[C@H]1Cc1ccccc1	23	-7.3923335	0.2720947	0.85539728
O=C1NCCCC[C@@H]2NC(=O)[C@@H](NC2=O)CCNC(=O)CCCc2n(nnc2)[C@H]1Cc1ccccc1	24	-6.8267436	0.39542383	0.96092379
O=C1NCCCC[C@H](NC(=O)[C@@H](N)CCNC(=O)CCc2nnn(c2)[C@H]1Cc1ccccc1)C(OC)=O	25	-22.361309	0.32048732	0.8540405
O=C1NCCCC[C@H](NC(=O)[C@@H](N)CCNC(=O)CCc2n(nnc2)[C@H]1Cc1ccccc1)C(OC)=O	26	-16.879242	0.39819816	0.94482762
O=C1NCCCC[C@@H]2NC(=O)[C@@H](NC2=O)CCNC(=O)CCc2n(nnc2)[C@H]1Cc1ccccc1	27	-2.6672537	0.54260963	0.87756228
O=C1NCCCC[C@H](NC(=O)[C@@H](N)CNC(=O)CCCc2nnn(c2)[C@H]1Cc1ccccc1)C(OC)=O	28	0.34442022	0.41999263	0.78562939
O=C1NCCCC[C@H](NC(=O)[C@@H](N)CNC(=O)CCCc2n(nnc2)[C@H]1Cc1ccccc1)C(OC)=O	29	6.9792137	0.36260137	0.82622057
O=C1NCCCC[C@@H]2NC(=O)[C@@H](NC2=O)CNC(=O)CCCc2nnn(c2)[C@H]1Cc1ccccc1	30	9.1514597	0.39401668	0.75762069
O=C1NCCCC[C@@H]2NC(=O)[C@@H](NC2=O)CNC(=O)CCCc2n(nnc2)[C@H]1Cc1ccccc1	31	14.207901	0.51528609	0.79837269
O=C1NCCCC[C@H](NC(=O)[C@@H](N)CNC(=O)CCc2nnn(c2)[C@H]1Cc1ccccc1)C(OC)=O	32	-4.9668097	0.45067513	0.68744051
O=C1NCCCC[C@H](NC(=O)[C@@H](N)CNC(=O)CCc2n(nnc2)[C@H]1Cc1ccccc1)C(OC)=O	33	4.542604	0.42529106	0.89633834
O=C1NCCCC[C@@H]2NC(=O)[C@@H](NC2=O)CNC(=O)CCc2n(nnc2)[C@H]1Cc1ccccc1	34	11.954076	0.30352968	0.89746457
O=C1NCCCC[C@H](NC(=O)[C@@H](N)CCNC(=O)NCc2nnn(c2)[C@H]1Cc1ccccc1)C(OC)=O	35	-91.403183	0.4733561	0.6595912
O=C1NCCCC[C@H](NC(=O)[C@@H](N)CCNC(=O)NCc2n(nnc2)[C@H]1Cc1ccccc1)C(OC)=O	36	-117.00694	0.29085389	0.92186409
O=C1NCCCC[C@@H]2NC(=O)[C@@H](NC2=O)CCNC(=O)NCc2n(nnc2)[C@H]1Cc1ccccc1	37	-101.00074	0.35293689	0.93048453
O=C1NCCCC[C@H](NC(=O)[C@@H](N)CNC(=O)NCc2nnn(c2)[C@H]1Cc1ccccc1)C(OC)=O	38	-72.761948	0.32672498	0.91003609
O=C1NCCCC[C@H](NC(=O)[C@@H](N)CNC(=O)NCc2n(nnc2)[C@H]1Cc1ccccc1)C(OC)=O	39	-94.11911	0.29713649	0.88092822
O=C1NCCCC[C@@H]2NC(=O)[C@@H](NC2=O)CNC(=O)NCc2nnn(c2)[C@H]1Cc1ccccc1	40	-64.506279	0.27160886	0.84741575
O=C1NCCCC[C@@H]2NC(=O)[C@@H](NC2=O)CNC(=O)NCc2n(nnc2)[C@H]1Cc1ccccc1	41	-84.860443	0.29323372	0.93158513
O=C1NCCCC[C@H](NC(=O)[C@@H](N)Cc2ccc(NC(=O)CCCc3nnn(c3)[C@H]1Cc1ccccc1)cc2)C(OC)=O	42	16.861866	0.42829001	0.97634572

O=C1NCCCC[C@H](NC(=O)[C@@H](N)Cc2ccc(NC(=O)CCCc3n(nnc3)[C@H]1Cc1ccccc1)cc2)C(OC)=O	43	20.141994	0.36281759	0.88496989
O=C1NCCCC[C@@H]2NC(=O)[C@@H](NC2=O)Cc2ccc(NC(=O)CCCc3n(nnc3)[C@H]1Cc1ccccc1)cc2	44	30.216309	0.48582417	0.84051621
O=C1NC[C@H](NC(=O)[C@@H](N)CCCCNC(=O)CCCc2nnn(c2)[C@H]1Cc1ccccc1)C(OC)=O	45	-5.4302721	0.4966419	0.64285988
O=C1NC[C@H](NC(=O)[C@@H](N)CCCCNC(=O)CCCc2n(nnc2)[C@H]1Cc1ccccc1)C(OC)=O	46	-3.4149508	0.5272125	0.79831368
O=C1NC[C@@H]2NC(=O)[C@@H](NC2=O)CCCCNC(=O)CCCc2nnn(c2)[C@H]1Cc1ccccc1	47	9.2347794	0.34794316	0.79531246
O=C1NC[C@@H]2NC(=O)[C@@H](NC2=O)CCCCNC(=O)CCCc2n(nnc2)[C@H]1Cc1ccccc1	48	12.712286	0.57459515	0.84453958
O=C1NC[C@H](NC(=O)[C@@H](N)CCCNC(=O)CCc2nnn(c2)[C@H]1Cc1ccccc1)C(OC)=O	49	-17.769306	0.5007661	0.66096705
O=C1NC[C@H](NC(=O)[C@@H](N)CCCNC(=O)CCc2n(nnc2)[C@H]1Cc1ccccc1)C(OC)=O	50	-6.6144142	0.42122838	0.83994621
O=C1NC[C@@H]2NC(=O)[C@@H](NC2=O)CCCNC(=O)CCc2nnn(c2)[C@H]1Cc1ccccc1	51	4.2219515	0.36738986	0.78082758
O=C1NC[C@@H]2NC(=O)[C@@H](NC2=O)CCCNC(=O)CCc2n(nnc2)[C@H]1Cc1ccccc1	52	14.881308	0.37162387	0.76978368
O=C1NC[C@H](NC(=O)[C@@H](N)CCCNC(=O)CCCc2nnn(c2)[C@H]1Cc1ccccc1)C(OC)=O	53	-12.096288	0.56741917	0.65458477
O=C1NC[C@H](NC(=O)[C@@H](N)CCCNC(=O)CCCc2n(nnc2)[C@H]1Cc1ccccc1)C(OC)=O	54	-11.137595	0.54705954	0.85922724
O=C1NC[C@@H]2NC(=O)[C@@H](NC2=O)CCCNC(=O)CCCc2nnn(c2)[C@H]1Cc1ccccc1	55	6.9486489	0.31544626	0.79178888
O=C1NC[C@@H]2NC(=O)[C@@H](NC2=O)CCCNC(=O)CCCc2n(nnc2)[C@H]1Cc1ccccc1	56	12.123857	0.56426167	0.64529794
O=C1NC[C@H](NC(=O)[C@@H](N)CCCCNC(=O)NCc2nnn(c2)[C@H]1Cc1ccccc1)C(OC)=O	57	-82.05674	0.54875678	0.64977336
O=C1NC[C@H](NC(=O)[C@@H](N)CCCCNC(=O)NCc2n(nnc2)[C@H]1Cc1ccccc1)C(OC)=O	58	-102.69968	0.42496893	0.80071694
O=C1NC[C@@H]2NC(=O)[C@@H](NC2=O)CCCCNC(=O)NCc2nnn(c2)[C@H]1Cc1ccccc1	59	-63.343781	0.37705129	0.751553
O=C1NC[C@@H]2NC(=O)[C@@H](NC2=O)CCCCNC(=O)NCc2n(nnc2)[C@H]1Cc1ccccc1	60	-82.777809	0.58810478	0.71517092
O=C1NC[C@H](NC(=O)[C@@H](N)CCCNC(=O)NCc2nnn(c2)[C@H]1Cc1ccccc1)C(OC)=O	61	-80.352592	0.39169192	0.87295389
O=C1NC[C@H](NC(=O)[C@@H](N)CCCNC(=O)NCc2n(nnc2)[C@H]1Cc1ccccc1)C(OC)=O	62	-104.75678	0.47762802	0.94119376
O=C1NC[C@@H]2NC(=O)[C@@H](NC2=O)CCCNC(=O)NCc2nnn(c2)[C@H]1Cc1ccccc1	63	-63.992599	0.31471997	0.83503079
O=C1NC[C@H](NC(=O)[C@@H](N)CCNC(=O)CCCc2nnn(c2)[C@H]1Cc1ccccc1)C(OC)=O	64	-9.718503	0.47863334	0.64831448
O=C1NC[C@H](NC(=O)[C@@H](N)CCNC(=O)CCCc2n(nnc2)[C@H]1Cc1ccccc1)C(OC)=O	65	-8.3961773	0.60411292	0.67564666
O=C1NC[C@@H]2NC(=O)[C@@H](NC2=O)CCNC(=O)CCCc2n(nnc2)[C@H]1Cc1ccccc1	66	11.761711	0.35096869	0.84563184
O=C1NC[C@H](NC(=O)[C@@H](N)CCNC(=O)CCc2nnn(c2)[C@H]1Cc1ccccc1)C(OC)=O	67	-18.718	0.511	0.5932
O=C1NC[C@H](NC(=O)[C@@H](N)CCNC(=O)CCc2n(nnc2)[C@H]1Cc1ccccc1)C(OC)=O	68	-8.4456005	0.4308047	0.86673069
O=C1NC[C@@H]2NC(=O)[C@@H](NC2=O)CCNC(=O)CCc2nnn(c2)[C@H]1Cc1ccccc1	69	6.9916992	0.30446869	0.82000983
O=C1NC[C@@H]2NC(=O)[C@@H](NC2=O)CCNC(=O)CCc2n(nnc2)[C@H]1Cc1ccccc1	70	17.265985	0.40353775	0.7802431
O=C1NC[C@H](NC(=O)[C@@H](N)CNC(=O)CCCc2nnn(c2)[C@H]1Cc1ccccc1)C(OC)=O	71	11.222864	0.43740579	0.68413889

O=C1NC[C@H](NC(=O)[C@@H](N)CNC(=O)CCCc2n(nnc2)[C@H]1Cc1ccccc1)C(OC)=O	72	10.067511	0.41724664	0.87867606
O=C1NC[C@H](NC(=O)[C@@H](N)CNC(=O)CCc2nnn(c2)[C@H]1Cc1ccccc1)C(OC)=O	73	2.5442712	0.35860908	0.75528985
O=C1NC[C@H](NC(=O)[C@@H](N)CNC(=O)CCc2n(nnc2)[C@H]1Cc1ccccc1)C(OC)=O	74	17.209806	0.61315691	0.73103625
O=C1NC[C@@H]2NC(=O)[C@@H](NC2=O)CNC(=O)CCc2nnn(c2)[C@H]1Cc1ccccc1	75	17.272606	0.34447932	0.83280057
O=C1NC[C@@H]2NC(=O)[C@@H](NC2=O)CNC(=O)CCc2n(nnc2)[C@H]1Cc1ccccc1	76	53.9048	0.6040653	0.857418
O=C1NC[C@H](NC(=O)[C@@H](N)CCNC(=O)NCc2nnn(c2)[C@H]1Cc1ccccc1)C(OC)=O	77	-83.284538	0.4169791	0.66488814
O=C1NC[C@H](NC(=O)[C@@H](N)CCNC(=O)NCc2n(nnc2)[C@H]1Cc1ccccc1)C(OC)=O	78	-101.91926	0.33758074	0.90776217
O=C1NC[C@H](NC(=O)[C@@H](N)CNC(=O)NCc2nnn(c2)[C@H]1Cc1ccccc1)C(OC)=O	79	-63.18932	0.45645005	0.63577461
O=C1NC[C@H](NC(=O)[C@@H](N)CNC(=O)NCc2n(nnc2)[C@H]1Cc1ccccc1)C(OC)=O	80	-87.65741	0.370906	0.88365996
O=C1NC[C@@H]2NC(=O)[C@@H](NC2=O)CNC(=O)NCc2n(nnc2)[C@H]1Cc1ccccc1	81	-41.251129	0.58475274	0.85370725
O=C1NC[C@H](NC(=O)[C@@H](N)Cc2ccc(NC(=O)CCCc3n(nnc3)[C@H]1Cc1ccccc1)cc2)<br>C(OC)=O	82	29.180834	0.43904787	0.82960278
O=C1NC[C@H](NC(=O)[C@@H](N)CCCCNC(=O)CCc2nnn(c2)[C@H]1Cc1ccccc1)C(OC)=<br>O	83	-13.468704	0.44628623	0.68228304
O=C1NC[C@H](NC(=O)[C@@H](N)CCCCNC(=O)CCc2n(nnc2)[C@H]1Cc1ccccc1)C(OC)=<br>O	84	-6.4742942	0.45334679	0.8344121
O=C1NC[C@@H]2NC(=O)[C@@H](NC2=O)CCCCNC(=O)CCc2nnn(c2)[C@H]1Cc1ccccc1	85	11.754751	0.35068744	0.73410743
O=C1NC[C@@H]2NC(=O)[C@@H](NC2=O)CCCCNC(=O)CCc2n(nnc2)[C@H]1Cc1ccccc1	86	23.866859	0.38330805	0.86330497
O=C1N[C@@H](CCC(=O)N[C@@H](CCCCn2nncc2CCCC(=O)NCCCC[C@@H]1NC(OC(C<br>)(C)C)=O)C(OC)=O)C(OC)=O	87	-84.348274	0.56439245	0.80527276
O=C1N[C@@H](CCC(=O)N[C@@H](CCCCn2nnc(CCC(=O)NCCC[C@@H]1NC(OC(C)(C)<br>C)=O)c2)C(OC)=O)C(OC)=O	88	-96.678932	0.62275279	0.84016353
O=C1N[C@@H](CCC(=O)N[C@@H](CCCCn2nncc2CCC(=O)NCCC[C@@H]1NC(OC(C)(C<br>)C)=O)C(OC)=O)C(OC)=O	89	-91.111252	0.50404018	0.80163246
O=C1N[C@@H](CCC(=O)N[C@@H](CCCCn2nnc(CCCC(=O)NCCC[C@@H]1NC(OC(C)(C<br>)C)=O)c2)C(OC)=O)C(OC)=O	90	-95.488235	0.63432962	0.74538469
O=C1N[C@@H](CCC(=O)N[C@@H](CCCCn2nncc2CCCC(=O)NCCC[C@@H]1NC(OC(C)(<br>C)C)=O)C(OC)=O)C(OC)=O	91	-92.237541	0.63055748	0.73572159
O=C1N[C@@H](CCC(=O)N[C@@H](CCCCn2nnc(CNC(=O)NCCCC[C@@H]1NC(OC(C)(C<br>)C)=O)c2)C(OC)=O)C(OC)=O	92	-163.11015	0.60197908	0.9719469
O=C1N[C@@H](CCC(=O)N[C@@H](CCCCn2nncc2CNC(=O)NCCCC[C@@H]1NC(OC(C)(<br>C)C)=O)C(OC)=O)C(OC)=O	93	-185.10393	0.56670898	0.93692285
O=C1N[C@@H](CCC(=O)N[C@@H](CCCCn2nnc(CNC(=O)NCCC[C@@H]1NC(OC(C)(C)<br>C)=O)c2)C(OC)=O)C(OC)=O	94	-166.7549	0.49497095	0.80682433
O=C1N[C@@H](CCC(=O)N[C@@H](CCCCn2nncc2CNC(=O)NCCC[C@@H]1NC(OC(C)(C<br>)C)=O)C(OC)=O)C(OC)=O	95	-184.94441	0.50868237	0.88095331
O=C1N[C@@H](CCC(=O)N[C@@H](CCCCn2nncc2CCC(=O)Nc2ccc(C[C@@H]1NC(OC(C<br>)(C)C)=O)cc2)C(OC)=O)C(OC)=O	96	-57.991936	0.37540352	0.86856055
O=C1N[C@@H](CCC(=O)N[C@@H](CCCCn2nncc2CCCC(=O)NCC[C@@H]1NC(OC(C)(C<br>)C)=O)C(OC)=O)C(OC)=O	97	-95.922333	0.49921075	0.72851467

O=C1N[C@@H](CCC(=O)N[C@@H](CCCCn2nncc2CCC(=O)NCC[C@@H]1NC(OC(C)(C)C)=O)C(OC)=O)C(OC)=O	98	-91.449821	0.55036283	0.9053961
O=C1N[C@@H](CCC(=O)N[C@@H](CCCCn2nnc(CCCC(=O)NC[C@@H]1NC(OC(C)(C)C)=O)c2)C(OC)=O)C(OC)=O	99	-79.807877	0.63354748	0.72110665
O=C1N[C@@H](CCC(=O)N[C@@H](CCCCn2nncc2CCCC(=O)NC[C@@H]1NC(OC(C)(C)C)=O)C(OC)=O)C(OC)=O	100	-78.364769	0.52900177	0.83459127
O=C1N[C@@H](CCC(=O)N[C@@H](CCCCn2nnc(CCC(=O)NC[C@@H]1NC(OC(C)(C)C)=O)c2)C(OC)=O)C(OC)=O	101	-84.878838	0.64835227	0.82031065
O=C1N[C@@H](CCC(=O)N[C@@H](CCCCn2nncc2CCC(=O)NC[C@@H]1NC(OC(C)(C)C)=O)C(OC)=O)C(OC)=O	102	-75.939598	0.6383611	0.79234838
O=C1N[C@@H](CCC(=O)N[C@@H](CCCCn2nnc(CNC(=O)NCC[C@@H]1NC(OC(C)(C)C)=O)c2)C(OC)=O)C(OC)=O	103	-167.03944	0.50624061	0.79291606
O=C1N[C@@H](CCC(=O)N[C@@H](CCCCn2nncc2CNC(=O)NCC[C@@H]1NC(OC(C)(C)C)=O)C(OC)=O)C(OC)=O	104	-187.67111	0.73933649	0.84168613
O=C1N[C@@H](CCC(=O)N[C@@H](CCCCn2nnc(CNC(=O)NC[C@@H]1NC(OC(C)(C)C)=O)c2)C(OC)=O)C(OC)=O	105	-154.54196	0.51802182	0.82181644
O=C1N[C@@H](CCC(=O)N[C@@H](CCCCn2nncc2CNC(=O)NC[C@@H]1NC(OC(C)(C)C)=O)C(OC)=O)C(OC)=O	106	-178.90086	0.5308134	0.83153403
O=C1N[C@@H](CCC(=O)N[C@@H](CCCCn2nnc(CCCC(=O)Nc3ccc(C[C@@H]1NC(OC(C)(C)C)=O)cc3)c2)C(OC)=O)C(OC)=O	107	-59.771255	0.43111831	0.8238073
O=C1N[C@@H](CCC(=O)N[C@@H](CCCCn2nncc2CCCC(=O)Nc2ccc(C[C@@H]1NC(OC(C)(C)C)=O)cc2)C(OC)=O)C(OC)=O	108	-58.245007	0.46353364	0.90409613
O=C1N[C@@H](CCC(=O)N[C@@H](CCCCn2nnc(CCC(=O)NCCCC[C@@H]1NC(OC(C)(C)C)=O)c2)C(OC)=O)C(OC)=O	109	-93.155052	0.60777521	0.67905009
O=C1N[C@@H](CCC(=O)N[C@@H](CCCCn2nncc2CCC(=O)NCCCC[C@@H]1NC(OC(C)(C)C)=O)C(OC)=O)C(OC)=O	110	-86.323273	0.63884324	0.78780383
O=C1N[C@@H](Cc2ccc(-n3nncc3CCC(=O)NCCC[C@@H]1N)cc2)C(OC)=O	111	35.899456	0.3728883	0.72347969
O=C1N[C@@H](Cc2ccc(-n3nncc3CCCC(=O)NCCC[C@@H]1N)cc2)C(OC)=O	112	33.645023	0.48053569	0.66790539
O=C1N[C@@H](Cc2ccc(-n3nncc3CNC(=O)NCCC[C@@H]1N)cc2)C(OC)=O	113	-66.020218	0.4614481	0.70884454
O=C1N[C@@H](Cc2ccc(-n3nncc3CCCC(=O)NCC[C@@H]1N)cc2)C(OC)=O	114	31.553934	0.29877383	0.79601592
O=C1N[C@@H](Cc2ccc(-n3nncc3CCC(=O)NCC[C@@H]1N)cc2)C(OC)=O	115	38.016705	0.31888345	0.7459178
O=C1N[C@@H](Cc2ccc(-n3nncc3CCC(=O)NC[C@@H]1N)cc2)C(OC)=O	116	57.691101	0.43886793	0.75752836
O=C1N[C@@H](Cc2ccc(-n3nnc(CNC(=O)NCC[C@@H]1N)c3)cc2)C(OC)=O	117	-36.910328	0.39949039	0.84595937
O=C1N[C@@H](Cc2ccc(-n3nncc3CNC(=O)NC[C@@H]1N)cc2)C(OC)=O	118	-43.840664	0.294438	0.83104438
O=C1N[C@@H](Cc2ccc(-n3nncc3CCC(=O)NCCCC[C@@H]1N)cc2)C(OC)=O	119	37.083546	0.38379994	0.68476152
O=C1N[C@@H](CCCCn2nnc(CCCC(=O)NCCCC[C@@H]1N)c2)C(OC)=O	120	-13.00311	0.43042102	0.70528179
O=C1N[C@@H](CCCCn2nncc2CCCC(=O)NCCCC[C@@H]1N)C(OC)=O	121	-12.119275	0.38151413	0.77146924
O=C1NCCCC[C@@H]2NC(=O)[C@@H](NC2=O)CCCCn2nncc2CCC1	122	6.0212989	0.39186087	0.72891116
O=C1N[C@@H](CCCCn2nnc(CCC(=O)NCCC[C@@H]1N)c2)C(OC)=O	123	-22.655432	0.40475857	0.76092941
O=C1N[C@@H](CCCCn2nncc2CCC(=O)NCCC[C@@H]1N)C(OC)=O	124	-13.135283	0.49564305	0.75771385

O=C1NCCC[C@@H]2NC(=O)[C@@H](NC2=O)CCCCn2nnc(CC1)c2	125	-4.9133601	0.5447709	0.6966098
O=C1N[C@@H](CCCCn2nnc(CCCC(=O)NCCC[C@@H]1N)c2)C(OC)=O	126	-17.423956	0.39693525	0.71853685
O=C1N[C@@H](CCCCn2nncc2CCCC(=O)NCCC[C@@H]1N)C(OC)=O	127	-15.194193	0.41603774	0.68488586
O=C1NCCC[C@@H]2NC(=O)[C@@H](NC2=O)CCCCn2nnc(CCC1)c2	128	-0.65803885	0.48575568	0.70409054
O=C1N[C@@H](CCCCn2nncc2CNC(=O)NCCCC[C@@H]1N)C(OC)=O	129	-108.63251	0.36954659	0.75336808
O=C1N[C@@H](CCCCn2nnc(CNC(=O)NCCC[C@@H]1N)c2)C(OC)=O	130	-89.262825	0.49788076	0.70646256
O=C1N[C@@H](CCCCn2nncc2CNC(=O)NCCC[C@@H]1N)C(OC)=O	131	-111.70306	0.51858413	0.72709286
O=C1N[C@@H](CCCCn2nncc2CCC(=O)Nc2ccc(C[C@@H]1N)cc2)C(OC)=O	132	24.85358	0.40268311	0.80658579
O=C1N[C@@H](CCCCn2nnc(CCCC(=O)NCC[C@@H]1N)c2)C(OC)=O	133	-19.275101	0.37477592	0.78823406
O=C1N[C@@H](CCCCn2nncc2CCCC(=O)NCC[C@@H]1N)C(OC)=O	134	-14.44465	0.36576176	0.72217441
O=C1NCC[C@@H]2NC(=O)[C@@H](NC2=O)CCCCn2nncc2CCC1	135	0.99981314	0.45221341	0.70502406
O=C1N[C@@H](CCCCn2nnc(CCC(=O)NCC[C@@H]1N)c2)C(OC)=O	136	-25.38719	0.39723027	0.7645489
O=C1N[C@@H](CCCCn2nncc2CCC(=O)NCC[C@@H]1N)C(OC)=O	137	-14.983081	0.3524591	0.78281516
O=C1NCC[C@@H]2NC(=O)[C@@H](NC2=O)CCCCn2nnc(CC1)c2	138	-8.4020452	0.44712493	0.73909682
O=C1NCC[C@@H]2NC(=O)[C@@H](NC2=O)CCCCn2nncc2CC1	139	7.71175	0.46809992	0.7144075
O=C1N[C@@H](CCCCn2nncc2CCCC(=O)NC[C@@H]1N)C(OC)=O	140	10.466128	0.39396542	0.83777636
O=C1NC[C@@H]2NC(=O)[C@@H](NC2=O)CCCCn2nncc2CCC1	141	26.26774	0.50152749	0.64698189
O=C1N[C@@H](CCCCn2nnc(CCC(=O)NC[C@@H]1N)c2)C(OC)=O	142	-1.9879884	0.41344902	0.73575932
O=C1N[C@@H](CCCCn2nncc2CCC(=O)NC[C@@H]1N)C(OC)=O	143	7.7377172	0.41473764	0.78866386
O=C1N[C@@H](CCCCn2nnc(CNC(=O)NCC[C@@H]1N)c2)C(OC)=O	144	-88.842125	0.43568647	0.72683185
O=C1N[C@@H](CCCCn2nncc2CNC(=O)NCC[C@@H]1N)C(OC)=O	145	-111.04406	0.3980346	0.85565907
O=C1N[C@H]2CCNC(=O)NCc3nnn(CCCC[C@@H]1NC2=O)c3	146	-75.802109	0.4468348	0.73548961
O=C1N[C@@H](CCCCn2nnc(CNC(=O)NC[C@@H]1N)c2)C(OC)=O	147	-69.678452	0.46289912	0.74438435
O=C1N[C@@H](CCCCn2nncc2CNC(=O)NC[C@@H]1N)C(OC)=O	148	-92.720367	0.54889184	0.75719941
O=C1N[C@@H](CCCCn2nncc2CCCC(=O)Nc2ccc(C[C@@H]1N)cc2)C(OC)=O	149	22.697845	0.3920581	0.70692921
O=C1Nc2ccc(C[C@@H]3NC(=O)[C@@H](NC3=O)CCCCn3nncc3CCC1)cc2	150	35.952286	0.34108546	0.81180215
O=C1N[C@@H](CCCCn2nnc(CCC(=O)NCCCC[C@@H]1N)c2)C(OC)=O	151	-15.423905	0.41586453	0.71418226
O=C1N[C@@H](CCCCn2nncc2CCC(=O)NCCCC[C@@H]1N)C(OC)=O	152	-11.998137	0.4303503	0.68871444
O=C1NCCCC[C@@H]2NC(=O)[C@@H](NC2=O)CCCCn2nnc(CC1)c2	153	-4.4272232	0.47201607	0.71824747
O=C1NCCCC[C@@H]2NC(=O)[C@@H](NC2=O)CCCCn2nncc2CC1	154	6.726584	0.44043195	0.72649437
O=C1N[C@@H](CCCn2nnc(CCCC(=O)NCCCC[C@@H]1N)c2)C(OC)=O	155	-14.881832	0.42390156	0.72647011
O=C1N[C@@H](CCCn2nncc2CCCC(=O)NCCCC[C@@H]1N)C(OC)=O	156	-15.104601	0.50110692	0.66826588
O=C1NCCCC[C@@H]2NC(=O)[C@@H](NC2=O)CCCn2nnc(CCC1)c2	157	-0.17798263	0.44851017	0.71295089
O=C1NCCCC[C@@H]2NC(=O)[C@@H](NC2=O)CCCn2nncc2CCC1	158	5.6430311	0.44692904	0.66004395

O=C1N[C@@H](CCCn2nnc(CCC(=O)NCCC[C@@H]1N)c2)C(OC)=O	159	-24.540197	0.46330363	0.71350104
O=C1N[C@@H](CCCn2nncc2CCC(=O)NCCC[C@@H]1N)C(OC)=O	160	-14.044133	0.4548575	0.64496201
O=C1N[C@@H](CCCn2nnc(CCCC(=O)NCCC[C@@H]1N)c2)C(OC)=O	161	-18.484922	0.46638444	0.68450415
O=C1N[C@@H](CCCn2nncc2CCCC(=O)NCCC[C@@H]1N)C(OC)=O	162	-12.911449	0.54059654	0.77229595
O=C1NCCC[C@@H]2NC(=O)[C@@H](NC2=O)CCCn2nncc2CCC1	163	-0.21514663	0.50794965	0.70846164
O=C1N[C@@H](CCCn2nnc(CNC(=O)NCCCC[C@@H]1N)c2)C(OC)=O	164	-88.966888	0.47320172	0.69720769
O=C1N[C@@H](CCCn2nncc2CNC(=O)NCCCC[C@@H]1N)C(OC)=O	165	-109.55763	0.52422678	0.70912975
O=C1N[C@@H](CCCn2nnc(CNC(=O)NCCC[C@@H]1N)c2)C(OC)=O	166	-88.259743	0.48106688	0.6789254
O=C1N[C@@H](CCCn2nncc2CNC(=O)NCCC[C@@H]1N)C(OC)=O	167	-113.18249	0.48407644	0.68193197
O=C1N[C@@H](CCCn2nncc2CCC(=O)Nc2ccc(C[C@@H]1N)cc2)C(OC)=O	168	24.03076	0.47078517	0.75115645
O=C1Nc2ccc(C[C@@H]3NC(=O)[C@@H](NC3=O)CCCn3nncc3CC1)cc2	169	61.218105	0.53493398	0.66567224
O=C1N[C@@H](CCCn2nnc(CCCC(=O)NCC[C@@H]1N)c2)C(OC)=O	170	-20.410398	0.33545983	0.78977543
O=C1N[C@@H](CCCn2nncc2CCCC(=O)NCC[C@@H]1N)C(OC)=O	171	-17.971935	0.49327296	0.66700298
O=C1NCC[C@@H]2NC(=O)[C@@H](NC2=O)CCCn2nncc2CCC1	172	2.2045178	0.45068887	0.68783325
O=C1N[C@@H](CCCn2nnc(CCC(=O)NCC[C@@H]1N)c2)C(OC)=O	173	-25.343792	0.35621271	0.78327304
O=C1N[C@@H](CCCn2nncc2CCC(=O)NCC[C@@H]1N)C(OC)=O	174	-15.54284	0.49479821	0.63783991
O=C1NCC[C@@H]2NC(=O)[C@@H](NC2=O)CCCn2nncc2CC1	175	9.4042511	0.57301158	0.62474722
O=C1N[C@@H](CCCn2nnc(CCCC(=O)NC[C@@H]1N)c2)C(OC)=O	176	6.2944975	0.38503465	0.77480108
O=C1N[C@@H](CCCn2nncc2CCCC(=O)NC[C@@H]1N)C(OC)=O	177	2.46229	0.54378366	0.63764811
O=C1NC[C@@H]2NC(=O)[C@@H](NC2=O)CCCn2nncc2CCC1	178	31.585995	0.46742818	0.6840266
O=C1N[C@@H](CCCn2nnc(CCC(=O)NC[C@@H]1N)c2)C(OC)=O	179	-4.0336452	0.36694306	0.80332661
O=C1N[C@@H](CCCn2nncc2CCC(=O)NC[C@@H]1N)C(OC)=O	180	5.6035581	0.50513762	0.67898184
O=C1N[C@@H](CCCn2nnc(CNC(=O)NCC[C@@H]1N)c2)C(OC)=O	181	-91.270409	0.56873423	0.62553579
O=C1N[C@@H](CCCn2nncc2CNC(=O)NCC[C@@H]1N)C(OC)=O	182	-114.49229	0.48644918	0.70251274
O=C1N[C@H]2CCNC(=O)NCc3n(nnc3)CCC[C@@H]1NC2=O	183	-88.4244	0.55775338	0.73375612
O=C1N[C@@H](CCCn2nncc2CNC(=O)NC[C@@H]1N)C(OC)=O	184	-95.262352	0.50209337	0.7013309
O=C1N[C@@H](CCCn2nncc2CCCC(=O)Nc2ccc(C[C@@H]1N)cc2)C(OC)=O	185	23.774298	0.44917879	0.8953523
O=C1Nc2ccc(C[C@@H]3NC(=O)[C@@H](NC3=O)CCCn3nncc3CCC1)cc2	186	36.792198	0.39035001	0.7618801
O=C1N[C@@H](CCCn2nnc(CCC(=O)NCCCC[C@@H]1N)c2)C(OC)=O	187	-21.284054	0.40950742	0.70367771
O=C1N[C@@H](CCCn2nncc2CCC(=O)NCCCC[C@@H]1N)C(OC)=O	188	-12.654502	0.44834211	0.63135093
O=C1NCCCC[C@@H]2NC(=O)[C@@H](NC2=O)CCCn2nncc2CC1	189	13.640135	0.57658237	0.68448555
O=C1N[C@@H](Cc2cc(-n3nncc3CNC(=O)NCCCC[C@@H]1N)ccc2)C(OC)=O	190	-61.487297	0.55730349	0.65523624
O=C1N[C@@H](Cc2cc(-n3nncc3CCCC(=O)NCCC[C@@H]1N)ccc2)C(OC)=O	191	33.906185	0.33495575	0.84904176
O=C1N[C@@H](Cc2cc(-n3nncc3CCC(=O)NCCCC[C@@H]1N)ccc2)C(OC)=O	192	36.444851	0.4706606	0.6142593

O=C1N[C@@H](CCC(=O)N[C@@H](CCCCn2nnc(CCCC(=O)NCCCC[C@@H]1NC(OC(C)(C)C)=O)c2)C(OC)=O)C(OC)=O	193	-95.932709	0.44948643	0.73212689
O=C1N[C@@H](CCC(=O)N[C@@H](CCCn2nncc2CCCC(=O)NCCCC[C@@H]1N)C(OC)=O)C(OC)=O	194	-24.297775	0.4446933	0.78133374
O=C1N[C@@H](CCC(=O)N[C@@H](CCCn2nncc2CCC(=O)NCCC[C@@H]1N)C(OC)=O)C(OC)=O	195	-27.987408	0.57644814	0.77464485
O=C1N[C@@H](CCCn2nncc2CCC(=O)NCCC[C@@H]2NC(=O)[C@@H](NC2=O)CC1)C(OC)=O	196	-8.6262798	0.4712207	0.72942191
O=C1N[C@@H](CCC(=O)N[C@@H](CCCn2nncc2CCCC(=O)NCCC[C@@H]1N)C(OC)=O)C(OC)=O	197	-30.048773	0.45003352	0.67349052
O=C1N[C@@H](CCCn2nncc2CCCC(=O)NCCC[C@@H]2NC(=O)[C@@H](NC2=O)CC1)C(OC)=O	198	-15.7844	0.4125	0.7093
O=C1N[C@@H](CCC(=O)N[C@@H](CCCn2nncc2CNC(=O)NCCCC[C@@H]1N)C(OC)=O)C(OC)=O	199	-122.39365	0.44811743	0.75603527
O=C1N[C@@H](CCC(=O)N[C@@H](CCCn2nncc2CNC(=O)NCCC[C@@H]1N)C(OC)=O)C(OC)=O	200	-127.06362	0.62935591	0.87537467
O=C1N[C@@H](CCC(=O)N[C@@H](CCCn2nncc2CCC(=O)Nc2ccc(C[C@@H]1N)cc2)C(OC)=O)C(OC)=O	201	7.8307261	0.30977336	0.78235883
O=C1N[C@@H](CCC(=O)N[C@@H](CCCn2nncc2CCCC(=O)NCC[C@@H]1N)C(OC)=O)C(OC)=O	202	-26.434462	0.47610196	0.85899597
O=C1N[C@@H](CCC(=O)N[C@@H](CCCn2nncc2CCC(=O)NCC[C@@H]1N)C(OC)=O)C(OC)=O	203	-25.989042	0.69431037	0.80237925
O=C1N[C@@H](CCC(=O)N[C@@H](CCCn2nncc2CCCC(=O)NC[C@@H]1N)C(OC)=O)C(OC)=O	204	-7.7790523	0.42934021	0.71295428
O=C1N[C@@H](CCC(=O)N[C@@H](CCCn2nncc2CCC(=O)NC[C@@H]1N)C(OC)=O)C(OC)=O	205	-5.7714996	0.72950101	0.8010062
O=C1N[C@@H](CCC(=O)N[C@@H](CCCn2nncc2CNC(=O)NCC[C@@H]1N)C(OC)=O)C(OC)=O	206	-126.45739	0.52185494	0.77277541
O=C1N[C@@H](CCC(=O)N[C@@H](CCCn2nnc(CNC(=O)NC[C@@H]1N)c2)C(OC)=O)C(OC)=O	207	-84.290054	0.50842738	0.67850226
O=C1N[C@@H](CCC(=O)N[C@@H](CCCn2nncc2CNC(=O)NC[C@@H]1N)C(OC)=O)C(OC)=O	208	-103.88399	0.52908731	0.92948282
O=C1N[C@@H](CCC(=O)N[C@@H](CCCn2nnc(CCCNCCCC[C@@H]1N)c2)C(OC)=O)C(OC)=O	209	-1.1749822	0.42554763	0.68725944
O=C1N[C@@H](CCC(=O)N[C@@H](CCCn2nncc2CCCNCCCC[C@@H]1N)C(OC)=O)C(OC)=O	210	3.4882541	0.49718201	0.72403628
O=C1N[C@@H](CCCn2nncc2CCC(=O)NCCCC[C@@H]2NC(=O)[C@@H](NC2=O)CC1)C(OC)=O	211	-11.667913	0.49097568	0.68537521
O=C1N[C@@H](CCCn2nnc(CCCC(=O)Nc3ccc(C[C@H](N)C(=O)N[C@@H]1C)cc3)c2)C(OC)=O	212	19.944044	0.46983764	0.81145644
O=C1N[C@@H](CCCn2nncc2CCCC(=O)Nc2ccc(C[C@H](N)C(=O)N[C@@H]1C)cc2)C(OC)=O	213	22.093369	0.4305861	0.81929398
O=C1NCCCC[C@H](NC(=O)CCNC(=O)[C@@H](N)CCCNC(=O)CCCc2n(nnc2)[C@H]1Cc1ccccc1)C(OC)=O	214	-44.537167	0.59666556	0.68835348

O=C1NCCCC[C@H](NC(=O)CCNC(=O)[C@@H](N)CCNC(=O)CCc2n(nnc2)[C@H]1Cc1ccccc1)C(OC)=O	215	-48.75119	0.26812798	0.90605903
O=C1N[C@H](C)C(=O)N[C@@H](CCCCNC(=O)[C@@H](n2nncc2CCCC(=O)NCC[C@H](N)C(=O)N[C@H]1Cc1ccccc1)Cc1ccccc1)C(OC)=O	216	6.6724072	0.69883889	0.86827999
O=C1N[C@H](C)C(=O)N[C@@H](CCCCNC(=O)[C@@H](n2nncc2CCCC(=O)NC[C@H](N)C(=O)N[C@H]1Cc1ccccc1)Cc1ccccc1)C(OC)=O	217	28.431448	0.48605844	0.7899878
O=C1N[C@H](C)C(=O)N[C@@H](CCCn2nncc2CCCC(=O)Nc2ccc(C[C@H](N)C(=O)N[C@H]1Cc1ccccc1)cc2)C(OC)=O	218	48.042835	0.48135337	0.7123825
O=C1NCCC(=O)N[C@@H](CCCCNC(=O)[C@@H](n2nncc2CCC(=O)NCC[C@H](N)C(=O)N[C@H]1Cc1ccccc1)Cc1ccccc1)C(OC)=O	219	-22.956503	0.44217589	0.7771526

18. References

[1] L.-H. Zhang, J. A. Kauffman, J. A. Pesti, J. Yin, *J. Org. Chem.* **1997**, *62*, 6918-6920.

[2] a) R. Mitra, K. N. Ganesh, *Chem. Commun.* **2011**, *47*, 1198-1200; b) C. Yu, J. W. Taylor, *Bioorg. Med. Chem.* **1999**, *7*, 161-175.

[3] M. D. Simon, K. M. Shokat, *J. Am. Chem. Soc.* **2004**, *126*, 8078-8079.

[4] E. A. Alemán, H. S. Pedini, D. Rueda, *ChemBioChem* **2009**, *10*, 2862-2866.

[5] B. Jagadish, R. Sankaranarayanan, L. Xu, R. Richards, J. Vagner, V. J. Hruby, R. J. Gillies, E. A. Mash, *Bioorg. Med. Chem. Lett.* **2007**, *17*, 3310-3313.

[6] A. Isidro-Llobet, T. Murillo, P. Bello, A. Cilibrizzi, J. T. Hodgkinson, W. R. J. D. Galloway, A. Bender, M. Welch, D. R. Spring, *Proc. Natl. Acad. Sci. U. S. A.* **2011**, *108*, 6793-6798.

[7] a) R. Mitra, K. N. Ganesh, *J. Org. Chem.* **2012**, *77*, 5696-5704; b) S. S. More, R. Vince, *J. Med. Chem.* **2008**, *51*, 4581-4588.

[8] a) D. Liu, J. Dong, Y. Yin, R. Ma, Y. Shi, H. Wu, S. Chen, G. Li, *Chin. J. Chem.* **2011**, *29*, 1489-1502; b) C. Couturier, J. Blanchet, T. Schlama, J. Zhu, *Org. Lett.* **2006**, *8*, 2183-2186.

[9] K. M. G. O'Connell, H. S. G. Beckmann, L. Laraia, H. T. Horsley, A. Bender, A. R. Venkitaraman, D. R. Spring, *Org. Biomol. Chem., 2010, 10, 7545–7551* **2010**, *10*, 7545-7551.

[10] W. Zhu, D. Ma, *Chem. Commun.* **2004**, *0*, 888-889.

[11] Chemical Computing Group Inc. (2012). Molecular Operating Environment (MOE) 2012.10, 1010 Sherbooke St. West, Suite #910, Montreal, QC, Canada, H3A 2R7.

[12] R. A. Bauer, J. M. Wurst, D. S. Tan, *Curr. Opin. Chem. Bio.* **2010**, *14*, 308-314.

19. NMR Spectra

Building block B

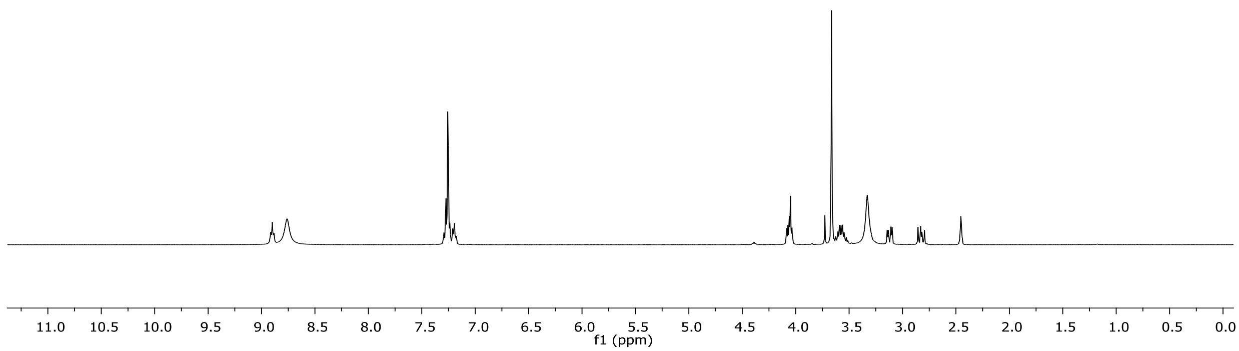

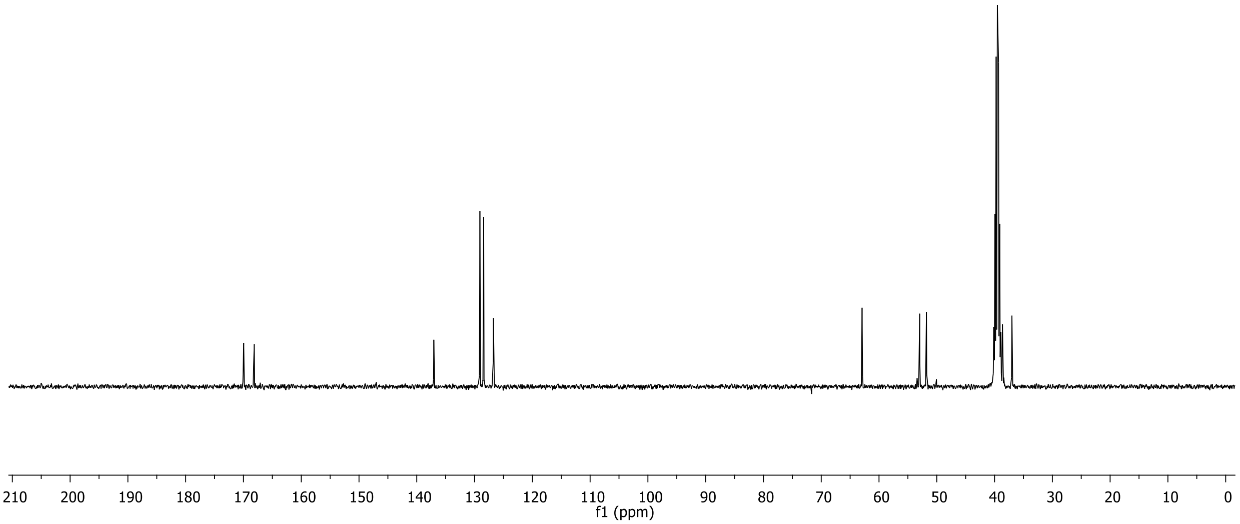

Building block C

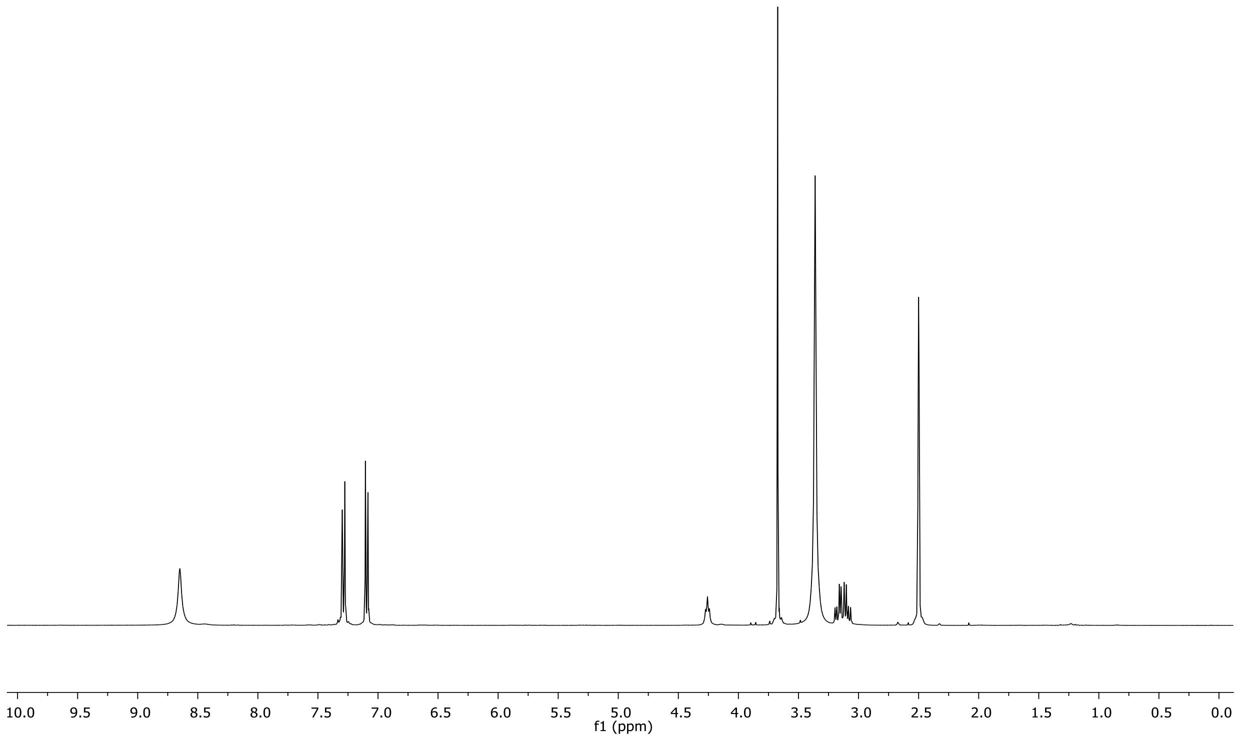

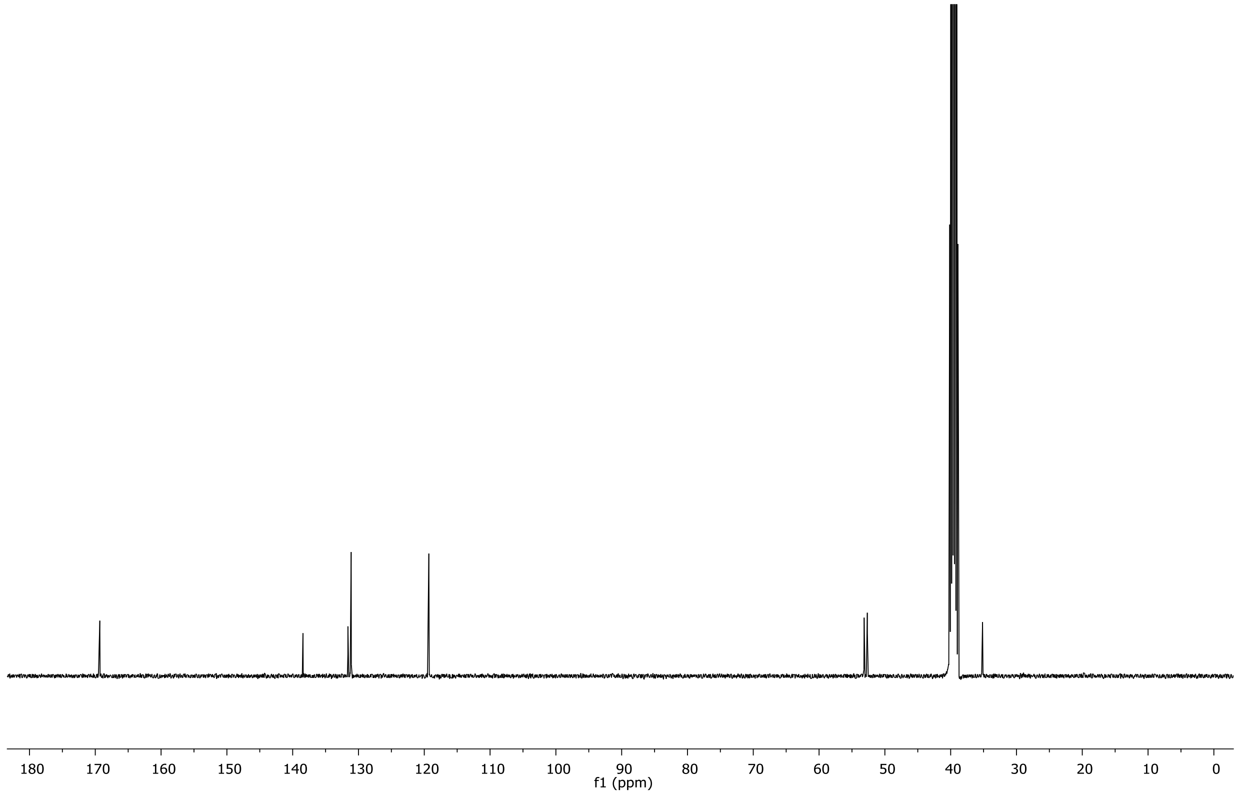

Building block D

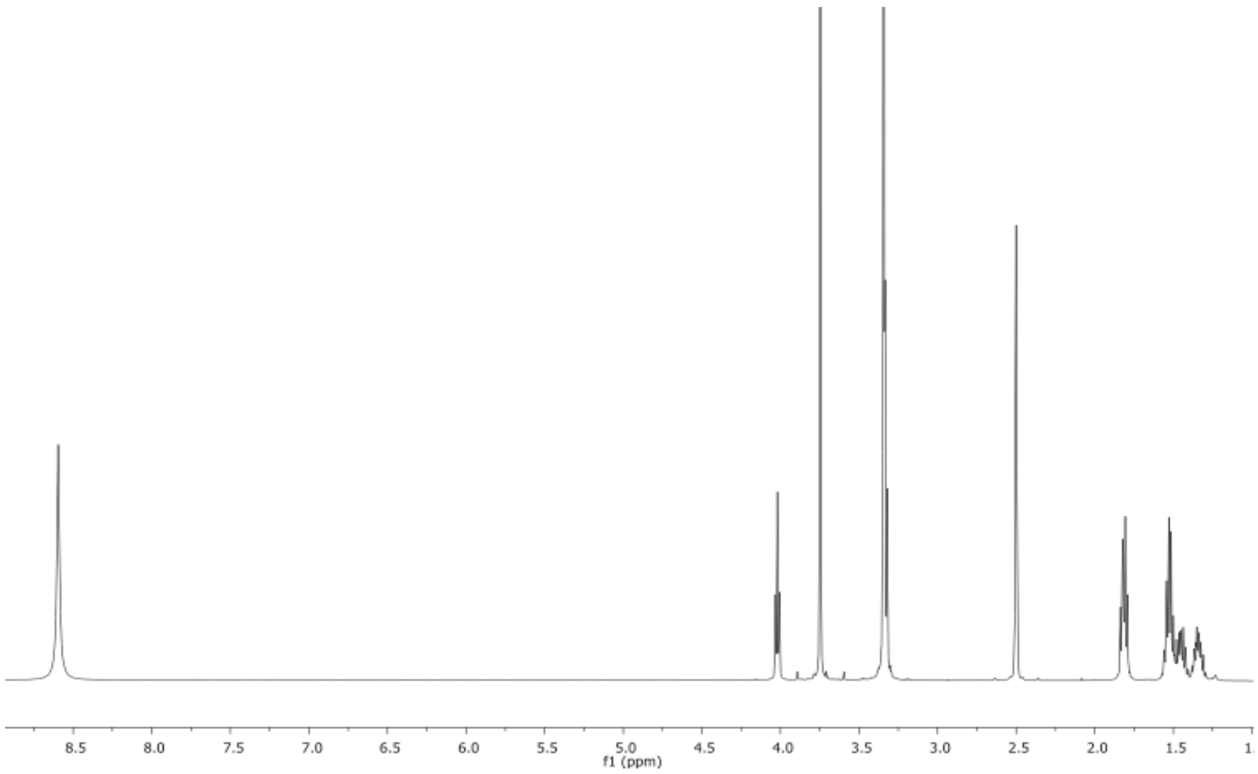

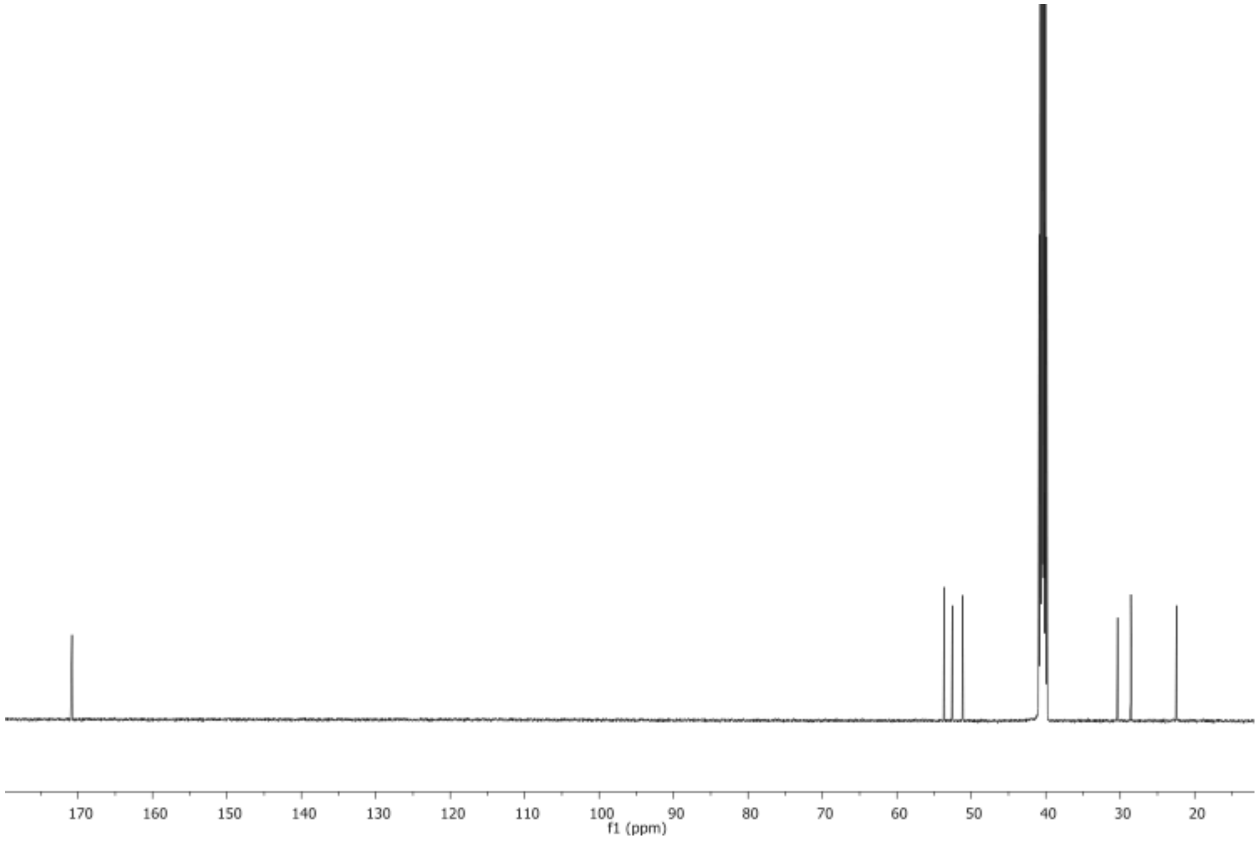

Building block E

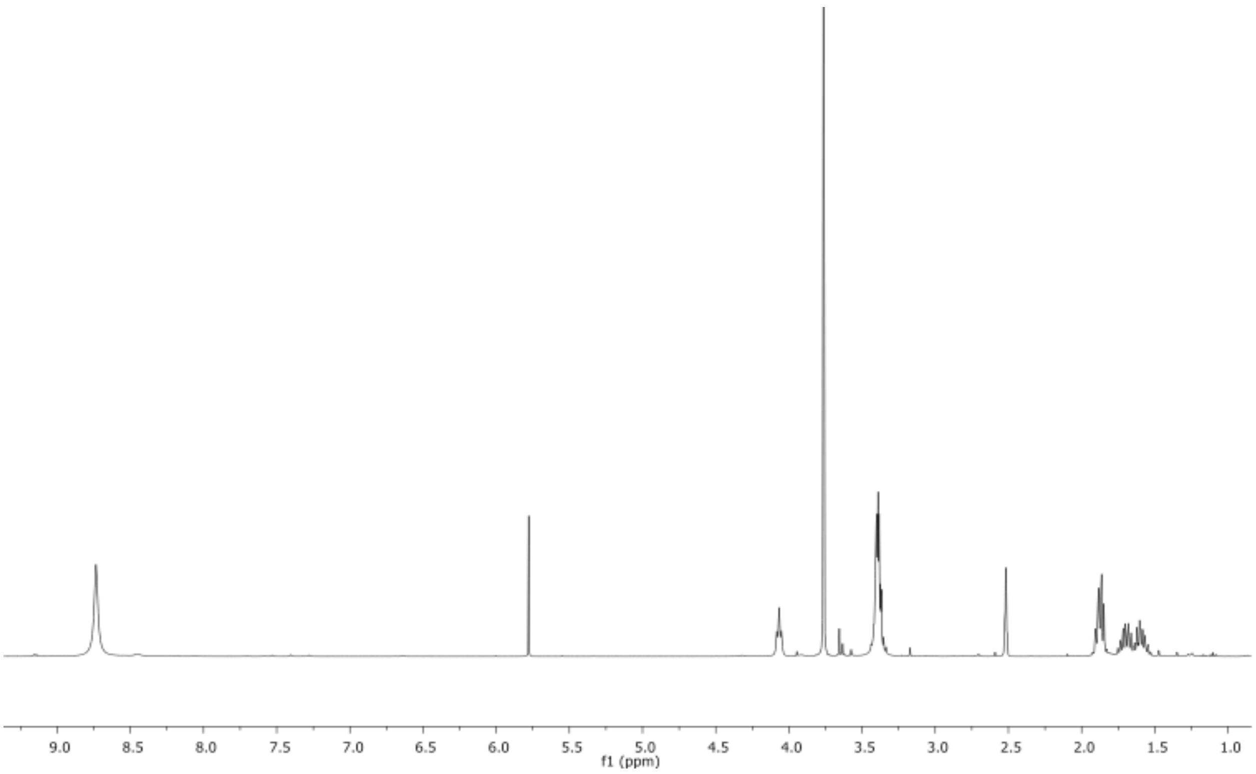

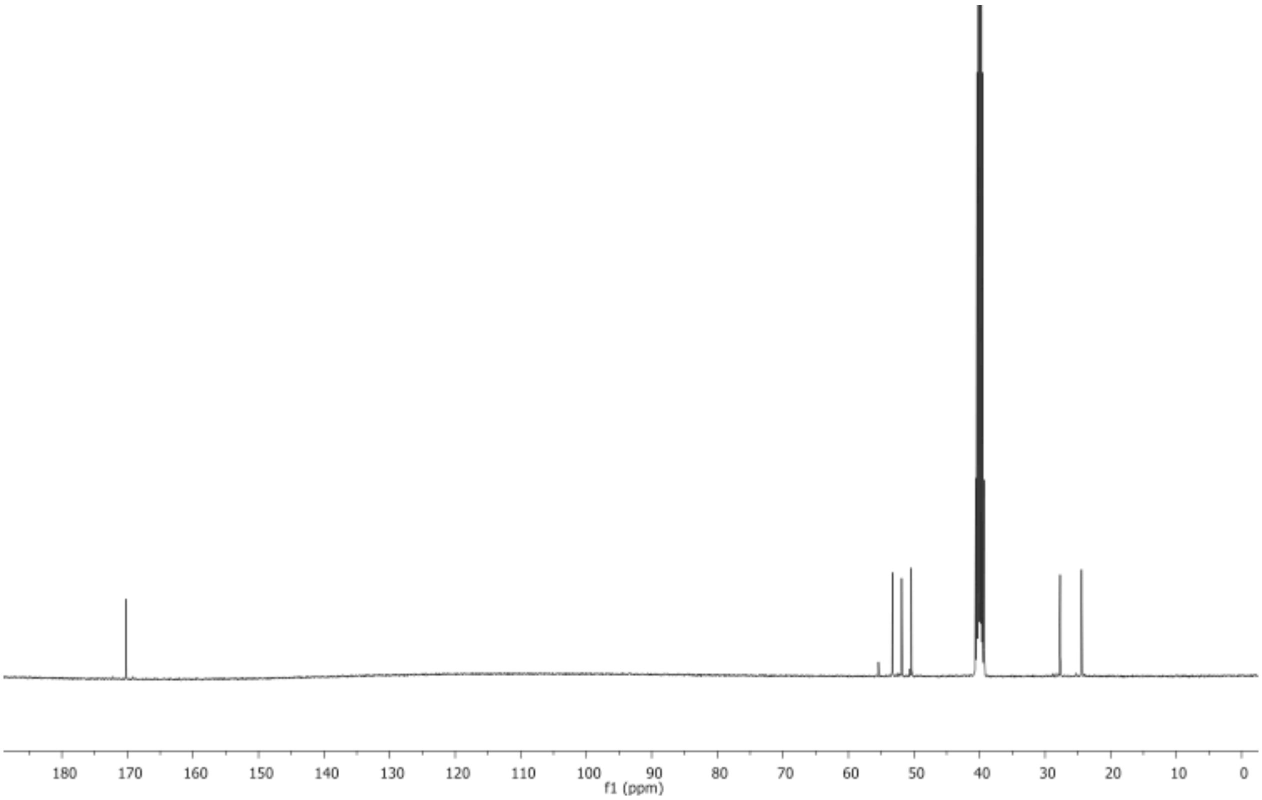

Building block F

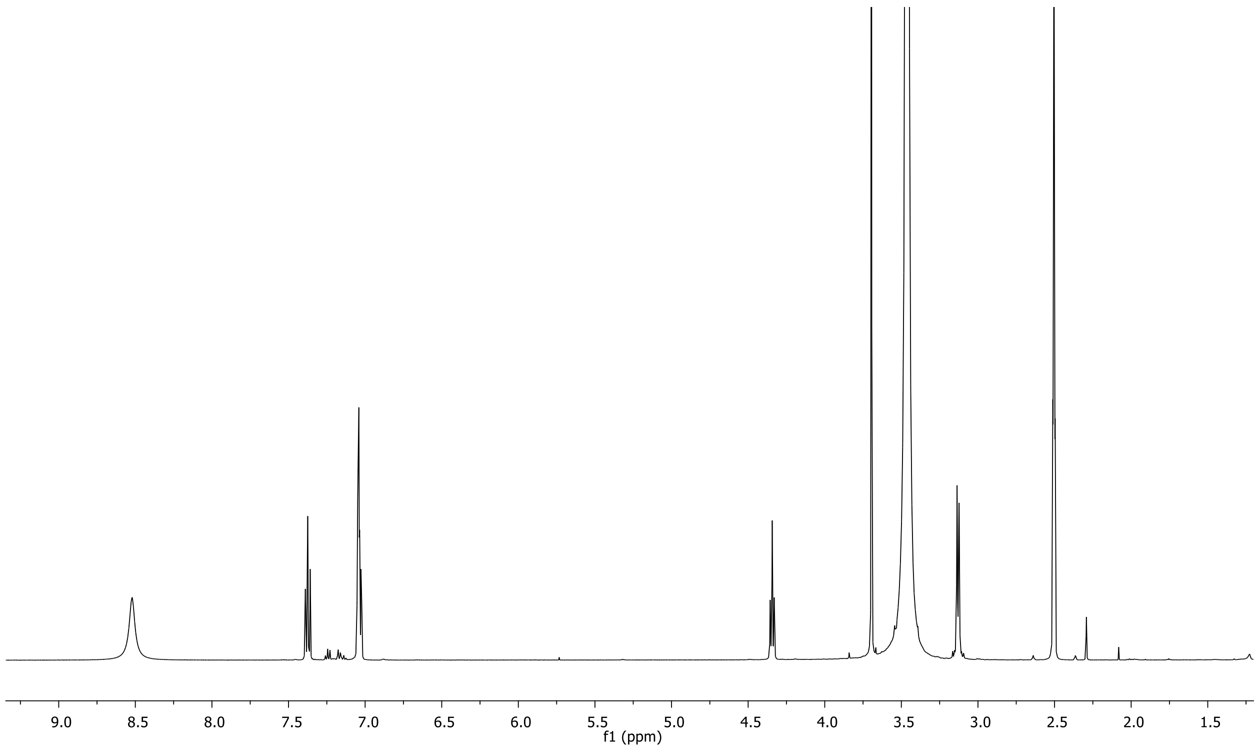

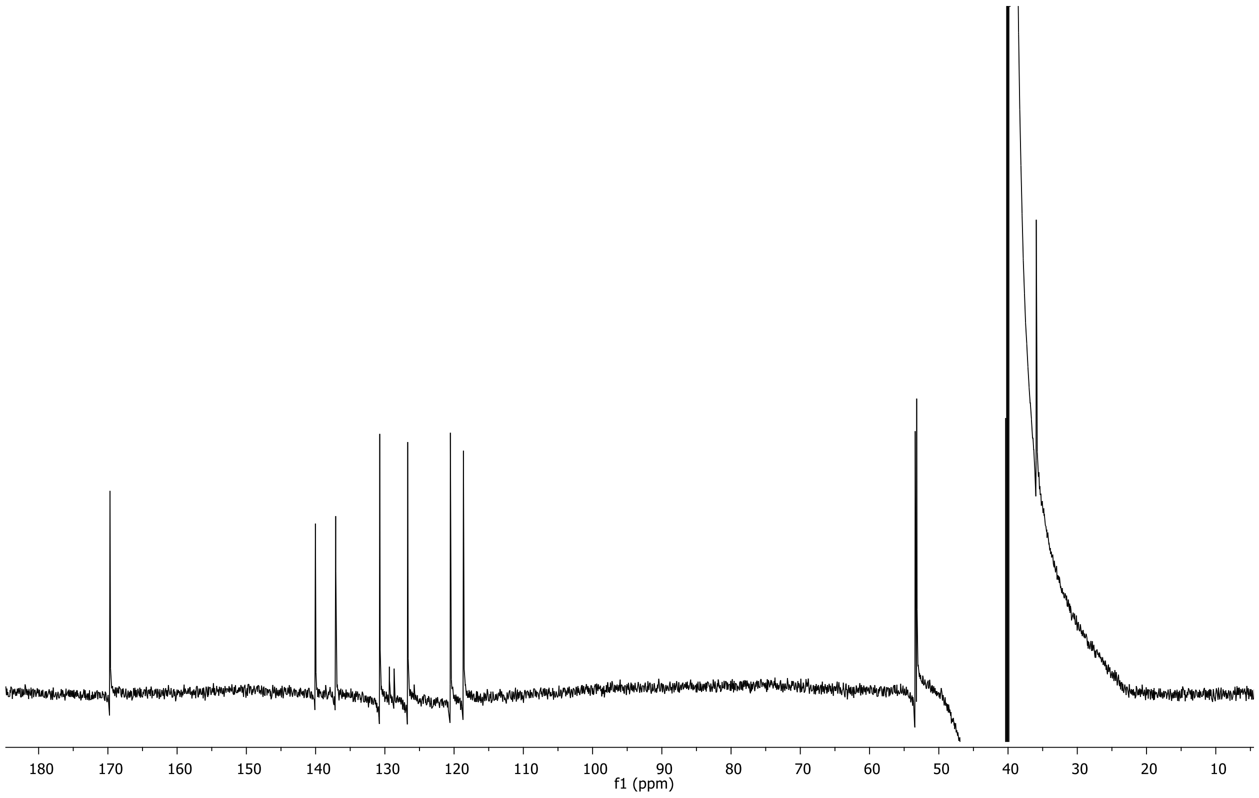

Building block 1

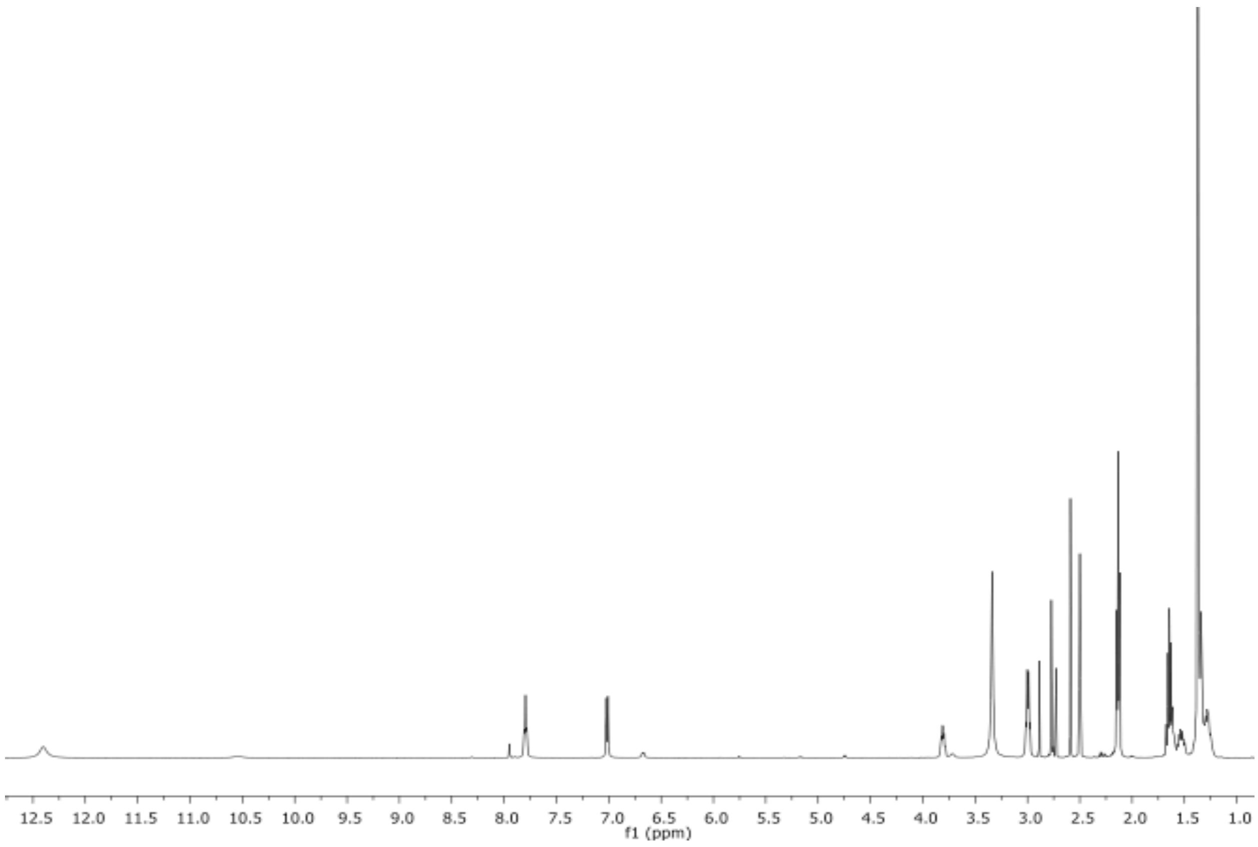

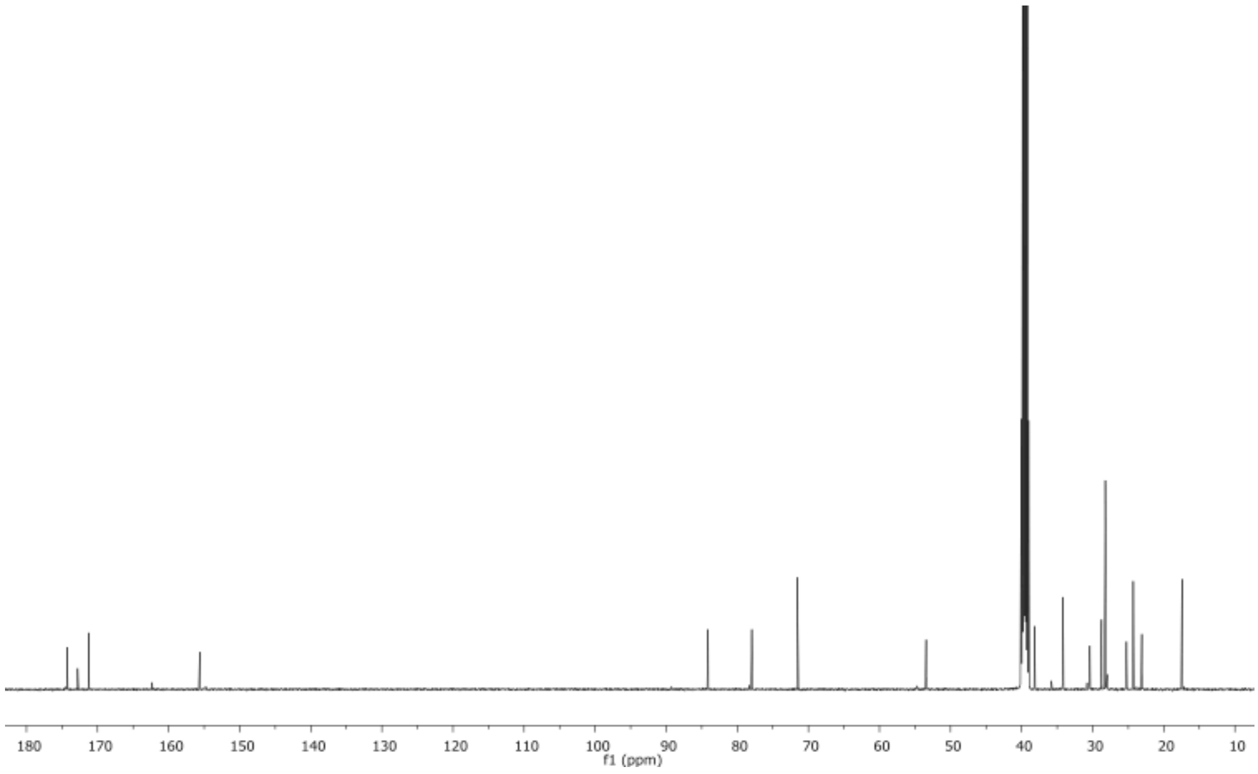

Building block 2

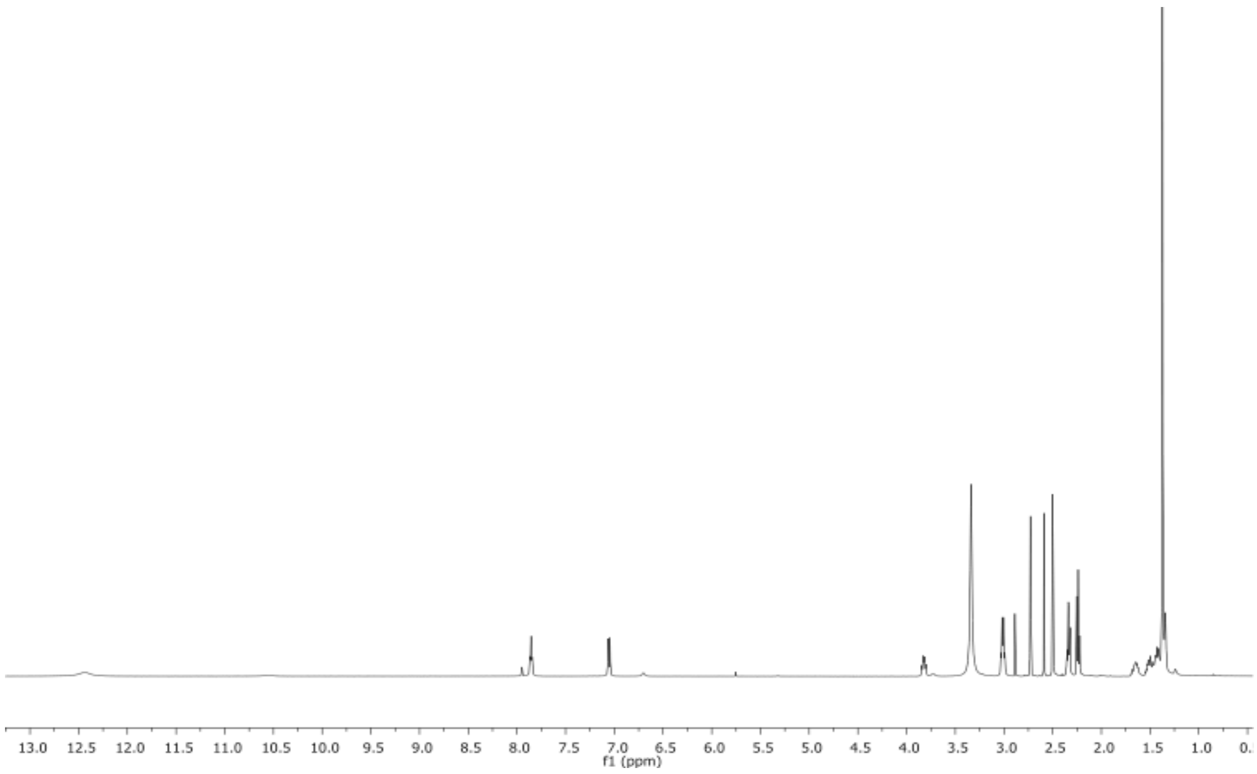

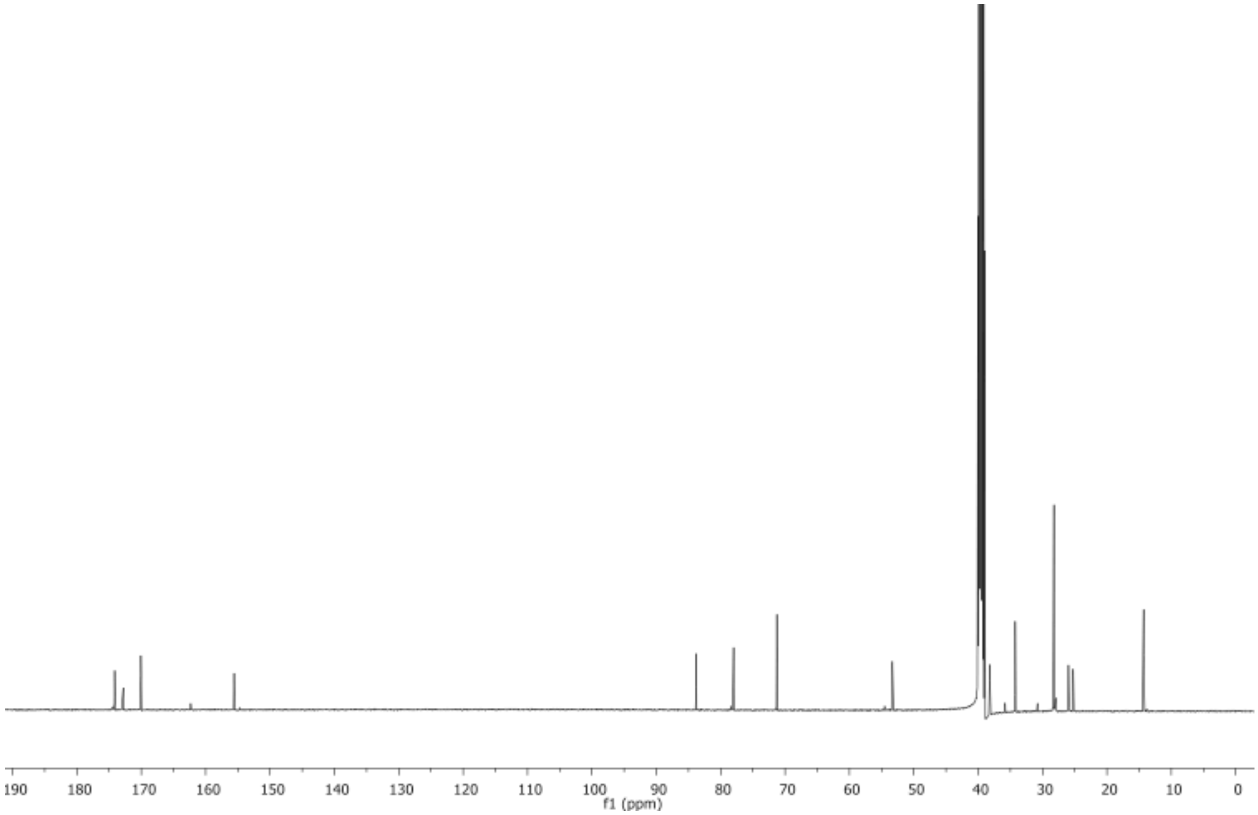

Building block 3

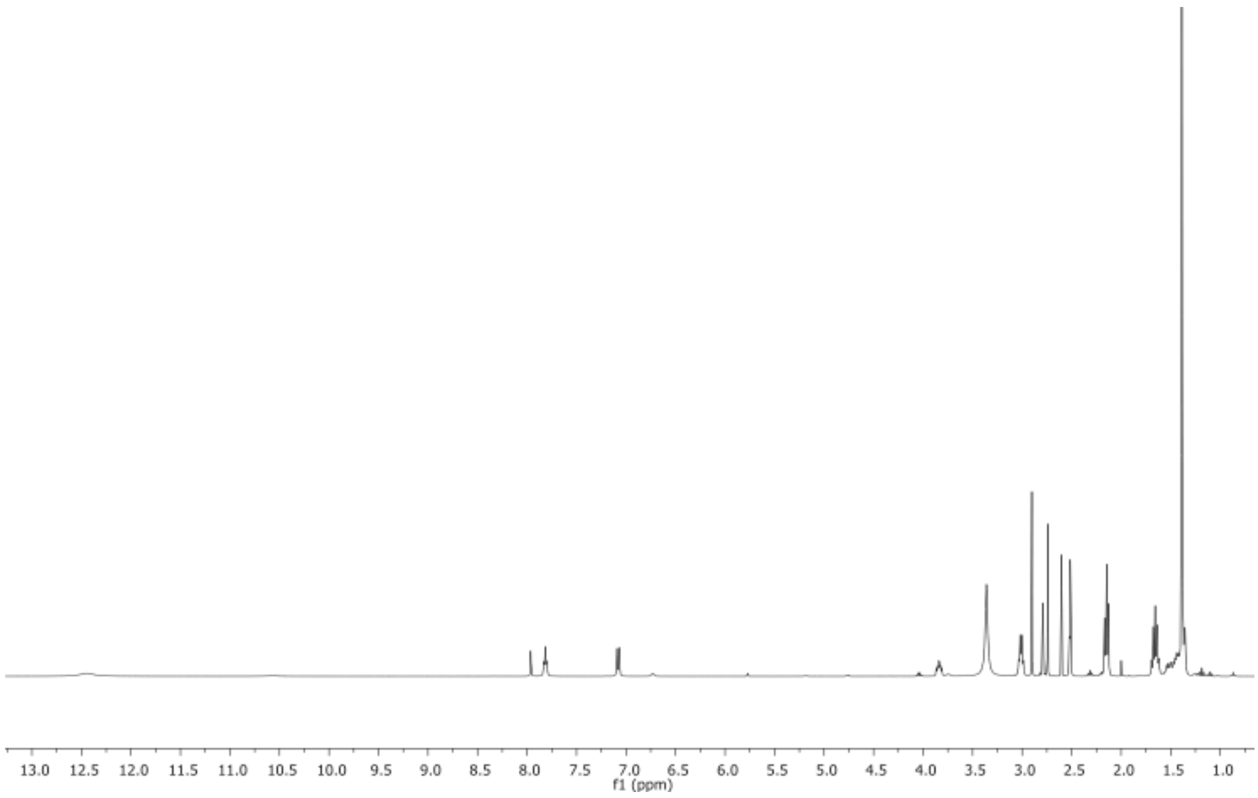

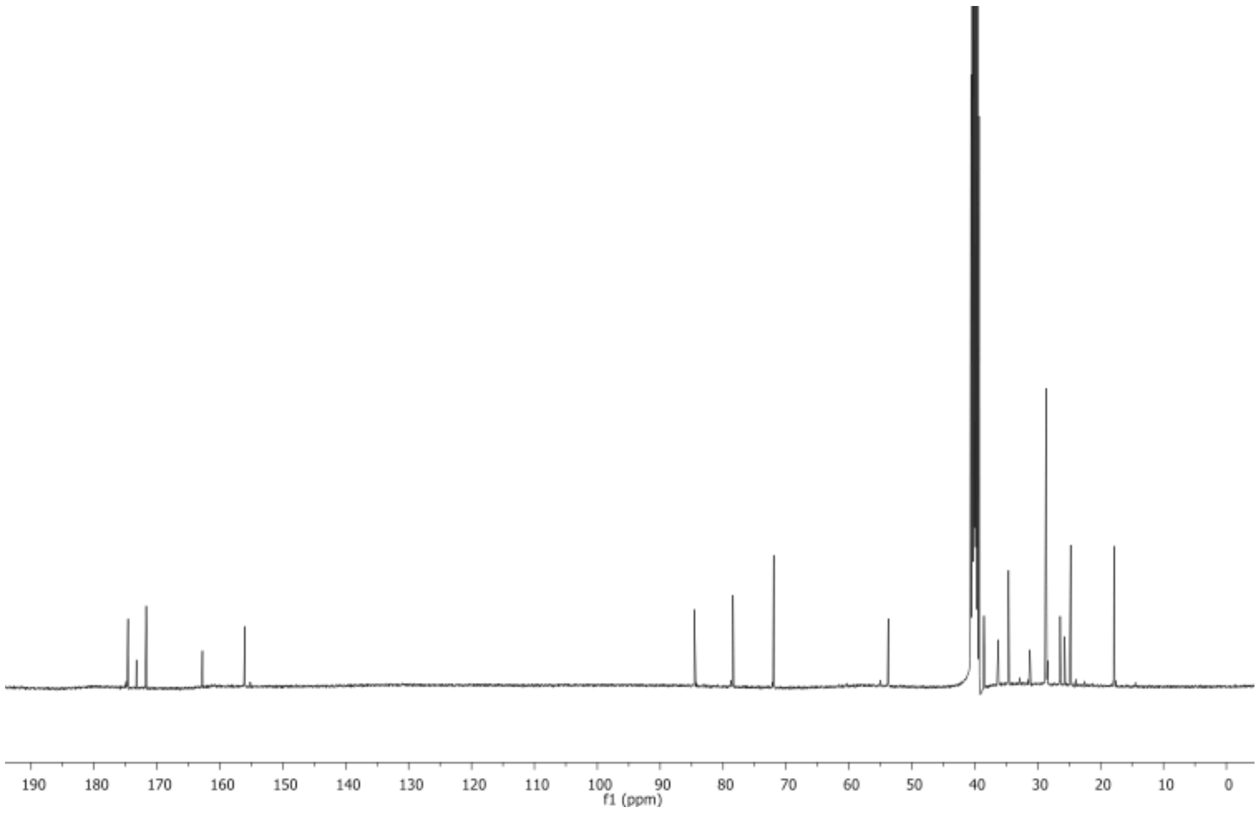

Building block 4

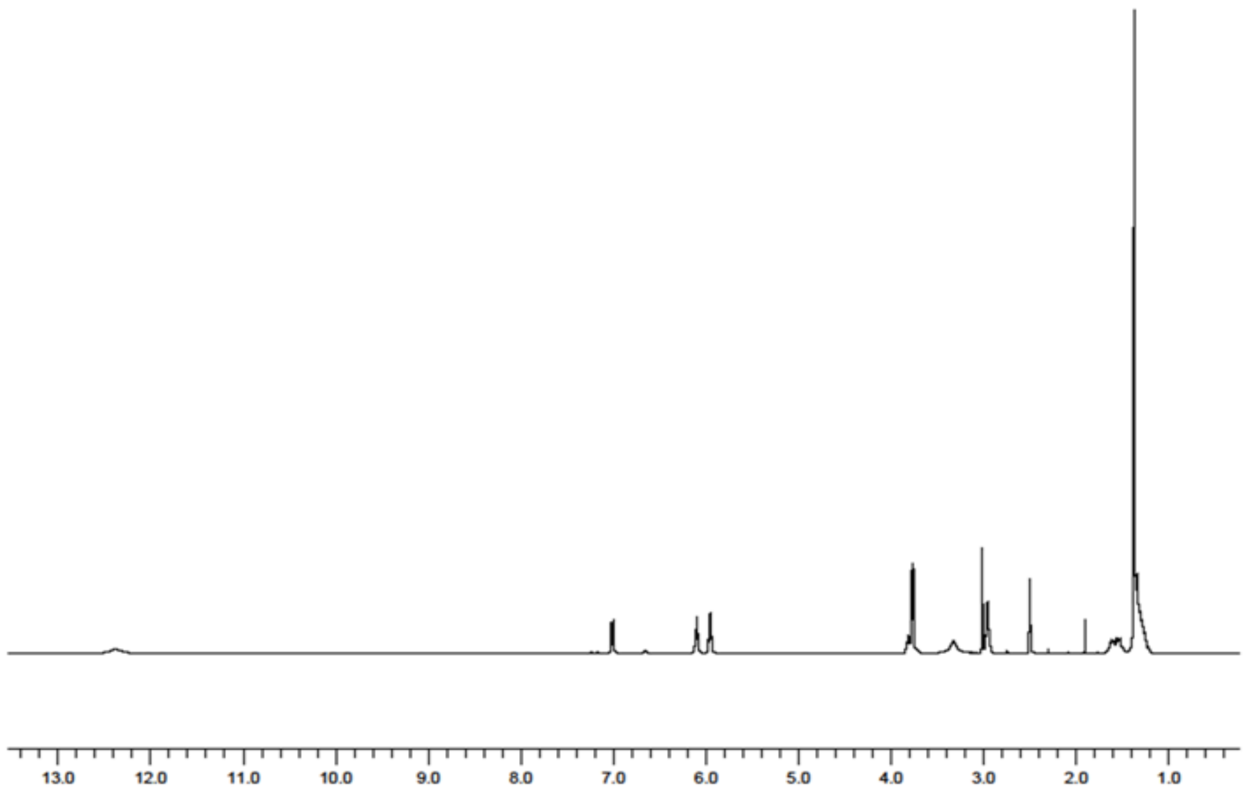

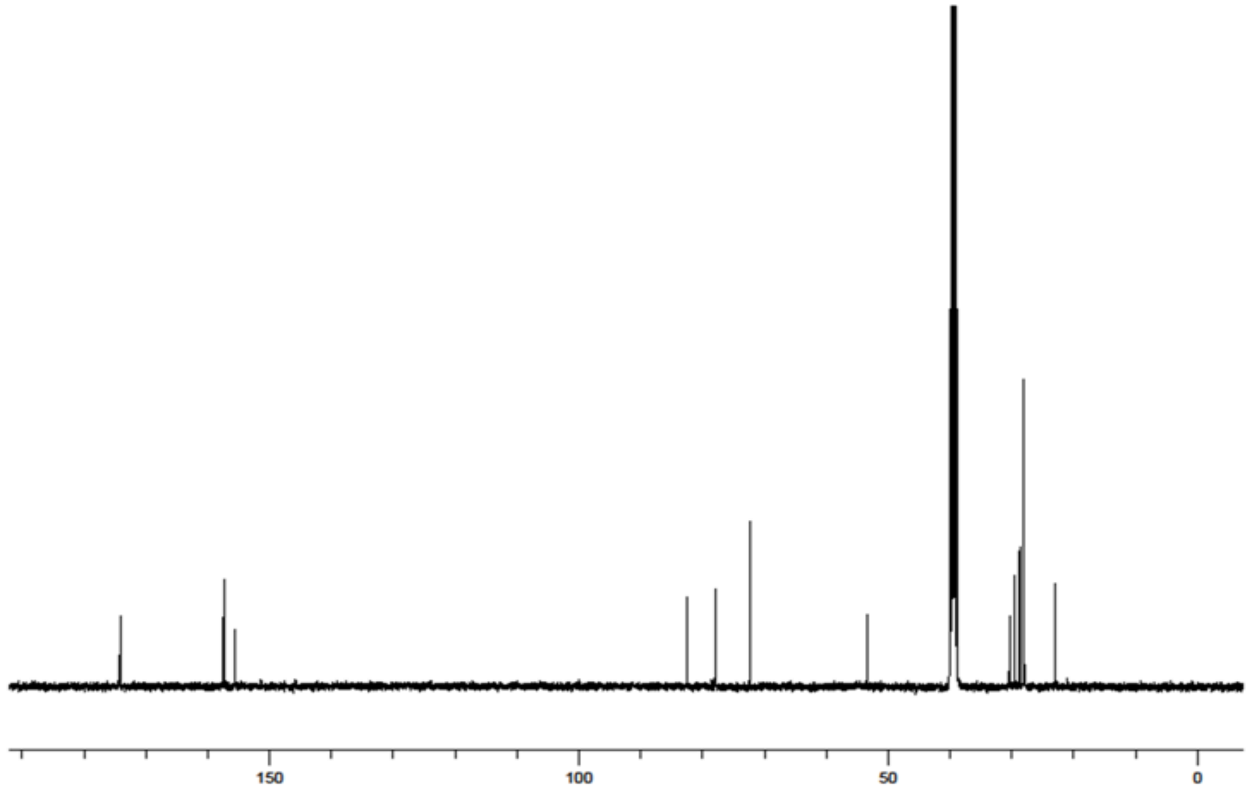

Building block 5

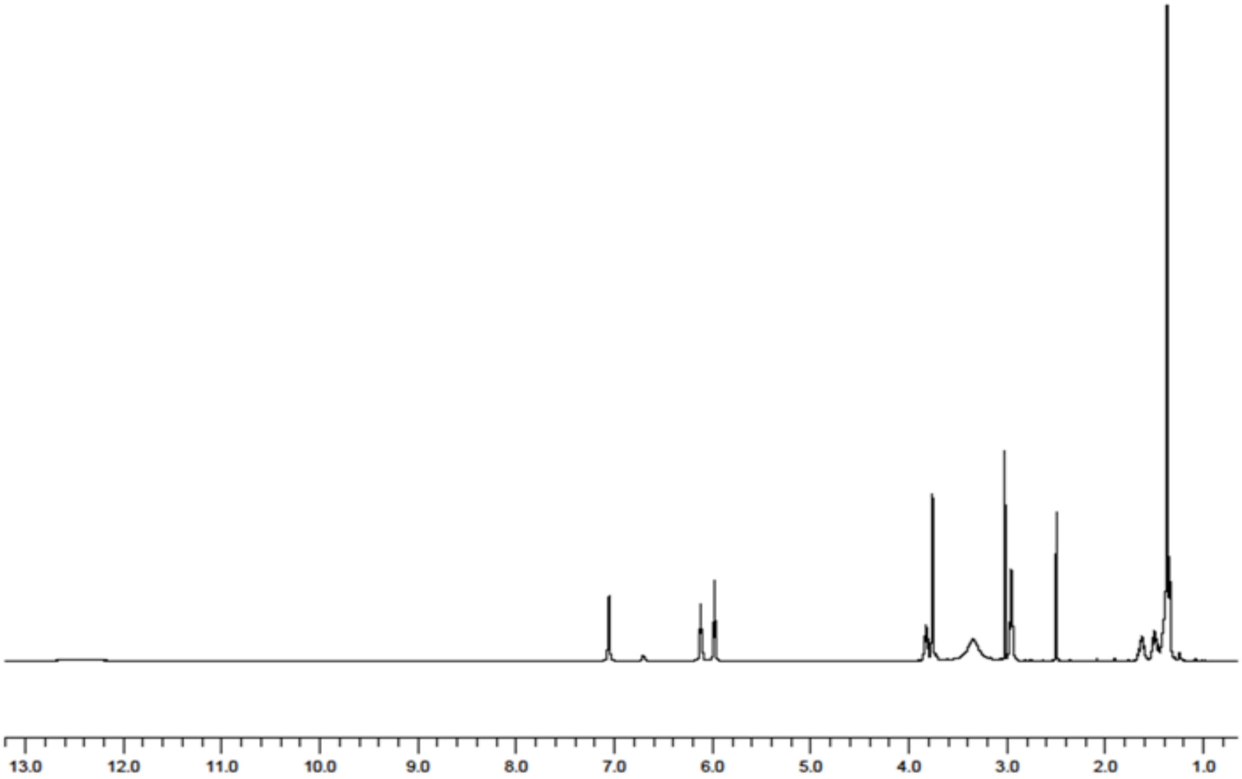

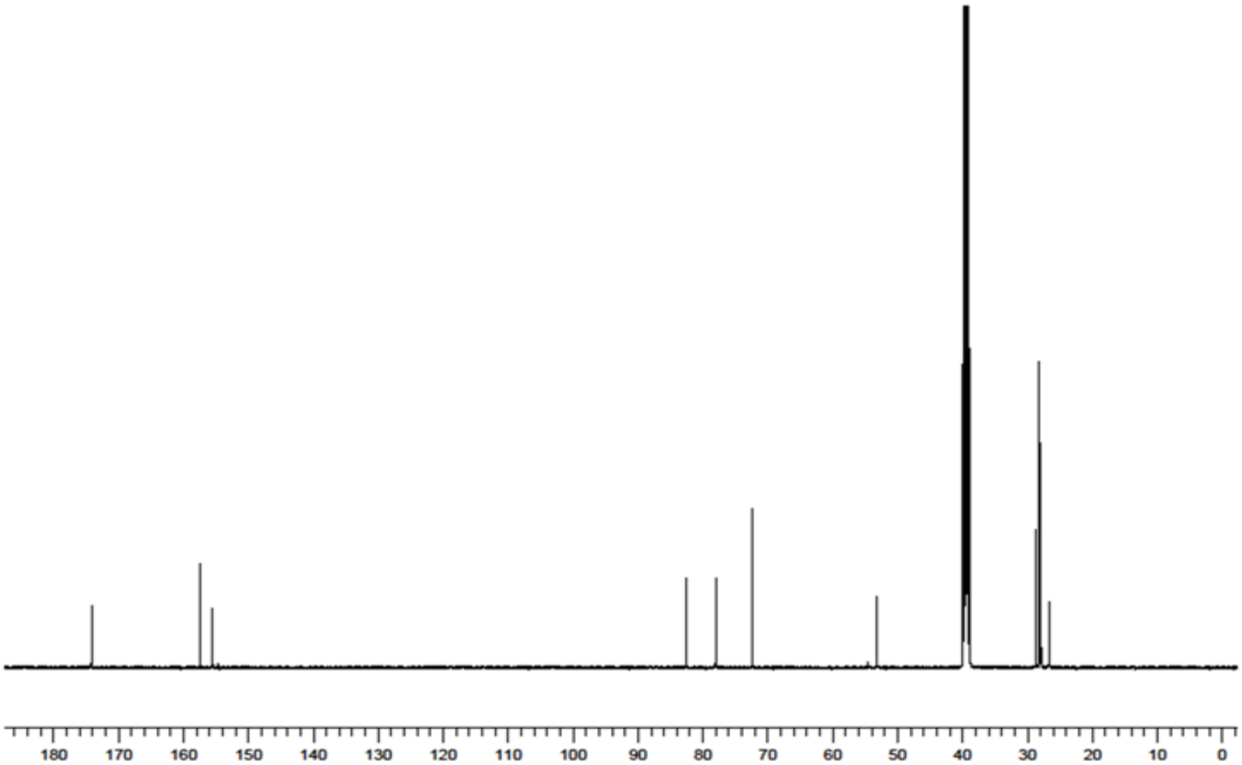

Building block 7

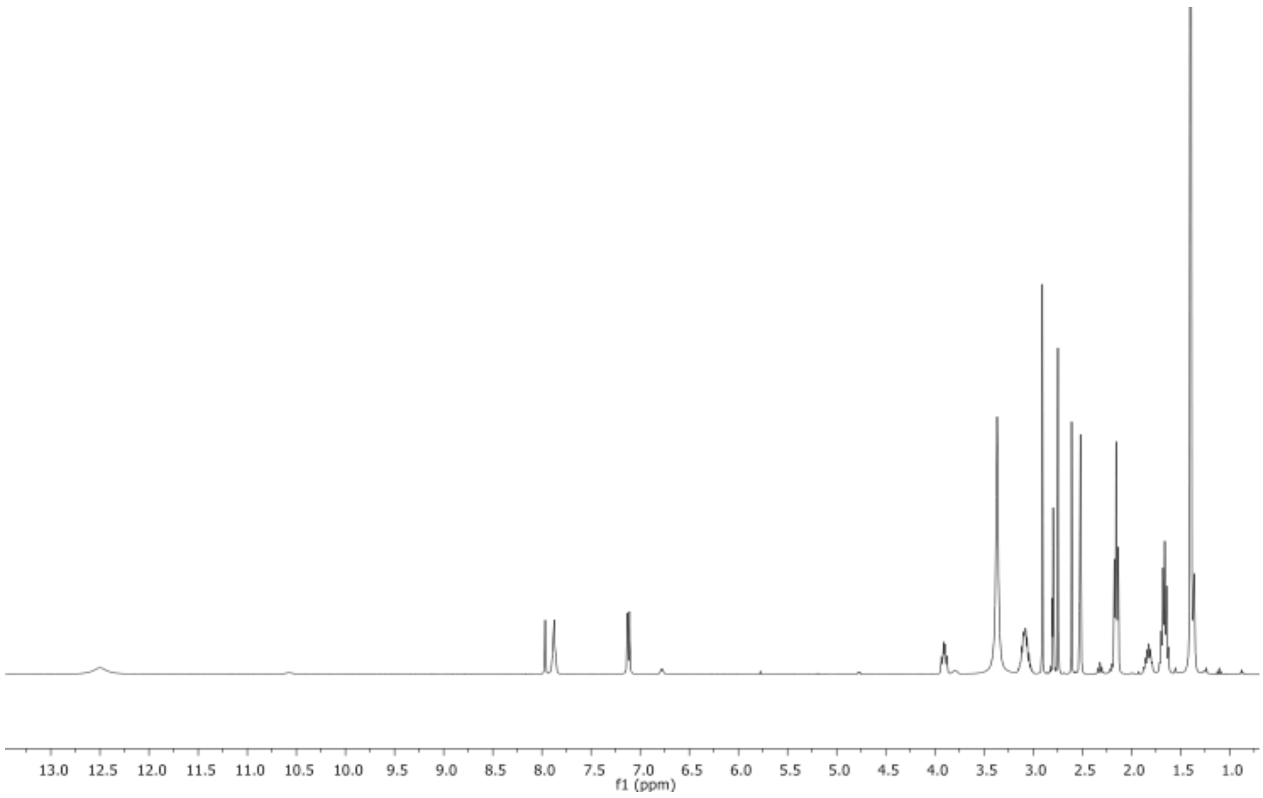

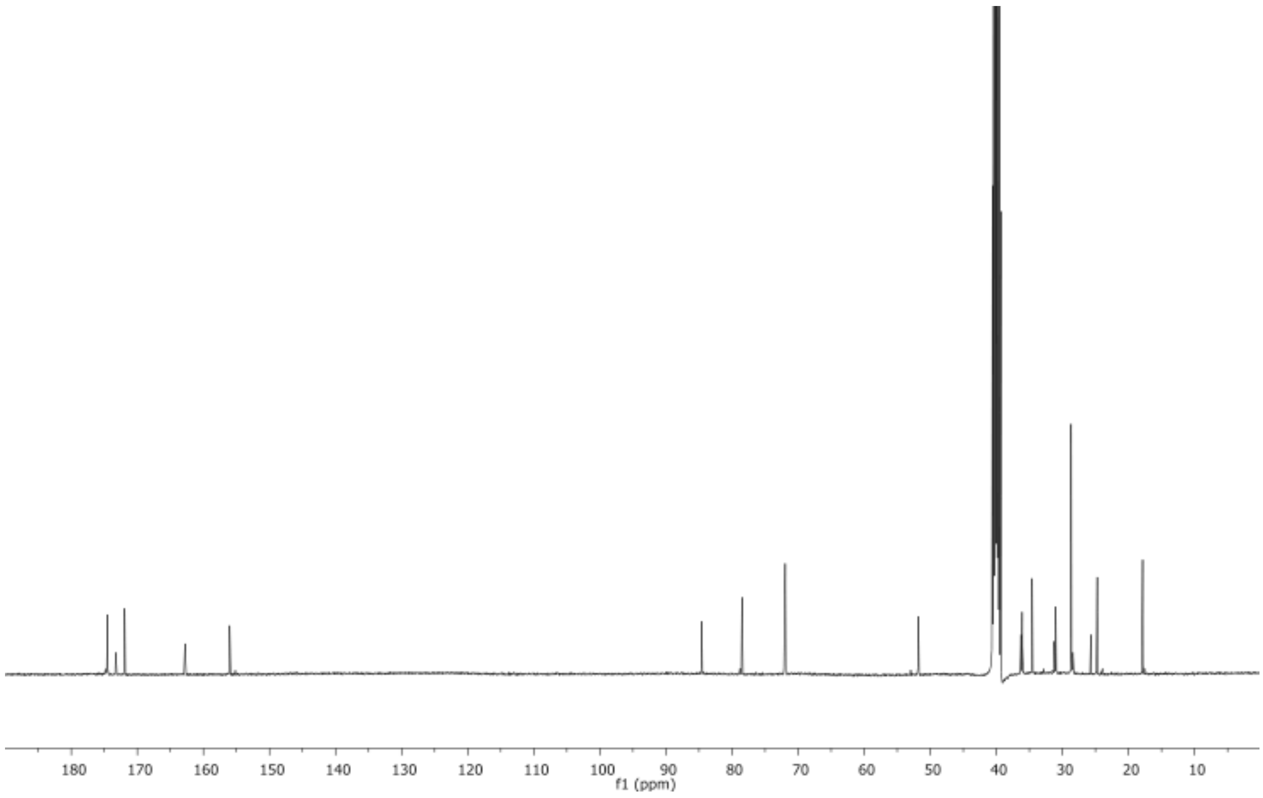

Building block 8

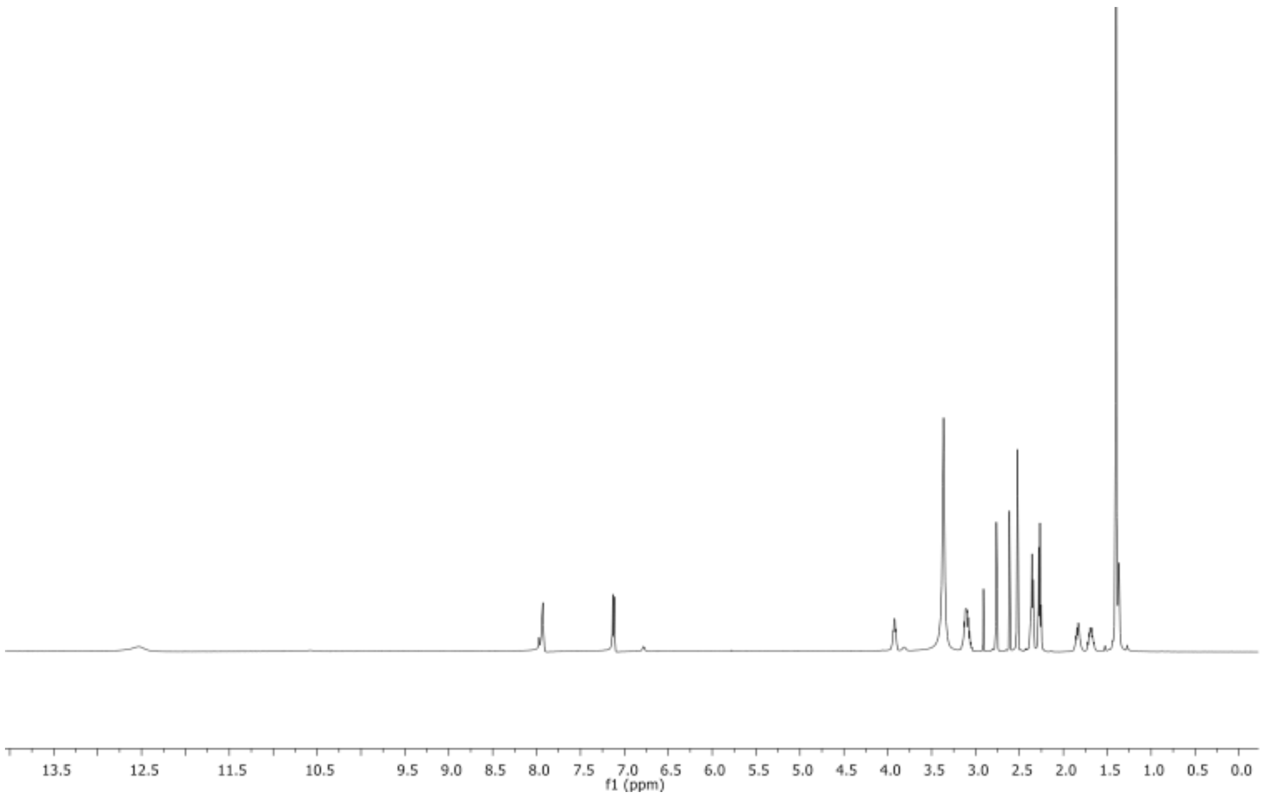

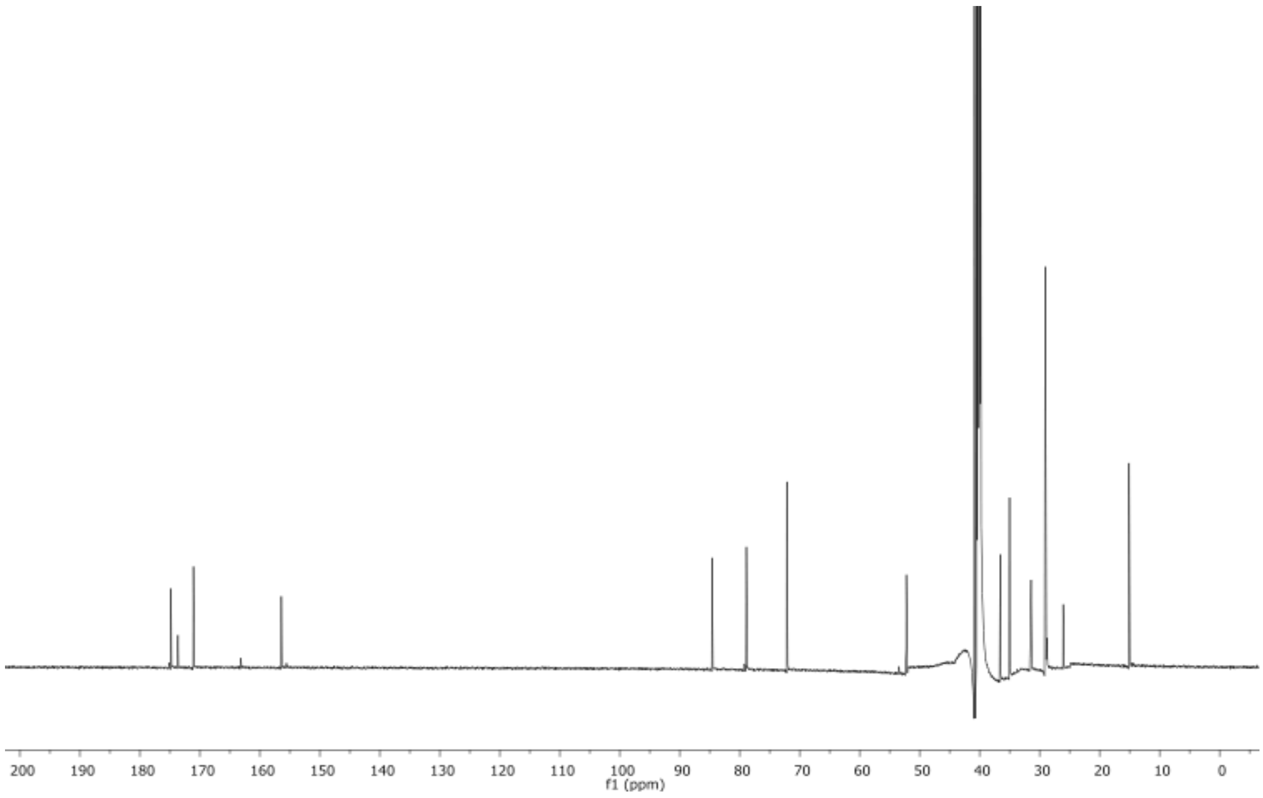

Building block 9

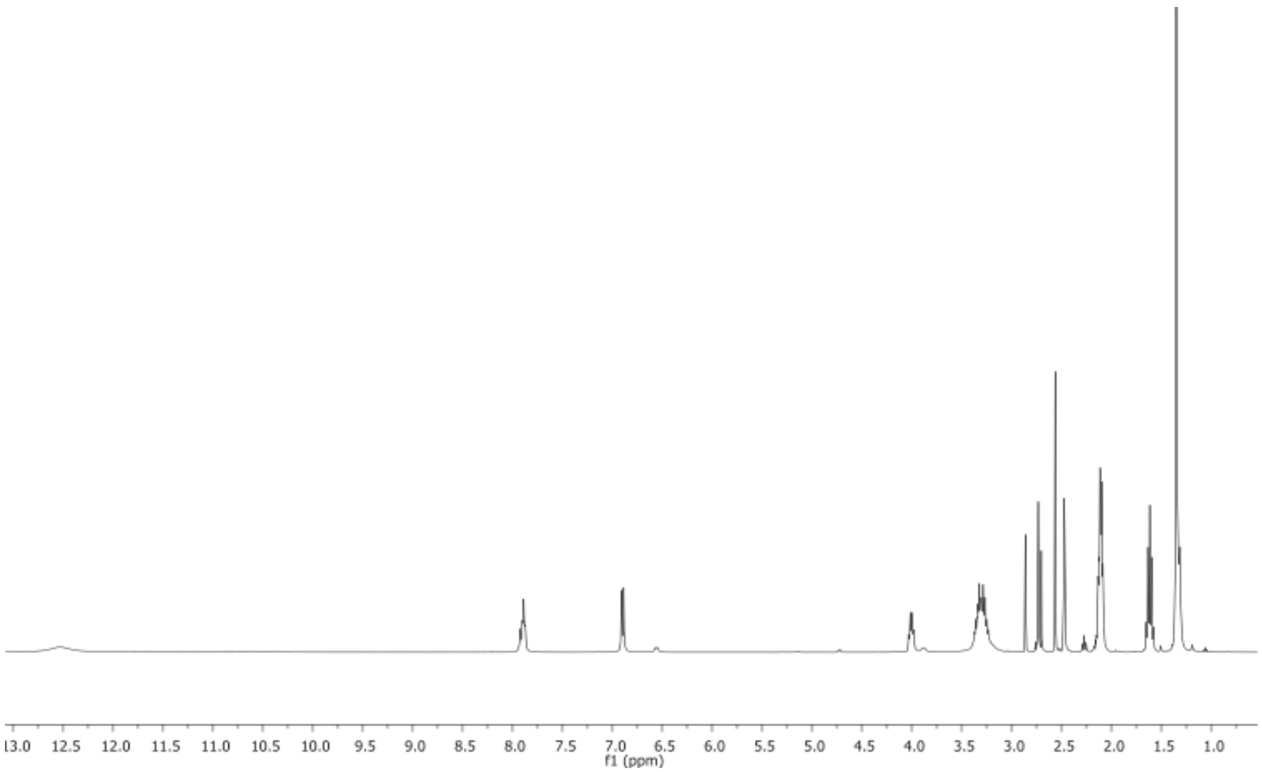

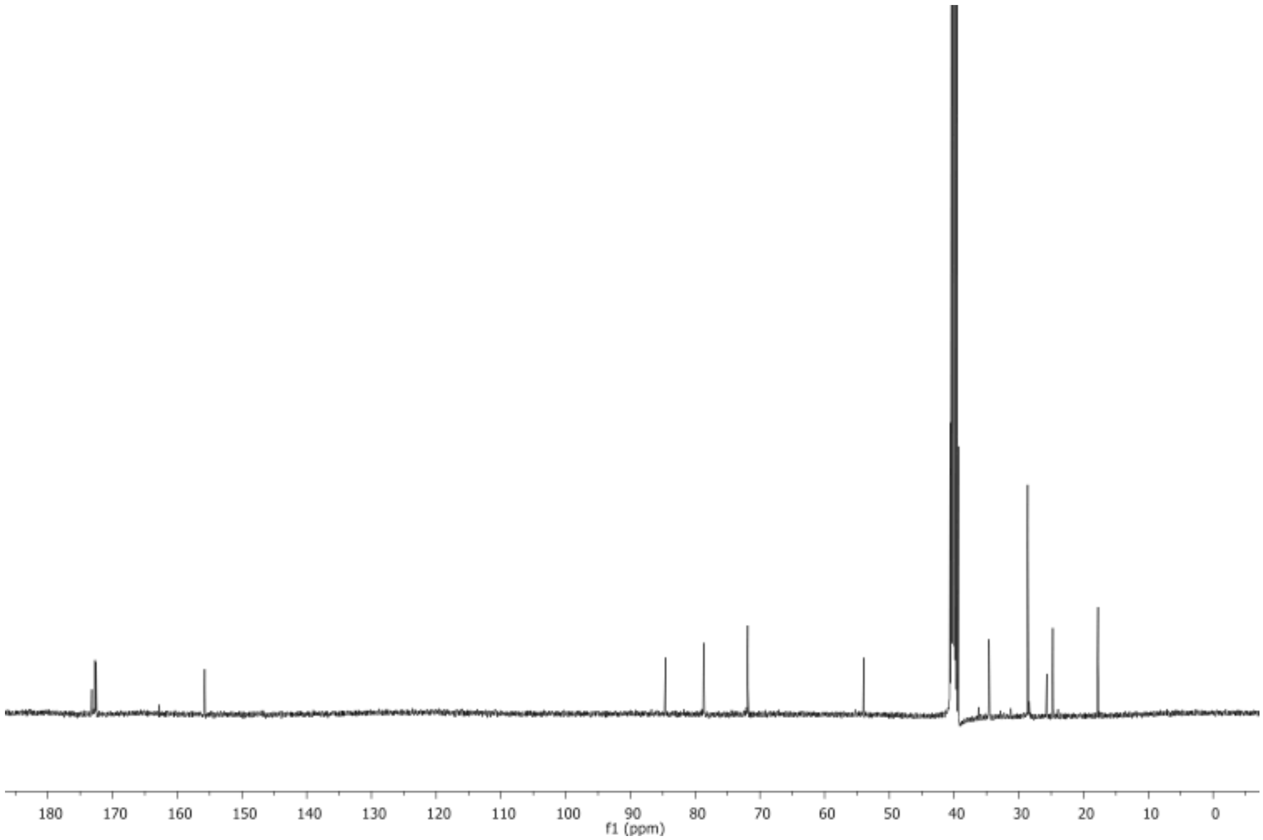

Building block 10

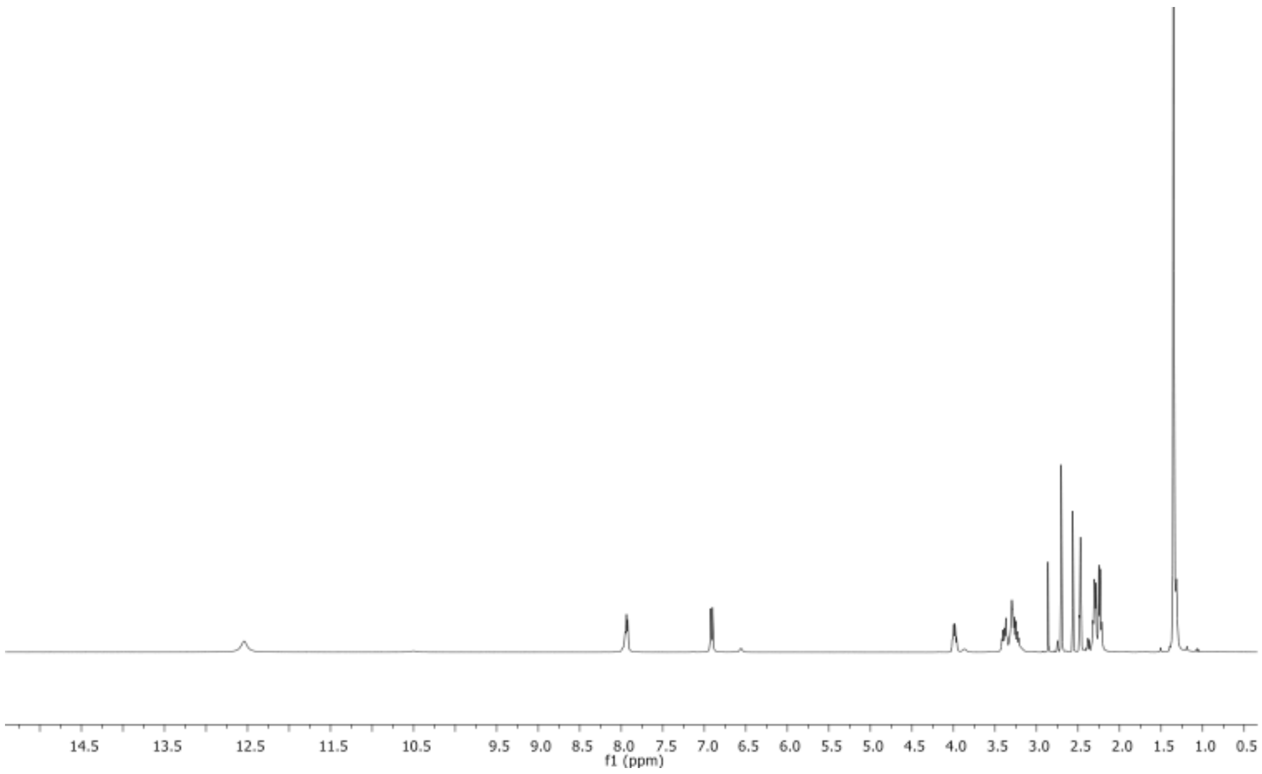

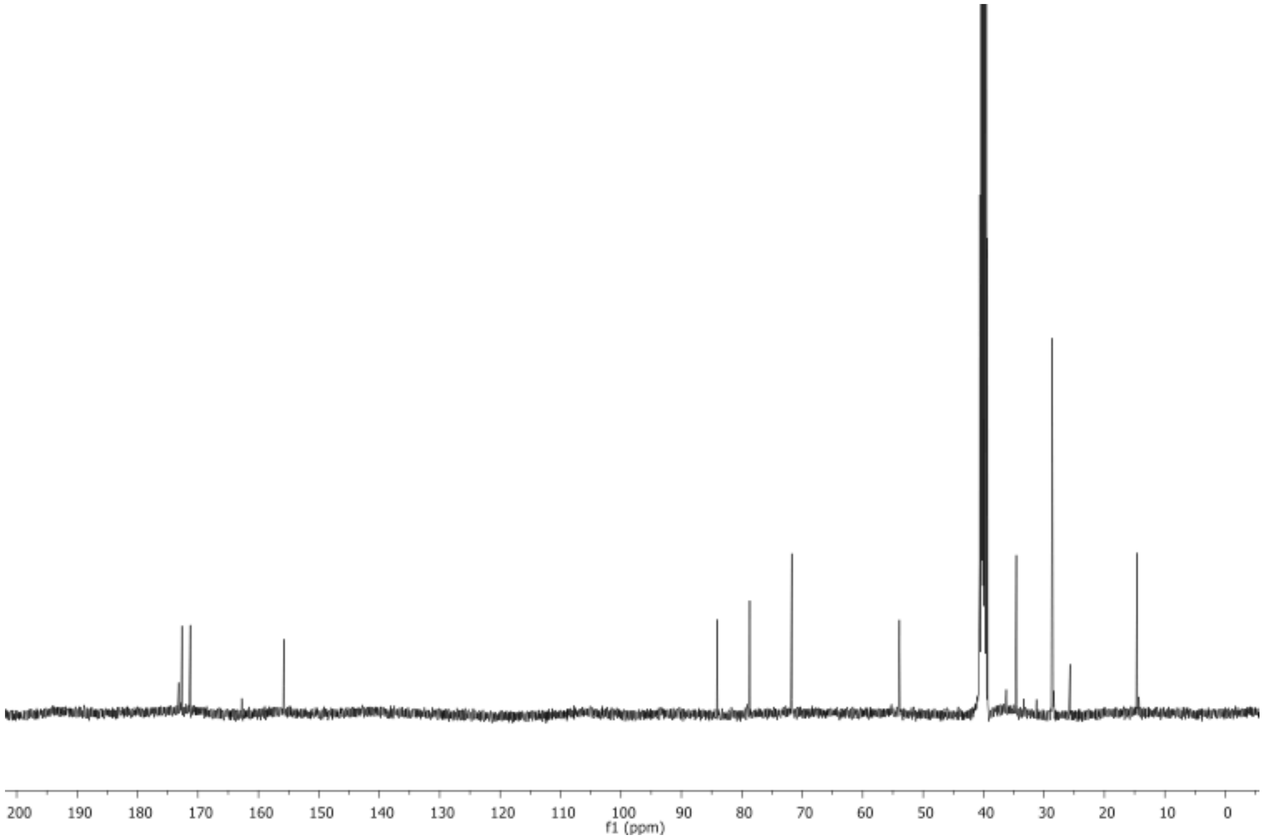

Building block 11

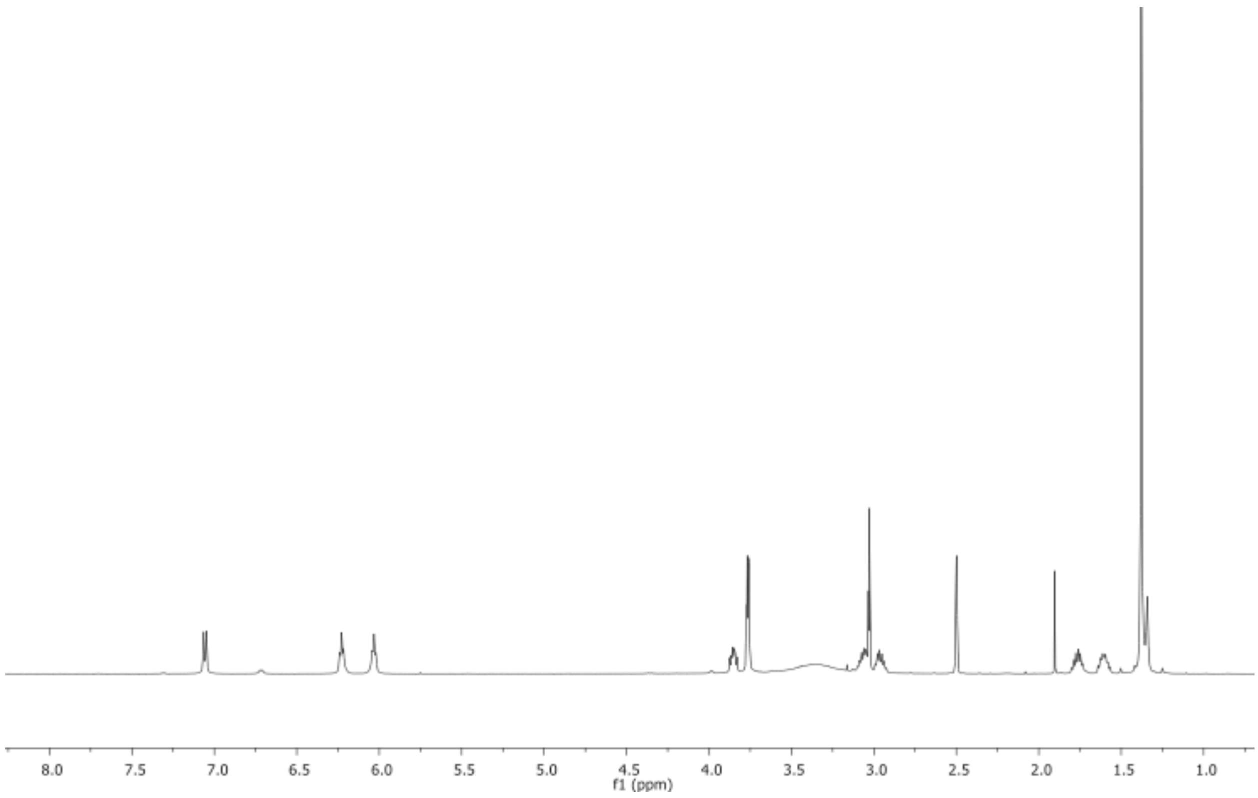

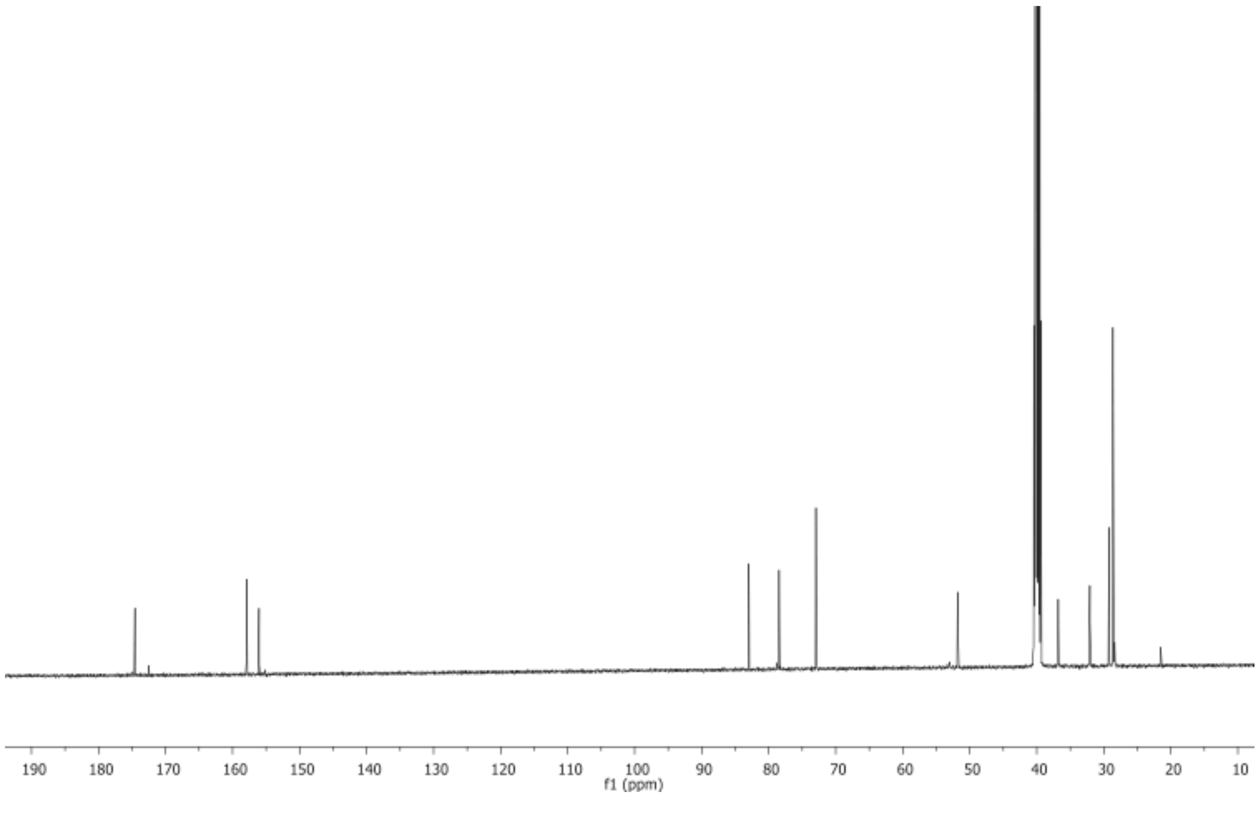

Building block 12

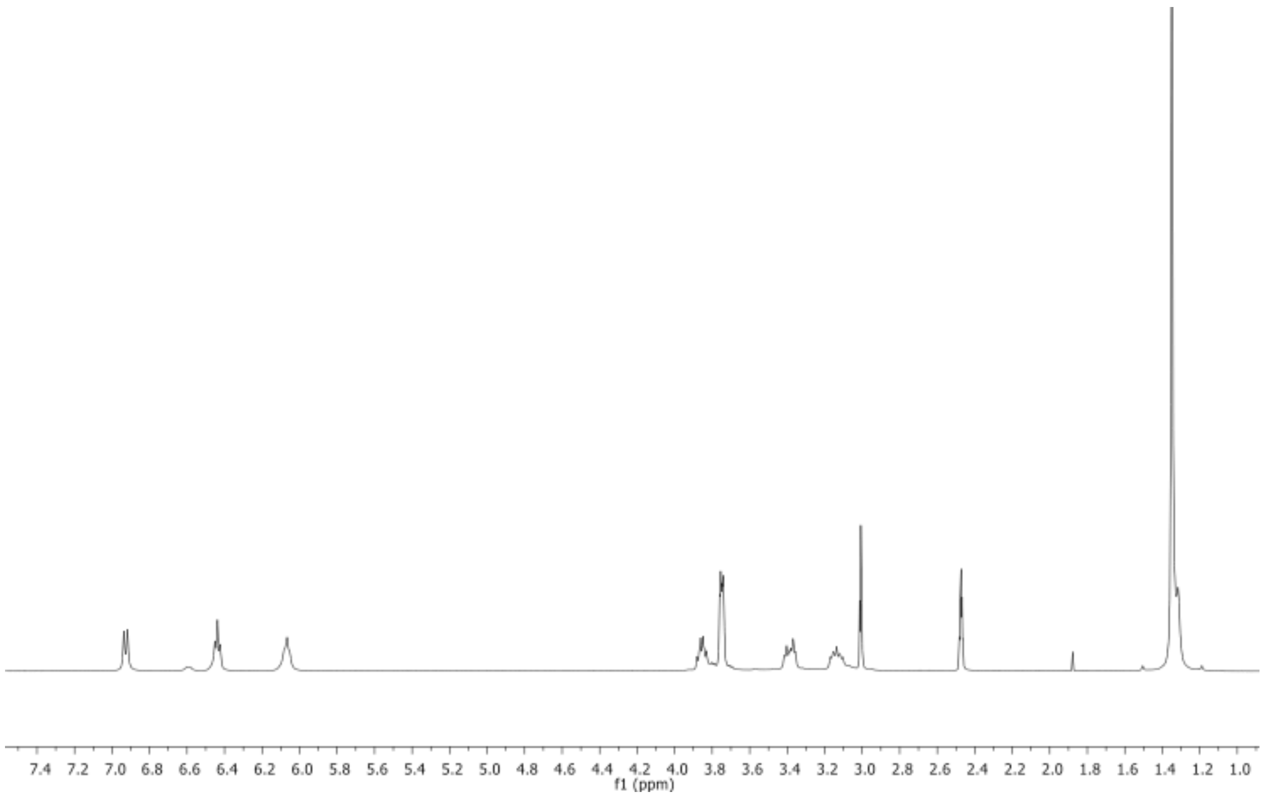

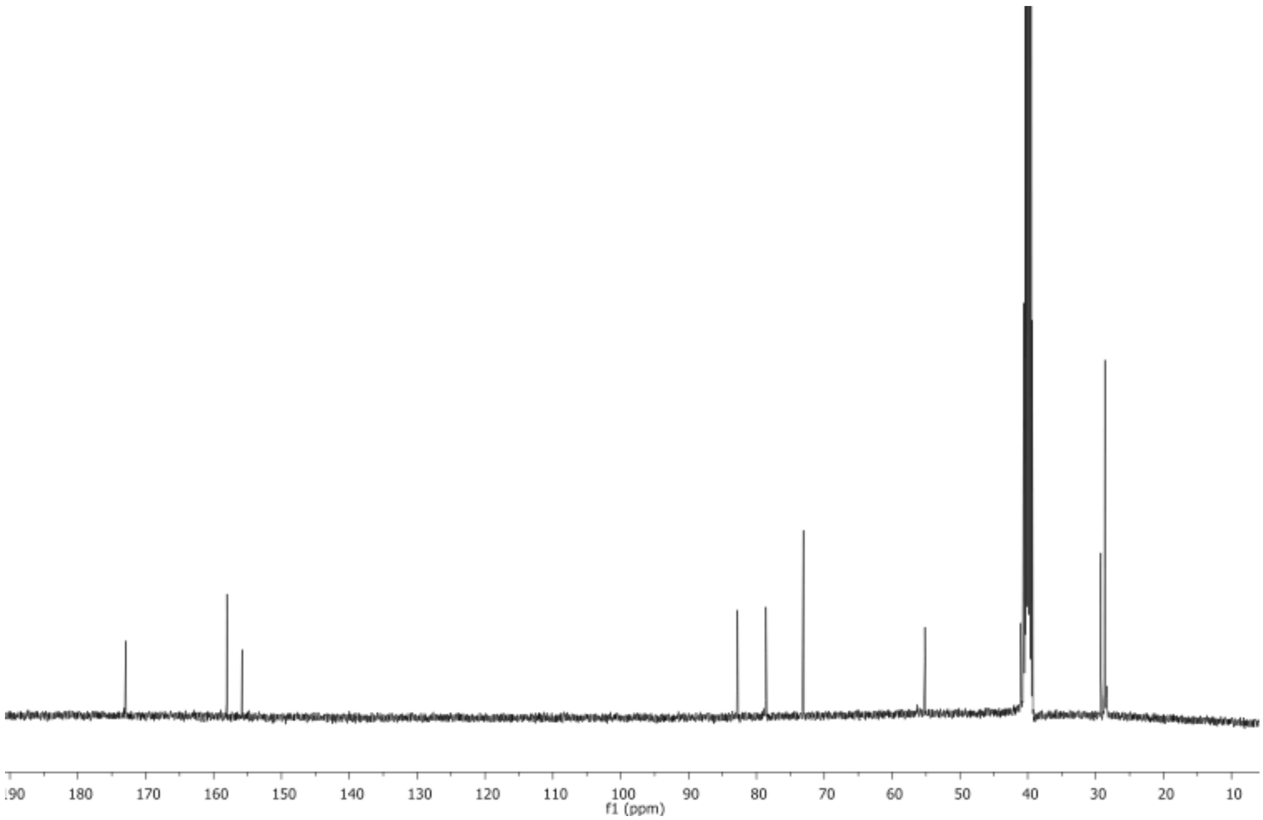

Building block 13

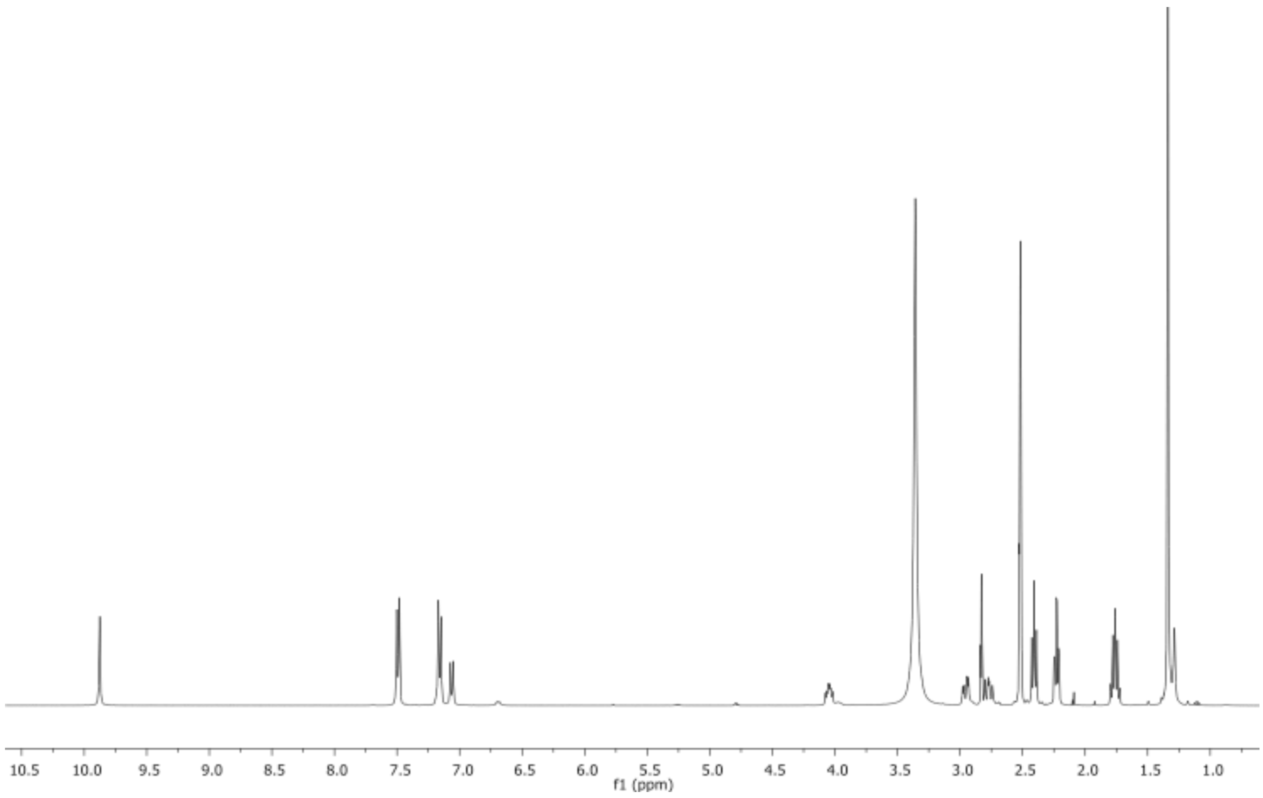

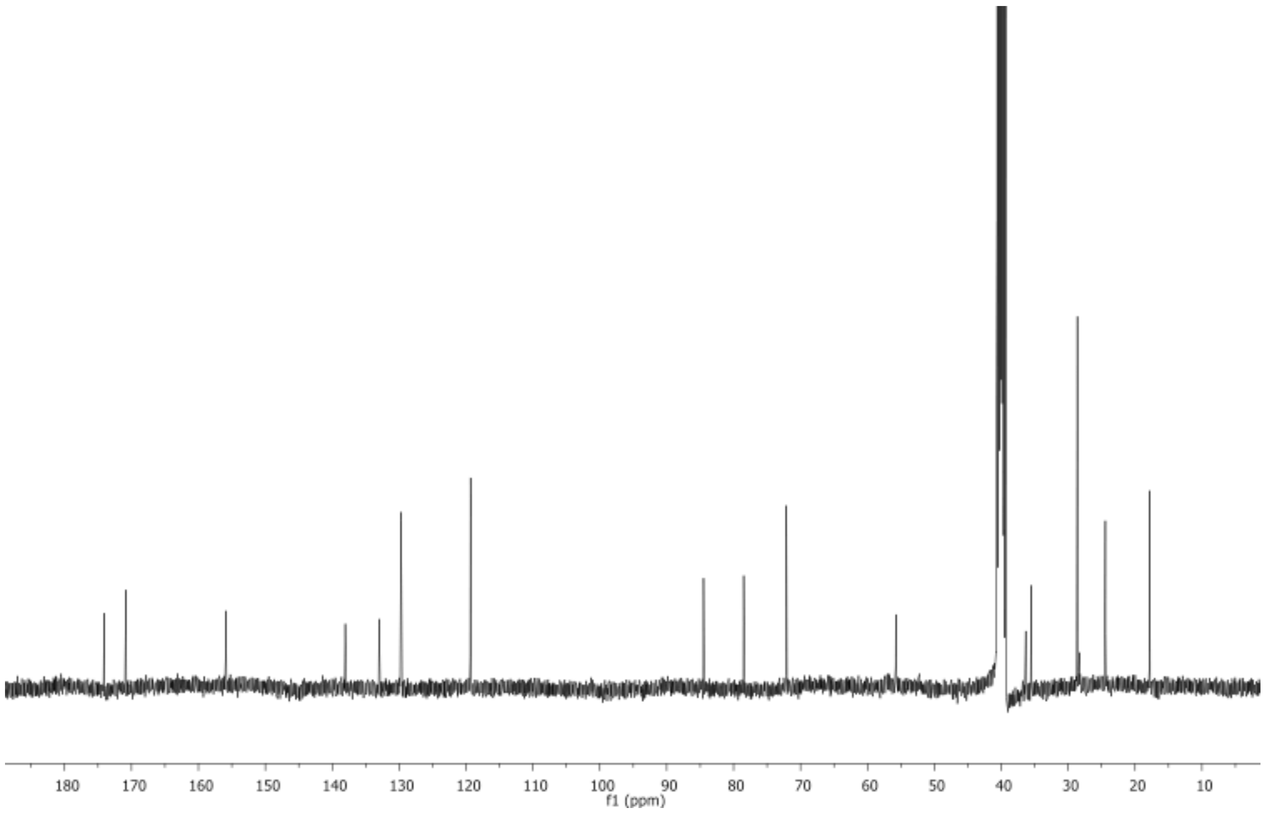

Building block G

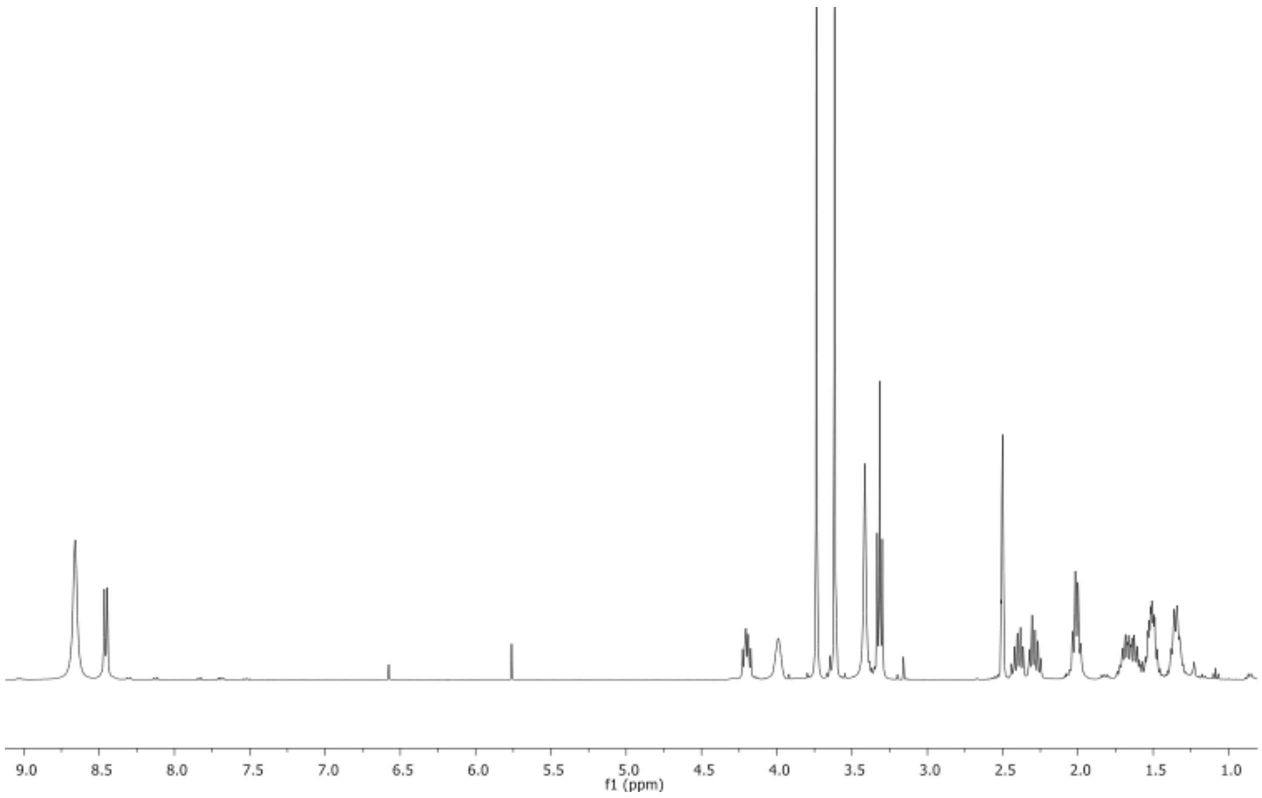

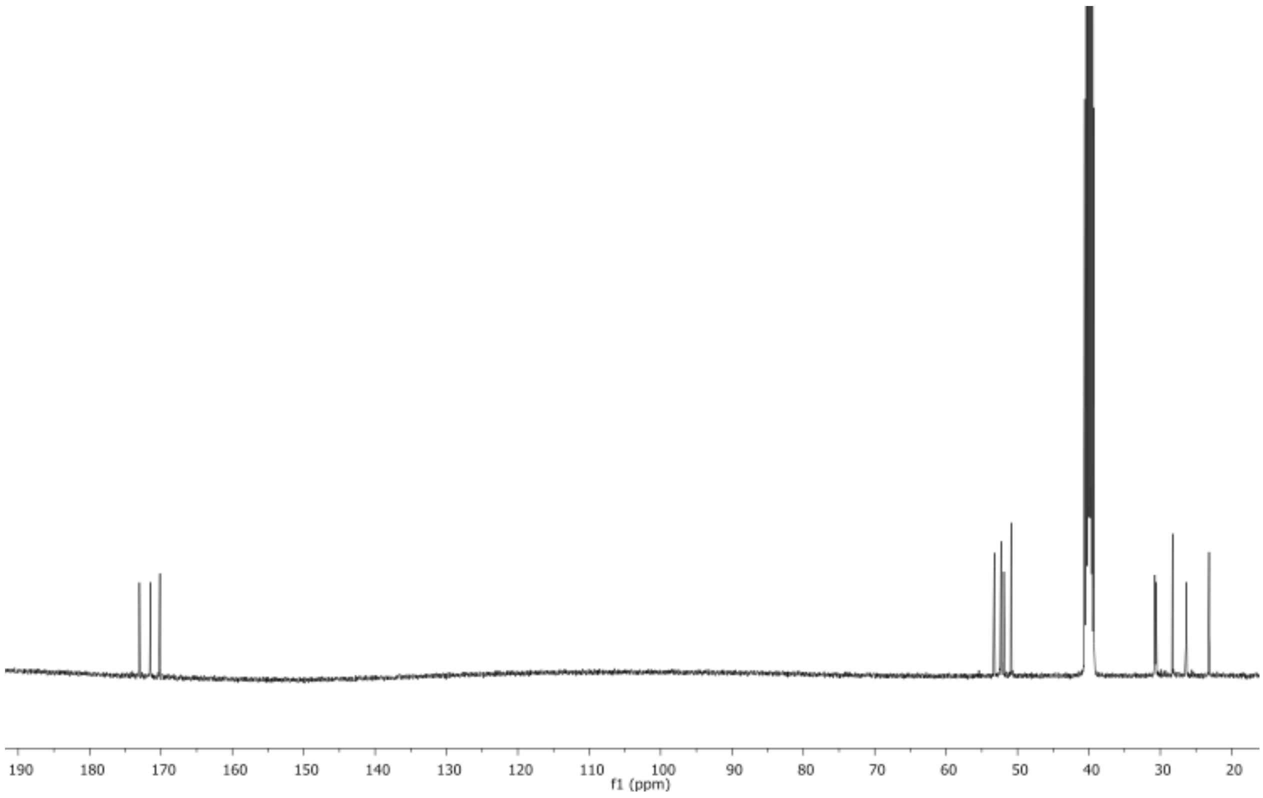

Building block H

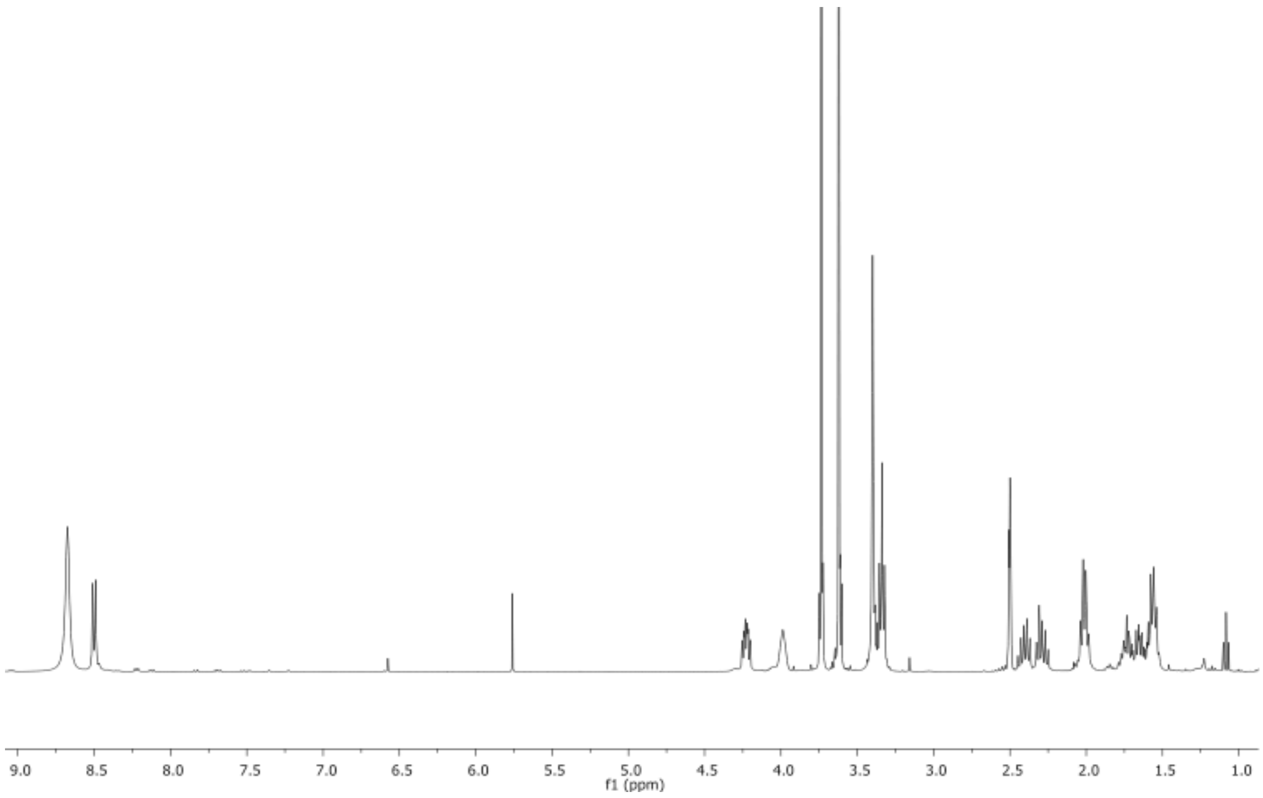

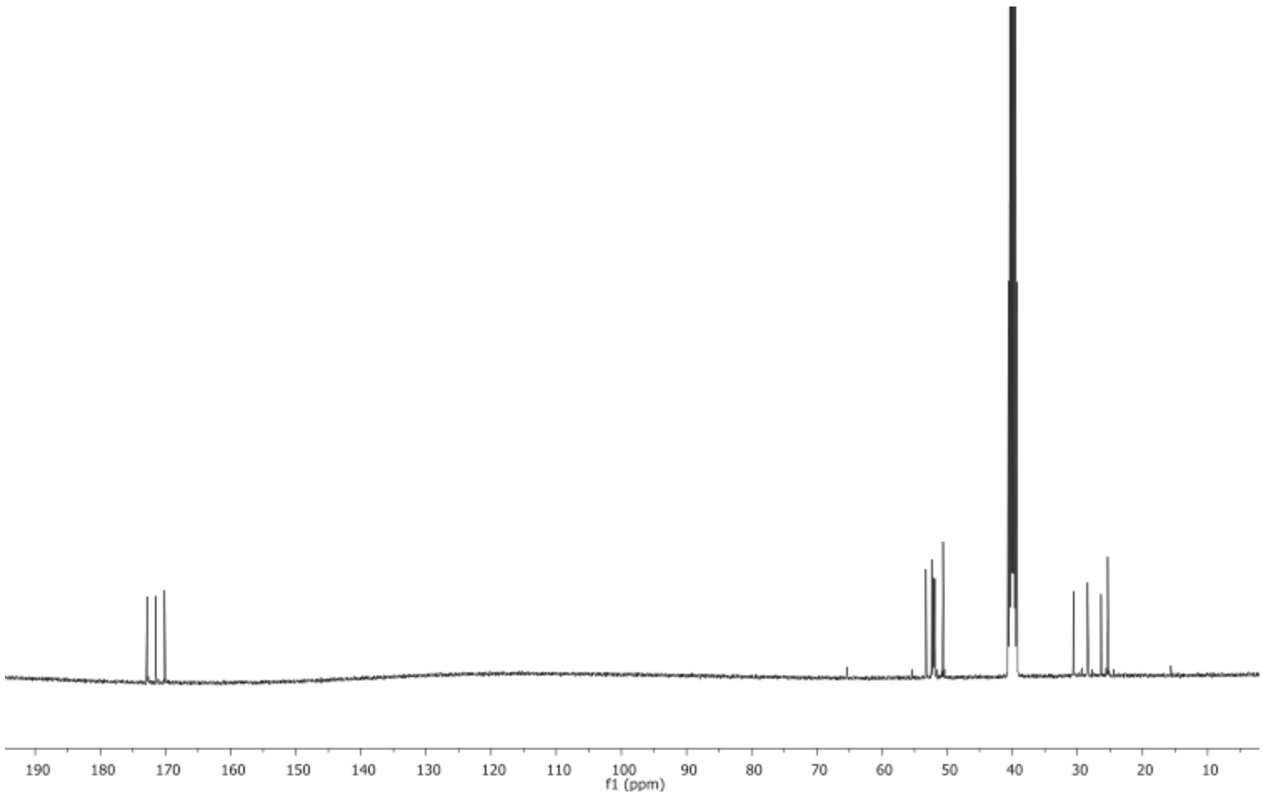

A12

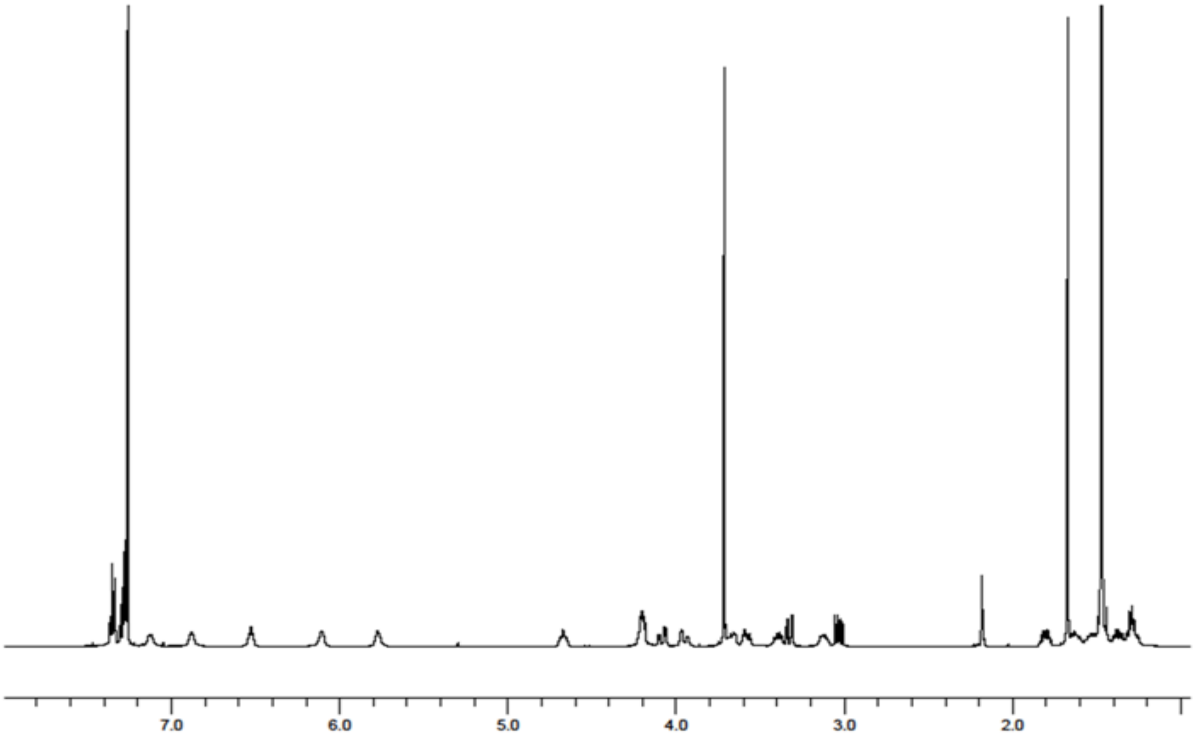

7.0
6.0
5.0
4.0
3.0
2.0

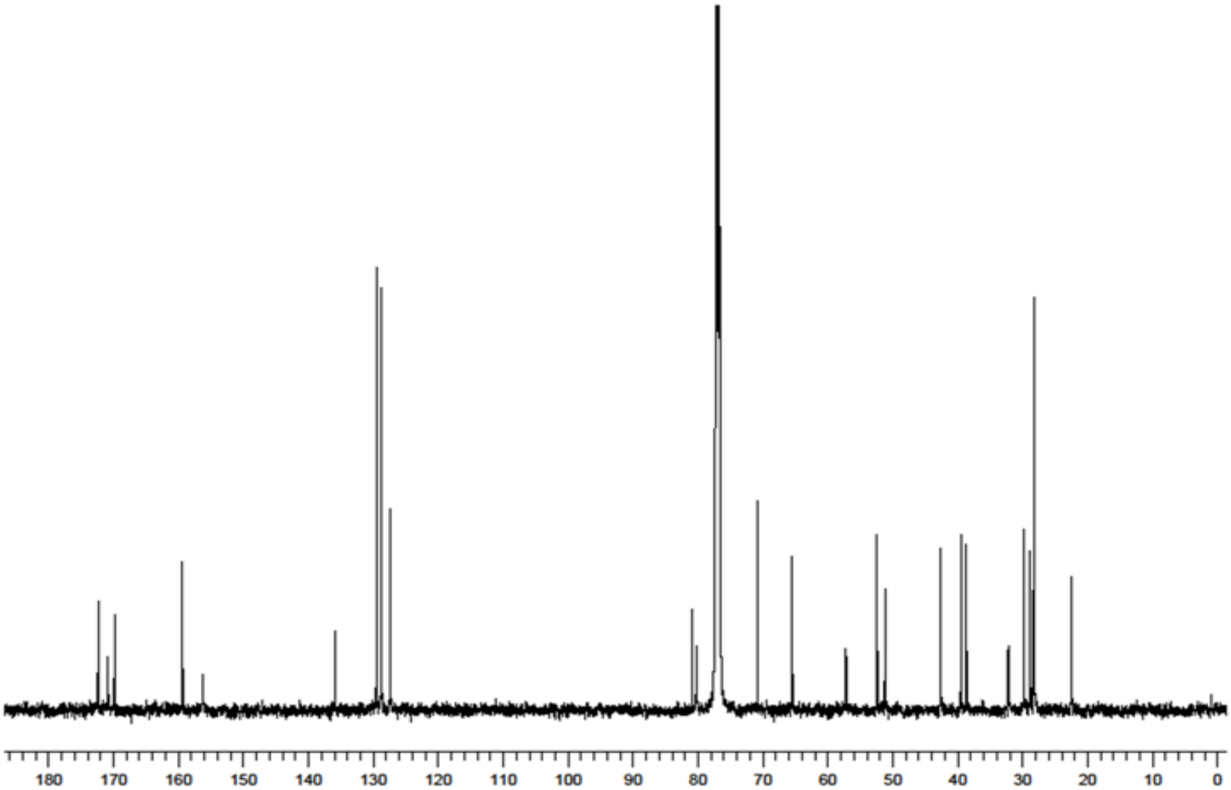

180
170
160
150
140
130
120
110
100
90
80
70
60
50
40
30
20
10
0

B2

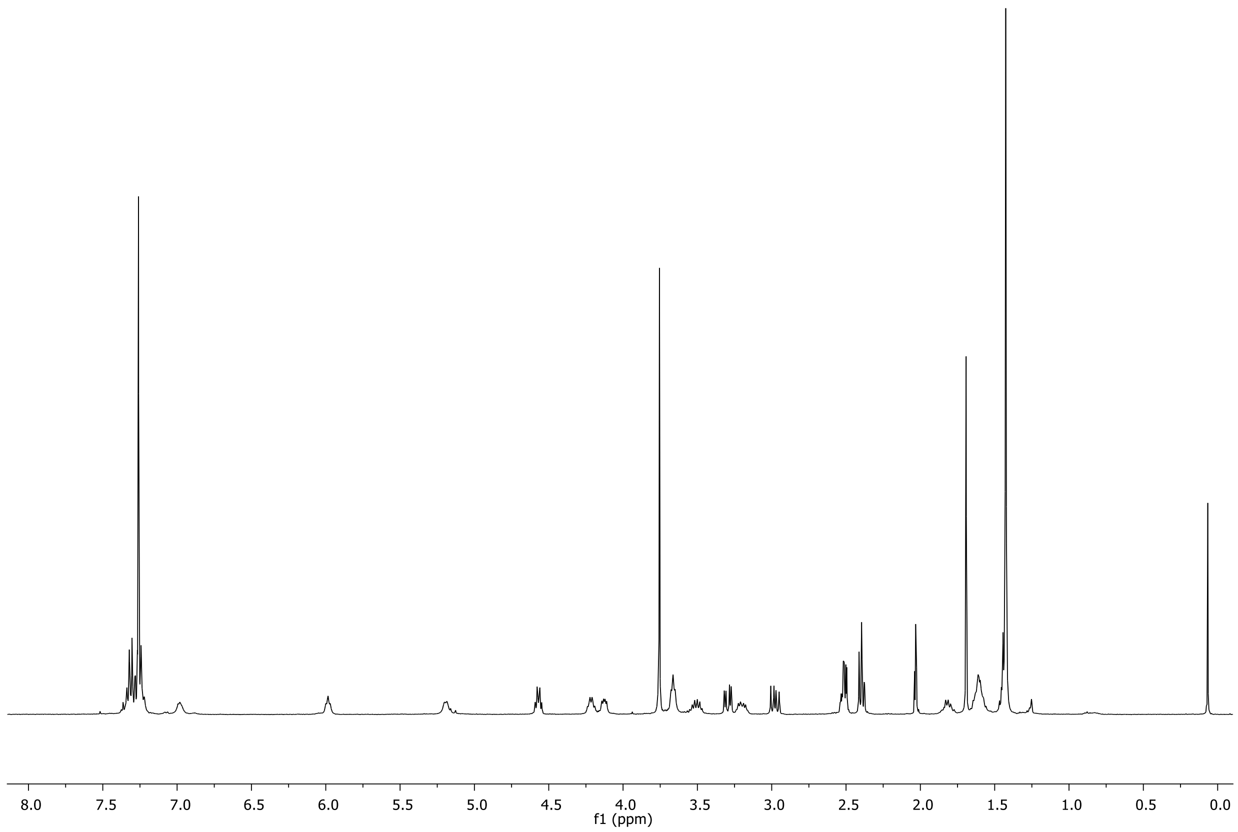
8.0
7.5
7.0
6.5
6.0
5.5
5.0
4.5
4.0
3.5
3.0
2.5
2.0
1.5
1.0
0.5
0.0
f1 (ppm)

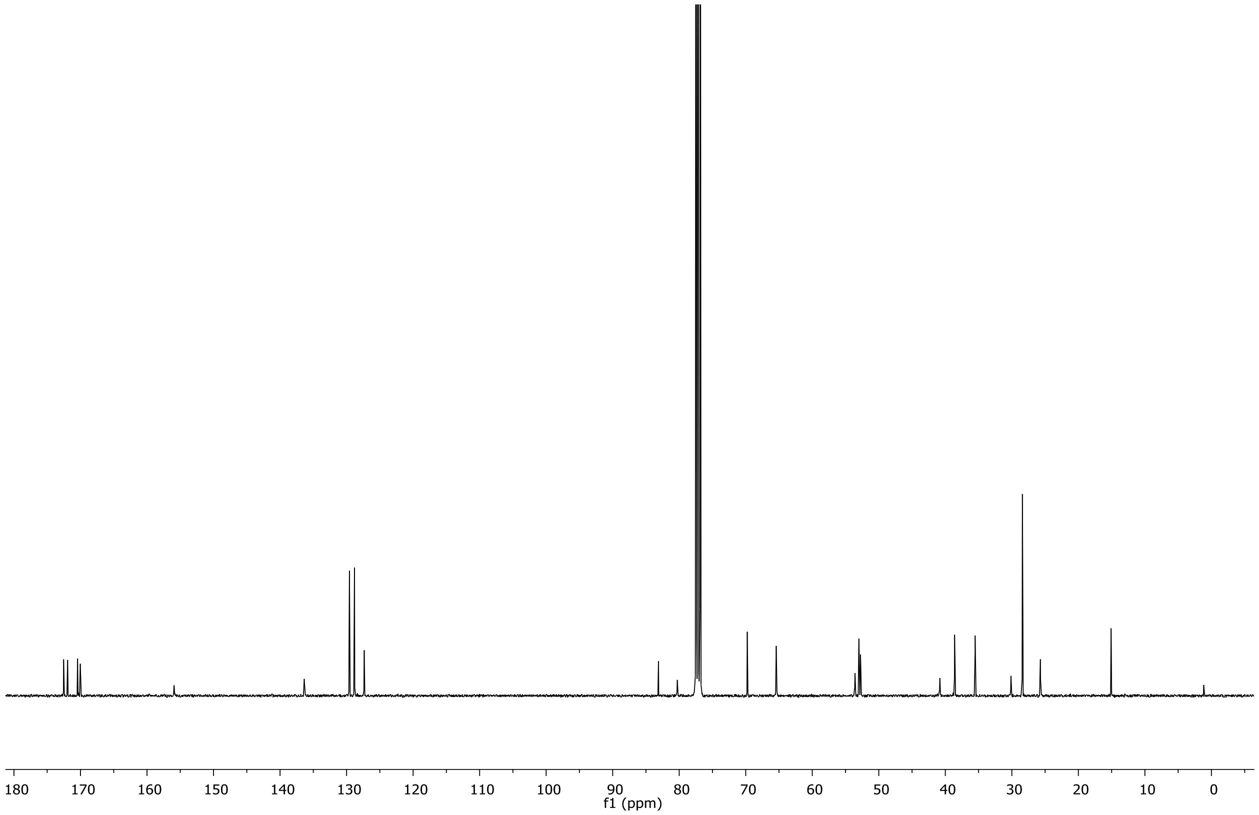
180
170
160
150
140
130
120
110
100
90
80
70
60
50
40
30
20
10
0
f1 (ppm)

B3

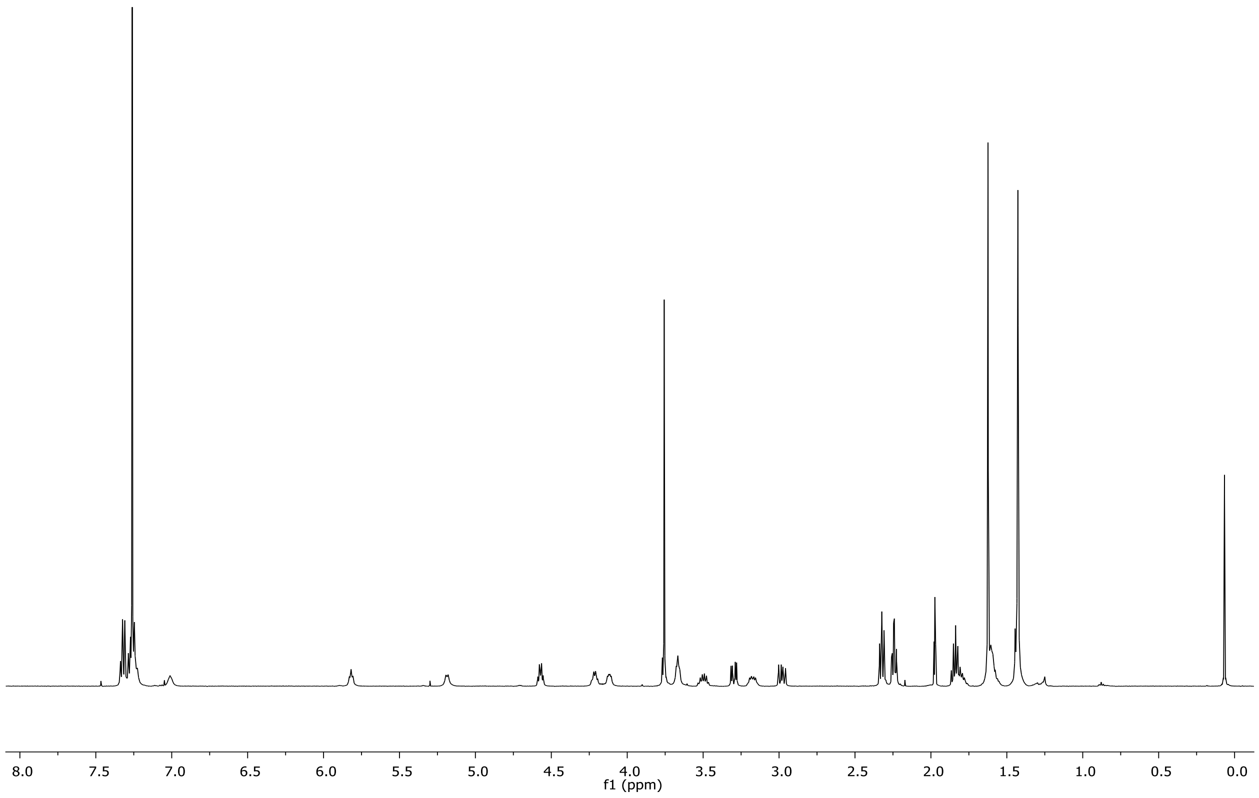
8.0 7.5 7.0 6.5 6.0 5.5 5.0 4.5 4.0 3.5 3.0 2.5 2.0 1.5 1.0 0.5 0.0
f1 (ppm)

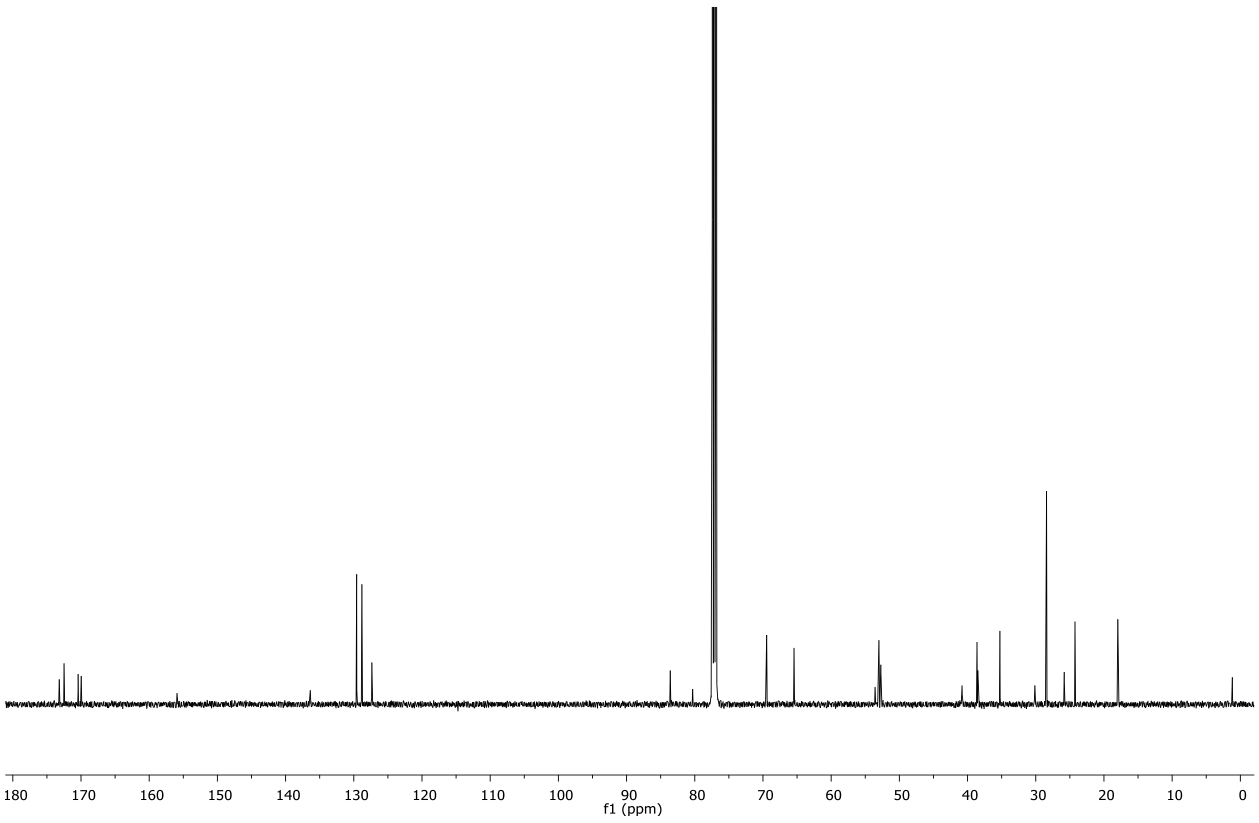
180 170 160 150 140 130 120 110 100 90 80 70 60 50 40 30 20 10 0
f1 (ppm)

B5

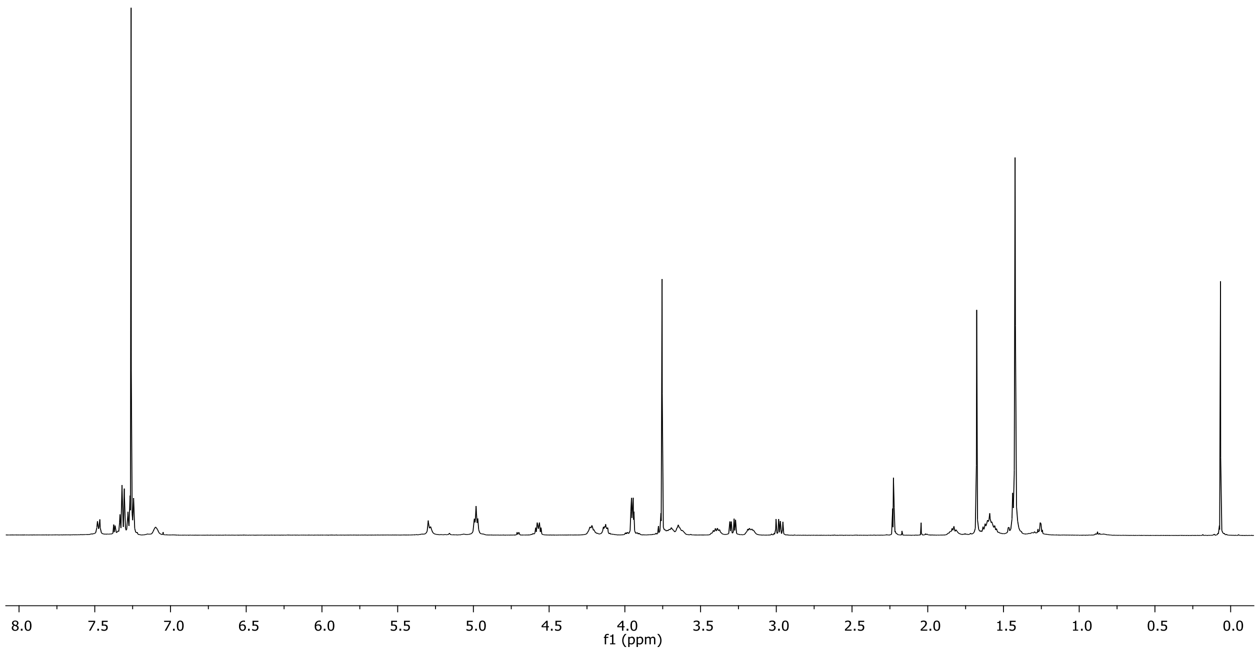
8.0
7.5
7.0
6.5
6.0
5.5
5.0
4.5
4.0
3.5
3.0
2.5
2.0
1.5
1.0
0.5
0.0
f1 (ppm)

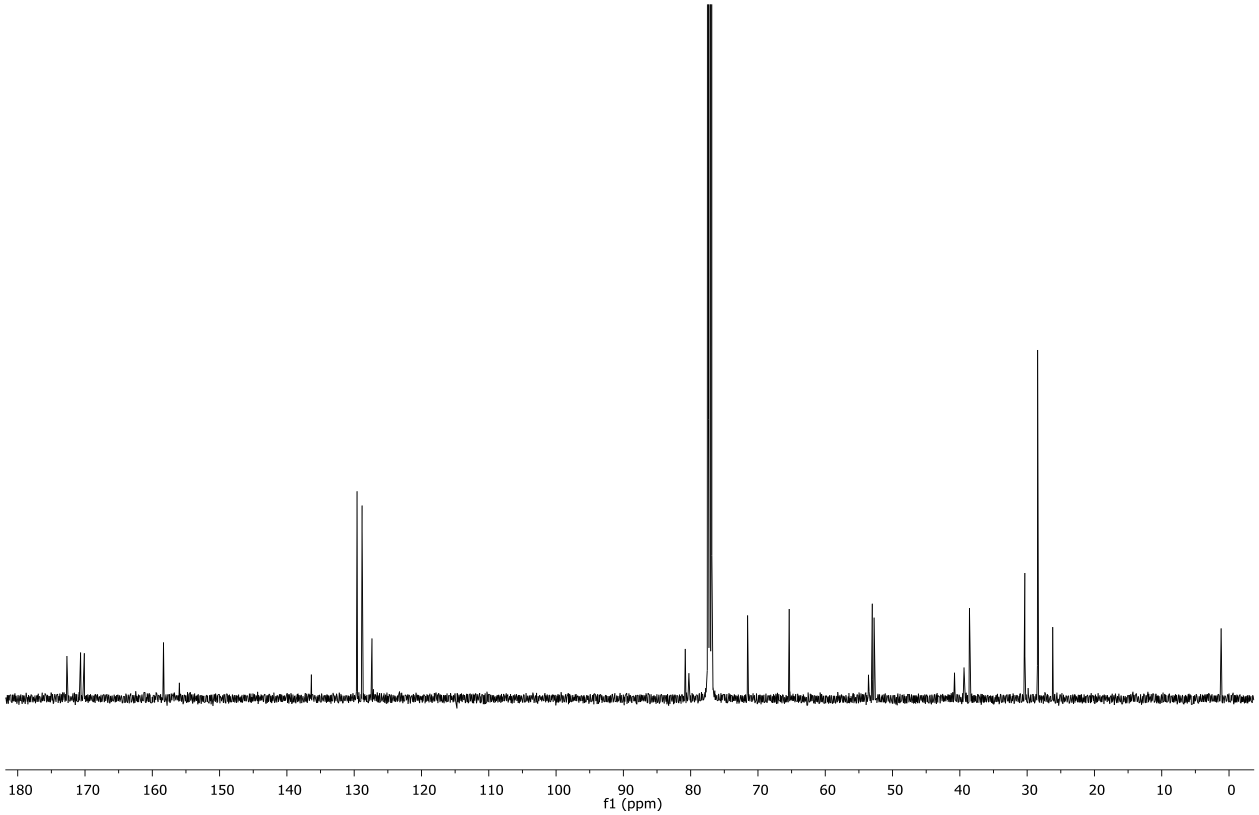
180
170
160
150
140
130
120
110
100
90
80
70
60
50
40
30
20
10
0
f1 (ppm)

B7

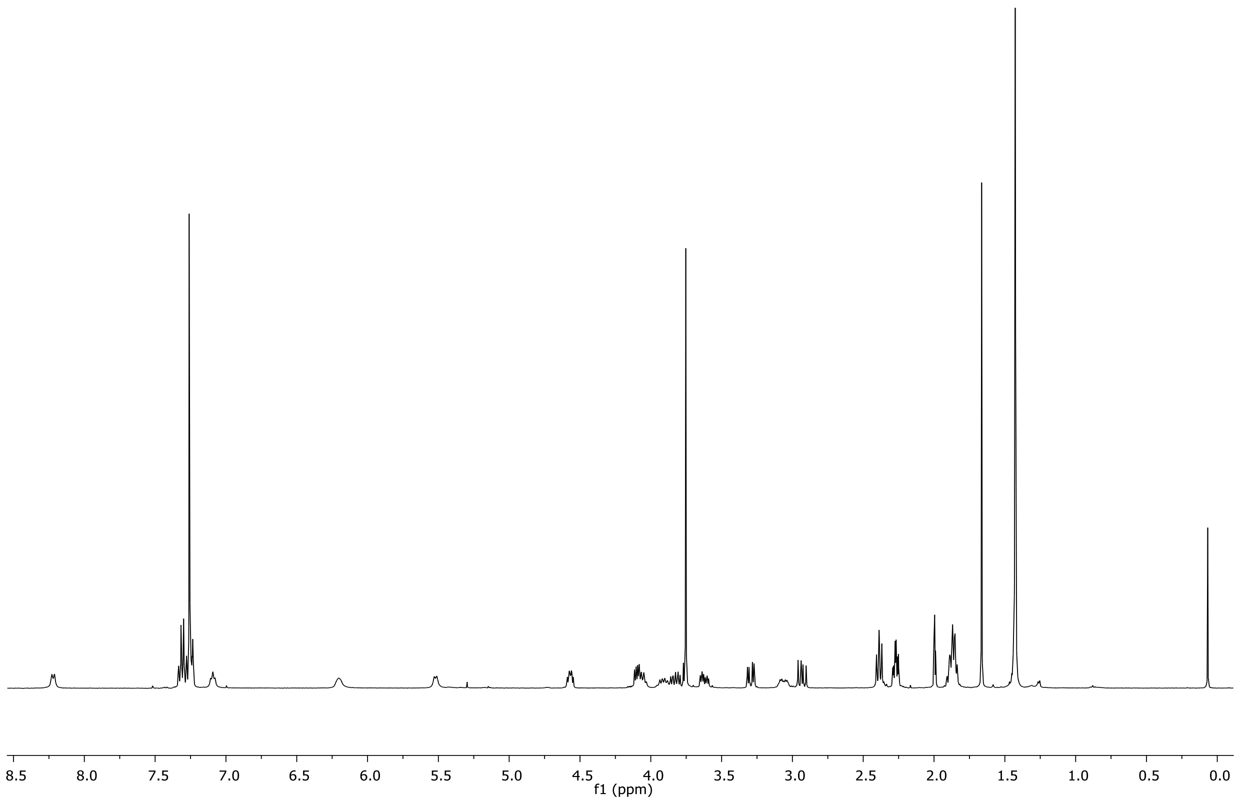

8.5
8.0
7.5
7.0
6.5
6.0
5.5
5.0
4.5
4.0
3.5
3.0
2.5
2.0
1.5
1.0
0.5
0.0
f1 (ppm)

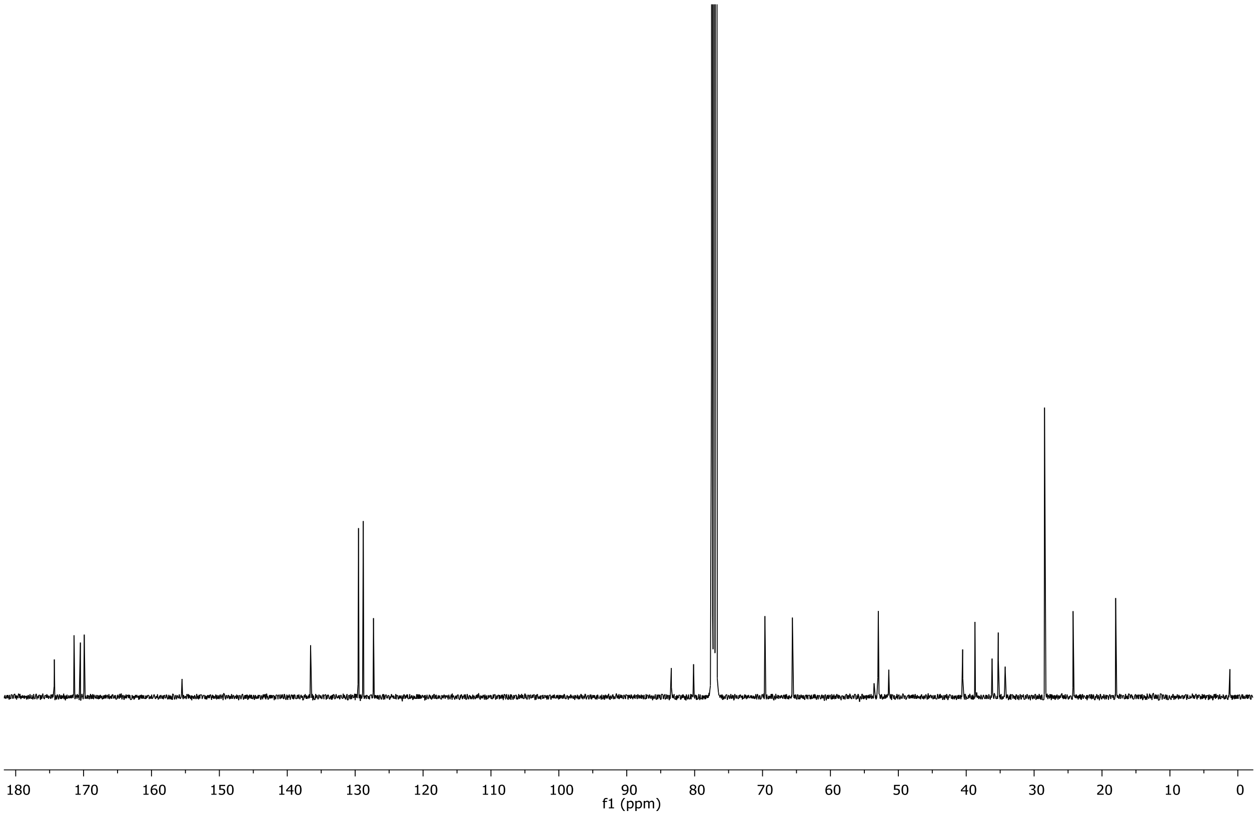

180
170
160
150
140
130
120
110
100
90
80
70
60
50
40
30
20
10
0
f1 (ppm)

B8

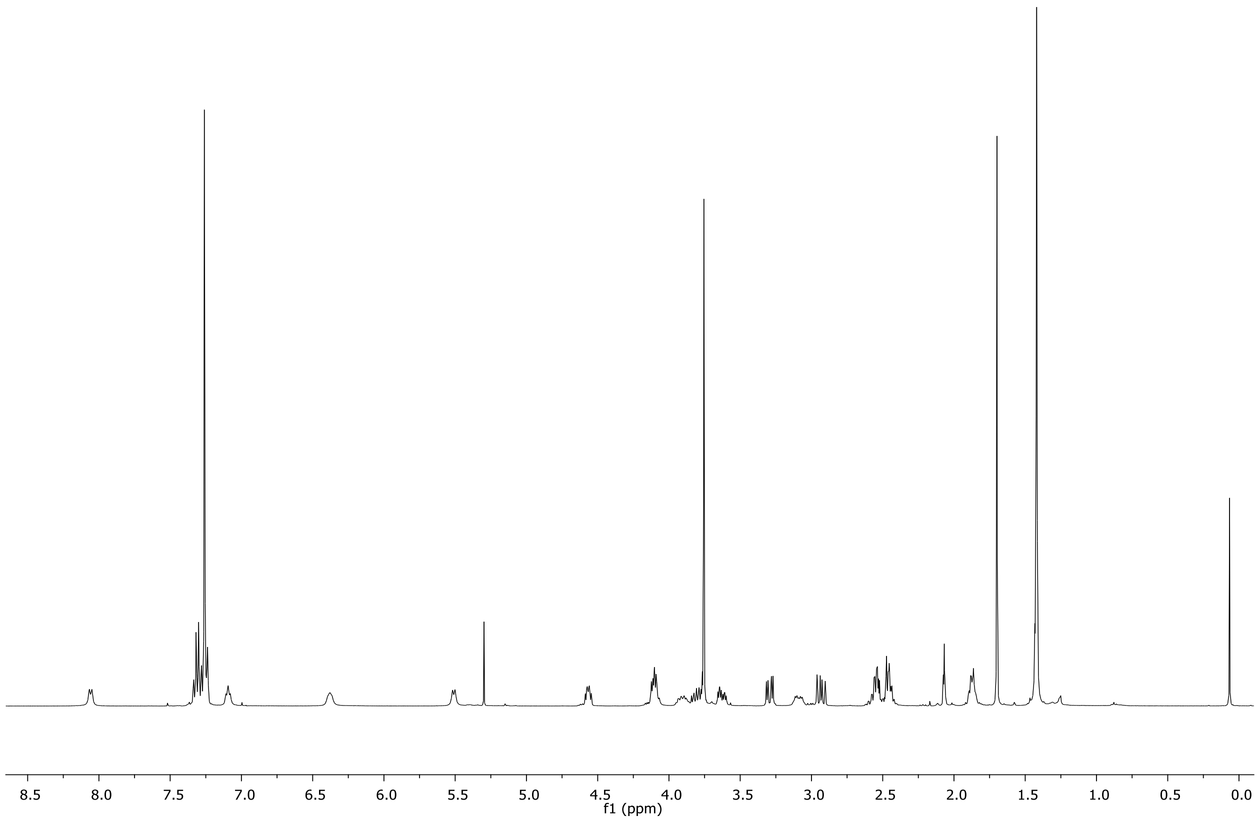

8.5
8.0
7.5
7.0
6.5
6.0
5.5
5.0
4.5
4.0
3.5
3.0
2.5
2.0
1.5
1.0
0.5
0.0
f1 (ppm)

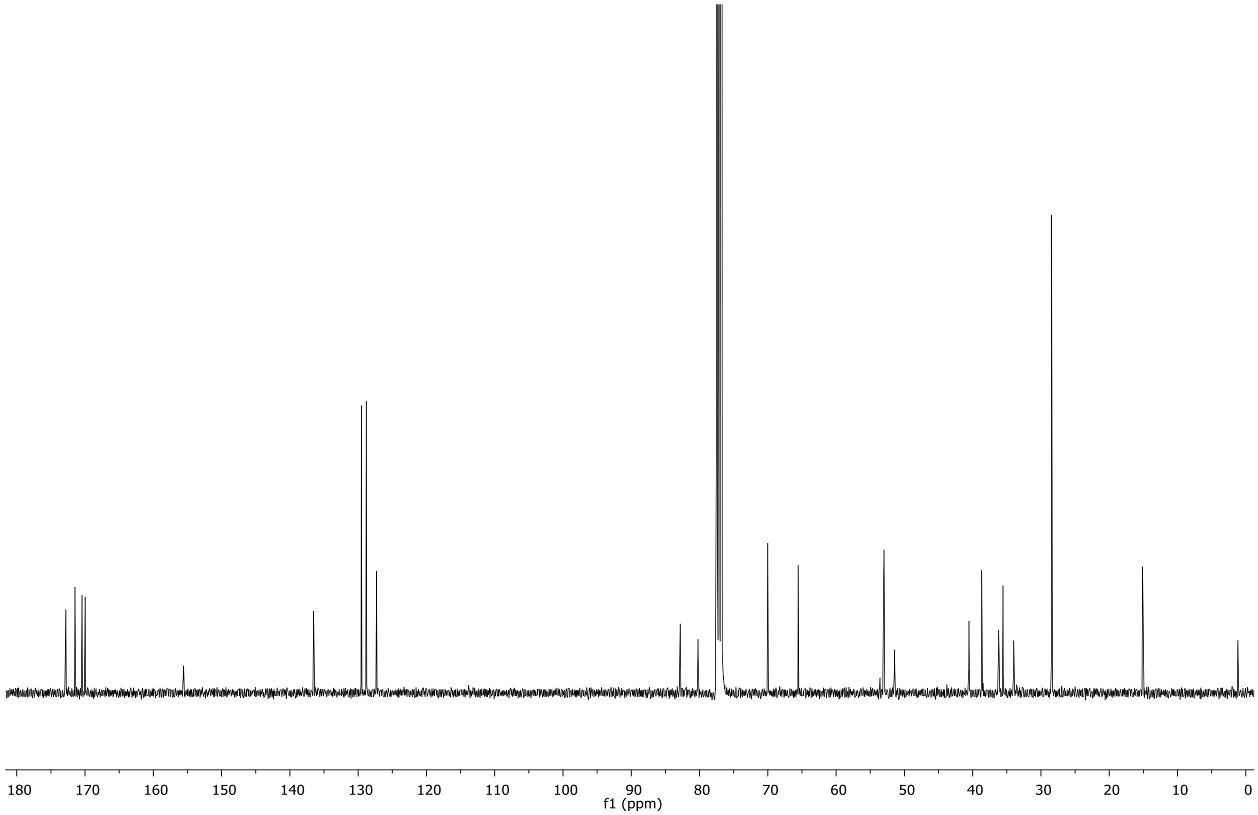

180
170
160
150
140
130
120
110
100
90
80
70
60
50
40
30
20
10
0
f1 (ppm)

B9

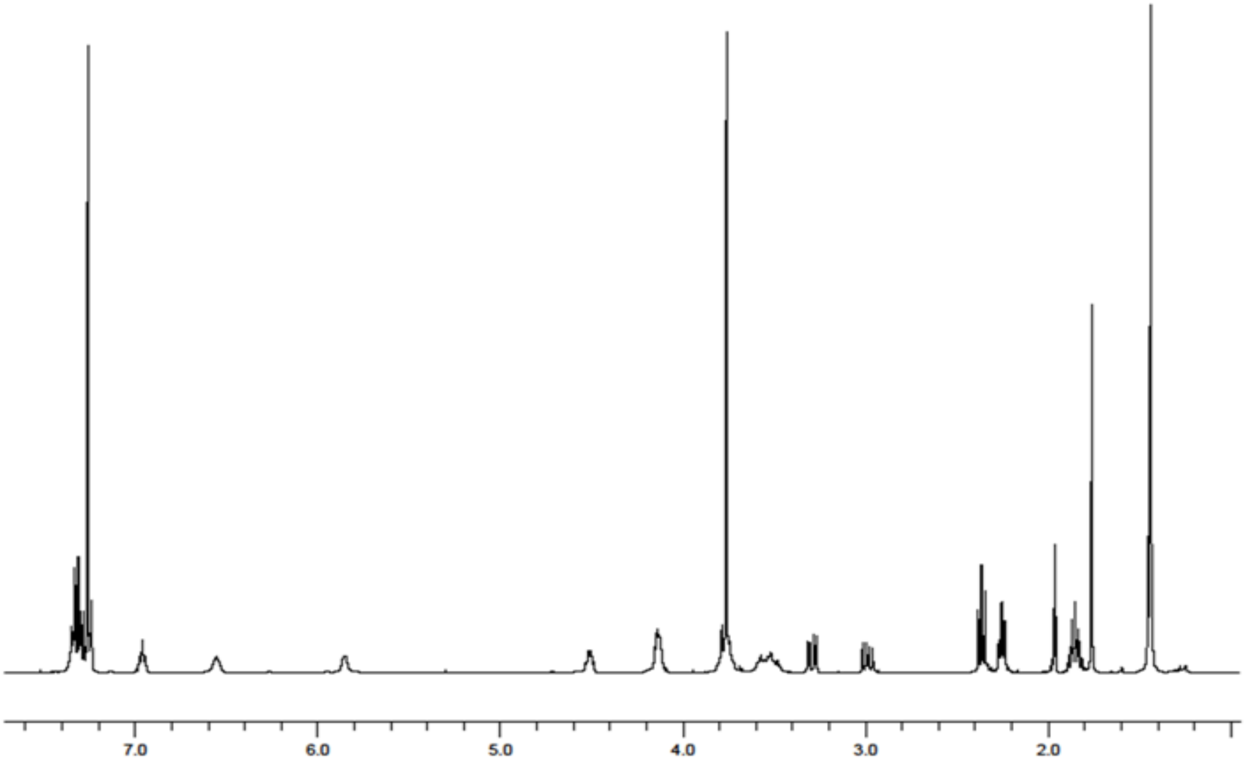
7.0
6.0
5.0
4.0
3.0
2.0

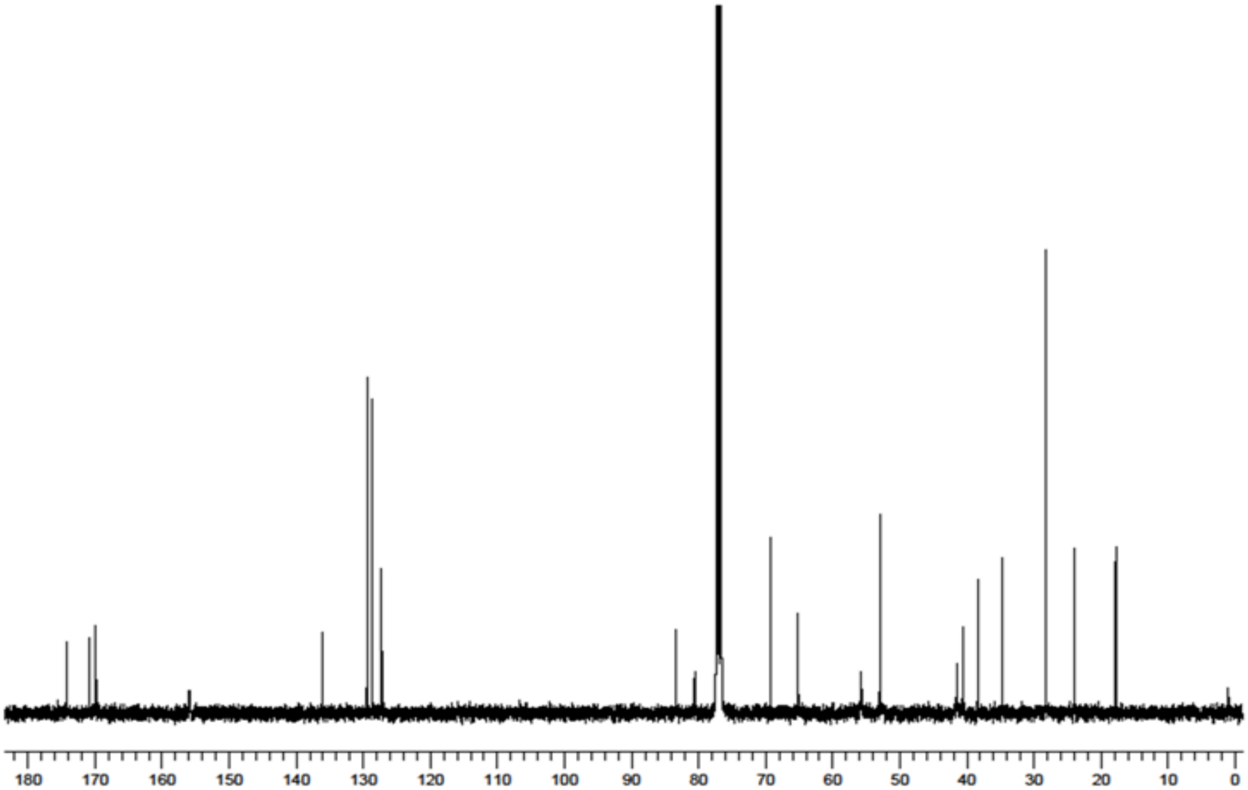
180
170
160
150
140
130
120
110
100
90
80
70
60
50
40
30
20
10
0

B10

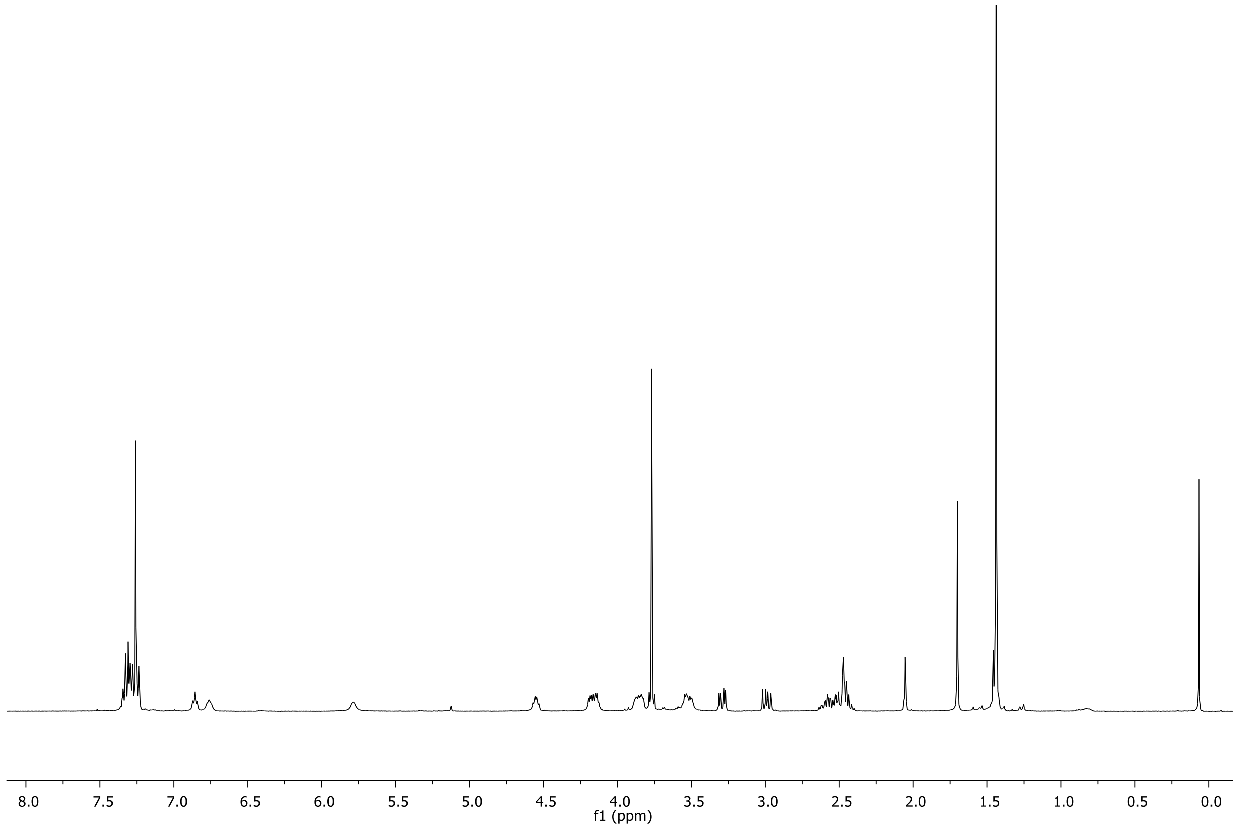
8.0
7.5
7.0
6.5
6.0
5.5
5.0
4.5
4.0
3.5
3.0
2.5
2.0
1.5
1.0
0.5
0.0
f1 (ppm)

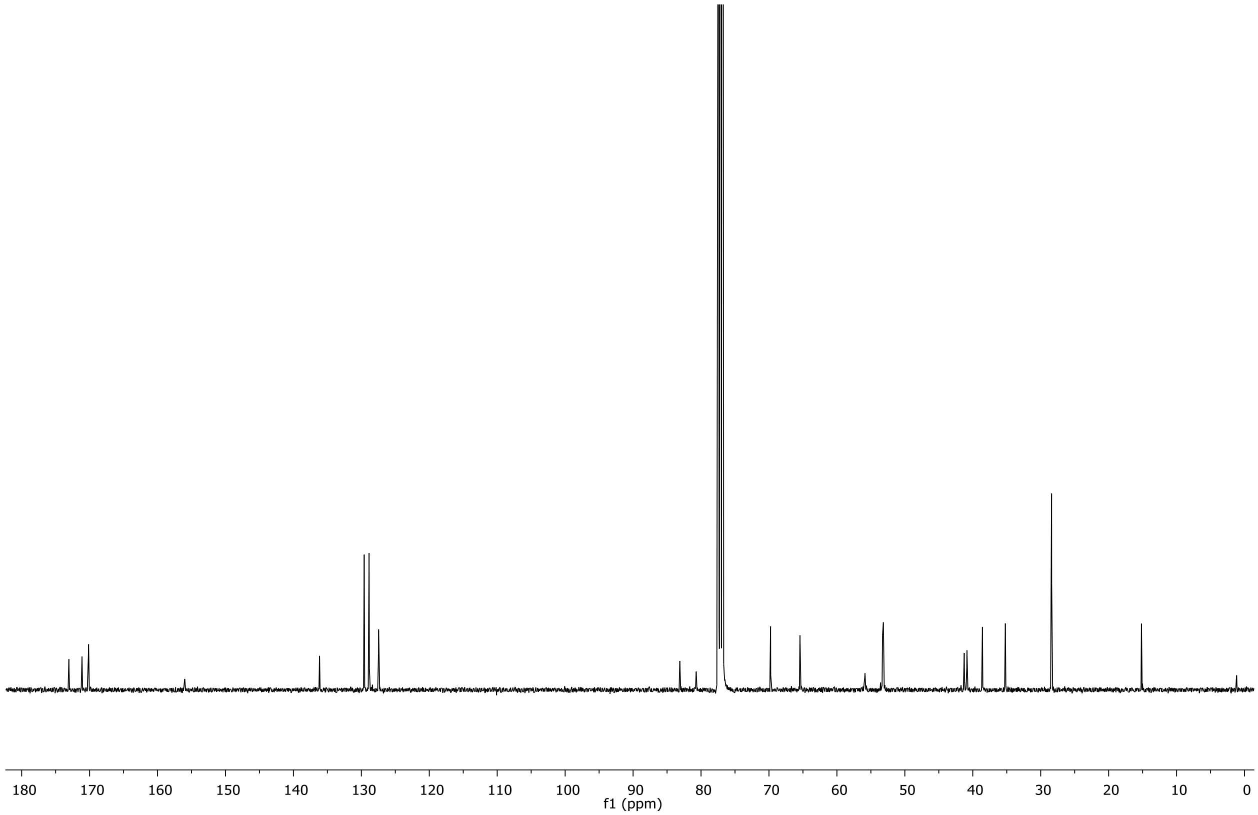
180
170
160
150
140
130
120
110
100
90
80
70
60
50
40
30
20
10
0
f1 (ppm)

B11

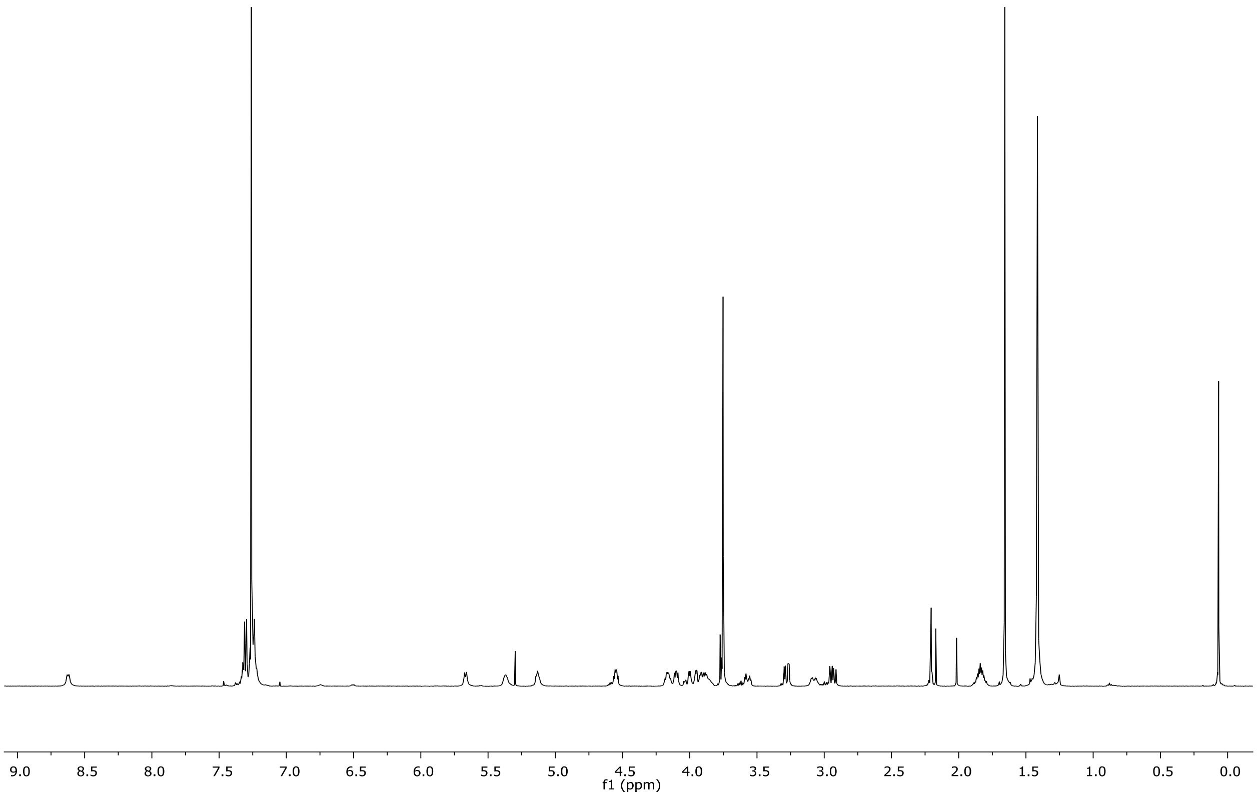
9.0 8.5 8.0 7.5 7.0 6.5 6.0 5.5 5.0 4.5 4.0 3.5 3.0 2.5 2.0 1.5 1.0 0.5 0.0
f1 (ppm)

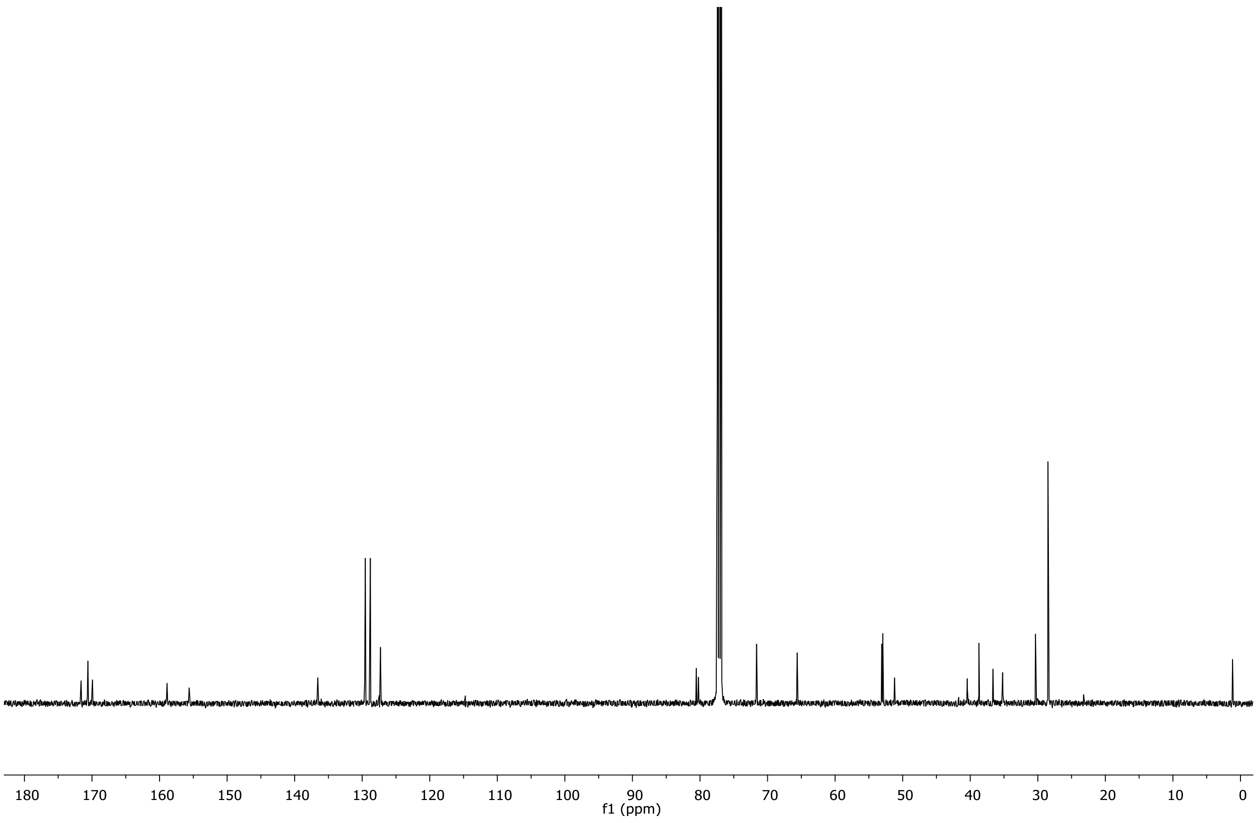
180 170 160 150 140 130 120 110 100 90 80 70 60 50 40 30 20 10 0
f1 (ppm)

B12

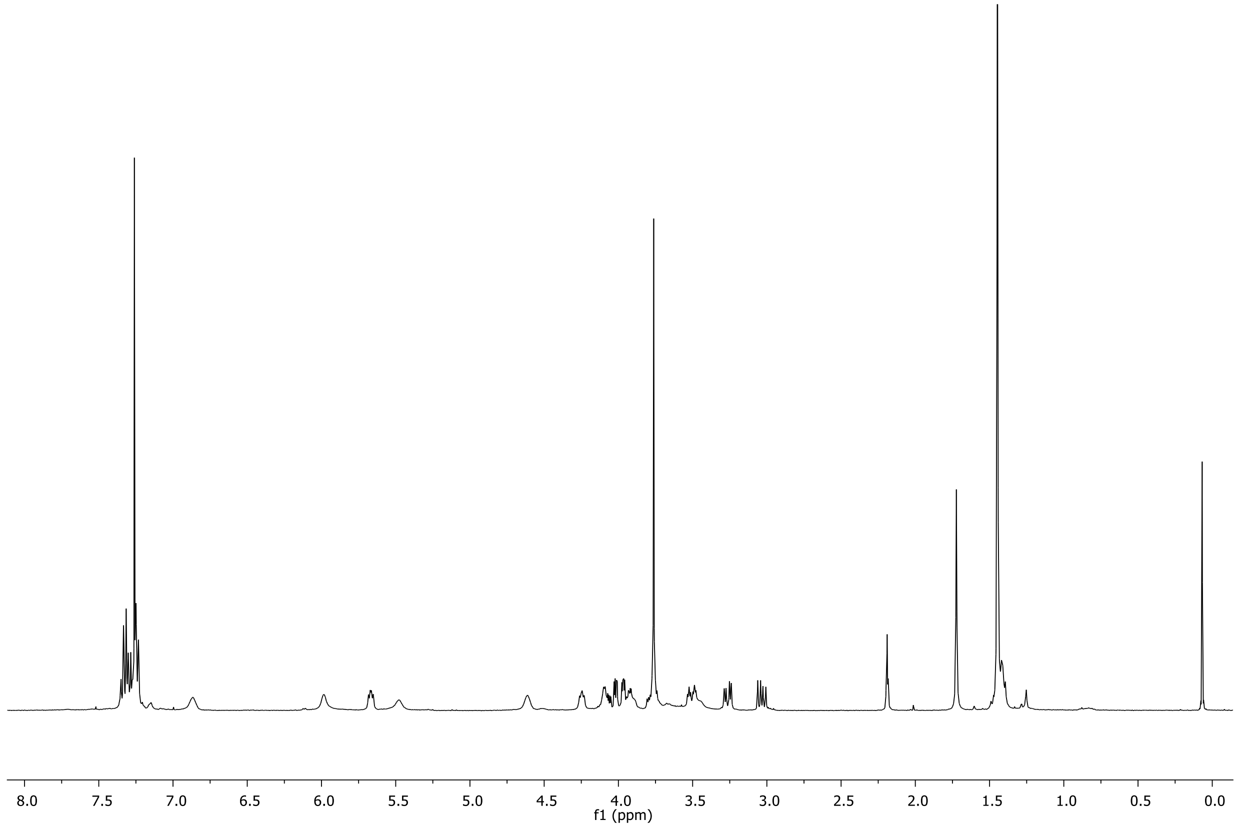
8.0
7.5
7.0
6.5
6.0
5.5
5.0
4.5
4.0
3.5
3.0
2.5
2.0
1.5
1.0
0.5
0.0
f1 (ppm)

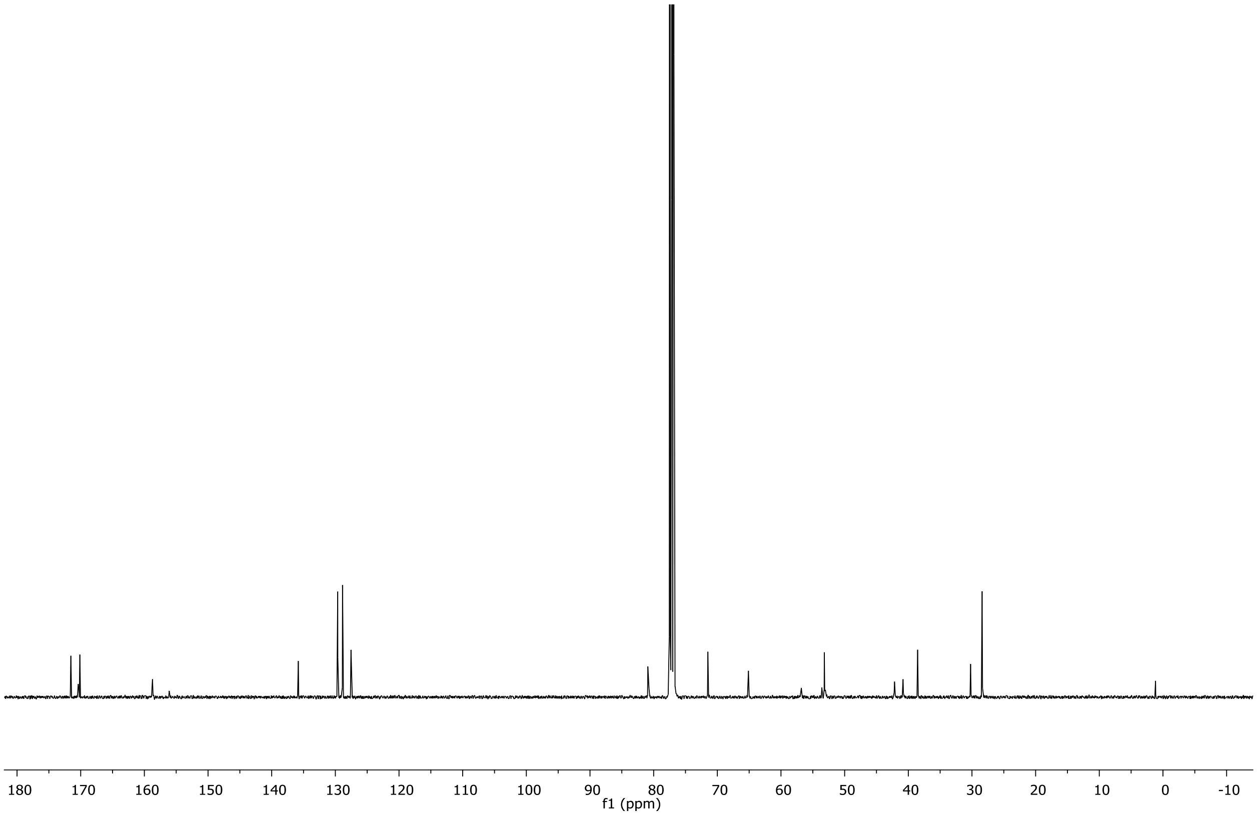
180
170
160
150
140
130
120
110
100
90
80
70
60
50
40
30
20
10
0
-10
f1 (ppm)

B14

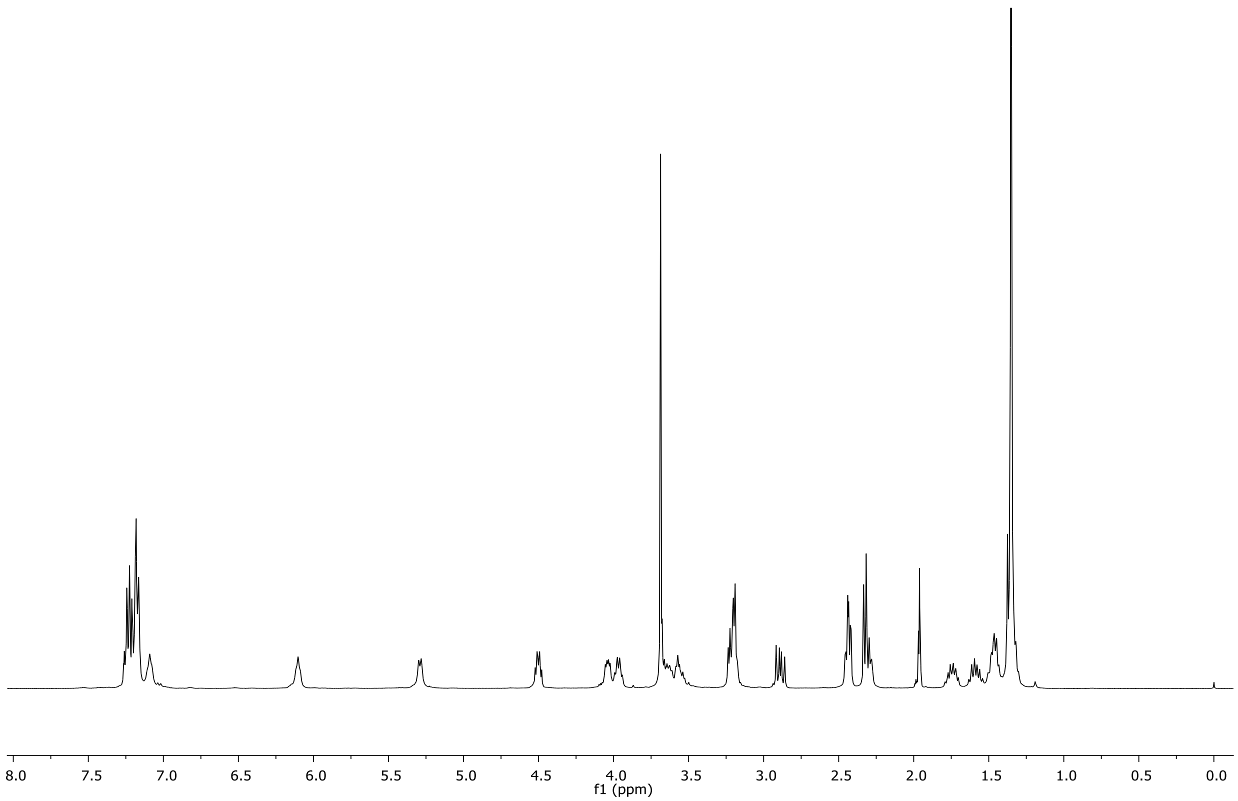
8.0 7.5 7.0 6.5 6.0 5.5 5.0 4.5 4.0 3.5 3.0 2.5 2.0 1.5 1.0 0.5 0.0
f1 (ppm)

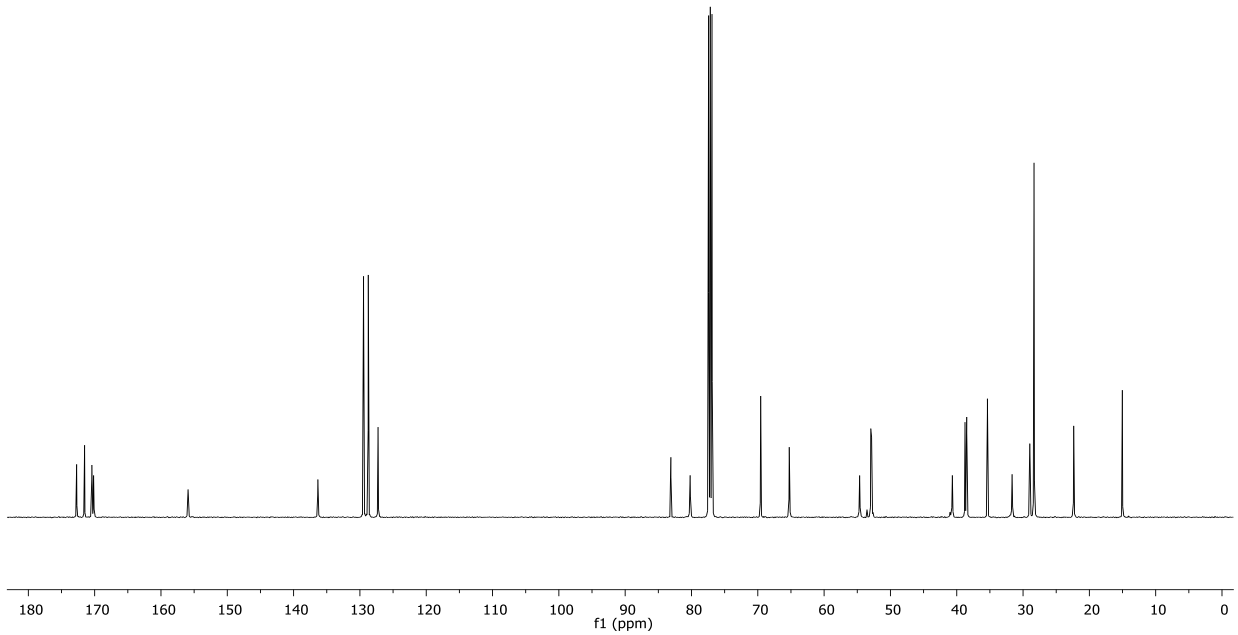
180 170 160 150 140 130 120 110 100 90 80 70 60 50 40 30 20 10 0
f1 (ppm)

C2

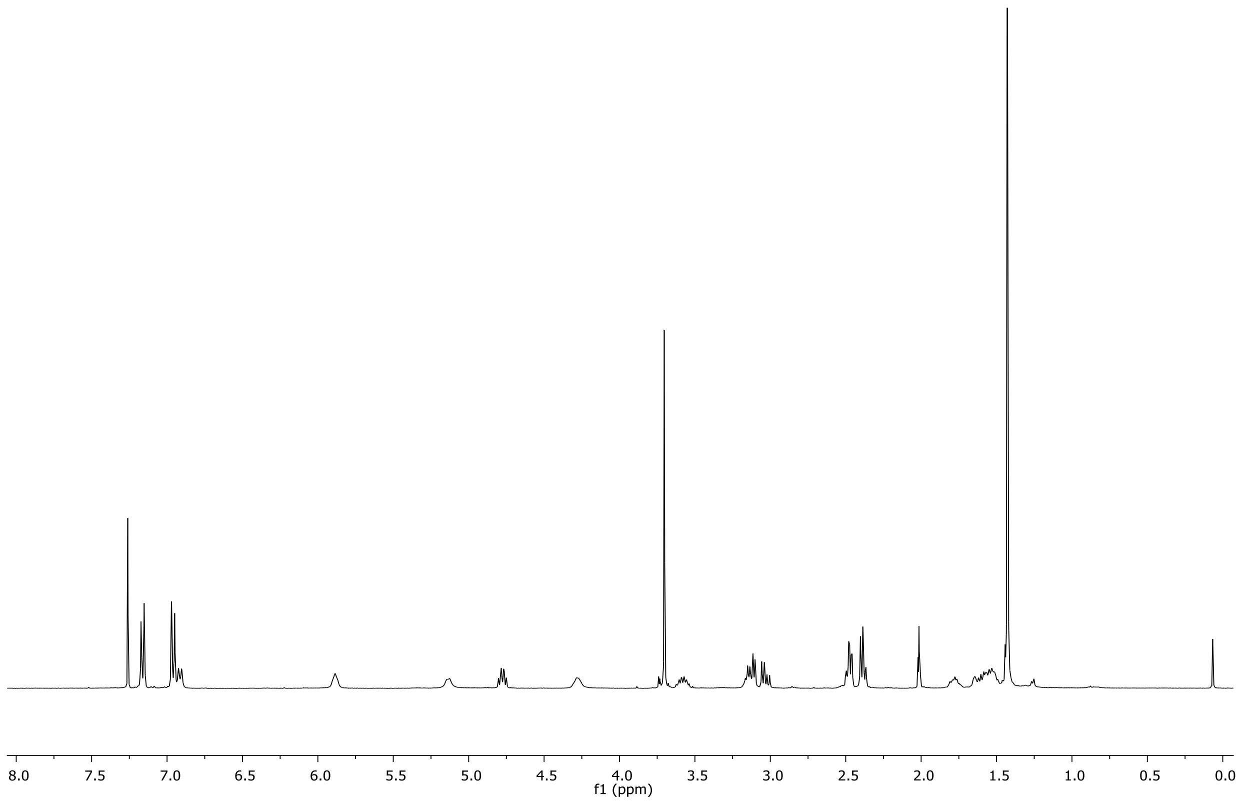

8.0
7.5
7.0
6.5
6.0
5.5
5.0
4.5
4.0
3.5
3.0
2.5
2.0
1.5
1.0
0.5
0.0
f1 (ppm)

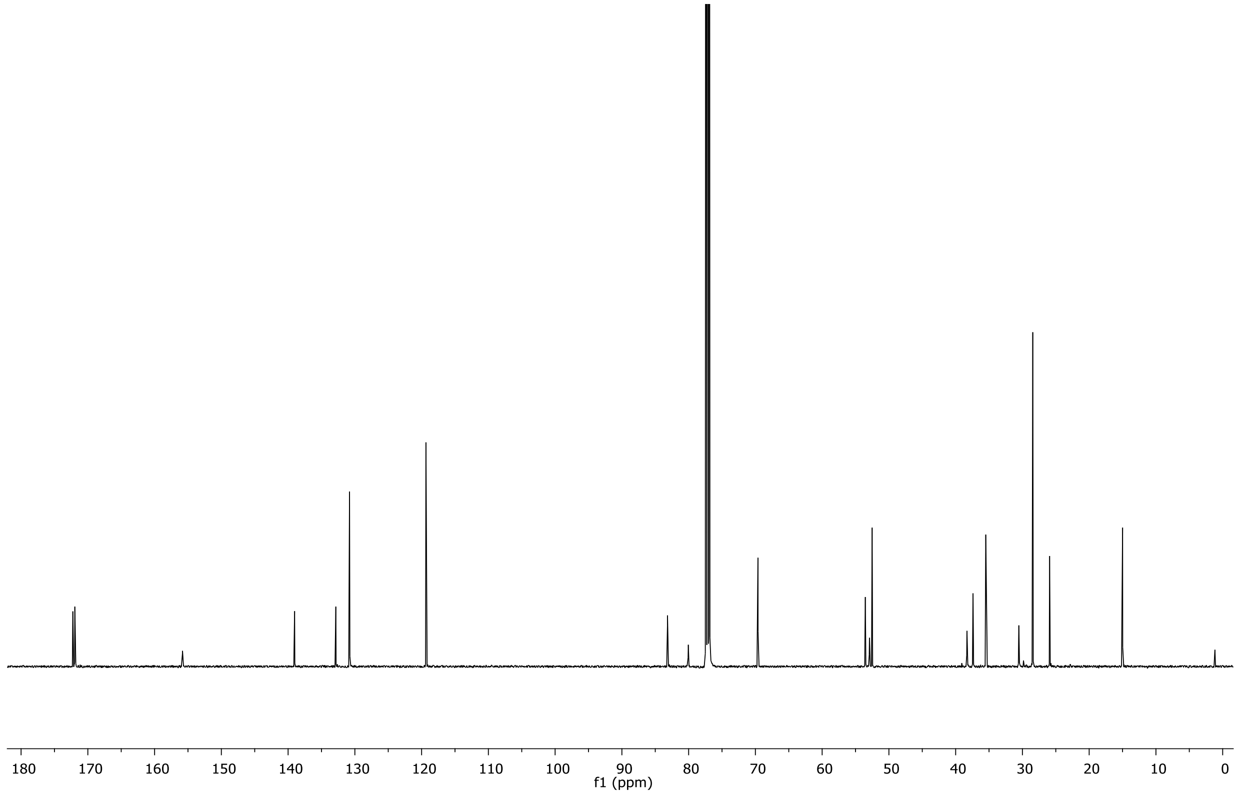

180
170
160
150
140
130
120
110
100
90
80
70
60
50
40
30
20
10
0
f1 (ppm)

C3

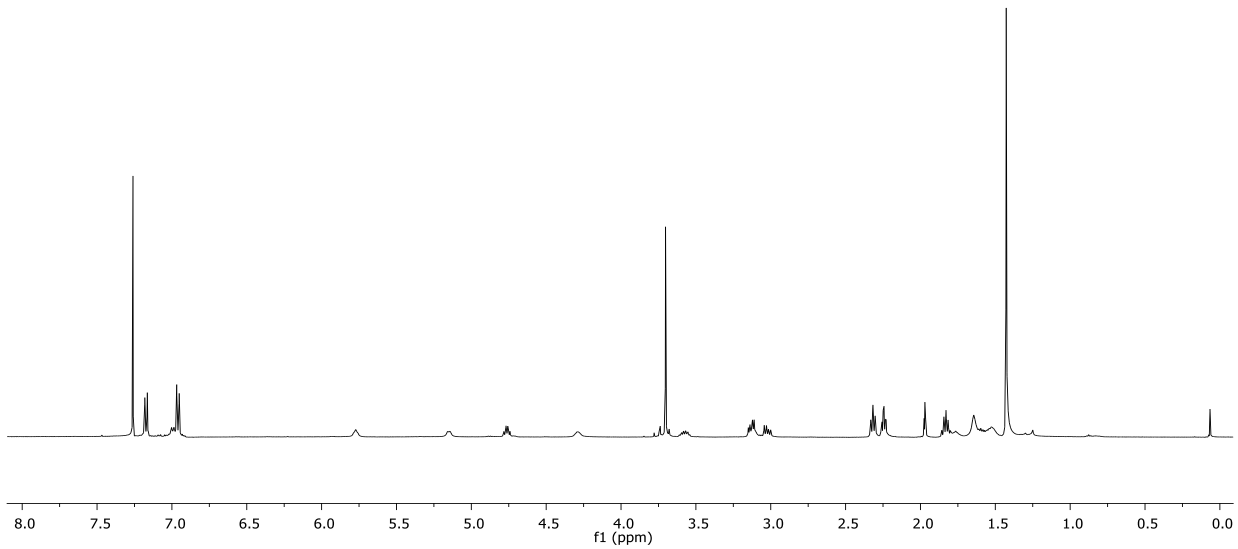
8.0
7.5
7.0
6.5
6.0
5.5
5.0
4.5
4.0
3.5
3.0
2.5
2.0
1.5
1.0
0.5
0.0
f1 (ppm)

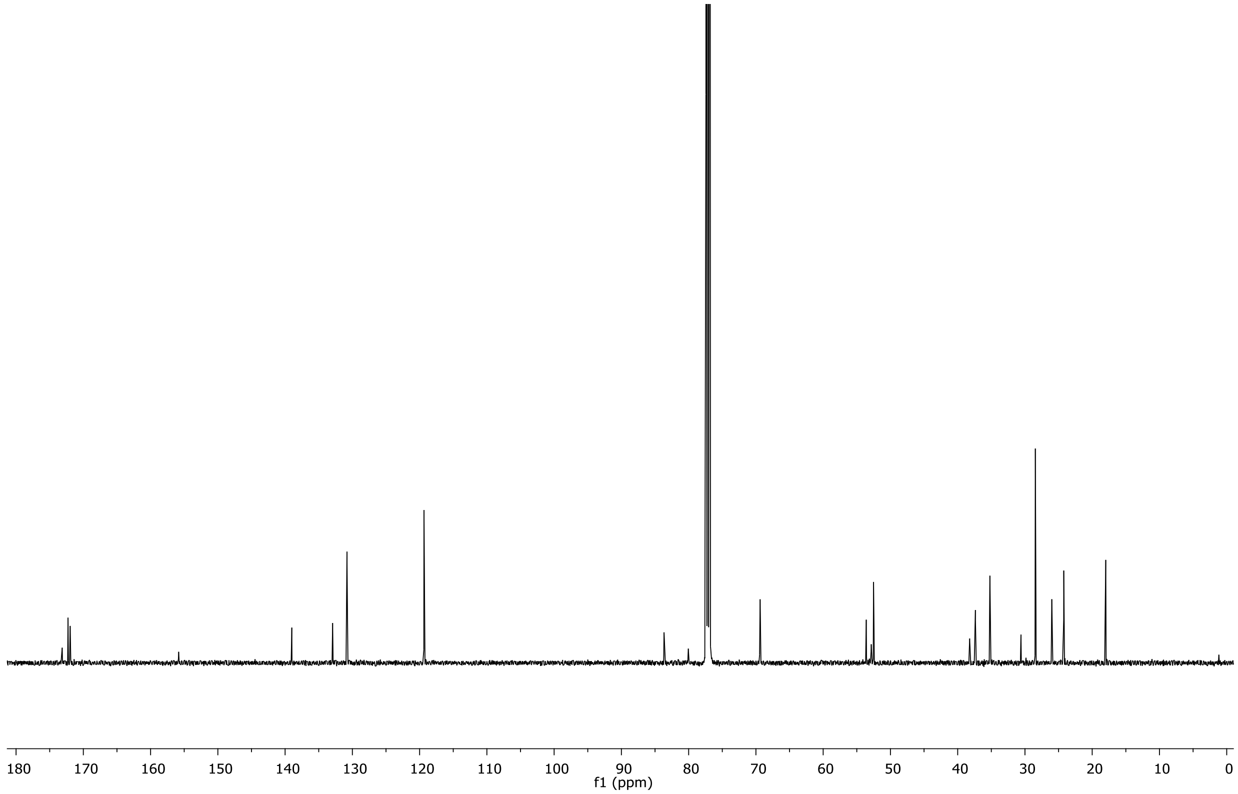
180
170
160
150
140
130
120
110
100
90
80
70
60
50
40
30
20
10
0
f1 (ppm)

C7

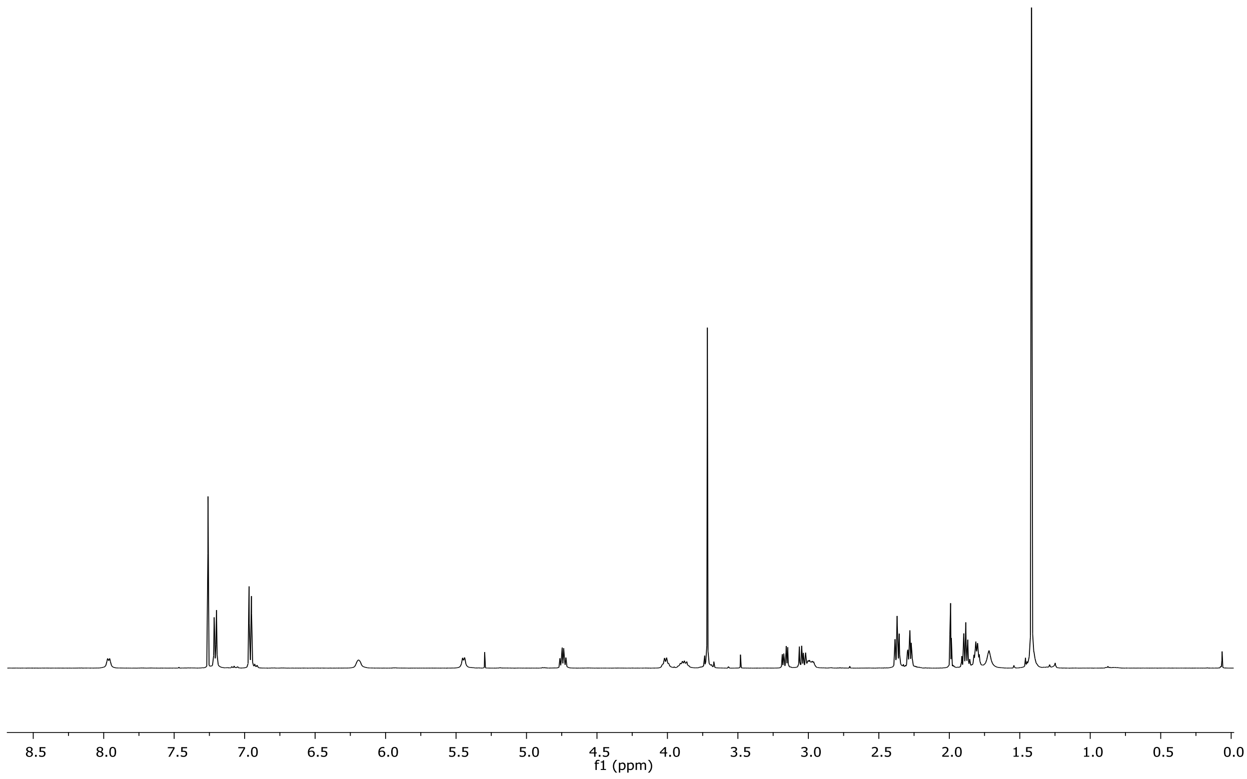

8.5
8.0
7.5
7.0
6.5
6.0
5.5
5.0
4.5
4.0
3.5
3.0
2.5
2.0
1.5
1.0
0.5
0.0
f1 (ppm)

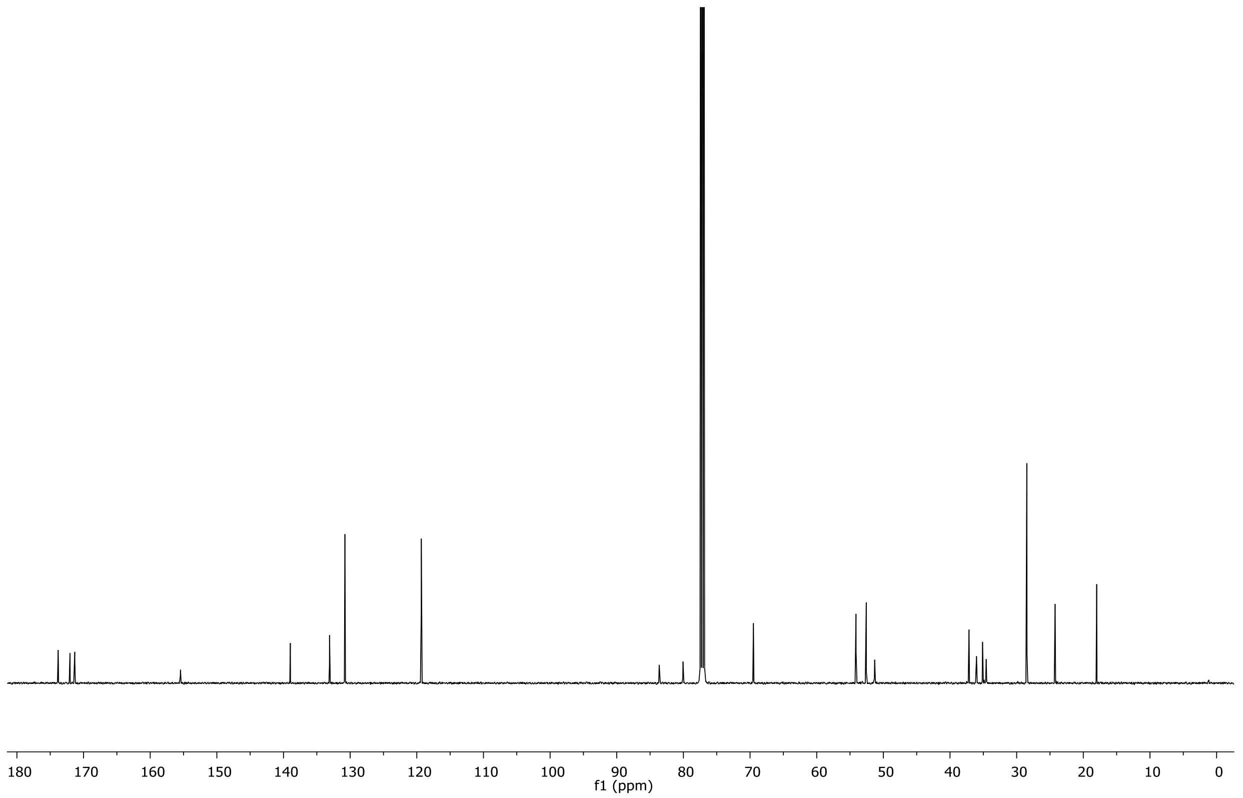

180
170
160
150
140
130
120
110
100
90
80
70
60
50
40
30
20
10
0
f1 (ppm)

C8

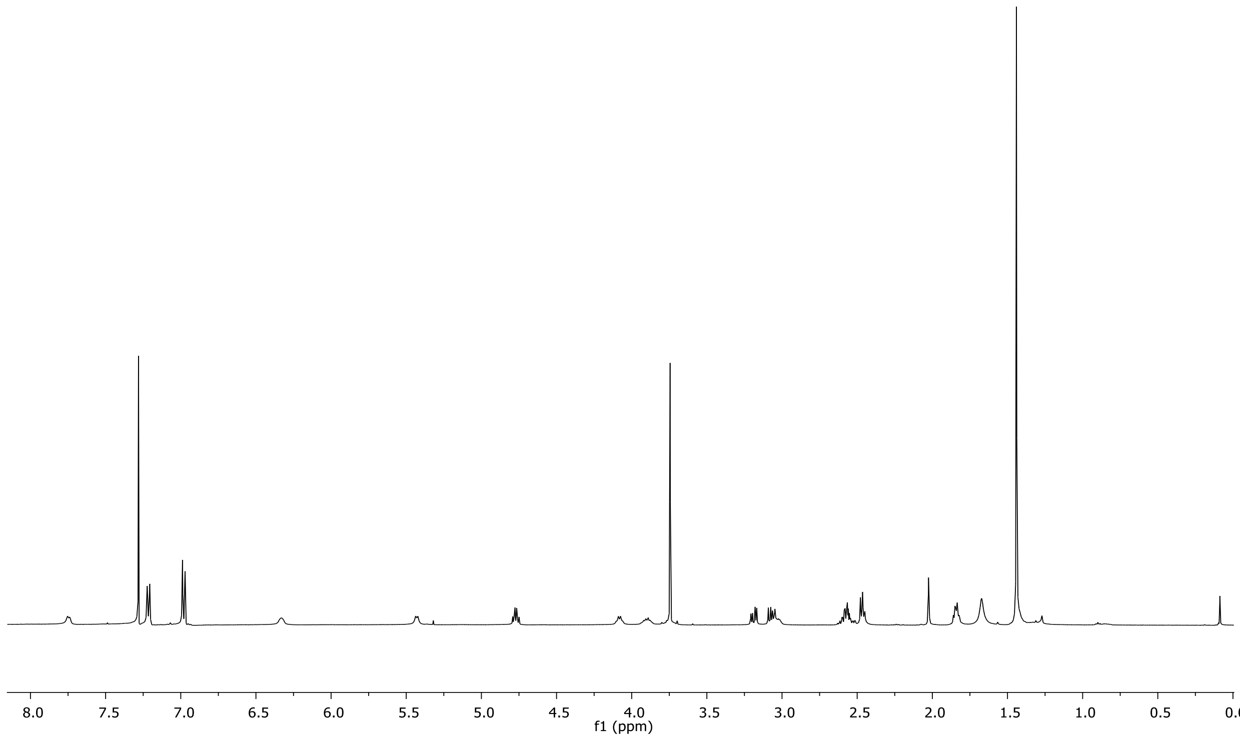
8.0 7.5 7.0 6.5 6.0 5.5 5.0 4.5 4.0 3.5 3.0 2.5 2.0 1.5 1.0 0.5 0.0
f1 (ppm)

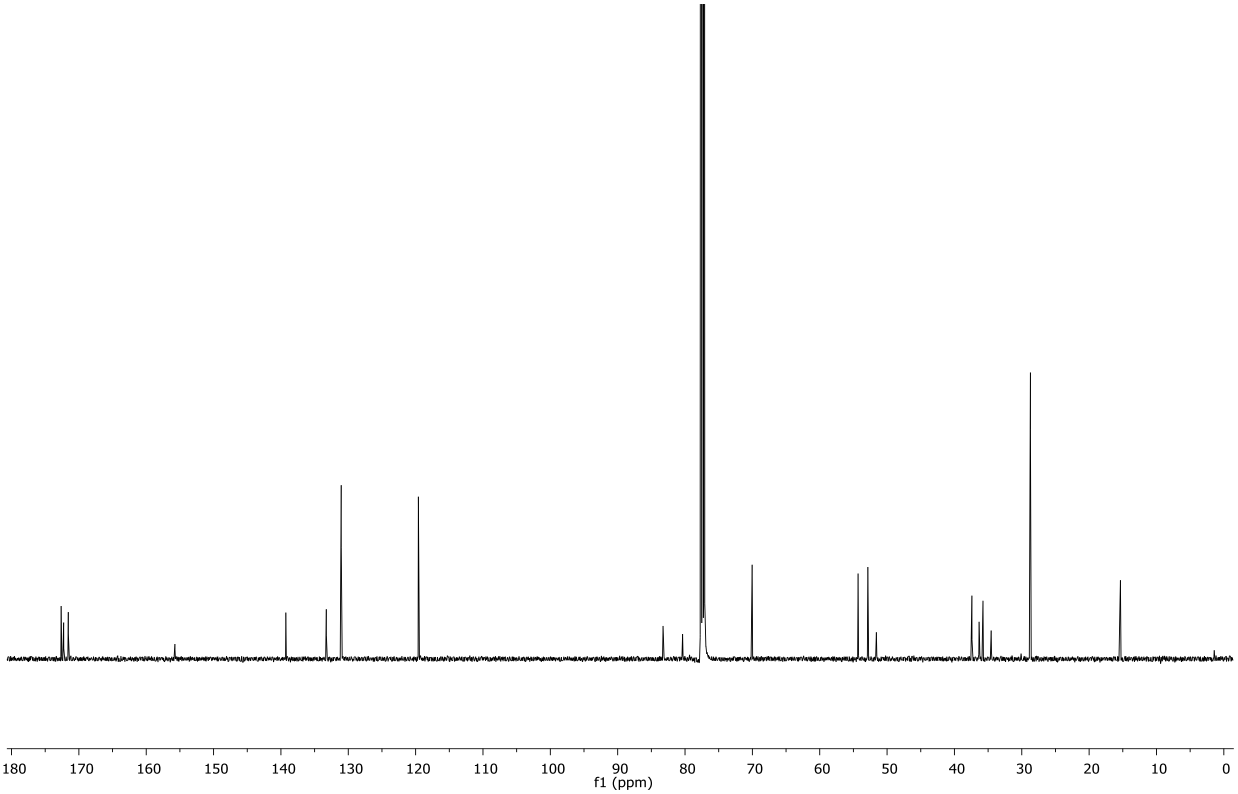
180 170 160 150 140 130 120 110 100 90 80 70 60 50 40 30 20 10 0
f1 (ppm)

C10

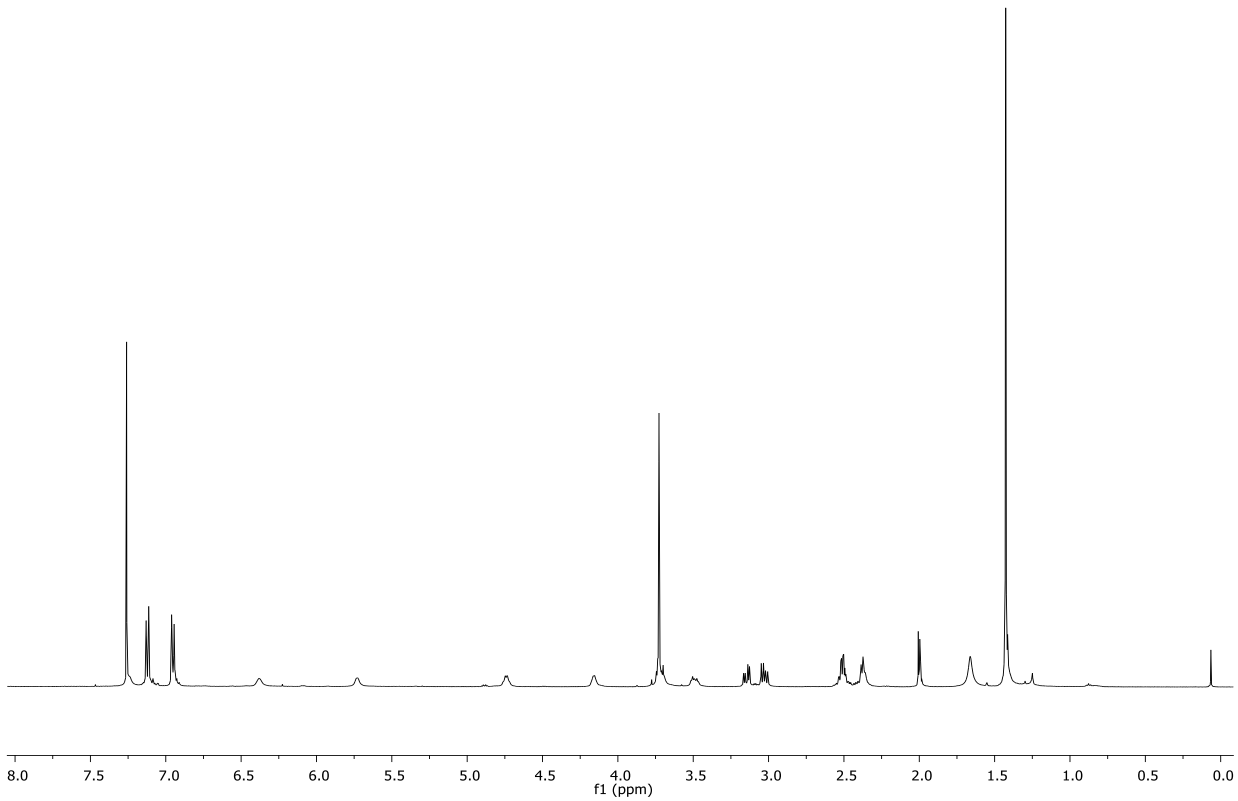
8.0
7.5
7.0
6.5
6.0
5.5
5.0
4.5
4.0
3.5
3.0
2.5
2.0
1.5
1.0
0.5
0.0
f1 (ppm)

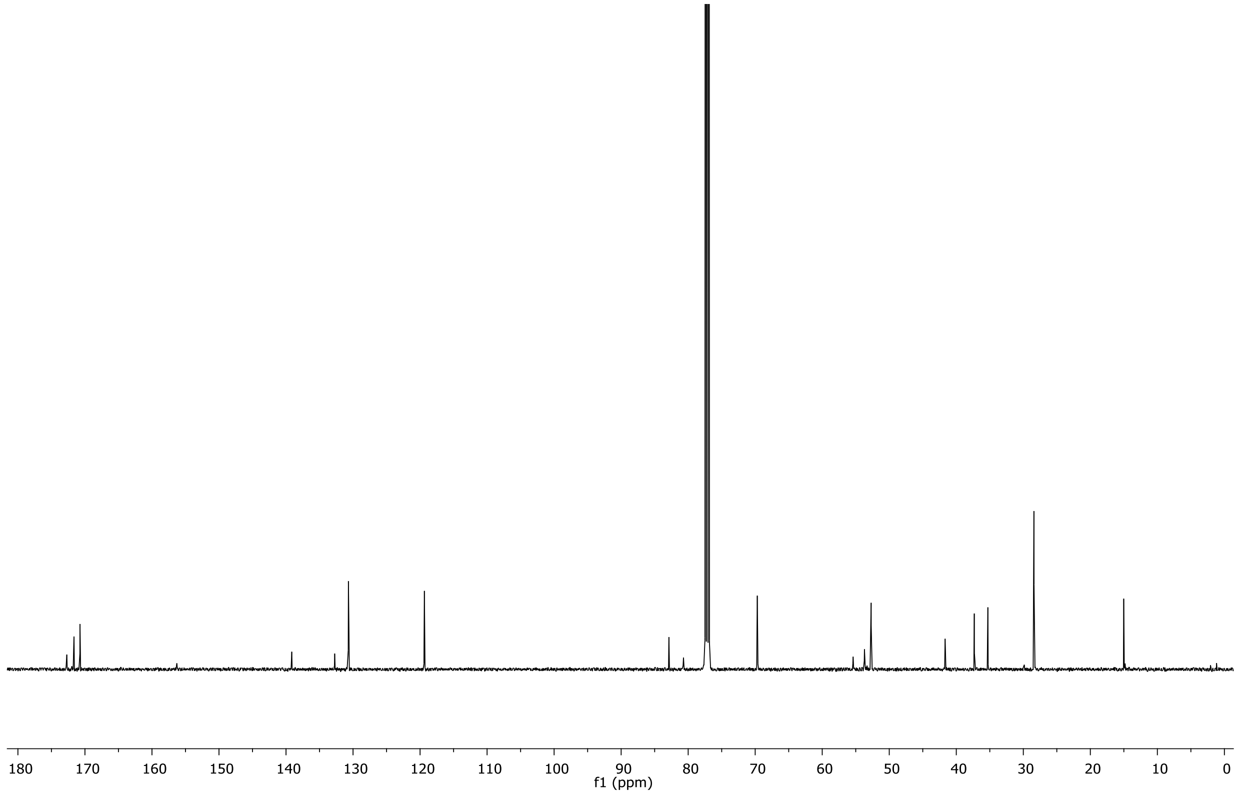
180
170
160
150
140
130
120
110
100
90
80
70
60
50
40
30
20
10
0
f1 (ppm)

C12

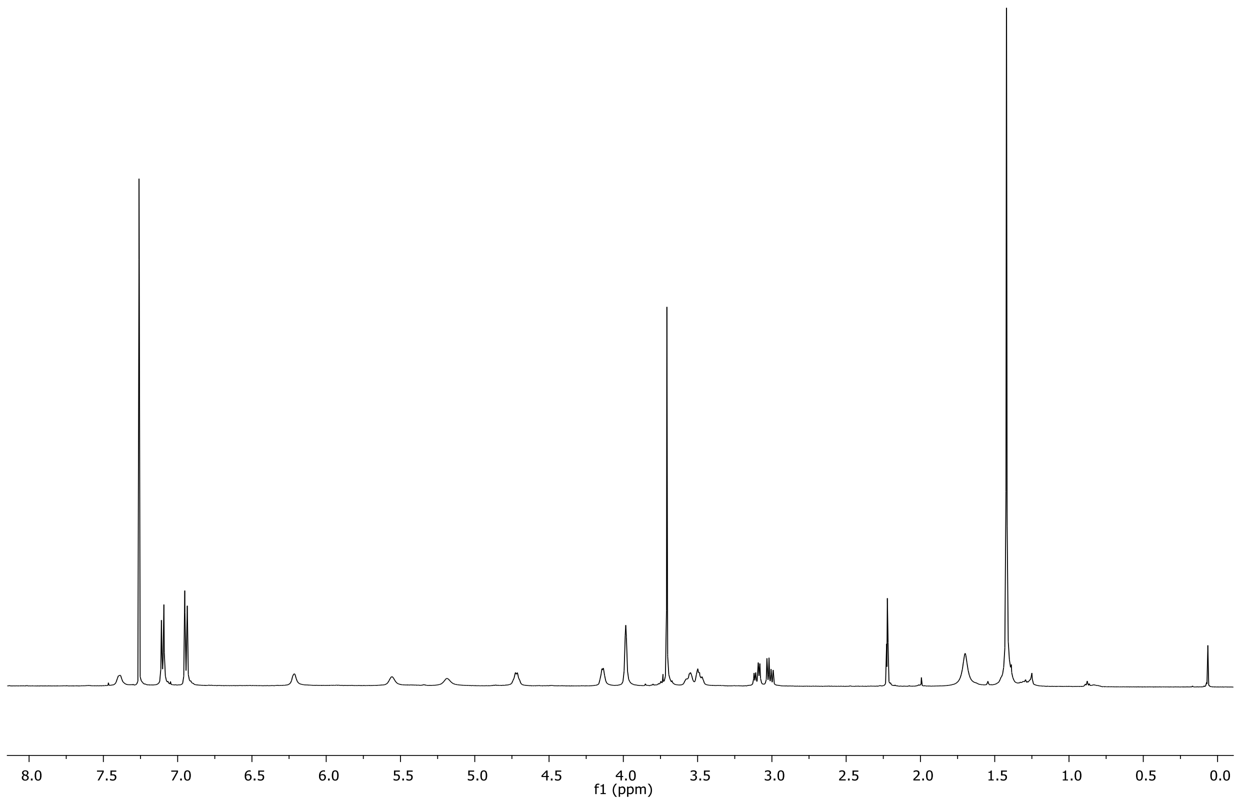

8.0
7.5
7.0
6.5
6.0
5.5
5.0
4.5
4.0
3.5
3.0
2.5
2.0
1.5
1.0
0.5
0.0
f1 (ppm)

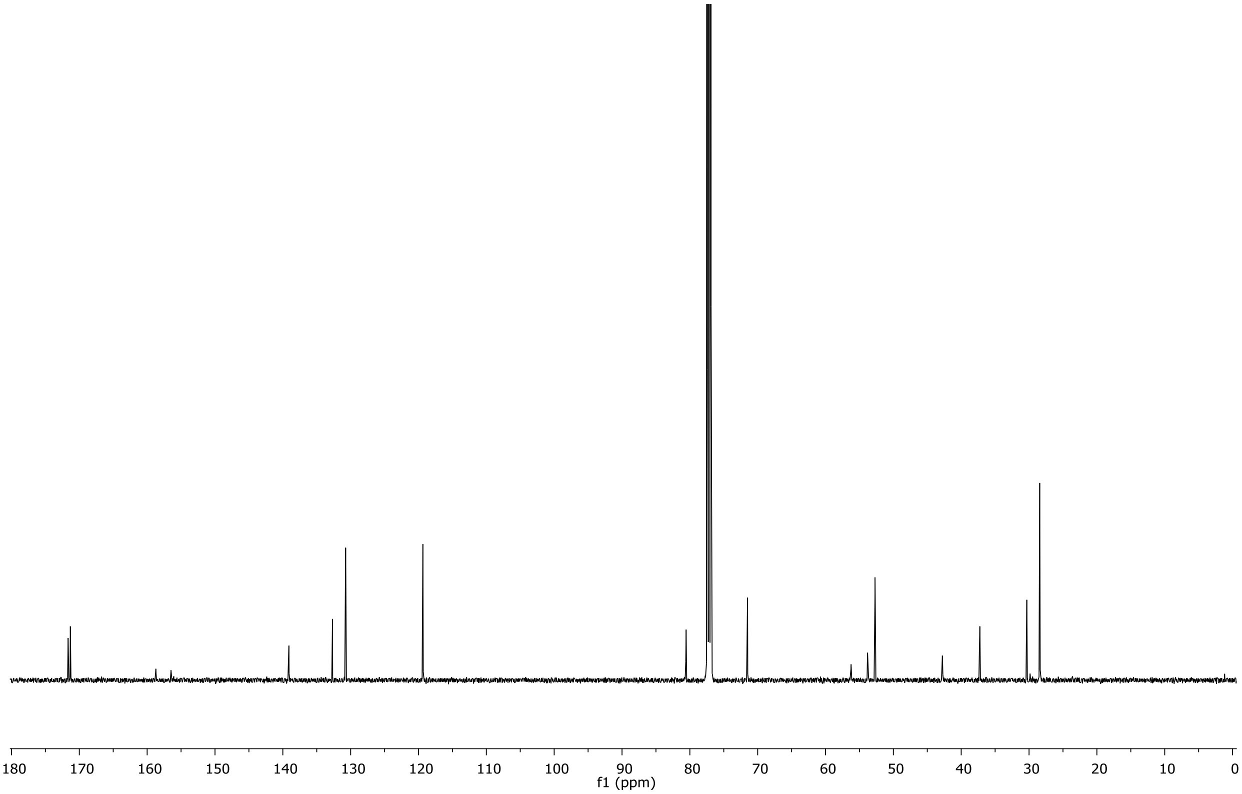

180
170
160
150
140
130
120
110
100
90
80
70
60
50
40
30
20
10
0
f1 (ppm)

C14

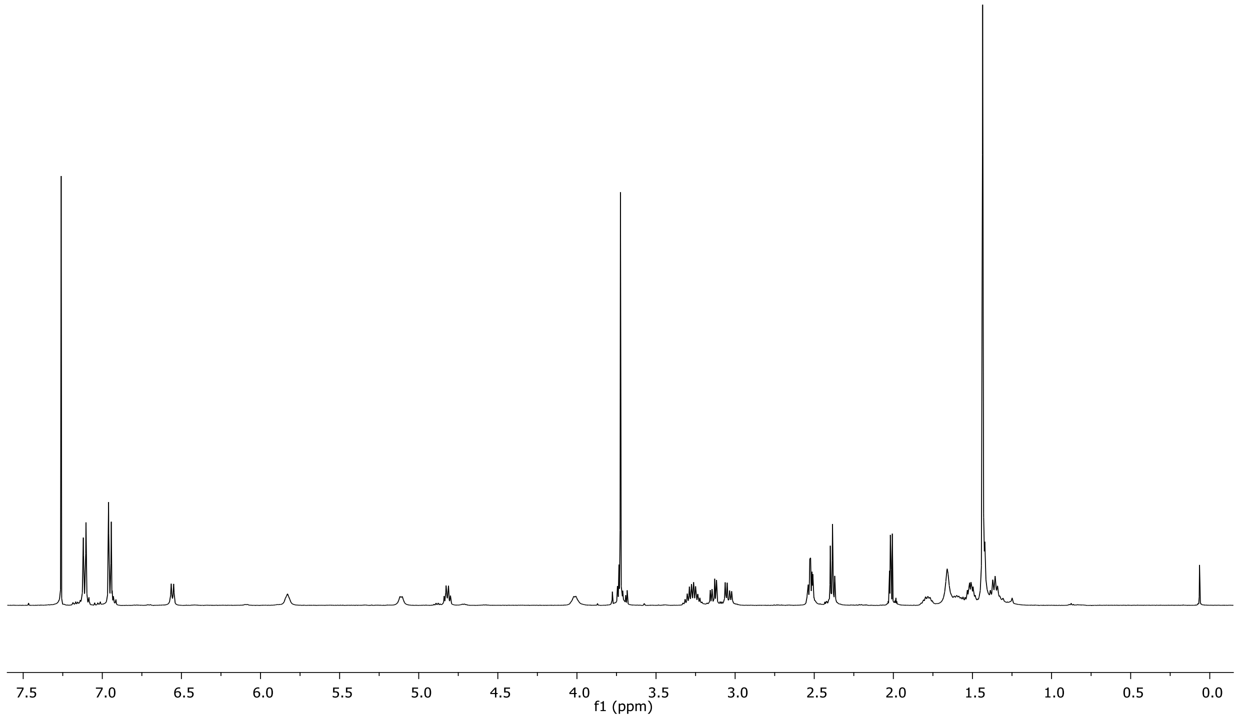
7.5
7.0
6.5
6.0
5.5
5.0
4.5
4.0
3.5
3.0
2.5
2.0
1.5
1.0
0.5
0.0
f1 (ppm)

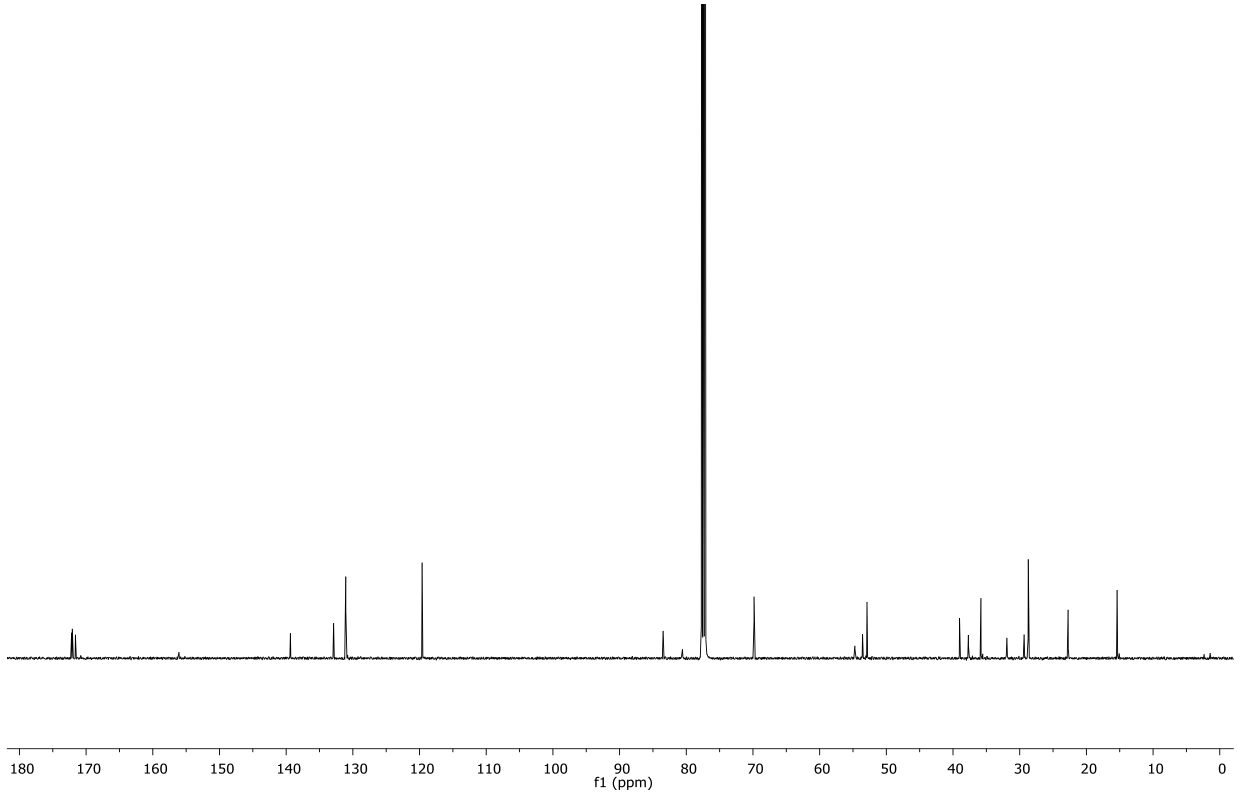
180
170
160
150
140
130
120
110
100
90
80
70
60
50
40
30
20
10
0
f1 (ppm)

D3

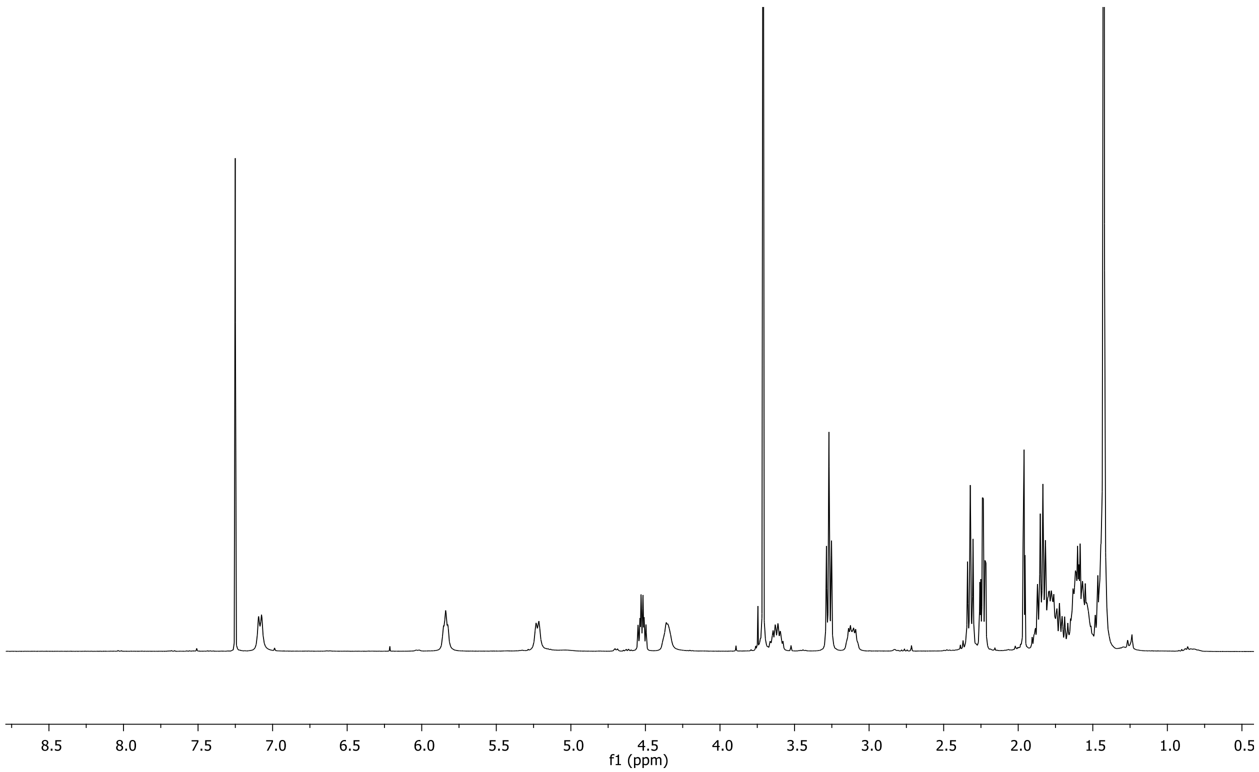
8.5
8.0
7.5
7.0
6.5
6.0
5.5
5.0
4.5
4.0
3.5
3.0
2.5
2.0
1.5
1.0
0.5
f1 (ppm)

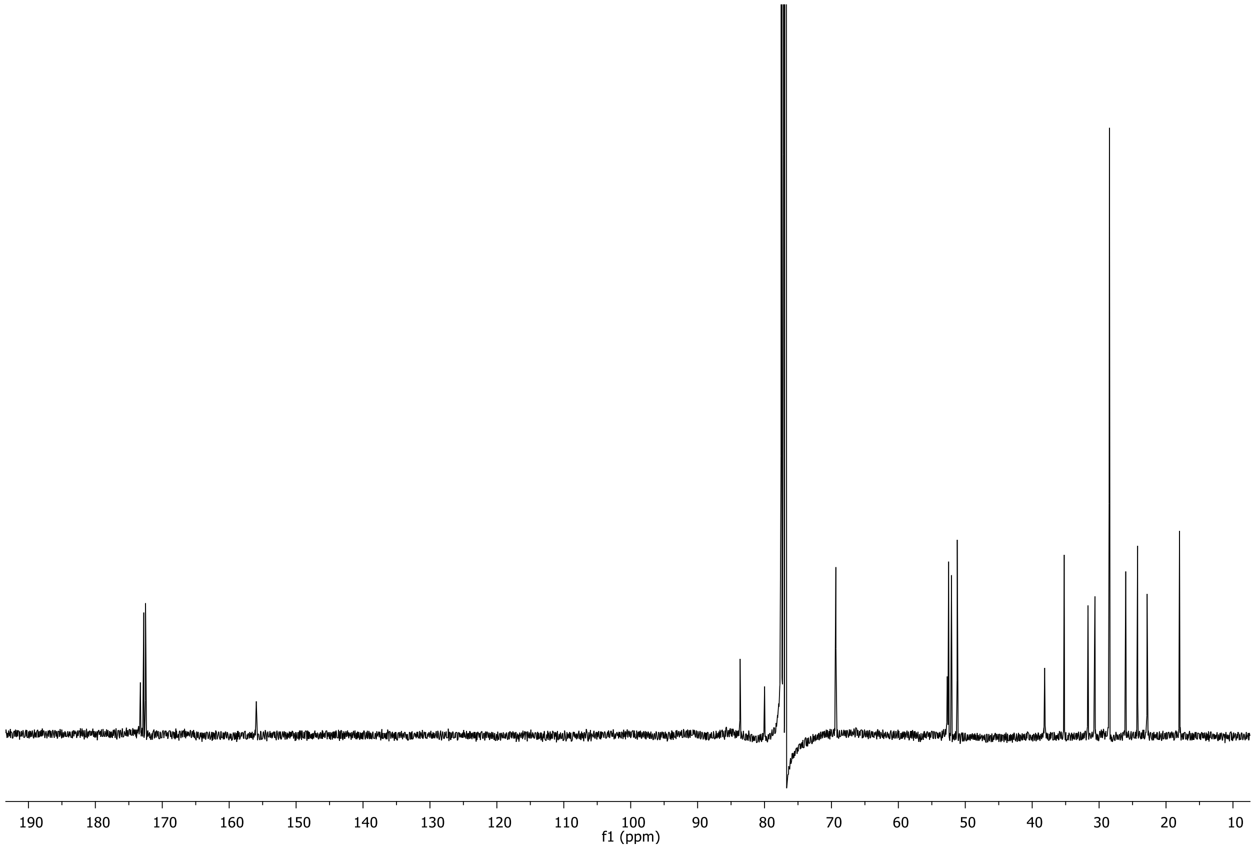
190
180
170
160
150
140
130
120
110
100
90
80
70
60
50
40
30
20
10
f1 (ppm)

D8

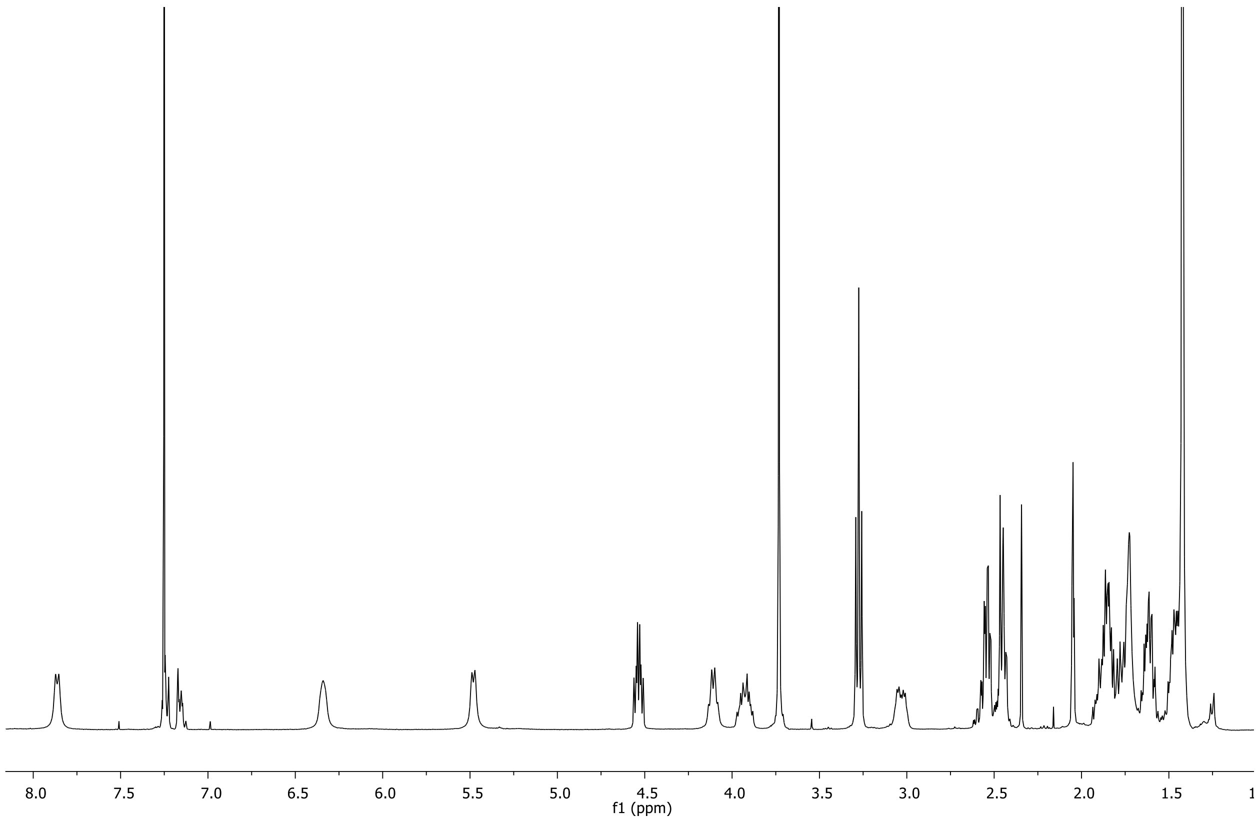
8.0
7.5
7.0
6.5
6.0
5.5
5.0
4.5
4.0
3.5
3.0
2.5
2.0
1.5
f1 (ppm)

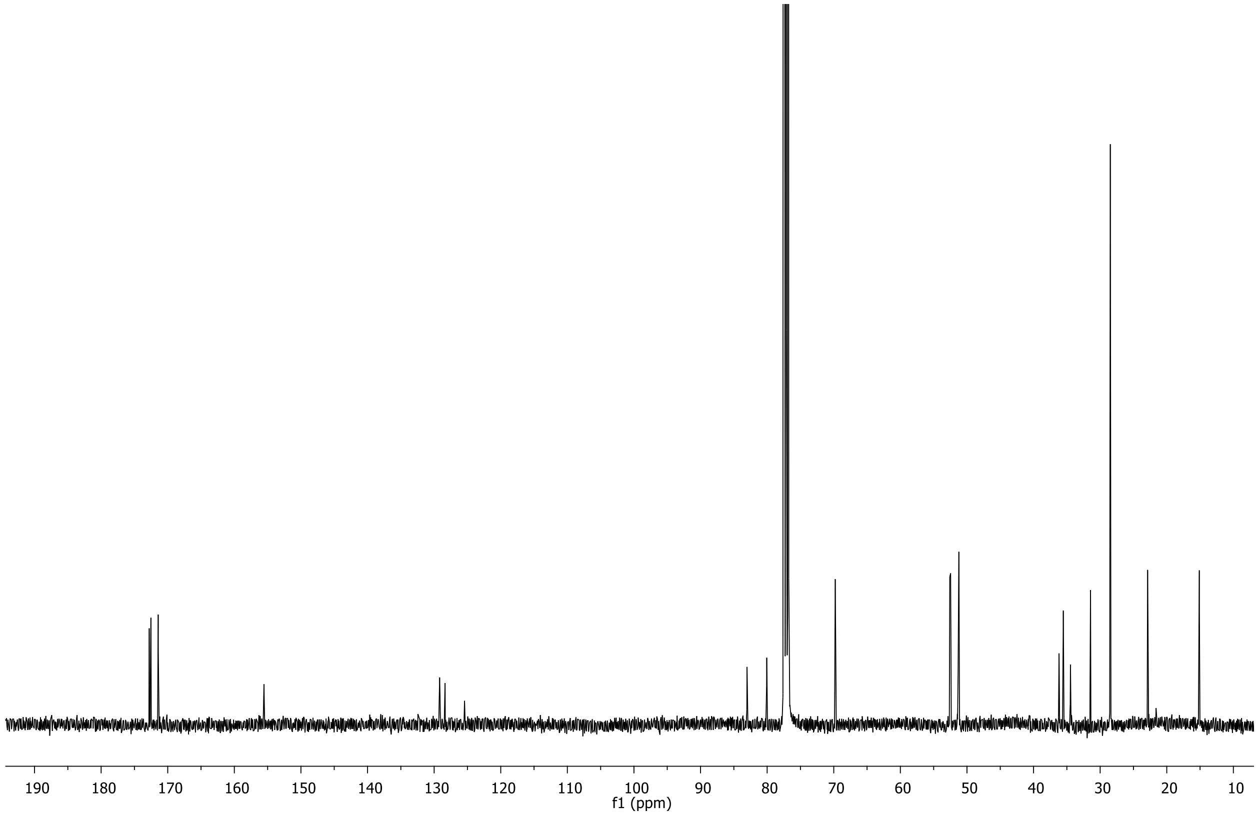
190
180
170
160
150
140
130
120
110
100
90
80
70
60
50
40
30
20
10
f1 (ppm)

D11

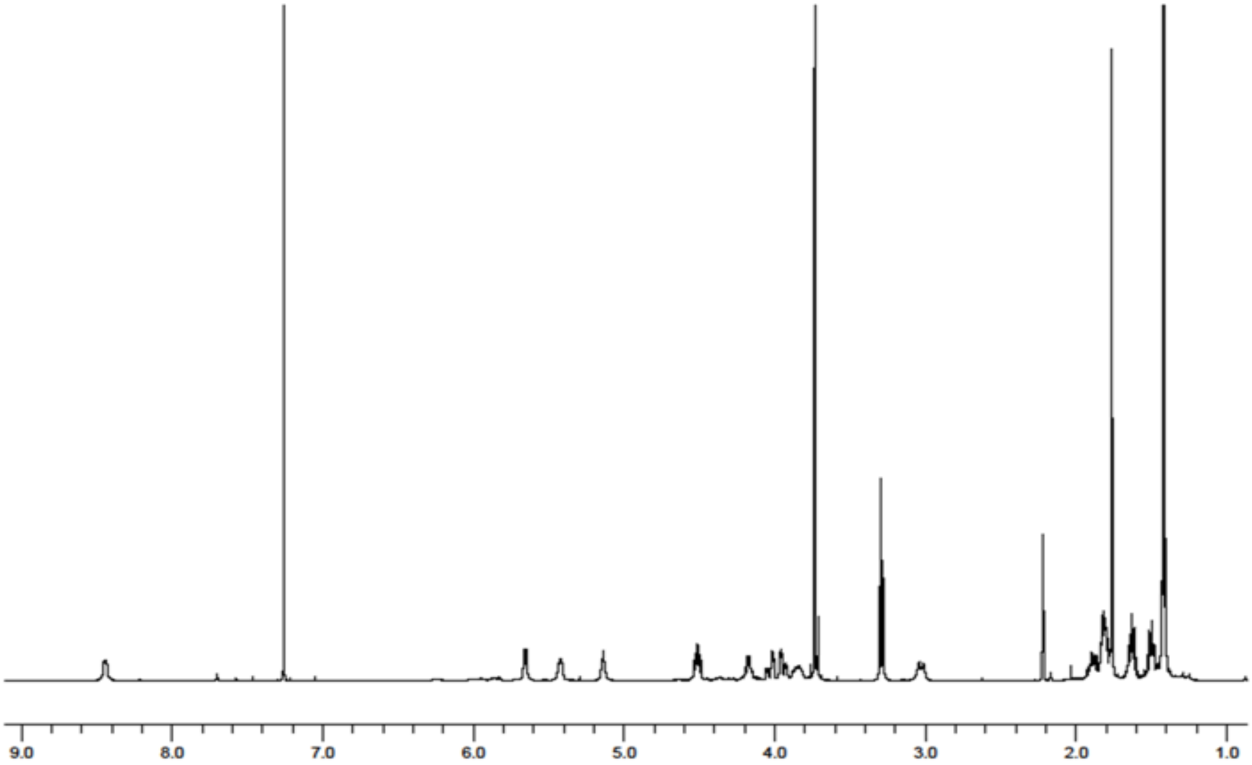

9.0
8.0
7.0
6.0
5.0
4.0
3.0
2.0
1.0

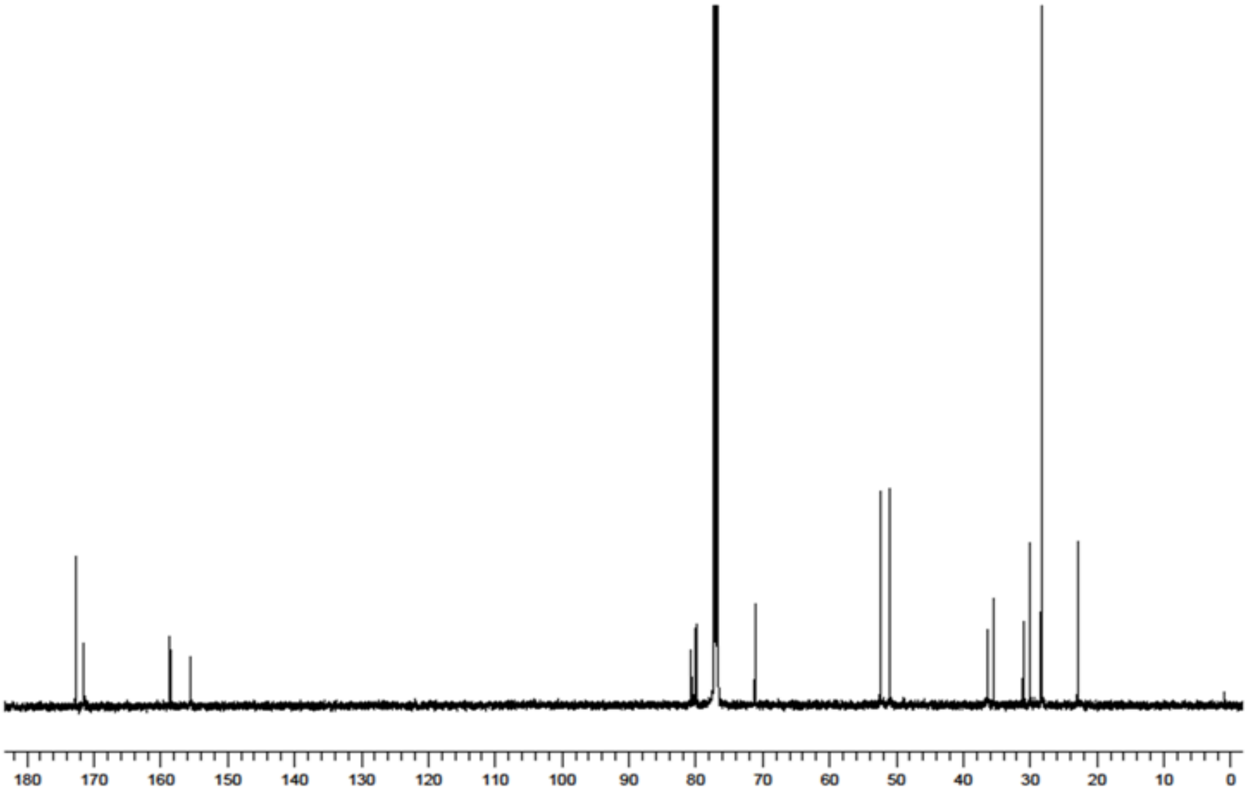

180
170
160
150
140
130
120
110
100
90
80
70
60
50
40
30
20
10
0

E3

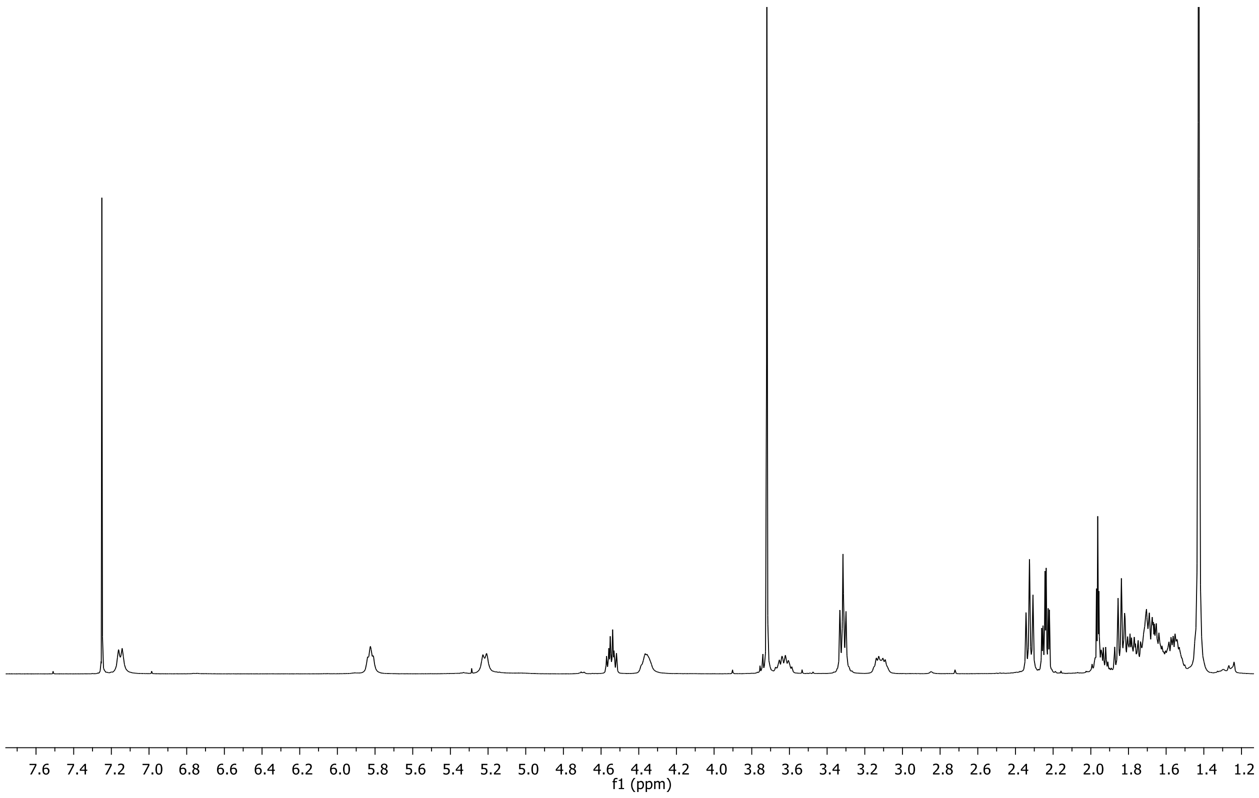
7.6 7.4 7.2 7.0 6.8 6.6 6.4 6.2 6.0 5.8 5.6 5.4 5.2 5.0 4.8 4.6 4.4 4.2 4.0 3.8 3.6 3.4 3.2 3.0 2.8 2.6 2.4 2.2 2.0 1.8 1.6 1.4 1.2
f1 (ppm)

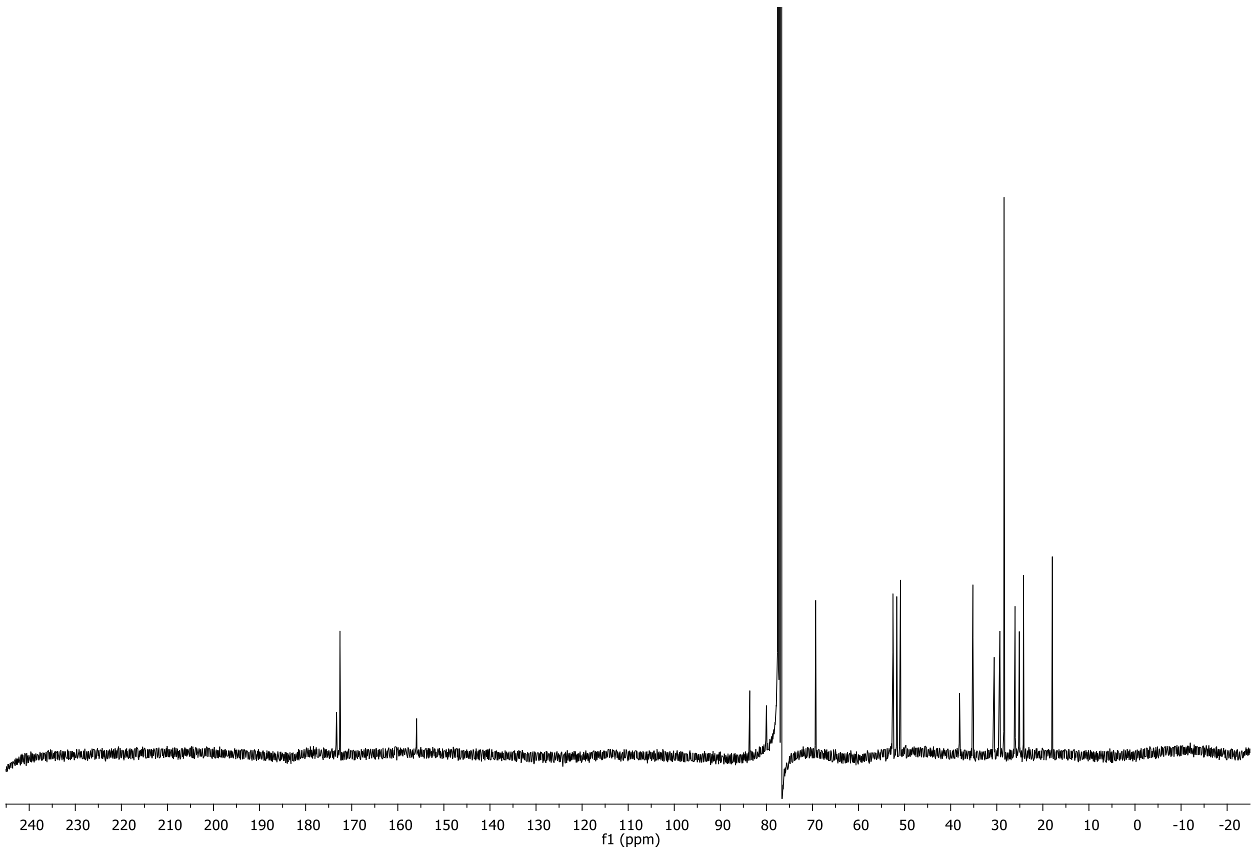
240 230 220 210 200 190 180 170 160 150 140 130 120 110 100 90 80 70 60 50 40 30 20 10 0 -10 -20
f1 (ppm)

E5

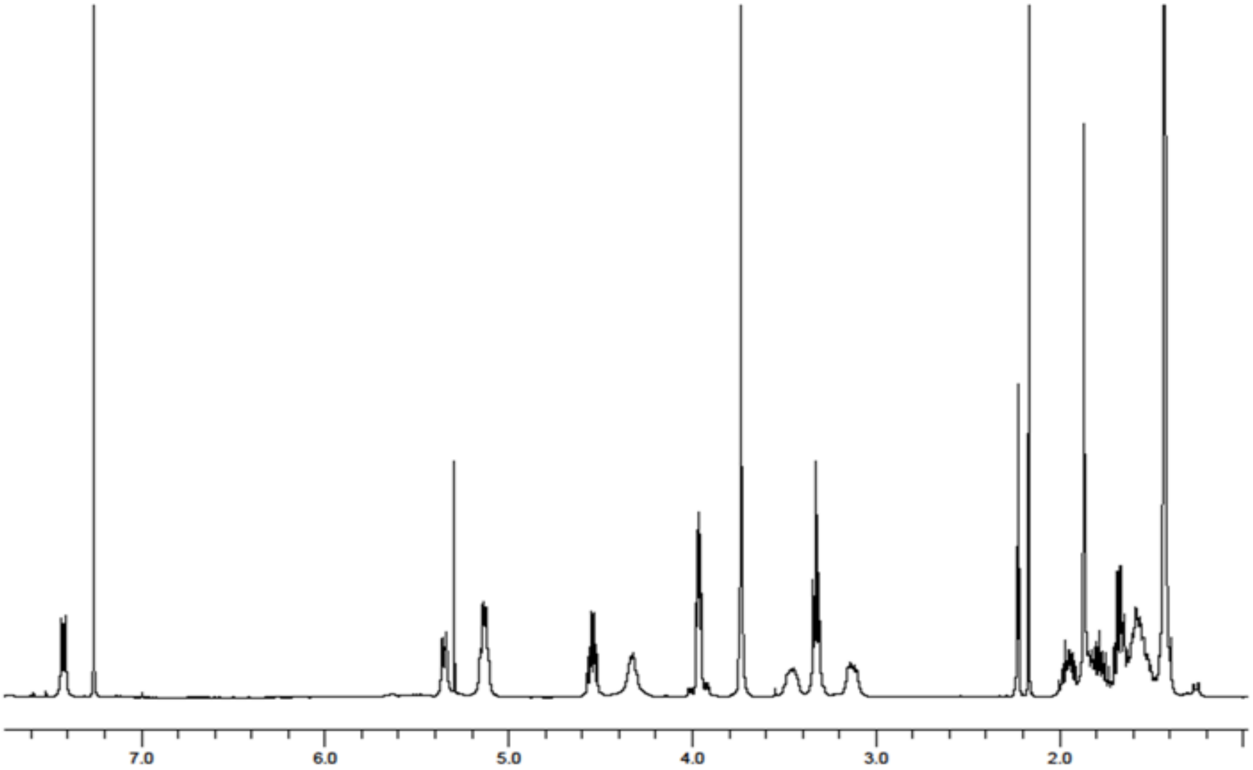
7.0
6.0
5.0
4.0
3.0
2.0

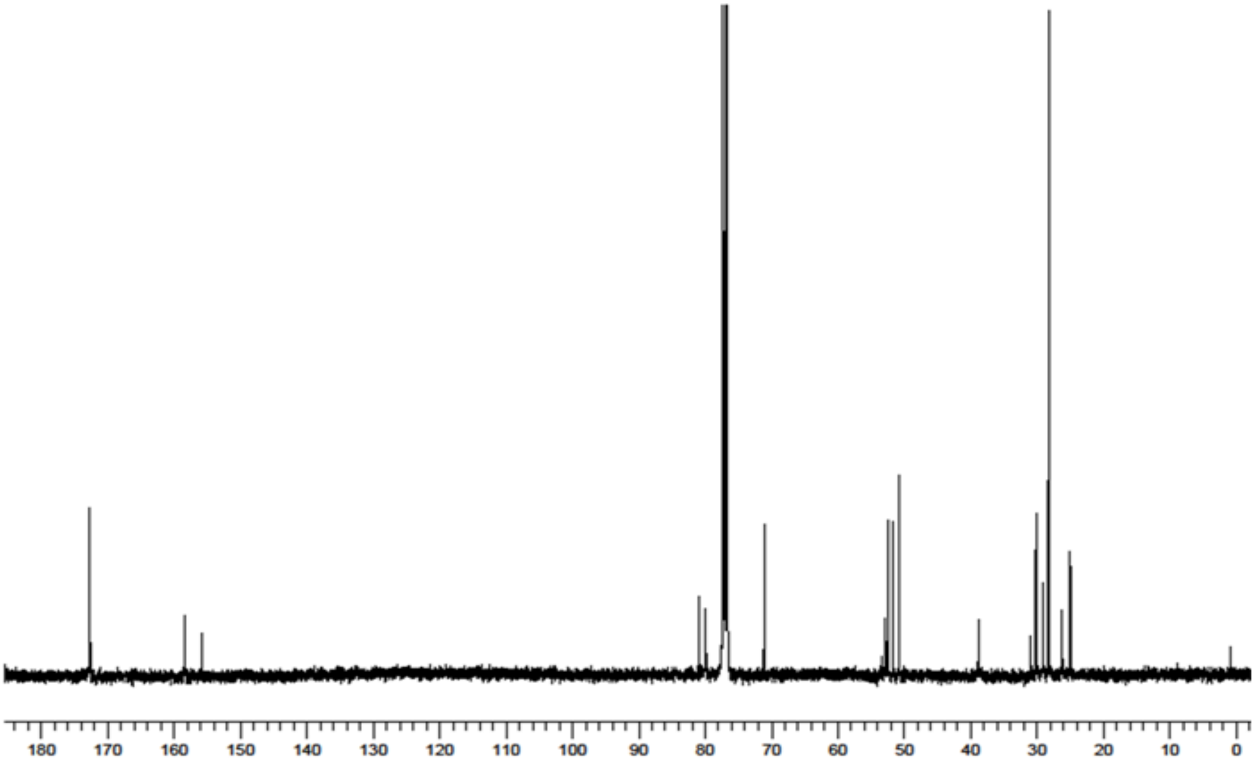
180
170
160
150
140
130
120
110
100
90
80
70
60
50
40
30
20
10
0

E7

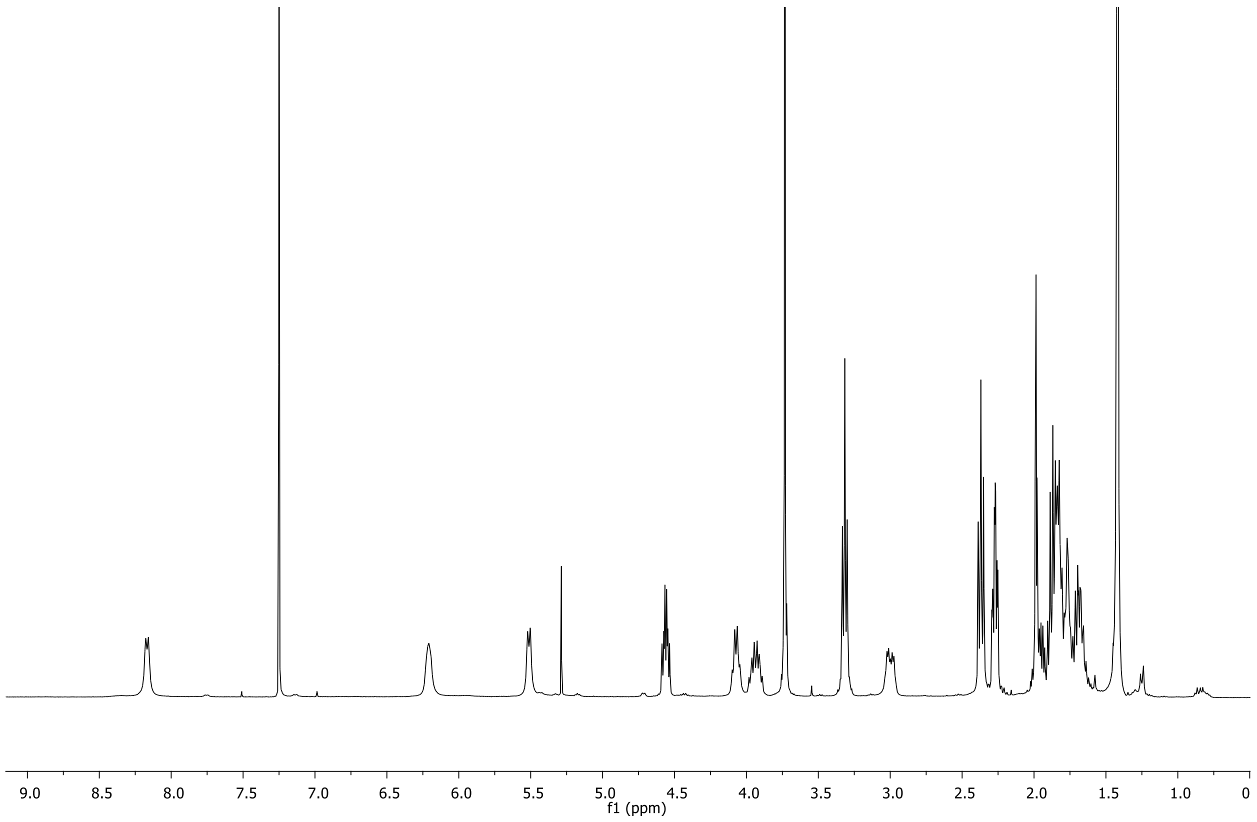

9.0 8.5 8.0 7.5 7.0 6.5 6.0 5.5 5.0 4.5 4.0 3.5 3.0 2.5 2.0 1.5 1.0 0
f1 (ppm)

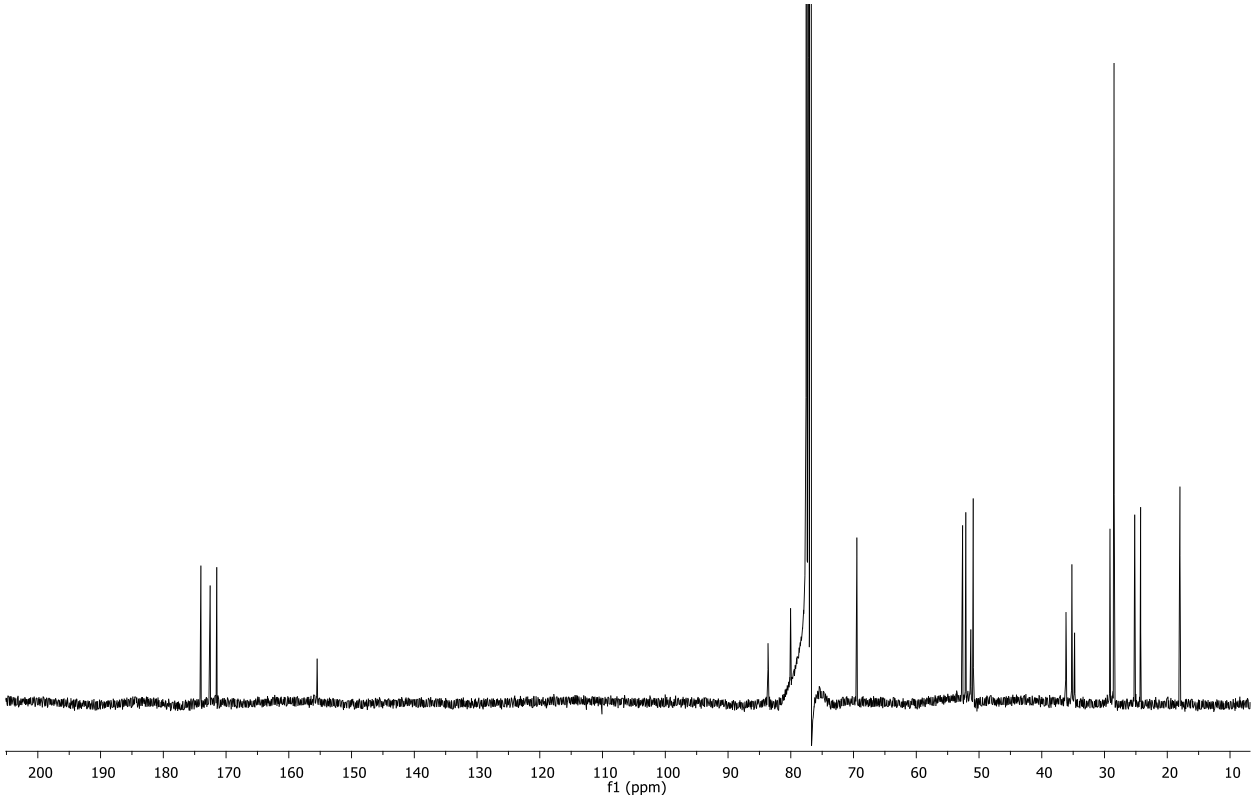

200 190 180 170 160 150 140 130 120 110 100 90 80 70 60 50 40 30 20 10
f1 (ppm)

G4

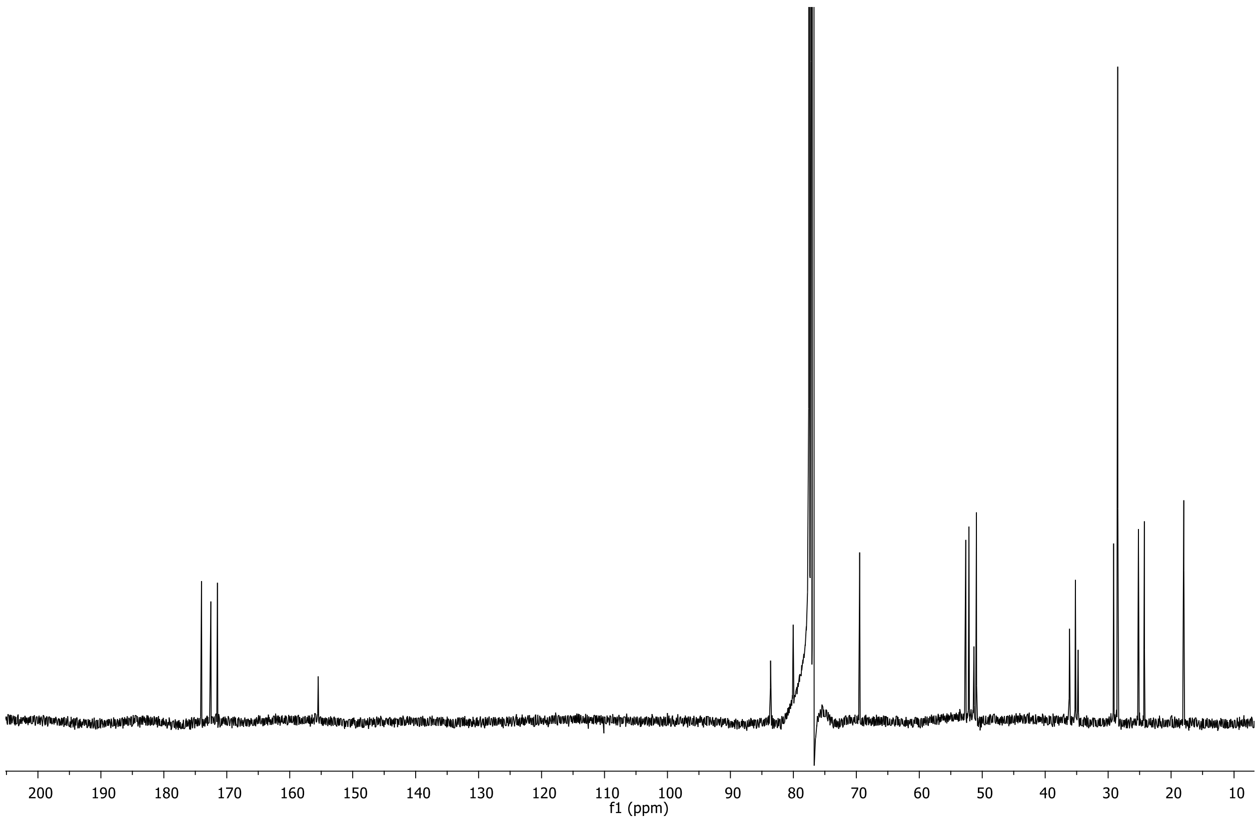

200 190 180 170 160 150 140 130 120 110 100 90 80 70 60 50 40 30 20 10
f1 (ppm)

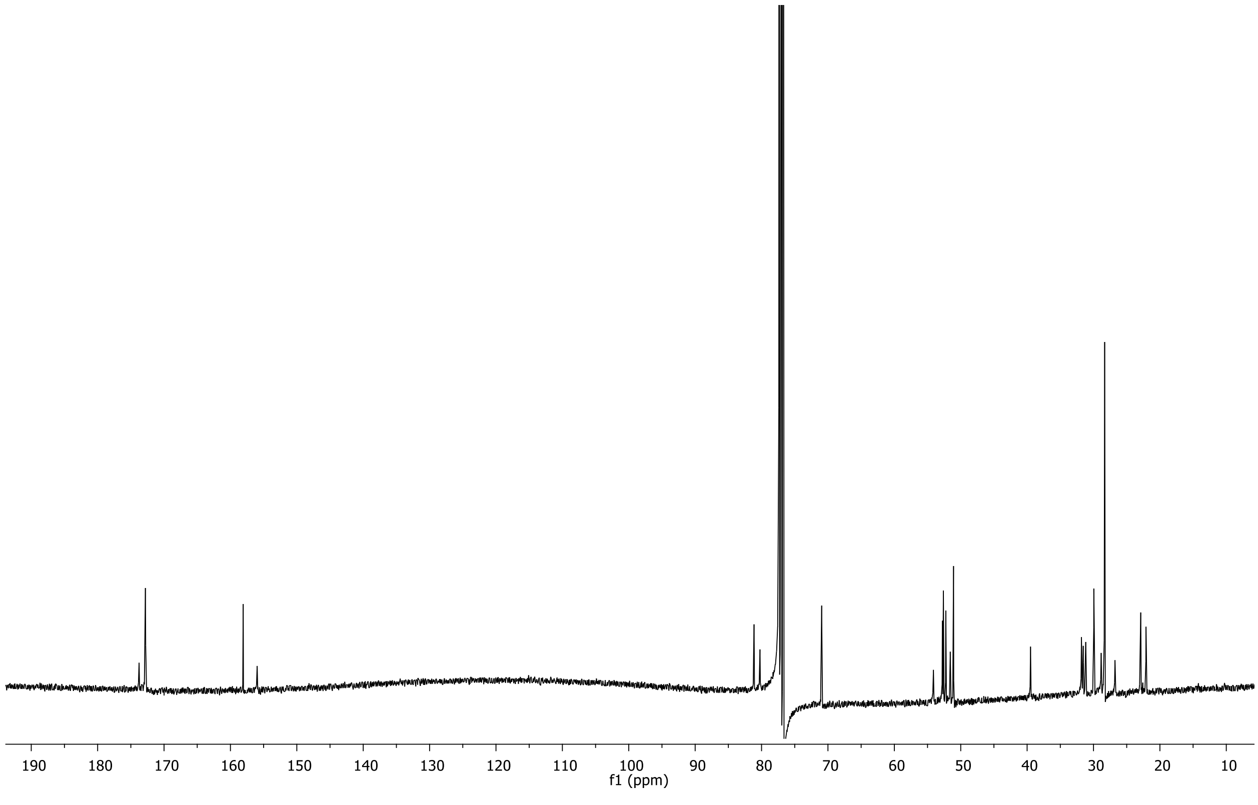

190 180 170 160 150 140 130 120 110 100 90 80 70 60 50 40 30 20 10
f1 (ppm)

G5

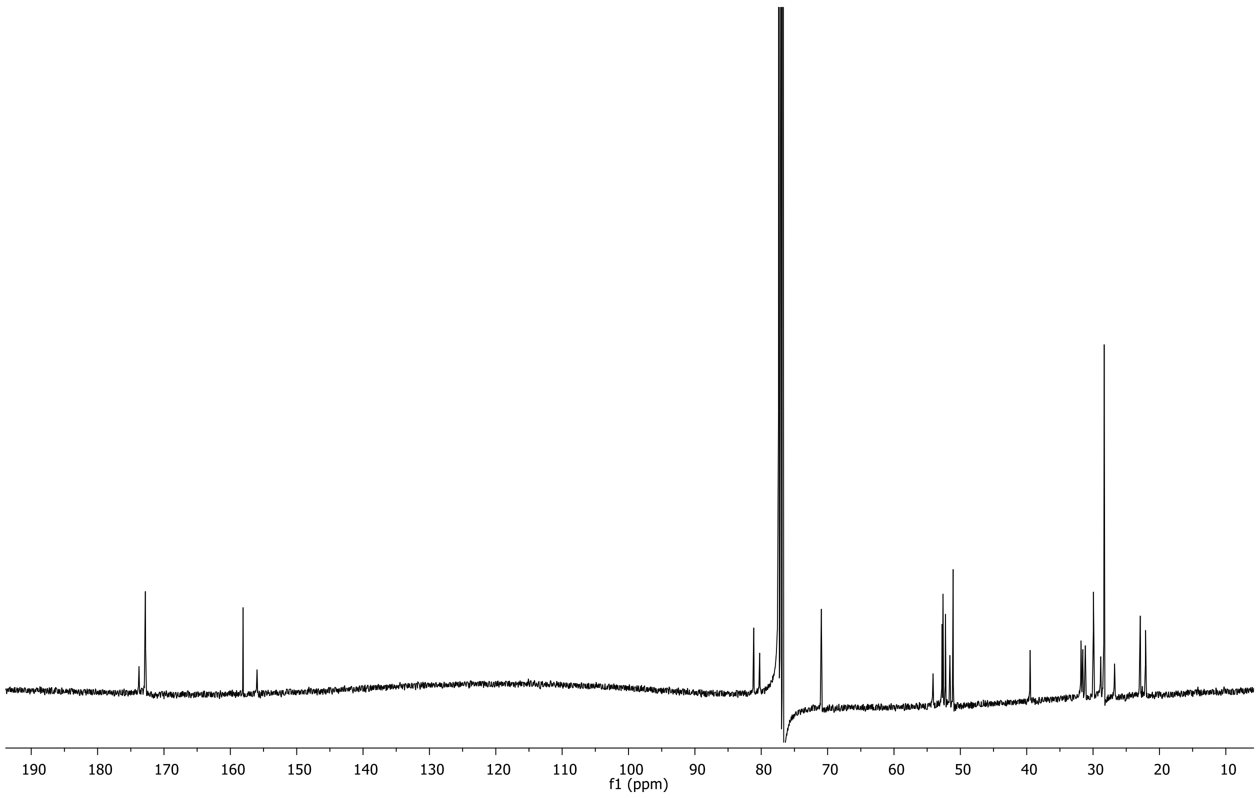
190
180
170
160
150
140
130
120
110
100
90
80
70
60
50
40
30
20
10
f1 (ppm)

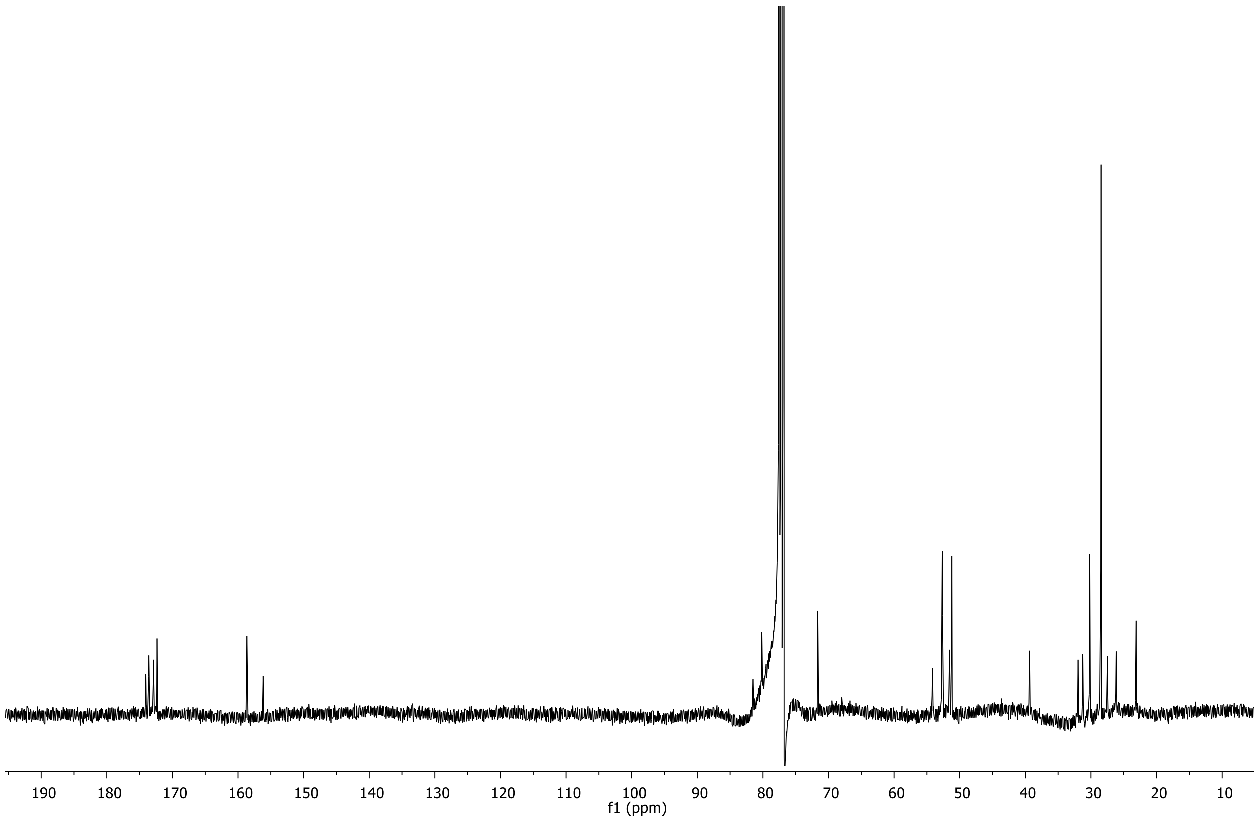
190
180
170
160
150
140
130
120
110
100
90
80
70
60
50
40
30
20
10
f1 (ppm)

H10

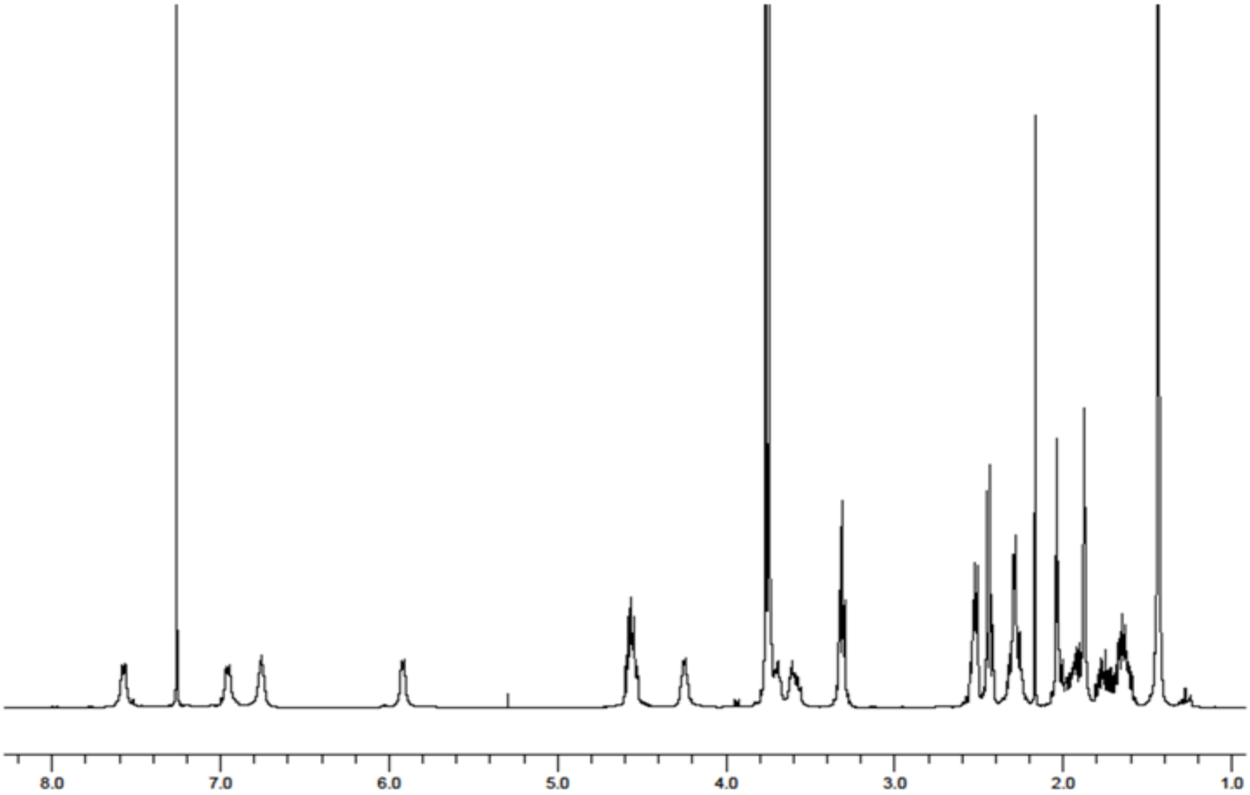
8.0
7.0
6.0
5.0
4.0
3.0
2.0
1.0

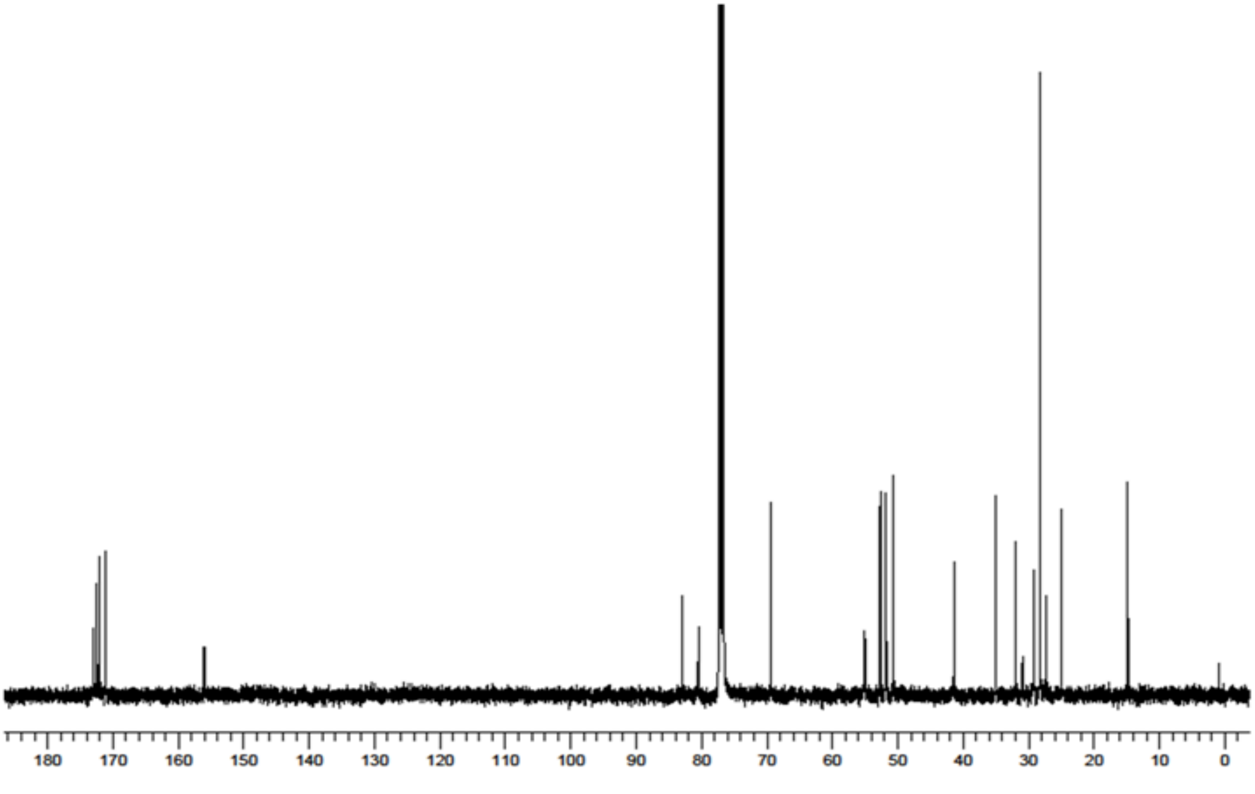
180
170
160
150
140
130
120
110
100
90
80
70
60
50
40
30
20
10
0

A1w

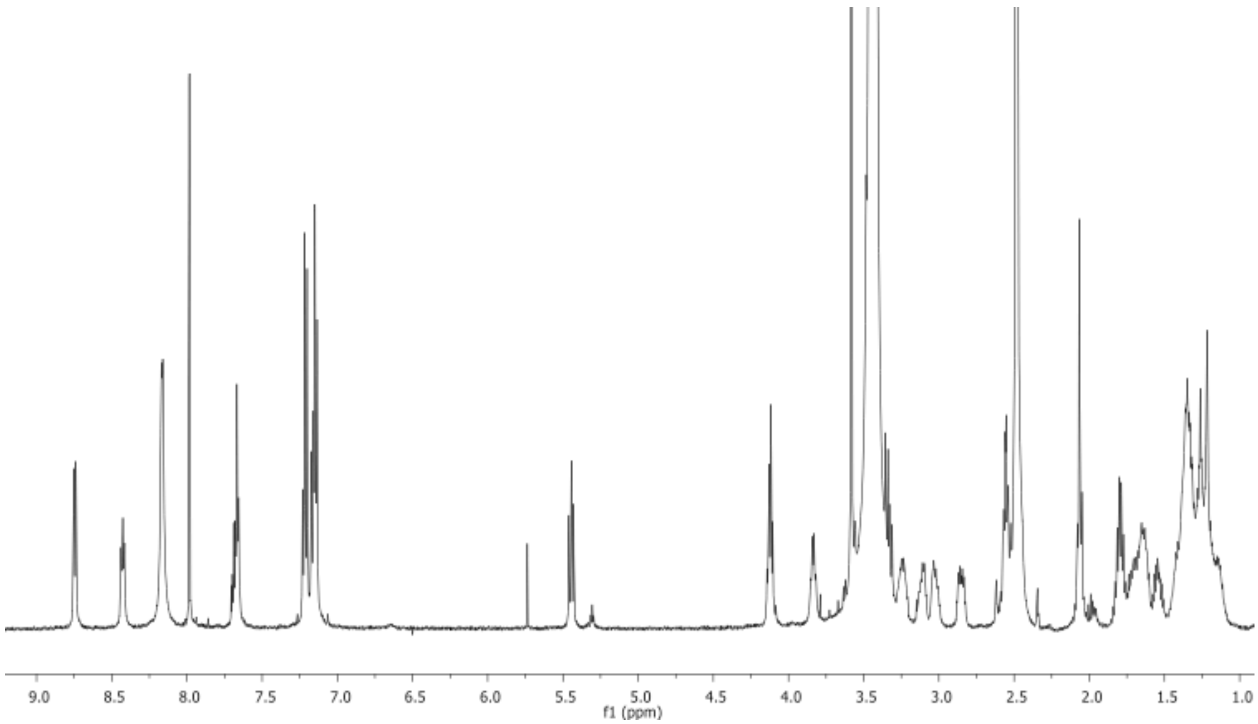

9.0
8.5
8.0
7.5
7.0
6.5
6.0
5.5
5.0
4.5
4.0
3.5
3.0
2.5
2.0
1.5
1.0
f1 (ppm)

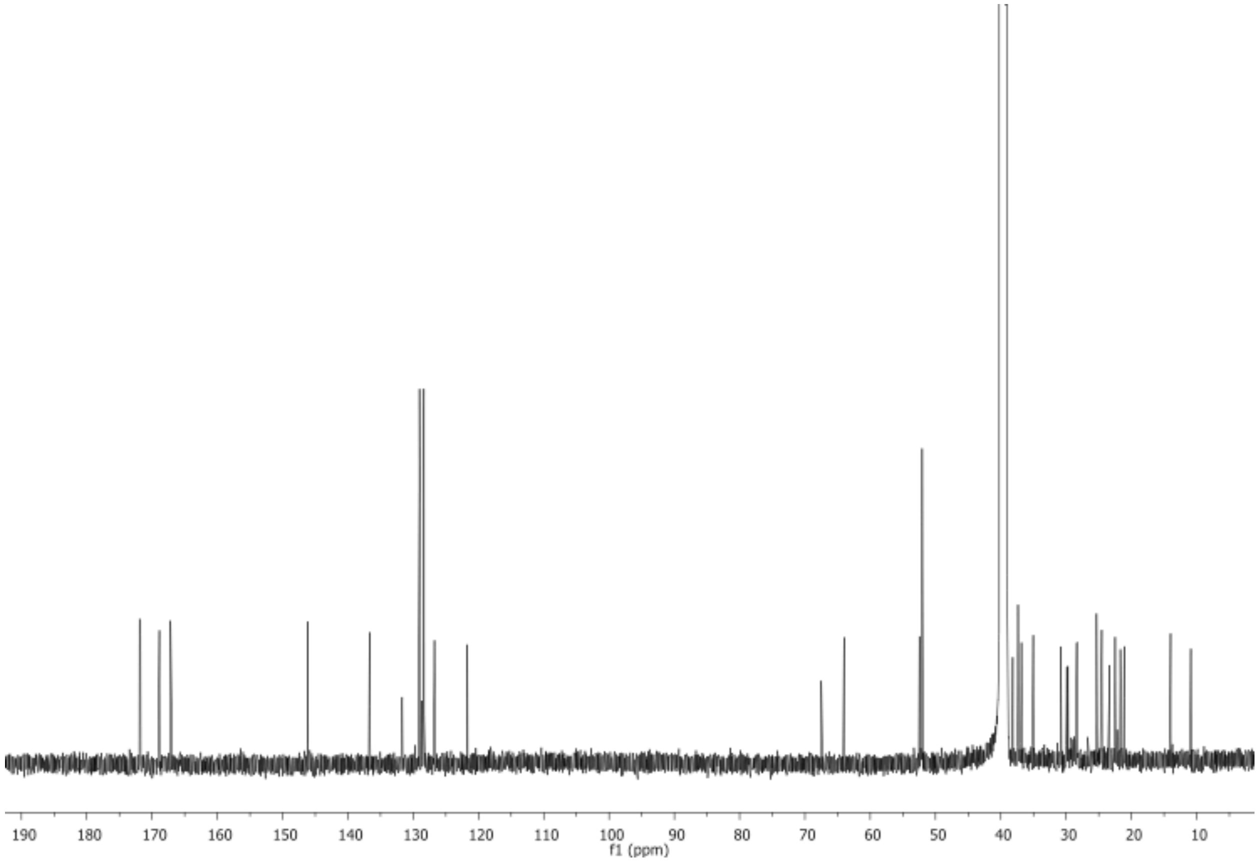

190
180
170
160
150
140
130
120
110
100
90
80
70
60
50
40
30
20
10
f1 (ppm)

B2w

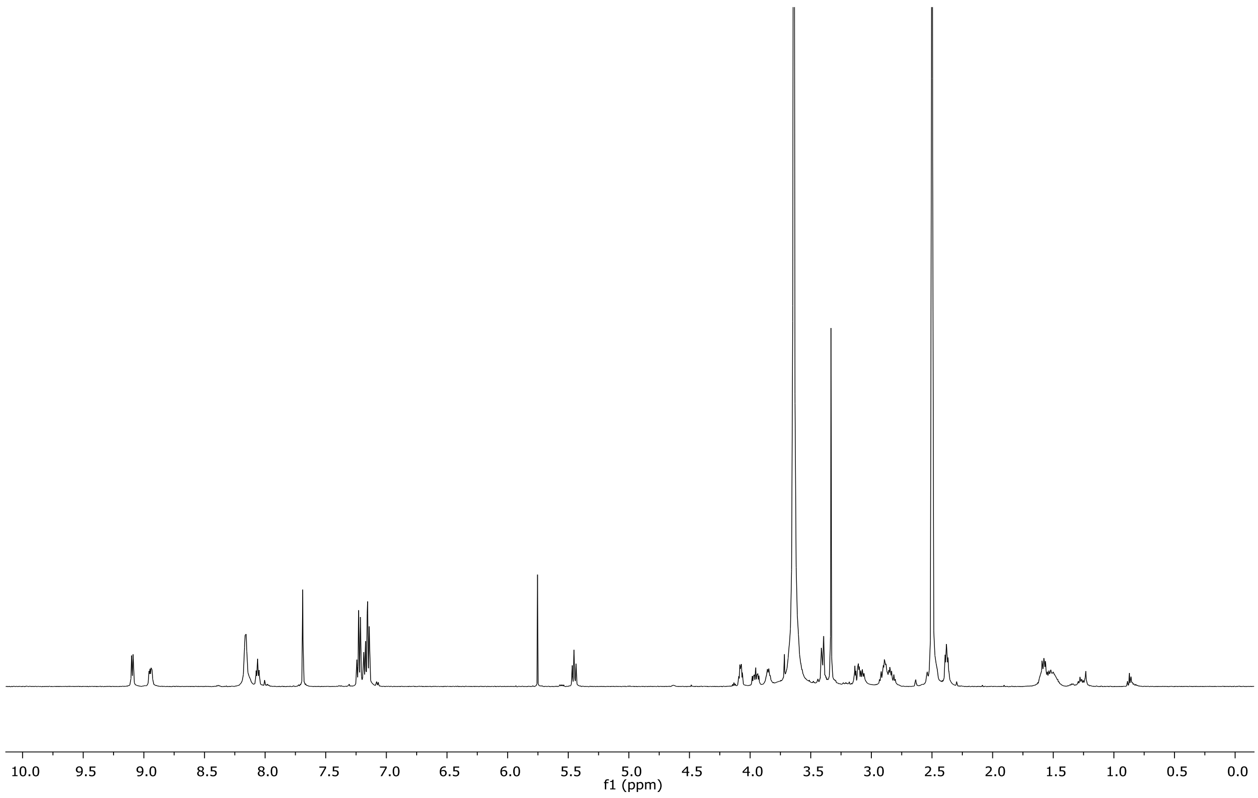
10.0
9.5
9.0
8.5
8.0
7.5
7.0
6.5
6.0
5.5
5.0
4.5
4.0
3.5
3.0
2.5
2.0
1.5
1.0
0.5
0.0
f1 (ppm)

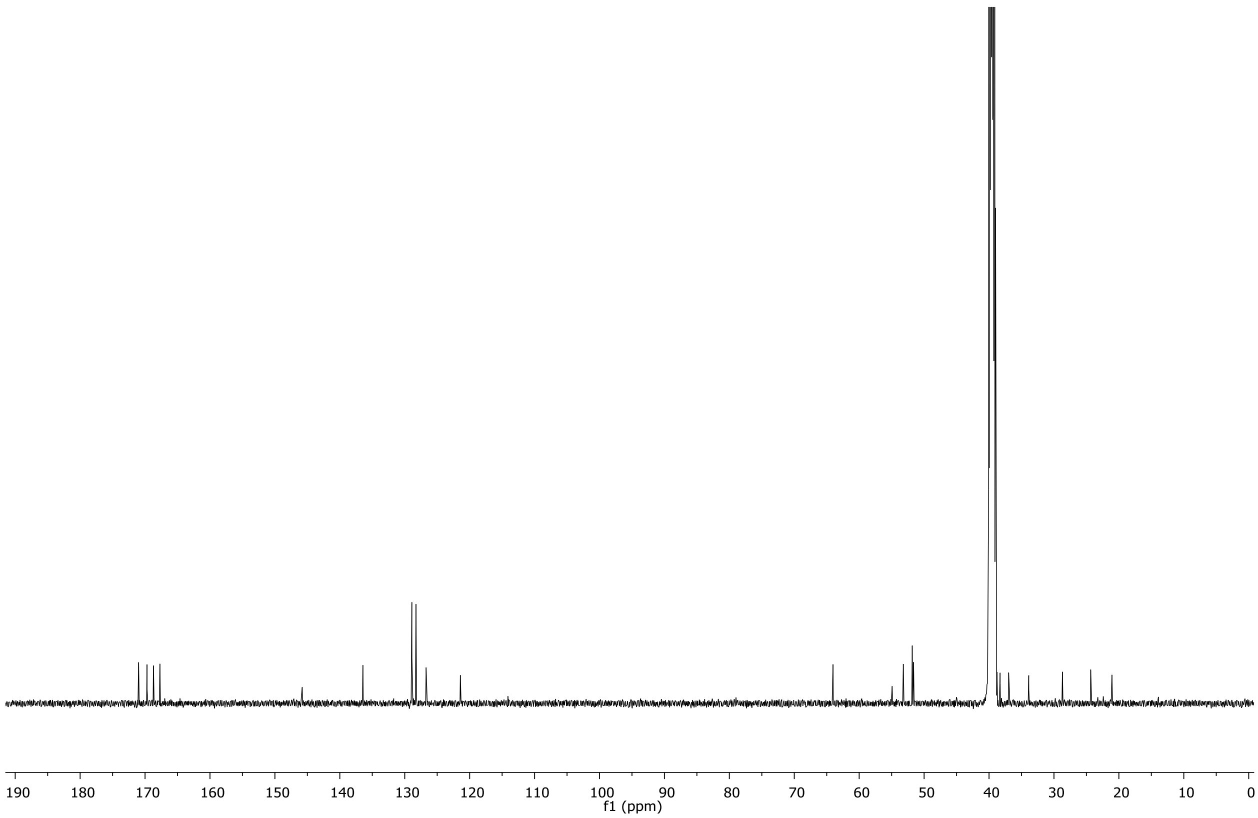
190
180
170
160
150
140
130
120
110
100
90
80
70
60
50
40
30
20
10
0
f1 (ppm)

B3w

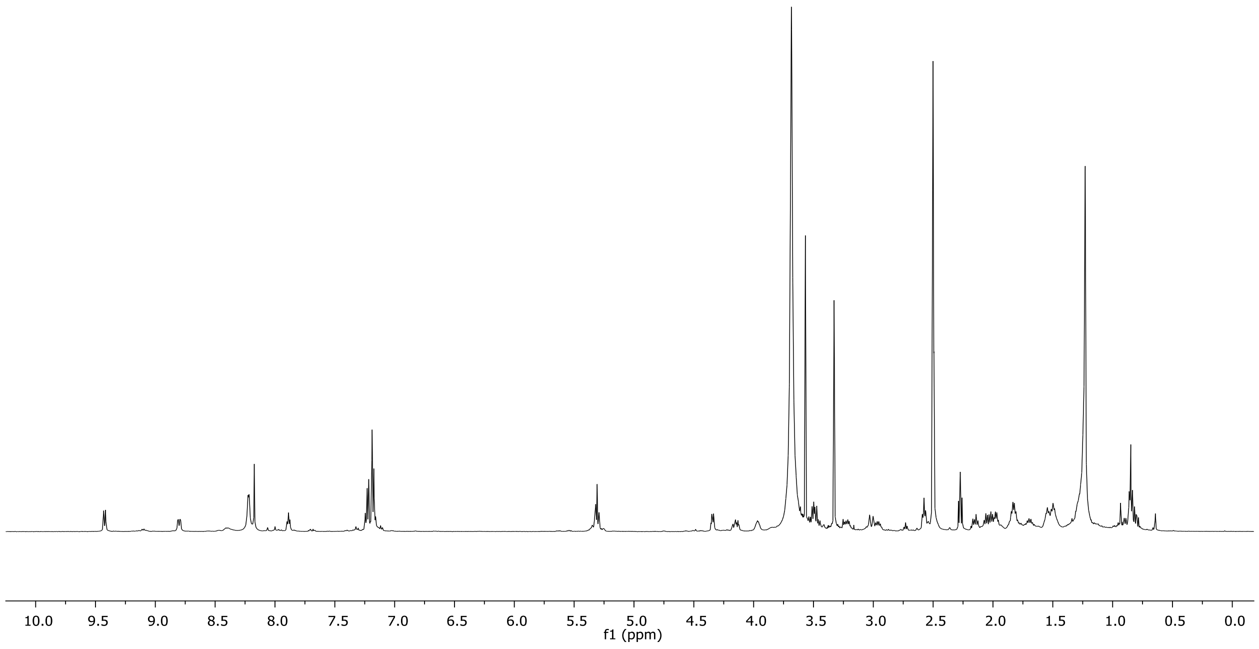
10.0
9.5
9.0
8.5
8.0
7.5
7.0
6.5
6.0
5.5
5.0
4.5
4.0
3.5
3.0
2.5
2.0
1.5
1.0
0.5
0.0
f1 (ppm)

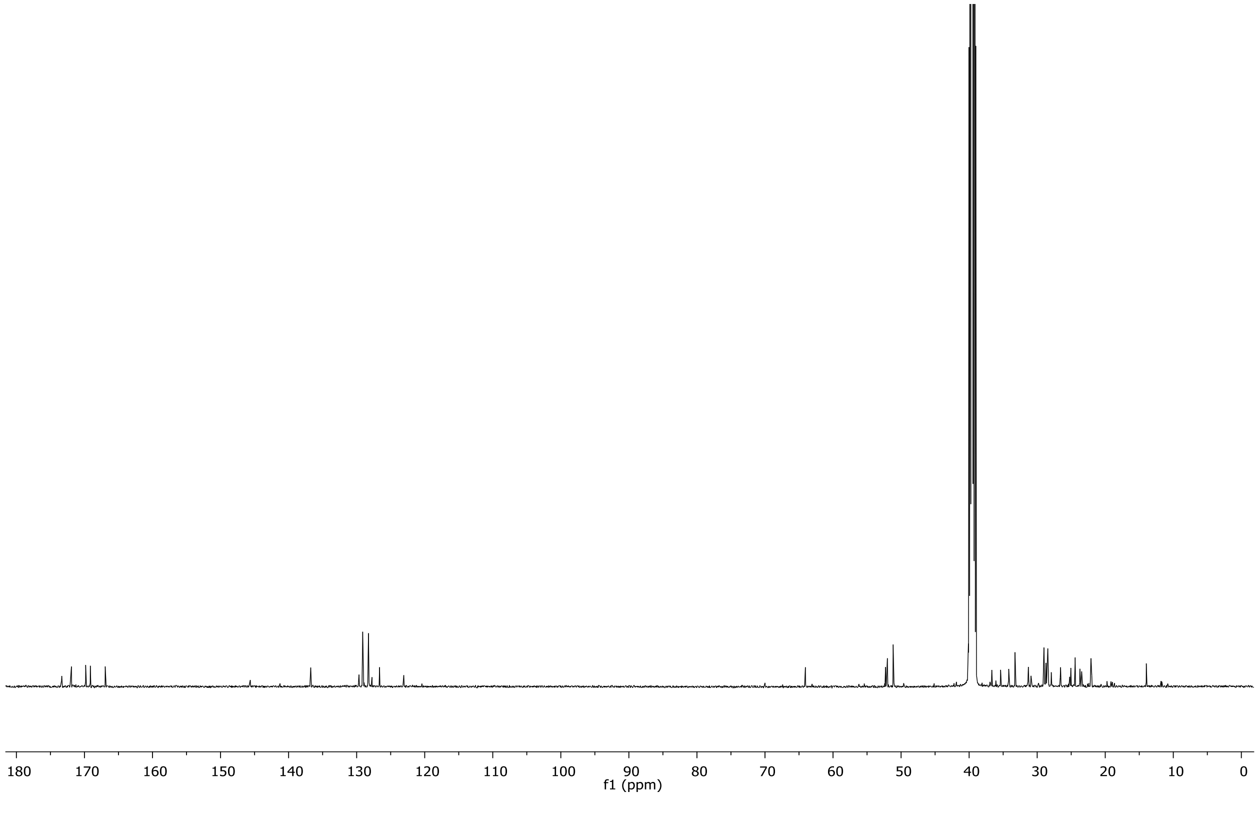
180
170
160
150
140
130
120
110
100
90
80
70
60
50
40
30
20
10
0
f1 (ppm)

B5w

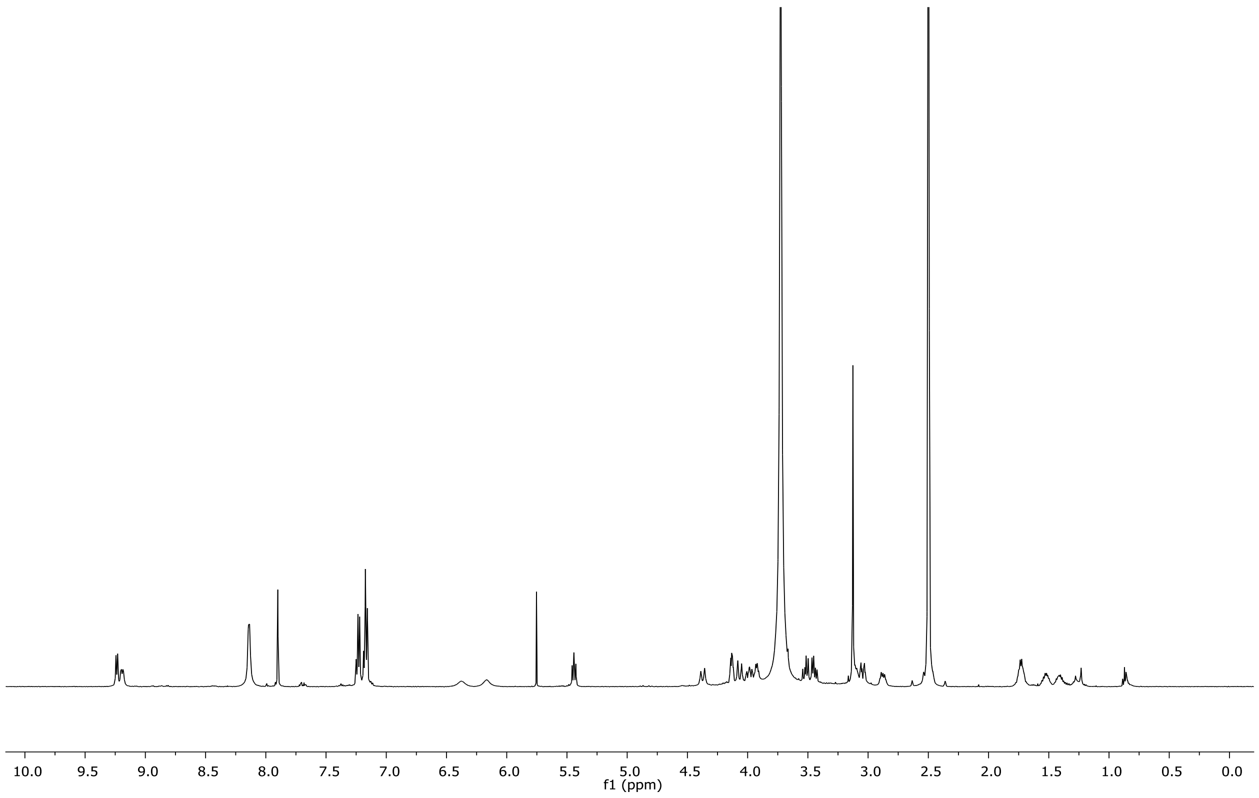
10.0
9.5
9.0
8.5
8.0
7.5
7.0
6.5
6.0
5.5
5.0
4.5
4.0
3.5
3.0
2.5
2.0
1.5
1.0
0.5
0.0
f1 (ppm)

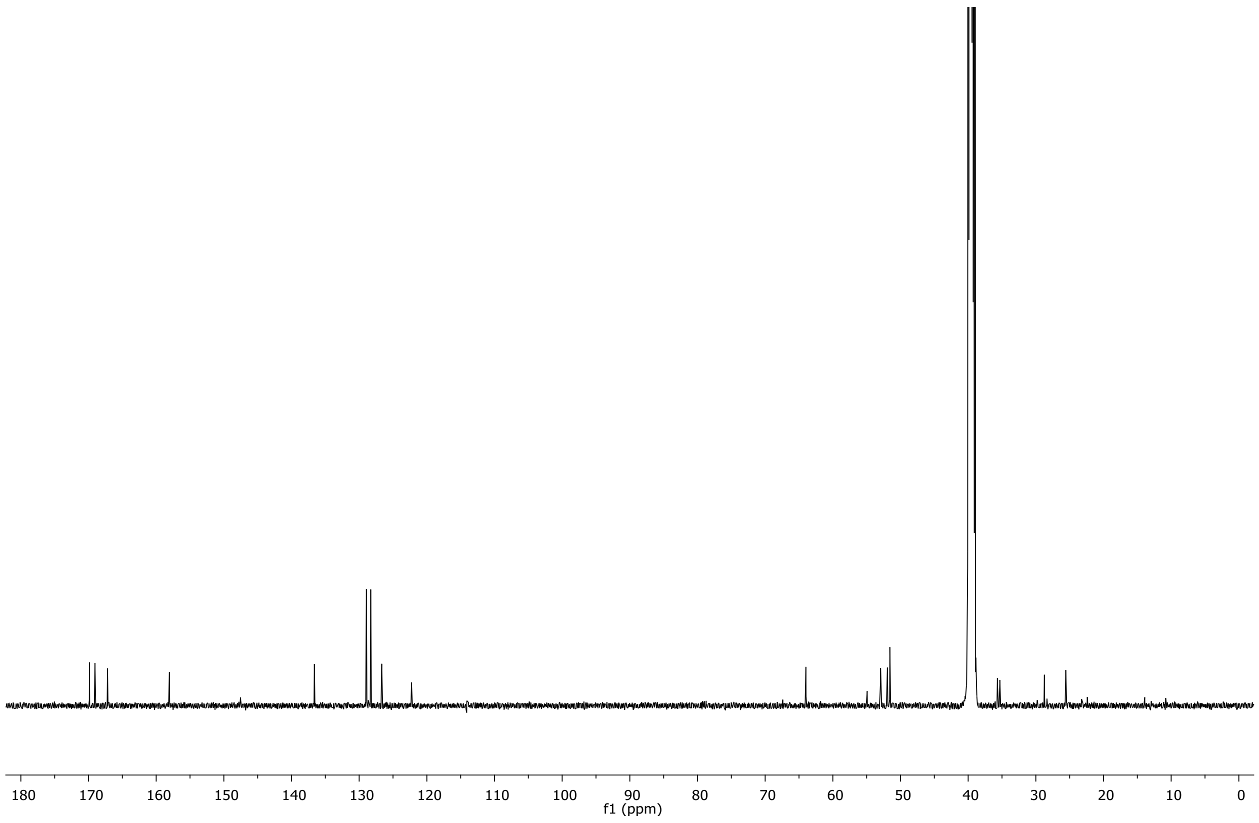
180
170
160
150
140
130
120
110
100
90
80
70
60
50
40
30
20
10
0
f1 (ppm)

B7w

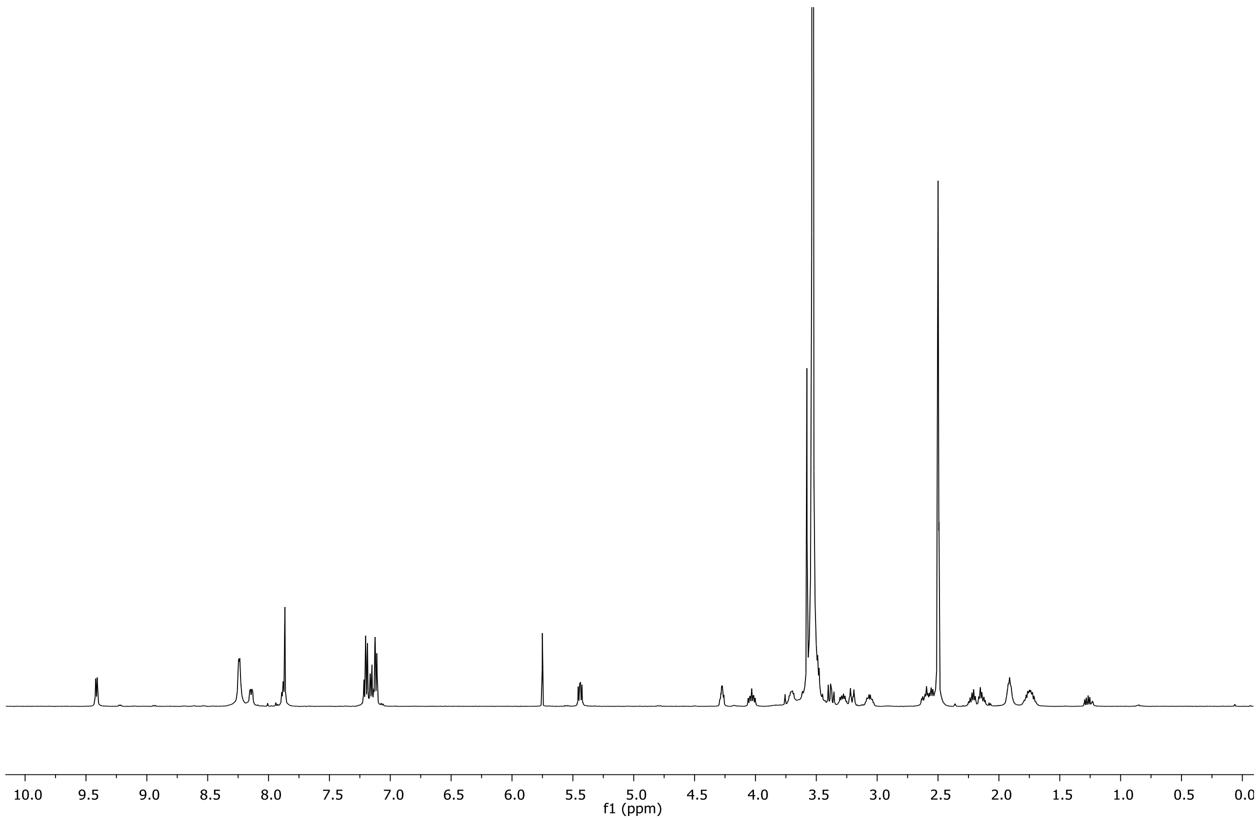
10.0
9.5
9.0
8.5
8.0
7.5
7.0
6.5
6.0
5.5
5.0
4.5
4.0
3.5
3.0
2.5
2.0
1.5
1.0
0.5
0.0
f1 (ppm)

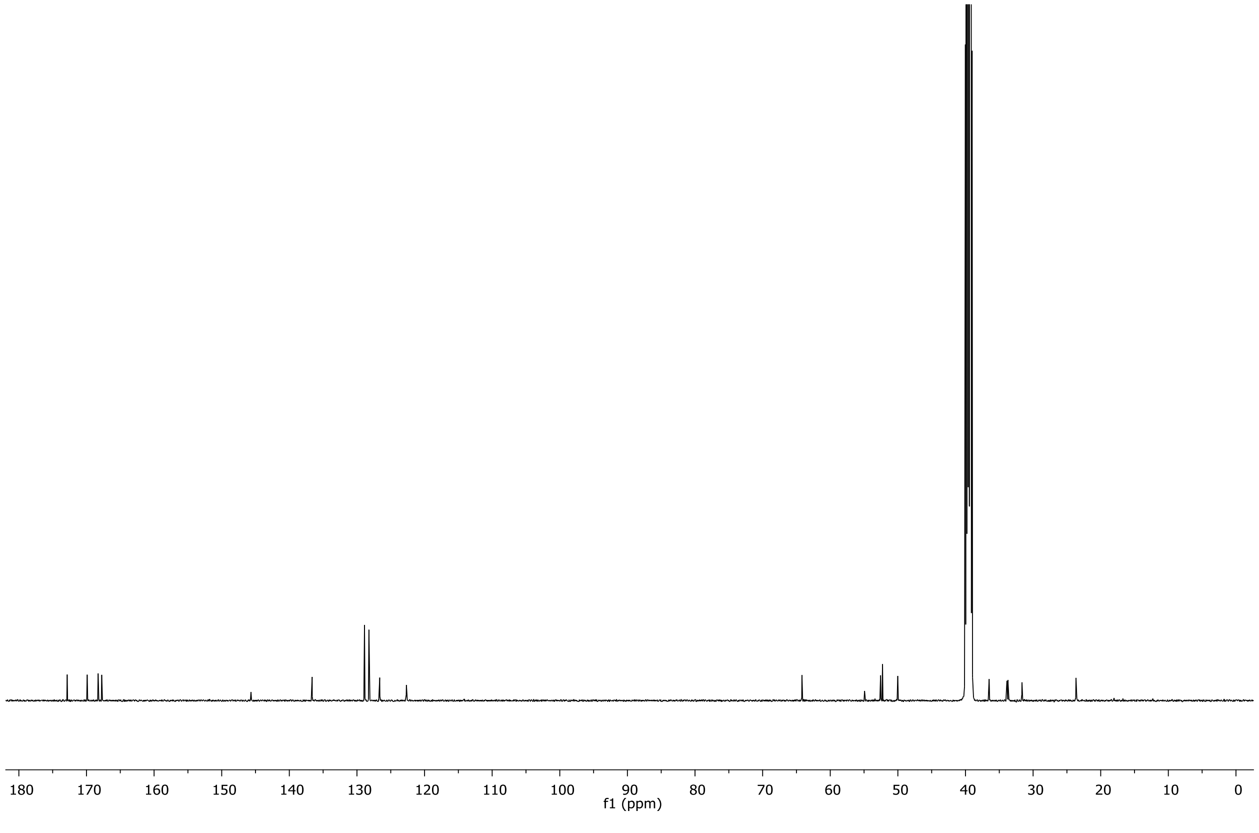
180
170
160
150
140
130
120
110
100
90
80
70
60
50
40
30
20
10
0
f1 (ppm)

B8w

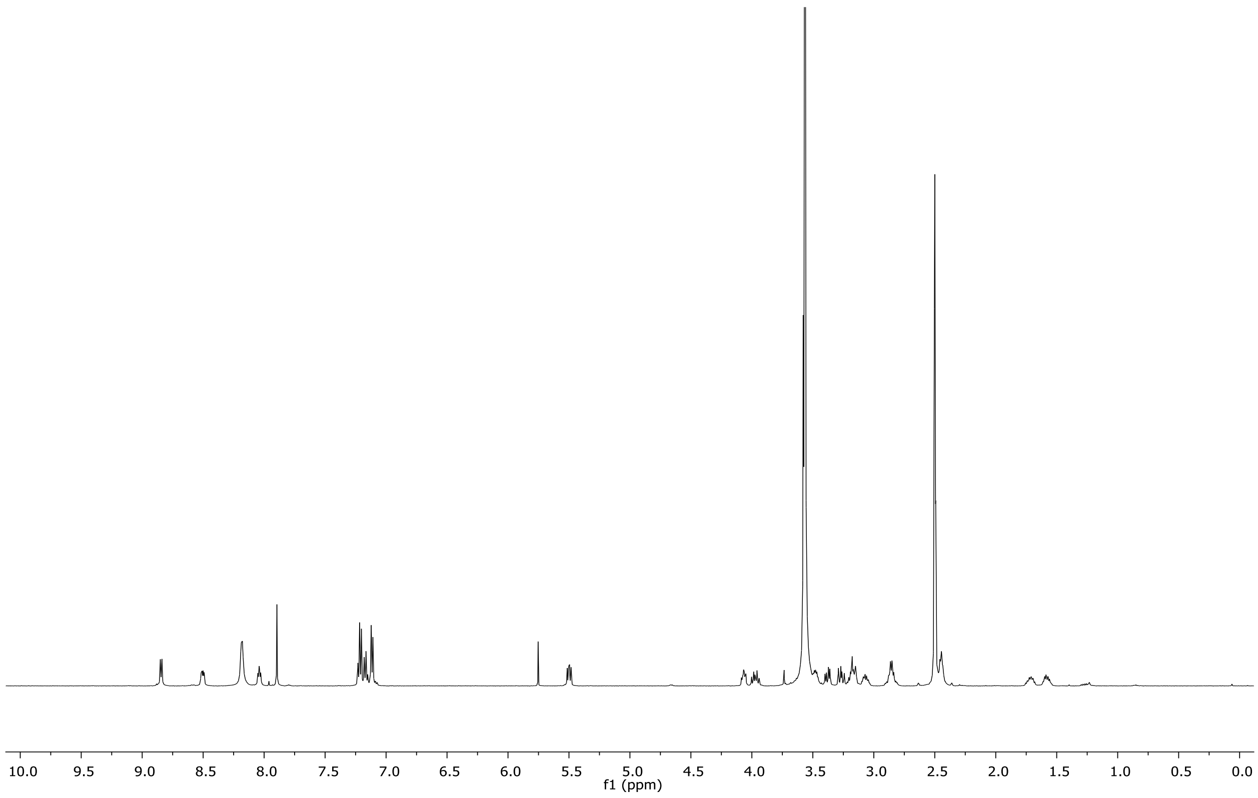
10.0
9.5
9.0
8.5
8.0
7.5
7.0
6.5
6.0
5.5
5.0
4.5
4.0
3.5
3.0
2.5
2.0
1.5
1.0
0.5
0.0
f1 (ppm)

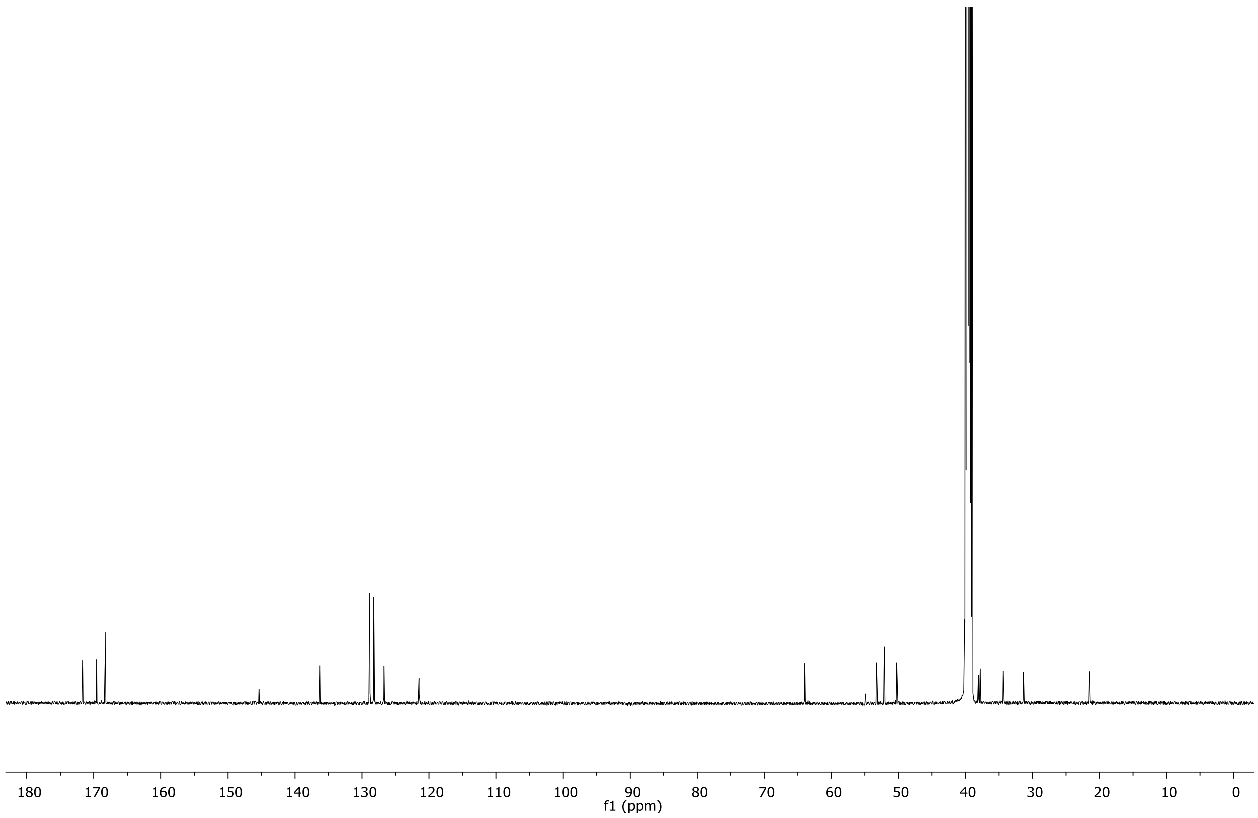
180
170
160
150
140
130
120
110
100
90
80
70
60
50
40
30
20
10
0
f1 (ppm)

B10w

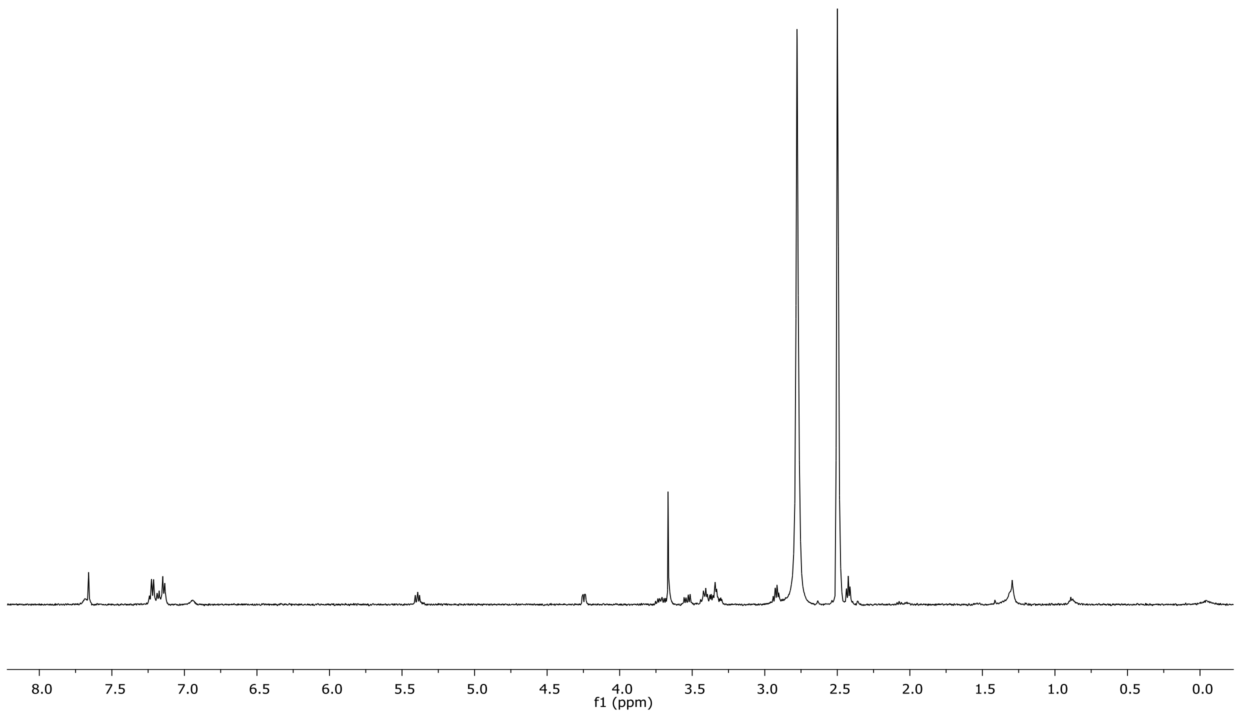
8.0
7.5
7.0
6.5
6.0
5.5
5.0
4.5
4.0
3.5
3.0
2.5
2.0
1.5
1.0
0.5
0.0
f1 (ppm)

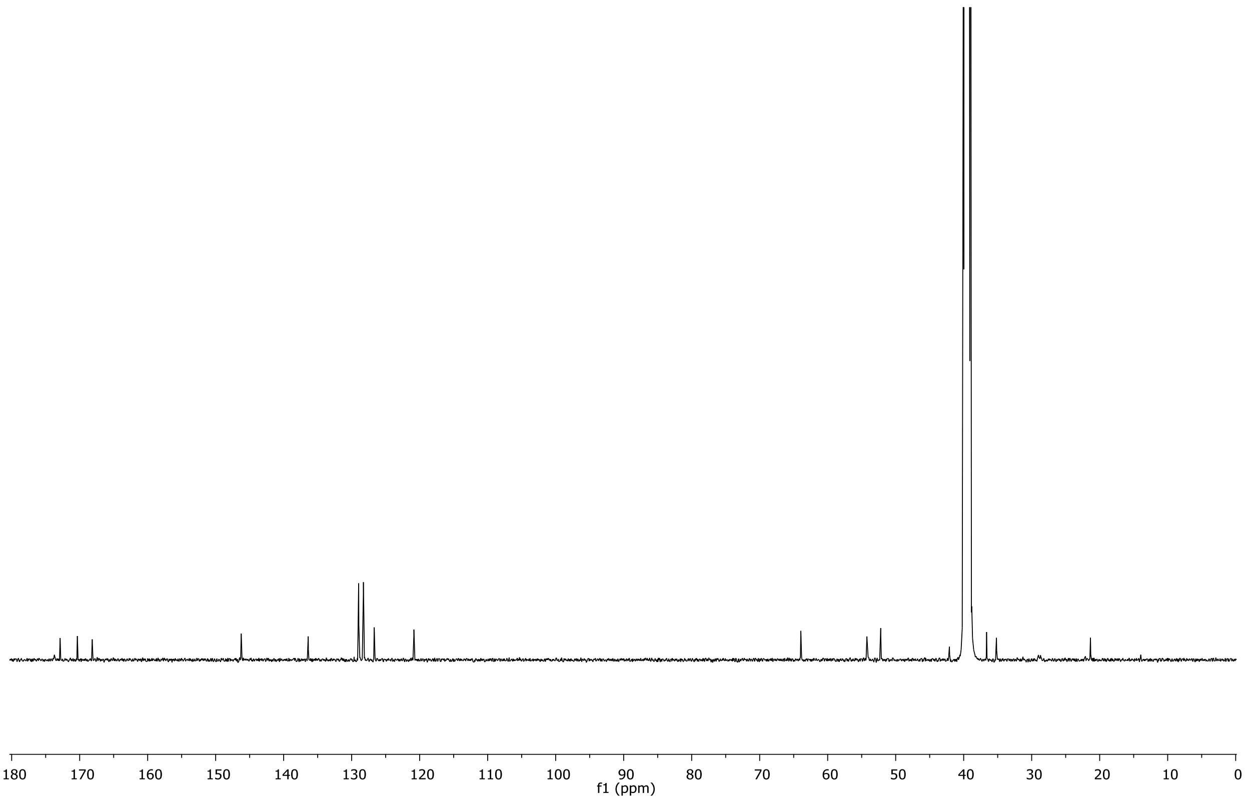
180
170
160
150
140
130
120
110
100
90
80
70
60
50
40
30
20
10
0
f1 (ppm)

B11w

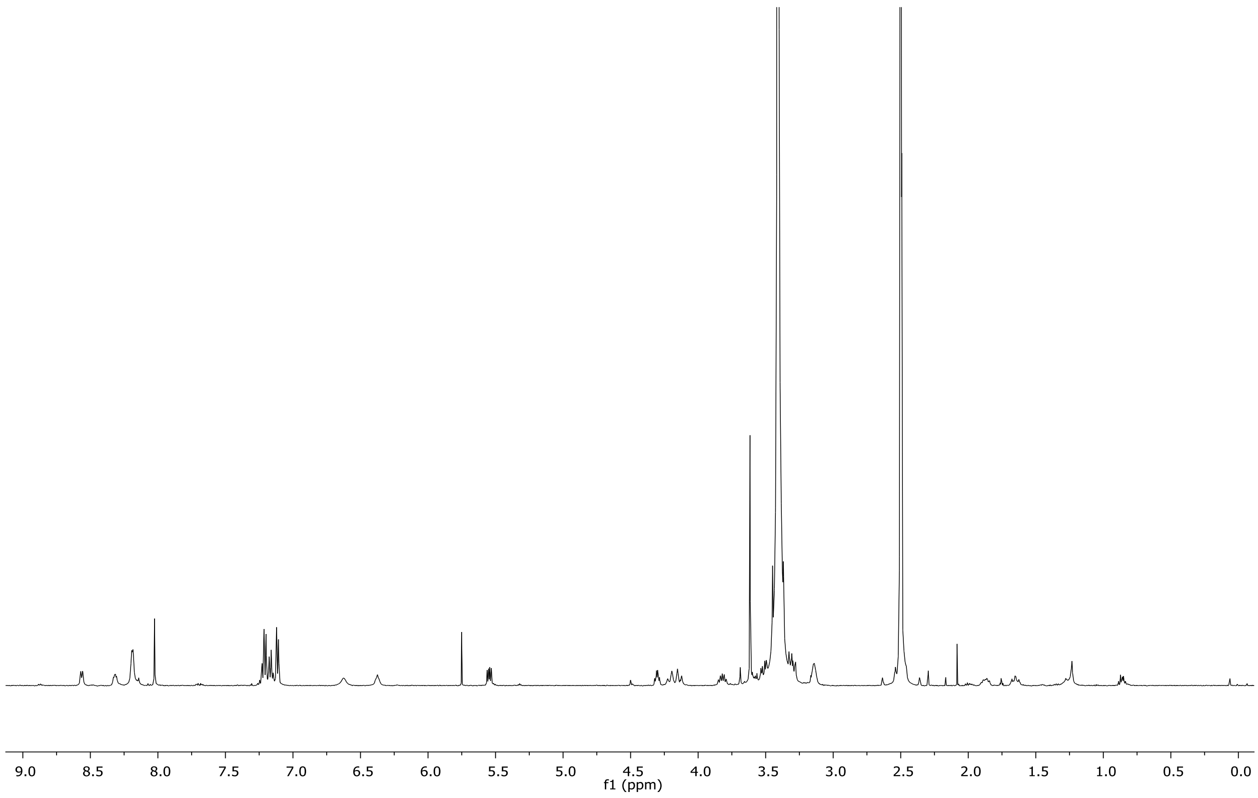
9.0
8.5
8.0
7.5
7.0
6.5
6.0
5.5
5.0
4.5
4.0
3.5
3.0
2.5
2.0
1.5
1.0
0.5
0.0
f1 (ppm)

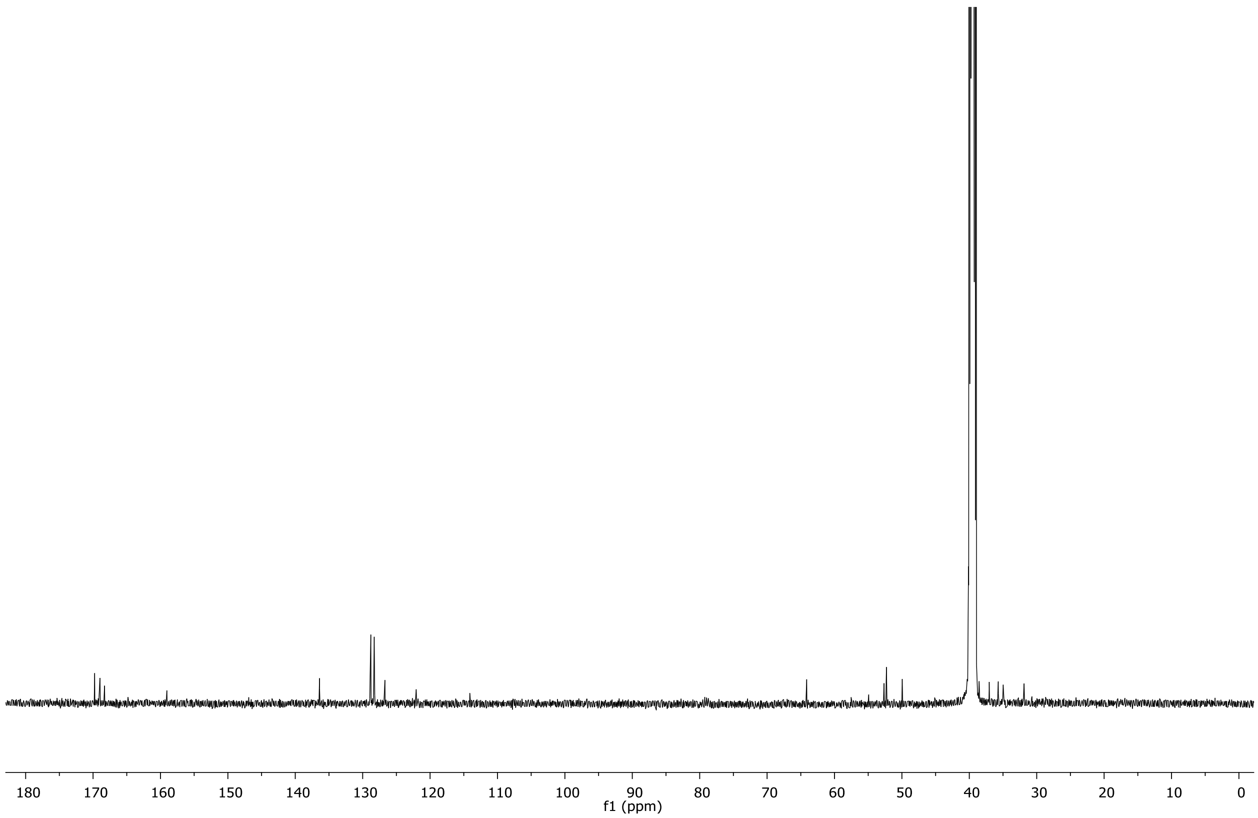
180
170
160
150
140
130
120
110
100
90
80
70
60
50
40
30
20
10
0
f1 (ppm)

B12w

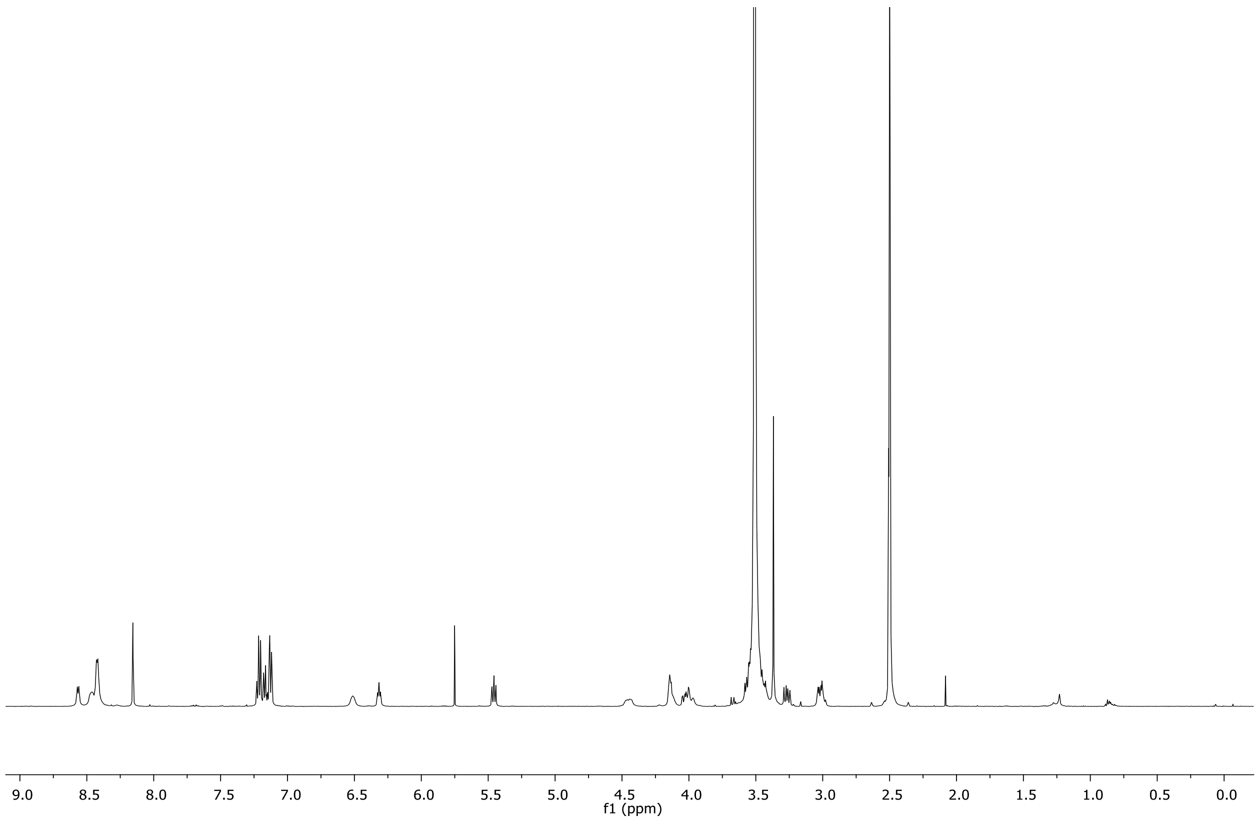

9.0
8.5
8.0
7.5
7.0
6.5
6.0
5.5
5.0
4.5
4.0
3.5
3.0
2.5
2.0
1.5
1.0
0.5
0.0
f1 (ppm)

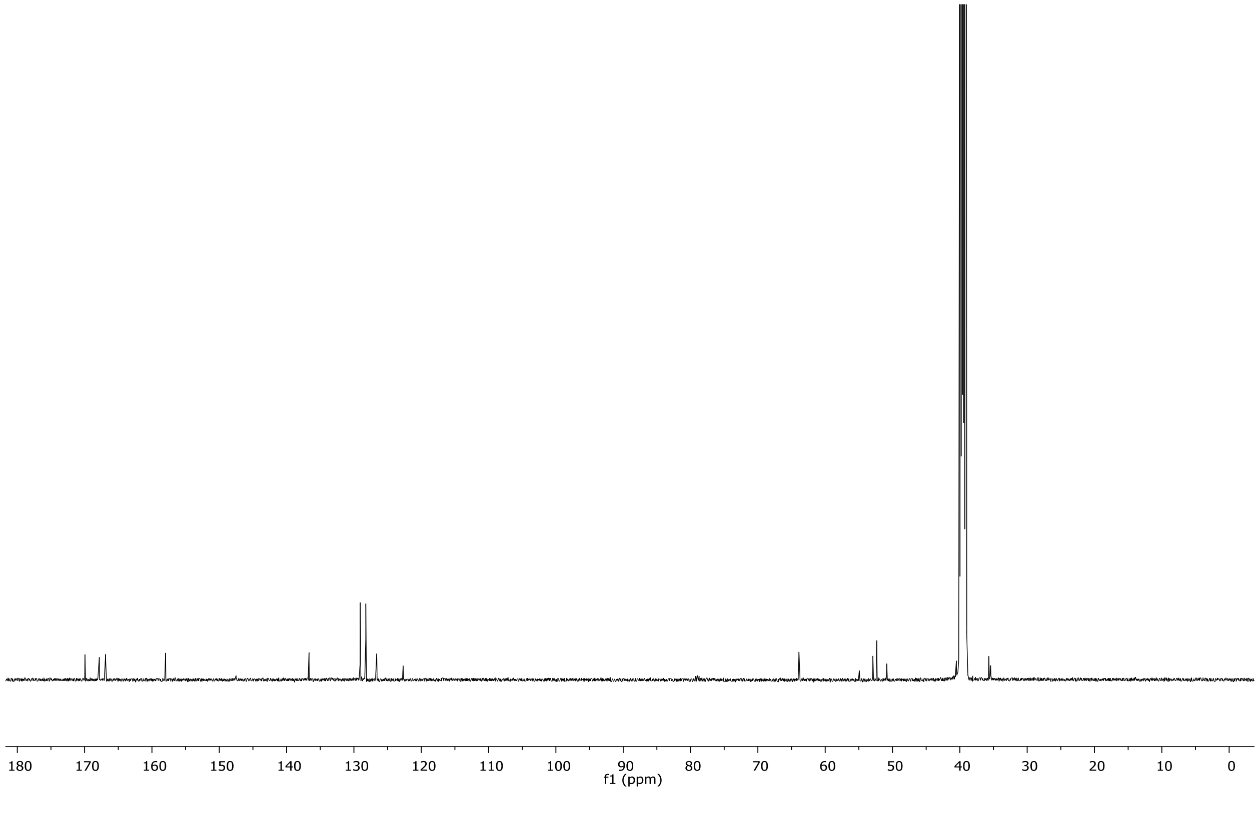

180
170
160
150
140
130
120
110
100
90
80
70
60
50
40
30
20
10
0
f1 (ppm)

B14w

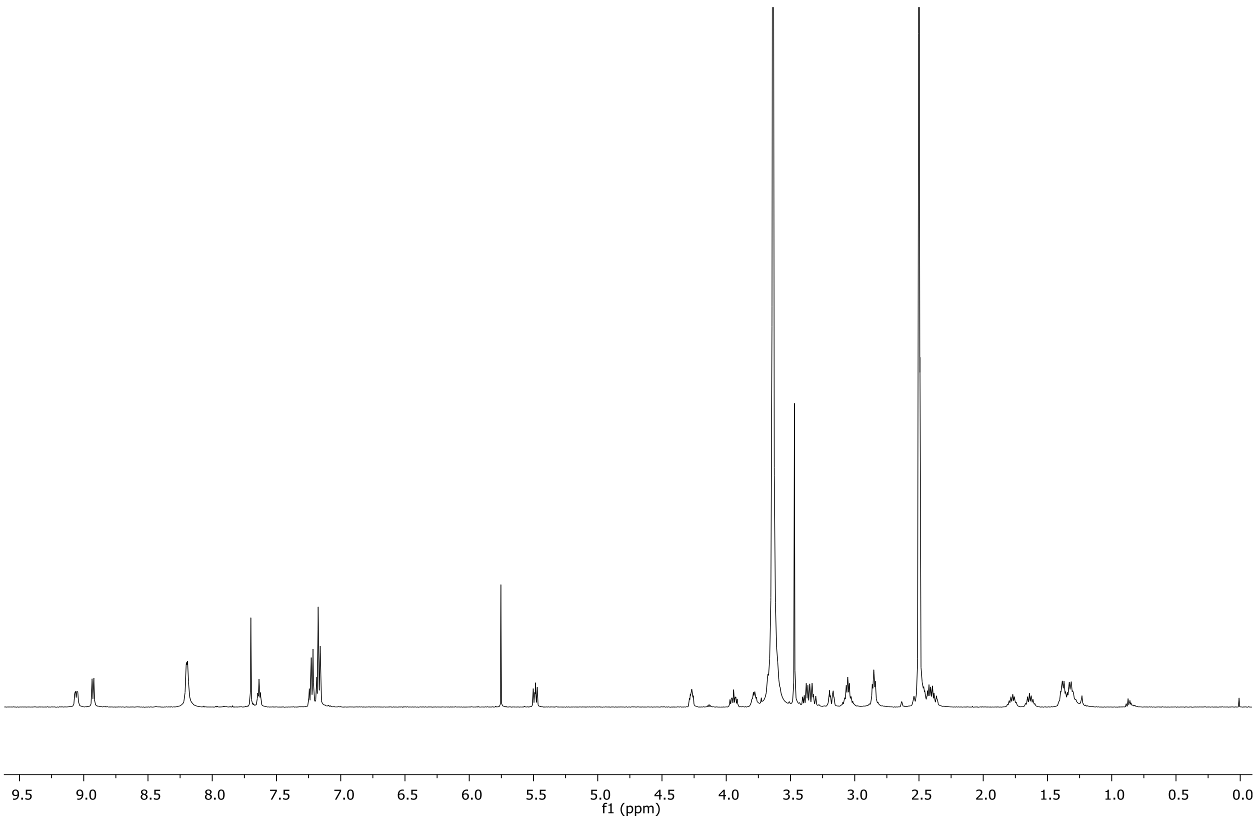
9.5
9.0
8.5
8.0
7.5
7.0
6.5
6.0
5.5
5.0
4.5
4.0
3.5
3.0
2.5
2.0
1.5
1.0
0.5
0.0
f1 (ppm)

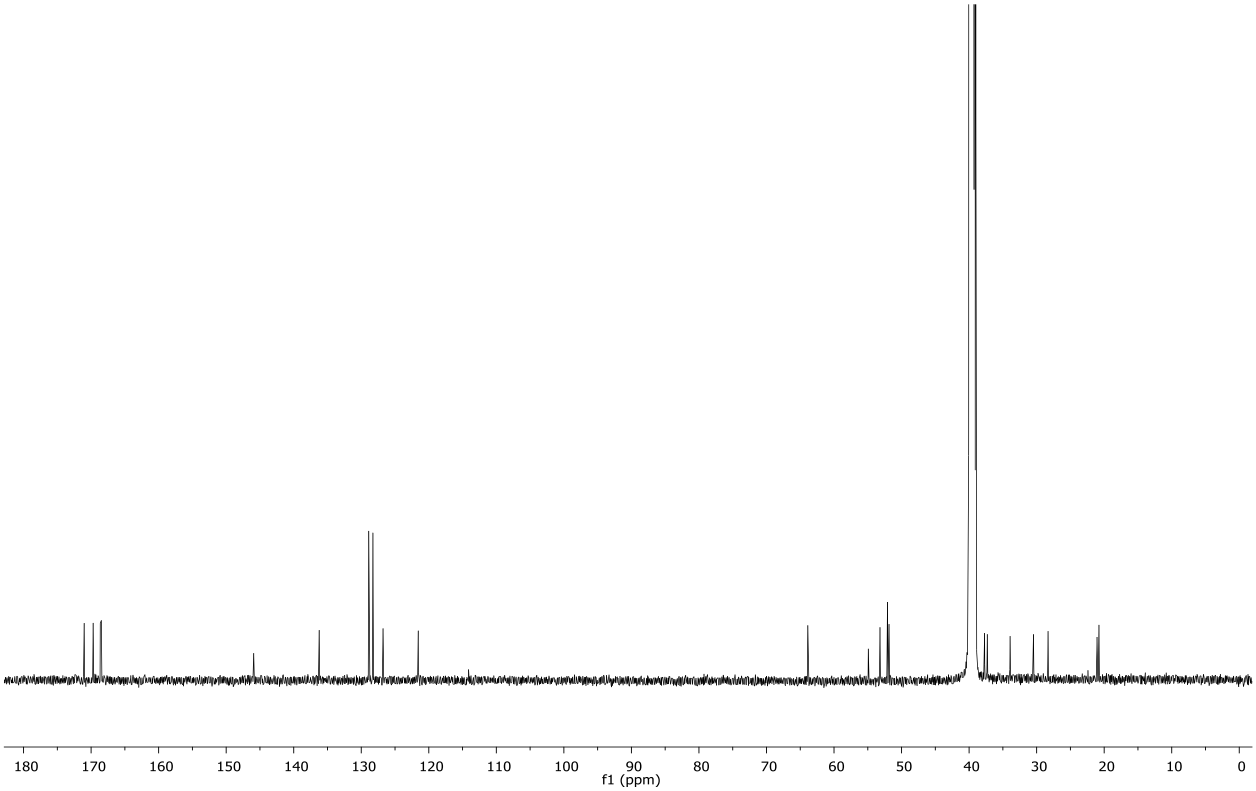
180
170
160
150
140
130
120
110
100
90
80
70
60
50
40
30
20
10
0
f1 (ppm)

G4w

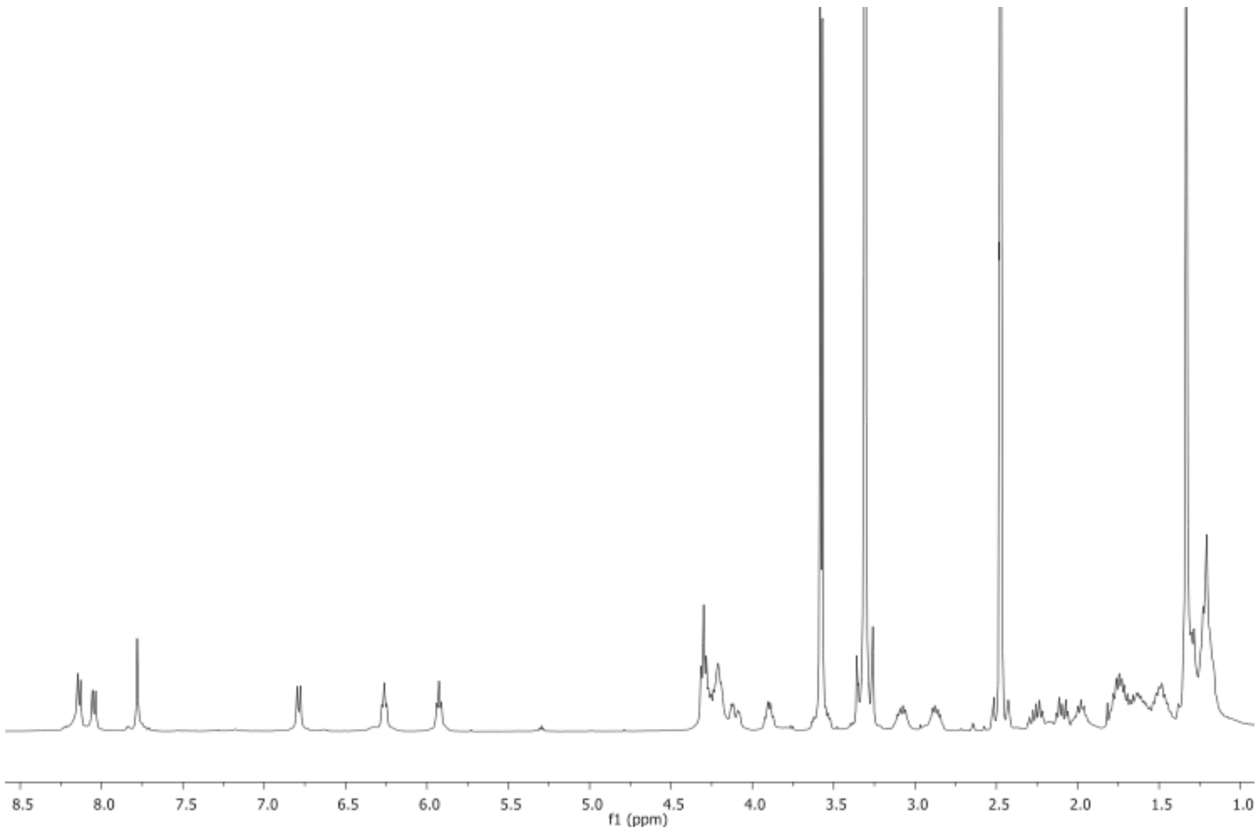
8.5
8.0
7.5
7.0
6.5
6.0
5.5
5.0
4.5
4.0
3.5
3.0
2.5
2.0
1.5
1.0
f1 (ppm)

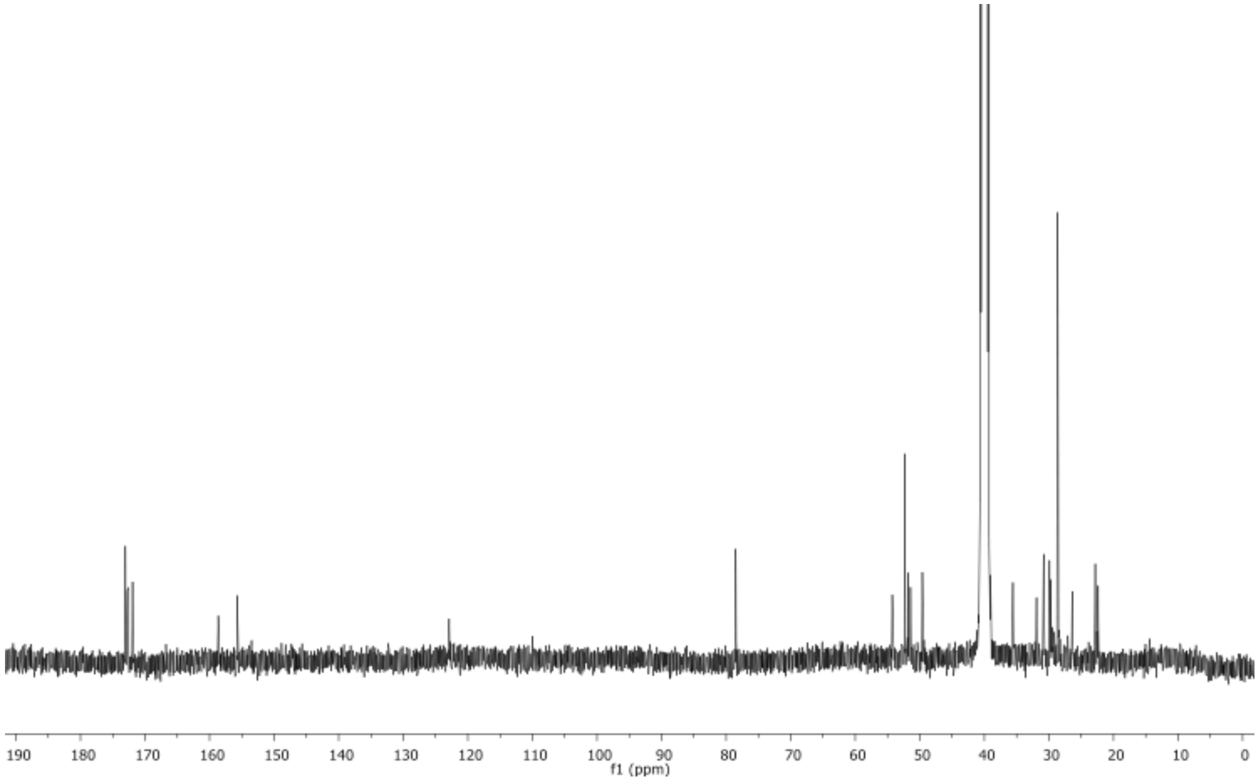
190
180
170
160
150
140
130
120
110
100
90
80
70
60
50
40
30
20
10
0
f1 (ppm)

G5w

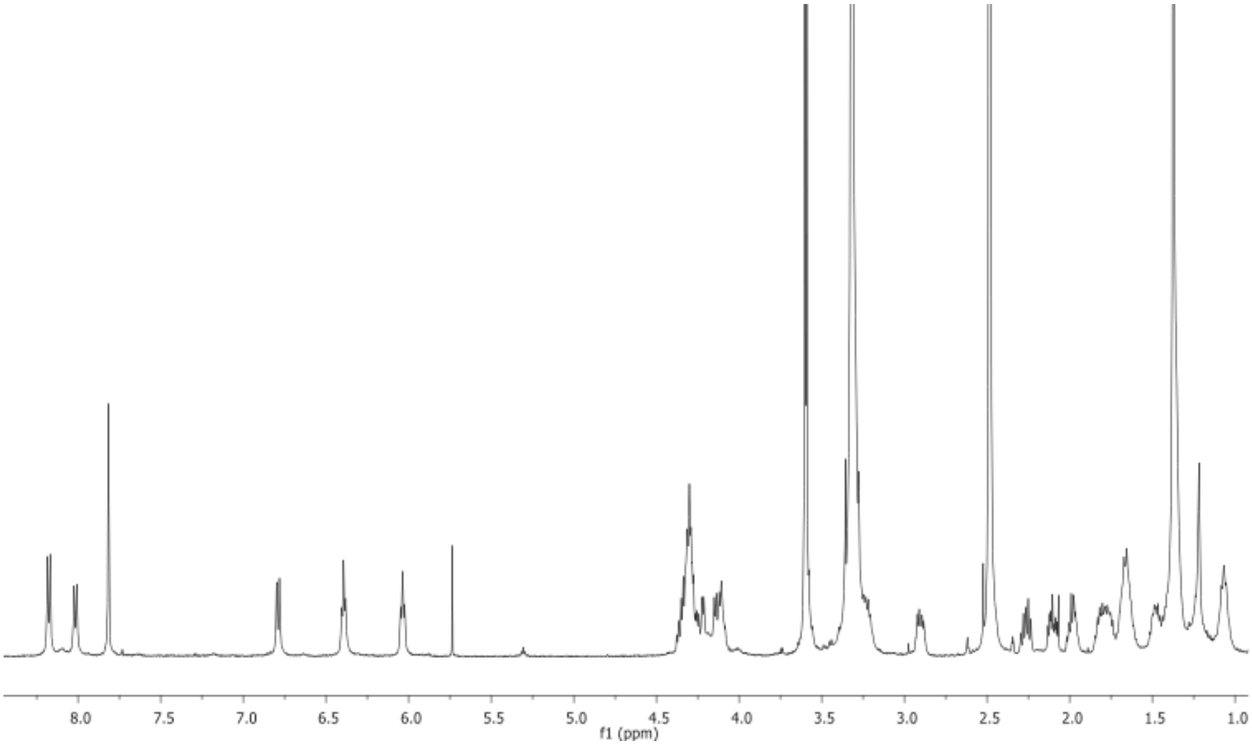

8.0
7.5
7.0
6.5
6.0
5.5
5.0
4.5
4.0
3.5
3.0
2.5
2.0
1.5
1.0
f1 (ppm)

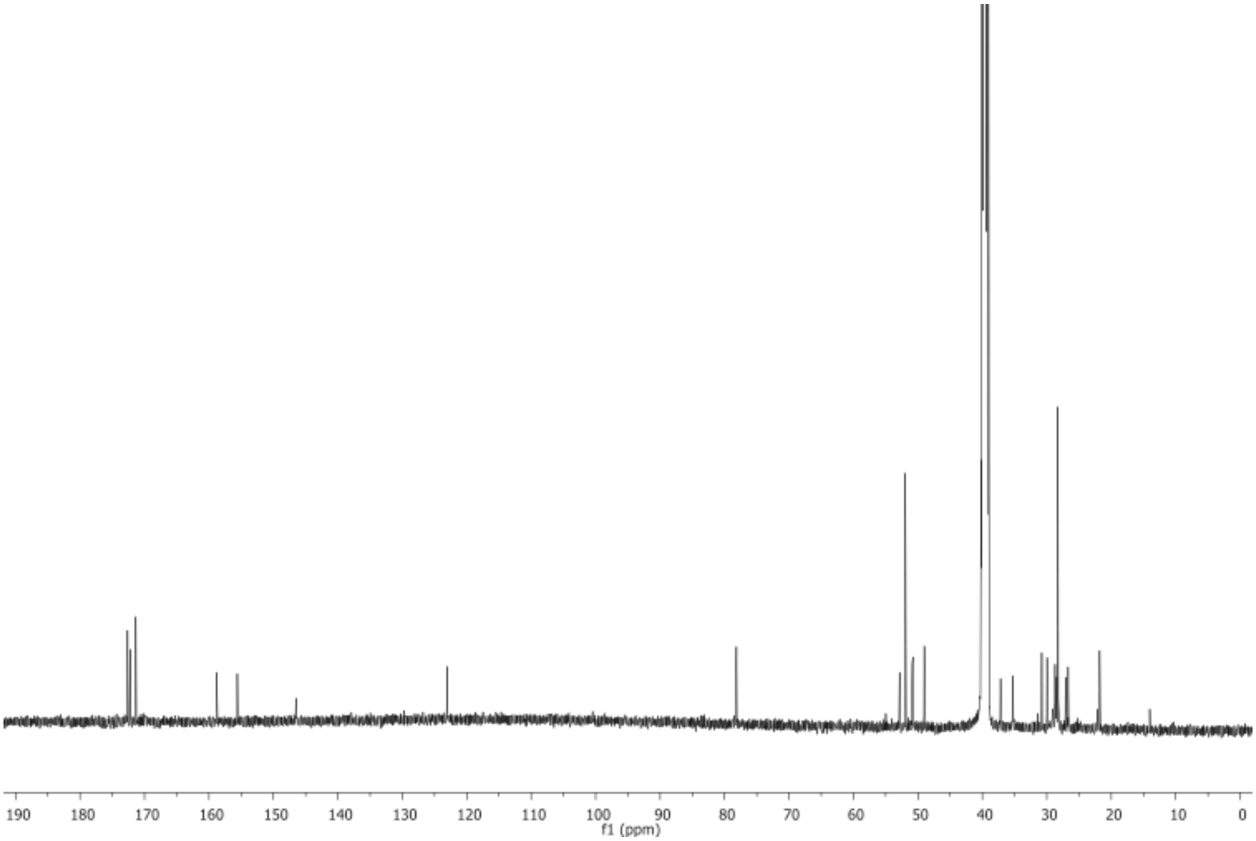

190
180
170
160
150
140
130
120
110
100
90
80
70
60
50
40
30
20
10
0
f1 (ppm)

A6x

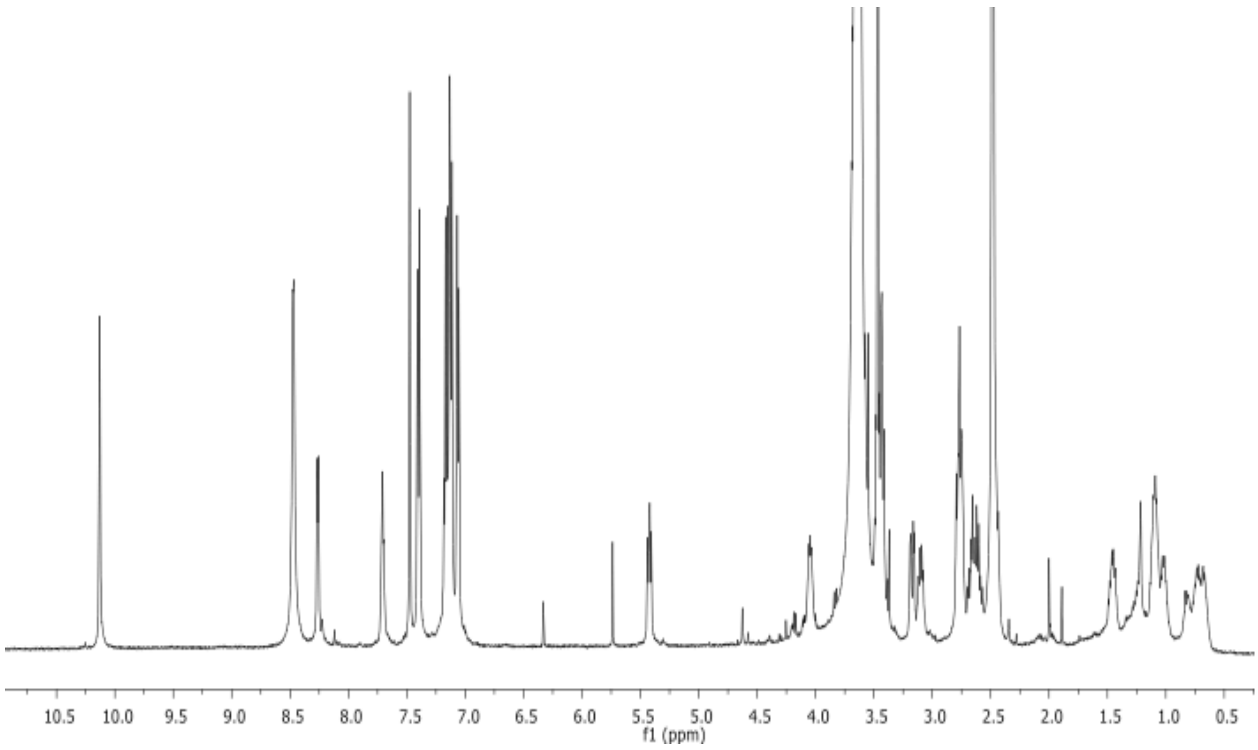
10.5
10.0
9.5
9.0
8.5
8.0
7.5
7.0
6.5
6.0
5.5
5.0
4.5
4.0
3.5
3.0
2.5
2.0
1.5
1.0
0.5
f1 (ppm)

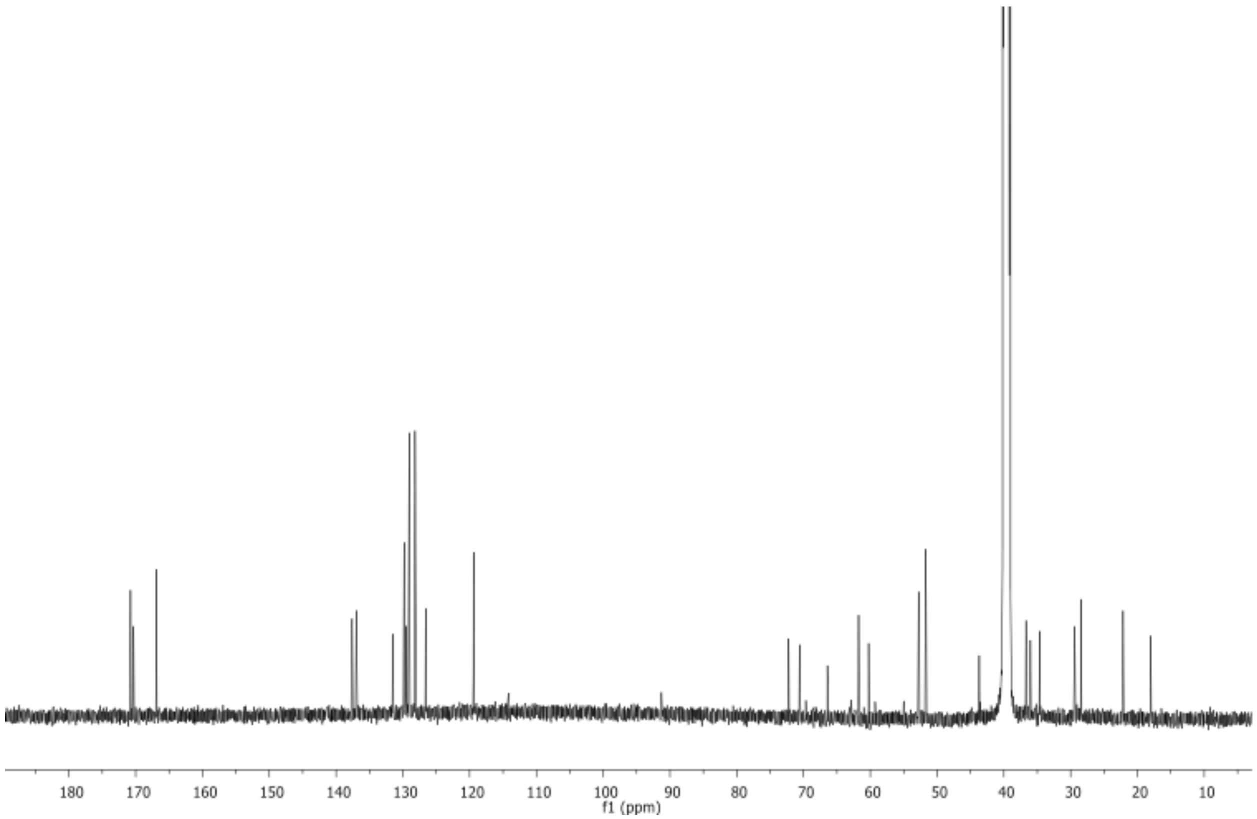
180
170
160
150
140
130
120
110
100
90
80
70
60
50
40
30
20
10
f1 (ppm)

A12x

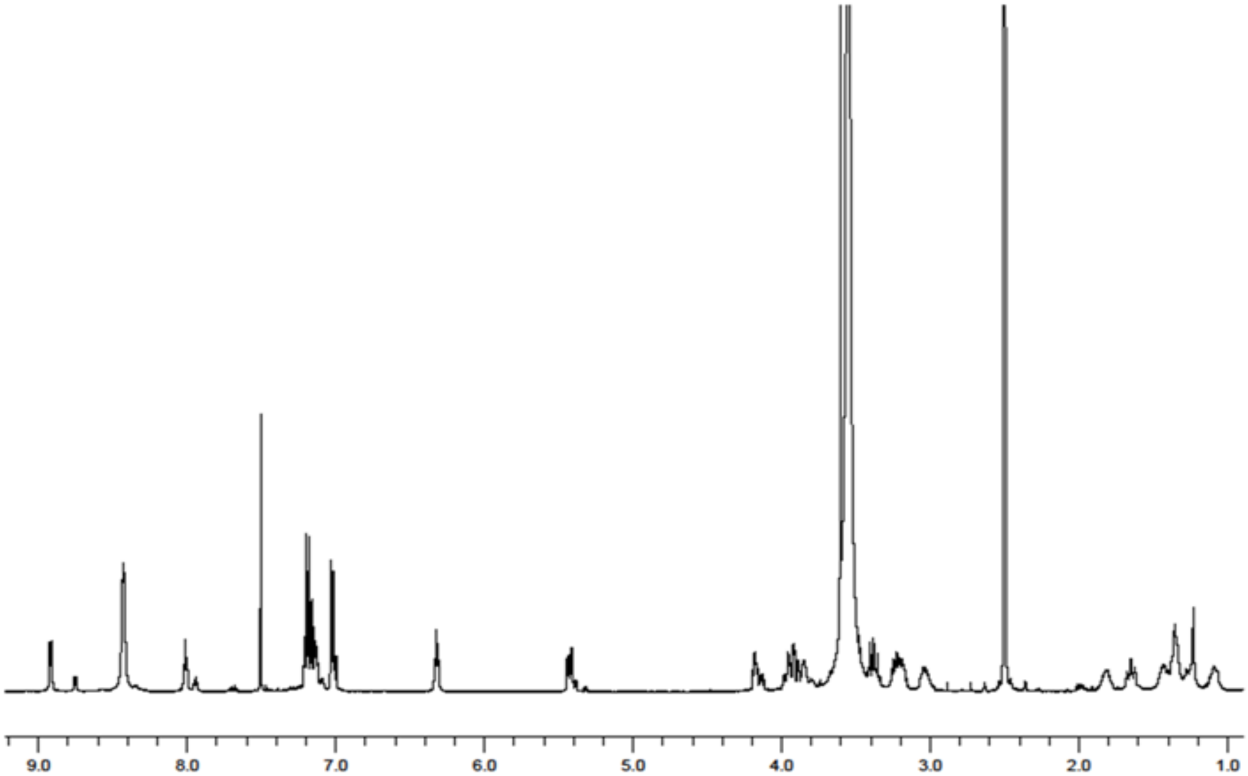
9.0
8.0
7.0
6.0
5.0
4.0
3.0
2.0
1.0

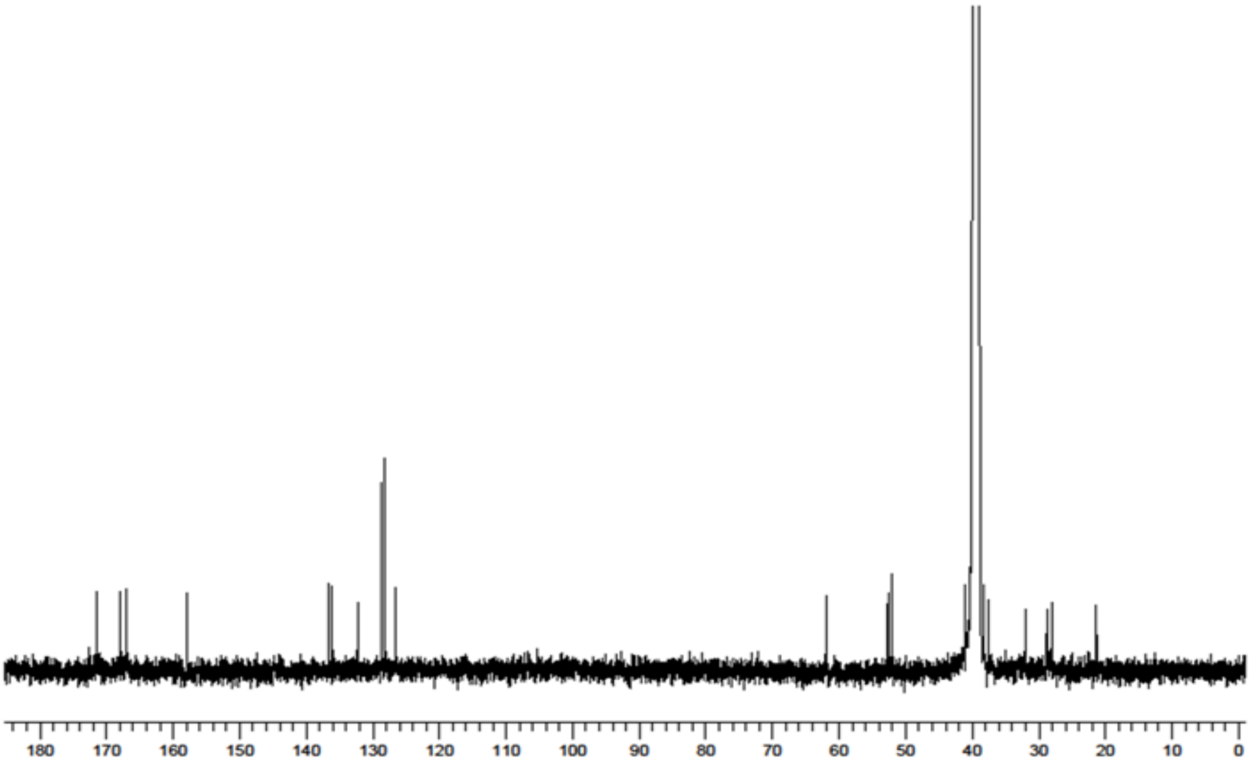
180
170
160
150
140
130
120
110
100
90
80
70
60
50
40
30
20
10
0

B2x

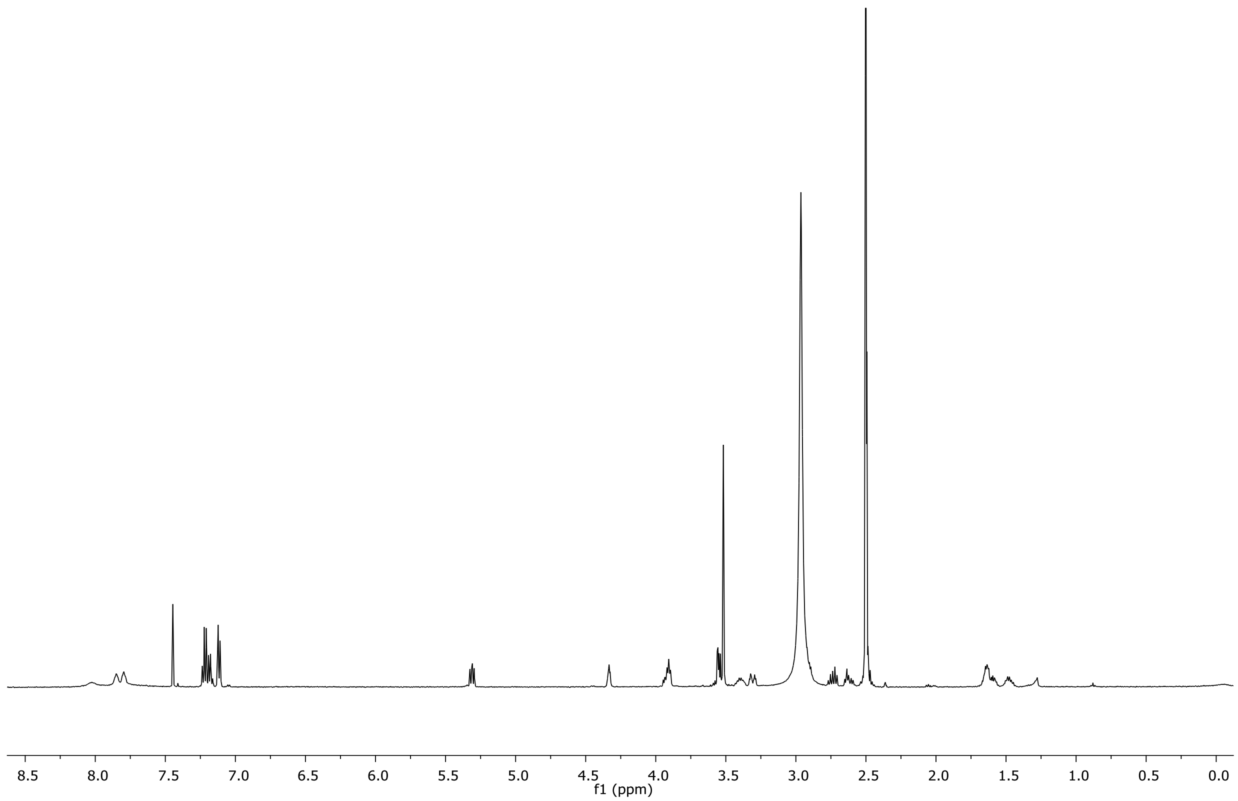
8.5
8.0
7.5
7.0
6.5
6.0
5.5
5.0
4.5
4.0
3.5
3.0
2.5
2.0
1.5
1.0
0.5
0.0
f1 (ppm)

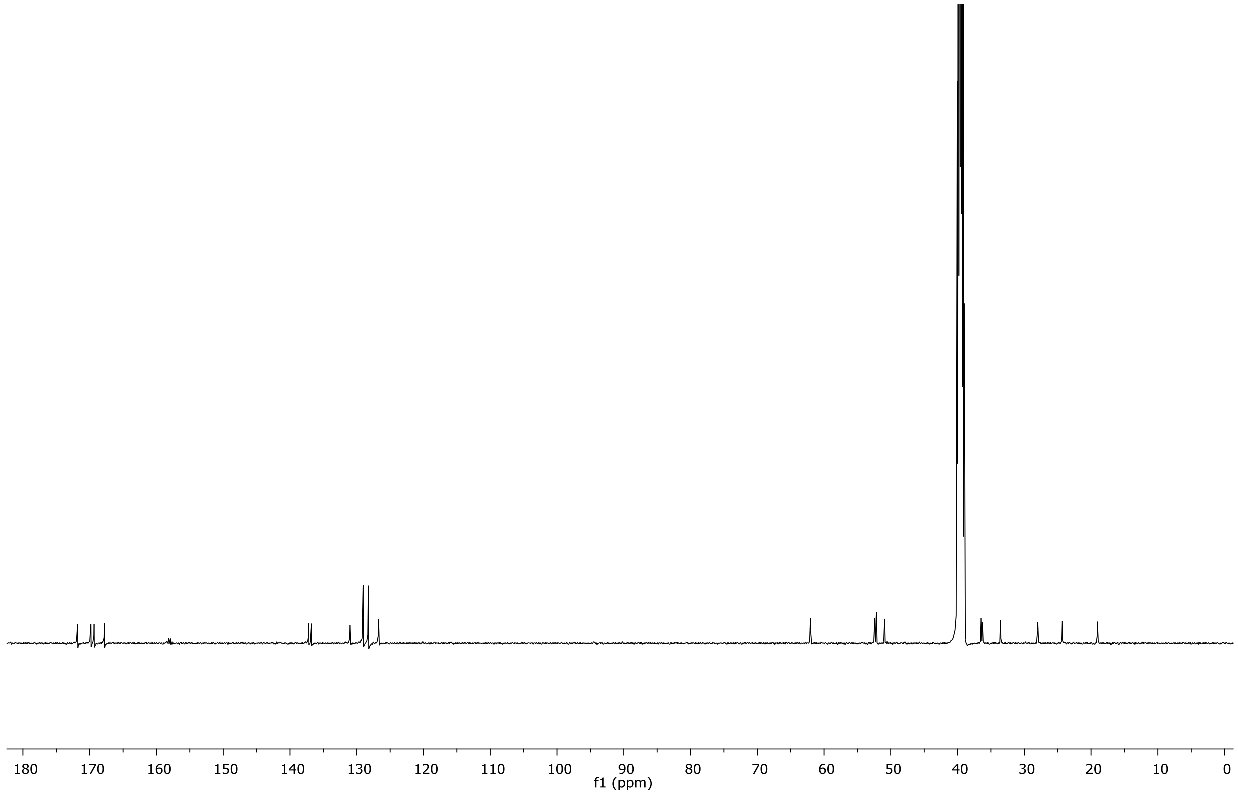
180
170
160
150
140
130
120
110
100
90
80
70
60
50
40
30
20
10
0
f1 (ppm)

B3x

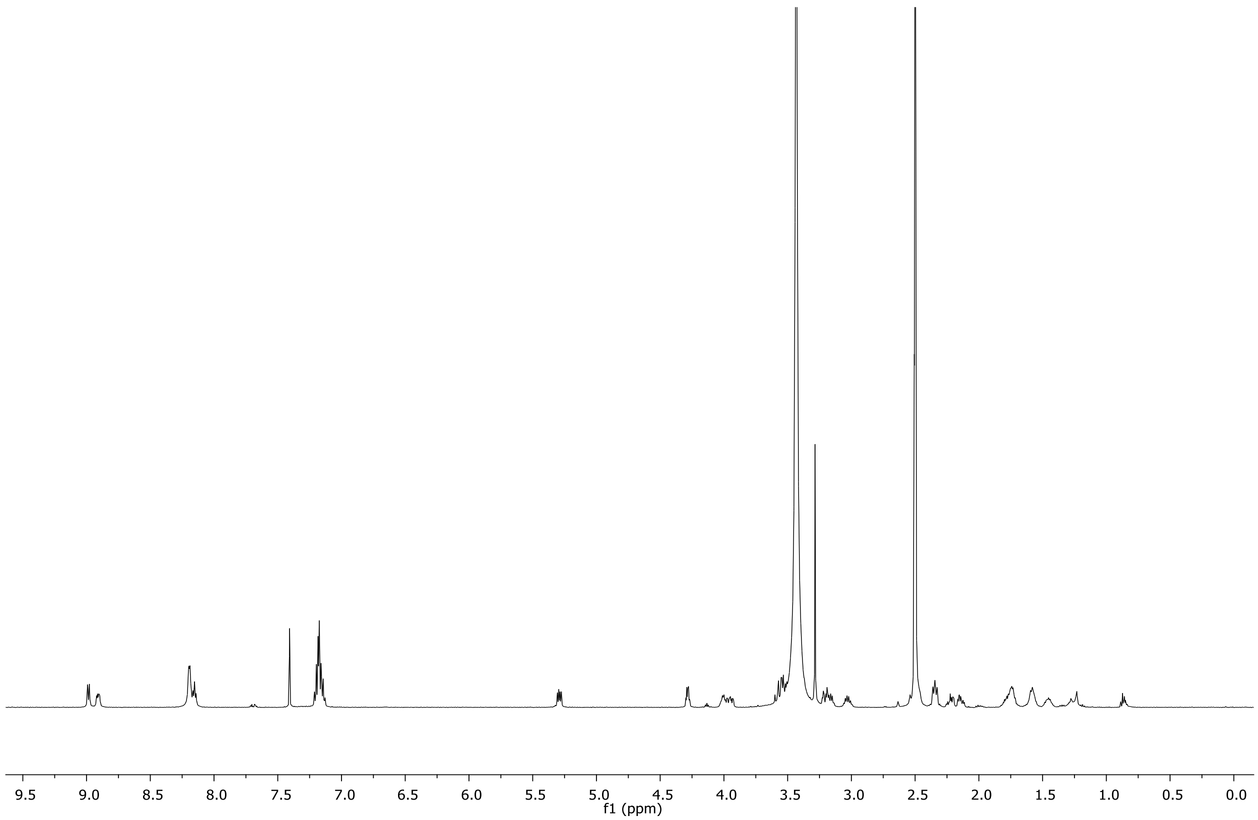
9.5
9.0
8.5
8.0
7.5
7.0
6.5
6.0
5.5
5.0
4.5
4.0
3.5
3.0
2.5
2.0
1.5
1.0
0.5
0.0
f1 (ppm)

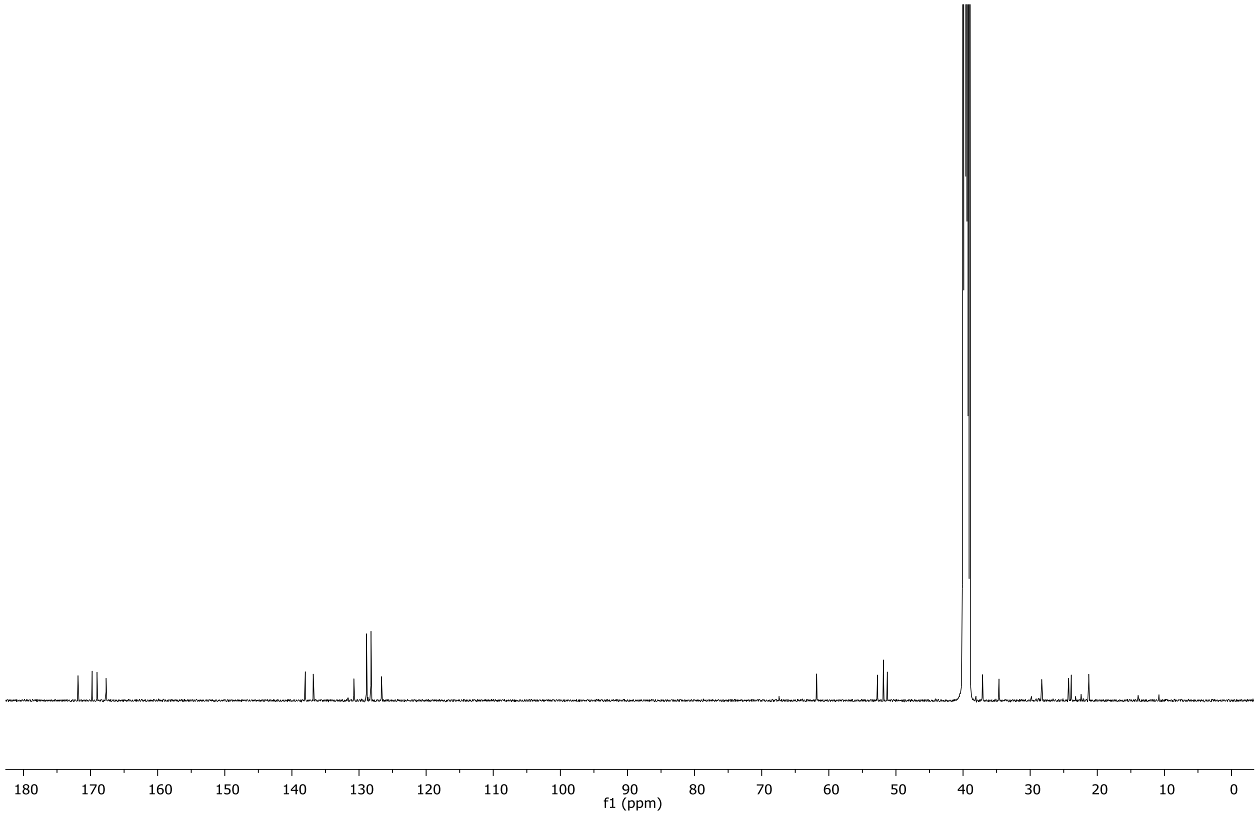
180
170
160
150
140
130
120
110
100
90
80
70
60
50
40
30
20
10
0
f1 (ppm)

B5x

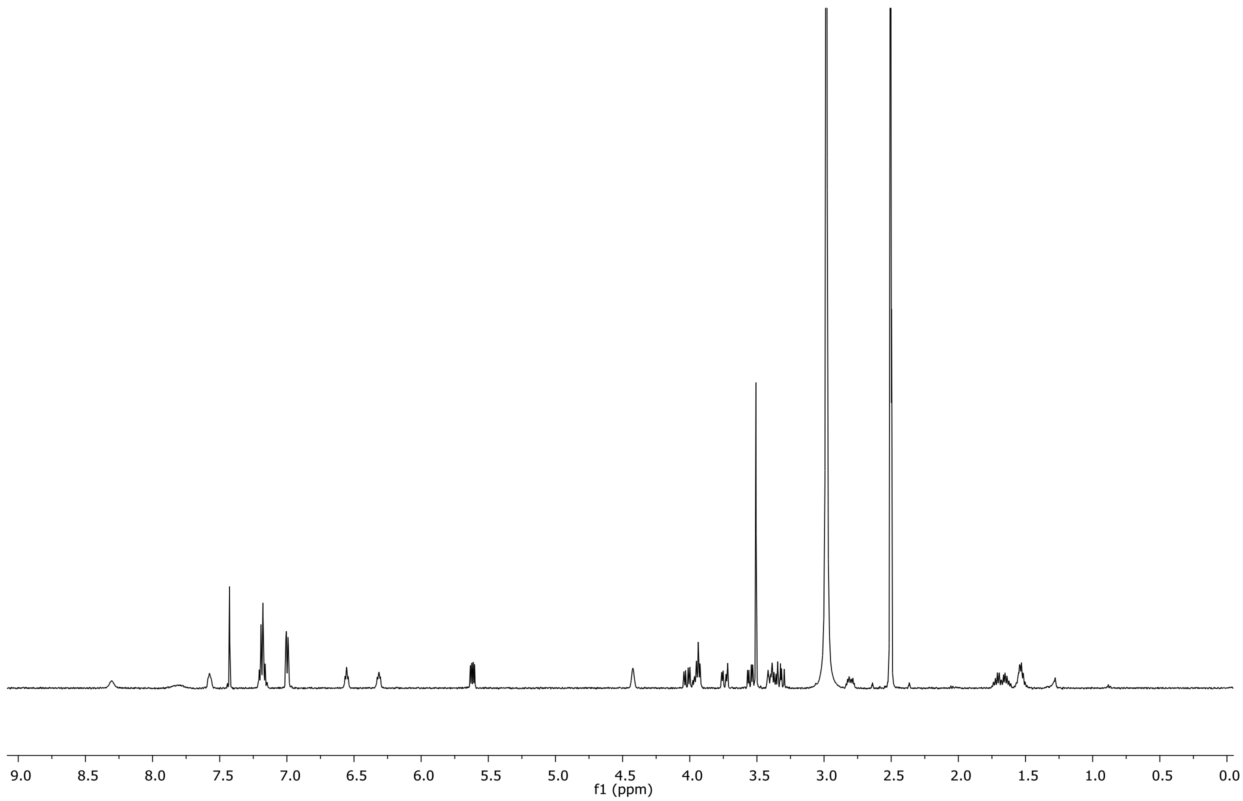
9.0
8.5
8.0
7.5
7.0
6.5
6.0
5.5
5.0
4.5
4.0
3.5
3.0
2.5
2.0
1.5
1.0
0.5
0.0
f1 (ppm)

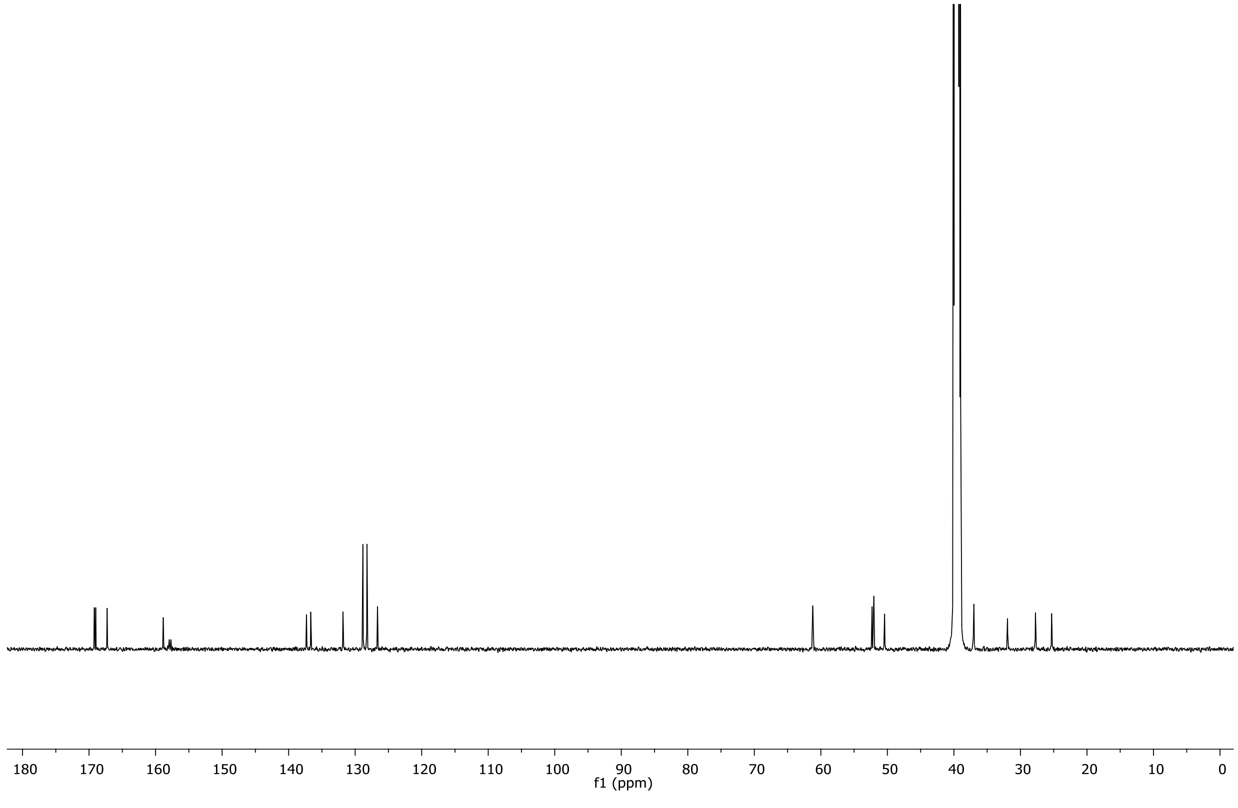
180
170
160
150
140
130
120
110
100
90
80
70
60
50
40
30
20
10
0
f1 (ppm)

B7x

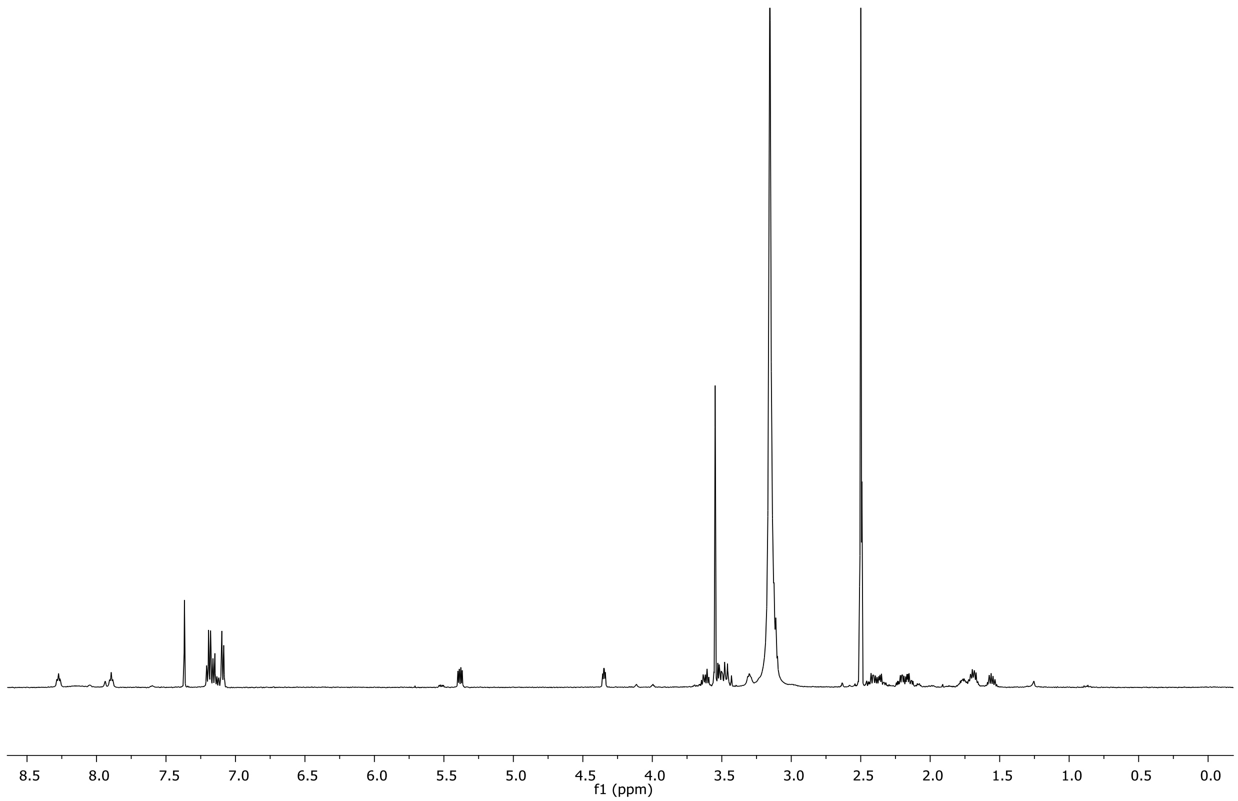
8.5
8.0
7.5
7.0
6.5
6.0
5.5
5.0
4.5
4.0
3.5
3.0
2.5
2.0
1.5
1.0
0.5
0.0
f1 (ppm)

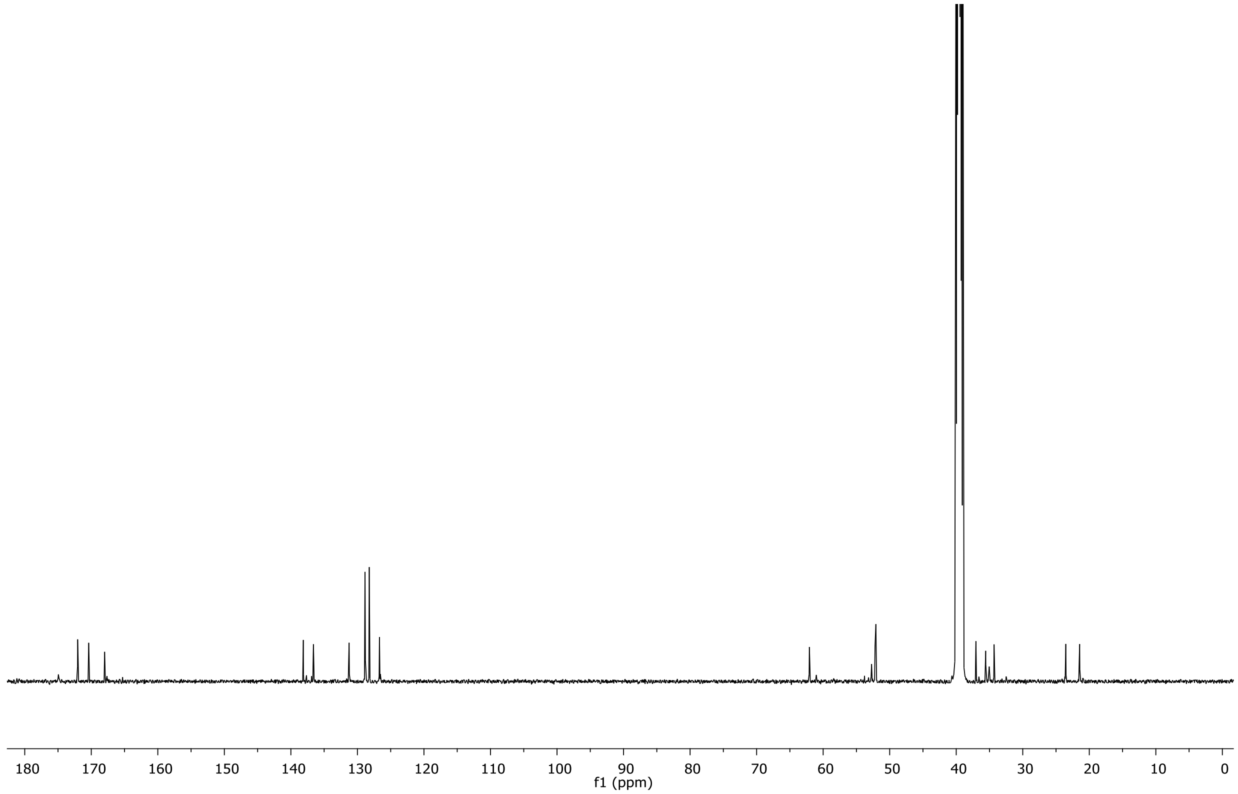
180
170
160
150
140
130
120
110
100
90
80
70
60
50
40
30
20
10
0
f1 (ppm)

B8x

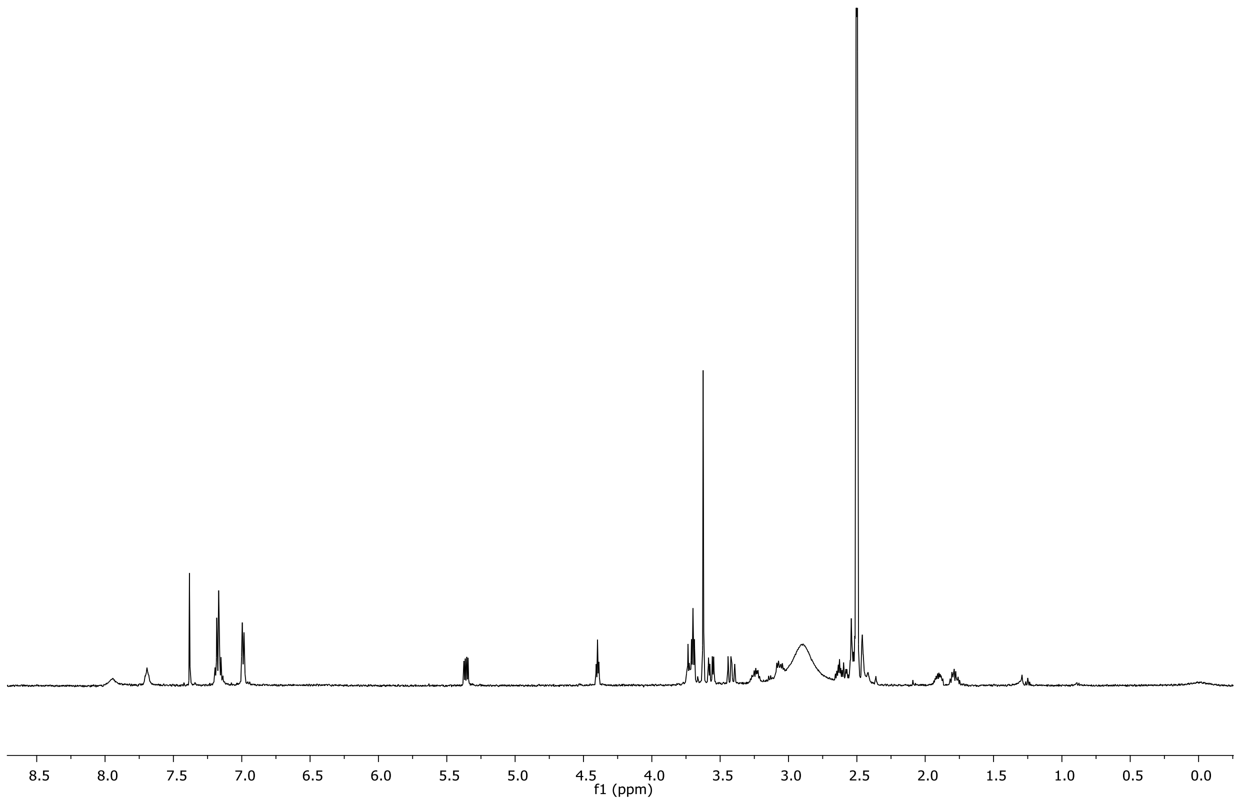

8.5
8.0
7.5
7.0
6.5
6.0
5.5
5.0
4.5
4.0
3.5
3.0
2.5
2.0
1.5
1.0
0.5
0.0
f1 (ppm)

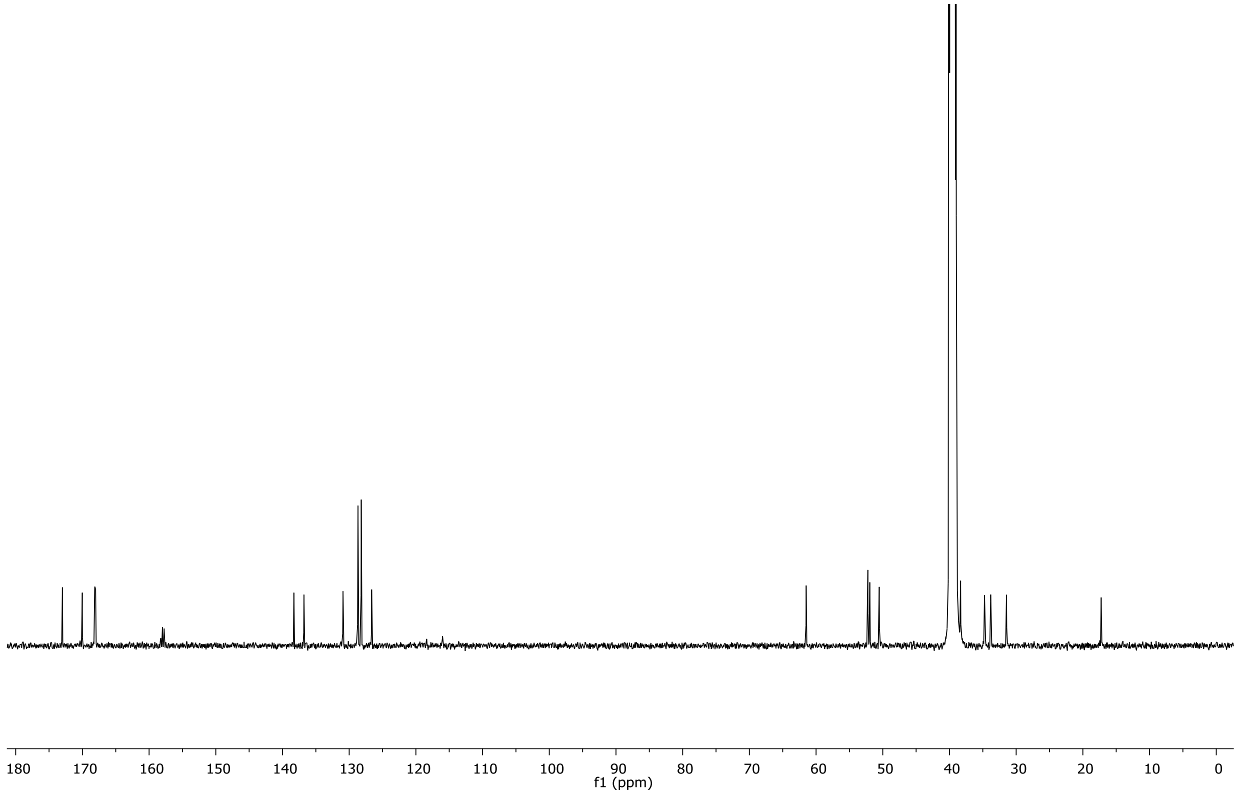

180
170
160
150
140
130
120
110
100
90
80
70
60
50
40
30
20
10
0
f1 (ppm)

B10x

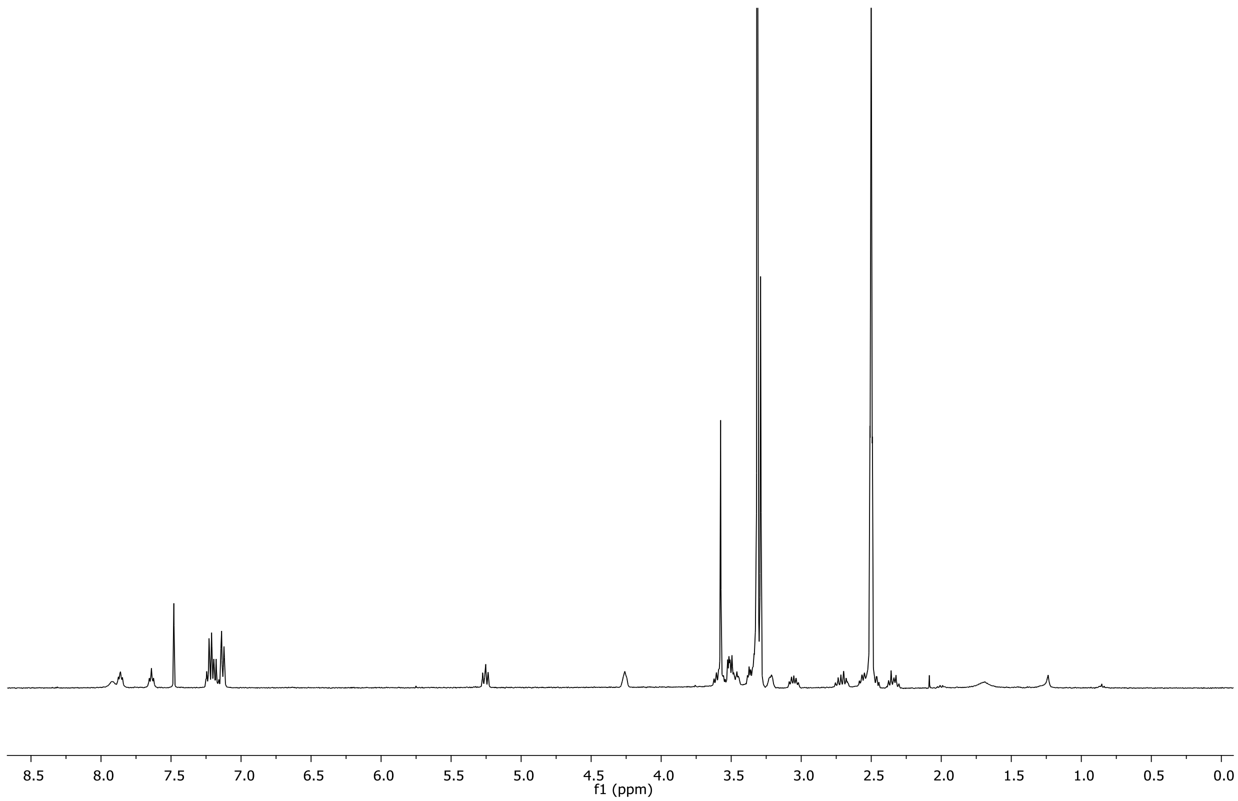

8.5 8.0 7.5 7.0 6.5 6.0 5.5 5.0 4.5 4.0 3.5 3.0 2.5 2.0 1.5 1.0 0.5 0.0
f1 (ppm)

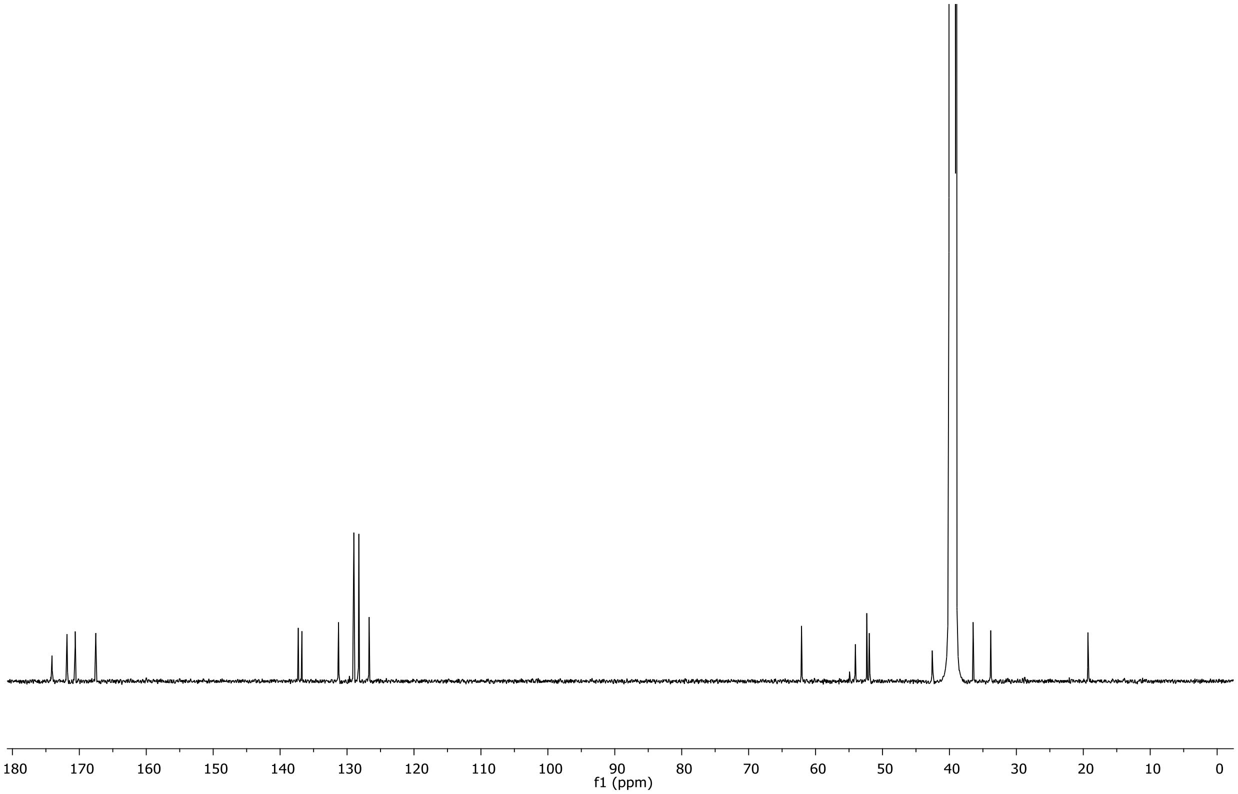

180 170 160 150 140 130 120 110 100 90 80 70 60 50 40 30 20 10 0
f1 (ppm)

B11x

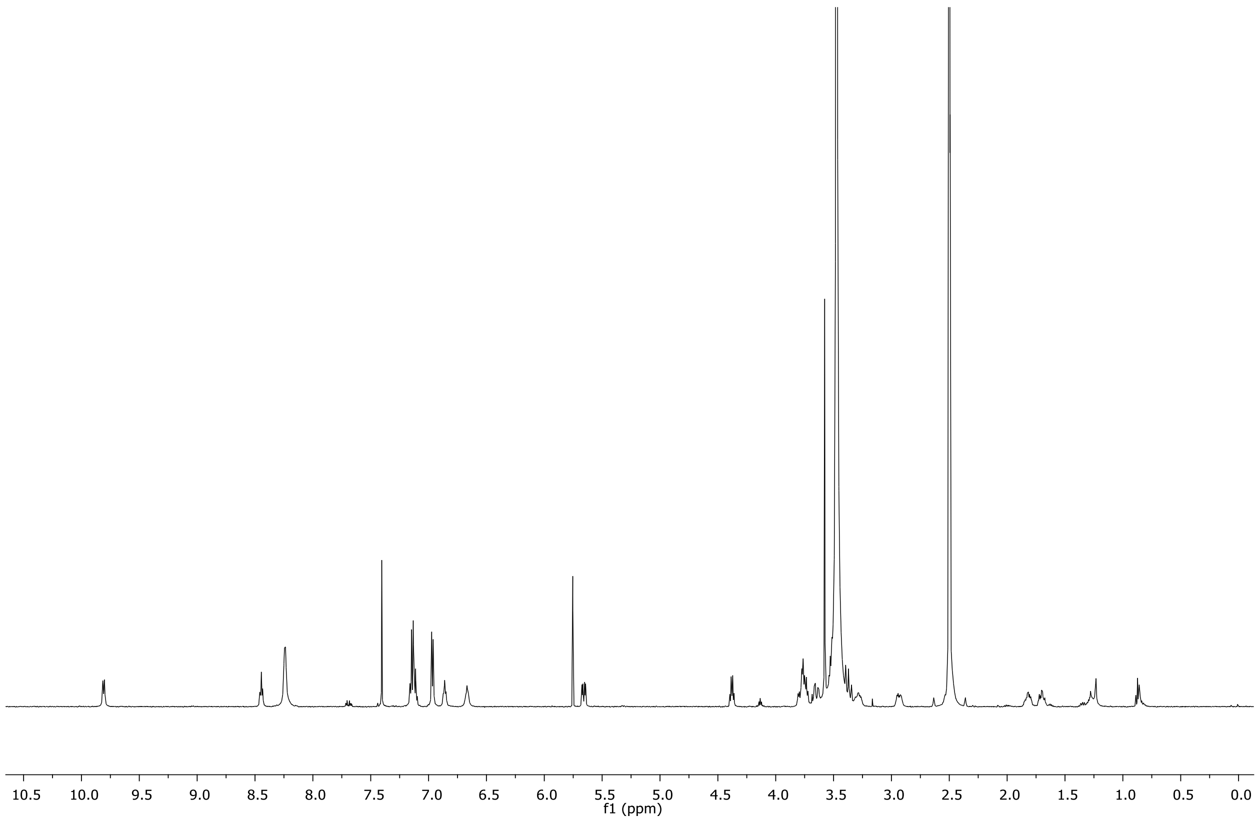
10.5 10.0 9.5 9.0 8.5 8.0 7.5 7.0 6.5 6.0 5.5 5.0 4.5 4.0 3.5 3.0 2.5 2.0 1.5 1.0 0.5 0.0
f1 (ppm)

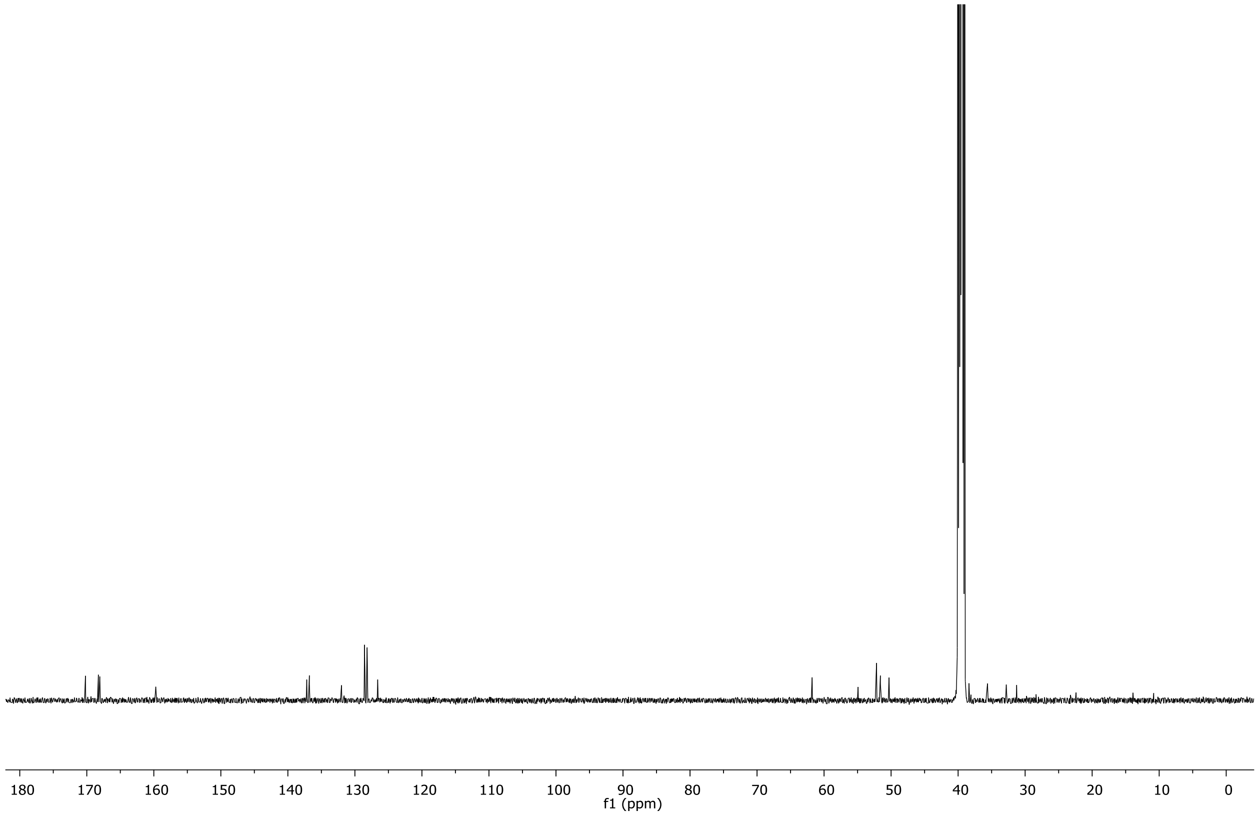
180 170 160 150 140 130 120 110 100 90 80 70 60 50 40 30 20 10 0
f1 (ppm)

B12x

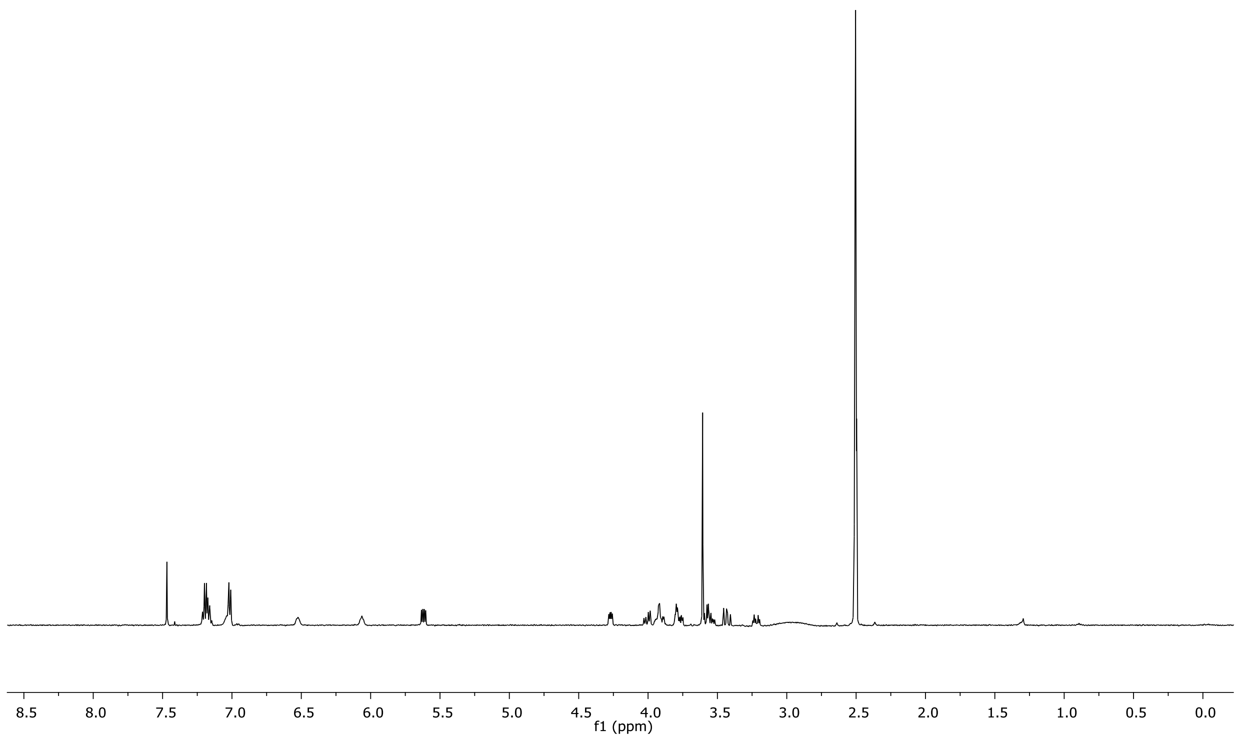
8.5
8.0
7.5
7.0
6.5
6.0
5.5
5.0
4.5
4.0
3.5
3.0
2.5
2.0
1.5
1.0
0.5
0.0
f1 (ppm)

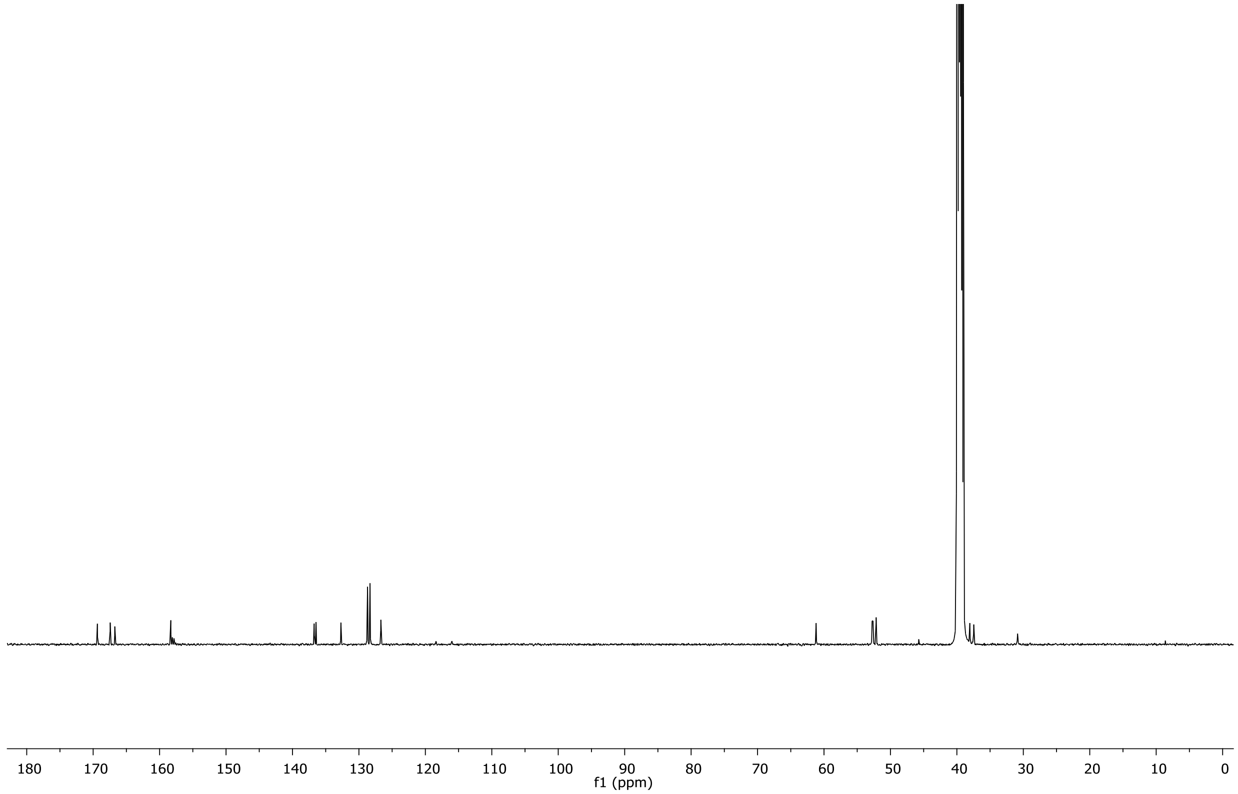
180
170
160
150
140
130
120
110
100
90
80
70
60
50
40
30
20
10
0
f1 (ppm)

B14x

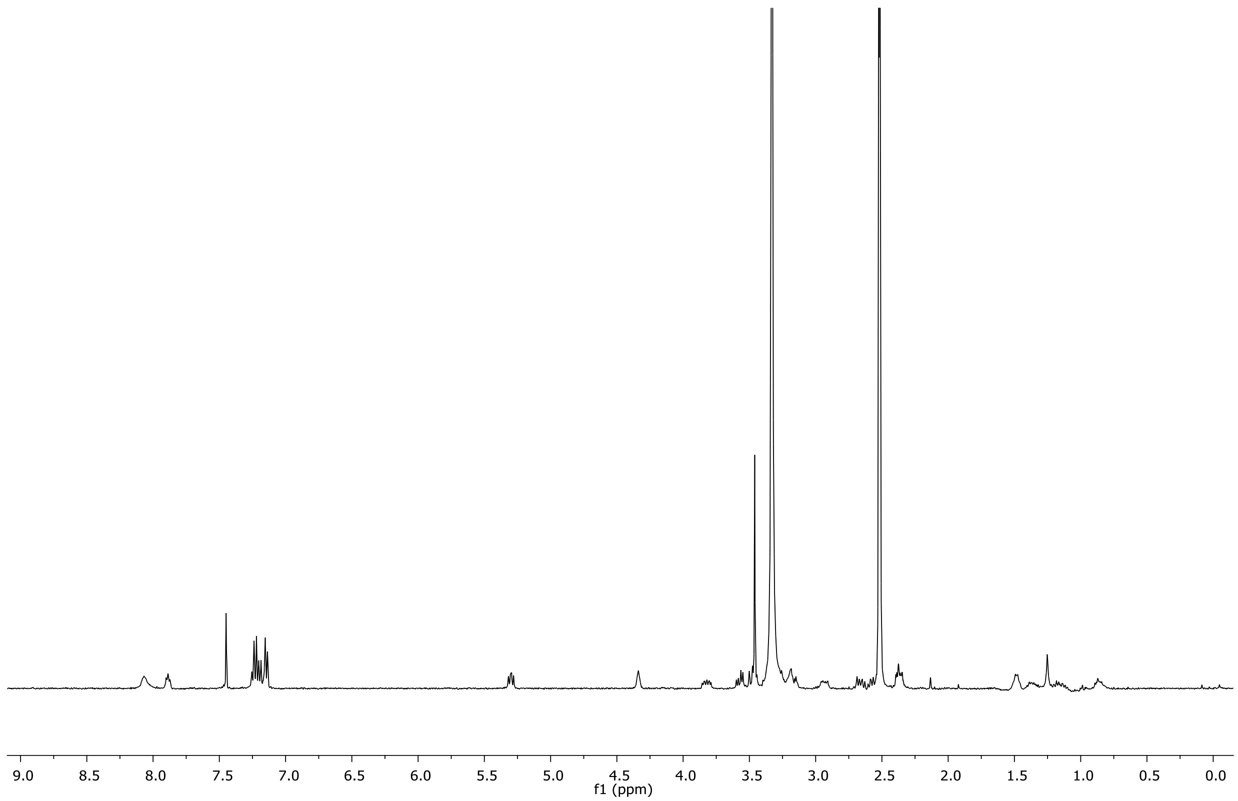
9.0
8.5
8.0
7.5
7.0
6.5
6.0
5.5
5.0
4.5
4.0
3.5
3.0
2.5
2.0
1.5
1.0
0.5
0.0
f1 (ppm)

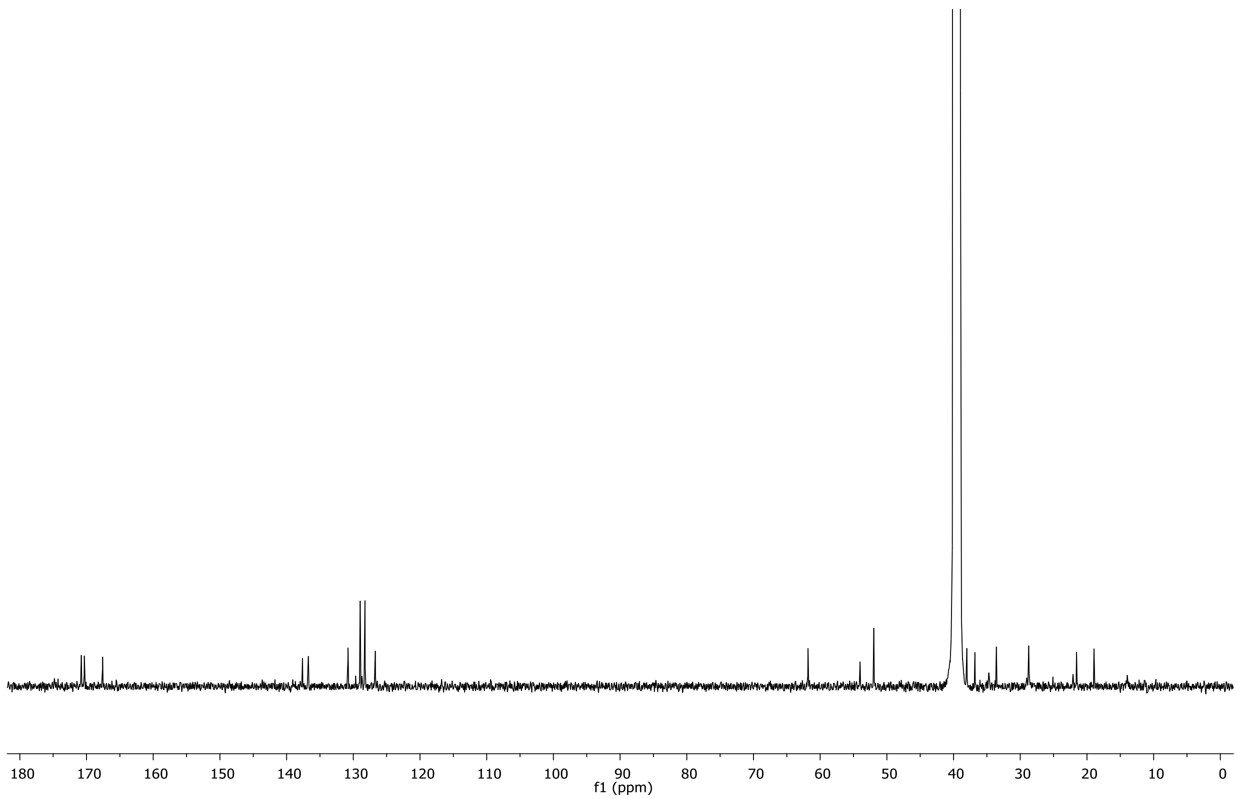
180
170
160
150
140
130
120
110
100
90
80
70
60
50
40
30
20
10
0
f1 (ppm)

C3x

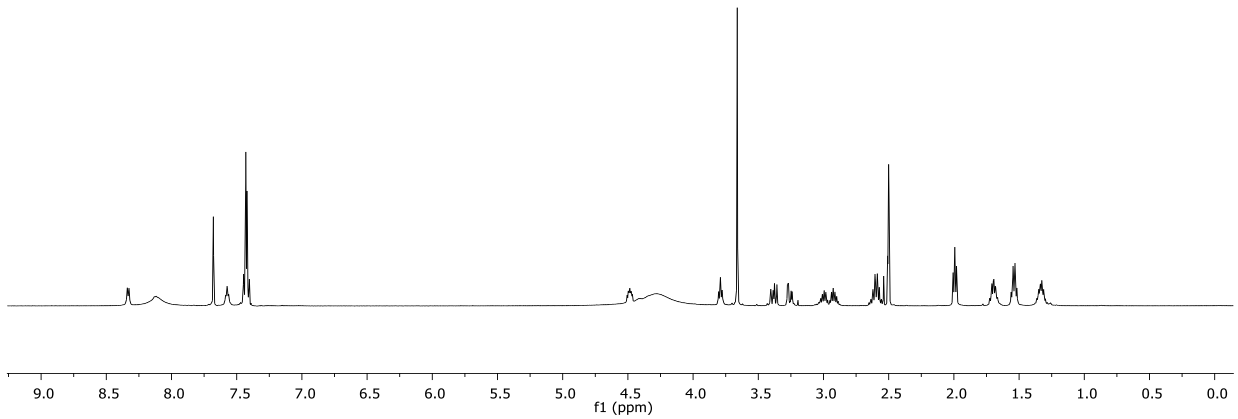
9.0
8.5
8.0
7.5
7.0
6.5
6.0
5.5
5.0
4.5
4.0
3.5
3.0
2.5
2.0
1.5
1.0
0.5
0.0
f1 (ppm)

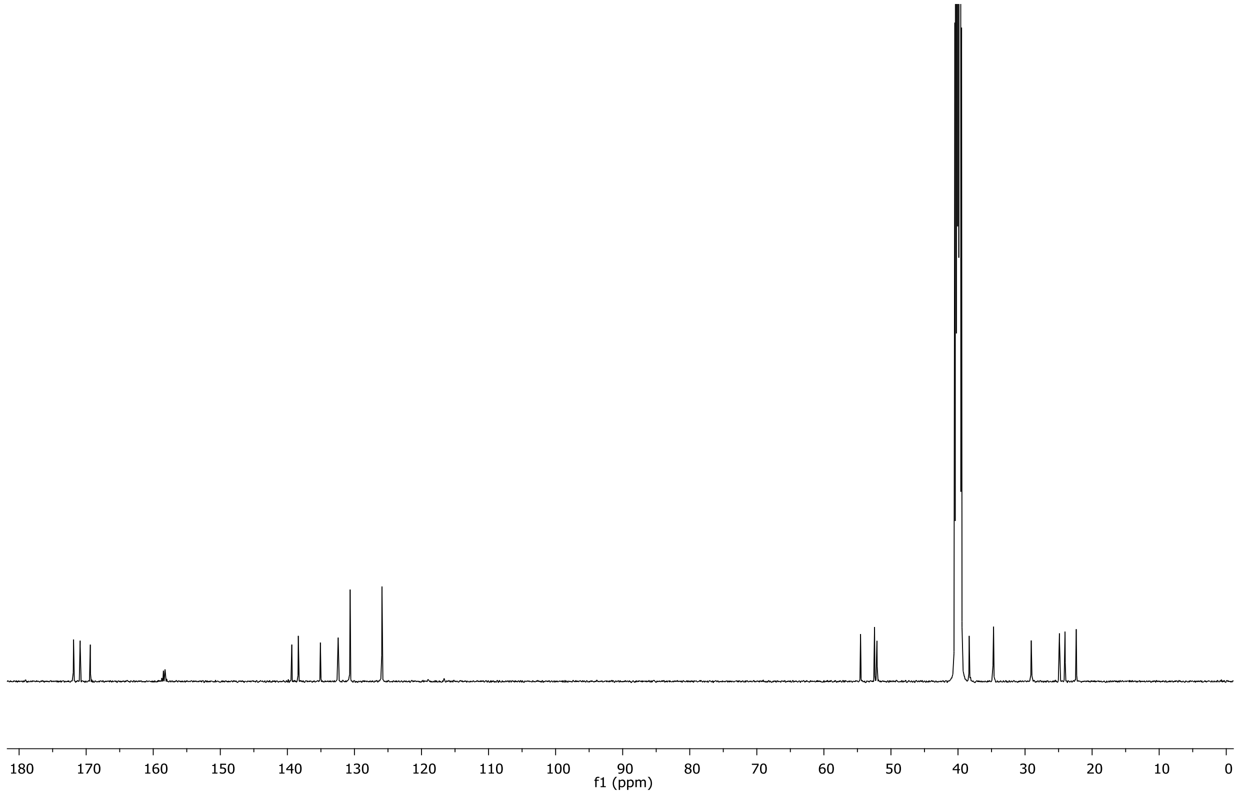
180
170
160
150
140
130
120
110
100
90
80
70
60
50
40
30
20
10
0
f1 (ppm)

C7x

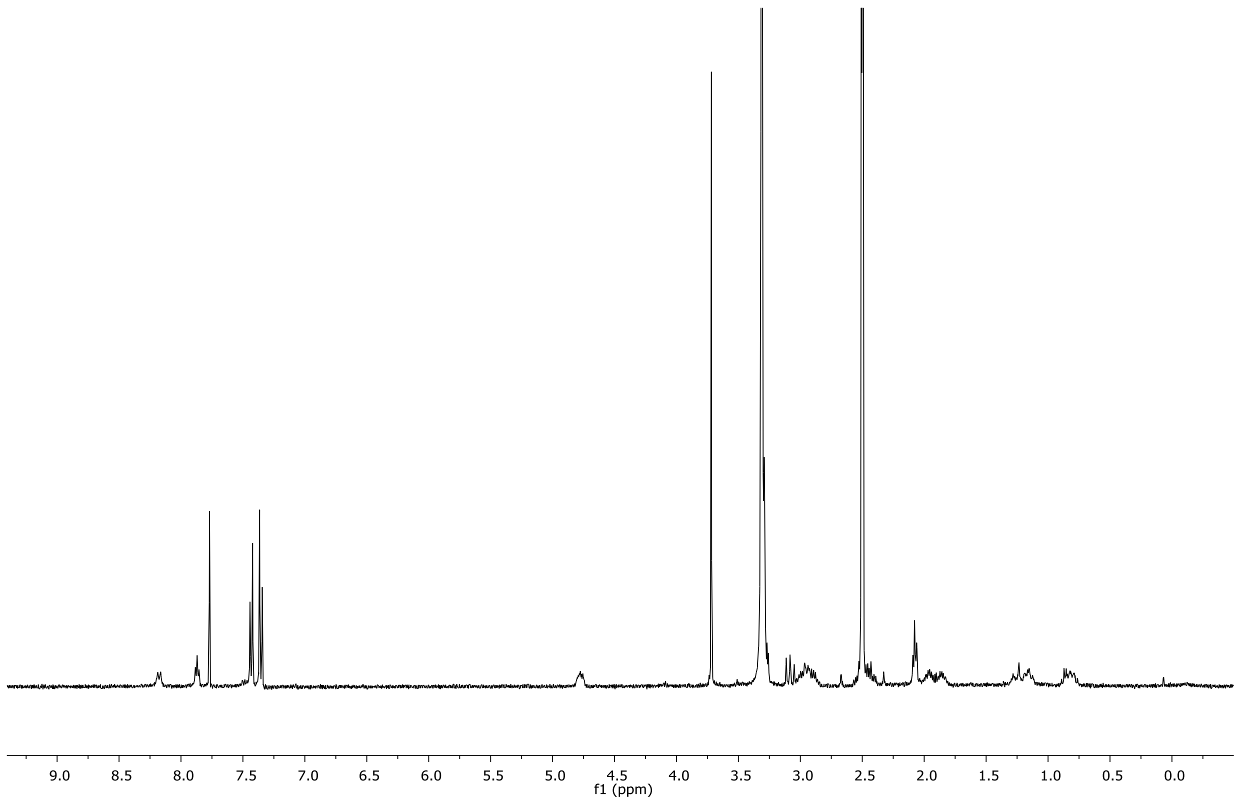
9.0
8.5
8.0
7.5
7.0
6.5
6.0
5.5
5.0
4.5
4.0
3.5
3.0
2.5
2.0
1.5
1.0
0.5
0.0
f1 (ppm)

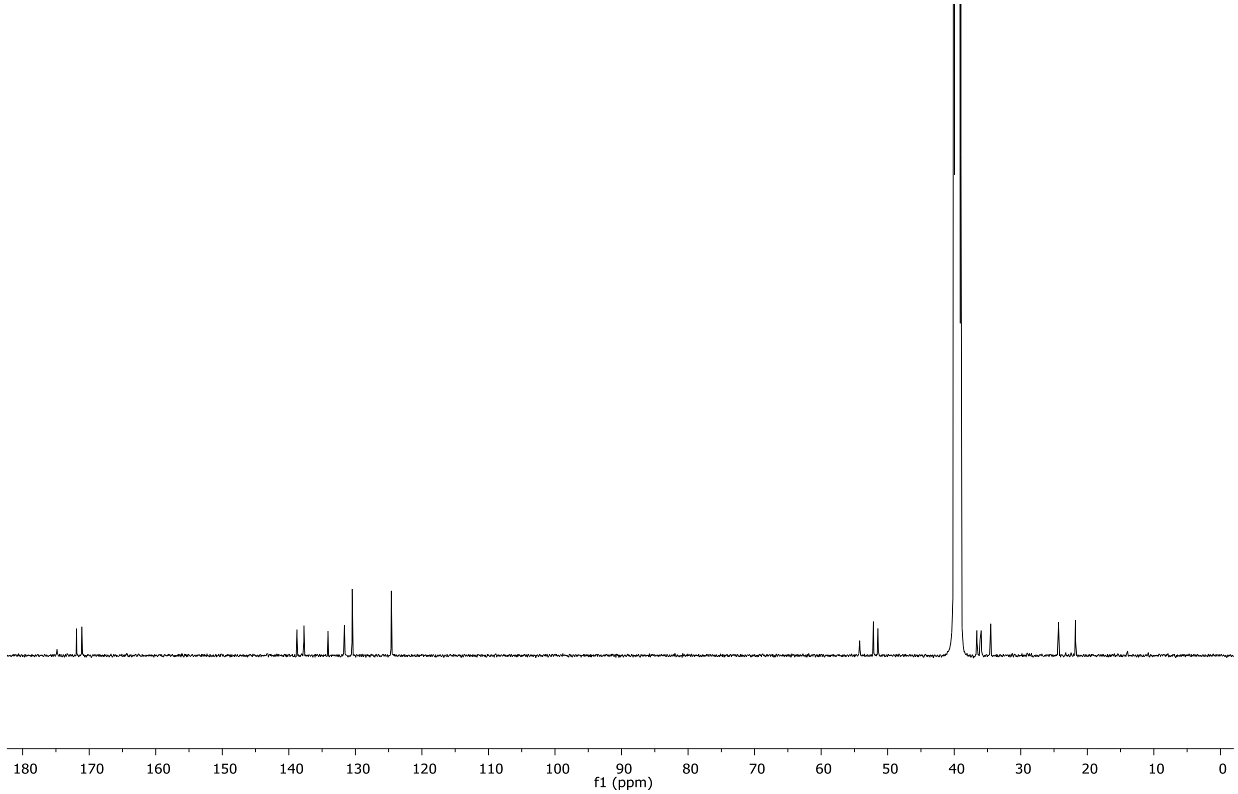
180
170
160
150
140
130
120
110
100
90
80
70
60
50
40
30
20
10
0
f1 (ppm)

C8x

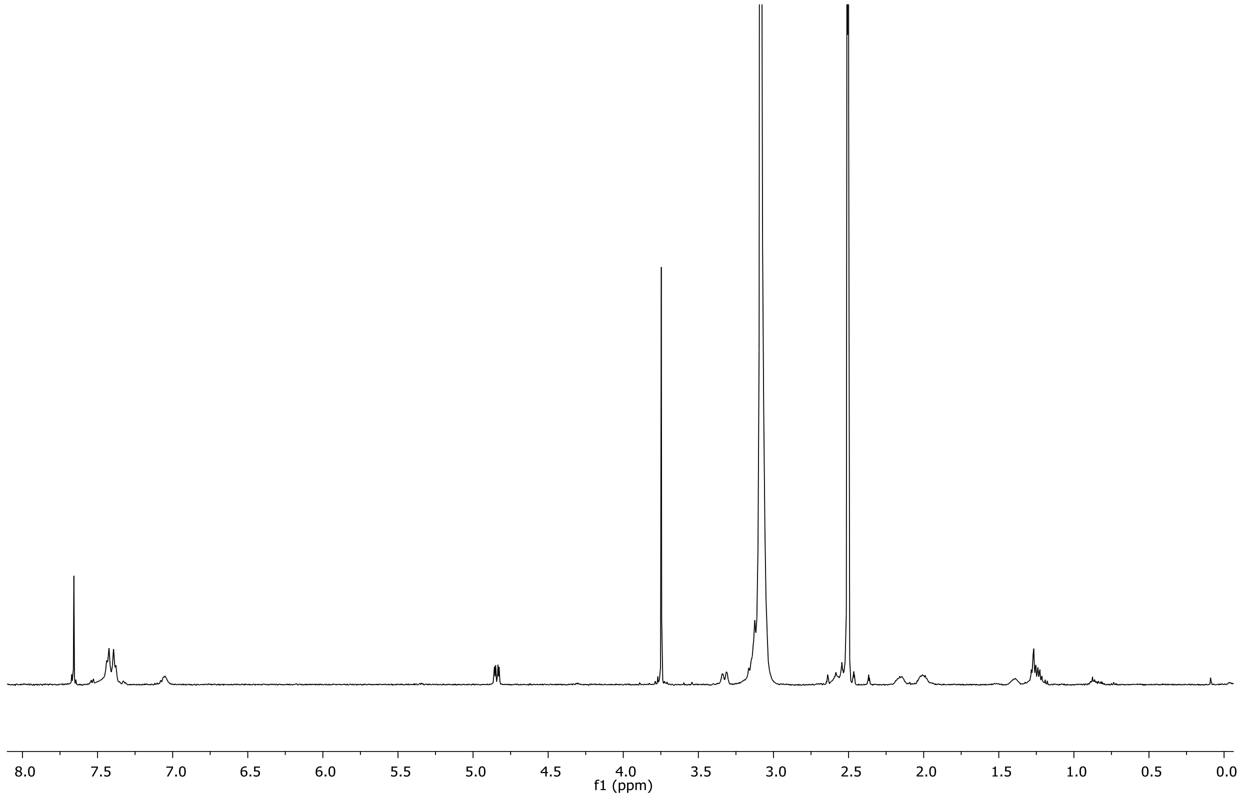

8.0
7.5
7.0
6.5
6.0
5.5
5.0
4.5
4.0
3.5
3.0
2.5
2.0
1.5
1.0
0.5
0.0
f1 (ppm)

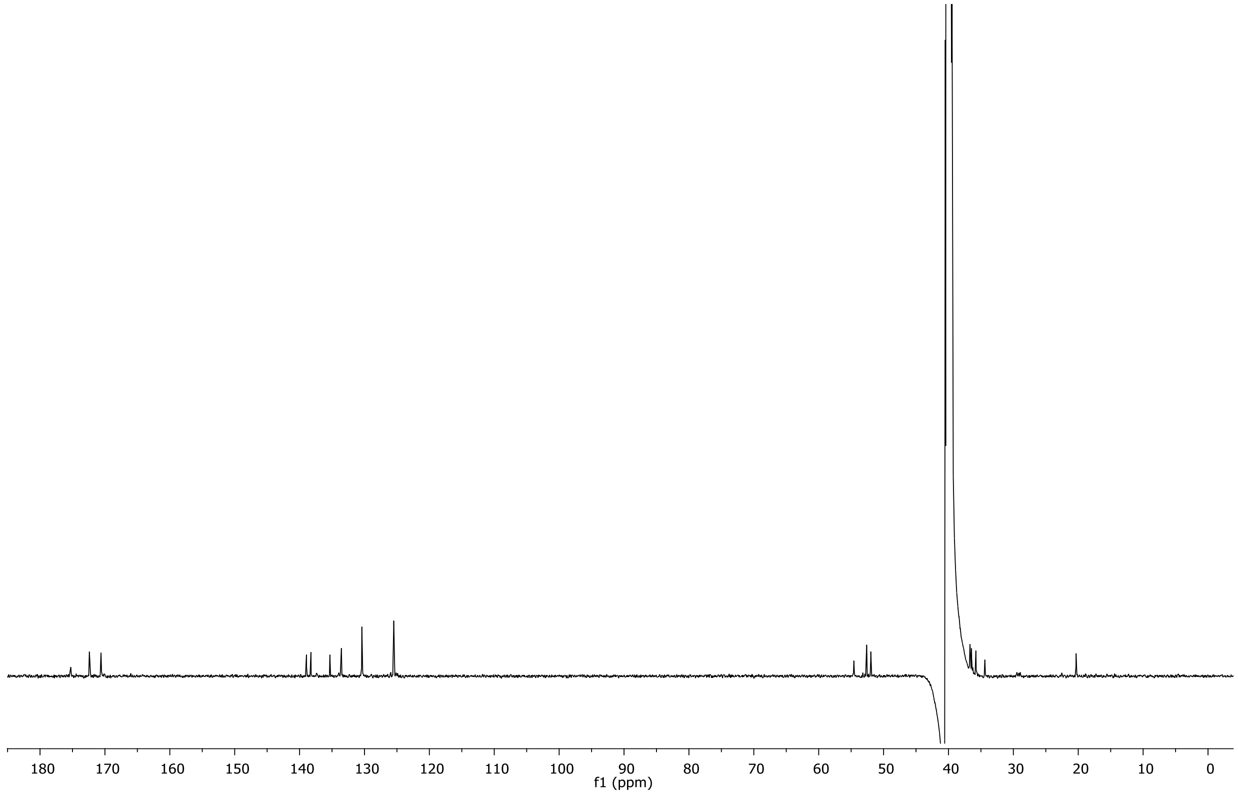

180
170
160
150
140
130
120
110
100
90
80
70
60
50
40
30
20
10
0
f1 (ppm)

C10x

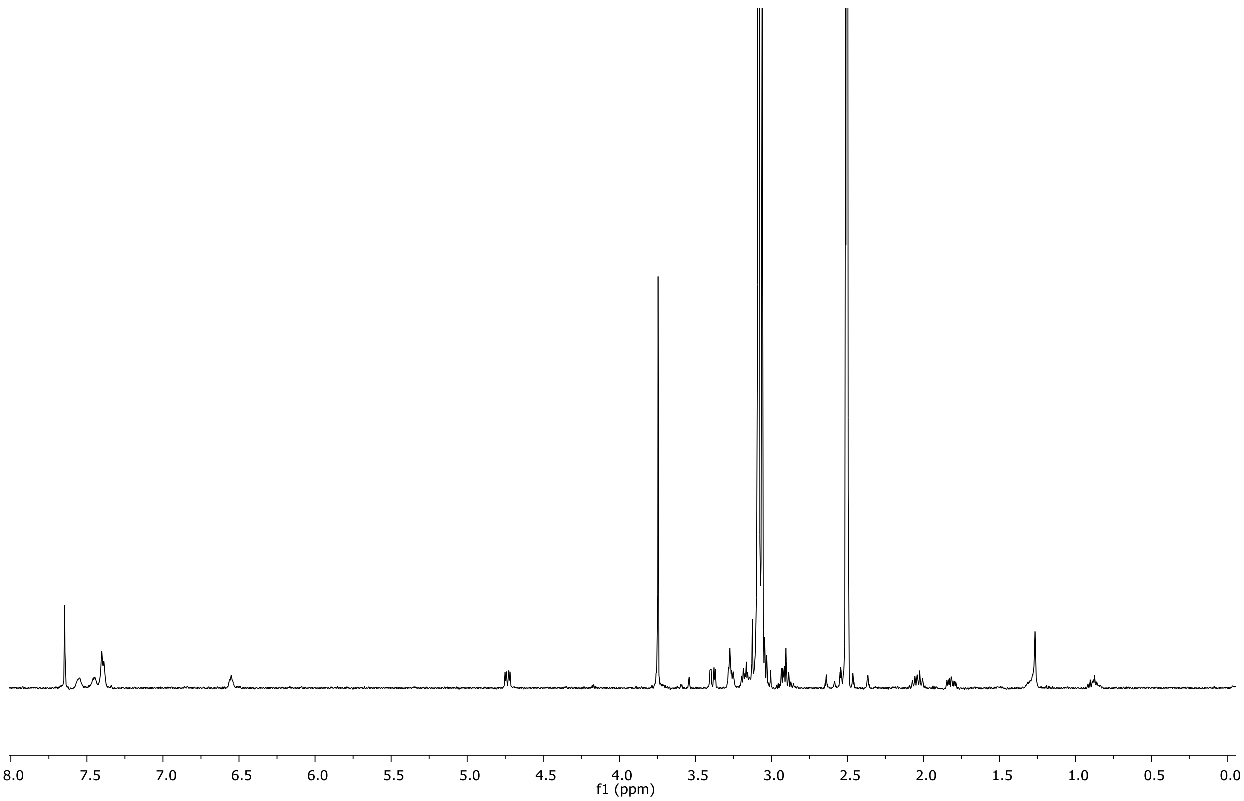
8.0
7.5
7.0
6.5
6.0
5.5
5.0
4.5
4.0
3.5
3.0
2.5
2.0
1.5
1.0
0.5
0.0
f1 (ppm)

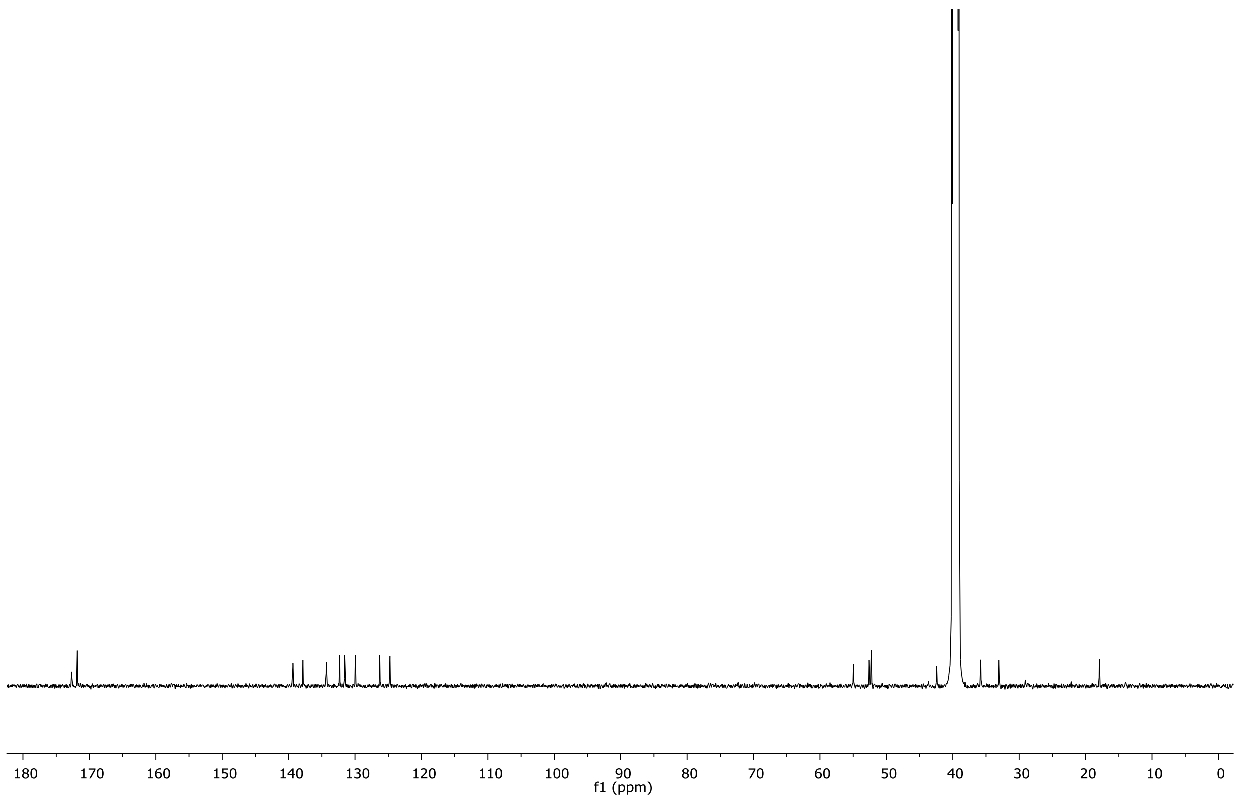
180
170
160
150
140
130
120
110
100
90
80
70
60
50
40
30
20
10
0
f1 (ppm)

C14x

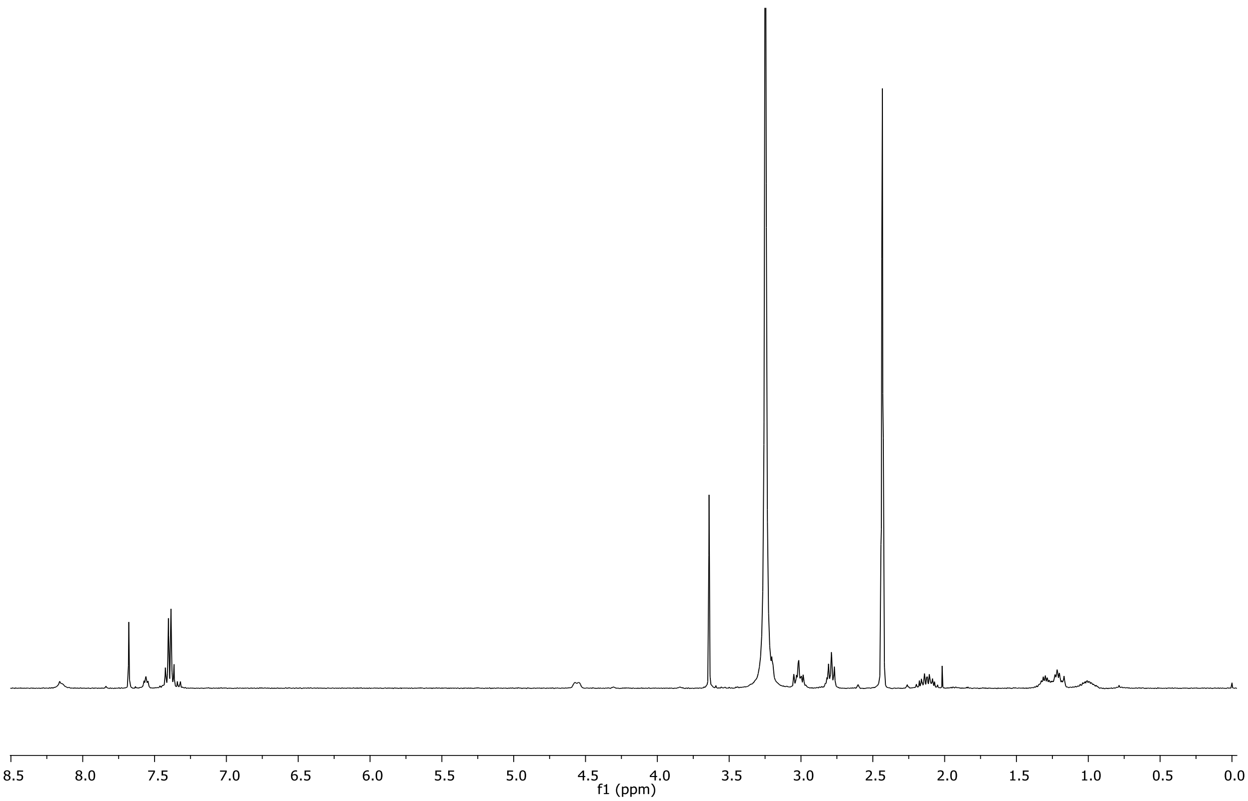
8.5
8.0
7.5
7.0
6.5
6.0
5.5
5.0
4.5
4.0
3.5
3.0
2.5
2.0
1.5
1.0
0.5
0.0
f1 (ppm)

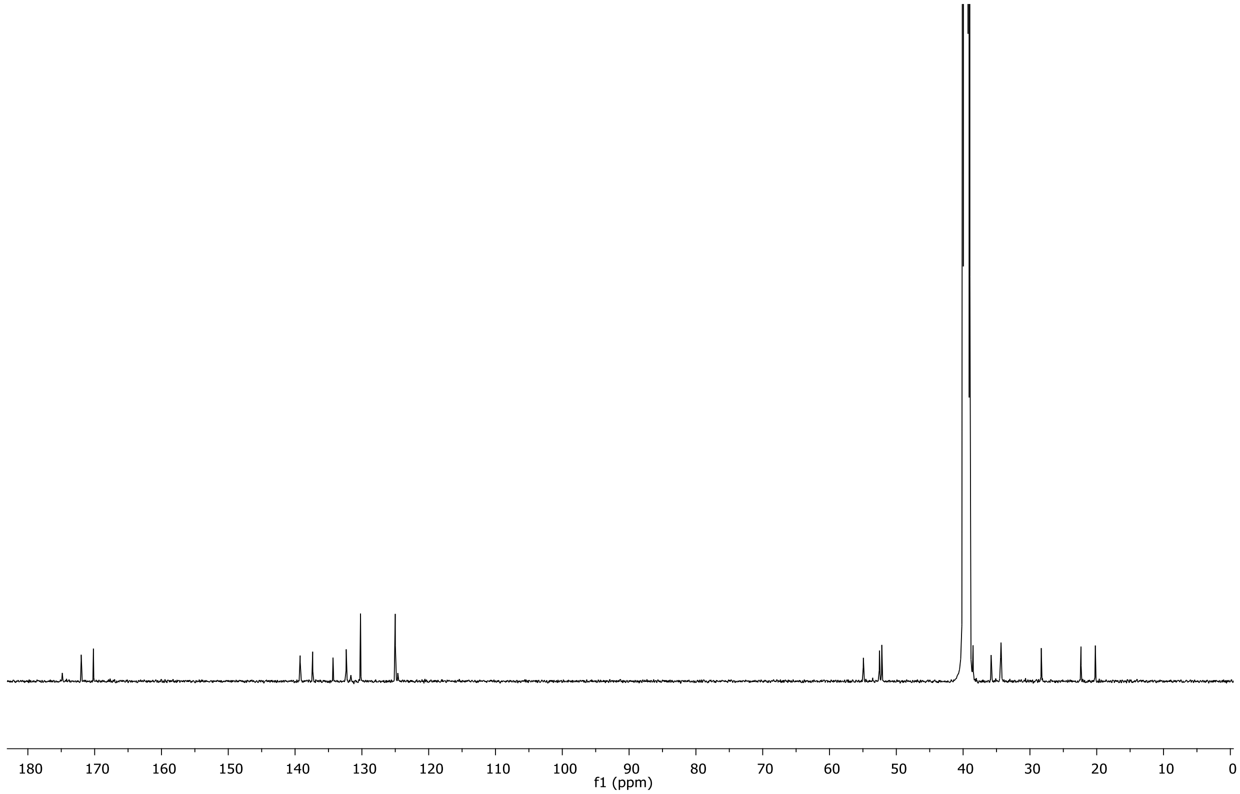
180
170
160
150
140
130
120
110
100
90
80
70
60
50
40
30
20
10
0
f1 (ppm)

D1x

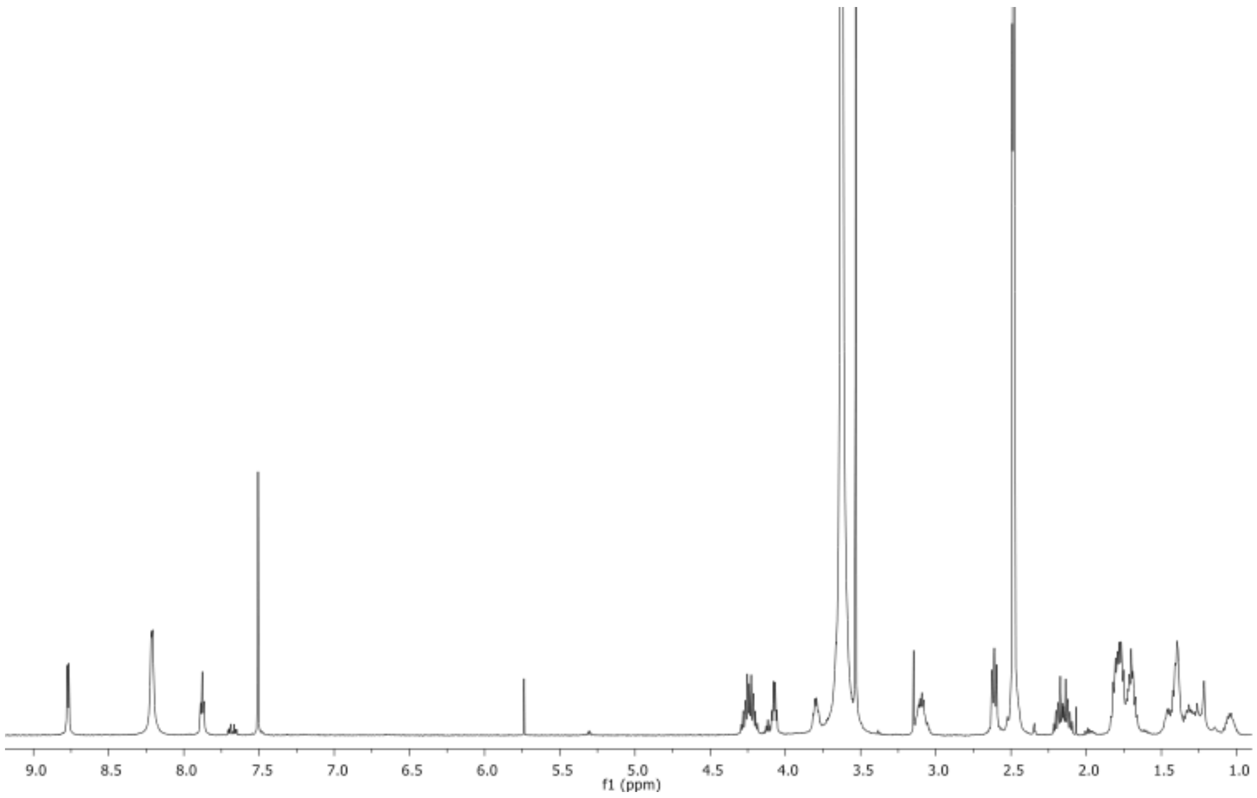
9.0
8.5
8.0
7.5
7.0
6.5
6.0
5.5
5.0
4.5
4.0
3.5
3.0
2.5
2.0
1.5
1.0
f1 (ppm)

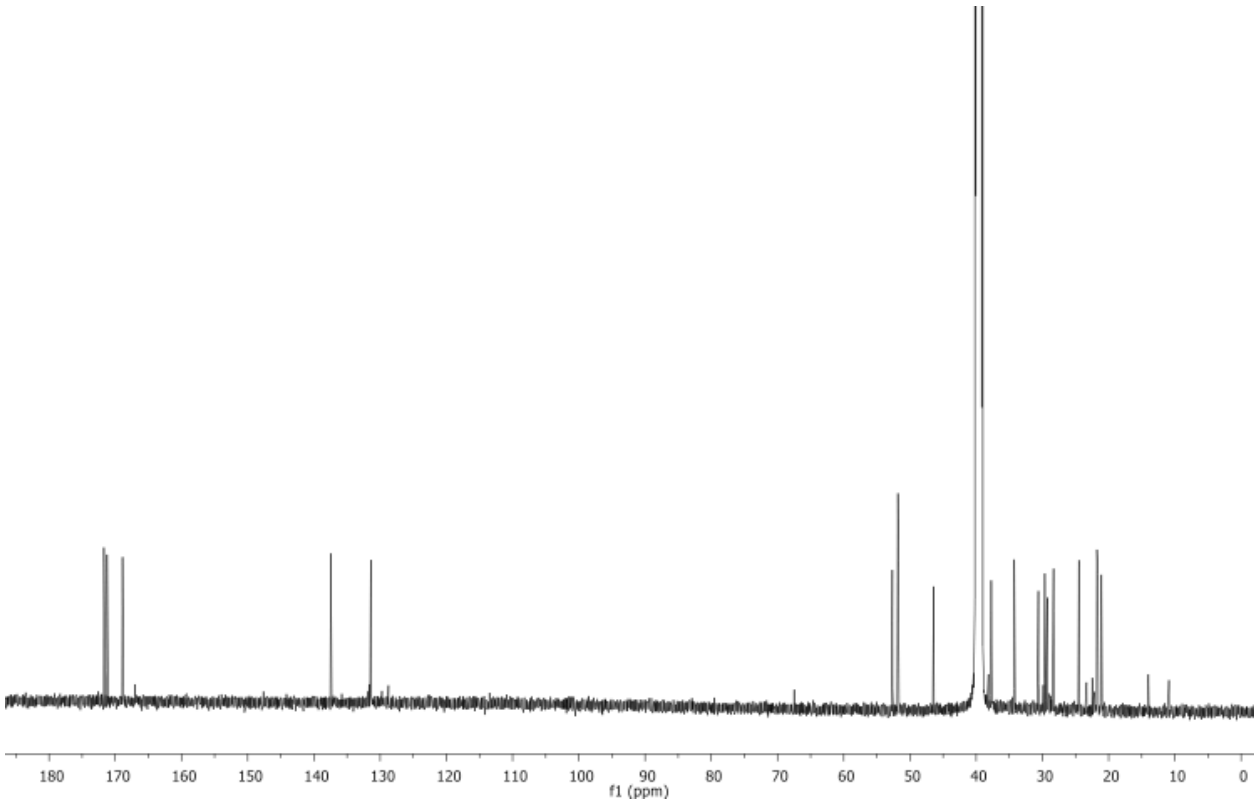
180
170
160
150
140
130
120
110
100
90
80
70
60
50
40
30
20
10
0
f1 (ppm)

D8x

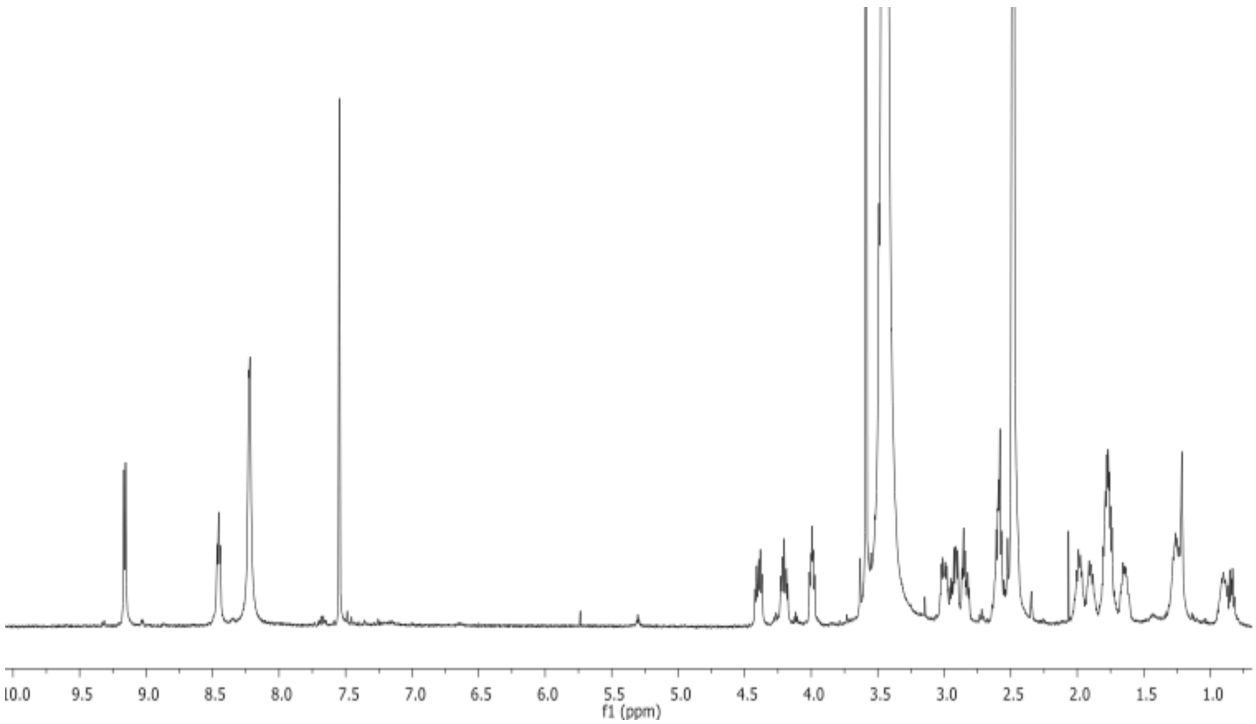

10.0 9.5 9.0 8.5 8.0 7.5 7.0 6.5 6.0 5.5 5.0 4.5 4.0 3.5 3.0 2.5 2.0 1.5 1.0
f1 (ppm)

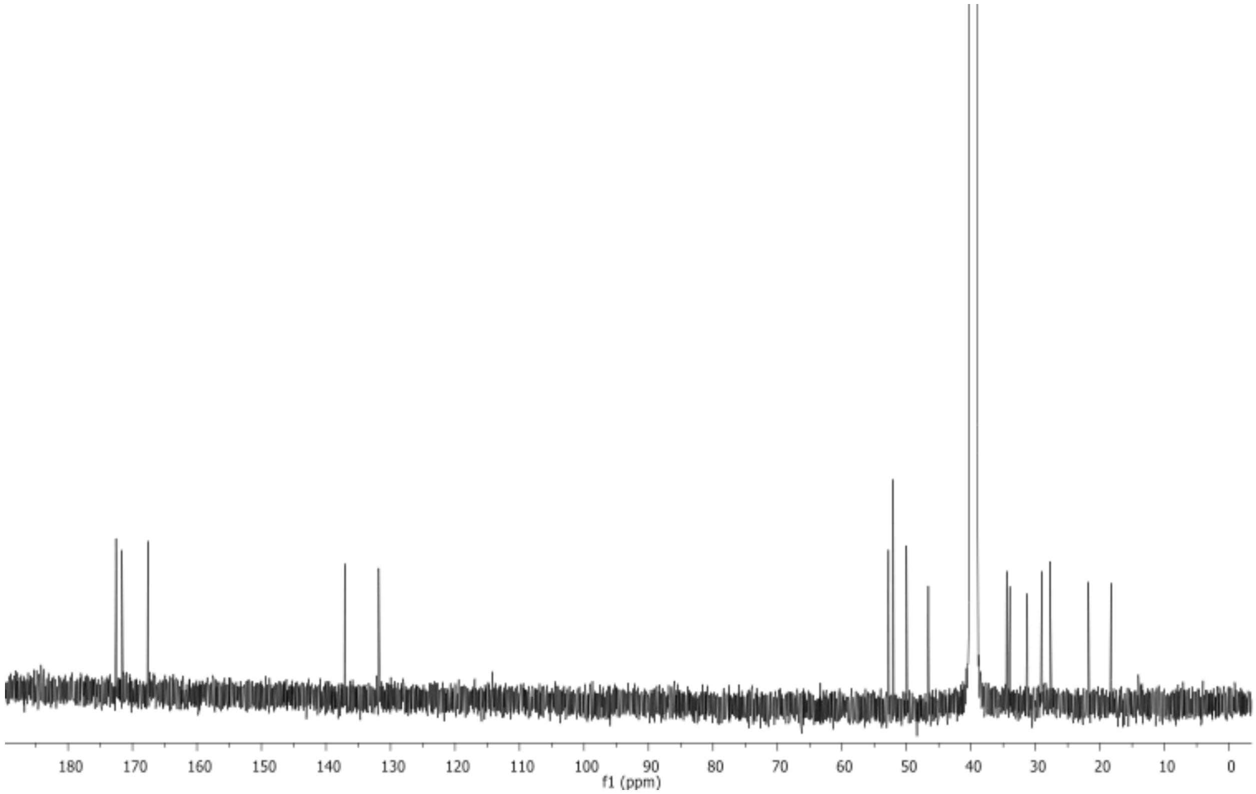

180 170 160 150 140 130 120 110 100 90 80 70 60 50 40 30 20 10 0
f1 (ppm)

D11x

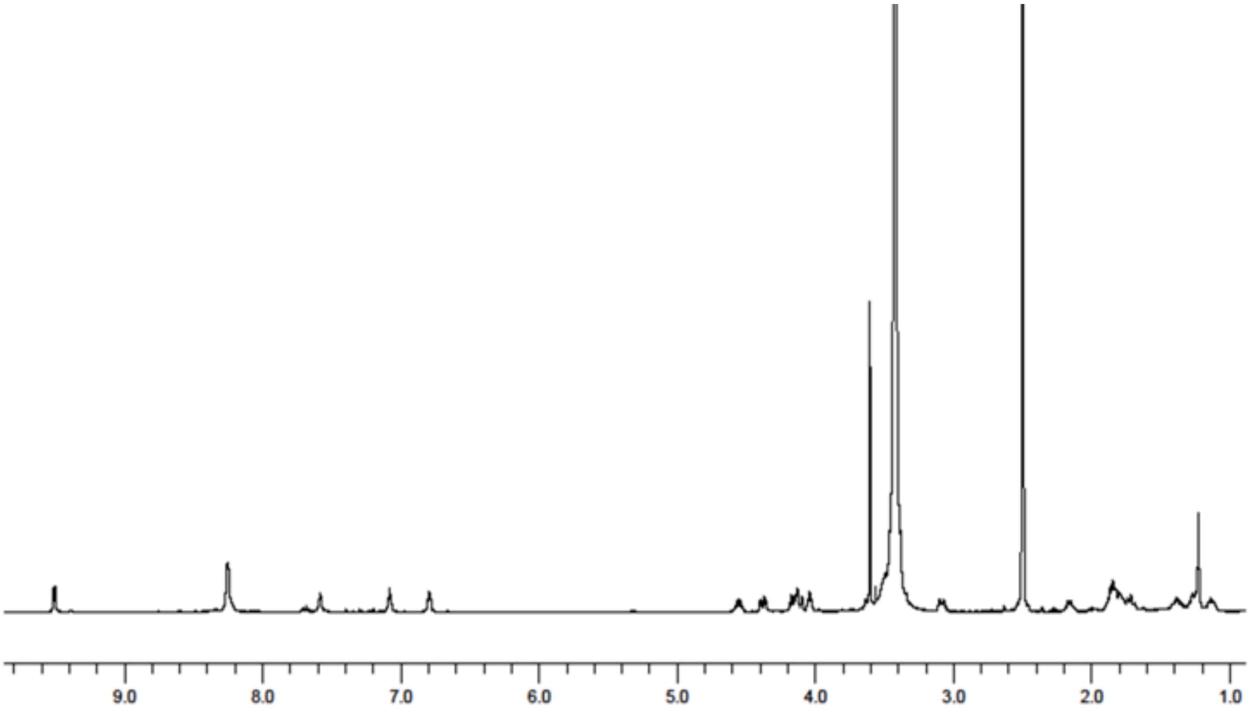

9.0
8.0
7.0
6.0
5.0
4.0
3.0
2.0
1.0

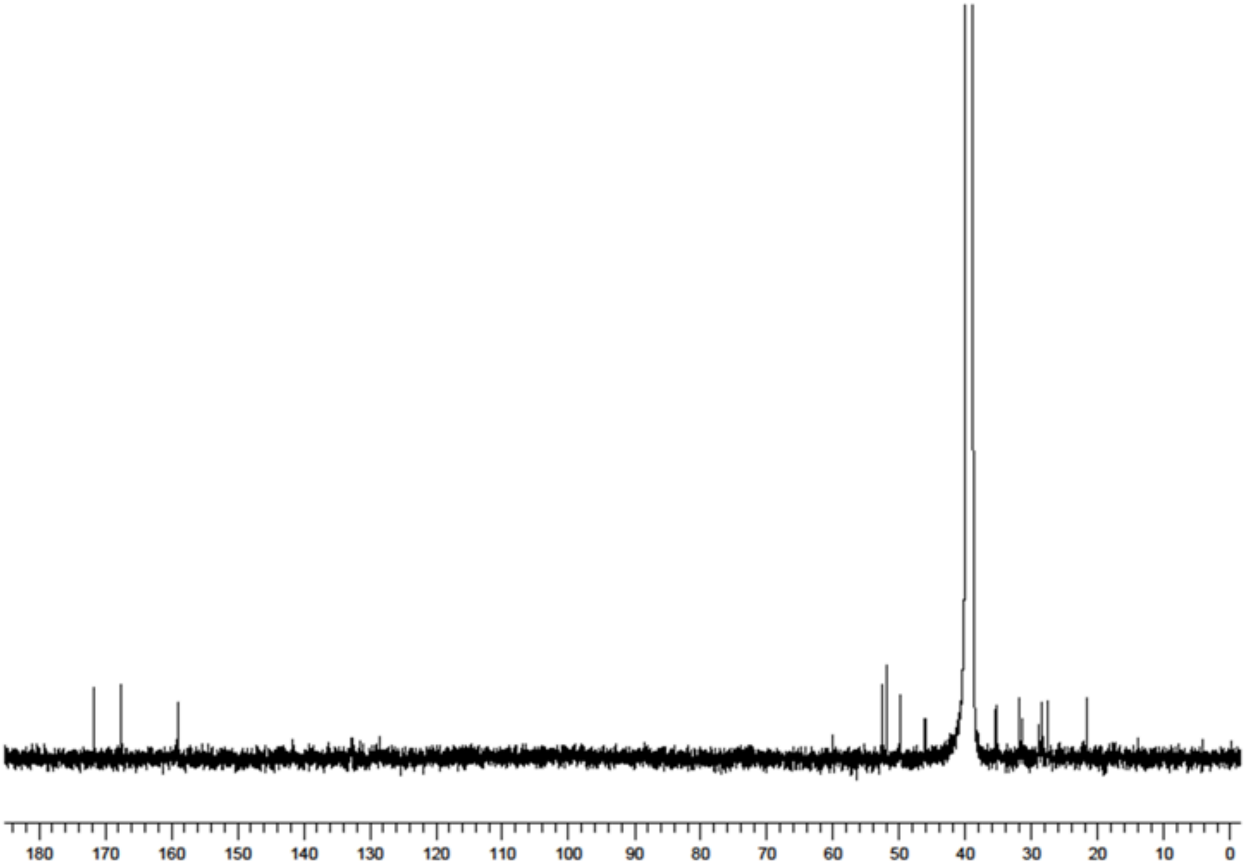

180
170
160
150
140
130
120
110
100
90
80
70
60
50
40
30
20
10
0

D14x

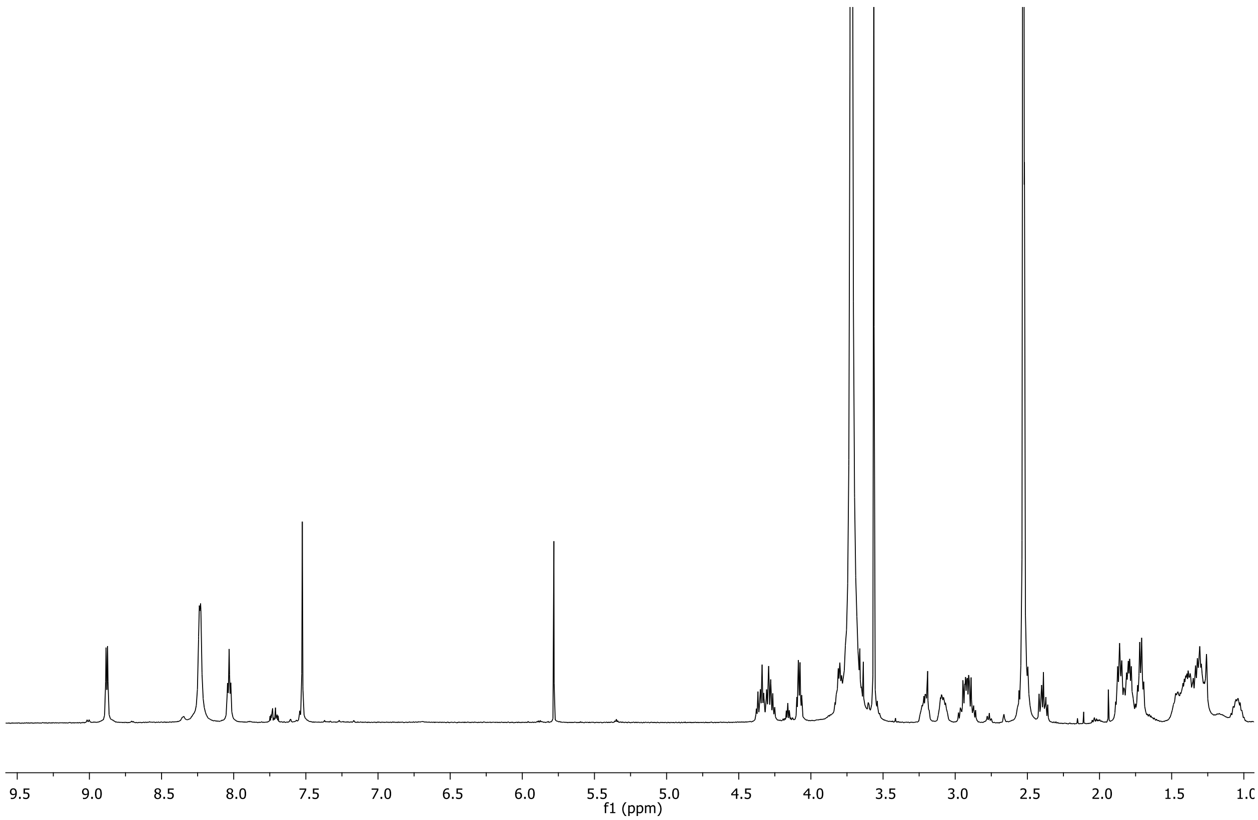
9.5
9.0
8.5
8.0
7.5
7.0
6.5
6.0
5.5
5.0
4.5
4.0
3.5
3.0
2.5
2.0
1.5
1.0
f1 (ppm)

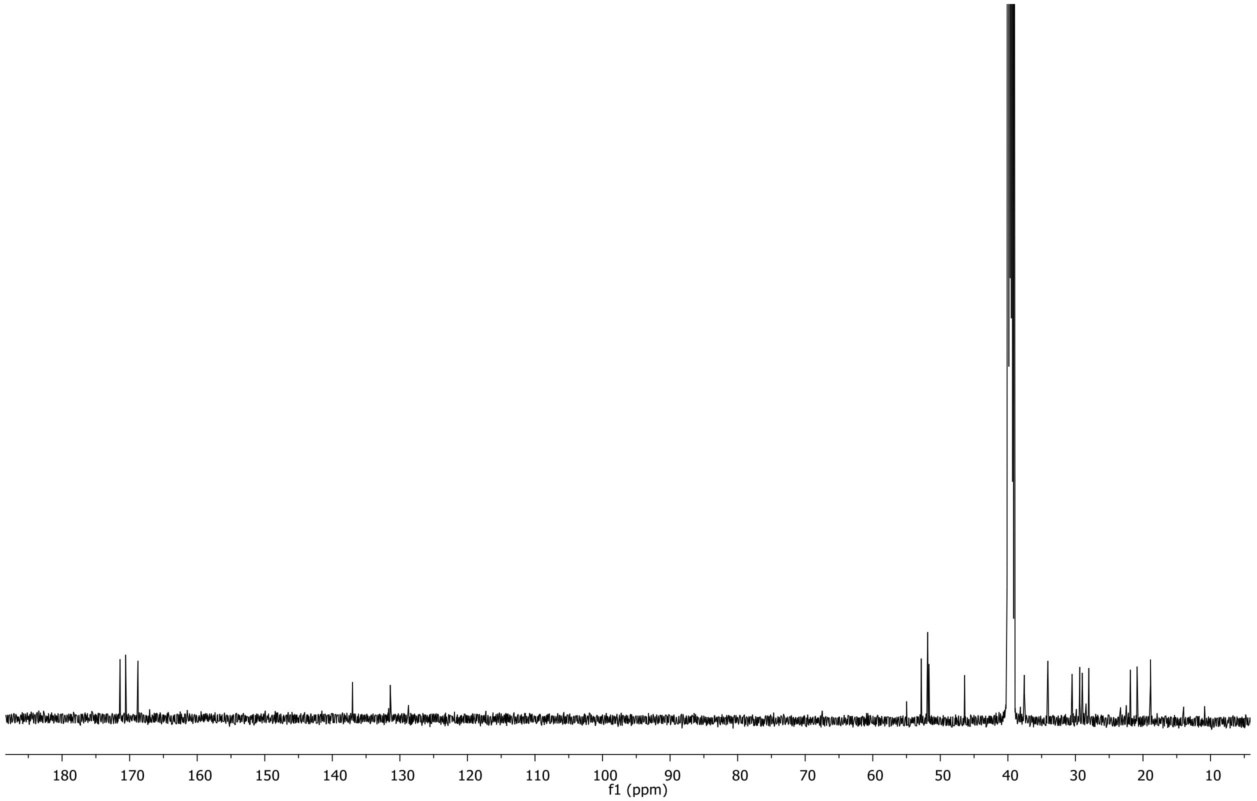
180
170
160
150
140
130
120
110
100
90
80
70
60
50
40
30
20
10
f1 (ppm)

E1x

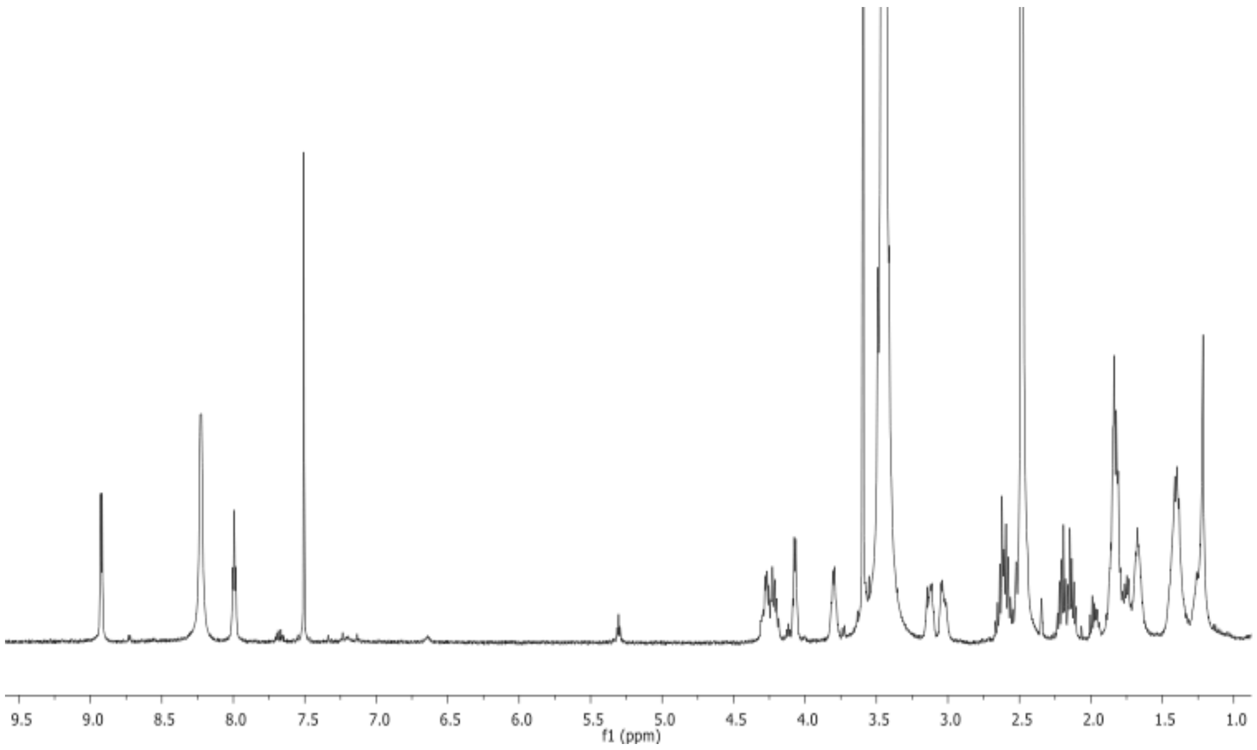
9.5
9.0
8.5
8.0
7.5
7.0
6.5
6.0
5.5
5.0
4.5
4.0
3.5
3.0
2.5
2.0
1.5
1.0
f1 (ppm)

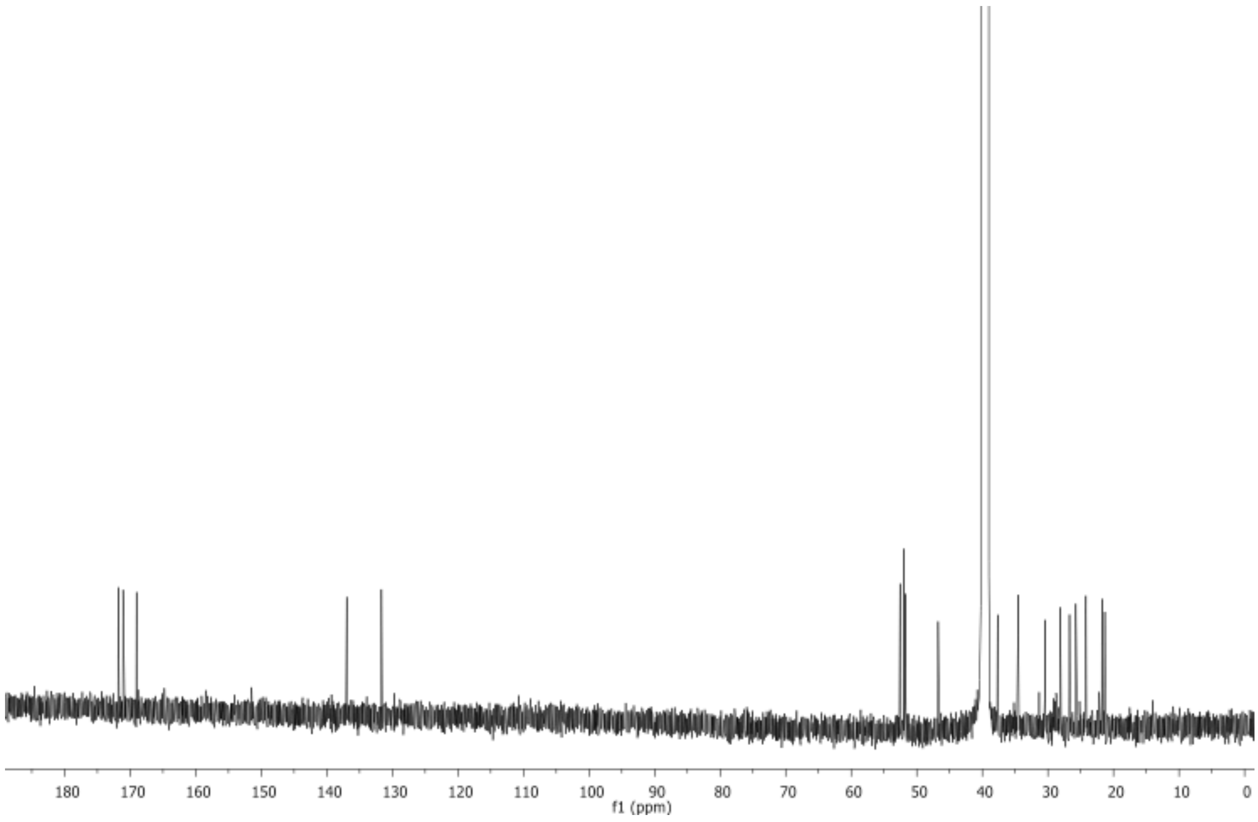
180
170
160
150
140
130
120
110
100
90
80
70
60
50
40
30
20
10
0
f1 (ppm)

E3x

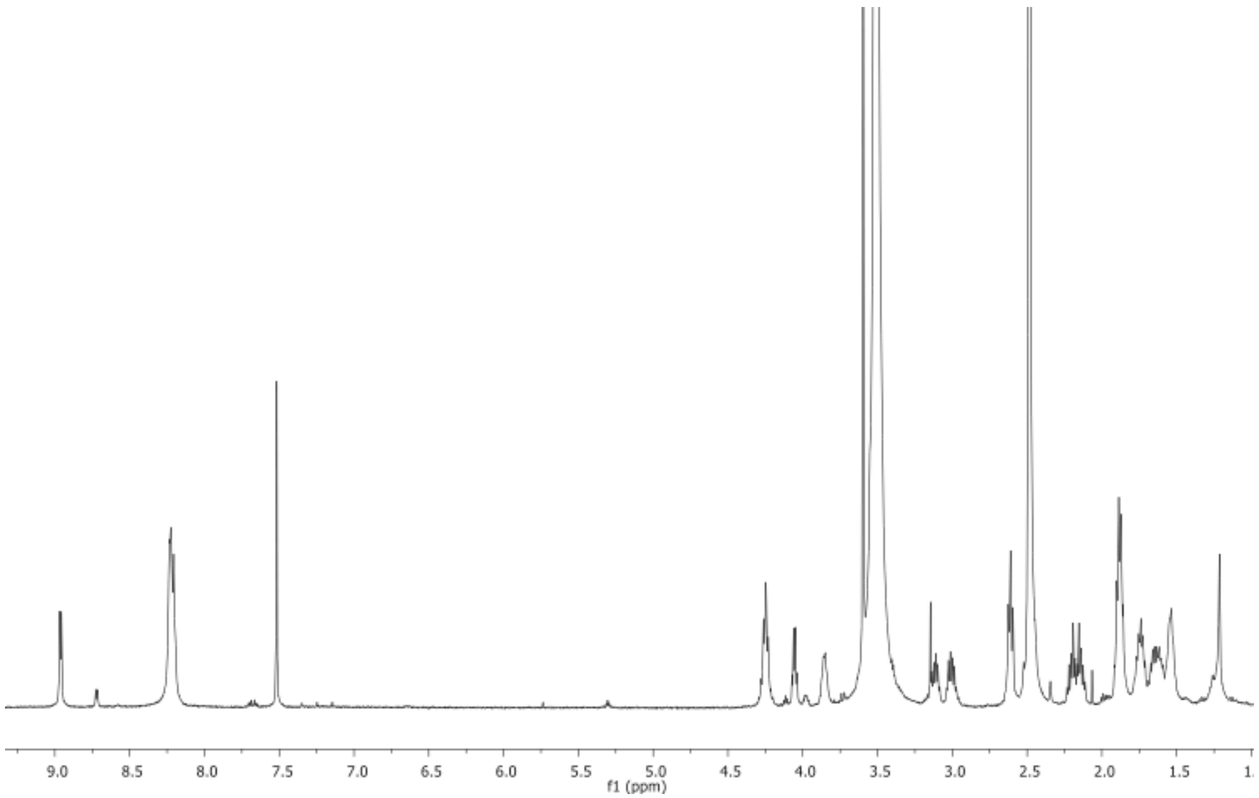

9.0
8.5
8.0
7.5
7.0
6.5
6.0
5.5
5.0
f1 (ppm)
4.5
4.0
3.5
3.0
2.5
2.0
1.5

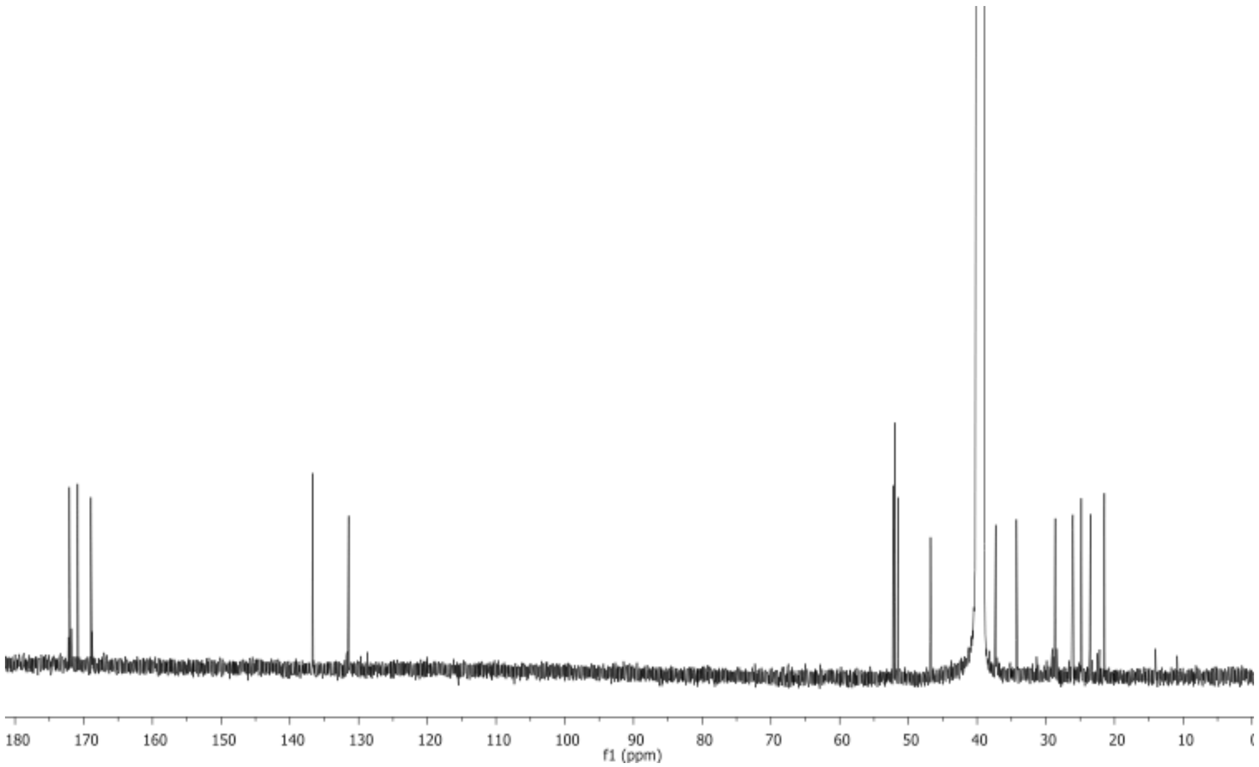

180
170
160
150
140
130
120
110
100
90
f1 (ppm)
80
70
60
50
40
30
20
10

E7x

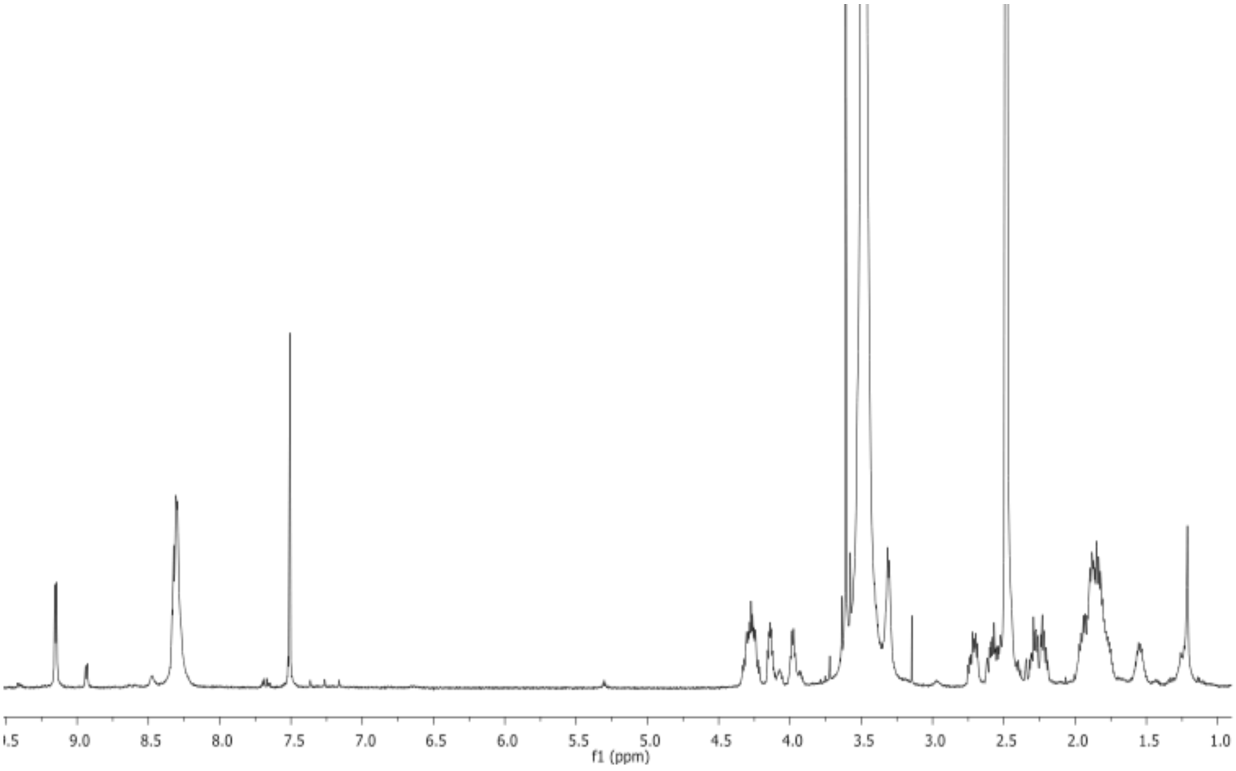

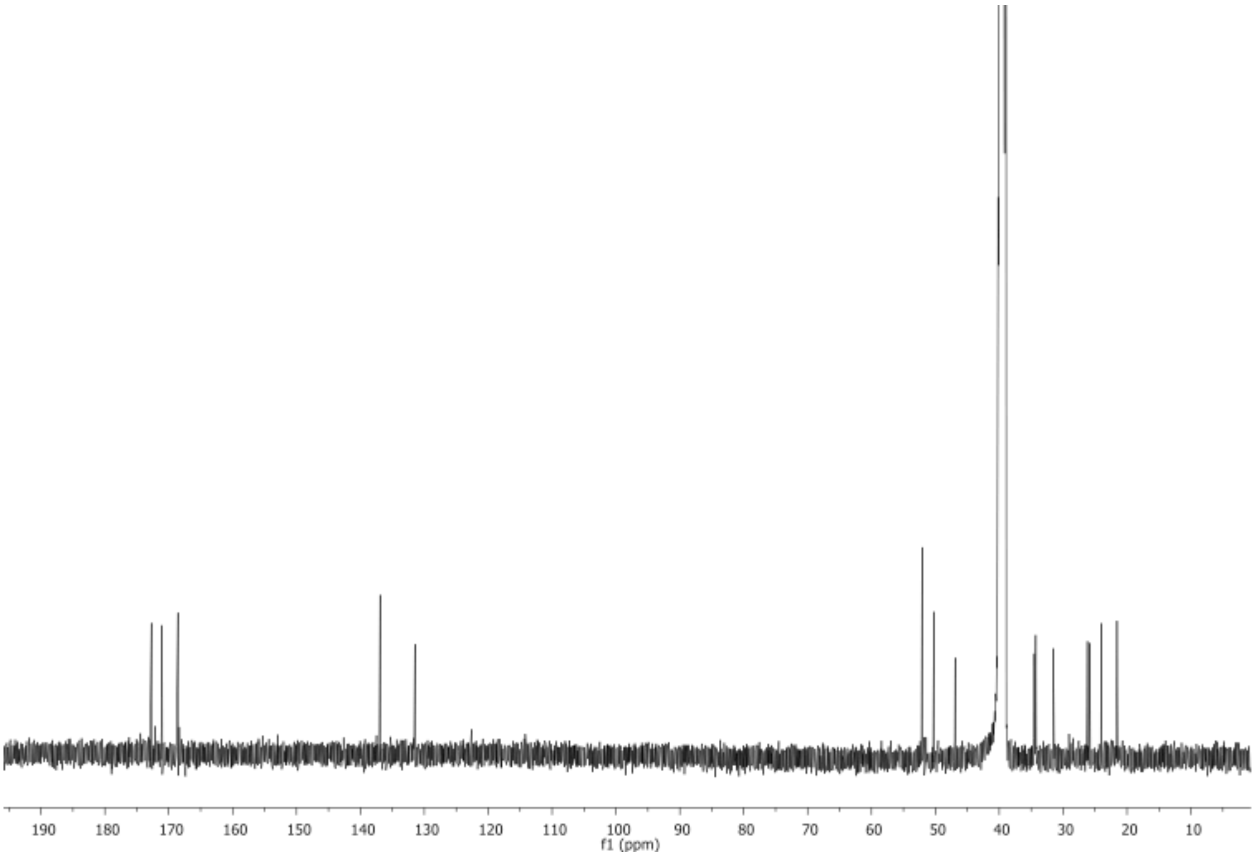

B2y

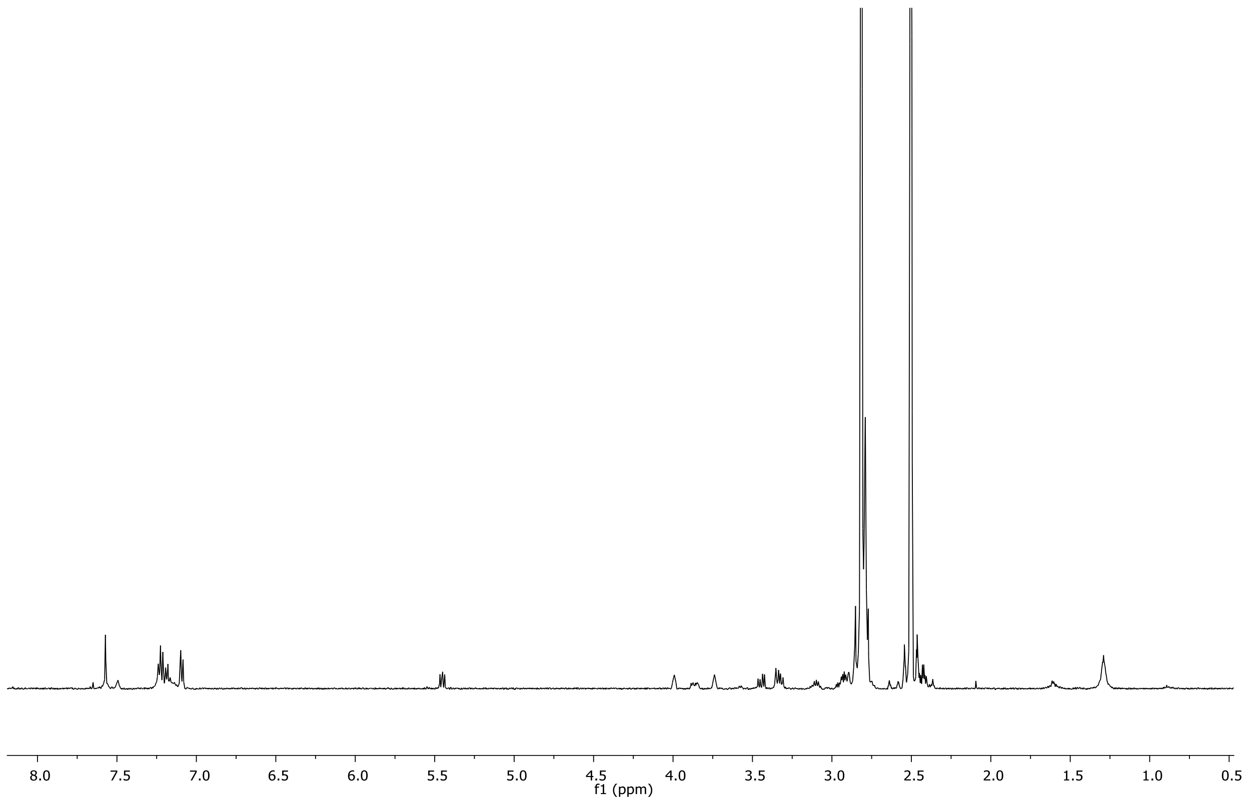

8.0
7.5
7.0
6.5
6.0
5.5
5.0
4.5
4.0
3.5
3.0
2.5
2.0
1.5
1.0
0.5
f1 (ppm)

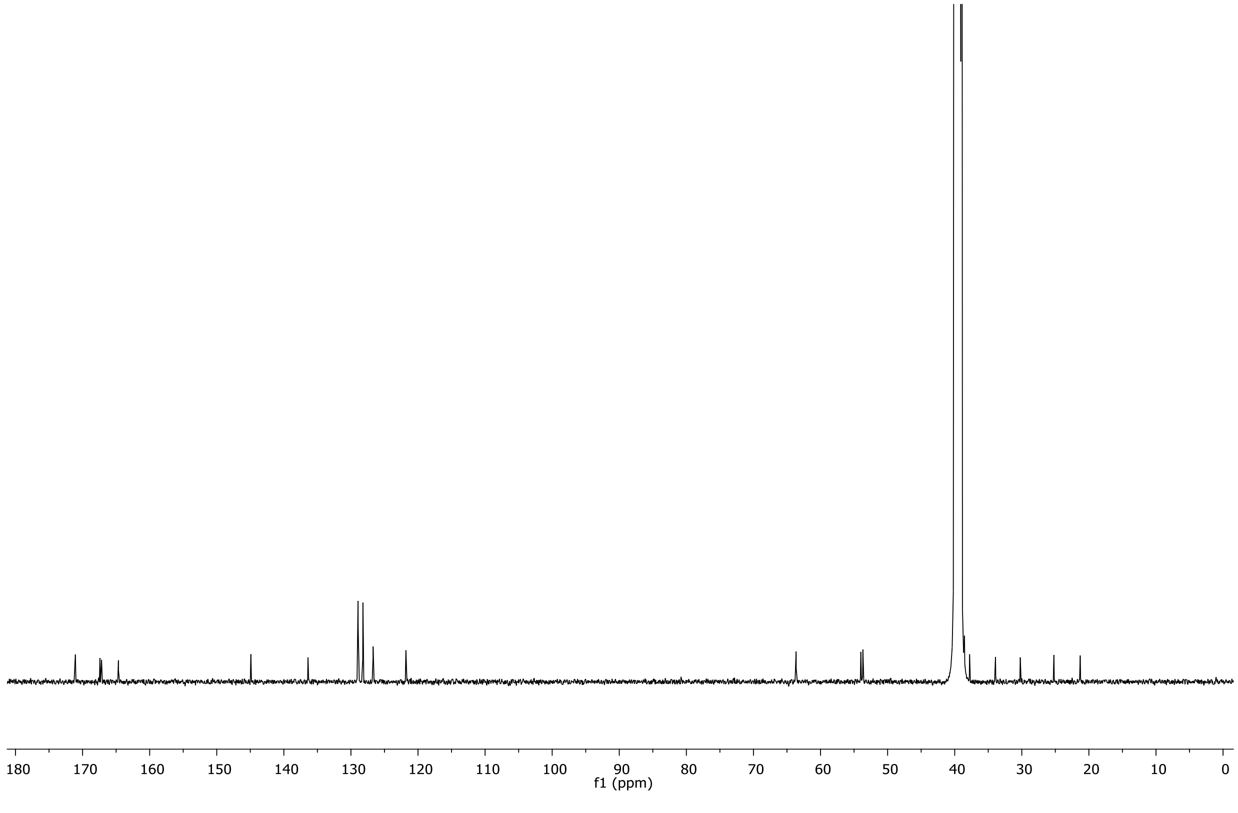

180
170
160
150
140
130
120
110
100
90
80
70
60
50
40
30
20
10
0
f1 (ppm)

B2z

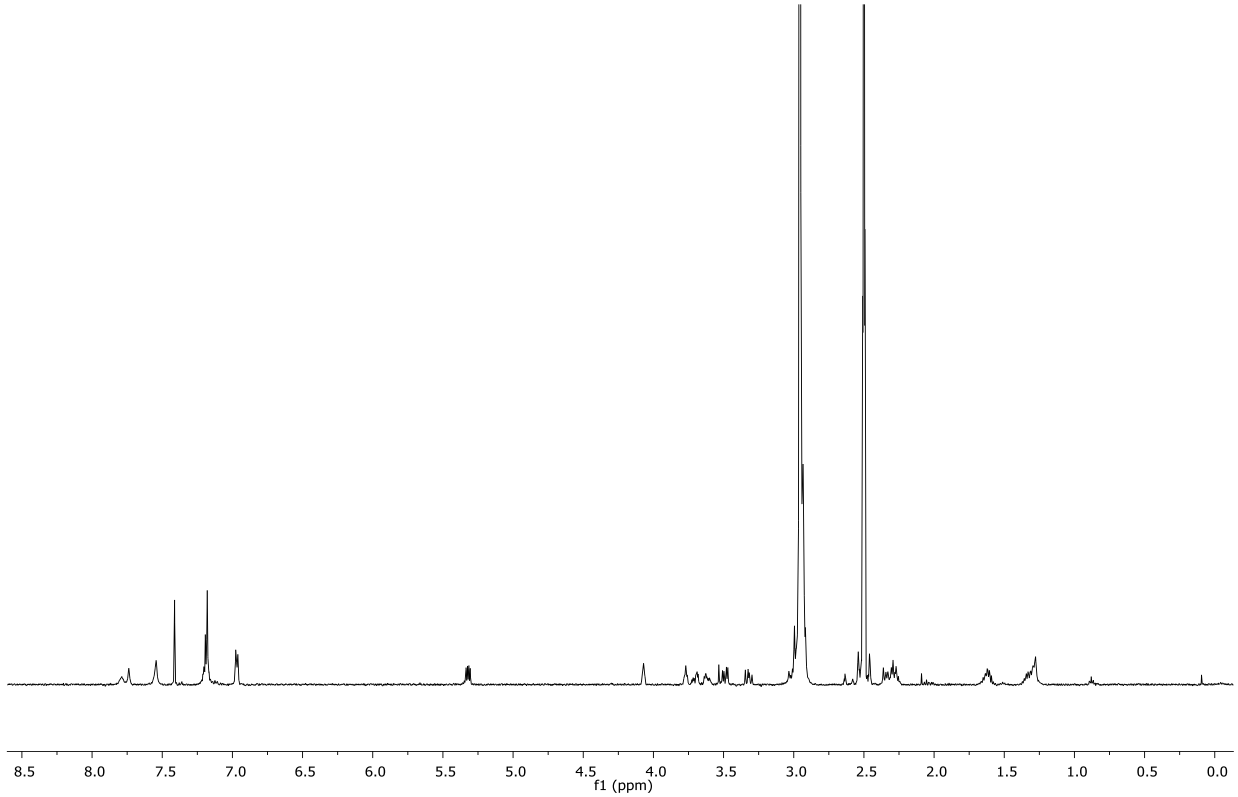
8.5
8.0
7.5
7.0
6.5
6.0
5.5
5.0
4.5
4.0
3.5
3.0
2.5
2.0
1.5
1.0
0.5
0.0
f1 (ppm)

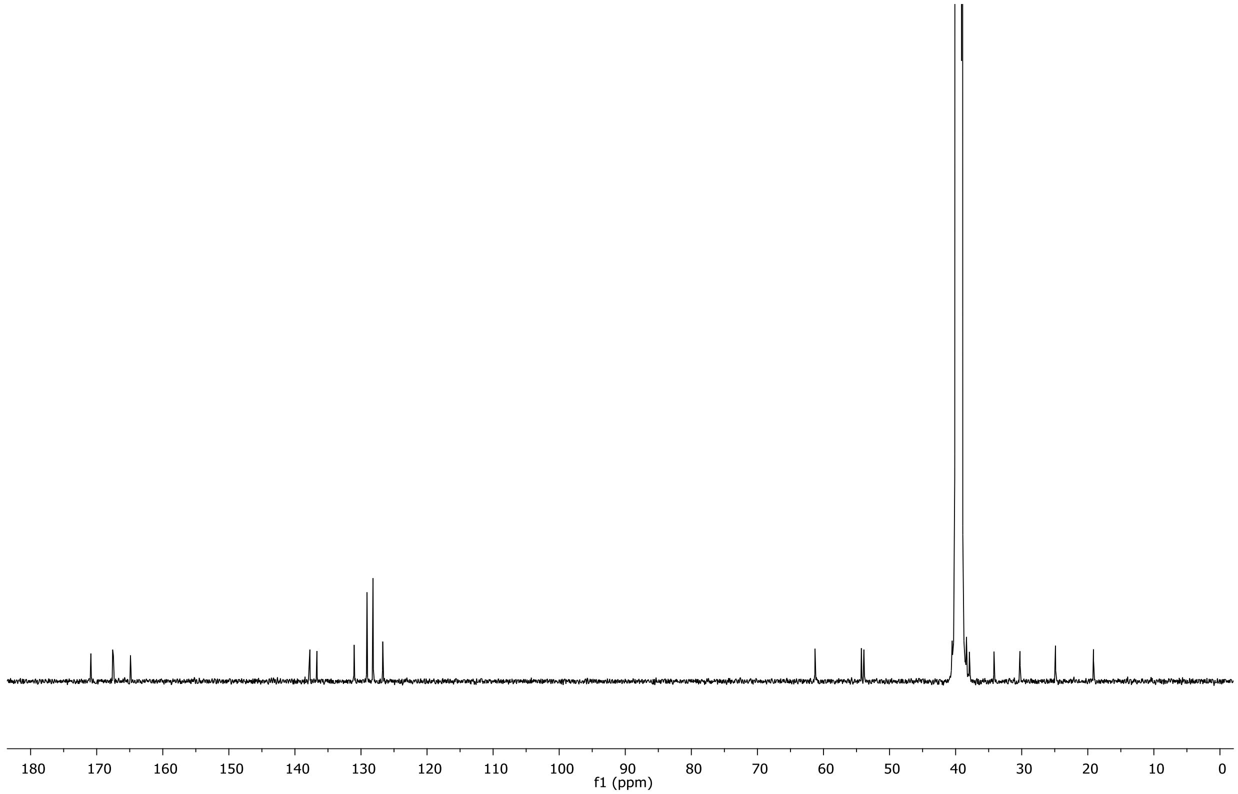
180
170
160
150
140
130
120
110
100
90
80
70
60
50
40
30
20
10
0
f1 (ppm)

B5y

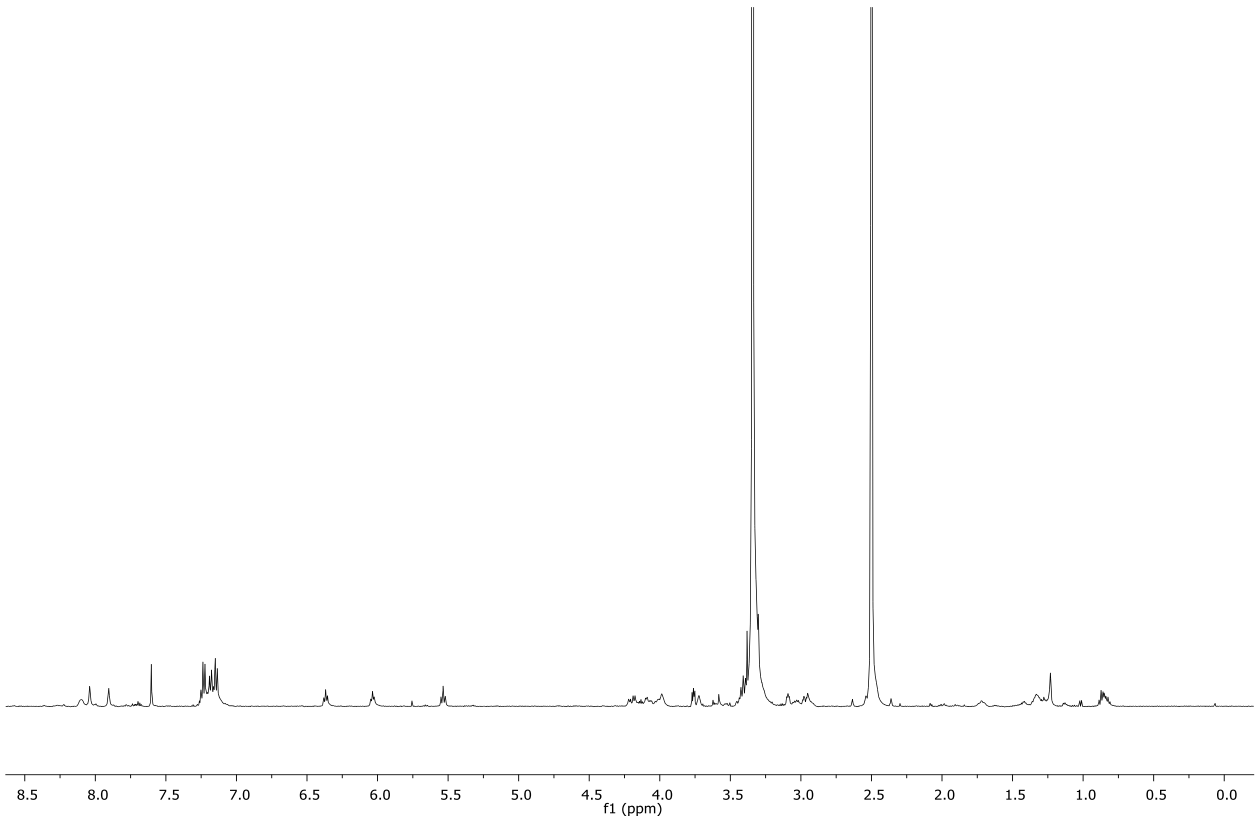
8.5
8.0
7.5
7.0
6.5
6.0
5.5
5.0
4.5
4.0
3.5
3.0
2.5
2.0
1.5
1.0
0.5
0.0
f1 (ppm)

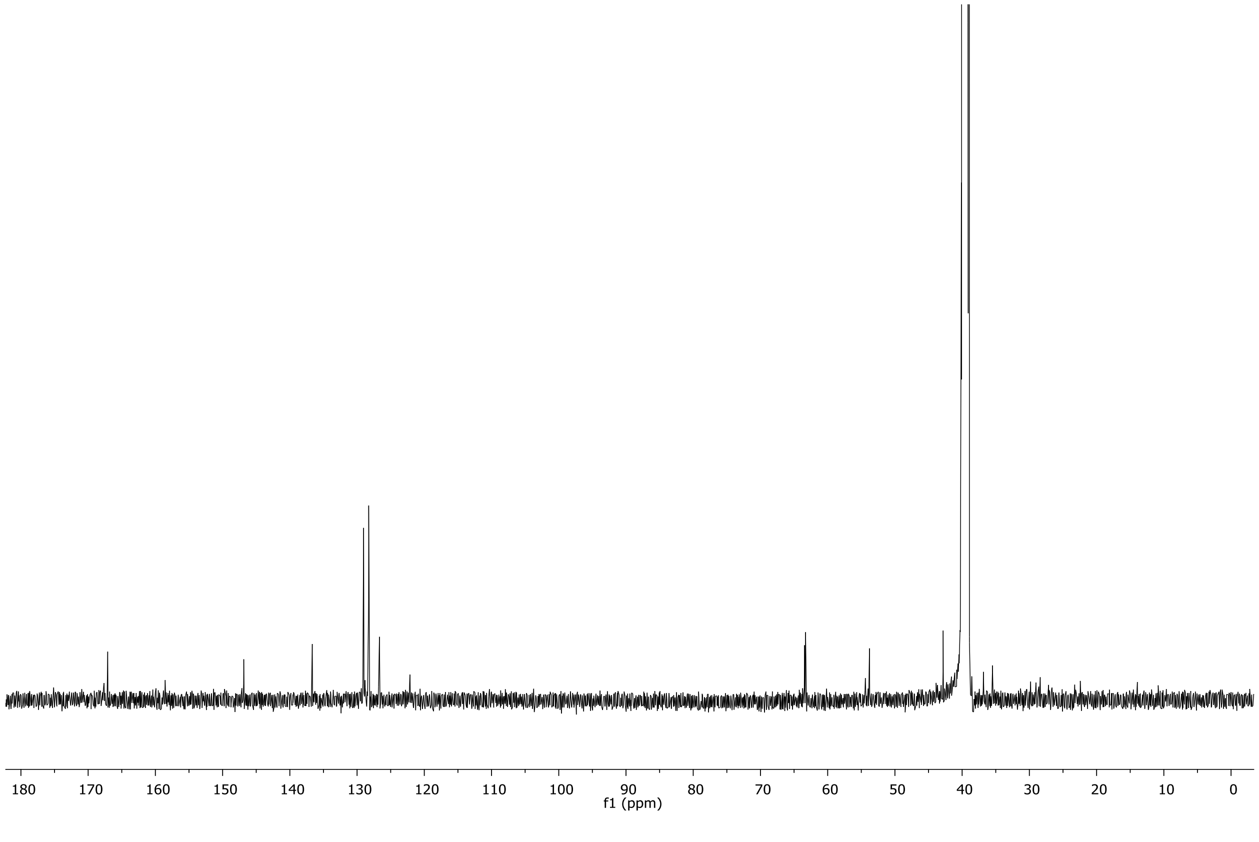
180
170
160
150
140
130
120
110
100
90
80
70
60
50
40
30
20
10
0
f1 (ppm)

B8y

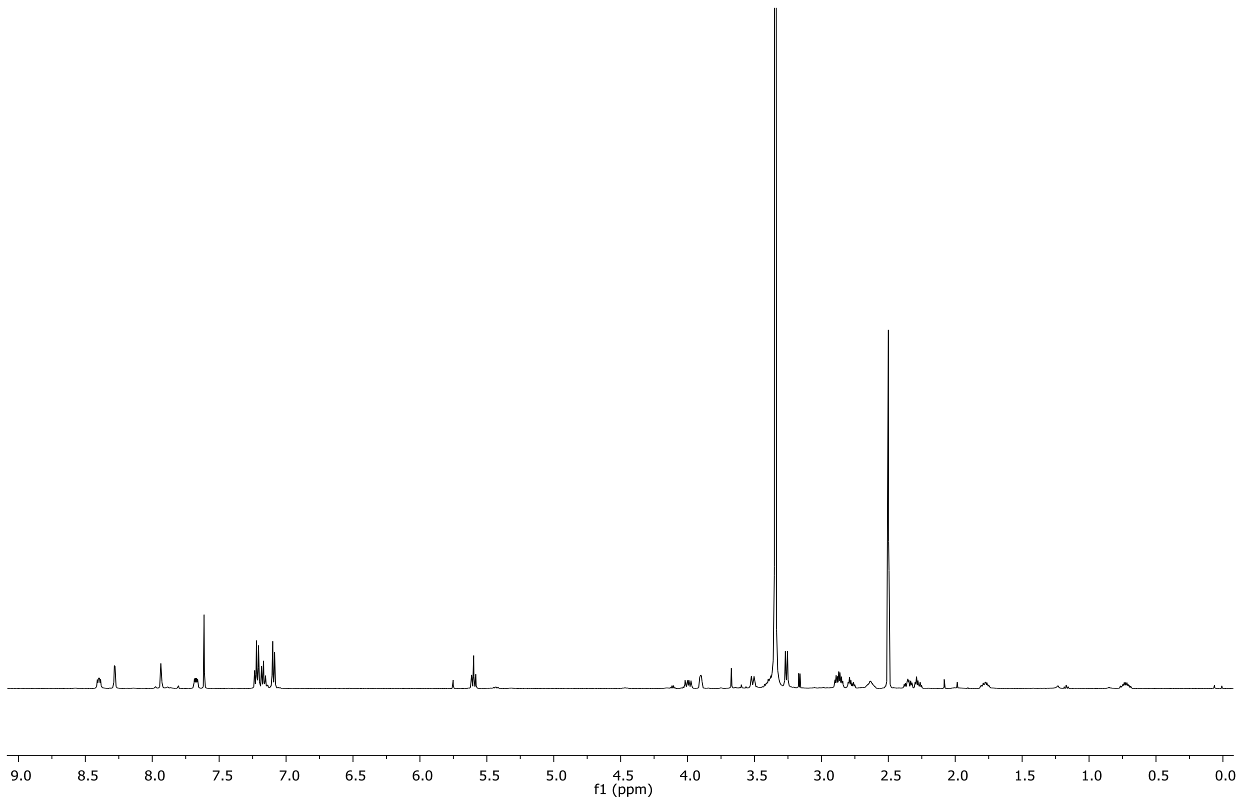
9.0
8.5
8.0
7.5
7.0
6.5
6.0
5.5
5.0
4.5
4.0
3.5
3.0
2.5
2.0
1.5
1.0
0.5
0.0
f1 (ppm)

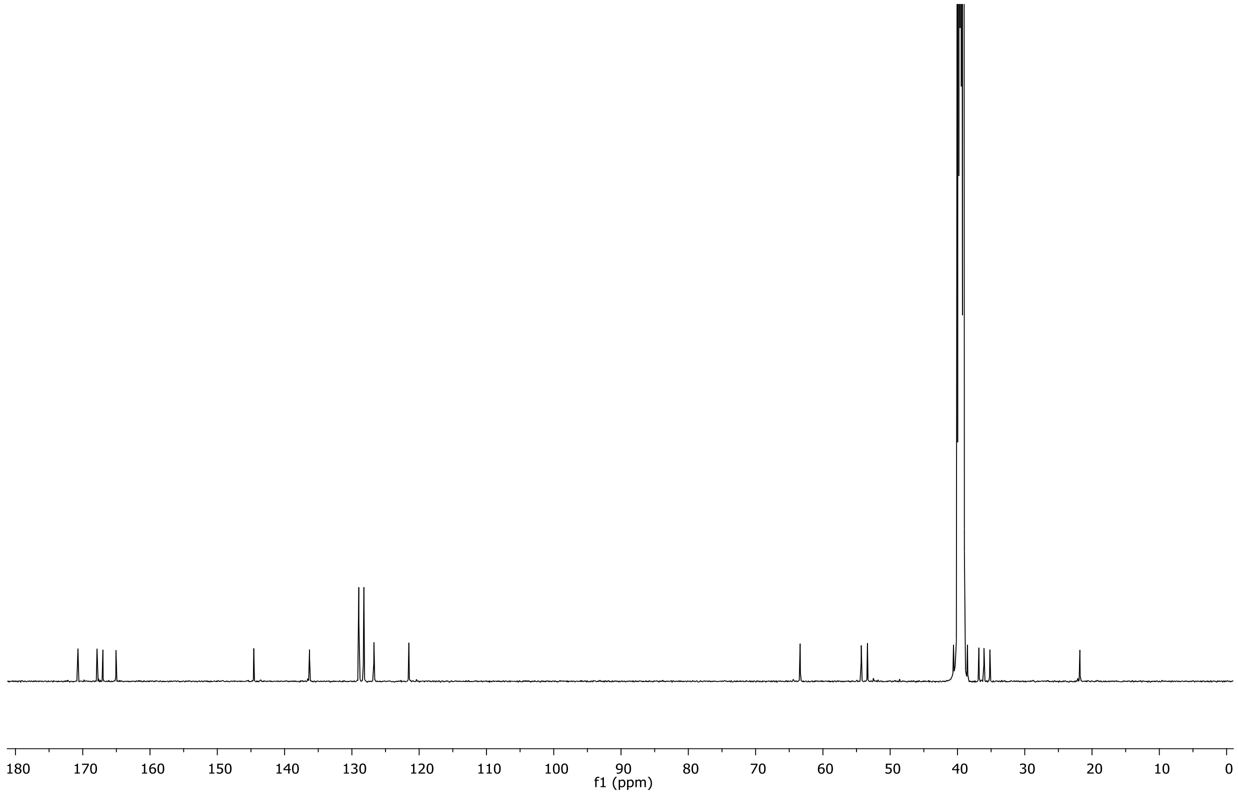
180
170
160
150
140
130
120
110
100
90
80
70
60
50
40
30
20
10
0
f1 (ppm)

B10z

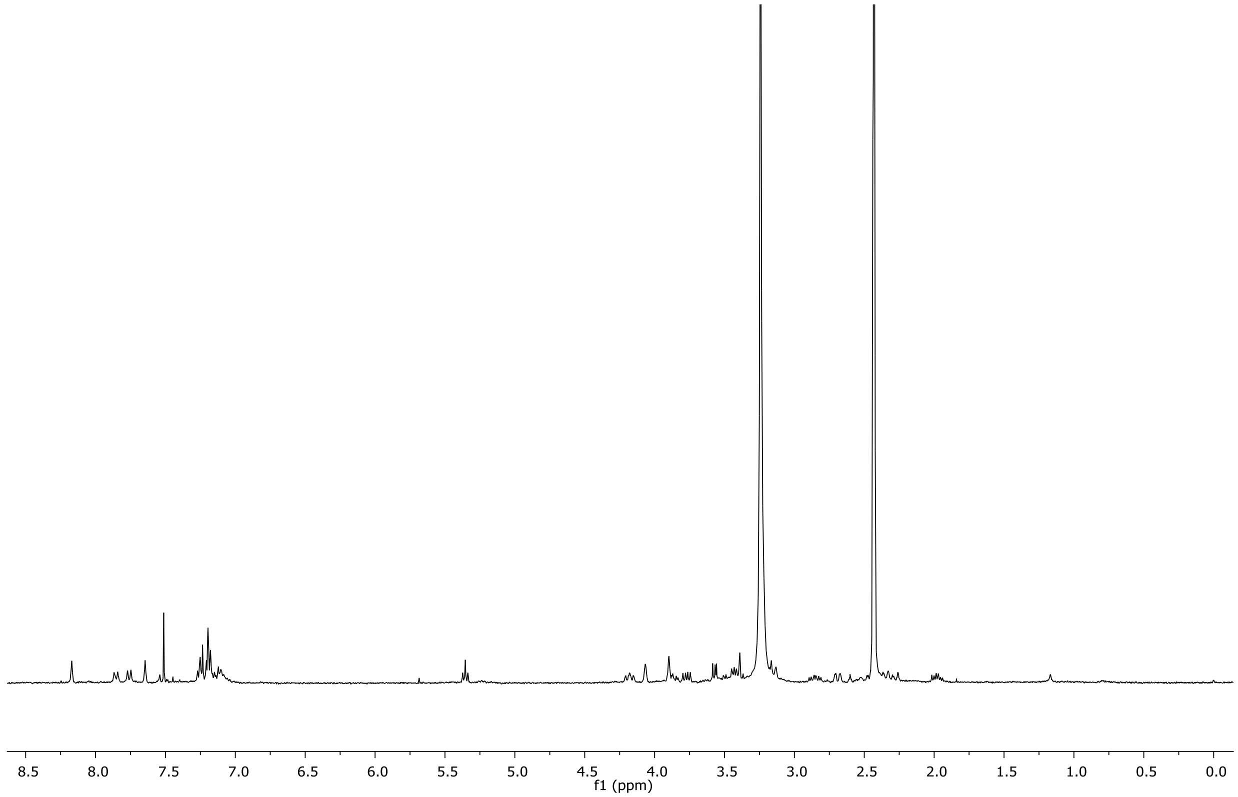

8.5 8.0 7.5 7.0 6.5 6.0 5.5 5.0 4.5 4.0 3.5 3.0 2.5 2.0 1.5 1.0 0.5 0.0
f1 (ppm)

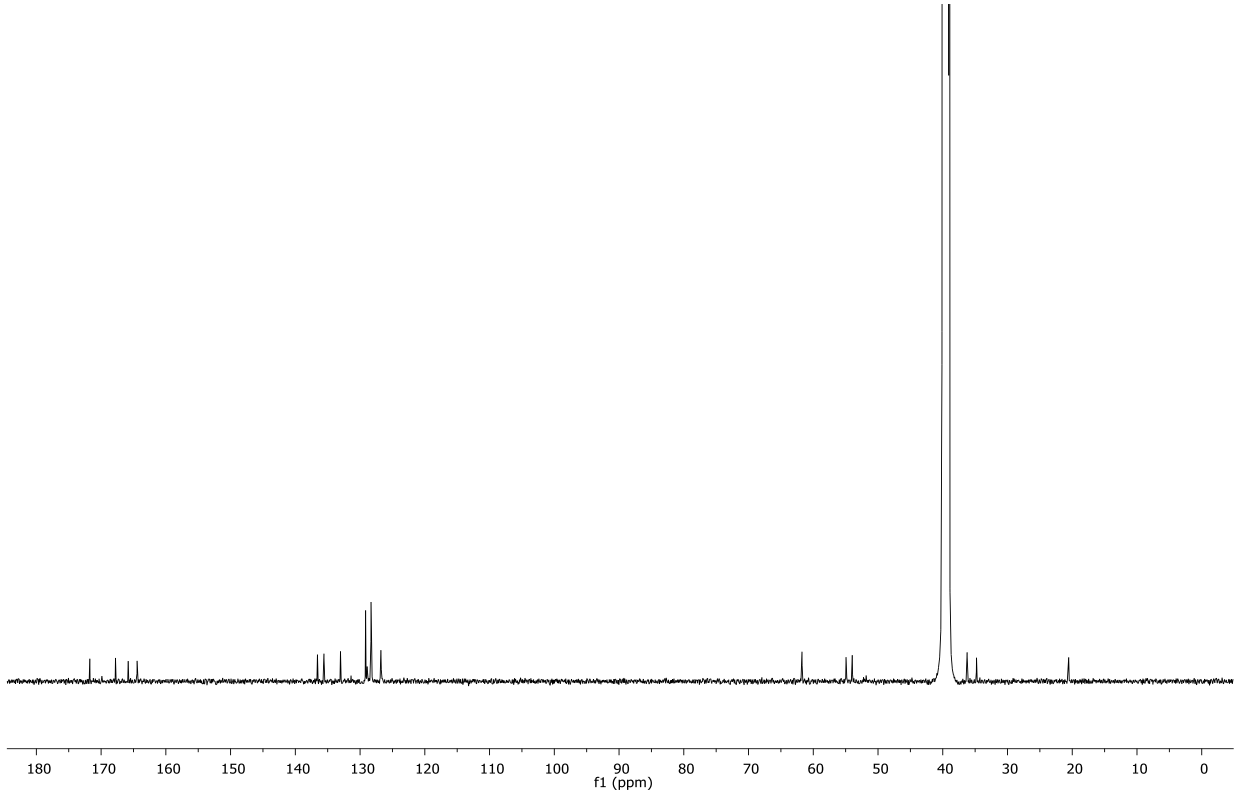

180 170 160 150 140 130 120 110 100 90 80 70 60 50 40 30 20 10 0
f1 (ppm)

B12z

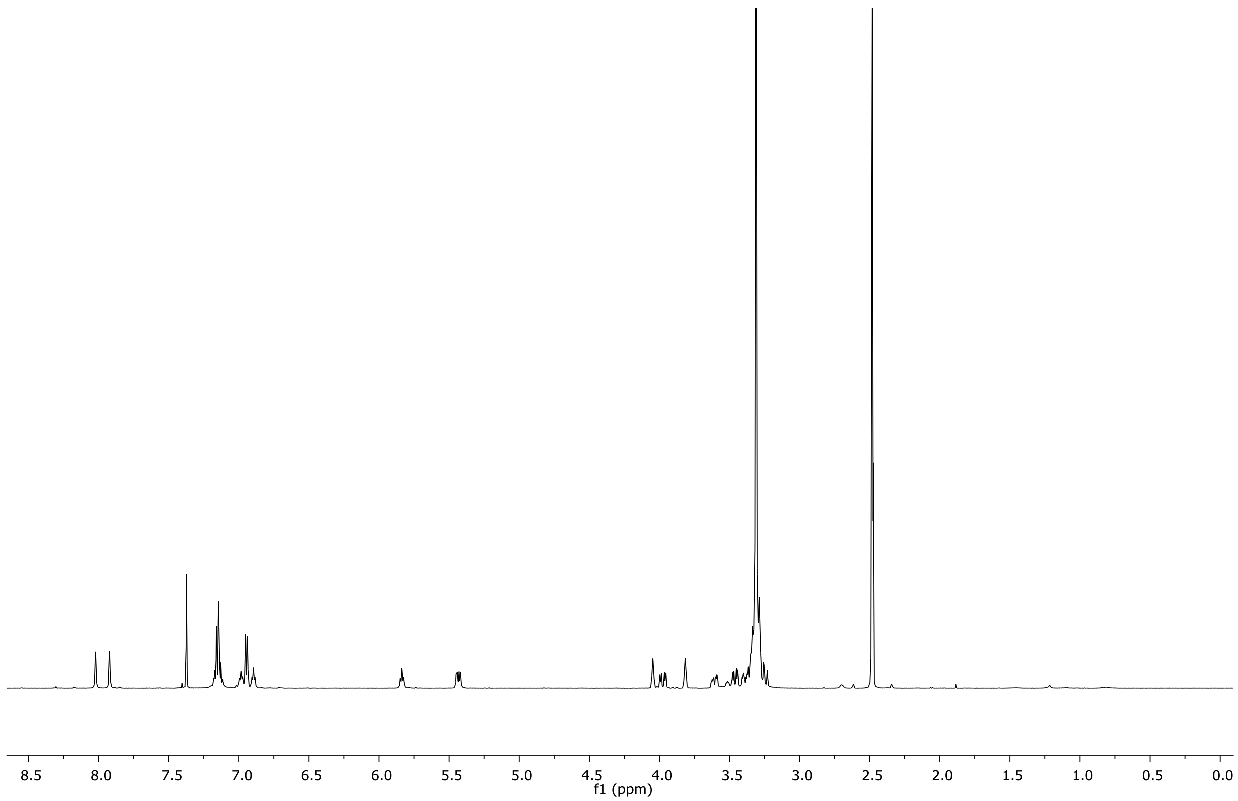

8.5
8.0
7.5
7.0
6.5
6.0
5.5
5.0
4.5
4.0
3.5
3.0
2.5
2.0
1.5
1.0
0.5
0.0
f1 (ppm)

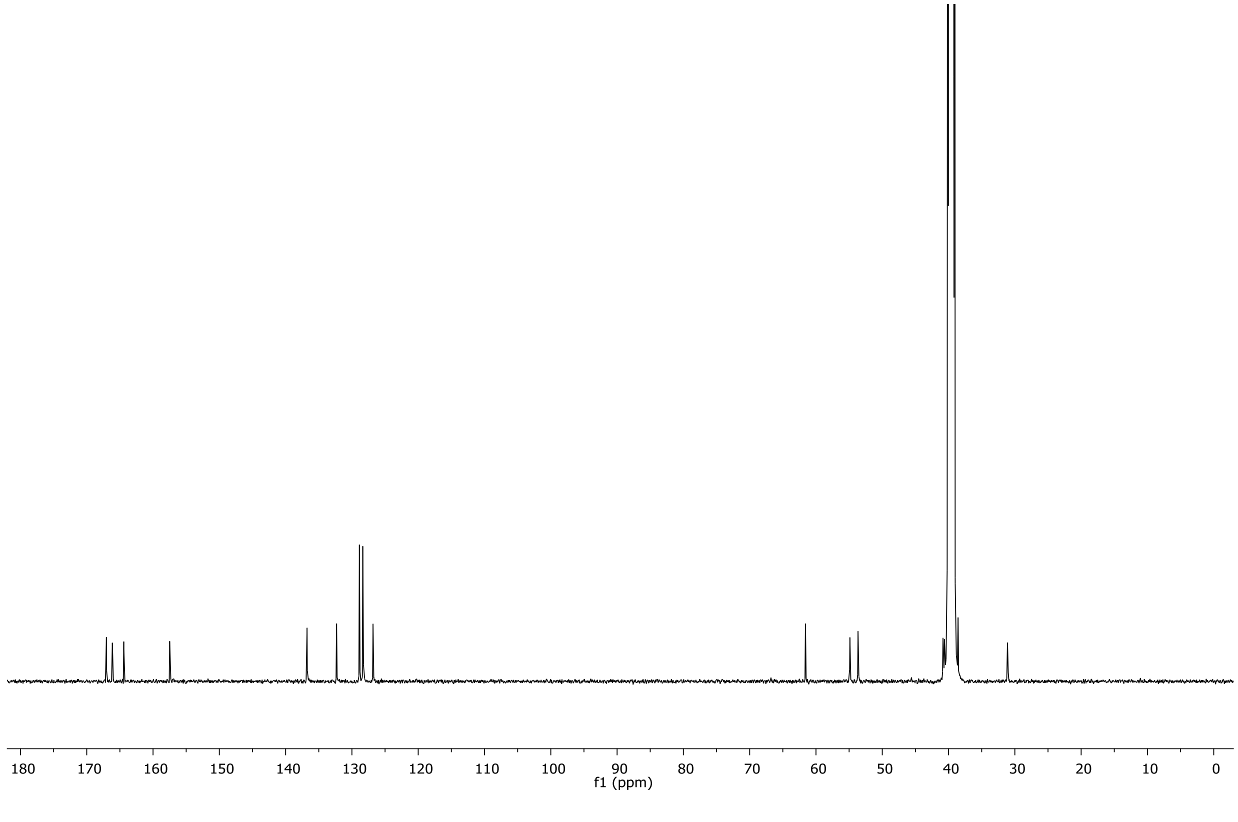

180
170
160
150
140
130
120
110
100
90
80
70
60
50
40
30
20
10
0
f1 (ppm)

B14y

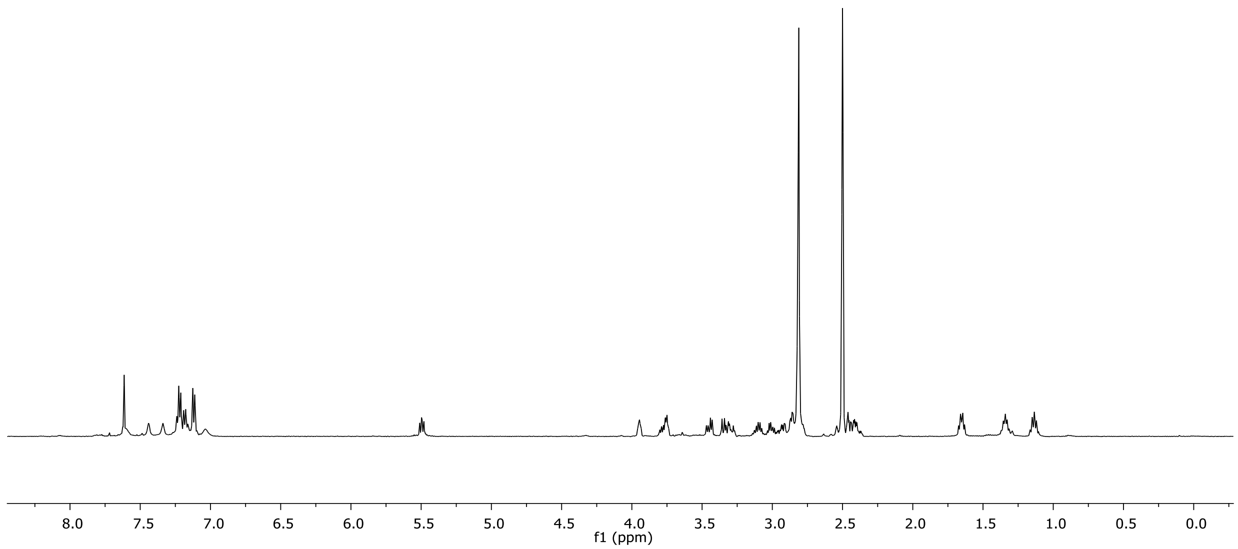

8.0
7.5
7.0
6.5
6.0
5.5
5.0
4.5
4.0
3.5
3.0
2.5
2.0
1.5
1.0
0.5
0.0
f1 (ppm)

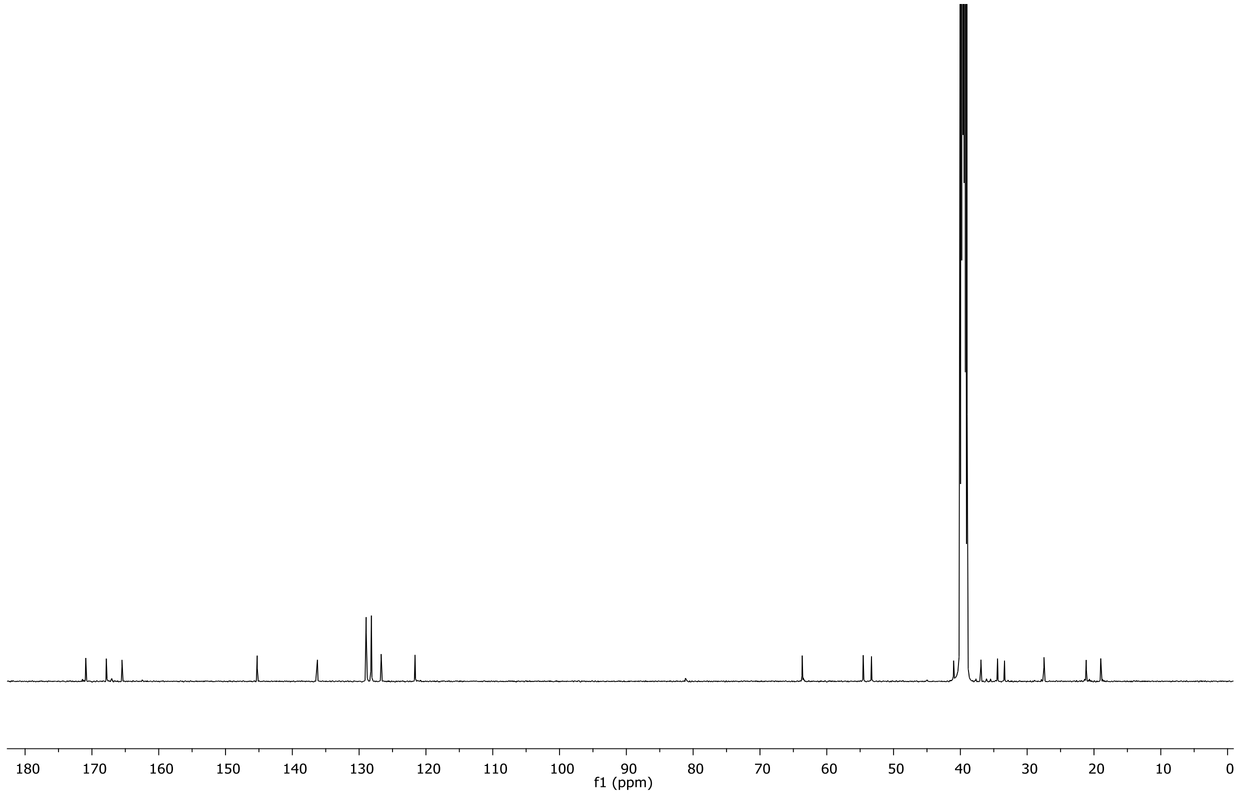

180
170
160
150
140
130
120
110
100
90
80
70
60
50
40
30
20
10
0
f1 (ppm)

B14z

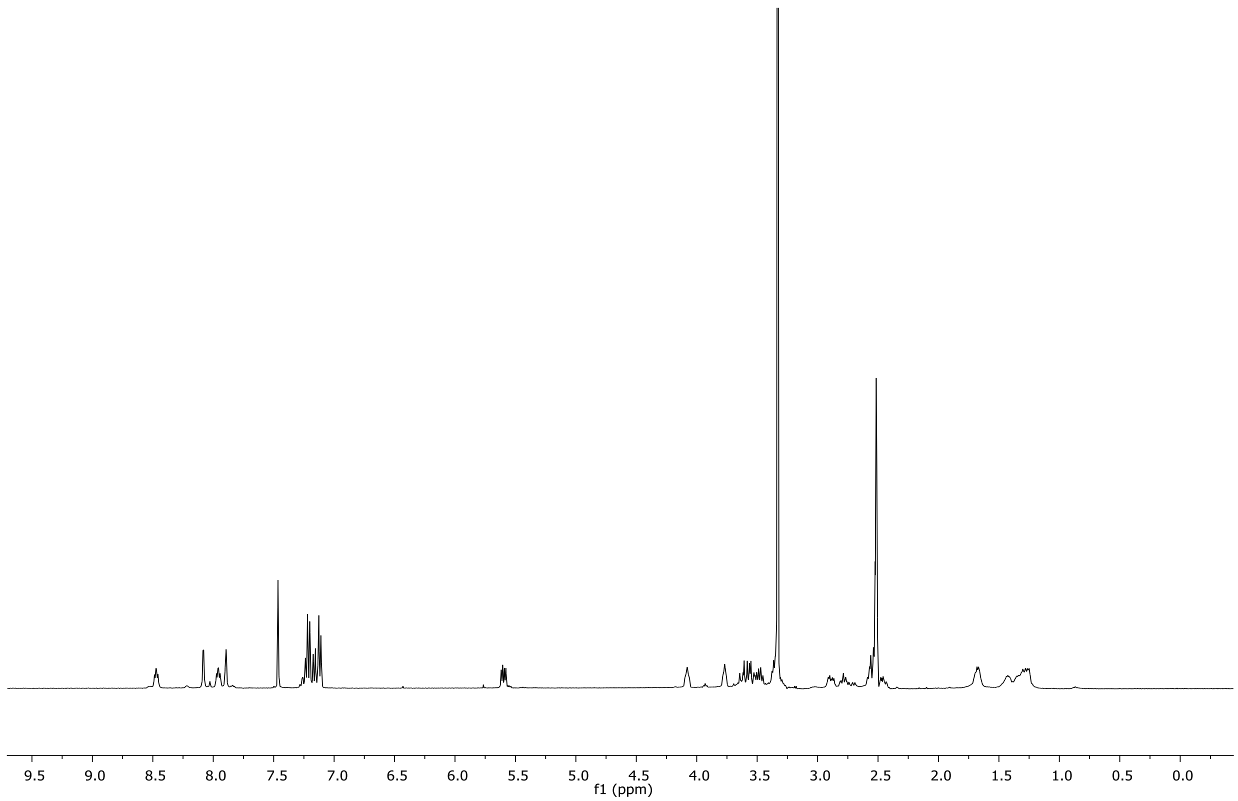

9.5 9.0 8.5 8.0 7.5 7.0 6.5 6.0 5.5 5.0 4.5 4.0 3.5 3.0 2.5 2.0 1.5 1.0 0.5 0.0
f1 (ppm)

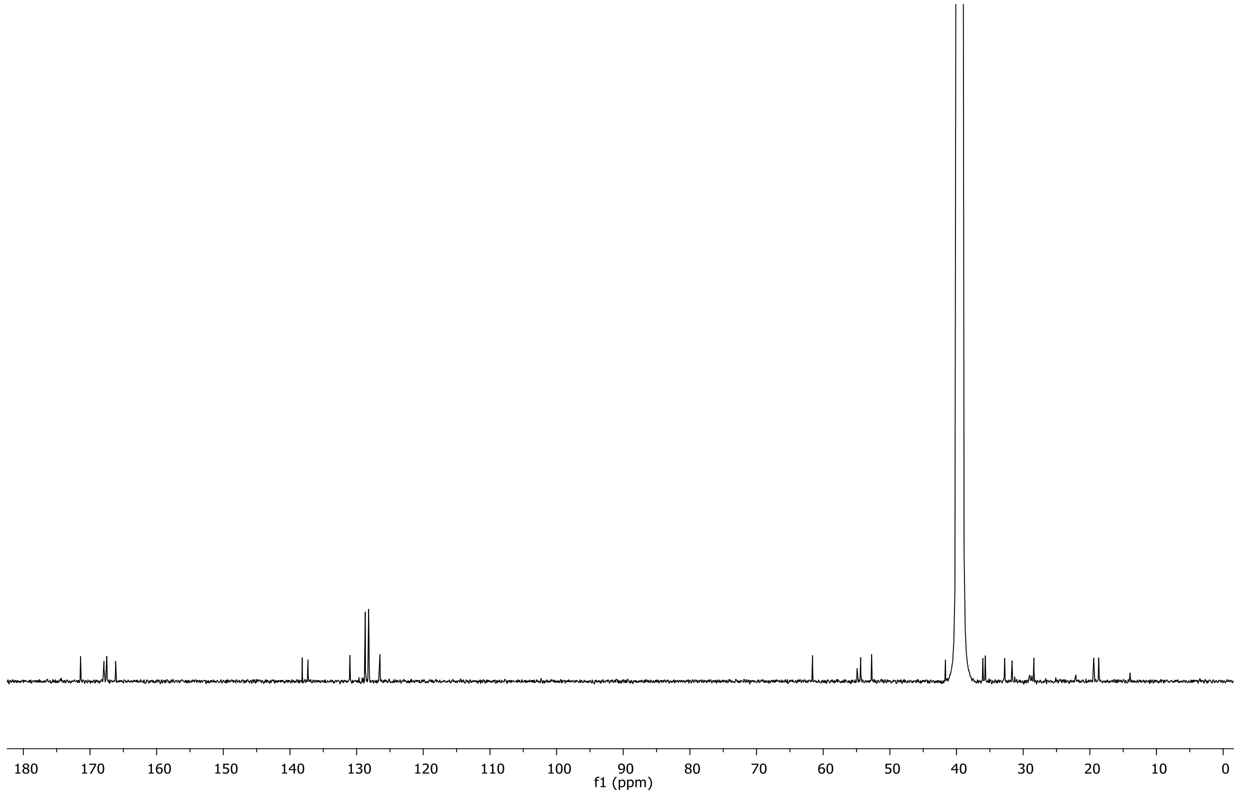

180 170 160 150 140 130 120 110 100 90 80 70 60 50 40 30 20 10 0
f1 (ppm)

D14z

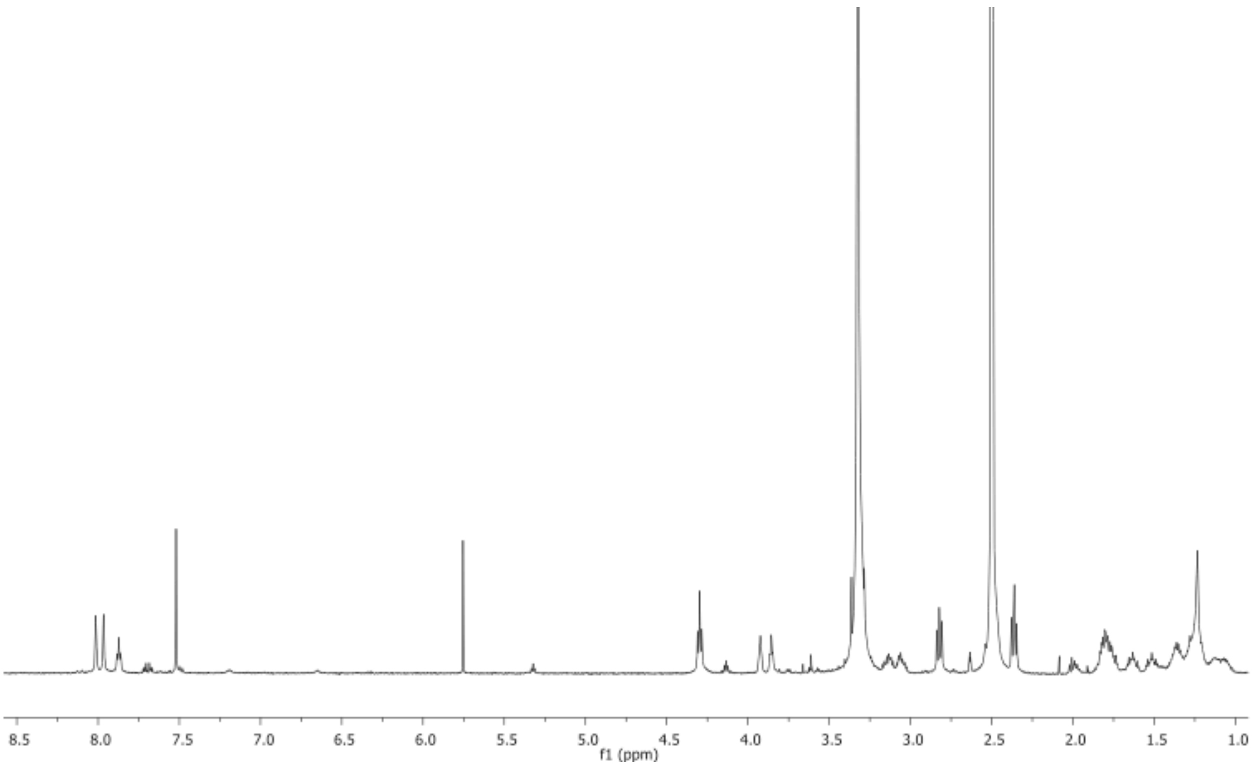
8.5
8.0
7.5
7.0
6.5
6.0
5.5
5.0
4.5
4.0
3.5
3.0
2.5
2.0
1.5
1.0
f1 (ppm)

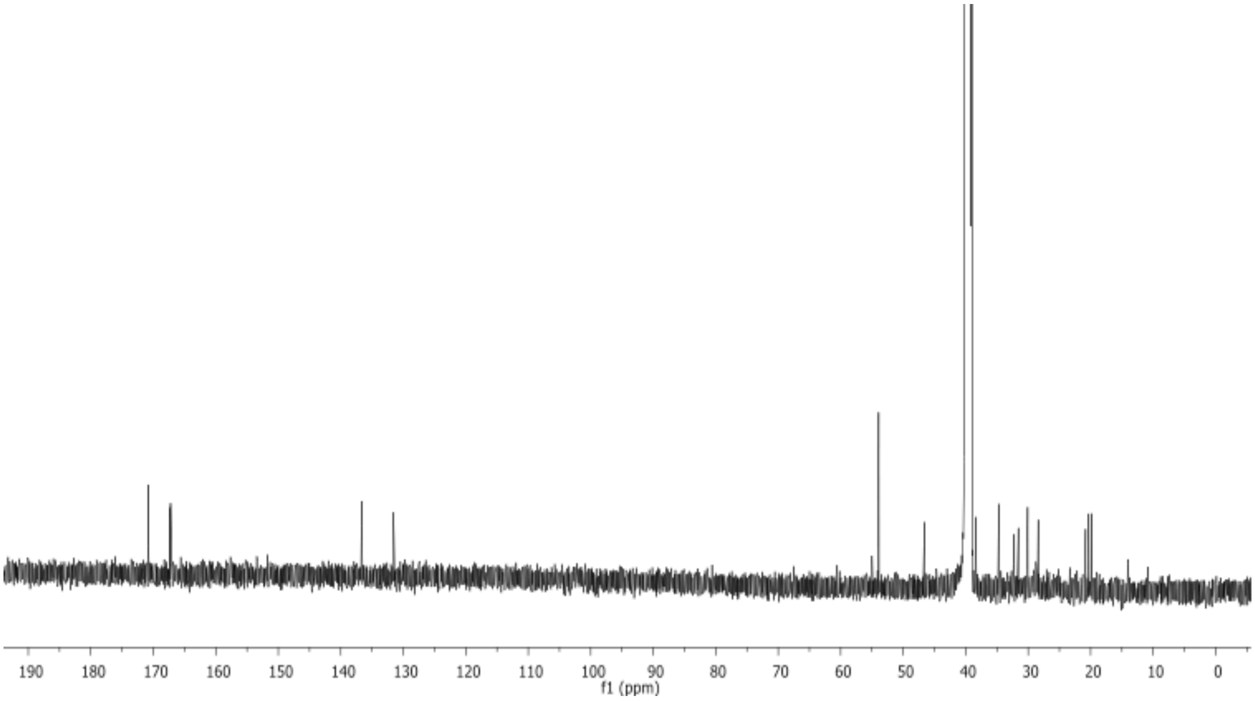
190
180
170
160
150
140
130
120
110
100
90
80
70
60
50
40
30
20
10
0
f1 (ppm)
